# Exploring Fish Parvalbumins through Allergen Names and Gene Identities

**DOI:** 10.3390/genes15101337

**Published:** 2024-10-18

**Authors:** Johannes M. Dijkstra, Annette Kuehn, Eiji Sugihara, Yasuto Kondo

**Affiliations:** 1 Center for Medical Science, Fujita Health University, Toyoake 470-1192, Japan; dijkstra@fujita-hu.ac.jp; 2 Department of Infection and Immunity, Luxembourg Institute of Health, L-4354 Esch-sur-Alzette, Luxembourg; annette.kuehn@lih.lu; 3 Laboratory of Genome Sequencing and Analysis, Open Facility Center, Fujita Health University, Toyoake 470-1192, Japan; eiji.sugihara@fujita-hu.ac.jp; 4 Department of Pediatrics, Fujita Health University Bantane Hospital, Nagoya 454-8509, Japan

**Keywords:** fish, food allergens, parvalbumin, nomenclature, molecular identity, multi-gene complexity

## Abstract

Parvalbumins are the main source of food allergies in fish meat, with each fish possessing multiple different parvalbumins. The naming convention of these allergens in terms of allergen codes (numbers) is species-specific. Allergen codes for parvalbumin isoallergens and allergen variants are based on sequence identities relative to the first parvalbumin allergen discovered in that particular species. This means that parvalbumins with similar allergen codes, such as catfish Pan h 1.0201 and redfish Seb m 1.0201, are not necessarily the most similar proteins, or encoded by the same gene. Here, we aim to elucidate the molecular basis of parvalbumins. We explain the complicated genetics of fish parvalbumins in an accessible manner for fish allergen researchers. Teleost or modern bony fish, which include most commercial fish species, have varying numbers of up to 22 parvalbumin genes. All have derived from ten parvalbumin genes in their common ancestor. We have named these ten genes “*parvalbumin 1*-to-*10*” (*PVALB1*-to-*PVALB10*), building on earlier nomenclature established for zebrafish. For duplicated genes, we use variant names such as, for example, “*PVALB2A* and *PVALB2B*”. As illustrative examples of our gene identification system, we systematically analyze all parvalbumin genes in two common allergy-inducing species in Japan: red seabream (*Pagrus major*) and chum salmon (*Oncorhynchus keta*). We also provide gene identifications for known parvalbumin allergens in various fish species.

## 1. Introduction

**Figure 1:**
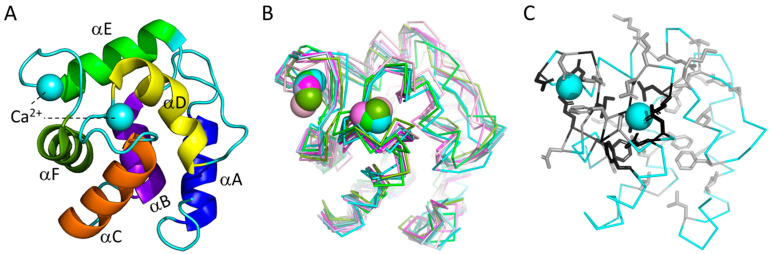
Parvalbumins have six α-helices A-to-F and two “EF-hand” domains for binding Ca^2+^ ions (indicated as spheres). (**A**) The structure, in cartoon format, of common carp pvalb4_(Chr.A3) (a β2-parvalbumin; PDB accession 4CPV) [7], which was the first parvalbumin of which the structure was elucidated [8]. Different α-helices are in different colors. (**B**) Superimposition of various parvalbumin structures, in ribbon format, reveals a common structure. Light pink, human α-parvalbumin (1RK9); pink, pike pvalb7 α-parvalbumin (2PAS); magenta, spotless smooth-hound shark SPV-I α-parvalbumin (5ZGM); green, human oncomodulin (1TTX); splitpea green, chicken CPV3-oncomodulin (2KYF); soft purple, chicken ATH β2-parvalbumin (3FS7); cyan, Atlantic cod pvalb2 β2-parvalbumin (2MBX); green cyan, pike pvalb3 β2-parvalbumin (1PVB); aquamarine, common carp pvalb4_(Chr.A3) β2-parvalbumin (4CPV); light teal, spotless smooth-hound shark SPV-II β2-parvalbumin (5ZH6). (**C**) The structure, in ribbon format, of common carp pvalb4_(Chr.A3) (PDB accession 4CPV), shows in black those residues that are well conserved throughout EF-hand domain family molecules and in gray other residues that are well conserved throughout parvalbumins; the sidechains of these residues are shown in sticks format. This figure is used, with permission, from our open access article [9], and the figures were created with the help of Pymol 2.5.2 software (https://pymol.org/2/ (accessed on 27 October 2022)).

If people are allergic to fish meat, this is usually caused by IgE-mediated responses to parvalbumins, the major allergens [1]. Parvalbumins are small proteins, typically comprising 108–109 amino acids, which can bind calcium ions with high affinity (Figure 1A). They are believed to be Ca^2+^ buffer proteins in a variety of cell types, including muscle cells [2]. Fish parvalbumins were originally identified by protein purification from fishes, which contain considerable amounts of the allergen [3,4]. As allergens, they were first identified in the muscle of Atlantic cod (*Gadus morhua*) [5,6].

The nomenclature system for allergens is led by the World Health Organization/International Union of Immunological Societies (WHO/IUIS) Allergen Nomenclature Sub-committee. Approved allergens receive a unique allergen code derived from the abbreviation of the Latin name of the species (e.g., “Gad m” for *Gadus morhua*). This is followed by the number “1” for the first fish allergens discovered, i.e., parvalbumin, followed by a four-digit sub-numbering for isoallergens and allergen variants, also reflecting their order of detection in the particular species. The first two digits are intended to distinguish parvalbumin isoallergens, which share >67% but <90% amino acid (aa) identity. The last two digits refer to parvalbumin variants that share >90% aa identity [10]. Because fish have multiple different parvalbumins, this allergen nomenclature system has unavoidably resulted in instances where different (non-orthologous, encoded from different genes) parvalbumins from different fish species received the same numeric codes, as will be explained below.

**Figure 2:**
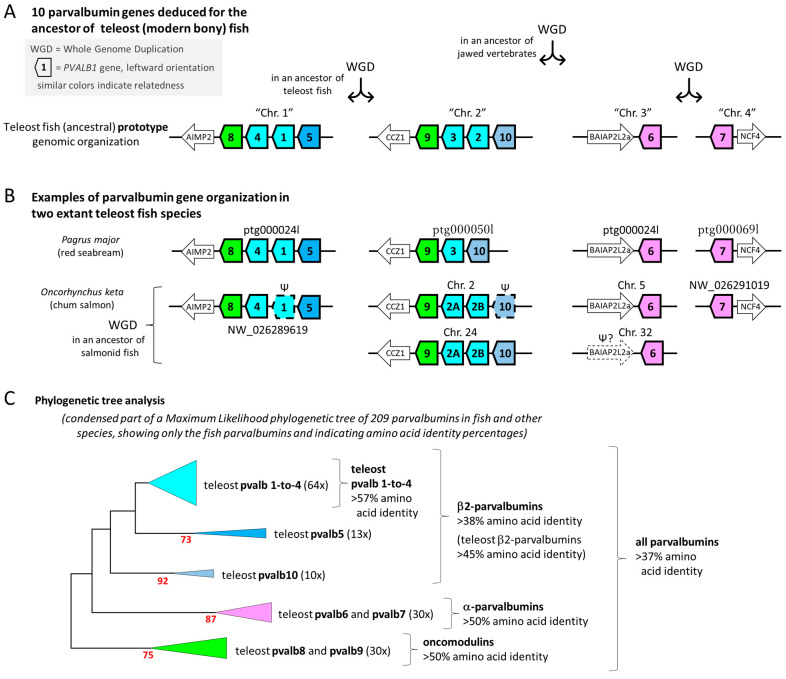
Parvalbumins in extant teleost fish derive from ten parvalbumin genes in their common ancestor and belong to three different ancient parvalbumin lineages. (**A**) The immediate ancestor of extant teleost fish possessed at least the genes *PVALB1*-to-*PVALB10*, spread over four different chromosomal regions deriving from two whole-genome duplication (WGD) events. The parvalbumin genes are indicated with thick-lined boxes that are pointed in the gene direction and are colored magenta for α-parvalbumins, green for oncomodulins, and different kinds of blue for *PVALB1*-to-4, e*PVALB5*, and *PVALB10*. Neighboring non-parvalbumin genes are indicated by lower boxes with their name abbreviations inside. (**B**) Parvalbumin gene organization in red seabream and chum salmon, with the direction of the depicted scaffolds adjusted for homogenization. For relevant genomic region information, or Genbank accession numbers providing access to such information, see Supplementary Files S1 and S2. Most symbols are as in (**A**), and the boxes with dashed lines and Ψ symbols indicate probable pseudogenes. (**C**) A condensed part of a phylogenetic tree created by the Maximum Likelihood method using 209 parvalbumin amino acid sequences of fishes and other species. Only the teleost fish sequences are indicated here, with between brackets the number of teleost sequences condensed in the respective part of the tree. For the complete tree and sequence information, see [9]. The percentage of trees in which the associated taxa clustered together is shown next to the branches if >50. Percentages of aa identity, calculated with the help of Clustal Omega (https://www.ebi.ac.uk/jdispatcher/msa/clustalo (accessed on 25 March 2024)), are indicated per cluster.

Parvalbumins are conserved in sequence, sharing >45% aa identity in most cases [11,12]. They can be divided into the three ancient evolutionary lineages “Alpha”, “Oncomodulin” (also known as “Beta-1”), and “Beta-2” (also known as “Other” or “Non-α/non-oncomodulin”), which can already be found at the evolutionary level of sharks (Figure 2) [9,12]. Commonly, in allergy research, the oncomodulins and β2-parvalbumins used to be classified together as “Beta” based on a higher acidity compared to α-parvalbumins. For a current overview of the allergy-relevant literature on parvalbumins, we refer to Dramburg et al., 2023 [13] and its respective book chapters on fish allergy and parvalbumin allergens.

In the present article, we will not use the Beta (or Beta-1) nomenclature, because Beta may not represent a true phylogenetic lineage and we consider the name to be easily misleading (unless only used as “non-Beta”). Identified fish parvalbumin allergens that previously were categorized as “Beta” all belong to the “Beta-2” group (a name introduced by Modrell et al., 2017 [12]). The α-parvalbumins, oncomodulins, and β2-parvalbumins all have a very similar protein structure (Figure 1B, which is an overview figure we also used in [9]), dedicated especially to the binding of Ca^2+^ while also being able to bind other cations such as Mg^2+^.

**Figure 3:**
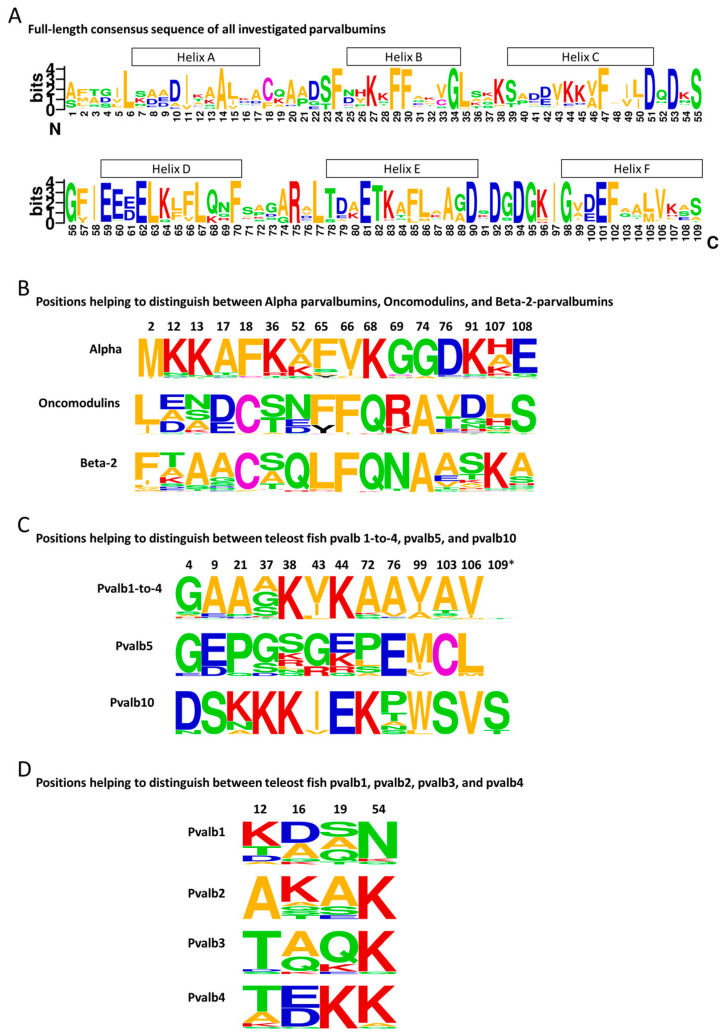
Parvalbumin amino acid consensus sequences. Consensus sequences were created using WebLogo 2.8.2 (https://weblogo.berkeley.edu/logo.cgi (accessed on 25 March 2024)) software for analysis of the parvalbumin sequences listed in [9], which tried to provide a broad overview of parvalbumin sequences while focusing on teleost parvalbumins. (**A**) Sequence logo for all analyzed 209 parvalbumin sequences, with helices indicated above the alignment based on the structure of common carp pvalb4_(Chr.A3) protein (PDB database 4CPV). (**B**) Frequency plots for residues at positions that help to distinguish between the α-parvalbumins (*n* = 45; 30 from teleosts), oncomodulins (*n* = 43; 30 from teleosts), and β2-parvalbumins (*n* = 121; 87 from teleosts). (**C**) Frequency plots for residues at positions that help to distinguish between the combined pvalb1-to-4 sequences (*n* = 64), pvalb5 (*n* = 13), and pvalb10 (*n* = 10) of teleosts. (**D**) Frequency plots for residues at positions that help to distinguish between teleost pvalb1 (*n* = 15), pvalb2 (*n* = 10), pvalb3 (*n* = 17), and pvalb4 (*n* = 22). The letters represent amino acids and their sizes correspond with their level of conservation. For a discussion of the structural importance of these characteristic residues, see [9]. *, many parvalbumins are a bit shorter and do not have a residue at position 109.

Because of the conserved structures and cation binding function, 42 of the approximately 109 residues are very well (though not perfectly) conserved between the various parvalbumins [9]. The positions and nature of these conserved residues are shown in Figure 1C and Figure 3A, respectively. The high level of conservation makes it difficult to determine the precise evolutionary relationships between many of the parvalbumins. The high level of conservation also appears to allow that, in species-specific evolution—despite the long evolutionary separation of these molecules—α-parvalbumins and β2-parvalbumins can seemingly take over each other’s function in Ca^2+^ buffering in muscle. Different fish species can use either one or both of these types of parvalbumin to regulate Ca^2+^ availability (for reviews on various parvalbumin functions, see [9,14]). We are not aware, however, of any species expressing oncomodulins as the main parvalbumin in muscle, so there may nonetheless be some parvalbumin type-specific restrictions in functional adaptation potential.

In the context of fish allergy, the following is important. Highly conserved parvalbumin linear or conformational motifs (epitopes) may serve as common recognition motifs for IgE antibodies, leading to IgE cross-reactivity to even distantly related parvalbumins in different species. For example, clinical cross-reactivity to chicken meat in Atlantic cod-allergic patients against fish can be explained by IgE-mediated effector cell reactivity to both β2-parvalbumins in cod and α-parvalbumin in chicken [15].

In single fish species, multiple different parvalbumin genes are found, ranging from 7 in Northern pike to 22 in Atlantic salmon [9,16]. This diversity makes analyses of the genetic evolution of parvalbumins challenging. How multiple parvalbumins are expressed in the same fish muscle is poorly understood. The approach of fish allergy research has been to focus on the most abundant parvalbumins because allergen expression levels are crucial for surpassing threshold dose reactivity and inducing clinical symptom onset [13,17].

The situation of multiple fish parvalbumins being very similar and therefore difficult to distinguish has been described as “microheterogeneity” and “high isoform complexity” [16,18,19]. In this review, we assign gene identities to known fish parvalbumin allergens using a gene nomenclature system established in our previous study [9]. We consider that such information on parvalbumin sequences is critical for a systematic comparison between fish species.

To additionally explain this new gene nomenclature system, we here identify all parvalbumin genes present in the genomes of two fish species that are common sources of fish allergy in Japan: red seabream (*Pagrus major*) and chum salmon (*Oncorhynchus keta*) [20].

We do not intend to propose an alternative nomenclature to the one maintained by the WHO/IUIS Allergen Nomenclature Sub-Committee. That official nomenclature has the strength of linking allergen names to clinical relevance. As a joint effort between authors from molecular sciences and allergy research, we hope that the present study will allow scientists to consider a complementary gene-based approach for the comprehensive identification of fish parvalbumins from a given fish species.

## 2. Three Ancient Parvalbumin Lineages and Ten Parvalbumin Genes in a Teleost Ancestor: Explanation of the *PVALB1*-to-*PVALB10* Gene Nomenclature System

Commercial fish meat is almost entirely from fish species belonging to the infraclass of Teleostei (teleosts, modern bony fish). Apart from an earlier whole-genome duplication (WGD) event shared with other jawed vertebrates, teleosts experienced a whole-genome duplication (WGD) at their evolutionary origin, creating four genomic regions with parvalbumin genes and a higher copy number of these genes than common in other species (Figure 2A) [9,16]. From the genomes of multiple teleost species, we could deduce that there were at least ten different parvalbumin genes in their last common ancestor, spread over the four ohnologous (meaning that they resulted from WGD events) chromosomal regions. We named these genes *PVALB1*-to-*PVALB10* [9], expanding on the *PVALB1*-to-*PVALB9* gene nomenclature that had already been established for zebrafish [21]. Of these ten “teleost prototype” parvalbumin genes, six belong to the Beta-2 lineage (*PVALB1*-to-*PVALB5* and *PVALB10*), two belong to the Oncomodulin lineage (*PVALB8* and PVALB9), and two belong to the Alpha lineage (*PVALB6* and *PVALB7*) (Figure 2A) [9]. In many groups of teleost fish species, this number of ten parvalbumins has changed, by acquiring new or losing old gene copies, and gene copy numbers between 7 and 22, in total, have been found [9,16] (examples in Figure 2B).

In cases where there has been an intra-chromosomal “tandem” duplication of a gene, our gene nomenclature distinguishes the resulting genes by adding letters (e.g., *PVALB2A* and *PVALB2B*). If new copies were created by a WGD event, the resulting genes are distinguished by adding the respective chromosome designation (or scaffold name if the chromosome is not known) to their name (e.g., *PVALB4*_(Chr.A3) and *PVALB4*_(Chr.B3)) [9]. Adjacent similar genes can be susceptible to intergenic recombination so that occasionally hybrid genes are generated [22]. We believe that this has been the case for “*PVALB3*” in ayu sweetfish (*Plecoglossus altivelis*) in which only the 5′ end looks like *PVALB3,* while the rest is very similar to ayu sweetfish *PVALB2* (Supplementary File S3); in such case, we name the gene based only on its unique part.

Computerized phylogenetic tree analysis, if applied to teleost fish parvalbumin protein sequences, can readily determine if they belong to either the pvalb1-to-pvalb4, pvalb5, or pvalb10 families (these all belong to the Beta-2 lineage), or to the Oncomodulin or Alpha lineages (Figure 2C). This is consistent with relatively large sequence consensus motifs that distinguish these parvalbumin groups (Figure 3B,C). However, computerized phylogenetic tree analysis cannot distinguish well between pvalb1, pvalb2, pvalb3, and pvalb4, between pvalb6 and pvalb7, or between pvalb8 and pvalb9, because within each of these groups, the different members hardly have distinguishing features and they probably have overlapping functions [9]. Fortunately, for distinguishing between *PVALB6* and *PVALB7*, or between *PVALB8* and *PVALB9*, the chromosome on which they are situated reveals their identity (Figure 2A,B) [9]. Chromosome identity also readily distinguishes between *PVALB1* + *PVALB4* versus *PVALB2* + *PVALB3*. However, especially in situations where the ancestral situation has changed because of gene duplications or deletions, sometimes it is not easy to distinguish *PVALB1* from *PVALB4* or *PVALB2* from *PVALB3*, and, in these instances, small motifs of only a few residues in their encoded proteins are helpful for distinguishing them (Figure 3D and Supplementary File S3).

In investigated species, *PVALB6*, *PVALB7*, *PVALB8*, and *PVALB9* are stably conserved, which should mean that each of them has a unique functional importance. Between fishes, the variation in parvalbumin gene copy number mostly concerns the β2-parvalbumins: *PVALB5* and *PVALB10* are only represented by one gene copy per genomic region or were lost in some fish, but within the *PVALB1*-to-*4* family, both gene duplications and deletions occurred, with *PVALB4* being the most stably conserved among them [9]. The pvalb4 molecules have a consensus motif of the interacting (D/E)16 and K19 residues (Figure 3D) (for structural analysis, see [9]) that may give them a slightly different function from the pvalb1-to-3 molecules.

In short, especially if genomic region information is available, identification of the genes *PVALB5*-to-*PVALB10* is quite straightforward, as is the distinction between *PVALB1*/*PVALB4* versus *PVALB2*/*PVALB3*. However, the distinction between *PVALB1* and *PVALB4*, and even more so between *PVALB2* and *PVALB3*, can be challenging. For the future naming of parvalbumin genes in novel fish species using the here discussed nomenclature system, it will be simplest to compare the new sequences with those of closely related fish species analyzed in our previous [9] or present study.

As a word of caution: For some but not all parvalbumin genes, automatic gene assignments in the NCBI database (https://www.ncbi.nlm.nih.gov/ (accessed on 25 March 2024)) intend to follow the zebrafish parvalbumin nomenclature by Friedberg 2005 [21], which is also the base of our genetic nomenclature system. However, readers should be aware that this genetic nomenclature as applied by NCBI (like any automatic approach to naming sets of very similar genes) is not complete and contains obvious errors. We suggest using these automatic NCBI analyses as a convenient starting point for parvalbumin gene identification but not as a final analysis.

## 3. Nine Parvalbumin Genes in Red Seabream (*Pagrus major*)

**Figure 4:**
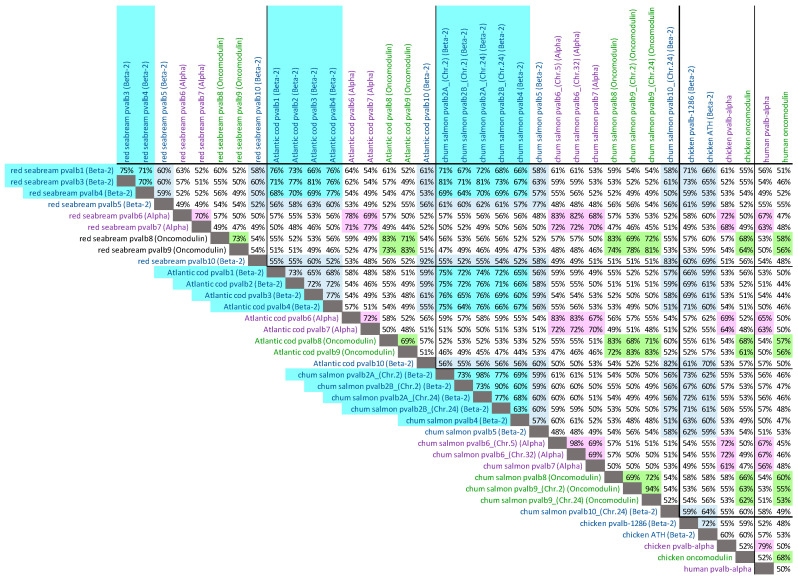
Percentages of amino acid identity between parvalbumins of red seabream, Atlantic cod, chum salmon, chicken, and human. Colors highlight comparisons between parvalbumins belonging to the same family: β2-parvalbumins (teleost pvalb1-to-4), blue (cyan); α-parvalbumins, pink; oncomodulins, green.

For red seabream, a first draft whole genome sequence was already published as GenBank assembly GCA_002897255 by the Center for Marine Environmental Studies, Ehime University, Japan. In that assembly, we could detect nine parvalbumin genes, but several of the scaffolds are short and lack information on one of the parvalbumin gene exons or neighboring genes. However, a higher-quality sequencing of the whole genome of another red seabream individual is currently ongoing as part of the project, “Accumulation of scientific knowledge for promoting public understanding of genome editing technologies”, funded by the Ministry of Agriculture, Forest and Fisheries, Japan, and performed by a collaboration of Kyoto University, Kindai University, National Agriculture and Food Research Organization (NARO), and Japan Fisheries Research and Education Agency. The leaders of this project kindly provided us with the sequences of long genomic stretches including the complete nine parvalbumin genes (Supplementary File S1). The genomic organization of these red seabream parvalbumin genes and some of their neighboring genes, which helped to identify the scaffolds, are shown in Figure 2B. Their deduced amino acid sequences are shown individually in Supplementary File S2 and aligned (by method, as in [23]), with the highlighting of characteristic motifs, in Supplementary File S3. The loss of *PVALB2* (Figure 2B) appears to be shared with the majority of neoteleost fishes [9]. That this gene is *PVALB3* and not *PVALB2* is supported by (i) the encoding of the residues T12 and Q19 (Figure 3D and Supplementary File S3), and (ii) the encoded molecule having a higher similarity with Atlantic cod pvalb3 than with Atlantic cod pvalb2 (Figure 4) and with zebrafish pvalb3 compared to zebrafish pvalb2 (85% versus 82% amino acid identity).

## 4. Thirteen Intact Parvalbumin Genes in Chum Salmon (*Oncorhynchus keta*)

Parvalbumin genes in chum salmon (*Oncorhynchus keta*) were identified as previously described [9]. Essentially, various datasets of the NCBI database (https://www.ncbi.nlm.nih.gov/ (accessed on 25 March 2024)) were searched for chum salmon parvalbumins by similarity searches, and genomic scaffolds with parvalbumin genes were analyzed by gene prediction software. The intact parvalbumin sequences are shown individually in Supplementary File S2 and aligned in Supplementary File S3. The sequences predicted from genomic DNA were confirmed by chum salmon transcript reads retrieved from the Single Reads Archive (SRA) database of GenBank (Supplementary File S2).

Early in the evolution of salmonids, an additional WGD event occurred [24], originally leading to an unusually high number of parvalbumin genes [16]. However, according to our analysis of known chum salmon sequences but also related species, one of the original duplicated genomic regions that included *PVALB8*, -*4*, -*1*, and -*5* appears to have been lost in the genus *Oncorhynchus*. Furthermore, in chum salmon, at the expected locations of *PVALB1* in scaffold NW_026289619 and of *PVALB10* in Chr. 2, only pseudogene fragments of the respective genes were found (Figure 2B). The loss of *PVALB3* and a tandem duplication of *PVALB2* (into *PVALB2A* and *PVALB2B*) (Figure 2B) appears to have happened early in the fish clade Protacanthopterygii, as it was also described for Northern pike (*Esox lucius*) [9]. In the alignment figure, it can readily be seen that the chum salmon pvalb2A and pvalb2B sequences match their name counterparts in Northern pike (Supplementary File S3). These sequences are classified as *PVALB2* and not *PVALB3* because (i) they encode the residue A12 and a relatively small residue at position 19 (Figure 3D and Supplementary File S3), (ii) the encoded molecules have, on average, a higher similarity with Atlantic cod pvalb2 than with Atlantic cod pvalb3 (Figure 4) and with zebrafish pvalb2 than zebrafish pvalb3, and (iii) phylogenetic trees based on nucleotide sequences cluster the pike and salmonid *PVALB2A* and *PVALB2B* sequences, together with *PVALB2* of other teleosts, apart from *PVALB3* [16].

## 5. Fish Parvalbumin Allergens Catalogued in the Allergen Nomenclature Database of the World Health Organization (WHO) and the International Union of Immunological Societies (IUIS)

We searched the Allergen Nomenclature database (http://allergen.org/ (accessed on 15 June 2024)) maintained by the WHO/IUIS Allergen Nomenclature Sub-Committee using “parvalbumin” as a “biochemical name” search word and retrieved all the sequences with few exceptions: (i) the entry under species name *Gadus callarius* is not discussed here, as this name is just an alternative for (or a subspecies of) *Gadus morhua* (for which reliable information is available, see Table 1), and the 1975 deposited parvalbumin sequence Gad c 1.0101 (GenBank accession P02622, [6]) is so different from all known parvalbumins, including all gene-encoded sequences deduced for the genus *Gadus*, that we assume it to include considerable sequencing errors; (ii) the entry rainbow trout Onc m 1 is not included here because a polished full-length sequence was not determined (see GenBank accessions P86431 and P86432 in which multiple unknown residues are depicted as “X”); and (iii) while preparing our manuscript, the allergen Tric l 1.0101 was added to the database for *Trichiurus lepturus* (Atlantic cutlassfish), but the sequence information seems not to be available at this moment.

The retrieved fish parvalbumin allergens catalogued in the Allergen Nomenclature database are listed in Table 1, together with their gene-based identities that we determined following the method described above. The sequences are shown individually in Supplementary File S2, together with arguments for their gene identification, and aligned with other fish parvalbumin sequences in Supplementary File S3.

The fish parvalbumins that so far have mostly been associated with food-allergic reactions are pvalb1-to-4 (Table 1), which belong to the Beta-2 family and are expressed highly in muscle tissue (meat) [9]. At the protein level, variable allergen levels have been reported for β2-parvalbumins of commonly consumed fish species [25]. However, in some bony fish, parvalbumins of Alpha lineage have been described as the most abundant parvalbumin in muscle (pvalb7 in Northern pike [26]) or were shown to be bound by IgE antibodies of allergic patients (pvalb7 in striped catfish [27]).

Because the official parvalbumin allergens are numbered based on the order of identification per species, such allergen designations are different from gene-based names (Table 1). Indeed, the striped catfish pvalb7 allergen “Pan h 1.0201” is an α-parvalbumin that shares <67% aa identity with the other listed parvalbumin allergens, which are β2-parvalbumins (e.g., cod Gad m 1.0201). However, since allergenic cross-reactivity against α-parvalbumins and β2-parvalbumins appears to exist [15], it does make clinical sense to classify them both into the fish allergen “1” (parvalbumin) group. As we discussed in the introduction of this review, the principles of the WHO/IUIS allergen naming system are based on clinical significance [10], and we do not suggest changing it. However, we here propose a complementary approach of applying parvalbumin gene identities to the designation of the molecules. This may also benefit the understanding of molecular relationships and cross-reactivities of the parvalbumin isoallergens and variants in the official WHO/IUIS allergen nomenclature system.

The last two digits of the allergen names as proposed by the WHO/IUIS Allergen Nomenclature system are dedicated to allergen variants that share >90% aa identity [10]. As a comment, the differences between Atlantic cod Gad m 1.0101 versus Gad m 1.0102, and Gad m 1.0201 versus Gad m 1.0202 (Table 1) are so small (only 1 aa different) that they appear to derive from either allelic (same gene, different allele) variation or sequencing errors (Supplementary File S2).

## 6. Parvalbumin Allergenicity and Molecular IgE-Binding Sites

The importance of fish parvalbumins as food allergens has been attributed to their high abundance in fish muscle and structural stability [1,9,28,29]. Parvalbumin allergenic epitopes, meaning specific IgE-binding sites on the molecular structure, can be linear or conformational [19,30,31]. For cod parvalbumin, epitope studies have shown that multiple regions of the protein engage in allergic antigen–antibody interactions [30,32]. A more complex IgE response, characterized by a more diverse IgE epitope repertoire, is associated with more severe clinical symptoms of fish allergy. The high level of conservation between all parvalbumins creates a realistic chance that evolutionary sequence “fluctuations” may generate identical allergenic epitopes in very different fish species. Even different parvalbumin types, alpha and beta, might become cross-reactive, such as that observed for bony fish parvalbumins and parvalbumins from cartilaginous fish, birds, and others [15,33,34].

So far, depending on the fish species, protein isolation and gene expression studies have indicated that either the pvalb1-to-4 family molecules or the α-parvalbumins are the most abundant parvalbumins in muscle [8,9,35]. This is in agreement with only molecules of these two parvalbumin families being among the identified allergens (Table 1).

Fish allergy studies focus on allergens that are abundant in clinically relevant amounts. This is why the allergenicity of less abundant parvalbumins is not well understood. However, at least from a scientific point of view, it might be interesting to select a few model fish species where the allergenicity of all parvalbumins—all expressed parvalbumin isoallergens and variants—is comprehensively studied. In Japan, red seabream and chum salmon are important consumption fish and major causes of fish allergies [20]. In the present study, we determined their parvalbumin sequences so that in the future we may determine the allergenicity of each of their individual parvalbumin molecules.

## 7. Conclusions

Parvalbumins are the major allergens in fish meat. Teleost fish species express multiple very similar parvalbumin molecules, among which the precise identities and evolutionary relationships cannot be assessed in a simple manner. For the advanced understanding of parvalbumin allergens recognized by the WHO/IUIS Allergen Nomenclature system, we here propose to additionally consider a gene-based (orthology-based) identification system. Knowing the identity of a parvalbumin in a genetic classification should make it easier to compare parvalbumin situations between species in future food allergy research.

## Figures and Tables

**Table 1:** Nomenclature of fish parvalbumin allergens.

Species	Allergen Name ^1^	Gene-Based Name ^2^
*Clupea harengus* (Atlantic herring)	Clu h 1.0101	pvalb3
	Clu h 1.0201	pvalb1
	Clu h 1.0301	pvalb4
*Ctenopharyngodon idella* (Grass carp)	Cten i 1.0101	pvalb4
*Cyprinus carpio* (Common carp)	Cyp c 1.0101	pvalb3_(Chr.B12)
	Cyp c 1.0201	pvalb4_(Chr.B3)
*Gadus morhua* (Atlantic cod)	Gad m 1.0101	pvalb2.01
	Gad m 1.0102	pvalb2.02
	Gad m 1.0201	pvalb3.01
	Gad m 1.0202	pvalb3.02
*Lates calcarifer* (Barramundi)	Lat c 1.0101	pvalb3
	Lat c 1.0201	pvalb4
*Lepidorhombus whiffiagonis* (Whiff)	Lep w 1.0101	pvalb1
*Pangasianodon hypophthalmus* (Striped catfish)	Pan h 1.0101	pvalb4
	Pan h 1.0201	pvalb7
*Rastrelliger kanagurta* (Indian mackerel)	Ras k 1.0101	pvalb4
*Salmo salar* (Atlantic salmon)	Sal s 1.0101	pvalb4_(Chr.3)
*Sardinops sagax* (Pacific pilchard)	Sar sa 1.0101	pvalb4
*Scomber scombrus* (Atlantic mackerel)	Sco s 1.0101	pvalb4
*Sebastes marinus* (Ocean perch, redfish)	Seb m 1.0101	pvalb3
	Seb m 1.0201	pvalb4
*Thunnus albacares* (Yellowfin tuna)	Thu a 1.0101	pvalb3
*Xiphias gladius* (Swordfish)	Xip g 1.0101	pvalb4

^1^ Names in the Allergen Nomenclature database by WHO/IUIS (http://allergen.org/ (accessed on 15 June 2024)). ^2^ These gene-based names follow the nomenclature system first introduced in [9], building on [21].

## Data Availability

Sequence data and information on their sources are extensively provided in Supplementary Files S1–S3.

## Supplementary Materials

The following supporting information can be downloaded at: https://www.mdpi.com/article/10.3390/genes15101337/s1, Supplementary File S1: red seabream (*Pagrus major*) genomic scaffold sequences with parvalbumin genes; Supplementary File S2: teleost fish parvalbumin sequences; Supplementary File S3: sequence alignment. References [36,37,38,39,40,41,42,43,44,45,46,47] are cited in Supplementary File.
