# Supplementary material for: Exploring Fish Parvalbumins through Allergen Names and Gene Identities
Source: Genes (Basel). 2024 Oct 18;15(10):1337. doi: 10.3390/genes15101337 (PMC11507022; doi:10.3390/genes15101337)
Supplement: Supplementary file 1 [file genes-15-01337-s001.zip › Supplementary File S1.pdf]

Supplementary File S1

Red seabream (*Pagrus major*) Genomic Scaffold Sequences with Parvalbumin Genes

This file contains four genomic scaffold sequences from red seabream (Pagrus major) that were determined as part of the project "Accumulation of scientific knowledge for promoting public understanding of genome editing technologies," funded by the Ministry of Agriculture, Forest and Fisheries, Japan, and performed by a collaboration of Kyoto University, Kindai University, National Agriculture and food Research Organization (NARO), and Japan Fisheries Research and Education Agency. The scaffolds were selected because, together, they contain all the red seabream parvalbumin genes. Scaffold 1 (page 1) encodes pvalb1, pvalb4, pvalb5, and pvalb8. Scaffold 2 (page 31) encodes pvalb3, pvalb9, and pvalb10. Scaffold 3 (page 59) encodes pvalb6. Scaffold 4 (page 84) encodes pvalb7. Scaffolds 1, 2, 3, and 4 represent parts of the chromosomes that in the main text Fig. 2 for the "prototype" organization in the teleost ancestor have schematically been named Chr. 1, Chr. 2, Chr. 3, and Chr. 4.

SCAFFOLD 1 (129,997 nt); encodes pvalb1, pvalb4, pvalb5, and pvalb8

```
TCTTACCTGCACTTGAGAAACAGATGAATAAAAAATGGTAAAAGAAAATACTGGAATGTATTTATACCGTTGATGTTGCTT
GTTAATCGTCCAAATGACTGTTAGAAATCTTTTCTGTTGCCATAAGACAGTAAAAGAATGTACTGAAAAGGACCTCTGCAT
TTCACCGTCGGGTCCTTATGAAAACGTTGTATCTCAGCGTCGATACTGAAATGATGTTTGAGGGGAGGTGCATCCAAAAGG
GCACACAAGTGATCTTTACCCTTAAGTCCCTCTTGTATATACATTTGCATAACAGATGCTTTAAAAATATTGTCCTTTGCC
ATCTAACTTAACTTTTTGTAGAGCTAGACCATCATGGAAACAGGACTTTTGCCTTAATGACACATACGTAATGTAAAAATA
TCATGTATCTGTTGCATTCTTCAGATTGTGAACATGAGTTGTGACAATCTCCTCCATGCTGACAGTTTAATCATTTTGGTC
TTTGGCCCACATTACATCCCTAATAAAATTTGCTGTATGAAAAATAAAGATTTATTTCTTCAACTCGCTGTTATAGTTTGG
TTTCATGCACAAAGAAAGTGTTTGAATGTTCCTATGGTCTGAAAAGTAACATTTCAGTTTATTTACAGGAAGCGATGTTCA
TGCATTTGCAAAACATAAAACAAAAAAATGTCTCAGCAGCTAAGTAGCATTCATTTTTAATTACAGTAACTCATATATAAA
AAGTAAGAACCTCAATCATAAGAGCGTGCGTAGTGTGTTTTCTAAAGCAAGATTTTTTTTTGAAATCCCTCCCACCCAGT
TTATATTTTTCTCTTCTGCCATAGACAGTTATATATCATCTTGCTGCGATGGAAGACGTAGACATGGAGATACTGCACATA
CAAGGATATAGACATTCTCGATTGATTGTCTGGTTGGGAGGAATGGGGCAGGGTGACTTGTCACAGAGTACTAAGATACAA
ATCCAGAGATTAAACACATTATTGAGAGATTTCTTCAGCTTTTACTCTCAGCATGCTTCCTTGAAGTGGAACTCCTCGCTT
TGACATAAAGAGTCCTCTCAGACTAGGAAGAAGTCCGAAGTCAAACAGTGATTGAGGTTGTAAAGTCGAGGGCATTTCAGA
TTGTATGTACCTGAGGTCTGCATTGGCCCTGGAAAAGCACAACCCAGCAGAATAATCATCAAACAAACGGTCAGTGCAAAT
ATGGCCCCAAGAAATAATATCAACAAAAACAGACAGAATGGTGCAGGCTTTCTGTCTGATGGAAGTCCATTGTTCCAGCCG
GGACAGTTCACGTCCAAGGATCTTTACACGTCTTTTTTTTCTCTTAAAAATGTTGTATGTGTTACTCCATGTGACACGAAG
ACGCAAAAAAAAAGGTTCTGGAAAAAAAAAGCTAATACATCAGTGATCTACATGGATCATGTGAGTACAATGTTACTAAGCAC
AAAACAAAAAAAATGTAAGCAGCGTGCCAGCAAAGACAAAAAAAAAAAGGAAAACAAAAAATCAGGCACAAAAACTACACGTG
CCTCAGTTAACTCTGGCTTTTCAAAACATAAAAGGAATCTGCTCAGATCCGTGGATAACCGAGCTAAATCTACAGAAGAAA
TCAATGACTGATGGAATCTCTGATGATCACTCAGCATGAAGTGAAGGCACTGACAACAGCTGGACACTTACAAATGCTAGA
ATATATCCTCAACTGGCAGATGAAAACACCGTTCAACACGTGCCTCTAATAAAAACTCACGTCGTAATTCATGGTAATATC
AAGTTGATCATTTGTTGTACATAACATAATGGCAACAGCAATATGTGCAATATTAAGAAAAGATAACAAAATAAAAGACAC
AAAAATGCTGGATTATGTGTGTGTTTGTGTGTATGTGTGTGTTGTCTATGCATGGGAGCATGAGCAGGTGCATGAGCTGTG
GTTTGGTCCCTCTAGCCGAAGGTGGCGCCACAGTTGGGACACCTTCTCTGGCGCAGTGCGAAGCAGAAGAGGAGCCCAAGG
```

GGGAAGAAGAAGATGGCACACAAGATCCCCAGGCAAGTGAAGTCATCCTCCAGGACACCAACTCTGCAACACACACAACAG
AAATACGATACAGGATTTAAATCACAAAAAGACATTTGTCTGGATGGATTACTGAAAGAGAAGAGACACTAAATGACCACA
AAGAGGCCTGAAACATTGAAAGAGACATAAATAATACAGAAAGATGCAAAACAACTGACACAAAACTAACAACAAAGATAC
AAAAAGAAGCAAAACAACTACAAAGAGACATTAGACAACTACAAGGACATGCAAAATTACCGCAGAGAGACACTGATCGGC
CACAAAGGCCCAAAACATCCACAAAGTGATGTCAATGACCATAAAGAGACACAAAAACAACAACAAAGAGACACGAAACAA
ACTATCTAGTTTAACTAACTGTTAATTATCGACAGTTTCTTATGGCTGAGATTCAAGGCTTCAAACTTAAGTCTGAACACA
TTTTACTATTGGGCCCCACTTCTTTAAAACTTAAGAAACTCACAGGTCACGTCTGCTAAAGGCTTCTTCAGAAGTGACCGA
GGTCTGACCTGACTAATAACACTTAACCGAATGCTGGAGTTCCTACCTTCAATCTAAGCTATGCCCTGACATTTAAACAAC
AATCAGGAAGGAACTGCTCAGTGCCGTTTCCTGTTTTCCATTTTTTTCTTCTTCCCCCATAGAGCTCGATCACTGCCTCAA
TGTAGAGACCGGAGAAAGAATGAGAATCATTCAACACCTTAATCATTGTGTTTTTTTCAAGTTAGAAGGAACAAAAGACTG
TTTGACTATTTATAAAAACATCTATTTATAAAACATCTAACCTTTGACACATCTTTAATTTATCTTTGTGACAATATTTAA
AAAGGTTGCTAATGTCTTGTTGAATTGCTAAAAGTTGAAGAAAGTACAATCCTGAGGTCTTGAAGGCCGCGTCTCGCAGCA
GCTTTAATCCAGAGTCACGGTGAACAAGTGCTAACCGCGGTCTCATGACTGCACCACGACGACCAACGTGGCCACTAAATC
TGCTGGAGGCCTCGAGGAGGAATTAACAAATGACGTGTAACAACCCAGATCCTGAAGAGATGGGAAATGAGAAGCTTCACT
GGAAAGCATTTACTGCTAATGCTATTAGTGTTGCATAGCTGTAGATTTTTTGATTAAATGGAAATAATCTTCATAAATAGG
CCTTAATTACGCTGATAACATTAGATTCTGCTGCGGTGATGAACTGGGTTTAAACCCTATTAAGATTTCTCTAGCTCCAAA
AACATTATACACAAATACATGGTATTGCAAGTTAACAGTGTTTGGTCTGCCGTGGCACCACACCACAGACTATGTGCCATG
TCACAAGTAGTTGTATTTGCACAGCTTATCTGTGCACCGCTGCTAGGAGCTGTGGCTTTTATTGACCCAGGAAACGTGGCC
ATGATGCAAATCCTACTCTCTTACACACATTTCACACACTGTAGACACAACAGTGTGCGTTAGTGTCAGGGAGTAGCATGT
CAGGGGTACTGAACTGGGGACTACATAAAAAACAACTCAATGAAAACATAAAATGGCTCTATTCAGTTTGTCCAGCATAAT
AAAAAGTCTGTGGAAGCCCCGAGATCCCAAATTTATTTGAAAAGACTATGTTTACACCGTGGACGCCTGTGATCCTGAGCA
TGACCTCAGTGTAACTCTACCTTCAGGGTGAGCAGAGAAGATGTCAATCAACAAACAAACTATGAGCTCATTGGACTTGAG
AAAGGAAATTCCTTTAGAAGTTGTTTAGGAGAATACTGCAACACTATCCTCCTGTGAAGTTCCAGAAAAGTTTTGCGGACT
TAAAAAACTTCACCCAACTTTCCACTGGCATGAGGGTGAGTAGTTAATGACTGGATTTTTCGCCGATCTTATCCTTTAAGA
CCATTTTATGCCACTGACATAGTGATAATATAAGAGCACTGTAATACAAGTACAGCCTTTCAAAGACCAGTGAACTTTTAA
TTCTCAAGAATATTTAGACATTGAAATTGGAATGTGAATCAATATTAAATTATCCTAAATAAATGAAACACAAATAAAAAT
GGACTCTGTGGCTGTGTTGAACACAACAGGCAACACAGCCACATCCAGAAATATGGATGCAAATGATCAACATCATGTACG
TATGACTGAGTTGATAAGATGAGTGTGATAACTCCAAACAAAAACACACAGAGTACATTCATTTAACAGCCGTGTCATTGT
GCAGTCAGAAAACCCCCAGGGCCCCGCTGCAGGCTTAGTGGAGTCAGTCCAGGTGTTCCTCTTTTTGGGCAACTTTATAAT
TTCGTAATACAGTAAGTTTCAGAGACTGCCTGTAATGATGATTATTTAGCAAGAACATAAATAACCACATATGTAACCATG
TTCCTATAACTGTTCTGCATCTTATTTTACATGAATCATGGCATTGTATCCCTGTTTTTATATTCATTCATGTCTTTTGTA
TGTCAAGCACATGTCATTTCTTTGTTATAAGGCACTTTGAGTTGTATGAAAAGTGCAATACAATTAGAGTTTGTTATCATC
CTTTTTTTTTTATTAAGATAGCTTTCAGTTGAAATAAAACCAACAGAAGCACACTTGCAGGATATTCATCATTCACCTTGT
GACAATAAACAATCCTGCTGAACTGCTGTGTGTTAAAAAGCTACACAACCACATTCCTTCAGTTCCTGGAATAGACATACA
TTACATTTCATTATTTAAATTCAACCAGCGGATGGGAGCACGTGTGATTCCCATTTGCTGTTCTTGTGAGTCGTGACCTGA
CCGGCCCCCCCCCCGTCACATGTTCCAGGCTGAAAATCATCTGACTGACAGGGTGTTGACTCAAGCACAGTGAAACCTACA
TAAAACTGTCGTCTAAAAACTGAAAGTCATGTCCGCTTGACATCGGGTCACACTGTAGGCCTGATAATAGGCACGCAGGAA
TGACAAAACCACACAGACATGATGGTTTTGGCCTCCAAAGGAACTGTGGTCAGGAACCAGCTTAGTCACAGTTTAACCTCG
TGCTGATCGAGAAAGTGTGTTATTTTTGTTCATGCCTACAGTATCTCCTTCTTTCCTCTTTGTGTATGAACTGTGAATCAA
GTGTTCTGATGTATGTGGGACACTTAGACATCATATGATCATATTAGCCCTCCGTTGTCTAAATGATATACACTCCAATTT
GTATATTTTTGACCCAATACACTGTTTTTACTCTTGCATTTGTTTTGCACTTTTACTTCAGTATACATTTTCAGTACTCTA
CCCACCTCTACAAAGATGTTAAGATGATGAGTGGGTGTGCGTGTGCGTGTGCTCTCACCTGCAGGCAGGGCAGCCCCCGAC
TACCACTACAGAGGGTTGGATGATGGTGTAGGTCCCAGCATAGGGCTGCTGGGCTACTGCAGGGGCCATTTGGGCTGATGG
GTACCCTGTGGTTGACAGAAGGAGACGATATACATGTTATATATACACTGTATATACATACATGTTATGGTTTGCAGGAGA
TCTGTTTACATGGGACCCTGCACACACCCAGCCCTGTGACACAGTTAGAAAGCACTTAAGTAATAAAACACAAGTTTATAA
CTTCTTTAGCACCAGGACAATAAAAGGAAGTGACACTGCAGGTTATACAAGGTGGTATACGAGCTTTAGTCCAGTCTGAAG
TTGTATGACATTGATCTATTGGTAGGAAATTAGGTTGAACAATCAAATGTCATGTCAGAGAGCTACAATCTTTGTGAGAAT
GTACCCTCTGTGTATACTCAGGATATGGCGATTTATGGGGGCAATGATACTGATTTATAATCACAGGGAAGGTTCAAAGAT
CAGTCAAATCTTTCCATGACAAAACACAGCTCGGACCTCTTATAAGACTTGTCTTTAATTCAAGTTTCTGCAGTGAAAAGC
TGGCTGGTGAAAGTCAAAGACAGGTTACGCCAAATCAGAGCTGATCAGTGTGTATATTGTTTGTTGGAGGGCTCACTGGTT
CGTTTCACTCTCAGGACATATTGATTTCTTTTCCAACCACCTGTGTATGTGGGCGGTGATCATTTGTGTTGACACACTGAA
CTTTGATACCGAACTTCTCAGAAATGTTTACCAACACCTTTTGTCTGATAAGGATGCCCCCCTGTAAAGACAGACTAAAAC
TCAACACAGTACAGCTGTACATACAGTAAACATAACAATTGTCCCTTTGAGTTGCAGCAACAGACTGTGTTCATCTTGGGA
GAAAACCAGAAGCACGTGATGAGCAAATAGGAAATTACATGCAACTGCAAGAAATGACACTTCATGTCAACTGCATAACTT
CCTCGTTGTAATTAGAGCTGTAAAGGTTAGCTAAGTAATCTATTAACTGGTAACTATTAAATCATATGATATATAGTCCTA

TTTTGATAATCGGTTAATCATTTATAGACTTTTTTGAAAGAAAGATTCTTTGATTTTGGCTTCTGAAATGTGATTTTATTT
TCTTCTGATTTCTTTACTCCTCTGTAACAGTAAACTGAATATCTTTGGGTTTTGGAGAGATCAAGACATTTAAGGATGTCA
TCTTGGGCTGTGGGGGGGGGGGGGAATAATCACAATTTTTTACCCTTTCCTGACATTTTATGGAACAAGCCACTAATCGCT
TAATCGAGAAAATGAACCAATTCAATCACAGTCAGATGATGCCTGCCTCCTTCACATATAACACACACATACTGTGTGAGT
ACATGTACATGGTGGTGCAGAGCTCCCTGCAGTCTGAGGAAACTTCTACATATTTCATATTCTGTTGCTTCACATAAAAAC
ACTTATTTGATTGATTGTCTGTAAACATCAAACTGTAAACTGTGCCTTTTTGTAATTTCTGTGAGCACTCATGGTTGACTG
CTTTAAAAAAAACAACAGCGTGTTACCTAATTTATGCAAAAAACTGGTCAGTATAAATAACACATCGCTATTGCCCTACTG
CGCATGCGCCACATCGGAGCACCTGATAAGCGAAACTAATGGTTCTTTGACGGGTTTTGTAACTTTAAGTCTCAGACATGG
TGGATTAAAATCGACCCTGAGAGTTAATGACGGCAACAAAGCCGAAACAAATCGACATTTGATGTTTGAACGAAGACGGAA
GTGATCGGGGGTGAGCCCTCTCTTCCTGTCTGGGTACTTTCAGCTCACCGTGCGGACCGTGTGACATGAGCGGGGCTACTT
GCCTTGGCCGTCGGGGTACGGGTATGGCGGCGGGGCCGGCGGGGGGATGGCTCCATAGCTGTTCTGCTGCGGGCCGTACTC
ATAAGCCCCCGGGACGGCGTTGTAGGCGGGAGGTCTGTCCTGAAGCAGAGGTTTGCTGTCCACCATCTCAGCCGACGTAAT
GCGTCCTCAACTTGTCTGCTTTAACTTAATAAACTCAGCAGCAGAGAGGCTCTGAACTGAACTCCGGCTACTCTTCTTCTG
TGGCACGAAAAGCCGTGCGACGTTCACTGACGCGCAGTGGCGACAAAAACAATGAAAACAAAGAAAAGACGCTGCGCCAAA
ATGAAACTTCGTCCCTCTCCATGCGCCAACTTTGTCCCCGTCGGCGCAACAACCCCAAATCTTTCCCGAGCGTCCGCTTCA
AAGACAGGAATGAGGCTGATTCAGTCTGGTAATGGTTAACTTAACTCCATTTCCCTCCGTTCACTTGGTCCTGTGGCACCA
AAACAACAGTCATCTGTCATCGCAGCACCTTGTAACTCAGCTGGAAGTCAGAAAGGCTTTTCCTTTCTGTCTGGATTGTCA
CAAGAGAAGAATCATGTGATGAGCTGCTGTAGCGCCTCTGCGCACTCAAAGACTTCTTAAAGCGACAGCGCACGAAAATAA
AAGGCTGCAGGCTGAGCACTGCAGAGCCAGCTGCTCCACCTCACAGCACACCATCTACTGTAGTGATAGGCCTGTGTACAT
AGTGCAGTCACTGCATGTCCATGCCGCTTTTCAGGTGGTGCACATGTGAGATTATCATGTTACTTGTGGGACCAAGTACGA
TTTATTACTCATGGGGCCTCTCATTAAATTGCTTACACTTACAGTAACTTACATAATTGCTGTCCTGATTATGATTGGGCC
ATCCTTGATGTTGCATAATATTGATCTCACGTCCTCGGATTTAATCAGAGTAGAAGGTTGCAGCAGGTTTTTGGGAGGATA
AACAGGATTTTCCACAAAAAACCCCCCAAAAAATCAATATTAAACACTTTAAAGGGGCAACTTACTTCAATATTTTAGCTT
CTCTGTTAGGCTCAGGCCATAATTTGATGAGAAAACATAAATAAAACTGTAAATCTGTGACGCTTTGAAGCTCTTAAAAAA
TATTACAACCTAACGTATTATTTATTTAAAGGTGCGACATGTCAGGAGTAGCCACCTGTTGAATTTATACTCAAAACAGAT
GACCGTTAACTGCTACTAACTTTAGCTGCCATTAGCTCAGTTAGCCATGCAGCTAGCAGTTCAGACTGGAAGCCCGGGAAG
TGTTGGTGTTTACATTACTCATACAAGAACATTGGGGCTAGATGATTAGCATGCTAGCACCAGTAAATATCTGCAACACAA
TACGCAGATGTTATGTCGTCACTTCCTGCTGAAAATGTATGTACATTTTTTAACGATTTCAAATAAGATTCTGACATGTTG
CACCTTTAAGAGGGGTTTTGTCTTGAAGAGTGAGACTTTATGTATAGTATCTGATGTACCAGATGTCTATGAATATAAAAT
ATACAAGTAACCTGTTACATTAGTCATTTATTTAGCTGAGTATTTATTACAGCAATCTTTGCTCCCAGCACCATTGCACTA
TTGTATTCTTGTTTACAATTTACAGAAACAGTGTGACTTCTACAGTATATAGACATGTATGTTGTTGTTAGTTGTTGCTTT
ATATATACACACTGTTTCACCTACTTGTATATACATGCTGTTATCATTAAATATTTTCCTTTGTTCAGCTTCGTTGTCCTG
GTTCTTAGCTCTATTTCTATTTCGAGTGAAATTCCTTGTATGTGGACAACCAAAGAGATTTAGGCGATATATTCTGAATGC
ATCGATTAAAGGTGCACTCTGTAGTTCTGGGGAATACATTTTAATTAGACGAGAAAGATCTTTTATGCCTAAATAAACAAA
CGCTCTTTGTTTTCAGGACTGAATAAACAAACTGACCTTAAAGGACAATTTCATACTGTTTTACTTTGTTTATATGTGGCG
GACCCTGCCACCTTTCTAGCTTCAAACAGTGTTCTGGGACCTTATTTTCCTCTGAGAACAGCTTGTTTATTCATTTATGGG
AAAAAAAACAATATTTCAGAGTTTGTATTATTACCTCATATTGTATATATTACAATTCTAAGCTTGAATTTCTTGTCCAAA
ACTACATAGCGCCCCTTTTATTGGCTACTACTTGTACTCACATACTCAGCTATGTTATACAATTAGATACTACTTGTATAA
CATAGCTGTTGCTGCTACTTATACCAACTCCTGCCTCCTGATTAACACTTATACAAGATGAGGTCACCGATGTGCTGCAAT
AAAAACCTGCAGCTCAGGTGGATCATGTCTAACTTCCTGTTTTACAGCCACCTCTGTTCAATTTGCCGAACCCTGGACAAG
ACAGAGGTTCATCCTGAGAGGCAGCATAATATCATTTCCCCACCCAACATAAACGTTACTGAGGGAAGTTGGCCATGAATA
AAAGTTTTTTCCGCACCACTCCACTGTTTCTATCCTCCCAGCTTCTCTCTGCATTAAATATACTGTATCCAAACACGTAAA
ACCAAACCTTACGCTTCAATAATTCATTTGGCGCTGAATGAATTATTGAAATATGCGCGTCAATCAGCCGAGGCGTCGGAA
AGAAAGAAAAAAAAAGAACGCGCACCAGAGGGGCTCCTGTGTTCGACCAATGGGAGAGCGAGGTCCCAGAGCTCCAGCCAA
TGGGAGCGCCACTTCATCCATTTCCTGTTGTGGAGTCGGGAGCTGCACAGAGCATTCCTCTGCTTCCACTATGTGAATTAA
AACACCACGAATGAGTCCACCTCTCCACAGCGGAGGTGTCGTTCCTGACACAGCCTCATGTGTAAGTAACAGCCATGTGTG
TGTGAGAGTGTGTGTGGCCGCTGCCTGCAGCCCGGACCCGTAAACAGATTATAGCCGACTTTGCGGCATCCATATCAGCTG
TCTGTGTTATTACGATGCAGAGCGTGTTTCTCACTCCTCCTCCTCCTCCTGCTGTCATCTAACGTGGATCCAAACATCTTG
AATGTGTCCTAGCGTGGGCCTCGGAGACGGCAGGTATCGGCTGTTGCACCTGATGTCATGACACGTCTGTTGATATTCCCT
TGACAACCGCGGATCGGATAGCAATATTCAGGTAGGCTGCTGTTGTTTTTCATCCTGGATTTACTGCAGGCCTCTGAGTGT
GTGTGTGTGTGTGTGTGTGTGGAGAGCTCCACTGCAACATATAGGAGACATGAAGTTTTAGTCTGTGAAGCTGCATTCTGT
TACAAACTATGTAATGCGTTGGGCTTTATTTGCTCCAGGTGATTTCTGTGTCTTTAAACGCATCATTTCCGTCCATTTGAT
TTTGGAGAATAAAGTTCAAGCGGCTGAATGTGTCACGTTACCGTACATTTGTTTAAAGAAGACAGGGTGAAGGCCTGTGTG
TGTGGCACAGTGACAGTCAATATCGTAGCGGATTAGTATCAGTCGATAAAGCAAGTGTCAAGCTACGTGGTGAGAAGAAAA
GCGAGACTTCCGGTCAGAAATTTCAAAGTAAAACATCATGGCCACGCTGTAATGGTCACTTGAGGAAACAAAAAATCCAAG

AAGGGAATGTTTATGAATAAAGTTAGAAGCCTGAGAATAAAGTAAGAATTCAAGAGTTACCATAAATAAAAATTAAAGGAT
AATTCCATTTTTTTTCCTCCAATTGTTCACATTAAAGTGTGTTTACAGGTCTTAGAGAGTCTTGGAGCTTTATGTATGTTG
TAAGTTTTAAGTTACTATTTTTTGTTTGATTAATCGAGATGTTTGCGTTAGTTAGATTAGAGTTTGATAGCAGTTTTTTTT
TGCTAGTTATTTGTTTAGTTTAACAATTATTTAGCATGTTTTTTTTTATGTCTAGTTAACATGTGTTGTTGTTGTTGTTAA
TTTGGGTAACACTGTGGGCACCCTAGGTTATCTAGGTAGAAGGATAGGTGTAGGACCATGATGCACCTGGGTGGGCCTATT
TTCTTTTTTTTCTTCTTTTTTCTATTTTTACCAGAGCAGGAGAGGCATGCAGGATTCCTTTGCTGCATACAATATAAGGGT
CATGCCTGCATATGTGAAAAAAATTGTATAAAGCTTTTTTGTTTCTCCAGAGGAACCTGCATGTCATCTGATAAATTGCCT
TCAGTAATGTCACTTAGTGGCTAAAGTGCATTGTGGGTAATGTAGGCTCCAGGTTTTTAAAAGGAAGAAGAATGCGTGGAA
GAAATAAGGTGTTATCTCTGGCTCTGTTGTATTGATTTAAATCAATTGTTTTTAAAACAAATACAGACTGACATCACTCAA
GATACTATGCATCACACCTGGAGCAGTAAAGTCATGGATTAGACAAGATTATAACAACTATGTAGGTGTTTGTGCAGAAAT
GTTTTGACTATTTTTTTTCTTAATTGTGGACAGTGTGTGGGAGTTTTTTGTTCTGATTCTGTCAACCCTGTTGATCCAAAGC
CGTGCAAATGGTTTGCTCTGTCACCATTATGTGACATTTGTGGAGGTCTGTCATGCTTTCTTTTCATCTAGCACTACAGCA
CGTTATATGTTTATCAAAAATAAAAATTCAGCTTCTGTGATTTGGATGTTGCTGTAGGCCATTTTGCAATTTTGGTTAATT
ATGCAGCCCTAATTAGATAATTAATGTAATTAAATGAATGAAAATAATCTGAAAAAAGCCCCCAATAAATATTCATAATAA
AACAATAAAATACAACAAAGTATATATAATATGACATATGACATATTTCTCATATGCCTCAAGTCTTATTTCTTATTACAG
TGATCCAAGCTACCAGAGACGGAGAATAAATGTTAAAGAATGAATCTAAATGCAAATGTAGCCTTTGCCCATGGTCATAAC
TCTTCAGGATATCAATAAAGCTGAAGCACACAGTCATAAAGTAAAAACCCGACGGCCTGGAAAGTTGAGCGCTGTATCTGT
CTCTGCTCAGGGGCCATCATGCCCATCACGCTGCTGTGGGCCGTGGATGTGTACGGCCGGGTGTACAGCCTGTCCACAGCT
GGCCAGCGGTGGGAGCGTGCAGACGACATGCTGCTGGAGCTGAAGCGCGTCACTGCGGGAAAGGGCCGCTGCTGGGGCATC
GGCTGCGACCACCGTGTTTACCTCAACATGATGCCCAGCGAGACCTCCATCCGCTACAGGGAGGAGACCTACGAGAACCAG
GTCGGCCCAAGTCCATGCACTCGTGATGTAGACGCTGTATGTCGTCAGTGCTCAAGCGCTCTGTATATGTGTGTGTTTTCA
GAGGTGGAACCCAGTGGACAGCTTCACTGACACACTGCTGCCCACCGACCGCTGGCCGTGGAGCGACGTGACCGGGATGAA
TCCGCAACCGCTCCACAGCTTTGAGCTGCCCTCCCACAGCTGGGAGTGGGAGGGGGACTGGTATGTGGATCAGAGCTGTGG
AGGAGAACCCAGCCAGACCGGGGTAAGAAACAAAACACAAACACGCAGTCCTTTCATTTATTTCTATGCTTTTTTTTCTCG
CTTTCTAATTTTTATGATGATGTCTAAATGATGTCTTCTCATATTGTTTAGGGCTGGGAGTATGCGGTCGATTTCCCAGCC
AACTTCTCCCCGGACAAGAAGTGGAACTCTTGTGTTCGTCGGCGGCGATGGATCCGCTACAGGAGATATATAGCACAAGGC
ACCTGGGCCAGGGTTTGTACACTCTCTGTTTTTAATCTACATTTCTGTCAGTGTGGAGAAGTTGAGTTCAGGTCAGAGTGA
AGATTCAAGTCAAAGTCTCTGTCTCTCTGCAGATCCCTCTGGACAATCCCAGGAAGCCACCACTTCCGCTCTGTGACATCA
GCTGTGGTGGTTGGGAGATGAGTGACCAGTCTGGGAGGTATCCCTACCTCTGGGGCGTGTCACAACAAGGACAGGTGAGTC
ATGTGACGAAGCCATTTTCCGCCACAGTTCACCAGAATATCAAACATAGATATCATCCCTCTTACCTGTAGTGCCATTTAT
CCATCTAGATTGTTTTGGTTGGAGACGTCTGCCTCCTCTCGAATATGATGAAACTAGATGGCACTCAGCTTGGTTTGCTTA
ACGCAAGAACTATTTTCTTTCCGTTATCACCACACAGAAGGAAGCATGCATCTACTCATGGATGAGGGGTGAGGTAACTAA
GCTAGCTAACGTTACAGCTCGGCCGAGGAGGACGCTAATAATGTTTACATCCCACACAGTCACGAGAACAAACCTCTCATC
CAAAAGTAGATGCTCGCTTCCTTCTGTGCGGTGATACGGAAAGAAAATAGCTCCTACATGAAACAGCTCACAACAAGCTCT
GTGGATTTTCTTTGAGCCCCACAAGCTGAGTGACATCTAGTTCCATTATATTCAAGAGAAGGCAGACGTCTCCAAAACTTG
GCAATTAATAGTATTTTTTGATTTTTGGGCGAACAGTCCCTTTAAACATATGGTGATTTCCTTAAAACTAATTAAACAAAT
AAGGTGCTTGACCACCTCTACACACATTAACCTGTTATACAGCCCAATATGTCTCAGGCAAGCACTCTAATTTAAAGCAAA
GATTTGAATTTTATTAGATGAAATGCTGCAAATGTGGTTCAGTCAGATACCGGAGCAACAGTCTGCTGTCCTGCTGTAAGT
CCACTTCACTTTTTTGGCCACTAGAGGGCAGCATGAGCTCAGGTAATTACTTCTTGAGGGATTTAATTCCCATTCGACTTG
ATGCATCCAAGTCTTTTTGAACACAGCTGAAATTATATTTGGAATAACAAGGTTTAATGATTTATATACATTCAATATATA
GTATTGAAGATGTATTATTTCATCCCAGCCAGTCCAGTCTCATGACTTCATCTTTACCTTGCAGGTGTGGTTCAGGGAGGG
CATCCACCCCCGGGTCCCAGAGGGCACTACCTGGGAGGAGGTAGAAGTGCCCAAGGAGGTGGCTCAGTTATCGGCCGGCCC
CGGAGACCTGCTGTGGGCTTTACTCTGGGACGGGAACCTGCTGGTCCGCACTGGCCTCAGCCTGGACAGCCCGACTGGTTG
GAATTTATTGTAGATCACATTTACCCCCAAAAGTTATTGTTTATTTTATTTTCATTCTGTTTATTTTATCACAGTAATAGG
GATATCTTGACCAAACAGCATTTTCTCTTTCCTTCAGGCATATCGTGGGTGGAGGTGGAGTCACCAGTGAAGGAGGTTGAA
GCTCTTCATGTATCAGTGGGAGTCAGTGTGGTCTGGCTGGTCACTAAAGACTACAAGGTGAACCTCAGCTCCTCACATGTA
TTAACCTCTGATTAGCTGCTGCCCTAACATATCTGTGACTGTATGTTTATGTGTTTCAAGGTGTGGTTCAGGCGTGGCGTG
AACTCCCACAACCCCTGTGGCTCCGGCTGGATCAGCATCGGAGGGGAGATGATGATGGTGGATGTTGGACTCAATGACCAG
GTTAACAGATTTCTGTTCACAGTGTGATGAGAGCCTCTGCCTGATCACATTGTATTGCTTCGTCCTTTAAAGGTTTAATTC
AGAAGACAGCAAAAGTCAGCTGCGTAGTTTAATTCTATTTATGTGGCCAATAGCACTTTTGTGTATATAATATAATTTCAA
TAACATGTCTATTACTACTGATTCAAAGTAAACCCTGATCCTGTTATTTAGGAGCTGTACCTAAACAGGAAGTCTGGATAT
TTACAGCCAGCTTTGTATCATTACAATGTTGGTCTTGTGTGCAGCTGGACAAATCCGCCCAACTCGCAGTACGTCACCCAT
CAGCAGGAGTGTTTATTGGTCCTGAGCGGTGCAATGCATTCTGGTTGCTGTAGTTTTTCTACCTTTTGAGCCAAAGGGAAT
ACCACAGCCCTCATTTCTCTGTTTTCTCTGGTCATGTAGCACCAATGTCAGGAAAATTTGAGTCTTTCTTCTGCATAGACT
GTTTGAAAAGTTGAAAGGGCCATGCTCCTAGCAGTGACATACTTCTTTAAGTAAGAGTATTTCCATTTCATATTACTTTCT

ACCTCCACTCCACTAAATGTTGCACTTTTTTCTTCAATACATTTACATGACAGCTATAGTTTATAGTTACTTCTTTATAGG
GATGGTTGAGTACACCGTTATCTGTATCCGTATCTGTACTCAGAATGGCTGGTGTTTGAACTGGAAAGGGTTGGGATTCAG
GTTGACAATAATTTGATAAATGTATAATTTATTTGAAAACTATCTATAGAAGAGCTTCATAATTGATCTTCAGATCAATGT
CTTTAATCGTAAGAGTAAGAGAAATATTTACTGATAATCTAAGTCATCAGCTATACGTTGACTTTAACTTGGGTAACATTT
TGAATTCAGGACTTGAACTTGTAACAAAGTATTGCTACACTCTAGTATGTCTATTTCTAGATACAATGAACATATAAAATA
TATAATTAAATCTGCACACTCCAGTCTTTTTGAATCCATACTATAGCCTGCTTCCACCACAGCGAGCCTCTGCTCTCAAGC
TGCTTCCCCTCTGACCGATTCACTCTACCCTGGCCTCTGGATCTGGTTTCAAGACGAGCCAGATCAGGCCTGGGGTCAGGG
GCAGGGAGTCTTCGCTCTGTATCTCACTGCTGAGGACTTTGCTAGCACTGCAAACCCACATCTCACACCTCCTCCCCTTCT
GTCTCTCACCTCCCACCTGCTCCCCCACAGTTCTTCTACCTCTTAGCCCCACTGGTGCCCTGTAACACGAGCACAGCCTGC
CGTTCCAGCCAATGTCTCGGCAGTAGCACCTCAGGCCCAGCCTTATATATCCCAAGACTATATCCCCACTGGCCTGACATA
TGGGAGACTCCCTAATTAGAAGCTGGCCAAGGCGTCGTCATCATATCCCATATTTCATATTTCAGGGCTGTAATTATGGAT
ATGGCTTGTGTGAGGTGACGGGCGCCAGTGTTACAGTATATGGCCTTATAAAGTAACAGTCGCCTACTGCCTCTAATACGC
CATTGAAGAGTGAATACACGTTTTATTTTGTTTGACATTTGTGAGGATTCTGCCACATTAATCCTGTTAGTCTGTTTATAT
TGTTACGGAATTTCCTTCCTGTGATTATTAACTGAATTTATTAAGAGGTCATATATTATGTCAATTTCCAGGTTCATGCTT
TTATTTTGGCTGTCTACTAGAAAATGTTAACATGTGTTAAGGTTCGAAAACCACATCACGCTTCTCATACTGTCCCGTGCT
GCACCACCTCTGTTCACCCTCTGTCTGAACGCTCCGTCGTAGTCACTGTAGCTCTAAGGCCCTCCTCTTTGAAAAGCCCAG
TCTGCTCTGATTGGTCAGCTCTCACAGGCCTGAGCAGGCACCAACAACCATGTCTCCGCTTTTTTTGCCTACGCAAACTTC
TGAACTAAATTCTGCTCTGCCGAACTGGGGGAGCAACTTCAGCATGTGTTCGACCTGAGCCTGAGGCTGGGGAGGGTGGCT
GGCTGGCTCTGGAAGACCTCATGTCTCGTCCCTGTTCCCAAGAAGAAACACCCCAGTGAGCCAGGTGGCTTCAGGCCAGTT
GCACTGACATCACACGTCATGAAGACAGTCATTGAACGGCTGTTGCTTCATCTCCTCAGACCCCAGGTCCACCACGCGCAG
GATCCACTGCAGTTCGCATACCAGGCGAAGGTGTGTGGGGAGGATGCCGTCCTCTGTCTGCTACACAGGGCTCACTCTCAC
CTGGACAAAGGGAGCGGCGTGGTGATTTTTCCAGCATGAGGGTGGATGCAGGGGGGTGCAGGTCAAACCACCTGCAGCTGA
ACATCTACAAGACCGAGGGGATGGCGGTGCATCCTGTGTCCATCGAGGGGGTCGATATGGAGGTGGTCCAGAAGTACAATT
ATCTGGGGCTGGATTGGTCAGCAACCATCGATATTCTGCACAAGAAGGGGTAGATCCGTTTGTACTTCTGAAAAAAAAAAC
TGTAACAGATGTTTTTCCAGTCTGTGGTGGCCAGTGTCCTTTTCTACACTGTGGTGTGCCATCCTGGACCACGCCCACCAC
CCTCTGCACAGCACCTTCACCAGGCAGAGGAGCATGTCAACGGAAGACTGTTGTCCCAGTCCTGCTCCACTGACAAAGTCT
TTCGTCCCCCTGGCCATTAGATTGTACAACAACTGTTAGTTATGGCAGGGGGAAACAAACAGACAGGCAGACTGAGGCTTG
ATTACCTAAAGCATTTTTAACATTCAACCGCACATCTCTTTTCACAGAGACAGCTTTTTTGCACTTGAATGCTTTAATGCC
AATTTACATGAACAAGTTTTGGGCAGACTCTTGTTTCATCTTTTTTTTGTCAATTTTACTTGAATAAGTTTCGTATGGACC
CATTTTTTGGTTTCAACCGGTTTCATTTTGCACAACCTACATTCAGGCACGGCTGTTTTGTTTACTGTTATTATTTTTTCT
TTGCAAAGTTTTAATCTAATTTAATATTTACTACTACTACTACTACTTACTACTTTCATACTGTTTATATTTGGCGGACCC
TGCCACCTTTCTAGCTTCAAACAGTGTTCTGGGGACCTTATTTTCCTCTGAGAACAGCTTGTTTATTCACTTATGGAAGAA
AATAAGTATTTTGGAGTTTGTGTTATTACCTCATTAATAGTAAAACATAAAGAGTTTGAATTTCTTCTCCATAAACTACAT
AGTGCCCCTTTAACGGCGAGTAAGATGGCATGTTACAGCAGGTATGTGTGTGTCTGTGATCATTGTTTTTCACCTTTGTTG
TCGTACATTGTTTTCTTCTCTTTATCTGTCAGGTGTGGGCAGTGGGTGAGGACCGCGGCCTGTATTTCAGAATGGGTGTGA
CCCCGTCAGAACCAAGCGGAAATGGCTGGATCCCTGTTTCTGCCCAGTGGGGCAAAAGTAAAAAGGTTATTCCACCCAGGT
CTGTATGAGTAACCAAGTGAAATCCTCCTTATTCTTGTAATGACACCAACTTGTTTATACCAGTTTTTTCTTTCATTATGT
GTTTTGTTGATTAGTAGGAAAGCGTGTGAGCTCAGTGGCCAGCTGACCGAGGCTTCAGTTCTGAGCTGCACCGACTCAGAC
TCCGAGTTAGGTCCTCCTGACCCACAAAACAGCACCAATGACACCCCAGTCCTAGAGGCAGCAGCGCCCTCTGTGGTCCCT
TTAGGGGGAGCCGACTCACCCTCACCTCCCCTGAAAACAGAGGACGCATCTTCAGCCTCCCAACAGGACGCCCCCAAACCC
TTCATCCCTGCCAGCGACAGCTTTATCAACAGCCTGGTGTCAGACCGCGACAAAACGTCCACCACCCAGCCGTCCATCACA
GAGGCACCGCAGGAAGAATGCGAGGAGCCGTCCCCGGCCCCCGTACTCCCGACGCCTGTGACTGAAGGACATGATGTCCCC
TGGATGAACGTGGACCTGGAGGGGGCAGAAGCGGCTCGGAGTGCCCAGGCAGCGGGGCTCTCACTGGGCGATGGAAGCACC
GCTGCGACTTACTCTCTGGAGACCTCTCAGGGCGTTGGAGAGGAGGACGGGCCGGTGTGGACCTGGATCTCTGGTGGAGGC
TGCGACGTGGATGCCAGTTCACAGATCAGCTGGCTTAGTCCTACAGGTATATGGATCTTCAGATTTATATATATTTAATTA
TCAATGTGCAAAAGTCCAAAACACAAACGTCAATTTTAGTTGATTGTCAGTTTATATGTGTATATCTGTGTTGAACGTTGT
ACACCGTGTGTCTTTGTGGGTTTTACATGTATAGAGATATACTTCACGTGTCATCTCTATGGTTGTCCGTCTAAATGTCTC
GTTTGTACGTCTCTCTGTCAGGTCCTCTCACCAGCTCCCTGTCTCTGACTCCAGTGCAGTCAGCAGCCTGGAGCGAACAGC
AGCAGCAGCAGCAGGAACACAGAGAGGAGATCAGCAAGAAACCTCTGGAGAGAAGCAACGTAAGACTCCTTTTACTTCTCT
TTAATAATCATCGATCGGACTGCATCTCCACTCTGGTCATTTTTTTACATGTTTGTGCGTGCATGTGGTCAATCCTCCAGT
CGGTGTGGGTGCGTAAAGGTGCGCTGCGCTGGTGGAGAGACTGGAAGCCTCAGCGCTGGGTGGATGTGGGCGTCGCTCTCG
AGCAGTCCACCAAGTCTGACGGCAGGAAGGACAGCATATTCTTCGTCTACTACACACAGTATGATGAGAAGAAGGTGGGTT
TCGCTGTCGTGTTATGCTCAGGTTAAAATTCTTACAAACAGCACTATGAGTAAGATTTCCTCATGTATTTTATTTATTTTT
CAGTACCTCCATGTGTTTATAAATGAAGTGACGGCGCTGGTTCCAGTGCTCAGGGACGGCCACTATGCCTTTGCCGTGTAC
ACAGCTCAGAGGATCAAACAGAGGTGGCCTCTGGTGTTGGCAGCTCAAACCGAAAGGACATGAATGAATGGGTAACAAAG

AAACACAGAAAATACATTTGTAGTTTAGAAGATTTTTTTATTATTATTTGTATTTTTTTTAGTTGATACAGCAAAAGAAT
ACACATAACACGGGACATTATGATGACAGAAGTGAAGTCACACAGTTGAATTGTGCCACCGGAGCCCATAACTGAAACCTT
GTAGAGCTGATCTCGCATATTAGCTAGTTCCACCACTAGCTAGTAGTGGAAGGGGTGGGACTTAGCCGCTCTACATACACT
CACAAAAAACAAAAAAAACAAAAAAGTAGATAAACTAAAAAAAGACAACATATGAATATGCATCTGAATGCATATGAATAT
TTCATCTAACAGAATAATCTTCTGTCTGGAGACAGTTTTCATCTAGTATTTGAAGGTAGAATATCTTTAATTGTAAGATTC
TGGAGTAGGAATCGTTTCGTCATATTGTTCTGAAAAATATTATATTAAAGTAGTAATTCATCTCCCCCAGACGAACAGACT
GAACTTTATTTTGAAAATTATTTTACTTATCTTTGTTATGGGTTGCTAATTTGACACCATGTTGTAGATGCACTGATCTGC
TGGCTTGTTTTAGTTTCCATTTCCATCTTTTACATCACATGTACTAGCTGGAGCTCACTGCTGATCACATCCCTGCTGTTT
CTGGGTGTGTCCTGTGTCTGTAGCTGTCCCTGTTGTCTGACTGTTGCTGCGAGTGTCGAGGGATCACAGGTCCCCCGTCCA
GACACGCCCTGTGGTCCACAACCTCAAAGGGAGACGTCATGGTCCACGAGCCGTCCTTTTCCTTGGAGGCGACTGCGCACA
CACCGGCCTGTGACCTCATGTGAGACTGATACCAAACAGCAGTCGGCCTGTTTTATTTGTTTTTATTCTTCTCTTTCTCTT
CACCTGGTTTCCCATCTGCCACATTAATTCTGCTTCCTGTCAGTTAATATGTTGCTAACGTGCTGCTCTGCATGGTGACGG
TGTGTCTCTGTGTGGTGATGATAACGTGGCTAACGCTGGGTGCTTCCGCTGTGTGGTGCTGCAGGTTCTGGCGGCAGGTCC
CGGGTCACCTGCGCTGTGTAGAGTCTAACAGTCTGGGGCTGGTGTGGGGCATCGGGTGGGACGGCACCGCCTGGGTCTACA
GTGGGCGCTATGGGCCCCAGCCCACGTCGGGTAAACATGCCCCTGTGGACATATATCAACCCTTATGTTTCCCACTTCATG
TCCTTTTAAATTTCCTTTCTTCCCAGTTTGTCACGTATTTATTACAAGTGTTGAAACGTTGAAAACAAAACGTCTGGAAAG
AAGAGTTTAAGATAGCCTTTTTAGGCCAGGCGACCATTTTCCTCTGACTTCATGAATTTATCCTTATTTGTATTCATATGC
TAAATGCTTATTCCATAATCTATAATACAACAATGACCATTCAAATGTCAAATTGATGTCAATGTTAAATATTACCTTGCA
GAAGAGAGAATTGATAAATCAGTCAATCTTCTTGCTGACAGCTTGTAGTCTGTGTGCAAGTGCTTAGCATGGTTCAAGACT
TTACGATCTGTTGCAGCTGTAGACACATGTATGCACACAAATGTATATCTTTACTAGCATTACATCTGTGTCTGTATTCGT
GTTTCCTGCAGAGGATGCAGTTCAGATGCACCAGCAGACTGATGTCAGGAGCGTTCATGTGTATGAGAATCAAAGGTGGAA
CCCCATGACGGGATATACAGACAAGTAGGAACATTTCATCTTTTACAAAGTTTGATAAAGTCTTTGCAGCTTATCTTGATT
TTTGTGTCCGTGCTGTTTGACTGCTCCTAAAACATGCAACACTTTTGCTCCAGAGGACTTCCTACAGACCGCCCTATGTGG
AGTGACGAGAGCGGACTGAAGGAGTGCACCAAAGGCAACACACACCCGCCCTCCCCTCAGTGGTCCTGGGTAGGTGGGCTC
GAGTCGAACACACTCACTTCTTGTCTCGTTTCACCAGCAGCTAATCCTAACTGTGTTTCAGGTGTCCGAGTGGGCTGTTGA
CTACAACGTCCCTGGAGGGACGGACAAAGAAGGCTGGCAGTACGCGGCAGACTTCCCCGTGTACGTATATATTATTTCTTT
TTTTAAAAATCTAATATACAAGAAGGACATCTGTGAATTAATCTGACTCATCTCTCTCCATCTCTACAGGACGTTTCACGG
CCACAAAACCATGAAAGACTTTGTCAGGCGCAAAAGATGGACGAGGTAATTAAAAACACACTCTGTTACACATTTCTGTGG
TTTCTTCAGCATTAAATTAATCACATATCCTGCGTACAGGAAGTGTAAGATCTCACTGAGAGGTCCATGGCAGCAGGTCCC
ACCCATCCCTTTGACTGACATCTCTCTGATGCCATGCCTGGCCCAGAGCAGGATGGAGCAGGTGCCTGTCTGGGCCCTCAG
CGACAAGGGAGACGTCCTGTGTCGTCTGGGAGTCAGCCCCCAAAACCCTGCGGTGAGAAAACAACTTACTTCCTTTTAGAT
CGTAACCGAGAGAGACATACAGTGATTTTCAGATCTACTGAATATTCCTAGATGTTAGATTTTTACATCACAATAACAATA
ATTTGATTGTAATTCGCTCCAAACGTTCTTCTTTCACAGGGCAGCTCGTGGCTACACGTGGGCACAGACCAGCCCTTCAAG
TCCATCTCTATCGGTGGAGCCAACCAGGTGTGGGCCATCGCCAAGGATGGAGCTGTGTTCTACCGAGGATCTGTTTCCCCG
CAGAACCCTGCAGGTCAGCTGAAGATGTGATACTCGCAATGAATCTGCGAGATAAAAAGTCTAAATGTTCAAATTCCTATC
AAGGGCCAATGAAGGTTTTGTTATTTGAGTACATGCACCAGGAAGTTAGAGACCAATTAGAGCCAGACTATCAGCGGTCTT
GTCCCTCTGCAGGGGAATGCTGGTACCACATCCCGTCTCCCCCTCGACAGACTCTCAGACAGCTGTCTGTTGGCCGGACCT
CCGTCTTTGCTGTGGATGAAAACGGTGAGTAGCAGACTTATCTTTTATTGAAACCAAGGACTCATGGAGATAATTATTTCA
AATCCAAACCTTTTTGTGTTCATGAAGGTAACTTGTGGTACAGACAGGGCCTCACACCCAGCTACCCACAGGGCTCCGCCT
GGGAGCTCATCTCTAACAACGCCACCAAGGTTTCTGTGGGGCCGCTGGACCAGGTCAGTGTTTCTACAGCCTCCATCTGGG
CTTTGAGGACGGTGATTTAGCCACCAGAGACAAGTTTTATCAGCGTGTGACGGGAGTGATTGTTGGATCCAACTGTGAACA
TGATTGCTCGGTGCTGTTGTTCTCTTCAGGTGTGGATCATAGCAGACGGAGTTCCAGGCTTCCCCGCTGAGACTCCTGGAG
CTGTCTGCCACAGGCTGGGAGTGGGACCCATGCAACCTAAGGGCCAGTCCTGGGACTACGGCATAGGGGTGAGTGTACATA
CCACTTCTTATTCAGGTTGCATTAAAAACATGGAATGAAAAATACAAGAAAATCACATACTGTTGCCTTTCTACTCTGTAT
TACTGTTTATGTCTGCATTTTGAAATATATTTGCTTTTAATATTTACATTCAGTTTATTTAAATTAAGCCTAAACAAATAT
AATCATAAAGAAATAGCCCCAAGCTTATTGGTTCCTACTGAAAACTTTAATCTTTAAAAGGGGCAGTTTGTATGATATGGC
CAGAATTTTTGTTTTAAAACAATCAAAGATTAACTCAAGTTAATCAAGAGTATGAAGAAACAACAGGGTTGATGTCAAAGA
TATCTATGTATTTTGTAGCAGAGATCTCCACTGAAGTTTGCATGCTAACAAGCTAGCAGTGAGTTTGCTACTTCGTCTAAT
AAGTATACAAGTATAACAAAATAAACTCACAAAAAGGTCAAAAAGGGAGATATTTTTACACCTACTTTCCCCATTAGTCAG
AAAAACATTGGAATTATGCTGCCCCCTGTAGTTTATATAGTTTTCTGGCCAGTTCTTACAATTTTCACCTTTAAAAACTGC
TCACAATTATATAGTTATAACCCACCACCAGACAACCAGCCAAAAACAAGGGGCAAAAAACACCAAAAGTTCAGCAGTGTG
CAAGGAAGTCAGTAGAGAAATGAGAACATCAGTGCCTGAAAGCCTTGATTTGCAGTATTGAAAGACTTCTGTGTAACCTCC
AGGGAGGATGGGAGCACATCAGTGTGAGAGGCAACTCCGCAGAGGCTCCTCGCCCCCCTCATCCTGGCGCCGCCGGACCCC
CCCGCAGCCCACTCCCCCTACGACCTCCGACACAAGTCAACGGGAACGCTGTGGGTGTGTAGCACCCTCCCATCTCCTCCT
CCTCTTCCTCCGCCCCCCAGCCCTGTTTGTGCCTGGATCCACATCTGTCTCCTCGGACCATTCCCACAGGCCCTTATCCCT

TCATGTAAAGGGAATGCACAATCGTACCACTGAGTTTATACCGTATATTTTTGGTGGATTTGACTCTTGATCGATGGATGA
ATGTTATTCCCTGGGGATGGTTTCCCAGGCGGAAATCACGTTGGTATGCAGTGACGACAGAGAAGGAAGAGAGCACAAGAG
CATTTAGGTAGAATGGATCTTAATGTCTTGTGGGATCAAAATTTAAAAGTGAGTGCATGTGTTTGCTCTAGGCTTTGGCTA
CTGGGTGTTTTGGGATCTAGCAATAGCTTTTTCTCTTATCTAAAATATCCTGTGAAAACTTAAGAGGGCGGGTCTTCAGTA
AAGGGCTTGGGTAACTAACGTAAACACAGCCCCACTGTAGCCGAAGTTGTCATGGTTTCTAGAATCTTGTTTTGTTTGTTT
TTGTTTCTGCCTGTCATACATTTGCATGTTATTTATTTATTTATTTATTATGCATTTGTTGAATTTTGTGTCTCCATATGT
TTTGGCCTACTATACGTTTGCAATGGAGTGTTTCTTGCCCGTGTTGCTACAAGTCTCCACAACATTGGAGGCTTTTTTCAA
GATTTCTCTTCTCCATACTTCAAGAAAATAAAATGCTCTTTCAACTACAAGTGACCACAGAGAAAAATATGGAAAATTCAG
TCCGCACTACCAGATGATACCTCTCTATCCGCCTGCAATAAGGAAAGTTCTTCCCCTTAGTATTATATCCGTACAAATAAC
TGCTATGACCACGATGCGACCGTGACTGTTGAAACTGTGCGGCAGGTCTGAATGACTTGACATTCATCTGACAAGGGCATT
GAAATGTGAAATAAAAGTTTAAAAATATTCTTTTATGTTCTGACATCATTTGAGTCGCTTTGGAGATCAAGTCGATTGCAG
TTTGTGGACTGAGAGCAGACAGAAATTCACTTGAGTTTGAAGTTTTTATTTTAATAAATGGATTGTGATATTTGTGGAGGG
CGCCTTTATTTGATGGATGTATGCATATAGTATTTATCATACATAGTTTAGAGTACGTTATAGTAAATCAAAAACGAAACT
GATCAAATACAAATCCATCTGCTACTTCAGCACCACGGACAGCGCCATGGATTTATCAATACACAGCAAAGTTGGGAATAC
TACAGGCAGCTGTGTGTCTCTATGATCCTCTTGCTCTTCCGAGACCATTAAGTACTGTTAGTATACACTGATCAGCCAGAA
CATTAAAACCACCTACTTAACATTGTGCAGGTTCCCCTTGTGCAGCAAAAACAACTCTAACTTGTCAAGGCATGGTCATAA
GACCTGTGGGGGTGTCCTGTGGATCCTTTGGGTCCTGTAAGTAGGGGGTGGGGCCTCTGTAGATGGGACTTGTTCCAGCAC
GTCTCACAGATGCTCAATGGGATTGGGATCTGGGGAATTTGGAGGCCTGGTTGACACCTTGGGGTCTTTGTTGTGCTCCTC
AAACCAATTCTAAGCGGTTATTGTCAGTGTTATGGCGCATTATCCTGCTGGTGGGGAGTGCCGTTGCCATGAGGGGCTGTA
CTTTTTCCACAACTGTGTTTTTGATGGGTGGTGCGTTTCAAGTGGCATCCACATGAATGCCAGCACCCAAGGTTTGATCGG
TGTATAGTTATTTAATATTATAGATCTGTGATGAAACTGATGAGACTGAGAGAAAAAGGTGTTATTACAAATCCAACTGTT
ACTTCAGCACCGTAGACTTATCCCTTCACTGCACACTCAAGAATACTACAGGCAGAAGTGTGTCTCTATGATCCTCTCGCT
CTCCAGAGACAACACGATGCTACTTGTCACATTGATCATGAAGGTGACTCTACTGTCGAGGATGGTGTTTGGACGGATCGA
AAGGTCTGGACCCAGGATACAACGCAGCATAGCATTAAGTATTTATAGTATATAGTTCAGAGAATATTATAGACTCAAGAT
GAAACTGATAGGAGGGTGGGACTGAGAGAATAAAGGTGCAAATATAGCACCATGGATTATCAATACACAGCTCAGTTTGGC
TACTGATGTTTCAGAGACTTGGATTCTAACTCTCACACACCTCAGTCAGATAACAGATCAATGAGTCATGCAGACCAGACT
ATGATTTTCAACTAAACGACCCCTCTGCCTCTGCCCTCTCTCTGCTACTAGGGGATACCGAGAAGGGGGATGACTCACCTG
AGGCCAGAGTATCCCTTCACCTGCCCAAGACTTGTCACAACAGCCAACTCCTATCCAGGAGGTTAAGTTTCAAAACCGATA
ACGAGCCAGCTACATCTAAATGATCCCTCCCAAAGTAGTTCTGGCTGGAGACCCGGTCTTTGTGGCCAGGGGTCATTCCTT
CCCTGCAGGACGACTGGCAAGGCACCAGACCTAGATTGTGCAGCAACCACACAGAAATTAGAAGTTCATTTGCATTTATTT
TCATATTAAACAGTGTAATTATAAACATTTCATTCAATTACACTAAGCATGATAAATATGAAGTAAAAATATTTTTAAAAT
CTACAAGTTTAAAGGACAGAATGATTTCACATCTGCTTTTGTGTCTTAATTTAACTGCTTTTAGCCAACGACTGATATGTT
CCTCCGATCAACTCAGCTTTAAGTTACAAAATGAAACCTCATACCTAGAACTCAGTTACATCATTTCCCATTTACAGACAG
AAAAAAAATTAACATTTACATACATCAGTACAGTTCAACATTAAGTTACATTTCTCAAATGTGCAAGATTAAGCAAATGTA
TACTGTAGTCAAATAAAACAAGATTTCCTTAAATAAATGTTCAAGTTTAACTGAAGCACAGGCTGCTGGTTTTTAACAGAG
GTTAACATTATCTACTGTACAGTGCCTTCTGTATGGGGCGGTGGAGATGGACATTATCTACTACAGGTGAAGTAGTTTGTC
AGGTCATTTGTGACAAAACCTTTAAAAAGAAAGAACCATACAGTAACTGTATAACTTACAGTTTGCAGGAACGGTGTTTTT
ATAGCTGTACATGAAAACCTTTTGGGGGGGAAGGGGAGAGGGGGGAGCATGTGTTGCTCCTCAACTGTTTGCATTATGCTT
TGTACAGAAACGCATCCCCAACAAATCCTCTACAGCCACAGAGACGATGCACGGCCTGCAAAATGATATCATACATTAATG
AAATGATGAATGCTTAAAATTGCAACTGGACAATGGGTCCTTAAGGAGGAAAATGCCATCTGAACAGGTTAATTAAGTTTG
ACACAAAGTAAAAAAAACAAAGGCATGACTCTGTTCATGACACTGACGTTGATAAAAGCAGCAGTGTTTTCTGTTTTTTTT
TTTGTTCTAGTAGTGAAGCTTTAAGTCCCTGCACATTACCGTAGAAACGCCACCACGTTCACCAGGTCTTCCATTGAACTC
AAATTTCAACTGATTAATGAAACTCATTGCACATTTGTTTACAAACAGCTCATCACATTTCTCCATTCTGACATTTAAAAA
TAAAATCTCCCACTTTTTACACAATGATTTTAAATTTTGTAGTGATAATAAACAGAGGAGTGCAGACTTTTTTTCTTTTTT
TTAACAGCATCACAGGCACTGTGATAAAACCCCAGTGACGTCCCAGCAACTTCCTGCACAACCTCAGAGCAAAAAAAATGA
AGCATTACAAAATGTTTTTAAAGTTCTTCCTCACCACTGTCTTACAGCTGGACCACCTCTGTAAGGATTCATACGACAATC
CTTATCTAATAACACCAACAGTTCTTACTGACATGTCTCAAAAATATGAAACTACTTCCTGTTAATGTTCCTTTCAAACAC
AAATAAAAGGAACATACAGTGTGTCACCTCCCACATGAGGGATTTTTAGTTAAGGTAGTTATCAAATCATCCTATGCATGG
ATGGTGCGTCCTAAATTTCAACCCTTCTTATTCAAATGCTGCTACAGAAGGATACCACACATATTGTCTCTCCAATTAGGA
GACTTTGACTAATACCCAACAAGAGTTTACGAAAAAAAAAAAAAAGGCAACCACTGAAATAATCGCACAACATGAAGGAGAA
AATTTAAACAATTTAAGAGAGCAAAAAAGCTTGGATCTTCAGTTTTAGGTCTGCTGAAATCGAAGAGCTTATTCCTATTTG
GCTGTAGTGATAAGTTTGTCAGCCCAGTGTGTTGAATTCAGAACATAAAGTCTTTTTTTTTAAATCTTTTTTTTTGGTGAT
GCAAAAGTCCTGGAAAAGGAGTGCACTTGGACACACAACAAAAAGAAGCAGCCAAACACCTGGAATGCGCTTGATGAGTCT
TTAAAGCAAATATTTAGAAACTTTTTTTTTTCCCCCAAGTGGAATTGCACTGAACTGAAATCATACACACTGATGTAGCTG
GAGAGGTGATGTGGCTCAGACCGGGACTGGGACTAGTCTGTCTTTATTTGCTGAATGTCAGTTTGCAGCCATTGTTCTCAT

GGCCATCTCCATGTAGGTAAAGATCAGTTCTGCCTGAGAGAAGCATCCGCGGTTTGTCCCACAGTTCTCCTTTCCCAAAAA
GTGCGAGTATAGGTTCATTTTTTAATAGTGTGATGATGAATGGTCCCTTCATTTATCACACTAGTCCTTTTCTCCATCTTC
ACTGCTTCCTGAGGTAATAAAAAGAGATTGAAGACAGTTAGATTGCGCTCAACTGATTGATGAGTATTAGTTAAACATGGC
TACTTCATATTATTACAGGGCTCAATGAGCATGGTGTATGTAAAACCTGATAATAGTAGTCAGTGAAGACATTAACTAGGA
AAGTGACAATGTCGTAAAGTAGTAAAACTAATATCATCTTAACACATTTTCATTAAGAAATGCATATAATGACATTAAAGA
TTGCAATGAAACAAAACCTTTTAAAGGAACAGTTTTTGGAAAAGACAGGGATCAATAGAAGGCTGTTAGCTTAGCATAGCA
TAAAGCCTGGAAATGCAAGCATGGCTCTTGTGATTTAGATACACTATATTTCGAAAAAATTGCTAGACTTGCCTCCTTGTT
CAATGTCAGAGTCAGTGAGATGTGTGAGGGAGAAGCTGGCGATGCTGGCTGGTGAGATGGAGAAACGGGAGTAGTTTGAGG
AGCTGTTCCAGCTGGGCTCCAGCACACGCCCTACTGCGGGGGGCGGAGAGGAAAAGCGAGATGCCGCCGATGAGGACATAG
CCTTTGATACGGGGTCTTTATCCGTCTTGGGCAGGAAGGCAGGTGAAGGGGAGGAGAAGTCATCATCCCACTCAAAACCAC
CACCTGTTGGAATTAAATGAACCAGTCAGAACACGTAATGCAAAGACGATATTCAGTAGTCTTGTACATTTAGTGGGTTTT
GGCCTGACCTTCTTCATCTGTGGAGCTCTCGGAGTTGGTGAACCGGTTCAGGTAGCTGGCAGTGGACACCGGGCTGCTAAA
CTCTGGTACCTGGCTGTTTGAGCCCGAGGAGTGGTCACCCAGTTCCTTGGTGGGCGTCTCCTACATCAGAATGAGTTGGAT
TTTAGTTAGAATGGCCATATGAAATCACCTAGCTAGTTACACCTACAACAAAACAGACAATACTTTACCTGGTCAAACAAA
TAGACGGTGACATCATCGAAGAAAGACACAGCTCTTCTGGAGTTATCTGCATTGCTCCTCGCAGCAAACGGGGAAGACGAA
GCCTCGACGTTCAGCGTTGTTGGTTTCAACAAGCTCTTCAGGTTCTTTGCACTGCTGTCATCAGATACAATAACTGGCACT
GTGTGCACGACGTCGTCCTCACTGTCTGAGCTGGAGCTGTGCATCTGGTATGCACGGATGTCATCGTCAGAGTCATCGCTG
TTCTCGTCCTCCTCATCCGCATCCCCGCCATCCTCCTGTCCATCTAAACCTGAGCCGTCCCGCCTCCCTAGGTAACGCCCC
TCCATTTCCGGCTCTTTTATTTTATGACCATCACTGGCGTAGGTCCTGGTTAGTTTAGCGTGACCTGTGCTCTCTGCTGTG
GGCTTTGAGGGGGCAGCAGAAGGGAGTGAGGTTGAGGTTTCTATATCTTCTGATGGGTCTGGTTGATCAGGAGATGTAACG
TCCTTCAACACCTCTGGTTTGATATGATTGTTGGTAGCCTCTGTTGCTTCCTTCTCACATTTCTCTGTAAGATCTGCCTCT
GGTAATGTCCCCCCAACATGAGGATCAAGCGTTTCTCCATCAGCCTGTGCTTCAGTGGTTTCAGAATATTCTTGCTGAGAA
AATAAAGAATCAACATCTCTCTCCACGTTAACCTCCAATGGTGCACTTCCAGCTGCCTCGCTGACCGAGTTCCTCAGCCAC
GTGACTTCACCGGAACCTCCAGCAACTGTTTCTTCAGTCTTTTTCTCAACTTCTGATTCATTGTCTGAGAAGTAGGCAGAG
TCTCTGTAATTACTGCCCATGAGTGGTTCCTCCGCTGGGGCTTCATGATCTGGTTGCTGGTCCTCACTAGGATCCTCGCTG
TGAGCGTTCAGAACACCCTCTACTTCAGAGATAATGATTTCTGGTGGAACCAGATTTGTCGCAGCCGAGGGCTCTTCCGTT
TCCTCCTCTTCTTTGGCAACACTCAAGCCATTTTTGACAGGGGACTGGTCTTTGACGCTAGGCTGAGAGTTCCACTCAGGA
GACTCCAGGTTCTCCGTCTCATATCCACTGTCTGATATTTTGTATGGAGGCTGGAGTTTGTGTTGGGCTATTTGAGCACTC
AGCTCTTCTCCGTGTTCACTTAGCCTGTGGATGTCCAGAGAGTCTAAAGAGTCTGGTGTCTCGGCTGAGTTATCAACAGTT
GGTAGCGTGGTGGACATACTGTCTTCCAGTAAACTGTCCTGGCTGATCTGGTCCAAAGACGGCCCAGAGACCAACAAAGAC
GATCTGACAAGAGGACGCCCATTGGTATCCATAAAGGATTCCTCATGGTCAGACAGAGTGGTTTCTAGTTCTATATCTGCA
TCTTCAATTAGTTCACTCAACAGATCGTTACTTGTTAGGTCTTCGCTGTGTGCGTTGTCTGAGGACAATGCTTGGACTTGC
AGGTTTTCAGGCATTTCACCTTCTATGGGCTCAACATTATCCTTTGAGGTTCCGAGGTCATCTTCAAGGCTGGGAATTTTG
GCATCTGTGCACAATAAGTCTGTAGTGGCACTTTCAGAGAATGCAGGGGTGTGGTCATCAGTTTGACTGATGATAATCTCA
AACCTCTCTGAGGTAGAATCAAAACCAGAGTCTATACCTCTGTCTGAGGATTGCAGCAGACCAAGGTCTTCGGTGGAAGAA
CTATTTCTAACTGGCAGCTGGTTGCTTGTGTCGTCTTCTGAGACCACTGTAATGAAAGGCACCAGTGTTTCATTTTCTACC
AGAGACTGACCAAACTCCCCCAAACTGGCACTTGGAGAGTCTAAAAGTTCCTGTCTTGAGGATGTGCTACTTGCTGCAGGT
TTTAAATAAGTGTCCATTGTGGTTAAGCTGCCTAAACTCTCAAGGTTGTCCCAAGAACGCATTCTGAATGTGTCTTCTGGC
TCGTGGGCTACACCGGGTAAAAGAAAATTATCCTGTGGGCTTGAGAAACTAGATCCATCTTTCATCAGTCTCTGGTCTTTG
AGAAACATGAAGTTGTCAGCAAGTTTTTCAGTATTCAGAAGCCTCTTCTCATCTGAGTTCTCCCCCTCCACTTCCGCAGAA
TCAGTCAGGTGAGGGGCTTCTGAACCATCCTCTAAAAAAGAATCCCCATAGCTTTTATCGGTGTCCACCTCTTGGACTTCT
AAAGGCGCACCTTCATGGAAAGGGTTCCCATTGCGTTCTGGAAGCTCCATAAAACTCCGTCTCCAGGAAGGAGAGTCCTCT
TGCTTGGACCCCCCTTCATTGAAGATGTTGGCGTGGTATGGACTGTCATTTTCAAGAGATGACCAGATGTGGCTGTCAGGT
AAATATGAATCCTTGGAGTCAATACTTTGGTGGAAAAAATCAGCATCGGTGCTAGATTCATCAAGACGGACATCTTGGAGA
ACGACAAAGTCTTGCCGGCCGGAACTGAAGTCAATGTCTTGCCCTCTGTTCCCATCTTCCCCAACGGTGCTCTCTCCTTGT
TCTTCCAGTTGTATGTAAAATTCATTTCCATTCAATGGCTTTTGTGCGTCAAACACTGGTACAATCCCAGGAATTCCTGAA
GAAGTGTTATCACTTTCTTTGCCCGCTGCTCTAGCTGCAGGATCGGGGAAATGAGCCTGAATGTCTTCACTGGAAACTGGA
AAGAACATGCTGTGATAGTTCAACGTTGTGTCCATGCTTGAACGTCCATGGTTGCCTTCGTAGTGGTCGTGCTTGGCTGCC
TCCCAAACGTACTCAAAGCTGAGGCCTTTGCTTGTTTCAGTGACAGTGAGGACTTCATCGATCTCTTGTCGCAGGGCATCA
TCAGCAAACTGTTCCAAGATTGGGAAAGAGGAGTGCTGACGGTGGTCTGCCGTGTGGAAGGGTTAGGCTTGAGAGCATCCC
AGCGTTGTTCAAAATCTTCCTCTATGTCCTTCTGGCCTTGCATGCGTAGGTAAATCAGCAGACGGTGCACTTCCTCTGCTG
TGGCTCGCTTGTCTGGAGACAGCCAGCAGAATTGCAGGACCTCATACCTGCAGTTACAGGGCAAAAAACATCTCTTAAGAT
AATGGCCAAAATCTTTTTTTTTTTTTTTAAATCAATATTTAACTTATAATAACTTTTTGTTTCATAATATTATTGTTATGT
AATTTGTTTCGAAGACTCACCATCTGTCAGAATAAGGAAGCTCCAGCTGTGGTTTAAAGAGTTTGACTTGTTGGTCCTTGA
TCACATGATTAAGAACTTCCCGGTCAGACAGATGTGGGTACGGCTGAGCCGCGTTCTCAAAGAGCTCCCACAATGTGACCC

CCAATGCCCTACAGAGAGGTGAGGATAAAATAACTGTTGGAATTTACGGCAGGACAAGAGTTATCTGGCACACTCCAAGTT
CAGCAAGTGACATTGACCTCTTGACCCAAAACCATAAAACTTAATGAAGGAAACTGCTAAAAATATCCAAACCCATGAAAA
CGGCACAGTAACGTGGATTATGCTACCATTGATGCTATTTACATGGTGAACATTTTGAATCTCAACATATTTCTAGTCGGG
GGGTTGGGGGGGTGGTCTGGCTTATGTAGCTACAATTACCTTACATAACTCTGCAATTTATGATCGGAAAGCATTTGGGCA
ATGAGTCGTGAAGTCAAGGAGGAGCTGAAAAGACTGAACAATGTGGAGAGATGCTTCGCTGGTGCACTGTCAGTGTCTTTT
CCCCTCTTTTGGAAATTATCCAGATTGCTTGGGATTTAACAGCTCTGCAATTAGCAGAAATGGCTTGTTTTCTCAGCATGA
GCCAGAGGTGGACTGCAGAGCTATTTACTGCCATACTTCATTCCAAACGTGTATATCTTGATTACAGGACATGGCTCCAAC
TGAATTATCTGCAATAGGGCAGCATAAATCTCTCCTTCTTTCTCAGGAGTGTCATTATAAAATATGTTTGGATCAGATTCA
GTTTCACTCACAGTCATTATGAGTAATGTGTTTTTAAAAAAGGTCCTCTAGTGTCAGTGAAGAGCTACAATCTGGTACCTA
CCAGACATTGCTGGGCTTGGTCTGCTCCAAAGTAATCACACCCCCATGGCGCTCACCCACCAGCTCTGGAGCCAACCAGCG
AAGGGGAGCGAACACATCATCCTCTGTGATGATGTAATCCTCCTATAGTCACATTGTAAAAAATGTCAGTCACACAACATT
TTTGCTACTTTTACGAACCGTAACTTTCTGGAACACACTGTAAATAATAGAGCAAACACTTACACATTAGATACACACAAA
CACGTCATTAAAAAGCACTCAATAGAAGAAATGTGGTTGTTTATTTGCTCTCATTGAGGCAGTGAACTCTCACTTCGCCTC
TCCCTTTCTTTACAGAAATAGATTCAGTCAGTGACCTTATGGGTATTAAATTCAGACAGCCAAACTGATGGATCAGCAGTA
TTTAAAGAATCCAGTAGACAGAACTGCCTCAGGACATACGCAGCGTGTCAAGTTACTAAAGAAGATGGTCTCAATTCTTCA
GTGAAATATACAATTGCTCAATTGGCAAACACCTGCGCAGCCAACGACGAAGATGGTTTATTTTCAGTCAGGTCATCTGGG
GCTGGTCGCACAAAGATAGACTTTGACAGTGACTTCATTACAACAAACAGCCAGACGTCAGACAGGCAACTTAAGTTCATC
AGTTGCGCAAAGAGGAGTCGTTCTTAACCACCTTAAGAAGGACGCATTAGTTGAAATCCTGCTTCAACTACTAAACTCTCA
TTTACGTGTGTTTACATTATGTGGCTTGTGGAAGGATTCAACAGTTACAGTGATATTCATGAGGCCACATTTTCTAAGAAG
TTTGAGTAGAAAAACTATAACGCAAGATGCTACTGGTCTATAAAAACATTTTTTTTTTCTAAATTGCTTGATTTACTATTA
ATGTTTTCTTCATTGGCTATTAAAACAGCACCTTGATGTAAAATAGTCTGGCGTAGGCTGGCTAACTTTTTAAGCTTAAAA
TGTGTCTAAGATTAGAGATGCACCAATTAACTGGGCTCTTTTTGTTGGAAAATATGTCAACATAAATATCTTGACCTTGAG
TTCTGTCAGAAAAAAATAGAGCATAAATTAGGCAGAACAAACGGAAATGTTTTCACTGCATCATCCATCACAGATGCCCAGT
CCCTCCCACAGTTTCTCTCTAGCTTCACAAATACTCCAGATGGCGCAGTCAACCTTGGTGGGAGTCAATATACAGTAGGGC
TGCTTGTATTTTACTGTATATCATTGGTCAAAGCTAAGCAGGCACTGAGGGCCAAGGGGCTAAGACATCTAACAGACATCT
TCATCTGATATGCATTTGGATATTTCTGTCATGTATCAGGGTTTACAATTTTGTTTTTTGGCTTGTATCATGGCTTATAGC
AATGACGGGAAACGGGATAAGAGAGAGGGGGGATGACACGCAGCAAAGAGCCACAGGTTGGACCTGAACCCTGGGCTGCTG
CTGCGAGGACACAGCCACCGCACATGGGTTGCACGCTCTACCAACTGAGGTACTGGGCTGTCCCTAATATCAGCTACTTCT
TAGTGCACATTTTCTCAGTTTAGTGACTACATAAATATTAACCGACACAGCAAGACTTAGTGGATTACCACTGGTAAACCA
AACCAGCAATTTCAAATAATATCACATTTTTTAATCAGCATCAGCCCTGAAAGTTTGAATGGATCGTGTCTGGTCTTAAAA
ACAGCCTAATTTACAGTATCTTTGTCTAAAATGGCTCTGGTGTTGACAAGAATATGCGCTTCCATACAGTAATTATGATAC
TGACATAATCTCTCTGCAGAATAGATCTGAAATTGAGATTTATACTTACTTTGTATCTGTAGGGTCCAATTCCATAGTCTC
CAACTTTGACTGTTAGGTCTGCGGTCAGGTAGCAGTTCCTCAGAGCCAAATCACTACAAGAGCACAGAAAGAGACATCAGC
ATGACACATGAGGACAGTTTAGAGTGTGTACTTGTACACAAATGTACAAGTACCAAAATATTCACTAATTATGAAACTTGA
AAGTTTATGATCATCGATCGAAGGAAAATATTCAGCTGATGCTTGTTTCTGCCCCAGGAAATTCATTTTAGGCAATAACCA
TCATTAATAAATGCTACATTTATATTTAATTAAGCTTTAGTTAATAGCTATTTCACTGTTAACAAACAAGGAAAAGCTTTA
TTATCCATTTATTAAACAATTGGAAAGGTTTATTAATAGTTGCAACTTGAAAAAAACAAACAAGATATTAAGATCAACAAT
GACTCCAACATCTGCTCTTAACCTTAAAGGCTCAGATTACCTCACAACAATCAAGGGCAAAGGACTCTACCAAGGACGGAC
AAATGACTGGCAACTATAGGAGCTTCTTCGTCTGTCATTGGCCTCGGCTCTTCACCTTGCAACAAGACTCACACAGGAGCT
ATTCCTGACTGGTGCATACAGCAGCAGGATATAGTTTCTAGAGGAAAACACAGGCTCTGAAAACTGTTTGTGGGGCTGCCA
GTAGAGGGCGCTAAGACGCTCAGAGGCTCCCTCTGCACATTAAACAGACATATGGTAGTTCATTCACACTGAATGAAGAGG
CAGGCTTTGGTTTTCCCTTTAGCCCTGCGGCAATCCCTGTGCACTGGTTCCAAACGAATGACACTGCTGACACCAGTGCTC
TTGTGTGTGTGTGGCCCAGCTACCGTCAGAGGCACTTAAACCTCTGGAAGACGAATGGAGGTTTATTTTAAACTATCCTAT
GCTGCTGGCTGAGTGCCTAACATACAGTAACACAGCAATATGAAATGGGTCAAACATCTTAACCATACAAGGCTAATACAT
GGCATGGCAATGATTGCTCCTGTACCACCAGACACCAGAACAGTCTGGTGTCATTTCTGTTGGCAGTTCAGTTTATTGGTT
TTATTTGTGTGATCAAGAGGAAAAGAGAAAAACCCTGTCACATTATGTTCAATACCAAGAAATTACTGTTAATAATGATCA
TCTTTCAAAATAAAAAAAAATAAAAATTAGATTTTCACCAAGTTGAAACATTTGAAATACAATAATAGATATTAAACTATT
AATATTTTTTTCAATTTCTGCCAATAGATCCTCCTAAATCCTACCTGCTGGACCTTTAAAGGGGAAACTAAGCTGTGACTT
AGGCTTAATTAATGTTTGTGTAATTGAGAAATTGGATTCGACTACGTATCAGCGAAATTTAAGTTTTTGAAAACTGGTGAA
AAGTTCTGGGTAGCAGATCAACCACTTAATACCCACAAACTTGGCTCATTACTTGACAGAAAGTCTCAAACTTGCACTGAA
AAAAAAAAAAAAAACGTAGAGCAATCACTGAACCCTGCCTTTTAAGAAACTTCTTGCACATTTTCTTAATGAGAATATATT
CGTGGCACACAGACAGCACAGTATGTGTTTTTCTCTTTTGCAGCACCATTCTCAGCCTTGCATTGCAAAGCTGAGGTTTCA
GCTTTGGATATCAGCCCACACAGCACTCGCCCCAAGGGTTTTGTGTCTGCAGTGCTGCACTATCAACATACATTCAATTAT
GCCAGCATGTGGCTGTGCTCTGAATCAGTATCTGTCAAGCTTTATTTAAATTTTGTGGCCATAAAACAAGAGTGCCACAGA
AATATTATATATATATATAATTGTAAATGAAATTGGTCCACATTGGAGTGAAGCTTGTAGAACTCCCTTTAAGTTGTAACA

AGGATTAAAACACAACAACGAATAGGAGGCTATCCTTTTACACACAAAGAGCAGAATCATCATATCCTTCTGGCAGCGACA
GCTGACACTGTGGCAATGCATGGGAGCTGTGTAATTGGAACTCTCTGAGCTTATTCTGAGCATGCAAAGTAGAGCTGATGA
GCGGAGCATCGTATGTTACCCCCGAGCCACAAGTTCCCAACACCCCCTGTACCCTACTGGCTGCTAGTATCCAGACATCCA
GTGCTTGCTGGGGGTAAAAATGAAAATGAAACGCGGCCTCTCTGCTGTACACACCTTTTTTGGAGAACACTGGTGCTCCTG
AGATTGGCTGGACAGTGAGCACTCAAGTAAAACTAACAGTTGACCGAAGTCTTTACAATCATAATTTATGATGTTTGTGGC
TATATATTTACAAACTGACATTACATAAACAGAGATTACTTCATATTAAATCACATGCCATAAAAAAGTGTATTAGTATGC
CTTGACTTTAACATTTAGGACACCAACATTTATATGTACTTTGTTTTATGTGAAAATTGTTAAACATATGACATTTCCACC
AAATCTTTAATTTAAATGTCATCTAATTTAAATCCTTAGAACACCATAATGATATCAGTTATGTTGCACTACACAAGAGTA
GCACACGTTTAGGAGAGAAATGTCAAAAGATTTAATTAATAAACATCCTTTAGAATTAAATATAAATACAGCTAAGAGTTT
CAAAAGAACCACGTCTTCATTAAGTAATACCTATGTATTTATTTCAAAAGAACAACTAATCTAATATATTAAACTGAGGCA
GAAAAGTACCAAATGTTGGCTCAGGTTGTAATTAAAATACGTCATTGAAGTGTTGTTGTGTCTAGCCGTCACGGAGAGTGA
GTGGTACCCTAAACAACAGCTGCCAGATTAAGTGTTATGAGACTCACAGTGCTTAGCTCACTGGGTGTAAAATCTGTGAGC
TTGCTCAAGTGTTGCAGTTCTGAAGTGCTACCTGTGCAGGAAGTTGTGTTTGTGAAGGTGAGTGACACCAGCAGCGATTTC
ACAGGCCATCCTCTGCAGCTGCAACAACTCAGCGTTTCTGAACATCCAATCCTGCTGAGACAGATAGCCCCGCAGATCTCC
CTGCAAACACACACACAGACACACAGCAACATCAGTTCATCAGTGTCAAACATAAAAAAATCAATAGATCTAGAAGAATCT
TATTTGTATTTCAGAAATATGAGAAATTTAACAAGTGGTTGTAGTTAAATGCACTGTGCAGAGACCGCCACACAGGGGCAG
CAGACTCTCCTGTTACCGTCTCCTCAGCACTTCTCACTGCTGATGTGTGAGAGGACCAAATAAGGGTAACACGGTTTAATG
GACATCTATGCTGTGTCACAGAGATTTATAAATAAAAGAGTGCGGCTCAGGTCAGGTGAAATACGTTGGGTGCTCCAATTG
GGTTTTCGGGAGCTCACGAGTGGAGGGGAGAAGAGAACAAGGTGTCCCGTGAGGAGAGTAGGACATGATACTTTGAGTTAA
TTAATCCCACACCAACATGATAAACAAGCATGTGTGGTTAGAGAGAGAGTGAAATTAAGGCTTATGTTACCAGTTTGACTG
ATCACAGTGCCATGAGAGGTTAATATACGACAGTGAATCTAGAACAATAAGTTGCTTGTCATCTTGGTTTTCATAGGGTAG
CTACACCCTCATGCATACCCTGCTTTAAATTTCATTTTGCCCTAAATAGGACCATAACTTACAGAATGACGATCAAGCTGT
ATTTAGAGTGATTGAGACCATAAACTCATGAGGAAACTATATACTGAAGTAATAAATCAAGTGAGAAGTAGGGCCATTTTC
TCATAGACGTATACTTTTTGCAACCAGTTAAGGTGCCCCTTGCTTTCCATTAGCAAGACTGCAGGTTTAAGGTACTTCTGA
ATGGCTTCAATTTCAGACCGAGTGCAAGTCCACTTCCTTTAGACGTAATAAAGCTGGAAATGTCTGTGGACACCTGAAGTT
ATTCAATAACATGTACCACCAAACTTGTCAATATGGTATATTGTACTTTGAGTCTTTCTGATATTTCACTACAATGGGTGC
ATTAAAAAACAAAATCAAAGGGCAGTCTGAACATGCTTGTCTACATTTTGAACACTATATTTATCTCTGACTTCCAAAATG
TGAAAGCATTTGAAATCATGTTTACAAAAGAGTAAGGCGAAGGAGATTCCTGATGAGATGAACGGTGCTCCCCTGAGGAAT
GAGCAACCAAAACCATGCTGTACACATCAGAGTCACATAATATTTGAGGCGCTTTTGTTAAAAAATGCCTGCAAGCCGTTA
CTGAGCGTGCAAGTTTGTGTTTGTGCGTCTGTGTGTTTACAAAGGTTTAACTTTTCCCTGAGCTATCTCTCTACAAGCAGC
TTAGATCGGTCATCTTACGTAAGCAACATTGCTTGCTTCAACCACATTCACCCCTGCATACACACACACACACACACACAC
AGAAACAGTATCACAGAAAAACACACACAGCTCCGAGAAGCAGTCTTCAGAGAAGAGCTATATTAAATGTATGGTAAGCGA
TACCCTCTCTGCCTGTCTTCATTTGTGAAGTCTCTGCAGCAGGTGATTTGGTCTGGGTTTCAAGGGGAGGATTCAGCGCTG
TTAATGAAACAACATTAACTGTGAAAGGCCAAAACAATTAGACCTCGTGTCTGACCTGTTAATAAGCAAGATTCATGTCTG
ACTCAACCCCCACATGATCGAGGTATGTAAATAAGTGGATACCAGAAGCAGGCTGTTGTTCTGATTACTGTTAGCTTATTT
GCTTGTAAGCTAAAGAGAGCAAGTGGGCCGTTGAAAGAAATGGAAAATCCAGGATAAAGCACTTAAAAAGGTGCAATTTAC
CCAAAATGGTTTGTTACAGTGTAACCAACTTAGCCTAAAGAAGTCAAGAAAGACACCAACCAGATAAGGACAACTTTACCC
ACAAGGCAAGTTATTATAATGCTCAATATGCCTACTGGCAGAGCATGGTCACTTAAGTATAACTTAATAAATGCCATGACA
GTAAGAGCGCTTCCATTACACCATGAGGATTGCTGCTTTAACGAGCATAATAAATGAAGGAGATCAACGTCTTACAAAAGC
CAAAAGTCAGCTGGCTCATGTCTGAGACCCTGAACCAGAGAAGGGTCTGAAAAACGTCCTGGCCAGGCACCAGGAAGGAGA
TCCATGCATTAGAAGCTGTCACTCACACTATGGTATGTGACATTCTGTGGCCCCCGCACCAGTTGCTTGATGTATTCTGAT
GTCTAAAATGCTTTGAGTCAGGGTTACCCTGCACGGGTGATACAGGACAGAAAAACCACCATCAATCATCAGGCTGTCCCT
GTACGTTGGCTTTTTCGGGATTCATCTTGTCCACTGCGCAGCTGCAGTGTCATATTTCATGTTTTGGTATAAAGGGGGATG
ATTGAAACAGCCTGTAGTATTAAGAATGGTCTTTAACTTTTTTGTCTCATGTGAAGCAAGTATTATTTTAAGTTTTATGTG
GCACAAATGGAGCATGTGGAGTGGAGAAACACACATTGTGTAAGACAATACCCCACTTGGATGAAGACCTCAAAACTCTTT
ATCCACAGAGGGATAACGAGCATAGACATCACTCAGTTGTGCTACCCTCGCACATTATCACCTTCAACAAACAGGACCAGG
TCCTCATCACGCTATCTTCACTAATGAATCACACACACTTGTGATAAGTCATTCACCTTGACTCATTTCCATCCCCTGTCA
GAGACGATACAAGAGCCGACTGGGAAAGACCACAATGCCAGGACATGGGCCAGGCCTGGGCAGCTGACAATACACCGGCCC
AACGAAGTTACGCAATGTCTGGCAACATGAGCCAAACCGGACCCTGCTGGAAGCGTTACCAGGTCGTAGAAGTGGGAAGGC
AAGGTGGGGGTGGAGTTACTCAAAGTGTATGTGTCACTTAAGCAGCAGAAATGCATTTCCTTCTTCCTGCAAGCTAATAAA
ATGTGGCTCAACATCCAAGGAGCAGAAATGAAAATGACGCTGCAGTCTGCACATTTGCACAAAGGCAACTGATACATCACA
TAAAACAGATTCTAATCCACTTCTAAAAATACTGTGAAGTCAAACTTTATGGCTCAGGTTGGGCCAACTCATTCATCGTTA
CCACAAATAAATTACATTCTGAATCCTGGATACATGAAGCAGCTAATGGCTGGAACTGTGCATCTCTTATGCACCCTGACA
CACATGGGTCTAAATCATTCAGACAGACACACACACACAAGAGTTATCACTGCCCAAGTAGGGCAAGGGTCATCTGATTCA
ACAGTACAGTCACGTAGAGATGACAAGTAAAGCCTGCACACTGGCTGACTGGTTGTTGACATTAGTGTTTTAATTAACTCT

GGTTATTTTTGAGGCTGTAGTTACTAGTGCTGGTTGTTTTTCATTTTAAATGTTATACTTGGAGGCTGTTTGTCAGACTAA
TATTAAGACATAAGTTATAATGAGAAAGTGTAATTTAATAGAGTAAATGATGAGTCAATTTAAAAATAAAAACATATTATA
TATTTAACCTGTAATACCTATTTTTTGGTCACCTTGGGGCAGTAAAAACAAGGTGTTAACAAGGTTTTAAGTTGATATGGC
AAAGTTGGTGAATGCAAGTCTTAAATTCACTCTCTTTTAACTCTGTTTTGGTTTTTTACCAACACTGTAGGGTTTTATCTG
GATCTTCATTTGCTCACTGTGGCCAAAAATCACAACATCAGAAGGGTGAGAGTGAATCAAAACAGAAAAGTGACGGACTGG
ACAGCTAAACAATGAGCACGAAATTATAACAAATCTTTTGTTAACCTAAAAAAAAAAACCTCTGTTTTCATCCTGTTAAGA
GTCATGTTAAGGATATAATGGCTATAATGATTACGTATTACTGTGATATTTTTTGGAGATGGTCAACGCACCTTGAAATCT
CATTGTTGCTGTTGTGGGATTATATCATATTTGAAGGTGTTTCTAATATTTTTGCTCCCACTTTTTTGACAATGCGATCAC
AGAATTGGAAACACTTTTCAATATAATTTTATCAAACAAAACATAGTGTAGCCTACAACTATAGCCGAAAATATTGAATCA
GCATCTCTACCTTCTTACAATCAGTTGGACTGCATTATAATGTGCAGGGTTTCCTATTTCCTTGATTACTAAATCAAACAG
AACCTAAGTCGGAGTATATTTTTAGCTGCTTATTGCATTTGTCACCCCATAGGTTTCTTTGGAACAGCTTAGTTTACTTTA
GACTGAGCTCAACAGTCTCCTACATACACAAGGTGCAACCCATAAAAATGCACAAACACAGCAACTGGTGGAGCTGACACC
AGAGGAGCGTAAATGGAGCCTGTTGCTTTACTGGGTGAGGTGGGAATGTGAGAAGTCCCAAGCTTAGACTAGCTATGCGAC
TTCAGGCAGGGGGAGGGGAGCAGAGGAGGGAGGGGAGGGGAGGGAGCTCCCAGCAGTCCGGGCCCGGCCCACCCAGGACAG
ATAGAGAAACAGACAATAAATCAGCTACTGTGATGGAGCCTGAGTCACTTCAAAATGTGAAGAAGAACAGAAAGGGCTAAT
TTTGGATTGAGATAGTAGGCTTTCTGTCCAGATGGTCAGCAGGGCCGGTCAGCTGTTCATGCTTGCTGGACAGTATTTAAA
GGGGTCTTGCCCTCTGAATTTGTGAGCTTAACAGACTGACGAGAGCTAATTCTCAGATTTCTCAATTAATGAAGCCTTCTA
AGTGAACGAGGACCGAGATGGCAAGCAGTAAACAGACGATGCCTCAGTCCCTCCACACTTGCACACAAAAACTGTATGTTA
ACTGGCACAATAATGCTAAATAAGAGTACACAGTGCAGATTCAAAATACATTTTTTCATTCAATATTCATTATAATGTTGT
TCATTTTGGCTGCAATTAAAAGATTCTTTAACAATCAACATTTCTTTGAGCATTAATTTGTGCCTATAACTGACACTCAGT
AGAGCAGGTGGCCACTCACCATTTGACAGTACTCAAAGACAAGCAAGAAGGGAATGGCCTCAACACACTGTCCCAGGCATT
GGAGGATGTTTGGGTGCTGCAGCACTCTGTAAACAAAGCTGATGTTAATAAGAACTGCAATAAGCTTTTTAAATAATTCCC
TCAAAACAATGTTGGTACAATTTGAGAGGAGATTAGGTCTAATGCAGTGTGACAGAGGAAAGGTAGAGGGTGCTGCTAACA
TTACTGTGATGTCAGCTATTTCTGTCCACATGCACTGTACGCATGTCCTTCCAAACACATAATCAAAACACTACCATTACT
CAATCGAATATTAAATATCTGAATTGCAGATTAAAAACATCTGAAAAACAATAATAGTATCCGGTGAACAGAGTTACAGTG
TTTACCTGTATGGATCCCCTTGCTGCAGGAAATCATTCTGCTCCTTGGCACTTGCATTAGCTTTTAACTCCTTCACCACCA
CTCTGGCTCCACCTGGATCTGTGTAGATTTCACTCAGAAGGACCTGTTAATGCATACAAACCTTTTAGACTTGACAGGATA
CAGAGAAATGGACCAGGTTAAAAGAGGTCCATTATTCCCAACAACTGTAGAGAAAATCACTGACAAAAGGCAAGAGACCTT
GAGATGTTGCAGAATAAGCAGCAAAAATTCAAACACTACGCTAACTTACAATTTGTATTTTTAAATTTTTTGGAAAAGTGG
CAAACTTGTACATTAAAGGAAACAAATCTCACCTGGCCAAACCAGCCATTGCCGATCTCTTGGATGTAACTCAGGCTGTGA
CGTGCTACCTGGAAGCCTGTGGATCCCTCTGTGAACCAAACCACAAAACAATGTTTACATAGACTGTACTAAAAATAATAA
TTTCACTTTGTGCACTTGTTGCTCCTAAGTGTGGTAATTTCATCACAGTACTTGTGGGCAGCAAGTACACAACGTGATACA
TCATACTGAGTAGTTACAAAGTAGTCCCTCATTACTTAATGATAAGGACACGTAGCTGATTTCTTAAAACAAAACTAAGTA
GATACTTGTCCTAATCATGATGAGGGATCAATGAGGGATTACTTGGTAACTACTCAGTATGATCTATGATGATCACTGTAC
CTGACAGGGCACACAACATAGTAACCAATGATTAGGTAATTAGAAGTAATGAATTGTCACTAATTTGTGCCACTCGGCAGC
TGGTTTAGAGTTAATCGGTAGCAGCTTGTGAAGACAGTGGTCAGTAGGTTGATTCACCAATATTTATGAATCATGACCTTA
ATAATAAATATTATAGTATTTCCCAGTCTGTGCTCTAGTAACTCATCATTATCGACTGCCAATCACAAAAAAACACACCAA
TCTTCCATATATTTTCATAGATCTGTATGTTTACAATTAATTTCTTTTTAAACTAAAAGTAATAACAAAAAAAGGTGCTGC
AAAAAATCTAGTAAGTCAAAAACATTATAAATGTGTCATTTGTATGTGTATGTCCAGTATAGTATGTAATATGTAGAGTAA
AAGTGCATGTTTAGACACACTATAAATCTGTTTGTCTGTGGGTGTGCATGAGTCTGACCTGTGATGCGGGCAGGAGGTTGG
AGGTGTGGGGGTCCAGGCAGAGCCACAGGGGACACAGCGAGGGTGTACACCTCAGCTGGGGACTGCATAGAGGGAGTGTCC
TCTGCCGGTGGAGTGAAGTCGATCTCATCATCAAAATGGTCCTCGAACTCCTAAATCAACAACAAAAATCAAGAGTGGATG
TAACCTGGCTTGTTTCACAACAGAAACCGAATTCACAGACACTGTCCATTTTGCTCCGACCCCACTCTGTATTCATTAGGT
GTGTCCCACATGGGCCCAGGTGGAGCTATGTCTGTCTGGCTAGCTGCCTGCTGGGACTCTCACCTTGAAGTTGATCTCCCT
CTCCTTGCAGCAGGTCACACAGTTCACTAGCAACACCACCAAGGCCACGAGCGCCGTCAGTGACACCACCAGGGACAGGTA
GAGAGACACAGACAATGTGTCGCCTGTGCTTCCACCTGAAATGAGAAAAACGGACAGAAACCATGCTGAATCATTTATAGT
TGTGCTTCAAAATTAATTTGGAGGAGAATGAAAGAAGGCAGCTGACAGCCTTGTCATAAGCATTAAACATAGGGTGTGTTT
GACAGTAATACAGCACACTTGGGTTGCACTGGCTGTTCTACTAAATCCACCCACAACAAACGCTTAACAATAAAAATAAAC
TGCAGTGCTCTTAAAATGCAACTTCTTGAGCTTCCTGCTCTCTTTCACCAGCAACACCTCTGCAGAGGGAAGAAACACAAA
GCTGTAATACTGTGAAAGACAGTAATTTTGCTATGAGGAGTCAAAACTGTGATTTTTTTAGGTTTACAATTTACACAAGCA
TGACATGACTCTTCAGGCCAAACAATTTCAACCCCTAATCAGCTATTCAGTATTCAGACAGCTGTGGGGGTTTCCACTGGC
TGATAAGCAGCTGATGGTGCAGCAGCGGTGACAGAAGGTGTGGGCCCGCTCCAGTGAGGATGCTGCTGTCACAGCATTTCA
AGTGTCAGGGCTGACGGTAGCCTTACAACCCCCGCCCACTGTGCTGCTTAGTTGCTGCTCTCACTACACTGATGCTGACCA
ACACACAAACACACACAGGTTTACACTGACTTCCATTCATTGAGCAGCATAACAGCATTAACACGGACCCTTAGCCTAATC
TTAACCAGGACCGTAGAAATCATGTTTTGCAGCATGAGGAGCAGTCTTTGGTCCTTATGAGGACTACTTGTCCTTACATGG

TCAGTGTTTAGGGATGCAAATTTGTAGCAATTTCTTTAACCGATAGTCAGCTGCCTTAACAATCAATGATCGGTTCATCAT
TAATATATGAAATATGCTGGGCATGCTTCCATTAGAAAACTGATAGAAAGTCAGAGTCGGTTACATTAGGAGGTAAAAGAA
CAGATAAACACAAAGTTACGTGAACAATACACTAACACAAAATAAAGACTTCATTTCAATTGCTGGGTGCCGTCAACAACT
GGCACAACAAAATCCAACCCATATTGAGGTTACTTAGTGGCTTAAATATAATAATCAGTTTGTATAGTACTTTTTAAACTA
AAAATTACTTGTATTTTAATCAAATATATTTTCTCATTCAAGTATTTTGTATTCCATTTTGATATATTTGATAAGTAAGTA
TTTGTAATAAATTAGTAAATTAATAATCTAAATAGCCATGATTGTTGGTTAAATTCTTAACAGAGACTAATCAATTAATGA
TTAATTATTAACATTCTTATCAGTGTTTATACCCGGAAAGGTCCTTAATAGGTAACAAAAGTGCATACACACACAAACAGC
TGAAGCTTTTCTTTCTAGTGCAACAGAGACTGCTTCACACACAACACACATTAACTAGTCGAGCTTCTGTAATGTATTTTT
GTACTAAAAAAAGATAATGTGCTACCACAAGAATGGTTAGGGTTAGGGTTAAAAGTTAAATAAAAGAAGGCTGCAGTAGGC
TGCAAAACTGTACACATTACAGCTTTTATTTTGAAATAAGTGATATATCAAATTAAATTCATCTGTATTATTTATAACCAA
AGGATGCTACACAGGCAGAATTGGTAAGTGTTAGCCATAGCCTTGTTTCTAAAAGTAAATATCTAAAAAATGCCTTCAACC
TCTTGTGAGCCATCGCTTGGGCTGGGAAATATGGCCCCAAACAATATTTTGATACACGATATATATACAGCAGTGTTTTTC
AATCCTTTCGCAGGGGCCCTCTGCCCTGCATGTTTAAGATGTTTCCCTGCTCCAGCACACCTGATTCAAATAATCAGCTCG
TCATCAAAGTCCACTAAAGCCTGATAACATCCACATGCATTTGAATCTGGTGTGTTGGAGCAGGACTAGAAATCACTGATA
TGCATTATCTCACAATTTTTTAATCTCCTCAAAAGCAGTTCATTAATGTTAGGACCGAGCAGCAAAATCGATCAACACATT
ATTTTTGACCAAATACCTCAATATCTGTGCTGCAACAATATTGTAGGGGCTTTGAGCGCTTTTTAGAAAATAATAACACAA
TGAGATTTTTGATAAATAATCATCAGTAATGTACTTCACTTAGTGCTATCACATTGTAATAAATGGTAACATTATCTCAGC
CCAATGCATCTTGACATGGCGCAGTGTGTGTTTGTGCGCGAGTGTGAGTTTGTGTGTACTCAAAGCCGAATGAGACTTTGT
GGGAGACTCTCCTCCCCGTTCTCCCCGCTGTGTTTATTCTGTCTCCCACTGGTCGCAGCTTGTCAGGTACATCCTGTCTGC
TCGAGGGGGAATCAGCACCTCCTGAGTCTCCTTCTGCTCAACTACAAATTCCCAACAGATGAGAGCGGGTGCTGAATACCC
CCAGACGACAAACCTACCTCTAGTTGTCCCATGTTTTCACACTTTTACATGGGGGGGGAAAAGAACAGACTTTGCGTATGG
CTGCAACACAAACAGTCTGAGCTCTTTAAATTAGAAATCAAAGTAAGGCCAATCAGTCTACGCTTAGCCACTAAATATGAA
AACATAACTTTTCATCTCCATTTTGACACGTTTTTCATTATATAAACTCATCCGGATGGGAACAATCTAAAAATGGTGGCA
CCTTGTAGTGTAAGCTCATCCAAATGATGCCATATGTTTGCTTGGACACTTGCTTGGTTCTGCAATGATCAGCAAGAATCT
AATTTTCTGTTGTTCTCAAGTCTGTTTGGATAGTTTATCCCACAATAGAGGTGCTATCTTTAATCTGCATTTATGCTTAGG
TTGCAGGGTTTTTCACAGACTTTTTTTGAGACAAAGGTAGCAATGGTTTGTGAGTGCCTTGTGATTAAGGGGGAGAAAAAA
AACAAAAAAACAATTACATGTCTTGCTTCCCAACCAGAGTTGAGGGAAAACAAAGACTAGGGAAAGAAAGGGGCAAAAGGG
CACAGAGCCTGATGAAAAAAGTGAAGACAAGAATGATGAAGGAAGACAGCTTTTCAATTTGACCCGTGCAGTGAGCAGATA
CCACTGGGTGGGCCACATGACATGGAAGCGATAAAGGTGAGCTAACCATCCTCTCACAACCAACACTGAATAAAACTGGGT
AAGAAGATGCCGCTGGATCAGGACTGAATGTCTGTTGAATATCTGACCAAACGTTAAATGCACCGCTGTGAGTTTCTGCTT
TCTAAAATGATCTGTCAAGTACCAGCTAATGAGGTTCGGTATCCAGTCCTATTTACTTCGGATTAATGGTAATTCATTGCT
ACCTTGACCTTACGTGTAACATGCCAACGCTTGGTACAGGCACATCAGAGCAGTTGCTGATAAAAGGCAGTTTCAGCATTA
AAAAAAAACAAAAACCTTTGTGCTTTTGTCCGAATATTTAACAAGGTGGCGGTCAATAATAGAAAGGTGGTCTGCCTTTTA
ATTGGATAGGCAGGGAAATCCTAAGGCGATAGAGGATTTGACAAGCATTAAATTATGAAATGATCAATGCTCTTAGTGCAG
CAGCATTGGAAAAATTGTTATCCTGCGCTACCCTTTGATGAATTTATTTTACACACATGCAGATATCAACTGAATGTCAAT
GAGAACATCTAAACTGTGCACGCTGCCTTATTAAACTGCTGACACCAAGCAAAGTAACTGGGATGTGTCTGTCAAACTCTT
ACTCTGTCAAAAGAAACAGGGGAAAGATGAACAGATTTAGCTATCGCTACTGTGCTGGCATTTCAGTGTGGACAGATCTCC
AAAAAAACAAAAAACTATTGTGGATGTATGTTGTTTTTACACATAAACAATGCTTTATCATATATTCACACTGGCAAAGCC
TACACCAACAACCATGCCATTGTTATAGATGACAGGCCAAGCCTCAGCTTCAGTACCAAAGCTCTTTTGCATAAGTGATTT
AATTAATCTGACATCAAGTCCTGCCTTCCTCTAAATTATAAATAAAATATTCTCTTGACATGAACCTGAGATTAGAGCTTG
TAGAGGCTGAAAGATAGTCTGACAACAGGCTGAGCAGTGATTTTACTATAAGTCAGGTCTGGTCAGATGGAGCAGTTGTGT
ATTCAGACAGCACAGCCTGTCAGGGCGCAGATGAGATGAGCAGCAATCACTTTATCTCCCTGGGGAGCATTAGATATGATC
CAAAGTGTCAGCCATGACCACAGAACACCAAGCTATCACTGACAAAAATAGACAGAGTGGCTGAATATGGTCTCTTGCATC
CTGACTTTTTACAATTGTCTTGAGTGCCGTTAGTTTTCCTCCCCGCACAGAAAACATACTGAACCTCGAATCCAAAACGGA
CATATCAAACAGAGTTCTGTTCCCCTTACCTCTGAGGAAACGTATACTTTGGGGAAAGTAATTAGAGAGATAATGTGATGC
TAACTGTAAAGGTTTGAAATACATGAAACACGGATATTTTACTGATTACTAAAAGATGGCTGTTCTCCTCCACAAACCTCT
CCTCTGTGCCAAAGATTGCATCGGAGAAACAGCGTATGTGGTGAAATATTGATGAGAAGAGGAATTAAAGATTAAATAGGT
CCATCGTCTGGACACAGACTAAGTACAAGTAGCTCTAAATGCAGATGTGTACTTACATCTGTTAAATTAAAGTGTTTCCTA
CCTTTGCAATTTATCACTGACATCCCGAGGGATGTGATAAGCCTTGTAAAGTCTAATATGGCCTCCTTAAGCTTATACACA
GTTCCTCGGAGGAACAGTAAGGGGAAGAAACAGGAGGGAAACTGCTGCAGCCCAACCTGATAGACTGAATCTGCAATAAAC
AACAGACTACACGAAATGTTTAATTATTTAAGATTGTTGTGAGTCAGACAATACAGTCAGCACTTGTCATGTCATATTCTC
AAGGTGGGAGACACTACAACAGGTGACACATCCACAGGATGAGAAGAGGGAAAAAAATACAAACTAGGACCCGTAGTTCCC
TTAACTGACTGACTTCCTGCACAAAGTGTCACCTGCCATTTTTACCTCACAAAAATGGGCATTAAAACAATTTATGTTAAA
GAAATTTAAACTAATTGAATTATTTTTCAATAGGAACATCTGGTGGGCCAATATAAATATTATGGCAGGCCGAATTTGGCA
TGTTTGGCGTGCATGGGTGTTCAAATGTCAAAAACATGGTGGTGGTCCCAGACACACCATTGGCCGGGCTTTCCATGTATT

AGCTTAACACAAAGTATTAGTAGTGAGTTGTTAGTTGAGCTGGTGAGGTACAGAGCTACATGTACCCTACATGTTAGGTAA
ACATCAAACCTCCATAAAAATGTAAAACATAAAACAATATGTACCGTATGCATCACTGTACTGATGCCAGATAATACCAAC
TGGCTTTAATAAAGCCAAAGCGTAACTTAACAACAATCCTGTGAAAAACATTTGCTGTTTATAGTAGGCAGCTTTTTTTAA
TATAAACACCTCCCTTCTGTTTCAGCGGAGCAGCGAGGTTCGAGGCGGTCCAGGACTCCCAGAGAGGCTGCAGTCGCAGTC
AGATAGGAAAAAACAGGAGCTGGGTCACGGCAGCTCTACATGGGATCAGTTCTCCTCCGTTAGGCCTTATCTGAGTCATGG
GTTCATATTATGACTTGGCAGGCACCACCAAATGACTCAACTGGTTTTTGGGACCAATACGGCAGGGAGCTGCCAGGTCTT
ATTTCGGTTCCACTGCATATTTCAGCAGGCATTCACTCAGAGGGGGAGCTGACCCATTTTTGCAGATTTCACCCACCAGCG
TGCACAAGAGTGGATGTCTGCGCTTTCAGTTTCATGCAGGATGAAGGATGGCATTGTCTGGCTGACATGTCTGGGTTGAGC
TTAGATTTGTCTGTCTGCTAGACTGAGACTCATCGATCAAATGATGAGCCCAGGGAATTGAAGCAGCCAATATTTATGGCT
GCCTTTGTCAGGATGCGTATTTGCAAGTTAGCATTCTGTCAATTTCAATTATAAACTGTCTATTAACGTACTGTCAGAATG
CAAAAAACATTGATTGGGGCAACATAAAGGAGAGGCTGGTGTTAAAGCACCATCCAGTACATCCTGGTGTGCTAGTCAATG
AATCACTGAGCGATGTAGTGGAGGGGCTAAGGTCAATGTCCACCTGGTCTTACAATCCACACATGACTCTATCACCTGGCT
GTTACTGTAGTTAAAACTTCCGTTAAAGCCCAACCTTTACCCATGTTTGATGTGTGTAAACAGAGCACATGTCACATGAAG
ACCGGCTTTGCAACTGACCTTAGTTATTATTTTAACTGTGATCTGACTCACAAGCCATAACCGAAATCAAAACTTTATACA
ATGAGGTGTTGCCGTACTGTATCTGTACGCAGTGGTAAACGAAGACTGGGGAAGTGGGGCAAAGAACAGGTTGCTATGTAA
TAAAAGCATTAAACGCTTGCTCATACTGCAACATCACGTCTTGTATGCTGTGTTATAATAAACAAACATCACTGTTGCTAA
TCATTGACTGATGCCGATAAACTAGGAATGTGTTACAGTTACACATTACCGACCCTCGCTACAGCATATTCATGCATGACT
CCATTCTTATATAATATAGCCTATGTTGTACTAGGGCTGCACGATATGGTCAAAAGATCATTTGTGATTATTTTGACAGAT
GTTGCGATGGTGATATGACTCACATTTAATGAGAATGATAATTTTTGCACCCCATTTTTCCTTTTCTCTGAAAAAAACTAT
GAAAATCATGAAGATGTGACATTTCTTGGGGTCTGTACTGAACAAAAATGTTTTCTTACATCTGAGGAACAAAATTTGAAG
GCCAGGGCAGCACGGCAGCACTGCAGCACTTCATGACAATGCTGTTTTGTCACACGTTTTACCTTTATCAAATAATTGCAG
CTTCTTGCAATTTGGACATTGCATTTGGCCATATTGCGATTTTGTGATTACGTTTCAATGAATTGTGCAGCCCTATTTTGT
ACCAAGTGAGGCCTGGAAAAATTGGACATATGGCATCCACAACATTAAGACTGAAACAAATGATTGTTGTTGGACTGGACT
CATCTACATGAACTGATTTATTAATGTTTGAATGGGAAACAGTCAAAATACTTGACATTGAATTCACAATGTTTTGAAATG
AAGGAAAACAAATATTCTTTGAGAAGCTGTAACCACCAATGTTTGGCATTTTTGTACTCAATAAATGTCTTCAATTATTAA
TAATCATGAAAATTGTCCTCACTTGGTTTCCTGTTGATCCACTAGCAATTATATAAATGTAATCTCACATTAATTTTCCCA
ATAAATCTGCTGGGATTAAACTGTCATACAGTCAATTTCCTCCCGTTTCTGTAATTCTGTTTTAAGCAAGCAAGAGCAAGA
GTCTCCTAATCAACATATTAAGTGTTTTTTAATCACACGGGCATATTACAATGTGACACACGATTTTGTTCCATGAAAACA
CTTTCAGCCCAAAATAATCCAATGATTACACCCGGGACAAAACCTGTTATGTAGGAAAGATGCCTGGAAACTAGAATCAAC
ATCAAAAAGGTTATTCAAGGTCCGTCTCGAAACAATTACAGTGTTTCCATGCTGGTAGGCCAACATCCAGTCCATTGTCCC
ATAAACTTTGTTAAATGTCAATGACTGGACCAACCCCACTGAAAGGTTAAATATCTCTGCACCTGCAGTTAAAACAGTCTG
TACTATATTAACTCAGACACATCACAACTTCCTGGAGACTGGTTTAGTTGTGTTAAATATTAATCACAATTAAAATAGACA
AGGTTAATCAGTATTTTCTGAGTTCTGGTAGGGGAATGTAATGTTTGTGTTGATGTTAATTAGCTGAATCGTGACGATGAC
ATGCTGATATTTTCCATTATATTCATTATTAGAGGATTTAACAGGACATATAGACAGACTGTAGAATTGCTATGGTGTAGA
CACAAACCTACAATAGTCAGTACAAATATCCATACAATGAGGAAACGCACTTCAGTAGTGAGAATGAAATGCAGTCAGGTT
GTTGTGTTGTTAGCTTTTAGCTGCTGGTTAGCAACAGCTACCCCGGCAAATATAAGCTAACAGAAACATCTGCTAGCTAAG
CAGCTTGATAGACAAGAGTTGTAGTGAGTGAACAAACAGTCGGTTAGCTGTGTATCAGCTTTCAGTCAGCTGTCTCACCTG
ATTTGTGAGGATACAGGAGAGGGGCACCTTCACACAGACATAACGCCGATAGAAAAATCCCACTGGCCAGCAACAAAACAT
ATCCATGGCGGCTCTTCATCCTCCTAGCTTAGCTAACAAAATGTTAGCTAATTGGAATCGTCGTCCTAAACTGTAAATGGC
TGATTTGAAGCTAAATAACGTGTTTTAATCATTGATAATCTGTTTTACTTCAGTGTAGGCTACATAAACAAGCATATTTTC
TGACTGTTTAGCGCTAGCGACTTGCTGTTAGCCAGCTAACTAGCTTCGGCTAGCATGTCAATGCTAACAGTTTCAGCTGAC
GACAGCAAACACCGCTACGAAATATAGTGTTCCGTTGTCCATAGAGACAAAAGGAGCGATCGCCTTTCACGACCTATCATA
TTTGGTGTGTTTAGCTTAGTGTTATTTAACTGAACATAGCGAAAGTAAATCAATCAATAGCCCTGACTATCATGAAACACC
TCTCCCCCAAAGCGCCATGTTCCTGTACAACGTCACATCACGGAATGAGGCGTAAAACTCCAGATAACTGACAGGCGCCCT
AACCAATCACAACAAGGAATACCTGCACGTTAAACGTACAGAGCCAATCAGCTTCGAGCTTTATCATCAGTCATGTAAATC
CAGTGCTTTGTATGAAATCTGGGGAAAGCTTTTTACATCCAGTCACTTGCCAACCATGTGATGCACGCCTTACAGATTACA
GAGACGACCTGTCTGTGTTCAACATTTATATTTCTGGATTTAAGACCGGCCATAAAATACCCAACAGCATGTAACTGGTGG
CCAGGCTGGTTTTAATGGTGCACTGATGCTGGCTCAAGCATCATGATCTATTTCTTGTGGGTTTTTATTCATTTTTTTTTA
CTGTAGTGATTTCATCTGATTTTTGCTGCTCGGTAGATTTCCGGGAAACTCGATATTCCCCATGATTGAATACTAAAACAA
AATGCAACTCTACTTTGATCACTACAGTTAGAAAAATAGGAAAAACGCTCTTGAACAATCAAGTTGTTGTAGGCTATTTAT
CCCAATTAAAGACAGTCATTACCTCTGCTTGCTTGTGTGTTTTCCTGGCTCTATGTTGTTTTAATGACTTCCAAGCAATAG
CACATGATATTTGATTAATTTTTATGGTGTTATTAAACCTCAGCAAAGAATATATCAACCGATTTAATTATTGTAATACTC
TGGACAAACCCACATATTACCCCTTAGTTATATGAAGCTAAGACTTACAAAATAAGAATCGCATATTCATGTGTTTACACA
GTGTCATTATAATTTTTAAAATGCCTACACATAAGTAATATTAAAATTAAAGGGGCACTATGTAGTTTTGGAGATGAAATT
CAAACTCAGAATTTCAATATTTACAATACTGATGAGGTAATAATACAAACTCAGAAATATCAATTTTTTCCATAAGTGAAT

AAACAAGCTGTTCTCAGGGGAAAATAAGGTCCCCGGAACGCTTTTGAAGCTAGAAAGGTGGCAGGGTCCGCCACATATAAA
CAAAGTAAAAGAGTATGGAATTGTGTTGTCCTTTAAGGTCAGTTTGTTTATTCAGGCATGAAAACAAAGGGTTTGTTTATT
TAGTTTGTTTGGGCATCAAAAGATTAGAGATTCAAATTTCTTCCCCAAAACTACGTAGTACACCTTTAACAACATCACATA
ACTAATTGATAATTATCACATTCATTTAATCTACTGAGAGATTCTGCATTTCCTGTGTACACCTACCTCTTATTCCTTCTT
AGTATCAATATGAACAACACTAATAGCATTAGGAGTTGTTGTTGTTGTTGTTGTTGTTGTTGTTGTTGTTGTTGTTGCAGC
AGGAAAAGCAGAATTAGTTGGATGAATATCGTATGAAAATGTATGTATGGTTATCAGATTGAAATGTCAATATTTTATCTT
TTTTGTATGTTTTTTTTTTCAAAACAATATGAGGGAGACAATTATCTGCTCTGATTAACTGATGAAGGAGGAGGAGGCTTT
CAGTGGCGTGGGGGAAATCTCCCTGGATTTGAGGACAGATACGTCAGGTTGCTTCTTGCACTCCGGATCACGAAGCTGCTG
CGAGAGAGTGTCGTTGGCTCACTGGCACAAAGCAAGAGAGGCAGAGCTCAGCACATCACAGGCTCAGAGAGCGCAAACCTC
CAACCGAAGCACGGTGAGTACCAAGCTCTTTTTTTTTTTCCTTTTTGCAAATTCATGTGCACAGTGATGTTAGTGTGATGA
TAGCAGTTCTGTGCGTGTTTGCACTTTGGAGCATGTCAATTCTTTTGTTTAAAAGAGGGGGGGGGGGGGGGGTACAAAACA
AAACAAAACAAAAGGATGTTCAACCTTTTTTGTGTTCAAGTTGTAATAACTTTCTCTGTGGGGAAGAAAATTCTTTCATTT
TGTGAAACAAACCTTGATCATCATGCACGTGATTTAGTCCATGTCATATGATGAAATATATTTGTTTCTCTCTCTGAAGGA
ACAATTCAAAGTTATATCTTTTGTTAATGTGGTTTAACCTGACTCTGAAAATGGTACAGCCCTCCTGATAAAACGTACAAC
TCAGACTCTGACGTCCCTGAAGACTACCCACAGAGGAATGATCATGCTCAATCTATCTTATCAGACAGAAACTGTTACCTG
ACAGCAGAAAACACCTGCACCTGTTTCATTCACTGTCACTTTGCATTTTTTTCCCGTGCGTGCATGTGTGTTTGTATCCCT
CAGGCAGCTACAGAGGACCATGGCATTTGCAGGGATGCTGAGCGACGAGGACATCAAGGCTGCGGTCCAGGCTTGTCAAAG
TGAGTCAGACGGCACACGCTCACACGCAGGGACGCACATACTTATCTTTAAGACATGAATACATACTTTAGGTGAACATGA
GTCCAAAGACAGAGAGAGTGAGAGGCATTGTTAAACCCACTCCCACTCCCACTCCCACAGGGGATACCTGTGTGATGTGTT
GTGATGCACAGTGGGGACAGGCTCCCTGTCCCCACTGTGAACCAGGCACAGACAGGTAACCAGGCAGCCATGTGGCAGGAG
AGCAGTTACCACCCAGATACCAAAGTGTTTATGTTGAGCATTCACACAGAGAAAAAATCACACGAGCTGTACCACCCTCAC
ATCCCAAAGCACTTAAACATTTATCTCAAATCTGACTGCATGACAAGGCGAACACAGCTCAACCGCACCAATGAATATCAA
ATGTCAGTACTTATACGCCCTTTATTGAAGAAATCCCTCCTTCTGGGAATGTTTCAGGCAAAAAAACATTCAAACTTTGTC
AGTTGTACGTTCATTTTTCGTGCGCATCAGCAAGAGAAAGAAGTATTTTTGTGATTCATTCCAAGAAGTGATAGAAATGAG
TCACCTCGGGGTTGGGGATACATTTTTTAAAGACTCCCCTCGATTTACACAAGATGATTCATTTTTAAATCAGAGTGCAAA
TCTTACACCCGAAAACTCACCCTGGACCAGAATTTCCACTCAGAATGGCTTTATAAATAAAAAGCTTTTAAGATTTTATGA
TTGCCTCAGACCCACAAGAGATATTCAGTCTGCTTTGACTGTTTCAATGGCATAGTAACAAAGCCTATAACAATCTAAGAC
AGTAATGCGCTACAGGGTGTATATTGGTAAATGTCACCGGTTGAAGAATGAAAGTCATCTGAGCGAATGAGTTAATGCGAC
ATCTCTATTCAGCCCCTCGCCATTCTTGCTTTCTCGTCATTCTGCGATCGACCTCTAGCTCCCACATCTGTCTCCTCTCTC
TCAAGTTCTCTCCCCCCCTCTCTCTCTGCATCTGTAGCTCCGGGCACATTCGACTTCAAGTCATTCTTTGCACAAGTGGGG
CTGACCGGCTCCTCTGAGGCAGATGGGAAAAAAGTGTTTACAATTCTGGACCAAGACAGGAGTGGATACATCGAGGAGGAG
GAGCTCAAGTAAGGATAGGGTGTGTGAGTGCATGTGTGTTACATCTATGTCCATTGTGGTGGCCTCAGGCTGAGGTCAGTG
TATGAATGAAGCACATTTCTTCTGTGAATGAACTCAGTGATACATGAGGGGAGGACTGGTGAGGGAGTGATGGAGGGGGGC
GGGTTAAGACAGCTCAGAAAAAAAAAAGCCCTAGAGGGGGAGAAAATGCCATCAAGAAAGATAAAATCCACAATGATACAA
GGTTGTACTTATCGTTGATATTTTTTTGTGCAAACATCAGGCTGTTCCTGCAGAATTTCTCTCCGGGGGCGAGGGAGCTGA
CTGTGGCTGAGACCAAGGCTCTGATGGCTGCAGGCGATAAGGACGGGGATGGCAAGATTGGGCTGGAAGGTGCGTGCATGT
GAAACATTTTCCACGAAAAGCTGTCTGCAGATGAGGAGAGGAGCCATCTGTACAGGGGAGCTGTCCATTACTTAGTGCTGA
AATTGCAGTCTAAAAAAAGCACACACAAAAACACAATGAAAGCACACAGAATATAATCTGTTTTCTGCTTGGTCATCTTGC
TTAACCATAATGTTGTGTGACACCGACTTCTTGCCTGGAGAATGCTGTTGTGCTTAATAGGAATCTGACTGAATCCTCTGT
TTCTGTTTCTCCCCCGCAGAGTTCTGCGATATGCTGAAATAAAGAGAACAAGCACACACGACCACGACTGTCTTTCCCTTT
TTTGAATGGTGGGTTTCTTACGGACATCCTGACTTTCCAGCTGGACCAGAAACCTCAGATAATCTGCACCTGCCTGCTGCT
GCTCTTCCTTGTTGATGTGATACTATAATGAATAAGATGTTTGGTGGACAGCCGGCTGAAGCTGGATATTAGTTGTTGTGT
GTTGATTAAATCAGGTACCTGTAATGGATTAATAAATGTATTTAAAATGTGATGTCACTGTGTGCTGTGTCTTATTTTATT
CTATGGCAGGGTATGGGCTTCAGAGGCTAGTGTGGTGACATGCAAGGTATTCAGGGTTTTAAATTAACTTGATCCTGACTG
CGTGTATACGAAAAGTTAGAGGTGACTGATAATATTATGATTATCAAATGATCCTACATGCAGCATAAAGACAGCAAGCTG
GTTCAGAGAGAAAAGCCGTTGCAGCAATACCTAGACTTGGTGGTTGGCAATGTCGTGTATATGCAAAAATGTGCATATTGT
TTGGATTGACGCAATAAAATGTTGAAATACTGGAGCTCAAATATTGCTGAAAAGGCTGCCGTCTACTTTGATACTGCAGGT
TTTGTCATAGAGTGCGATGAAGACCATTCGTACAATTTACATCATTTGATCTCTTGCTGCCCACAGAAGACATGTTTATGA
TACACAATTTAAAGTGCCAGGTGGCACAACTTCTGTTTGCAGCAGTCAACTCAACTTTGAGTACAAACCAACGTTACCCCC
GCCAAGGAGGTTATGTTCCATGTGTCGTTAGTTGGTTTGTCAGCAGGATTACACAAAAACTACTGAGCGGTTTCCCACGAA
ACTTGATGTCTTAGTGACAACACCTGGAGGTTTAGATCAAGATCTTATCAAGTGTCTGGGCCCAGTTAACATGGAAGACTT
CAAGGATCTGTATAAAAAAGAGCTAAACAAATAAAATAAAAACACAAGATTATTTGTAAATATGTAAATAAAAACAACAAT
TTGTTAAACCTTGGTTATAATATCTGGATATCTCAACAAGTGCATAGATCCCAACCACACCCACCCCCACCTCACCAAAAT
TGTGGGGGGTTTGGTCCCCCATGGTGTTGGCTTCGGGGTTACGGCCCTGATTGGGACGAGTATTGCCTTCTCTTAAACAGA
TTTCAGGGTCGGGCAGAAACGGGTAGAAATATCCACCAAAGTTATTGAAAATATAGAACCAATATGACTCTGAGACGAATG

TCCTTGACGACTGATTAAGGTTTAAACGATGGTTAACAGTCAAGGGGCACATATTTCTAAAGCGGTCACACAGGCCACAAA
AACACATTCATCAGTCACACAAATACGTTGAGAAAAAAAAAGGGGGGAGGTCTCCGGCCTGGTAAATTAGTCACCTGTGAGA
AAAATGAGCTGCGTTTCATGCCATGGAAGCGTAAAATGAGTTGTCAACATATTGCTAACTTGTTCGTACACGCAATCAGAC
TGCACTCAAAAAATAGGCTGAACGATTGGTGCCTACTAAGGTCAGGGATCCGGTGGTATAGGCCTCTTGTCCCTGTTAGGA
CGCTGGGTTGGAGTGAAACTCGACTCACTGCGTGTTGACTTCAGATGAACCTTATGGAGATATACGGTAGCATATACTTTA
GGTAGCTGGTTGATGTGACAGGACAGCAGATTGGCTGAGGCTGCACATATCTATCATCTGAGACCATCTCTAGGAATGTGC
TGCAACTGCTCCACATGCAAGAATGAAAAGCCTGACTCCAATAATTTAAAATGAACTCCTTACAAGCTGAGAAGAATACCA
ACTCTATGGGAAATAACCAGACAATGAAGTAAATTAACTCCTACCACTGACACTGCAGGCAAGAGTATCAAATAAATGATA
TTGGACAGATGCACTGCGGGCGTGTAATGCATATGAATGGGATCATTTGTGAACAACAGCACAAAGTAGGAAGAATCATGG
AAGGAAAGAAAGGAAGGAACTATTAAGCTTTAACTTAATCATAAACTAAAGCTAGAAAGTTTTTGAATCCCTTATAATCTT
TTTGTATTTCTGCATTAATGTGATCTAAAATTTGATCAGATCTTCATTCAAGTCCTAAAACTAGATGAAATAAATTTTTCA
TTAAAAATGTAATGGGATAACAATTGGGTATAAGTTTAGCCGTCCCTCTATCTTTATTATCAAAGTCTTGTCTTTACCACA
CAGGTTTGTGGAGGTGAGCCATGCCTCGATCAAAAGAGATTTCTGCAGAGACAGACAACAAGTTGTTGATAGTCACCAGGC
TGGAAAAGGTTCAAACCCATCTTTTAACAGTCAACAATACACTGTTAGGCAGAGGGTGTACACCACCCACAGAAAGGTGTA
TAATGCTCCTGGAGGGTCACAAAGAACCCCAGGGACACATCTAAGGATCCGAGAGAGTCTACCATCAGACAAACACTGAAC
GAGAACGGTGGGATCGGCAGGATAGAGCATCTAAAGTTTACTAAACACCAAATGGATGAACCAGAAGGCTGCTGGAACAAT
GTTCTGTGGAACGGATGATGACTTTTTTGGGGTTTATTTGTTTGGGGATGCTTTGCTGCCTCAGGACCAGGGCGGCTTGCA
ATCATTGACAGGTCTATGAATTCTGATCGAACCAGCAGATTCTAGAGAACAATGTCAAGGTACCTCTTCACAAAAACACAC
AAGTTACTACCAAAGACGATTAAAGCGGCAGTCCATTCCTAAATTCAACAGAAGTGTTCGGGGAAAACCATGAAGTGAGCA
GCTTTGTAAGGAGGAATAACCACAATCTGATTAATCAACAGTTTAGGAAACACTTTTCCCAAAAAGATAATTAATGTTAGA
TCAATTTTATCAACTTAAGAATGGAAAATGTTCTTTGTGGTATTTGCTCTCCTGGGTCCCCCATGTGTATTTTTTATGACC
AGGATGAACTTCCAATTACATTTAAGGAAAAATGGGTCAAACCTCAAAAGGGTTTCACAAACCTTCAAGGGATAACACAGA
CTCAATAAAAAGCAAGAGTGTTTTCGAGGGCAGCCCTGTCACATATATTCACAGCATTTTACTGACCTAATAGGCTACTTC
TCTCCAGAATGACGTATCACGTTTCAGGGCTACCCATTAAACACCCCCTATTAAAACAGCGATCAGCAGCTTATTTGGCCC
CGACTTCTTGGTGTTTTTCCTCACACGCACCAATCAGATCTCCCCATCGGCCTCCTCGACTTTAATCAGCCTGACACATTC
CCATTCATATCATTTATAATCTCGTCGCATCTTAAATGTTGCCCGTAAATCATAAGAGAGCAACATGTCCAGTGAAGCCAG
TGAGCAGCGGGCCTGCTCGGGTTTCCACACGTCTAGCTATAGGCCTCCGCACACATGGTAGCATCCTCATTTGTTCGCCTA
AAAATAGGACGAGACTACTAGACACATTTTTTCGAAACCCATGCATCTTGGTATACCTGATACTGAGACGCAAAGCGCTGT
AAATGGTGTTGCTGTCGGCTATAAAAGAGCGAGGGAAGGAGACTTGCGGCAGTCAAACGAGTCCGCCTTGGTTTTCACTCG
CCTCAACGCAGCGCAAAGGTGAGTGGGGAGCGCGCAAAGGCCGCACTTGGCTGGCAGCTTGCACTGGAAGGAAAGGGATTG
AGACCAGGAGAGCCCCGTGGACTATAACTGTAGGGAGAAAAGGAGCCAACATAGCCGACAACCACTGCTACCACACGGGAC
TGCAGGAGGGCCACACTTTTGATTTCTAAGGATCAACTTTTGTGGAGGATAATTGCGCTATGATCTCTTTTAACCGGATGA
AAAAAAAAAACAGGGAGGGGGAAGGAGAAAAAAAAAAGCATGTTTGCTGCTGCACTGTTTATTGTTGGCCTCACAGTGGGAG
CATCATCTAGCCTGTTTAGTGTCACAGCAGCATAACGCGTTGGGATACACACAGCCAGGCTTGTGTTTGTGGCATCCGTAG
GCAAAGAGTAGAGAAGTAAACGAACAGGAGCGGTGATGCTGCCTGACAGCATTTGCAAATTAAAGCAGTGAATTAGCTGAA
TCGTGACCGAAAGAAGCCGGTGGATATTTTTAAGCAAACAACTTTAAAATACAATGTTAATAATAATAATGCCTGATG
TGAAGCTAGAGGACACCGATGGTTTGAGTCTCTGTTTGATATCTGATGAGTTAAAACACAGGAAAGTCATGGGAGATGAGC
ACTTAGAATACATCTCTGAAGGCAATGCATCTTAGATCCACAGACACAGAATCAGTTTGCTTTAAAGCAGGATTTTCAGAG
CAACAACATGATCACTGTACTGGATTTGTGATGCTTAAACTAACCCTGAGCCTTGTCTTCTCTCTCTCCTCTAGAATAAAT
AAGAAAAAATGGCCTTTTCTGGTGTTCTCAGTGATGCTGACATGAAAGCCGCCCTGGATGGCTGCTCAGGTGAGCCTCCCT
GTCTTCACTTAGTGCCCTTTCTTTTCACTGCACATTTGCTGACTCCAAAAACATCTGGAGTGCACTCTTCACAATGTGTCA
CATAAACTGCCAGTTTAAACTGCAAAGTTATCACATATCACATTACTCAAGTAAGTCCTGTACCGTAGCAGCCTGGGTGCT
AATGGTGCTAACATGTATGTTCCAGCTGCTGACTCCTTCGACTACAAGAAGTTCTTCAAGGCATGCGGCCTGTCCGGCAAG
TCCGCTGATGAGGTCAAGAAGGCCTTCGCCATCATTGACCAGGACAACAGTGGCTTCATTGAGGAGGAGGAGCTGAAGTAA
GACACTCAGGAAATGCACACTTGAGCAAGAAATGGCCTCTAAGGCACCAAGACAAGATTTTAGAACTTGGTTACACACTTA
AAGCTCTAGACAAACTAAAGAAAGGCAATGTTTAATAACCTAAAACAGTTAAATACCTATGAGCAGGCTTATTTACACATC
ACATTGTGCCTTTAAAGGACACTCATTCTGCTCAGTGATTTCCAAACAGACCAATCAACACAGTCTGAGATGTTCATCGCT
TTAACTCCTGCTCTTTTGTCCACCTCTGTCAGGCTGTTCCTGCAGAACTTCGGTAAGTCCGCCAGAGCACTCACTGACAAG
GAGACCAAGGCTTTCCTCGCCGCTGGTGACAGTGACGGTGATGGCAAGATCGGAGTCGACGGTGAGAATCCTCTTTCCTGC
TTTGAATGTAGCAAAGATACACACGCCTGCAAGTTAATCAGCATTTAACATCCACTCACATTCTTCTAATATCTGCACACA
CTCAAAGTCTTGGTCAGCAAATGGGTCCAACCAATTTGCTGATGACACAAGAATCTGACTGTCTTTTGTTCTCTACTAATG
GCAGTCTCATTCTGTTTTCACAGAGTTTGCTGCCCTTGTTAAGGCATAAATTTCCATTGACCAAGATCCCCTTCTTCCTTT
CATGGAACTGTGAGCTCCTTGCAAGAACCAGTAATCATCAGCTGAAAGAATATTTTTTATACCTTATTTATGAGTTGCCTG
CGGAGTATTTCTCAAACATGGAACACATGCTTTTACTGTGAAATGTTATTTGGGTGTGTTAAATGTTCTGAAAAATGACTT
GTGCAAACTGTACATCACCACATGCACTATTGAGGAAGAGTGAAAAAACCAGGAATAAATACTCTTTTGGTGTAAAGTCAT

CTCCTTCAGGTCAGTCTTCTTTAGCATGCTGCATGGGGCTTTCTCAACACAATGGGCGGGGGATCTTAAAATGGGATTTTG
AAGAGGTGCTGAGAGGCAATTGAACCGACCTGCCCTGCACTTGTTTTGATAATTGCTTAACGGGGAGGGGGAGTTCAGCTG
GACACGAGCCACGAGCTGGCAAACGTAGTGGGAAAGGCCCTGGGGCTTTGCTTGCATGCTTCCAAGATCCTGGGAGGTTTG
CAACGGATAGCTGTCACACACATAGTGCAATTAGCTGGCCTGATCCAAGGTCTTTTTCTCCACCAACTGCTGCGTAGTCAA
TTAGATGATGTGCTGTGGCAACAGGGGGGCTATTAACAGCCCCTGTTGCATCAGCAGCCTTTTCAGCAGCTTGAATCTGGT
GCATAGATGCAGATCTTTAGATTTCTGAGGAGTTTTTTTAAAAAAATTTTTTTAAACAAATCAATTTTTTCTCTTTAGTTT
TCTCACTGGGATTTAAAAGTGACATAATTCTTTATACACAGAAATACTTAAGTGGAATTACAATCCTCAGGGGAAGCTACG
AATGCTCAGGGAGCTGCAAGTCAGCAATATTTAAAGCCTCAACCATATCAGATGACAGCCTGAGCTTGTTGCCATCAACCA
AGTGGTCAAAACAGAAAAGAGGCATGGTGACAGCAGGGTTACCTCACGGGACAGTGCTGCCACCTACAGGCCAGAGCTACT
ATCAGCATTACAGGCAGAGATTTAACTGTGCGGGCAAAGATTGAAAATTCCCTTCTCCCCTTTCCTTTCCTTTCCTTTCTT
TTCTTTCTTTTTCTTTTCTTTTTTACTTTATTTGGAGTTTATAGAAAGAAAAGTGTGAACAATTTGTGCTTTTTAACATGC
TGACTTATGACTTACAAAAAGATTGGCAAGGTGAAAATAGCAGTCGAAGGGGTTTTTGTCAAAAAATAATCTTTTTCTCAT
CTTATTTAGACAATGTGCACTTAAAAAACCAAGAAAACATGCCAAACAAAATGGGATTGATCCAAACTTGGAATAGAAGAC
TATTAAAAACCTGATCTAAATGCTTAATTCACTCCCAGACACAGGCTAAATCTGAACAAAACACAAAATATTAATTTAGTG
ATGATAGGTACACAGGCATCTATCGAAAACATCTGTTTCTCACAAGGTTAACTATAATGTGCACTCTGACACTATCTCATT
AATAAGCTCTACTCTTTTTCTCAAAGTGACCCCTGAATACAACAGTCAACACCCTCCAGCTCAACATTGTTGCTCAAAAAG
AGGAAAAAACAACCAATTAGCGCCACTTACGCGCCTCTAGCGCACGCACGCTCCTCCTGAATACGGCTTAGAGGTGCCAGC
AGCGCTTCCCAATTCGTCCTCCTCAGCAGTGCACAGGCCTATTCTTAGCGTTATGGGGGATATGAAAGACTTCTTCAAACG
CACCTTTTAAGCGTCTTCATTTGTGAAACTTAAAATAGACTCTGGCTCGCAGGGACGAGATTTTCGCCACCCCCCCTTCTC
CACGGTGTCAGTTATCTTATGCCTCAGATTTCCGATCGGAGCGAAACTATAAAAGGTCACCGGGGAGCACCGGGAAGATCA
CCGCAGTCGACTCGAGTCTTGTCTGTGTCATCTGTACCCCACCGAGCAAAGACAGCAGAGGTGAGTTTGAATGAAATGGAG
GAGGGAAGACAGCGGGGACCCGTGCGGGGAAAAGCCCGCAGCATCACGCTGGGCATCTCTGAGAAAACCTGATTTTTATTA
TTATTATTTTTTTTTAATGTGGACTTCACATTGCGATGAAGACGTGGATTACAGCGACTGTAGAGTTGTCACCCGTCAATC
TCTCGAGTGGGACATCAAACTCGGAAAGAAGCGGAAATTTGAATTTCTACTCTGATTTATTTCTAATCGGAATAATGACAA
TTTGACAATTCTGGCGACAGACTGTGAAGAGGCGATATGTTGTTTAAAATGGTCCATATCCACTTAATTTAATGTGATTCA
TTAGTGTACTTCTTCTAAAAGATTAAGCAAATATGAATCAAGTGCAGTTAAGTTGTTGCTTCACATGTCAGTGACTTTATC
ACCCGTAATATTCGCTTTTATTTGATTTCTGCAGTTCAGGATGCCTTTCAAAGGACTGAAGGATGCTGATGTCGCTAAAGC
CCTGGAAGGGTGCAAAGGTGACCCTCCACCTTACACAATCACAACCACAGCTCATCCTTGTTCTGTGCTTTTATGTGTAGT
TTGGTTTGTTGTTGTTTTAGTATAGGCTCACAGATCTGCACTCATTTCAGACGCTGGCACATTCGACCACAAGAAGTTCTT
CCATGCATGCGGACTGGCCGGAAAGTCCGGCGCGGACGTGAAGGAGGCCTTCTTCGTCATCGACCAGGACAAGAGTGGTTT
CATTGAGGAGGAGGAGCTCAAGTAAGACATTCACACAAATGTGCCCACACCAGTGAACAGTGCTTTAGGTGTATTGTTCTT
ACATGGCTGTGGGGAATGTAAAGTCACACTATCTTTAGCAGCCTGCTTCATCTTTCTGTTCATGTCCACCTGCCTTTTAAC
TATTGGAGGGGAATGTACATGAACAAGGCTCAAGAATCTTCATCAGGATCTATTTGTGGTCAGGTTGAGTCTGAACTGTGC
TCAGTCTGAGCTGAGGTCCGTGAACTAACCACCGCCCGTGTGTGTGTGTGCGTGTGTGTGTGCGTGTGTGTGTCTGCAGGC
TGTTCCTGCAGAACTTCAAGGCAGGTGCACGCGCGTTGACCGACGACGAGACCAAGAAATTCCTTAAGGCCGGAGACAGCG
ACGGTGACGGCAAGATCGGCGCTGACGGTATGGACTCTGCTCAGCTGTTATCTTGGGATATTAAAGGGCCGCTATGTAGTT
TTACAGAAGAAATTCAAAAACAGTTTTTAATGTTTACAACTTTAATGAGGTACCAATAGAGCTGTGCCCTAACACATCTAC
TCATACTGAGACAAATAAAGAGCAGCTGTGGAGGGAAGCGAGCAAGGAAGTCAGACTGCCTGGTAAGTTTTGGAAATACGT
CTCAGCACAGCATAGCTCTGATGGATCCGAACTCCATTCAGGGAACAAGCATTTTAAAAGTTGTTCACTTGTCATCTGGCC
GACAGACCTGATGAAGCATGAATTCAGGTTTTTTTACAAGGCAGCATTATGATGTTGTTATTTCAGCGTCCTTTCAATGCA
TTTATTGCAGTCAGATCACAGAGAAATCGCTTCATTTTGGGTTCATATCATTGTGGTAAAACAGATTAATTCAGTCTACAT
CTACTTGCTGGTCACAGTTACTATTTATTGATTTTAACACACCAATTAAGAAACTGCCAGTCACCAGAGACAGATAGACAG
GCGCTCTGCAGCTGAGAACCAGGCACCAGTCAGCCTCAGGTAGTGCTGGAAGCTGCAGTCATCCAGACACAGTGCTGGGTG
CCGCTTGAGTAAAATGAATAAAAATGAATAACCCTGTGACACCGACTAACTGTGTTTTTCTTGTGTCTCCCTGCAGAGTTC
GCTGAAATGGTTAAAGTGTAATGTGTCATACAATGGCCAATGACCAACAACACCTGCTCTGATGGAACAACAAAGCCCACG
TCGACCTCCCCCCTTTTCATCTGAATATAAATAATTTTTATACATTTGCTGAACAGACGTTTATCCTCCTATGCAACACAC
TGTCCAAAATGATGATGATTGTGAATGTCGCTCGGTTGTGTTCATGTCTTAAATATGAATTTTGCTTACTGTAAAATCTAT
ATGATGCACTTTCCAGGAACGAACGGTACAAAAAGAATAAATATTCTTTTGCTGTGGTCTTATTGGACTTTTGCGTGAAAT
AAAACAACCAAAAGAATGAACATAAACAGAAGAACTGGGACTTCTGTGGTTAGAAATGTATATATAGGTTACAGATAAATT
GTTTTACTTATCACTTACCTGTCAAATTCATACTCATCAACAAATAGTGAGTAGCATATCGTCATAGTAACTGCTAACTGT
AGCTGTCTTAGCCACTTAGCTCAGTTAGCCGTGCAGCTAGTGGTTCAGACTGGGAGCTCGGGTAACAAGGGAATTGGCAGC
TTTTAAACCGCTAGCACAGGAGCTTTGGACTGCTAGCTGGTTAGCGTGCAAATCTAGATACCACTGCAACAAGGTACATTG
ATATCATAATACCAAGACTGCTACTCAGTCACATTCTGTTGACCATTTTAGTTCCTTTTTGAATCTTTTCAACTACATTTT
TTTTTTACATATCACCCCTTTAAAAATATGAATTAAGTATAAAAATAGACTTTCAGTTAAAAAAACAGATATGTCTAGATA
TTAAGAACATTAGAAGAAGAGTATAAACGCACACACACTCTCAAATTACATTACATGTCTCAATTCTTTCTCTTTCTCTGT

TCAATCCAATGACTTTCATGAAAGTAAAAAATAGAGAAGAAGAAGAAGAAGACAAATTTGGCCAAAGCAGCGATTTGA
AAATATTTCGACAGTACATAAACAGGAATGGTGAGCAATAAATTCTATCAGCATCAATAAAAAGTCTAAATGTAGCCTGTG
TTCATGAGGCTCCATCCTGCAGCCACTAGATGGCCTCGTGTCCGTTAACAATAGAAGCTGAGACAGAGACAAGACCTTACA
ACGAATCATCTGGCAATAAAAAGACAGCTGTTTGGAAATCTGACTCTTTCGATTCAAACATTCCTGGCAGAAGAGCCTGAA
AATAAGTTATGGGGCCCTATTCACATGGGAAACACATGCAGGACCATATAATAATCATGTTTTATTTCCAAAAACAAGCCT
GAACGGAATGAAGGGAGAGGATCTATACATCCAGGCTTGTAAAGGACATTAGCAAATGTAAATGAATAAATGATTGCCCAG
TAACAGTATAGTCTGTCAGGTATCAACATGGCGCATGACATCATGCACCGGGTGTGGGTAAAGCTCTCGATCCGGTGTCCT
CTCCTGTCTCTTTTTCACACACAGTCCTGAGGAGATGATTGAGTTTCTCTTTACCTGCTCAGGAGTGCGGCTTCACGAGGA
GCTCTCAGCAGCAGCAGCAGAAGCAGCAGCAGCACATCACTTGTTGAGGGGCCCCATTAACAAGTTGGCACCGGGATTAGG
TTTAAGAGAGGCTGACAATGACAAACCACCGGAGATCCTACAAGCTTTTATTCAATAATGAAAGACAACAGGGTGCTGCCA
GACAATTCACCAAAAAAGCTGCAGTGCATCCACTGTGCACACAAAACACCTGTTCTTGTCACTGAACATCATGTCAAATTT
AACCCAGAAAATAACAAATACAATTGAAGAACATAAAATGTATTTGGATTGCTTCCTATTGACATATGTCTTCTGTCTTCA
GGCTTTTAAATAATGTTTAAGAGGCTCTTTAGTGCATGTCCTGACATCGCCCTGGGGCCAACACTATCTGCAGCTCACGGC
CACCAGCTGACGAGTGCAGAGAGCGACGCGCTGCCAAAAGTCTGACTTCTAATGTGAGGAAGCTGGCCGGGGGCTGCTGTG
TCCCTGCCTTTGGTTACATTCAAGGATATCCCCAAGTGTCAGGTTGCAGTCATTTTGGAGAGAGCACGCCAGACTCTGAAA
CCAGTTGGACTATTGATTAAATTCGAGCATTTATGTTTTAATATCTGGTTTAATATTGTGCATAATGATTGCACTGTGCAT
GTCTGTCTTTCTCCTCCTCACACTGCTGCTGCTGCATGCTGCATATGCACACCTTCAACTAACCTTACACCACGGAGAGAA
ATAACCTTCTTTTTCCATGTGCTGCTGCAGCCAACATTCTCCCAGGAGTGTGCGTAATGACCGCTGGATTTGTTTGTGCCA
GAGCCGCCGGGGGCCACAAACGCCCTCGGAAAAAGACAACCCTAAATTGGTCGTTTTGCAGCTGGACAAAAAGTCCATTGT
GCAGCTCTGTGTTCCCTTAAAACTGGATGAATCAAAGTGTCCAAATGACCAAAGTCCTATATAAATGAAGCCTTGTTTGAC
CTCTGACTGCTACACCTCTCTTTTAATAGTGCATGTGAATACCTGGGACACTGCTCATAATGCATCATCATCACCATCAAG
GCAGGACGGGACACACCGACAGCAGAGGGTGACCTCGCGTCCCTTAATCAGCACAGTTGACAAAGCAATTTAGCGAGACTG
TCCCCCTGTCAGTGTGTGTGAGTGAGAAGGCTCAGGGAGTCCCTGACATTCACCGATCAGCAGCTATATGTGTTTGAGGTC
ATATCACTGTAGACACCATTAAATATACTTAACTCCGTCTGTTGTCAATCTGAGCAACGACAGCCGATAGAAGCTTAATGC
AACCAAGGCAGGTGAAGCAGAGTGCACCAGACAAACAGCTTACACATGGATTCACCATATGGATGATACAGTTAATTAAAC
TTTGGTTAATACTTATTTCACTGCTACTAAAACGATTTAATGCTTTACAAAACCATTTCTTGAGCAACTGTAAAGGTTTAT
AAACATCAGTCTGATCTTCACAATAAACAATGAACTAATAAATTGGTTAAGCATTTCATTGTTTGTTAACAGTGAGATAAC
TATTAACTAAAGTTTAAGCAACAGGGCTCCAGGCATGTGCAACTATTTTGGTCACACATGCACCCAAAATTATTTCCTTAA
ACAATTGAGGTAATTGCGATTTCTTGCTGCGTGTGCTGAGAGTTAAATGAAGAAAAACACGTATTTGCAGCTCTTACCTGT
TAAAAATCATTCACATAATGTGAAAATAAATTAAAATGTAATATTGGGTGCACCTTAATGGAAAAAGACGCACCAGTGCAG
CCAGTATGAAAGGTAGTCTGGAGCCCTGATTAACTATACATTTAATTTAATGATGGTTATTACAAAGTGTGAATGAATTGA
TATTCCTCACTAATGGATCTCAGTGATAGGACTGATGGAATGTAACTAAATACATTTACTTAAGTACTGTATTTAAGTACA
TTTTTCAGGAACTCCACTCCACTTCATTGTACTTTTTTTTTCAACCACGTATATGTTATAACTTCAGTTACTAGTTGCTTA
TAGATTGCATGCTGCATCAGACCCAAAGCAGCATTTTTTTTTTATTAATTGATTTTTAATTCCAGTTATAAGAAAAATGTG
GATTATCGGCTCTGCTAATCGGCCAAGCGCGTGATCGGCCGAACTTTAAATGAGTATACAGTACATACATGAAAACGTACA
GTACGTGCTTTTACTTGTACTTTTGATAATTAAGTACATTTTATATGGATACTTACAGACTTTTTCTCAAGTATGCTACTA
TTTATAAGGGAGACTTTCATCTTTACCAAAATAACATTTTAAGCAATATCTTTACTTCTCCTCAAGGTGACACTGAGTCAT
TTTGACCTCTTCCACTCCATAATCTGTTCTATGTATTCTTTGCACCATATTCCTTCTGCAACAGCTCAACAAATGTGCCTC
GAATGTAATAAAATGGGCATATTTGCTGCGGGATGTAACTCAGATGTGCTCGTTTTGGGACCGCTGCCTGTAAAAGGATCT
CTGCATGAAAGGGTAGGGGTGGTGGTGGAGGGGGAGAAGGGAGAGAGAGAGATTGCAGTTACATTACAGCCTCCCTGCATT
TACTGTACTGTATAATTCTGGAGGTGAGAGGAGGAGGAGGGGGAGGAGGAGGAGGAGGGGTGAGGGGATGACAGGGAGGGA
GTGGCGGATGGGGGAGAAGAATGCGCTTTTTAACTTAATTTGCTATTTACAAGGGCGTTACAGAGGTCCGCGGTCCGGTCT
CTGTGTCCCCGGGCGTTATAAATAGTAAAGGTTAATTAGTTAGCTGAGAAATTGGATCAGGGGCGGGCAGTTAGAATTTGA
TCCACAGACAGGTAACTCAAGCAATCCCCATTGTCACTGCAATCAGACCTATATAAATCCGCCTTGACAGACAGCAGTCAC
CACATAGCCTTACCTCTCCGCTGGATCACCGACCGGGCGAGATCTCCCACTAACACGGTAAGTTTGGCTGCGAGGATGCTT
TGGATGGAAGGTTTTTTTTTAAACTTGTTTTTAGACTTGGCTCCTGCAGATTTAGCGCACTTTTTTATTTTAGAATGGAGA
GTCTGCCTGTAGTGAGGTGACTGGTGAAATAATGGCGTGCGTAAAAGGAGAGACTTTTATGCGTCGGAGGCGACAAAAACA
CAAAGAAAGAAAGTTATTATTTCTCTCGTGTTTGATCTGCAGGATGAGATTAAGATTTCACATCCTGACTCTGTAGATTTA
AGAACTTTTCCCTCTCCTGCACTCACTTCTGTGTCAGTTTTGTCACTTTCTACTCTCCATCAGTGTCAGCTTCTATGTTGT
GCTTCTGTACCTGGATACATATGTTAATAATTCACCATGGTCGTTCCTCCTACAGGAATAAAATGTCGCTCTCATCTATCC
TCTCCGCTGATGCCATCGACAGTGCTATCAAGGACTGCCAAGGTATCATCTTCCACCAATATCATTACTGCTCTCTGCATT
CGATTTGTTTTGTTCTATACAGTATTTCTAATGGAGCCTGTGCGCTCACCAGACTGCAAGATGTTGACATGAGAAAGATAC
ATAAGTGCAGTATAACCTAGGGCACAGGGTGACATTTGTTAAATGATTTTAGGAGCATCATTCGGGTCCACATATGATCCC
ATCAGTGGAGGTCAAAGATTAAGAGAAATTGGCCTCTCAAACCAGTAAAATATAAAATATTTCCACAGGGAGCGTTCAGGA
GCCTCTGGACAGCCACTTACATCACACTTAATTAACACCTGAGCAGTCATATGTCATCTTCTCTCACAATGTAAACAGACT

TAACCGTCTGAGAATTTGCTGATACTAAGGGAGTTTGCTTTAATTCCAGTTTAATGAAAAAACTAGAACACTAAATATTGA
CATGTACCTGGTGCCGTTCTCTATCCTCCTCCAGCACCAGACTCCTTCTGCCCCAAGAAGTTTTTCCAGGCTTGTGGCCTC
GCCCAGAAGAGCCCCCAGGATGTGAAGAAAGCATTCGGCATCCTGGACAACGATGGCAGCGGTTTTATCGAGGAGGAAGAG
CTCAAGTGAGTGAGCGACTCTTTCTTCTCTGTGTTTGCTGGTGGAGAAGATTTGACGACTTCTTTGGAAAGTCATCGCATT
TCTGTTTAGTTTTGCTGATGTTTTTGTGCCAGAGGGGATGTAGTGAAGGGGATACCCGTACATCATGAGTTTATCCAACTT
CTCAGGGCTGACATGACTTTTAGCATCACTCACTAGAAGTTGTGTTCTCTGGTTGTACAGAAGCACCCAAAACCTGATAAC
CTTTGGGAATTCCATGTGACTTGATTGACATCCCTTTAACTTGTTTTACATCTTGTTACCTGTGCAAATGTATCTTTTGTA
AATATATTGTACTAGGACAGATTGGACCCCTGTGTTCCTGCATTCTGTCTTTGTGGTCATGTTATGTATCCTCAGTTTCTG
TCCTGAACTTCTGTGTGTGTGTGTGTGTGTGTGGCAGGTTTTTCCTCCAGAGGTTTTCTCCCGGTGCTCGCGTCCTGACAG
ATAAGGAGACCAAGGGCTTCCTGGCTGCTGCTGATGACGACAGTGATGGTCGGATTGGGGCAGAAGGTGAGATTTTACATC
CACAAAATTGCAAAGAAAGACAACCGCAATGCTATGGAAACAATTTTCCAAACTTTTTGTATCTTAAATTGGAGATAGACT
AAATTTAGCTTGGGATGGTCATTCTGGGTCAGTAGGTAACACCAGAAAGAGAGCTGTATATTCAAGCACTAAGGTAACACA
AACAGCGATGCCTTTGTGTCCCGACACACTCCTTCTGTCCTCCAGCAGCTCAAAACTGACACCTGCACAGTGTTTCTACAT
CTGTAATTCTAGATTTGTGAATGAGCTTAATTGATCCGACATGTTGTGTCAGTGGTGTGCTGTTCACTTGAGAGCACAACA
CCGGAATTAGCATCACAATGCTATCTCTCTGTGTCTCTACTGGTGTCATGTCTTTATACTTGGAGGCAGGTGTCACTTCTT
CTAACGCTGTTTTCTTTTCCTCCACAGAGTTCCAGGCCATGGTTTTGTCCTAATCACCTGGTCGCCATCTCTCCAACTCAA
TCTGACCCCCATTACCCCTATTCCAACGCTTCAAGGGCTCTCTTAGTTTTAGTCGTTTTCCGTCTGGTTAGATAAAACCTT
TTAAATTATTTTTATTTTGCAATGACTCGCTCTGGTTCACAGACGTACACATTCTCATCATGTTTTTTTTTTCTTTTTCGT
ATGTTTTAGACAGACCATAAAGCTGTATTTCCCAAATAAATGTACAATTTACCAAATTACTGAGAAAATAAAATGATAAAA
TCCAAAAAAACCCTCTGTGGCTCAAGTGTACATCTTTGCATGTTTCTGTATGTCTTATAATCAGATATGGTTTGAGATGTC
ACCAAGTGGCATGACAAGAAAAAAATATTTTATGGTAGTGTTGAAATGTTTCATATCTGCACATGCACAGAAAAAGCAAAC
GTAAAGTCACACCATTTTCTTAACAGTTCCTCTGTGTCCTTAAACCTTCAATTACCCAGCGGTGAATGAGACTGAATGTCA
GACAGTATCACAGATATGAGAACTGAGACATGTCGTGCTTCAGACTGAGGTGTATATACAGTACAGGTGTGTGTGTGTATG
TGAGAGAGAGAGAGAGAGTTTAGAGCCAACTCAGGGAAAAAGCAGAGGAGGTGACCTTGATGACCACCTGAGTGTCACCGC
GGCTGGATCGAGTGACACCTGACCTCAGCTTTGTAAATAAACCCAGCTGAACCTTGGTGCTGCTCGGGCTGGGGCAACAAA
CCACACACACACACACACACACACACACGAAACCCAATGCATTTGCAAAAATCAAATGTGAGATCAAACACAGGCGGCCAT
CCATGCAAACACAAATAATATATGCACATAAATACACGCACATCAGAGTTGGAGGTAATAAAATGCATCAGTGTGGATGTG
TGTGTATGTGTGTGTGATGCACAGATGCAAGCTCTGCAGAAAGCTATAGCCTAGGATGCTAGCCTTACATGTATTCCTCTG
CCAGGCCCTCAGAGAGCTTGCTCAGGATATATTAAAGAAGAATCATTTTATCACACCTCTGCATCCATCCTCCGACAGACT
TTAAATAGCACGCTGTGTTTTCAATCTGTGCACTTAGAGGATTCACAGGATTAAAATTAAAAACTACCTCACTGTTCAAAC
GCCCGTCACAGGCGCCAAAATACAGTCCAGCAAGTTTTTTCAGCGGATCCACCTCGGTGTCATTTCAATACCTGTATATTA
CAGGGAGAAGCTGACTATTACAGCAGACACCTGCTACTCAGAGCTGTGGGGCATACCCTGGTATTAGCACGAGGTTGAAGT
GTAGGATGCTGCTTGATTCAGATGGTTTGCTGTGATAATGATAGAGGTTACAAACCGCAGTGTGTTAAACTGATATAAATG
TGACGAGGCCATAATGTTTTAAAGGCATACTATGCAGGATTTGTTGGTTGTCGTTTGTAAGTAAAATCGCGAGTCAGCAAC
CAATCAGTGTTCCCATGAGGAACAACTAGCAGCTACGGCGTGATTTAGGTGTGTCAACCGGCAAGAGAGGCGACTGTTCAA
CACTTTCCAGATGATATTGTTTTATTAGTTTTGACACACCATTCAAAAGGTGAAAGATAGTTGGTTTTTGGTCTCATTCTG
ACGGTTTGGGTCGGCAAAAGCACCACTGTTTGATTACCTGGGAATGAGACTAAACAATCAACTATCTTTCTCTAAAATCAT
TGTCACCCAGAAACTTGCCCAACTCGTCAGATACGGCAGCTTGGTCAGCGGCCTGTAGCTTCAAACTATGACGCAGTTTTA
AACGTGAATGGCTCCGTGGTAAACTTAGCAATGCAACGTCCAGTAAAACTTTAAGAATTAAGTTTGCTGACAAACAGTTTT
GAAAAGATAATACTTCTTCATAACTATCTTCACGCACGTAACTTACACAAGTTGTGTAAGTCCATCCATCCCTACCATCAC
TGACTCAGACTTTTCTCGTTCTAAAGACTACGGCACTTGTTGGGCGGTAGAGGGGTACCTGCATTGTCAAACTTAGAGGTG
TATGGCCACTGACCAGGTTGCTGTATTTGACGCATAAGATTTAGAACGGCCGTGAACACAGCAAAATCATAAGAAAAAAGA
AAATACTGGATTCAAACACTTCTTGTGGGTCACAGAGTCTATACATGATTTTTTTTTAGGAAATTCCTGCACAATACATCT
TTGATATGTTAAAATTAGTGACCATTTCAGACTGTATCAGTGAAAGTCGTTTCTATAACTCAGTGAAAGACAATCCATAAT
CGATCTAGTGTAAATGATTGAGTGTTGTATAATCAAAGTGCAGCGATGTGACTGAAGCCAGAGGTATTAACAGGAAAATAC
ACAAGATCAGGCATTCGTGCTTACAGTAAAGAAAGATAGTATCTAGTGGTGTGGGTTGAACTAAAAGAATGCAGAAGCAAA
CTTCAGACATCTTTGCATATTAATAGATTATGATTAATTAAAGAACTTTATTGCAAACTGTTTGTTCAAAATCCTATAAAT
TTTTCTTAACTTAAAGAAGAGCTACACAACTACACACTTCTCTTAAGCTCAAACATACTGTATATTATGCATATGGATACA
AAACATATGTGCATATTGTTAAAGAATGTTCATTAGATACATTTACAGATAAATTTATACCTAACAAGACAAAAAACACTT
GACATGCACAGAGAGACACAGCGCTAATTAGATCATCATGTATCAATATCTGCTGTTATCAGTCTGATCGCTGCAGATGGG
ATGGCAGAGAGAGAACTGGGGGGATGGAAGGTGGTGGTGGGGAGGCAGGGTACACGACTGAAAGCGGGACAAGGTTGG
GGAAGCCAGTAGGTAACCCAAGCCAGGGGACACACAATGGAGCCGTGACCCCTGCGGGTCATGAGAGGCGAGTGGTAAACA
GGAAGGAAATGTGAGCCAACAGGTCGACACTGACTGCTGGATGTGCTGCTGTGAGACAGCAGTACAAGATAATGTGACAAG
CAGATTACCTGGACTCAAGGGCAGAGAGATGGGGCGAGAGAGAGAGAGAGGGAGAGAGAAAGGGGGGGGGGGGGGGGGG
GAGCGACTGTAGTTTTGACAGGCCATTAATGTACAACAGTGCTGCTGGGATGATATTGTGAGAATGAAGACAGAAACCAGA

GGTTATTGGAGTGTAAGGTGAAGCATGGAGGAGGTCTTCATAGTCTTTCACAATATCAGCCCAATTAAAACGATTGTCATT
TCAAGTCCTGTGTGGTTAATCATGTACTCTCAATATATCGTTTCAGAGATAATTATTGAATGTACTGTATATGACCAGTGA
GGGTGTGATTTTATATTCTAAGAATAAAGGGAATTAAGGGTGAGGTTACAAGAAATAACCTGGTCCAGTGACTTGGCCATC
CCCAAATCTGCTTTACTCATATCAACGCATGTGTGTGCACTCTCTCCTACATAAGCACATGCAGAATGTCTGTTGTCTGTC
ATTATGGACAACTTTCTCAATCACAGGCGGTGGTGGCTCCTTCACTGACGCTAGCCACCTCTTGACTTGTAACATTAGCAT
CCTGGCTATTGTTCATCTCTTCAGCCTGGCTTTGTGACCTGACTGAAAAAAGTTAGTCAGTAGATTATTATTTGGGTCTTA
AGCTTGTGCCAGCTGCTTCTTTTTCTTCTTCTCTCGTTTCTGGCATCCTAATCCACGTGTAGGTCCTGGCAGCAATGAATT
ATTGCTCTTAATTATTACACGTAGGTTTGGTGCACCAATTTACGCTATTTTCTGCATCAATTCATAATGGAAATATTCTTT
ATTGTGTATAGGAAAACACAAAACTCAGATCCATTTGACAATTCAAAATATAATGGAGGATAATACAGTAAGGCTCTCTGG
CGAGCACCTTGTGAAAATAAAAGTCCTCTGCTCAGGAGTTGTGCTAAATCGTATAATTTAATGTAATGTGATAATAGTTGA
AATATTGAGGGGGACGTGTCCCTTGTGTATCCCTTGAAAGCTACACCTGTGGTAGCTGTGGTAGTAATAGTAGCAGTTGCA
GCAATAATCACCCTGAACTGTTGGGATGCAGCATTAGGTAACAAGAGTAGAGTGAGCAAGCGTAACTTAACTTTACTTTCA
TATTAAACTATTTTGTGTGACTGCTAATACTGTACCGTGTAGTTCATCTAGACAGGCGCTTTAGGATGGACACATAGGCTT
TCGCCTGCTTGTCTGCTTGGCACTTCAATAATTGATGCTCTCGCTTCATTGTCACCAAAAACTCATATCAAGAAAGGAGTG
TCCGGCTCACCTTACTGATTAAGTCTGTGTGAAACTTTAATGTGCACCCCATCTTCAACTGATAACAAACTCGAAGTGCTC
AAGCAAACGAGGTAATGGGAAGCTGAACAGCTTGGACCTCCGCCGTGCGCCCAGTGCATGCTCGGACCAACGCCGATCATA
ATTTGACTCATTCCCGGCCTTTTGGAGCCTGGAGGTATCAGGGATGTTGCTACCTGATAGTCTGAACAGGGAGGAACGTCG
TAAAAGATTCAACTGGCGTGTTAGGATGACTGAATGGGCTTTGTGAGCTCTGCGCAGGAACTCTATCCATAATGCAGGGGA
TGACAGCATTGCCACTGTTCCCCAACAAGCATTAGTCCTGAGCCTGTGCGTATGATGTCTGCGTGAAGCTTCCCTTCATTA
ATGAATGATAGGCCATGCACATATCAATGGCCACTCCTGATACACACAGGGCATTCGTGGAAGGCCATTTTTCGAAGTCTA
TTTCCACCCTGGCTCAAGCCTATTGTTTAGATATCCGCTAATTGATGTGTTTTACAAATTAAGCTGTGTCTACTAACTGGA
CTAATGCAAGATTGCAATTTGAACTAAAAGCCACACCCTGCTTAAAGCCCGGAGCAGCTGCTACATGTGCTGACTCACCGT
CAGACTGAGTAATGGCAGCCGTGGAGATGTAGACAGGTACTTTGCAGACTTTTCCAAATGAGAAGGCACAATCAGATAAGA
TGGAGAGAGTCATCCAGGACAAACTTACTTAAACTAGCTGAATAATGGAGGCAAACTGTCTGGTCAGAGAGGACAACGAAA
GGATGTTTGCGTCTCGGTGGAGTTTGGTCCGTAAACACCTTACAACATCCGTAAATGTCAAGGTAGATACGTACAGAGCAC
CTTCATAAGCTTGGATTACACCCATGGTGCATTCAGACAACATGACGTACACGTGTCTGCATCTTAATCGCAGTTTACAGT
GATAGTAACGCGGTAATAACATTACAACTACTGCTCTTAATGTTATATTTTGCTTTTGTTTTGTTGCTTTATGTTCCTTCA
CCCTTTAATTTTACAAGTTCAAGGACAAACAAAGTTTCATCTCCTTATAGCTGTGTAAATAAATATTAAGCAAACAGAGCT
CAAGTATCAACTCATCCCAGCACACCCTCAGGCTGAATACTGCACATGTACAGTGTTATGATTGATTTGTTTCCCTGTCAA
AACCCTTTTACGAACTTAATGCTCCTTCACCATGATAATAATCTTCAAATGAGTAAGATTTAATAACCACACTGTCCCTGC
GTACCACGTGCGATTTATAAGATGTGATCAAGGCAAAGCCACAACTCCCAGTCAAGAAAAAAATCACAAAACCTCCTGTAC
AACTACATATTGTTGTTTTTACACTTCAGGTTTTGCACGGATTAAAAATCAAGATATATAAAAGTTAATTAGTAAGCTGAT
TTTCCCCCTGTTTCCAGTGTTTGTGTTAAGCTAAACTAACCAGTTTCTGGCTTTAGACTTATATTTAACCAGGAGTGGAAG
TACAGCTTACAATTACTTTATTTTTCAACTTGGATTTTTTCAAGCCTGTTATTTTACTTGCACTTAAGTATGCATCACACC
ATGTAATGTACTTAGTTACTTTTAAAATCATAATTGAATACTTCCTGAGTACAATTTAAATCCATATCCAAACCTTTCATC
TGCATTTTTCTTTAGCCAATAAGGCTAAACACCAATCTCATCACAAACAGGCAGTGGTGTAAAGGCAAGGCACGTTTGTAT
GTATAGCACAATTCAGACACAAGGCAACTCAAAGTGCTTTACAGGGGCATACAATTAAATTTATAGACATTAAAAATTGCA
TTTAAAATACATTAAAAACAAGTAGGAAGACATCAACAAAAATAATAGGAAAATAAGCTAAAATAGAATAGGTTTAAAAGA
GAGAAGACTAAAGAATAAAAGTCACGGTGCAGTTCAAAGCCTTGAAATTGCTCCAGTGAAAGGCAGTGGCAAACAGAAAGG
TCTTCAGCCTTGATTTAAAAGAGGTGAGAGTTGGAGCGGACCTGCAGTTTTCTGGGAGTTTGTCCCAGATATGTGGCGCAT
AATAAAATAAAGAGCACTGAAAATCTGTACTCAAGTATTAGTTCAGTTACATTACTGAAATATTACTCAAGTGCAAGTAAA
ACTACTGGTATTAAAAAAATACTTAGTAAAAGTACAAAGTATTCTTCTCAAACGCTACTTTTTTACCGCTGATGTTACTCT
CTTGCCGTCTGTGAAGAATATTTATTTTAATACATGTAGTTTTACTTGTAATTGAGTAATATTTCAGTAATGTTACTGTAC
TTGTACTCGAGTACAGATTTTCAGTACACTTCCCACCACTGAAATCATGTTTTAACGGGTTAATGAGTGAGCTGATTTACT
TTTGGACGGAGCCAGGCTAGCTGTTTTCTCCTGTTTCCAGTCTTTGAGCTAAGCTAAGCTAACTAGCTTCTGGCTTTAGAC
TTATATTTAAAAGACAAATAAGAGAGGGTTACCAATCATCTCATCTAACTCTTTGCCAGAAAGTGAAGTATTTCCCAAAGT
GTCCAACTTTTCCTTTGAGGTTTTACTCTAAGCAAAGTATTCATTGCAGTCTCACAGTTCACACACTGTTTATCTATGTCA
GTGACCCAGTAAGACTGTGCAAGAGCACCTACAAGCCCTGTGTCACTCCACATACTACAGCCACTCTACAGCCGGACGTGT
ACCTTGTTGCCTACATGATTTAAAGAACTACCGTGAACTGTCAGGTTTCAGCTCCCATCGTAGTCTCAGTTTCTCGCTCAG
GTCTTAGGCTGGCCATCAGATCAGATTATTACCTCAGCAGAGCTGCCAGGTTGGCCCCTGTCCGTACACACACACACACAC
ACACACACACTACTCCTCCTTCCCTCTTTCTTGTTAGACTTCAGCAAACAGTGAATTATTCAGGGTTAACAAGCTGTCGGA
ACGGCAAACTCAATTGTGCATTTTCATTAAACCTCTCTGGCCGCGTCTCTTGTAGCTGAAGAGGAGAGGATGCGGCTAAAG
TGTGAAACAGCACTAAGCCGAGTCCTCTGTCAGAAAGGTGACAGAGAATGAGGTGAGTGCAAGAGGACATATCAAAGGAGA
GCCCAGTATCAGGCAAAGTCATGGCCTCTTCAATTGCTTCCTGGTAAAGGATTGTGTTGCCAGGTTTGATTAATACAAGGA
TCAATTAGACTGAGCGGATTGATTTTGATCGTTGCAACAGGTTGACTGCTGTGTAGCTGCTAGACTAAGGAAATGTATGAC

TGAAATAAATTTCAAGTGTCTCATCTGCTTTCATCCTAATCTGTCTCTGAAGCTGACGGTGACTGGACTGTTGATTCCACT
TATTCCAGATTCCAGATTGACTGTTTTATTTTCCTGCAATTAGACTTTTTATTGATTTAGTTTTTAAGATATGATTCTTAA
TGATTCTGATAATCAGAAAAATCGAATCTGATAATTGGCCGGGTGGAAATGGTTGACCCATACTATACAGTGCAAACATGT
AATATGTATCATTTAGGCCAAATTTTACTTTTTACAATTCATACTTAAGTACATTTATTGCCAGAAAGTTACTTTTGATAT
TTAAGTACATTTAATGGAACTTTGAGGCTTTTACTCAAGTACTATTGGTATAAATGCACAGTAGAAATATACAGTACACAG
TAGAAATACACAGTAGAAATACACAGTACACAGCAGAAATACACCTCACGCAGTAGAAATAGACAGTAAAAAATACACATC
ACACAGTAGAAATAGACAGTCGAAACACACAGCACACAGTAGAAATACACAGTAGAAATACACCTCACACAGTAGAAATGC
ACAGTAGAAATACACAGTACACAGTAGAAATACGCAGTAGAAATGCACAGTATAATACACAGTACACAGTAGAAACACACA
GCACACAGTAGAAATGCACAGTAGAAATACACAGTACACAGTAGAAATACACAGCACACAGTAGAAATACACAGTAGAAAT
ACACAGTACACAGCAGAAATACACCTCACGCAGTAGAAATAGACAGTAGAAATACACATCACACAGTAGAAATAGACAGTC
GAAACACACAGCACACAGTAGAAATACACAGTAGAAATACACCTCACACAGTAGAAATACACCTCACACAGTAGAAATGCA
CAGTACACAGTAGAAATACACAGTACACAGTAGAAACACACCTCACACAGTAGAAATACACAGCAGAAATACACCTCACGC
AGTAGAAATAGACAGTAAAAAATACACATCACACAGTAGAAATAGACAGTCGAAACACACAGCACACAGTAGAAATACACA
GTAGAAATACACCTCACACAGTAGAAATGCACAGTAGAAATACACAGTACACAGTAGAAATACGCAGTAGAAATGCACAGT
ATAATACACAGTACACAGTAGAAACACACAGCACACAGTAGAAATGCACAGTAGAAATACACAGTACACAGTAGAAATACA
CAGCACACAGTAGAAATACACAGTAGAAATACACAGTACACAGCAGAAATACACCTCACGCAGTAGAAATAGACAGTAGAA
ATACACATCACACAGTAGAAATAGACAGTCGAAACACACAGCACACAGTAGAAATACACAGTAGAAATACACCTCACACAG
TAGAAATACACCTCACACAGTAGAAATGCACAGTACACAGTAGAAATACACAGTACACAGTAGAAACACACCTCACACAGT
AGAAATACACAGTAGAAATACACCTTACACAGTAGAAATATGCAGGAGAAATGCACAGTATAATACACAGTACACAGTAGA
AATAGACAGTAGAAACGCACAGCACACAGTAGAAATACACAGTAGAAATACAAAGAACACAGTAGAAATACACAGTAGGCT
TTTACTCAAGTACTATTGGTGTAAATGCACAGTAGAAATACACAGTACACAGTAGAAATACACCTCACACAGTAGAAATAC
ACAGTAGAAATGCACAGTAGAAATACACAGTACACAGTAGAAATACACCTTACACAGTAGAAATACGCAGTAGAAATGCAC
AGTATAATACACAGTACACAGTAGAAATGCACAGTAGAAACACACAGCACACAGTAGAAATACACAGCACACAGTAGAAAT
GCACAGTAGAAATACACAGTACACAGTAGAAATACACAGTAGAAACACACAGCACACAGTAGAAGTACACAGCACACAGTA
GAAATGCACAGTAGAAATACACAGTACACAGTATAAATACACAGTATAAATACACAGTACACAGCAGAAATACACCTCACA
CAGTAGAAATGCACAGTAGAAATACACAGTACACAGTAGAAATACACAGCACACAGTAGAAATACACAGTAGAAATGCACA
GTAGAAATACACAGTACACAGTAGAAATACACCTCACACAGTAGAAATACACAGTAGAAATACACAGTAGAAATACACCTT
ACACAGTAGAAATACGCAGGAGAAATGCACAGTATAATACACAGTACACCGTAGAAATAGACAGTAGAAACACACAGCACA
CAGTAGAAATACACAGTAGAAATACAAAGAACACAGTAGAAATACACAGCACACAGTCGAAATACACAGTAGAAATGCACA
TTAGAAATACACAGCACACAGTAGAAATGCACAGTAGAAATACACAGTACACAGTAGAAATACACAGCACACAGTCGAAAT
ACACAGTAGAAATGCACAGTAGAAATACACAGTACATAGTAGAAATACACCTCACACAGTAGAAATAGACAGTAGAAATAC
ACATCACACAGTAGAAATACACAGTAGAAATACACAGAACACAGTAGAAATACACAGCACACAGTAGAAATATACAGTAGA
AATGCACAGTAGAAATACACAGTAGAAATACACAGTACACAGTAGAAATACACAGTAGAAATGCACAGTAGAAATACACAG
TACACAGTAGAAATACACAGTAGAAATGCACAGTAGAAATACACAGTACACAGCAGAAATACACCTCACACAGTAGAAATA
GACAGTAGAAATACACAGAACACAGTAGAAATACACAGTAGAAATGCACAGTAGAAATACACAGTACACAATAGAAATACA
CAGTAAACAGTAGAAATACACAGCACACAGTAGAAATACACAGTACACAGTAGAAATACACAGCATTTACTGTGTATTTCT
ACTGTGTGATGTGTATTTCTACTGTGTATTTCGACTGTGTGCTGTGTATTTCTACTGTGTACTGTGTATTTCTACTGTGTA
TTTCTACTGTGTGATGTGTGTTTCGACTGTCTATTTCTACTGTGTGATGTGTATTTCTACTGTGTATTTCCACTCTGTATG
TCTACTGTGTATTTCTACTGTGTGATGTGTATTTCTACTGTCTATTTCTACTGTGTGAGGTGTATTTCTGCTGTGTACTGT
GTATTTCTACTGTGTGATGTGTATTTCTACTGTGTATTTCTACTGTGTGATGTGTGTTTCTACTGTCTATTTCTACTGTGT
GATGTGTATTTCTACTGTATATTTCTACTGCGTATGTCTATTGTGTATTTCTACTGTGTGATGTGTATTTCTACTGTGTAT
TTTTACTGTGTGATGTGTGTTTCGACTGTCTATTTCTACTGTGTGATGTGTATTTCTACTGTGTATTTCCACTCTGTATGT
CTACTGTGTATTTCTACTGTGTATTTCTACTGTGTCATGTGTATTTCTACTGTCTATTTCTACTGTGTGAGGTGTATTTCT
GCTGTGTACTGTGTATTTCTACTGTGTATTTCGACTGTGTGCTGTGTATTTCTACTGTGTACTGTGTATTTCTACTGTGTG
CTGTGTATTTCTACTGTTTACTGTGTATTTATACTGTGCATTTCCACTGTGTATTTCTACTGTGTTCTGTGTATTTCTACC
GTGTTCTGTGTATTTCTACTGTGTATTTCTACCGTGTTCTGTGTATTTCTACTGTCTATTTCTACTGTGTGATGTGTATTT
CTACTGTCTATTTCTACTGTGTGAGGTGTATTTCTGCTGTGTACTGTCTATTTCTACTGTGCATTTCTACTGTGTACTGTG
TATTTCTACTGTGCATTTCTACTGTGTGCTGTGTGTTTCTACTGTCTATTTCTACTGTGTGCTGTGTATTTCTACTGTGCA
TTTCTACTGCGTATTTCTACTGTGTAAGGTGTATTTCTACTGTGTACTGTGTATTTCTACTGTGCATTTCTACTGCGTATT
TCTACTGTGTAAGGTGTATTTCTACTGTGTAAGGTGTATTTCTACTGTGTACTGTGTATTTCTACTGTGTATTTCTACTGC
GTATTTCTACTGTGTAAGGTGTATTTCTACTGTGTAAGGTGTATTTCTACTGTGTACTGTGTATTTCTACTGTGTATTTCT
ACTGTGTGCTGTGTATTTCTACTGTGTGATGTGTGTTTCTACTGTCTATTTCTACTGTGTGATGTGTATTTCTACTGTATA
TTTCTACTGCGTATGTCTATTGTGTATTTCTACTGTGTGATGTGTATTTCTACTGTGTATTTTTACTGTGTGATGTGTGTT
TCGACTGTCTATTTCTACTGTGTGATGTGTATTTCTACTGTGTATTTCCACTCTGTATGTCTACTGTGTATTTCTACTGTG
TATTTCTACTGTGTCATGTGTATTTCTACTGTCTATTTCTACTGTGTGAGGTGTATTTCTGCTGTGTACTGTGTATTTCTA

CTGTGTATTTCGACTGTGTGCTGTGTATTTCTACTGTGTACTGTGTATTTCTACTGTGTGCTGTGTATTTCTACTGTTTAC
TGTGTATTTATACTGTGCATTTCCACTGTGTATTTCTACTGTGTTCTGTGTATTTCTACCGTGTTCTGTGTATTTCTACTG
TGTATTTCTACCGTGTTCTGTGTATTTCTACTGTCTATTTCTACTGTGTGATGTGTATTTCTACTGTCTATTTCTACTGTG
TGAGGTGTATTTCTGCTGTGTACTGTCTATTTCTACTGTGCATTTCTACTGTGTACTGTGTATTTCTACTGTGCATTTCTA
CTGTGTGCTGTGTGTTTCTACTGTCTATTTCTACTGTGTGCTGTGTATTTCTACTGTGCATTTCTACTGCGTATTTCTACT
GTGTAAGGTGTATTTCTACTGTGTACTGTGTATTTCTACTGTGCATTTCTACTGCGTATTTCTACTGTGTAAGGTGTATTT
CTACTGTGTAAGGTGTATTTCTACTGTGTACTGTGTATTTCTACTGTGTATTTCTACTGCGTATTTCTACTGTGTAAGGTG
TATTTCTACTGTGTAAGGTGTATTTCTACTGTGTACTGTGTATTTCTACTGTGTATTTCTACTGTGTGCTGTGTATTTCTA
CTGTGTACTGTGTATTTCTACTGTGTATTTCTACTGTGTGCTGTGTATTTCTACTGTGTACTGTGTATTTCTACTGTGTAT
TTCTACTGTGTTCTGTGTATTTCTGCCGTGTTCTGTGTATTTCTACTGTAGAAACAGTAGAAATACACAGTACACAGCAGA
AATACACCTCACACAGTAGAAATAGACAGTAGAAATACACATCACACAGTAGAAATAGACAGTAGAAATACACAGCACACA
GTAAAAATACACAGTAGAAATACACATCACACAGTAGAAATACACAGCACACAGTAGAAATGCACAGTAGAAATACACAGT
ACACAGTAAAAATACACAGCACACAGTCGAAATACACAGTAGAAATGCACAGTAGAAATACACAGTACACAGCAGAAATAC
ACCTCACACAGTAGAAATAGACAGTAGAAATACACATCACACAGTAGAAATACACAGTAGACATACAGAGTGGAAATACAC
AGTAGAAATACACATCACACAGTAGAAATAGACAGTCGAAACACACATCACATAGTAGAAATACACAGTACACAGTAGAAA
TACACAGCACACAGTAGAAATACAGAGTAGAAATGCACAGTAGAAATACACCTCACACAGTAGAAATAAACAGTAGACATA
CAGAGTGGAAATACACAGTAGAAATACACAGTACACAGTAGAAATTCCTAAACCTAAACCTCACACAGCAGAAATAGACAG
TCAAAACACACAGCACACAGTAGAAATACACAGTAGAAATACATAGCACACAGTAGAAACACACTGCACCCAATAGAAATA
CACAGTATAAATACAAAGAACACAATAGAAATGCACAGTAGAAATACACAGTACACAGTAGAACTACAATGAACACAGTAG
AAATACACAGCAGAAATACACTTCACACAGTAGAAATATACAGCAGAAATACACCTAACACAGTAGAAATACACAGCACAC
AGTAGAAATACACAGCAGAAATACACTTAACACAGTAGAAATACACAGCACACAGTAGAAACACACTGCACCCAATAGAAA
TACACAGTATAAATACAAATAACACAATAGAAATGCACAGTAGAAATACACTACAATGAACACAGTAGAAATACACAGCAC
ACAGCACACTGTCACTTTTACCAAAGTTATTATTTTGAAAATAAACCTTCACTTTTACTTAAGTATGACTTTTGCTCACTT
TTTGCAACACTGTGTTAAGTTATGGTTAAGACCATAAAAGAATGACCACTTCCTCTGTTGACTGCTGACCTCCTGCACTAG
TAACGATTAGACGCAGAAATTATACTGTGGCGCCCTCTAGTGCATTTGGGATGATGCACGCAGGGAGAAATATGACAGCAT
GTATTATTACTGTGAACCTGGATGATTTTAGCATTACTGTGTGAACATTAAAAACAACTAGAGAAGTCCTGAAGAACTGAA
TGGAACAGAAAAATAAGACATTATAATCTATAATGGCTGCAAATTGAATCTGCTTGGGGTTGAAGGAAGGAAGAAATAAAA
GGTTACTCACCGGTTCTATTTATTTGTTTATTTTTAATGAACTTTATTGAAAAACAATTAAATATACAAACACATCAGACA
TTTATAATGGAAGAGAAAAATAAGTAATTAAAATGACTAATAACTGCAAATTGAATCTGCCTGGGGTTTTATTCCCTCTAC
GTTACCGGATGTGACGTCGCTGCGGCCTCTTCCGGCGCGTCGGTAGCTGCTGCTGCTGCCTGTCAACATCACACAGCCCCA
GAAAACACCGGGATTTAGTTTTTAACTGTTTGTCTGTGACAGTCCGCTGGCTGTCCTCCACCATGCCCATGTACAAGGTAA
AGCCCATCGGCGGGAGTCTCACAGAGACCGACTTACCAACCTGCATGTACAAGTTACCCAATATCCATGCGCAGCGAGGGA
ACAGCTGCACCTCCGAGCACGCGCTTCAGGTAACTGATAACTGATTCATATCGATAAGGTTGATCGATTACCAGCTGCTCA
GCTCGTATTTGTACCTCGGTGTTGGCCGCAGCTCGTTTTATTCTCACGCAGTGACACGCGATGCCCGCACGCTCTGTTAGC
ATTAGCTAGCATTAGCCAAGCTAGCTGGCTTGCCAGTACAATGAGTAATCTGACTTCTGGCTGATGCAACGATTACTGCCC
GTCTCCACTCTTCCATCAACATTTAAAGATTATTAAACGACAGTGGGGAGACCATTAGATTAGCTTAACTCTTCCATTTGA
CGCACTTGCTGCCACTTTCTTTATTATTAAATAAACACATGAGACATGATAAGTTCATGTATTGATGGTATGAATGGACTG
AGTACACCCCTTCAGCTGTGCAGTGGTGGAATGTAATCAATTACATTTACTAAGTTATTTGACTAAGTAAATGTAATCAAT
TACATTTACTAAGTTATTTAACTAAGTTAATGTACTCAAATACAATAATAAAAGTACTTTAACTGAATATCTCCATGGAAT
ACCACTTAATACTTCAGCTCCAGAGGCATATACTGCATTCATATTTACACACACTACACAAATTAACTTAATACATGATGC
ACTGTTAAATTAGCTTAGAGCTTTTTGATAAAAGCTGAATTGTTTTGAGTCATTTTTTAAGGAAAAAAAAGTCAAAATTCT
TTGATTCCAGCTTCTTAAATCTGATCTATTTACTCCTATACCAGTAAACTAAATATGGGTTGAGGACAAAAACAAGACATT
TGAGGGGGTCATCTATGGCCTTGGGAAACACTGATTGACATTTATGACCATTCTCTTACTTTTATACACTAAACAACTAAT
GGATTACTCAATAAAATAATCAACAGATTAATCAAGAATGAAAATAATTGTTGCAGCCCAACGTTTGCTCCACCTTGTCCG
ACTACAAGAGTAAAATGCTAAAACACACAGTAAGCAGTATAACAGTTGGTCTGGGTGTGTCTATTCTGGTGTGACTCAGTT
GCACATGCACCTTTAAAATTGTCAAGTGCGACTAAACATTGGCCCCAAAAATGTAGCAGATTATTTTTCCTGTCTTGATTT
CTTGTTGCATGTGCTGTGATTTAAATGAAGAAAAACATGGATTTGCACCTTAATTCCTATCAACAATCATGAACATGCATG
TATTATTGTGCGCCCCTAAATGATGCGCTGGTGCACAACGTTAGGCACACCAGTGCTACCAGTGTTAAAAGTTGGTCTGGA
GCCCTGAAATGGCTTTTTACTGCATTTACTACATTTTGCTGATGTTGCAAACTTATCCTTGAGTAATGTTTTCAATGCAGG
ACGATTACTTGTAATGGAGTATTTCTGCAGGGTGATATTGCTACTAATACTGAAGTACCTGGATACTTCCTGCAACACTGC
ACCAGTGTTCCATTAAGTGTGGCCATTGTGACACTTAGTAGCATGTTACAAGACTATCTAAAGTCTAGTCTTGGATCCGTC
AAGTGTTAGTGTGTGTACTGTGAGTTTATCTCAAGATTGGGAATATGCTAAATATATTCTCAGTACTGTTTCAGCTTCATC
TGTGTAAGGTCTGTTAAATGTAGAGACACTGGCTTACACCCACATTGTTTCAGGATTGTGCTCAAACTGTTAAATGCTCAC
ACTGTCAAATGCCCATTTCAGCCAATCTGAGCATTTCTGTCTCTCGCCTCCCCAGAATGGCGAGGTGGACCCGGCTGTCAG
AGCTCTGGAGGCCCGGCAGGATGAGATCATGAGAAAACTGTACGAGCTGAAAGCAGCTGTGGAGGGCCTGGCCAAGACGGT

GACCACCCCAGATGCCGACCTGGACCTGACAGTCAGCAGCAGCCTCTCCTCACAAAGCTGCAGCTCCACGACCTTCAAAGG
CGTCACGGACCTGGACACAGTACTGGGAAAGGTGAGAAACTGGGAATAACACTGGTGTGCTCTGGAGACAGTACGAGTCTG
TTTCATGAGACAACTTTGACTGCGTTTGATTTGATCCTTTCCTGATCACACCCATCATAACAAGTTATAAACACAAAGGCG
CACAACCTGTTCATACTGGAATTTGTCTTGAAGCACATATGGAGGTGTGGACTGATAACAGCCTGTTTGTGCAGTCTGTAT
TGGCCTTGTTGAAAGTGTTGTAATACTTTATTTATTCATCTCTTTTGTGTGACAAGGACCTCGGAGCTCTCCGTGACATCG
TCATAAACGCCAACCCGGCACAGCCCCCCCTGTCCCTGCTGGTGCTCCACAGCCTGCTGTGCCAGCGCTACCGGGTGCTCT
CCACCGTCCATGTCCACTCTTCCGTTACCAGTGTGCCGCCACAGCTCCAGTCCTGCCTCGGCCCTCGCCATGCCGACAGCT
ACGCACGCCAGATGTTCCAGCTGGGCTTCACCCTCATATGGAAAGATGGTAAGACAGAGTTACAGAGGCAGTGTGTGTGAG
GCGATGGACAGAAGACGAGGAGTGTAGGAGTTTTATACGCTGGTGCCACGCGGCCCTGATCCACACGTTGCTGTTTAATTT
GGATGTAATTTCTTGTGAAAGTGTGTAGGTGGTTTGTTACTTGCTTTTTCTGAGCTTAAAAACTAGTTAGAATACCTTTCA
CCCTGCCAAAGTAATCACGTCAAACAATAAGTTAAAAACAAACTGGAATCATTTGAGAAGGGGCTTGACTCTTTTACTGTC
AGTGTCAAGAGGCTTTTTTGAATAAAGTAATTTAATTATAAAGAAGAGAGTGTTGCAACCGTAAACACTCTGAATAACAAG
CAGTTTCTGGGTGTTTTTGCGCTCGTCACATTTTTACTTACGGTGGGCTTGTTTTTCACCGCAGTTAGTAAGTCCTGCATC
TCTTTCAGGTGTTTACAAGTCGATCGTGGTTCATTTTTATCGTCAAAATTGTGAATATCCTCACATGAATGACTTGAAAAG
CTGTCTAGCTCCCTGTCGTACAGTCCTATATTGGTTTCCTGATTTTGAGTCGCCTAAGCCTGTGGCCGTGGCAATATTTTT
AAAGAAAAATACGCCTTTTAAACCCTGTGGTAAGTTTTGGAGTTCAGAATCGTCATGTACAAAGACTCATTGTGTTTTAGG
AGAGCTATTTCTAATCATTCCGTGCTTTGTGATCATGTTATCCAGATGCTGTACATTTTTTTAGCTCTTGTTTACCGTGAG
ACACTATCCTTCATCCACAGTTCCCAAACTGCAGATGAAGTTCAGCGTCCAGAACATGTGTCCCATCGAGGGTGAGGCCAA
TGTGGCGCGCTTCCTCTTCAAGCTGCTGGCTCCCTACCCCAGTGACCCTGCCCTCGCTACACTGGTGGACAGCTGGGTGGA
CACAGCTTTCTTCCAGCTGGCAGAGGGCAGCGCCAAGGAGCGCTCAGCCGTCCTGCGGGCACTCAACTCCGCTCTGGGCCG
CAACCCCTGGCTGGCCGGGCCGGAGTTCTCCCTGGCTGACATCGCCTCCTACTGCTGCGTGCTGCAGAGCGGCTCGGCCTC
CGCTGCTCCCGCTAATGTGCAGCGCTGGATGAAATCCTGTGAGAACCTCGGCCACTTCAGTCCTGCCAAGCTGCTTCTGCA
GTGACCGGGGCCGTCCTGGGACCAGACGCCAGAAGGGCGTCTTACCCAAGCAAAGTGAGAGAAGGGCATTAGAATATTAAA
GTACACCAGTCATGTCTGGAACTCCCTCTAATGCTTCCCTCCTTACTACACTATATCACTGAACTGACAAAGACACAGTTG
AAGCAACATGGTTGACGGTTAAAGTAGCTGTGATATTTGTGCCAGCCATGCTGCTTTTGTATAATAAAGAGATTGTAACGC
TATTTTATGCAAGGAAGGAAGCATGAGAGGACTGTAAAGACATGGGCTTGGTGGTGGATTAAATCAAAGGTACTGACAAAG
GCTGTTAGTCCAACACACACTGTACACCTTCCACCCACTTTGTGCCTAACATGATCCTGCTCTAAACGTGTGTGTTAATTT
GTGGTAGCGGGTATTACGGGCAGTTTAAAAAAGTAATACTTTGTACCATTTGTTTTTCTGAAATCATTAAAACTTTGGATC
AAACTTTGAACTGGGCCTTGGGTTTGTTTCATTCAACTGTTTACTTTAAAAAACAAATATCCTCATCCAACTGAACAAGTG
GCATTTAATGACGTGCACCTACAGGTATGTGTCCAACAGGTGGCAGGCTTGTCATTCATAAAACTTCTTCTTTCACTAGCT
GATCCTATCCAGAGTCCCAAACACTAGTCAGGTGATTCTTCCATGACAAGGTTTAACATACTACAAGATACTTTTAACTAG
TTGTGAAACAGTTAAGTTTAATCCCTCTACAAAACCTACACAAGCAAGCTAGACCATCGGCAAAAAGATTGGCTATTTCTT
TAGGTATTGCAGTTAGTCCTGATGCCGAATAAATCGCACAATAAAAACAAGTTTTACAGCAGAGTGCTGAGGATGAATGGA
AGAGTCGCAGCGTTAGGAAAGAAGCTGCTCTGCTGTCTGCTGGTAAAGTGCTAAAACAAAGTTAACTTTGTTTGCTTTGTC
CTCGGGGCTCCCTGAATCAACAAAAACTGAAAGTTCTTTGAAATAACTGAAGAACTCTCATATTGGGGAAAATCTCTACTT
CACCTGGCTTCAAAGAGTCCTCATTCAGACCCTCTGGCTTCTGTTTTTCATTTATATATTGAAAACACTCTTCTTTTTAAG
AGCAGGTCTGGGTCTCCTCTCTGAGGTGGAAGATCAACAGGACAGTCATTACATCTTATTGCTCTTGTGTATTCACTCATG
CATACCTTTAGCCATGCAGGCAGCATAAGAAGTACACTGTAGATGAAGCACAGGCGATGGCTGAAATTTTTTTTCTGGTAT
GTGATTGGTTGAATTAGTACAATTTGGAGCCACCTTAAAACAGTTTTTTAAAATCAAGATCCATGTATTGCTCCCTGAGAA
ATTAACAGAAATGTCAAAAAAACACTATCTTGCAATGTTAAAGAAACAAATGACTAAATATTCCTGGATCTGTCAGTTTTC
CTGGAAAAATTAATGGGGTCTATTCTGGGTCGAGACCCACCCTCCATCCGACTTTTGTGGAAATCTGTTCAATAGTTTTCG
TGTAATCCTGCCTACAAACCAACCAACCAACCAACCTCTTGGTGGACGTAAAAAGTTATTTTTCATCTAACATTTAATAGT
CACCCTTTCTTTACTCAACATGGAAGCAATTATTGTCCCAAATCCTGCATAAAAGCACATATTTCCCTTATGTTCTGATGG
AAACAGATTACAGACTAATATTTTTCTAAGTATAAGATAGAAAACTAATGAAAAGCCGGCTTCTAATGTACTTTCGTCTTC
TAGCACTTAGTGACCTATTTTTAAGAGCATGTAATGTAATGATTGTAGCTTTCACTGAACTGCACTTCCCAAATTCACTGT
CAGAGCTTCACTCCCCAGACCTGTTCTGCACTGTACAGCTTTCACTTAAAATTGCAGCTCAGTTTGAAACTGGTCGAGCAG
CTTCGTTGTCTTCTATCGTGAGGTGACAATTCTATTTCCTGTGGCGATCCTTCCTGTGTTCTCTCTCAGTTGTTCAAGCTG
CGTTTTGTCCTTATCAGCTGATGTTTAGCTGAATAGAATCCCAACAGCCTGTCTGTCAAGCAGAATAGATTCTGGAGCAGT
GAGGGCAGTTTCACAGCAGTCCACTGGTGGAGAGGTTTTTTTTTCTTCTTATCTCTGTCTGTCTTTTCAAGCTGCTGCGGG
AAAATTCATAGTAAAAAAAACAAGAATATATACAGAGTCATACATGCAATAATGTTGAAAAGAAGAAAAGGCCAATCCCAG
GGTGCCTCACACTCTCTCGGGGGTTGGTTTTGCATCTTCAGAAGTGTCATGTTTTCGACAGCTCGGGTCCAGACAGAGCCT
GTGTATGAATGTGTGTGTTTGTATGCTCTGACAGCTTCCTGTTGGTCTCCAGGCAGCTGCCTGACAAGACGAAAGCTCTTA
ACTGACATCTGGACGCGTGGCGAGGAAAACACTTGGGACTCTGCAGCTCTGGAAATGTGGTGTTTGTGGTGCTGTGTGTCT
TCCTCACACACAGGCACACACACAGGCCTCTTTGAAACGGTTATCAGTATCTGTTGATGCAGTCTGGGTTGCATGCAAATA
AAGCAACCGTTCCCACACCAACACTAATCTCATTGCTTTCGGTTTCCGCCTGGTTTGCATAAAAAGGTCAGACACAGACAC

AAAAAGGCTCTTTCCGTATCTCTTTATATATGATATAAAACATACAGACAGGAGATGCATTCTCATCGAAGAGCTGTAGAA
AAAACAAACTAGCTTAACTGAAAGACAAAATCACTTCAGTTGAACCCTGTGGCTACTTCAAATCACAACAATAGCACAGAT
AGAAAGATGCTTTCTTCAGTATGTTCAGTCCTTGAATGCCATTGTCCTTTGATTGTCAATTTGATATTTCCAGTAGGCCAT
TCAGGAGTGGAAGATATGCTATCAAAACAGGTCAGAGAGGCGTATTACAAAACAGAAAACACATCCGGGCTAAATTGTCTG
AAACGAGATCTTACTCAGGGTTTTTCTGTAATGATTTTTAGCACAATATGGTTTGATTCTGCATCGATTAAAAGGCTGAAA
GACAGAGAGAGTCACAGGGAATGTTTTCACTCAGTGACCAAAGTGCACACGTTGCTGTTGTATCTCAACAGCTGAATGCGT
TCGAAGTAATCGTCGGTCAGAAAGTTTCTCTGCGAGGTGACCAGGTTGCCTTTCTGACTGAAGAGACGCTGGACTGGAGCC
GTTGATGGCAGCGTGGTGTTGTATTTGAGGAAAAGCTGCTTCACTCTTGGGAAGTCCTGCAGGCACTCGAGGCTCTTCCCC
GTTCCTTCAACGTACTTGCGGATCTCCTCCATCACTCCCCGCTGCTGGATCTGGATGGCGGGCTTGACGGGCCCGTAGCTG
AAGAAGTCGTCTTCGGATTCGATGGTCGACAAACTGCGGCTGGGGTTGGTCTCCGTCACGTTGCAGGGATCCACCTGGGAC
GCCTCTGTGGCCAGCAGGGTGCACATCTCCTCCCTCTCGGAGGCACCCATCCACCACAAGCGAAACTGAGGAGTGGTCGCC
GTTGCGATCTTCGCCTCGGTGCTGGCAAACAGCTCCTGGAACCGCACGTCGATAGCCCCTACCATGGCGTTGATGACCTCG
CTGAAGTAATTTGCCGCATCTTTCTGCTCACTTAACTTGTTCTTCAGGCTGAGAACAGTCGGGATGACCAGCCCGAGGTAA
CATTTCTGCTCCGCTTGGAAGAGTTCAAGTGCAAACGCGAGCGGGTGGAACACAGTCACGTACTCCTTTAGGAAGGCCATC
TCCTCGGGCTGCATGCGCGGGACCTCGAGGCGGGCGCACAGCTCCGTCAGCTCCCGCTCGGTGAGAGAGACGATCTTCTGC
ACAGCACAGTACTCCACGTTCCATCGTATGACAGCGGGGACGACAAGCGCCATCTTTCCTATCTCCTCCGCCGCATCCATA
CCGACCTGAAGGTGGTGGCATTTGCTCCATATGGCGTACACCTTGGCCATGGCACTGTAATGTAGCTGGCACATGGGCCCC
TGCGACACCGCCTGCCAGAAGTCCTCAGAGACAACGAGCTCCAGGGTGTGAGAAGCACAGCGCTGCACCGTGGGCAGAAAC
AGGAGCATGTCCTGCTCCGGCTCGCCCTCCAGAACAGCGCTAACGTTCTCGTAGAAGCCAATGTCATCGTCGCTTTCCGAG
CTGTCCACCGCAAACTCTTTAAACACGCTCACGAAGGGGCTGCCGTTGTCGGTGACCGTGGTCTGCACTTTGCTTTCGATA
TTGTACGCCACGTGGATGTCGTGTATCCGTCCGGCTATGGTGTCGTATGTGATGCGGCCCTGCAGCCGCGCGAAGCCCAGA
GCAGCAGATTTCCTCTCCAAAGAATCCTGGTCGATCCAGTGACAGGTCATCCCGAAGAAGCTTCTGTTGTTGGCTGTCCAG
ACGTCAGCCGTGGTGCACACGTACTGGACGGTGCTTAGTTTCGACATCAGCTCTTCCCGCATCCTGGAAAAACCCTGGTCC
ACCTTTGTAAACAAGGTCACCCTGTCCATGGACTTTAACCCTTCAGTCAAGCCTGCGATCAGCTTCCTGAAGCCAGGTTGC
TCTAGCAGATAAAAAGACTGACAATCCTCCACGATGAAGTTGAAAATTAGATCATCAGTCTTTGATTGGGTCATGCACTTG
GAGATGATTTCAGCTTTAAGTTTTTTGAGCTGGCAGTGCCGGCTCTCTTCGCCGTTGTGCTCTTCTTTCTTCCTTCCCCTC
TTGGCATCAGGCCTGGCTTCACAGCCCAAATGTGTTCTCTGGAAGACAAGAAAAGCCCACAGATACAGTTTCTGGTTACTT
GTTGGAGGTGTGCCATTCGTGTGTGCTGAGTTTGTAACAGTTGAAAAACTGAAACTTGCAATATAAAAAAATCCACGAGAG
CACTTTAAAACACACATGCAGAGTGAAATGAGTTGCTGGATGAAGAGCCTGCAGCGCCCTGAGTTAATAAGCATGCAGCTA
TAATGTAACGTTATTAATCACGAGGGCAGCTATTCATTCATTGACTCTTTCAAACATTAACCCGGCGGGTACTGCAGATCA
TTACAGGGAGATTACTTTGTGAAACTTACGTCTAAATGCTTCTTTAGGTTTGAGGTTGAGGTTTTCGACGTGGAGAGCAGA
TTAACCCTCGGCAGACATAGATTACACTGGACGATGATATTTTTCCCTTGATCTGCCTTGTACGTGAAATGGTGGCGATAA
CGCCAAGTATGCATGGCCGATTTAGTCTCGCCGCCGGGTTCACTGTGATCCACATCCATTTCGGACTCTATCTTTACTGTT
GCCGACGTCTTGAATTATAATAAAGTATCGTAATCCGATTTAGTAATCGTGATTACACGTAAAGTAACGATGGCGGTAGAG
AAATCCTCATGAAAAAGGGGCAGGCCTTTGTTCGGATGTTGCGCAGAAAACAAACAAGGAGCGGACACCGTCGTCGCTGTT
TGCGCTCGCGCACGCGCGTCGGCGGTCCGCAACTCCACGGTGAATGCGCGCAGTGCGCATGCGTCGTAAGCACACGGGCGC
CCGCACCGTGACGTCACTGCCCTCTCCTTGGGATTCTTATCCACATTAATGGAAGTACTTTTACCAGCCAGCATTTCCTTT
TCCTACACTTGATGCAGCGAGTTGGCGCCACCTTCAGGTCCGCGGAGGTAAAAATATCGCTGCTTTTTAATTGATGAATTA
TAAAGAAAACATTTAGTCCGTTACTACTTATAATACAAGTGACATCTTGGCTGCAATTACTGTAAATAGCTGTGCAAATAA
AAGCGGATTAAAAACAGAAACAAAGACAGAAATATCAGAGTACTGATTTAGATAATCCAGATAGGCTAATAATAATTATAT
TGATATGGCACCTTCAAATTAAGAGTTTACAAAGTCCTTTGACAGACAAAGCAAAAGCAGGTAACTCAGGGAGACAATGTA
ACAGAATAAAAAACTGAAGAGTCAAGCCACATAGGAAGAAAGAAGAAAAACTTTCCCAGCTTCAGTGGCGGACTCAAGATG
TTTGAGGGGCGGGGGCGAAAAAATGAAAAGGGCACCACAAGTGCAAGGGGTGGCTCCAGCAGAAAGCTGTGAGAGATTTAT
AGTGAAGAGAGGTCACTTCACCACGTTGTGTCCAGTGGAGAGGCACCCTACCTTACAGCTCAGACTACTTTAGTACACATA
ATATGACATTTTATTAGTATAGCATCAGGGAATGTATCCCAGCTTTGCTTTATCCTGAGGGCATTTTATGTTTTGTAGCTC
TAAATGAAAAGTGAAAGTTTAAGACAGCTAATCTGAGTTGAAGTGTGGCTGTCTGAATGCTGTCACTCTCTTATCTCAGGT
GGTTCAGCTTCTTTGAAGCAGTCATCTATTGCTTTTACAGGTCTTTCTATAAATTTGAAGCATACCCGCTAATTTTACAAA
CACACAATCGCCACTTTGCATTCTAAGATGAGCTTTATGGTGGAATTAACCAGTTTATTGGCCAGATTTTTGGCAGCACCA
TCGCGGGGTGTTTGTTTTCTTCTTCTTTCTTTTTTTAAAATCACGCCTGAGGCTCCTTGCCAGTGGAAGGCATCTCCCGCT
GGTTTGAAAAGCCCTCACTCTGGCTGAGAGGTGCATTTAGCATGCCTGTAATCTGCTGCTCACATGGGCTTGAATGGCAAT
TTCTTTGTGCATGTTTAATAAACCAATTTGTCAGCTGTGTGATCAAAGTGAGGCAGGTGAGGAAAGAGTTTATCTATCTAT
CTATCTATCTATCTATCTATCTATCTATCTATCTATCTATCTATCTATCTATCTGTCTGTCTGTCTGTCTGTCTGTCTATC
TATCATGGGAAATCCAATCAACCTGGCTGATTTACTTCGTGCTTGCTGGTATAACGCACTCACCCCGCATGTAATTGCTTT
TTTAATGCTTTAATGCAGGGAATTGGTTGTTTGGCGTTAGTGCATCACAGAGAAGGACCATTGTGTCAAATTGTCTCATTA
TTTCTCTCCACCACCACCATCCTTCCATCGTCTCCCTTGTGACTGTGAGTTTGGTCATAATCCTCGCTGTCGAGTTTAGTG

GAGAGTGTCCAAACGTCTCACACGAGACACATATTGTTTGGCTAATTGATATTTCCCTAAAGATGGACTACAAATGCATCA
GTGACCCAATTGAAAATTGTAATGTCACAGCACAGAGGTTTTTTTTCTTCCTTTCTTTATTCAACGGCACACCTCCTCCAT
TGGTTAGATTTGGTGAGGGTGGGGTCTCACTTAAGAGCCACTGACGTAGCCGACTTTGGTTTTAACCGAGGCGCAGTCTGT
GTAGGAACAGAGAAAAGTGTTTTGAGACTAAGATAAAGAGGCGAAACAGAGGAATGAAGTGGTGAGAGGGGGTTTAAAGTG
TCCTGATTTGGTACACGATGTTGATGTCGTGCTGTCCTGCGCGCTTCGAGACTGTGTGGAGCGTCTGGAGTGGGCACAACT
GGTGTCCGCTCTCATCCACAGCAGCAAACAGTTGGTTCTGAGCATCAGGTGATCGTCTCAGACAACATTCCCACTTCTTCT
TCTTGTTCTTCTTCTTTCTCTGGGTGGACACTTTTTAGCAGATGAGCAGAGAGAGTGGACGCTCCTGAACAAGTATGACCA
AAGGATCCGCGCCCTGACGCGCCTCGGTGAGTGCATGTTGGACCGGTGAGAGTGACTGTGGACTAGTATGAAGCGCCATGG
CTTGGTGTGACCGCGGGGTTCAAACGCTGCTGGCCACCGTGGGGGCTTTCGCTGCCTTCAGCCTTATGACCATCGCCATCG
GCACGGACTACTGGCTTTATTCGCGGGCGTACATCTGCAACAGCACCAACGCCACCACGGACGAGACCCAACCGCAGCCCA
AAACCAAGAAGGGCGACCTCACCCACTCCGGGCTGTGGAGAATCTGCTGTATTGAAGGTAAGCCTTACACAATGTTCTGCA
TGCTAATCTAACACATGCTGCACGATTTCCTGCTCCTTTCTACAGCAAGTAGTTTAAATTCTCTCTTTTTTCCGAGGAGGA
GTCTTTTTTTCCTCAGTGCGCACAGACACAAGGACGCACTCCTTTTACGCACGGCAAAATGTCTCAGTTGGGAGATGGGGA
CACAACAAAAAATGATCATAAAGGTGGGAAAACGTTCAGAGGTTATAGGTCAGATGGAGTGGGCGCACACTTGCGGATTTT
AACGAGCTGAAGTCGCAGATTTCCGACAGAAAAGCGTCCCGGCGCTAAACCCGTCTGCCAACTGTTACATCGCATTGTTGG
AGCACAGGCCCGCACAGTAAACACGACTGTTATAGTCTGCGAGAGACGGCGATGCCAAGCCGGCGCGTCTGAGCCCATTCA
CTTTTAAGCCGGTCGCTGCCAGGAAATGTGCGCGAGTCTACTTTCATTCATGTCTTCGTTGTATCCGATGCTGTTGCTTGG
CAACATCCCTGACATTGCTATGGAAACATGCCCAGTGATATAAAAATAGATGGCTCAGAGTTCACGTCATGTCCAAGCAAA
ATAGAAGAAAGTACAGTTTGAGTTAAAGAACATGAGGTGAACATCCCATAATTTATCTTTTCATCCTTTTGAAAGTGAATT
TTTGGATGTTTGGAGAGGAGGTGCGCTGGCAGTGACGTCTTACTTCAGAGGCAGACAGGCGCTCATTCATTGACCAATCTT
CCTTCCGGCCACCCCTTGACTTCTCGCCTCCGCTTTCAATGAATGAGTCGATGTTACTGGATGGATTTGTTCATGTCACAA
ACGTCCGTCGTCATGAGACAGAAATAGATCCGCAAATAAACGTCCTGCTGAAGGAAACAAACGAGATAAAACTCAGGTATC
TTATTTTGAAAGCACACCACAACTTACTAAATGTATCGTCAGCTGCATATGCAGTGCACATAACACAATCCTACACATAGT
CTACTCTGTCTGAATTTAAAATGTGCAAGCAAATTTGTTTTCAAAACAAGTTGCTCACTCAACAAAATTTCTTTACAGGAG
CACTTTGTAGTTTTTTGGAGAAGAAATTCAAACTCAGAATTTGAATATTTAAAATATTAATGGGGTAACTTCTTTTTTCCA
TAACTGAATAAACAAGAAGTTAGAAAGGTGGCAGGGTCCGCCATATATAAACAAAGTAAAACAGTATGAAATGATGTTGTC
CTTTAAGGTCAGTTTGTTTATTCAGTTAATTCAGTCATGAAAACGAAGAGTTTGTTTATTTATTTAAAAAAAAATCAGTCA
ATCAATATCTTTTCCCTCTGATTAAAATGTATTCCCCAAAACTACATAGTGCCCCTTTAACTTTAGCAAATTGCAGAATTA
AGAAGGATTTTGGCCAAATCTACAGACAGGAGGAGGTGCACTCCCTAAACACAGCACATGAAACGTGACACATTTTTAGCG
TCATTTCTGGAGCATTATTACAGAAAAACGTGCAGTATTTGTTGTGAGATGGAAACTTTTTGTTTGGTCACCTTCAAACCC
AAGAAGAATCTACTCTAAAATGTTAGTTTTTATCATCTTTTGTGTTGATACATTAAGACTTTTTATTGATATCCAGCCATT
TTTTATCTATAAAAAGGATGTTTTGCTTCTTCTCCCTAAAAAGCAGCTGAATATTTTACATGTTGTCCCCCAAAAATGAAA
AAAGGAAACATTTTGCCTGGACACTAGCTACTTTAATTGCCCTTGTCTTGGTGATGGATCATGTGCTTTTCCATTATTTGC
ATTTTATTCTGAAGATTGTTTGGTTTCCAACCACACTTGCAATACTTAATGTCACTAGAGGCCACATACAGCTGTCATCAA
TGATTAAACTGTCTCCGGGCTCATCCTGCGGTGATGATGGACGCTTTAGACACATGTGAAGAACATCCTGGCGTGTTATGA
CACTTGCAATTTACCGAGAGTCATGCATGGCTGGGCACCTGTCTGTGATGTGGACAGACTCACAGTCTGCGTTGCATCCTC
TGAGACCCGGAGAAATGAAAATAAATCACAAAAGCCTGCCAACTATTATGTCGAGCTTTGAAGTAAGTAATGGGAGTGGAT
TAATGACGGTAGAGACAAAGACCGGCTCTGTGCGATGACGCTTCCATTTTCAAAAGCAAGAATCTGACAAATAAAAGCCCG
GCCTATCATATCACTCTCTCCTGTGCTAACAGTTTTACTTCTGCGCCTCTGCTGAAACTACAAGATGCTGGACAGTGGCTC
CGTTGTCATGGAGACTCATAAGTATAGAGAAGACAAAGAAGGAAGACTCAACCCCCTCTCTTCTGACACACACACCCCTCT
GGTCATAATGACAAACACACTGTGTTATTGACGGGGCCGACTCTGTGTGTATCACAGCTACTGTGGAGAACATCACCACGT
TTGGCTGAACAGATTGCAGCGACAACACGTTGCACAGTTTCTTCTCAGAAGCCTCCAGTGAGCTTGTTTCAGTGTTTCCAG
TAGCTCGGCCCAGCAAGCGTTACCCGCATGGCATTTCAGGAATATACTGAATGAGGTCATCCACGAAGTCCTTTGGATTTC
TCAAGAGTTGTTTCGATTTTTTCTCCCCTCCTTTGTTTGTTTTCTCACATGAATGCCGCGAGGCCAGGGTAGCACTGAGTG
GCGCCTAATTTGCATCAGAATCGGCGATGCATCAGAGGCTGTCGCTTACGTGCGACTCGAGTAAATAATACCAACCCTGAC
AATGAGAATTGACCTAATGACTAATCCAATCAGCTGGTCCATATTTACAGTAAACATCCCAGCTGGAGGTTGTGTGAGTGG
CGAGGCAGCTGTTTTTCTAAGGGGGACAGTGAGGAGGCCTTTTATTTAGGGTTGCTTTGCCGTTCCCATCGTGCACCGCAA
GTCTGTAATTAAAACGCGTCAACTGAGAAGCCGTGCAGGGGCACTAATTAGCCAGCGGGCCCTCACTTCCACCTCAGTCAC
TATGTCCTCCTCTCTGCTTGATGTGCTCTCTCTTTTTAACTCTTTATTTGTTTATCTCCTCCATTCGCATCATCCTTTTAT
CTGTTTTGTCTTCTGTTATTCTACTTTTTGAGTTAACCCTCTGTCACCCCCTTCATACCCACCAACCCTCAGTCCCTTTTA
CTTTAGGATGCATTTCTCCTCTCGTTTAAATTGAGTTCCTTGTGTCATGCTGTTTGAAAATAGGTAACCCCAAACGAAGTT
TGAAATTATGGGAAACTGACTTTCTTGCATAGAGTAAAGTGAGAAGATTGATGCCACTTTAATGTTGACATGCTAAGCAGA
GCCGGACTCAGGTTGTTTGAAGGACAGGGGCCCACTTTGGTTTTTGTATGAATTTTACAAATAAAGTTTTGTGTGTTAATT
AGTGAGCTTTGGAGGTGATAGTAGATGGATTTTGTTGGCTTTGGATGGAGATAATCAAGATAGCTGTTGTGGTTGAGTGGG
CTTTGTATAGGACTCTCAGTCAAGAGACTGGGGAGCAAATCTTACATGGAACATAATATTATACCAATTTAACATATTATT

TTCAATTTCTTTACAGTTTTCTGTCGCTGTTTGGTTAGGTTTAGGGAGCTAAAATACTTGGTTAGGTTTAGGAAAACAGGG
TTTGGGTTAAAATACACCCCTTCACAACGGTAAACAGGTGTGTGAAAGGATAACTGTATTTTTCTGGTCGTTTCCCTGAGT
GTCAGTGATGGCCCTGGAACAACATTTACTGTGATTGTGATGGAACAACACCAATATGGTGATATCAGACCAAGCGTAGCT
TCATATTCACAGATATAAGAATGATAATCTGCTAATCTCTCGGCACAAAGGAGAATAAGTGTATTCCCGAAAAAAACTAAA
CTTTTCTATAAAACAAGCTTTTTAACATCGAGAAGTAGCAGGTCTTCTGTTGTTTAATTAACAAAACTTTACAATCCTTAA
CACTTGTCAGCCTTTGAGGTCATGGAAGACGACGACAAGAAAATTAGCCACCTTCAGTAATAGAAAGTTTAATTGAATATA
TATACACACTCAAAGAATAAAATCTTAAGCATTGCGTTGAGAAGCCATTATATATGTACATTTATAAGTAAGTGACAATGC
ATAACAGACTTCACAACAGACTACATGAGGCTACAGATCTCAGTTTTTCTTCTAACAACGTGGTTACACATGCACAGTCTA
CCCCAAGCAGGGCTGCGTTTGAAGAAACTGCTGCCTGGTGACAATTTAATTAACCAACTGTCAGTCATATCAACACTGAAT
CACTGATGTCTTTGCCAAATTGCTTTTCTTTCATCCTCACTTGGACCGGGGAAAAAAAAAACTGGAGGGGTTTGTTTGATT
ACACAGATGCTTTTATATTCTGCCCCGGCATTCCCTGCTCGCTTCCAAACTAATAAATGTATGTGTCTGTGCGAGCTGTGT
GTGGATGTCCGCATTTGATATCGAGGCCCGGCAACAGTGCTCTATCTGTTTACAGCTTCATAAAACCTAATCATCCCACCT
GCTGTAAACCTCAGAGCGCTCTCCCGTCGGTGTTCACACACTCTCCATCGCAGATCTGAAAGCAAGGCAGACTCCAGGTTT
GCTGTGCGTCTGTTATGTGAAGCTTTACTGAAAATACATCACCTGCTGGAGGCTACGTAGTTCTGTGAAATGCAATACTAA
AGTCACTTTTGATTGGCTTAATCATCAATCAATTTGCCGAGCTATTGAGGTGTGAAAATGAGGAAGTGGGGGAGCATGGAG
CTCGTTGTTGTGATGTACCTCACTCCCGGAGAGCGACGACTCAATGAGACAGTGCTATTGGTGTATTCCACTGGGGCCGCT
CCCTTATTGGCCGAGCAGCGCTAATTCAAAATAATGACAACGTGCATCATCCTGTTGCTAGAGGCATCACAGCTGTGACAG
CTGCTCCTGATACACGGTGTGCGTGTATGTTTGTATGTTTTGTGTCTGTGTGCATGTGTCCATTTGTGTGTGGCAGTCGTG
TTCTGTTAAGTGGTGACAGATCTGTGACGGGGAACAGGCTTTGTTCTGTGCCGTCTCTTATTTGGTGAGTTTCAAATTACA
ATGAAGATGTCAAAAGCAACCTTGGAGAGTGTTTATGTAAATGAGCTTATTAAAGACTAACAGGGGTTGCCATGGAGAAGA
GGAGGAGAGGCTCAGCATGGGTCTTTGCATCTCCAGTCTAGGCCAGGAAATCCTTTATTTCCTGTGTTATGTGTTGACAAT
TATTTCTCAATTTGTCACAACACAATCACCTTGTAACATCCCCCACATTTGTGGATAAGAGCTTTTTTATTTGTTACCAAC
ACTGAGGTGACCAACTATATGTCTATCCAGTCCAGTATTATGACAGTTTGTGATACAGCGACAAAATGTATCTACAGGATC
ATGTACATTGACACAGATCAGTTAAATTTCTAATTACATATAACCTAAATTATGATCTTTCCCCAACCTTAAAGCTACAAT
ATGTAAAAAAAAAAATTTAAAAAAAAGTTTAAAACACTCTTAGTCAACTTAGTTAAAATGTGTTTCTATGGCACATCTAAA
ACCAACGTGAGTTCACCAAAGTGCTTGACAAACATGGACAAAACAAAATAAGGTCATCAAAAGATCATGCAGAGTTCAAGA
TAAGCTCCCCGAGCAAAGTAGTTATAATCAGTGATGGATTCAGGATGTTTCAGGGGCAGAGTATTGATATATTTATGTGAA
GAGAGGGCACTTCACCACATCTTCTCCACTGTAAGGGCACCTGAGAGGGCACTTTATCATGTTTTATCTACCGTAGTGGCA
TCCAAGAGGGCACTTTCGCTGCGTTTTTTCGAACATGGGGGCACTGGTGGCTATAATTATCACAGAATTATCGTAAAGATA
TCTACTGAAGTGAGCATGCAAACCAGCTTGTCCCGGCCCAATGAGGTGATTCCAGCGGTGGGCCCTGACTGATGTCCGACC
TCCCTCGGAACCACTGTGGATCAGCTTACAACTGTATTCCTTGTCATCAAACTGGTTTCTGTTGGTCTTGGAAGCTGTGTT
TGGTTACAATCTGGCCATGTTCACTGAGAGGCCGCTGATCCCGTTTCTGCTGCCCACTTGCCCGGCTCCAGTTCTGGTGCT
CTTTCTGGTGGTGCTCCCCAAAAGCTGTGGCTCCATACTGCACGGCTAACTGAGCTAACAAGCTAATGACAGCTACCGTCA
GCAGCAGTTAACAGTTACTCTGGTGATATACTGCCGACAGGTGGCCAATTCTTACATATTGCACCTTTAACCAAAGTAAAT
CCTTGGGTTGAGCGTCTATTGGCTCAAACGAGTATCTGGTTTAGCTAATGTATTTTTTTTTCGAGTACAAATTTCATCGGG
TAGCTGCCTTCCTTCATGCTTATACAATAGTTGTTCTGTAAATAGTTCTCTTACTCTTGTGATCAAAAACATTGATCTGTG
GCTCAATTCTGAGGCATTTTCTAAACATTTTTCTGATAAATGATAAATATAACAACTTCTGATCAGCCTATAAAACTGCCA
GGCTTAGACGAACAACTAGTCGTGCCCATTTTTGGGTTATTTCCCATATGGAAATTAACTGCAAATGTGTTTGCCTAAACT
TAACCAAATTCCTCCCTGGGACTCCACCGCGGTTCACAAATGTATAGTTTCATTCAGTCGAAGAAACAGTGCAGGCACGAA
AGACAGTCATGCCGAGAGTCATGAAAAAAATTTGAAATCCGTGTCCATGTGAAAGAATCCTGTAGATTTACAATACGACAC
ACTGCCGTGACTATTTTGTTCTGTCTGGCCAACGTTTTGTCTCTACATGTCCCTGCAGTTTCACAGCTTTTACATTGCACA
GCCGTCCTCCGACCTTTGTTTCACAATCCACATTTTGCCCTTTAACACTCACTCCGCCTTCCCTGATTTCTCGCTTGCTGA
TTCCCTGCTCTCCTCCTCTGTCTGAGTTCCTCTTTAATGTGGCCTCCTATCTTTTTTCATCCCCATCATTCCTCTGCTGCC
TGTGCTGCTGTTGTTTCATTTCCTTGTACCTTCTTCCCCCGTTTAATCTTCTCAGCCCTTCCTCCCAGCTAGTCAGTATTC
AGACTTTATTTCATTTCTTGAAGGGTCGATCTGACAGAATGCACACACACACACACAAACTAAACATAGACATTGAGCTGA
TAACTGTCACTCATAAATCACGAGCCTTGAGGCCTCCGCTTATAATCAGATTCCCAATCAGTTGCAATATTGTCTTTTATT
TCTTCCATTCTCTCTCTCTCTCTCTCTCTCTCTCTCTCTCTCTCTCACACACACACACACACACATACACACACAAAGGAG
ACAAGACAGAGGCGTCAGACTGGCTGGGGTCTGGACTCGAGCAGAGGACGGAGAATATTTTGCACATGAGTCAAAATTAAT
AGTAGTGTTCTGAGTTCACAGTGAAAAGTGTGTTGTTTAGTAGAAAAGACACACGCTTGTAAAAATGCACAGATAAAATCT
GCTCAAGCCCGGACGCTGCAGCATGATCTAGTGTTTACACAGCAATTAACAGCAAAGCAAAGGTGTCTGTATTTTTGTTAT
CTTCACAGTTCTTGATCTAACCATGAGAGCAAGATGTCTTTTTTCACAAGTCTCATATCGCTGCTTAAAATAGTCCACAGC
CCTTTTTTCCATCAGTGACTAGACACAGAAACTTGTGCTCCATTTCCACTGACTGATTTGACGCTAATTAATTTGATGTCC
CTTATTAAGCAGCAGACCTAGTTAATGAAAGCGAGCGTGGCCTTAAAAGCTGTGGCACAGACGAAGATGGCTGCTGACCCT
TCGGCACCTGTGAAATGGAGTTGACAGGTGTCTGTCTGTCACAGGCGCAGGCTGGTCATTGGGAAGTTGAAATTTTAGGTC
AGCTCTAATCGCTGCTGCCTGTTAAGCCATTAGAGGTGAAAAGGGGACTCGGTATTATATTCTGATCAATTGTGCAGCTGT

CATTTCAAGTGGTTAATGTGCCACCGTTACAATTGTAAACTGCATTACATGCTGCAGAAATGAATGCTTGACAGGTGTAGC
CACACTAACTCAGGCTGTTGGAGCTTATTGAATGGTCAATTGCTTTCCCTCCTCCGGATGCAGACAAACAAAAATATGCAA
ACATACATATGCTCTTGTAAAAATATACAAGTGAAGCCACAGCTCAGCCCACCTTTATTTCCGTGGGTGCTATTTGCAGAA
TAACTTAAGTGTTGCGGCCATTCACAGCCCAAATGGTGAGTGAATAGTCAAAGAGCTCCCAGCATTACCACTGCTGCAGTG
AGCACATCCATTAACCCCACCCTAAACCCATTTCTCTCTGGACAAGGTGTTGTTTATACACCGGCTCAATTACCGGCTTCT
CTCAGTTTTGACCTCACTCTCTCTCTCTCTCTCTCTCTCTTTCTCTCTCTTCCTCCACTCTGTTCTCCCATTGCAACCTAC
CGCGGCATTAAAAGCTTCCAGCGCCACTCTTTTGTTGTTCAAATAGGCTCCTTGTTCCTCCCCATCACTCAGCCATTCACC
TGAGCTGGATTTGGACACGCGCCACAACCCCTCAAATCCCTCTGGGCAATGCAATCTTAATCCCACCGCCGTATAGCGTAT
GCACTGATCCTGGAAACACAGACTAACCCTCCAATATGTGTGCTCACTGAGCTGTGAATTTGAGTTTGCAGGATTCAGGGA
GCTCTGAACAACAACAACAATCATTGTCTGACCCCAAGTGTTCATTTAAAAGCTAAGGACAAAGGTCAAAGTCTCAATTAT
AGTCAGAGGGGAAAACCACGGAGAGGGTAATGATTCAATCCTAGAGAATGTGTTGCTTCTGTGTGCACAAGTGTGTACATG
TGATCGATCCAAAATGCATGTTTGATGCTTTCATATCTCATTAATAACAGCACTTTCAATAACATCGTCTCTCACTTTATG
GAAACCATGGCTCCGCTCTTGAGATGCTCCCTGAACTGACCAAGCAGCATTCTGAGCCAAAAGAGGGAGTGCAATGCAGAA
AACGCCCCAGGGTATATAAGAGTGAGCCCAAAGCAAGACAGTCAATGCAGTTTGTTTTTTTTAACCTTGAGCTCTGGGAGG
CAATGCAAATCAACCAGTTGTCTACCTGGAAGTGACTGTTGTTGTTAGCACGACGTCACAGTGATTCAGCCTATGAAGTTC
CTATTAATAGACATCGCAACTTTAAACATTGTGAACAATTTACTAGGTCTAGGTTTGCTAACCCTAACCCTAACCCTTTCT
CAAAATAACACTTAAGGCTGCTGTTTGTCGTCATTTTAGCTAACTTCCTGTCCTTTTCGCAGTTAGCAATCCCCTCCCTCC
TCTCTATGTCACTACACTTGCACGACTCCTTTCTTTTTTTCCGAGGCAGCTCCCTCAGAATTGAAATCTGTGTGTTCCTCA
TGCAGACAGGACCGCCTCTTTGTGGAGTACTCTGTCCTGATTGGATAAAGTGGGCAGGGATACCGGAAAATGTCACTCTAT
AACAGTGATTTCTTCGTGTCTCCTCTTGTTGTCTGCTAAAACATGAACAAGACTGAGTCAAAACCTACAGTATTGCTGGAC
ATTAAATGGTCAATGTGCTCATGAGGATGCACCTGTTCATTTTGAAACAGCAATGCCCAATGGGGAACGTCCAACACCTGA
CTGACTACATCACTCACTCACTCAGTATGACAGGGACAGACTTTAACGTTCAAAGAGCCACTTTGCTGTGTTCCAGCCAAA
AAATAACCAGCGGTCACCTGAGAAGTGACTTTCCACTCCTCCCTCTTTTATCAGGGAGAAGAGCACAAGTGCGTCTTACTC
CAGTGGACACAGTTTAACAGGTCACTTGGTTGGGAGCAACTGCAGCTCTGCCTCCAGCACCCACACATCCTTCAGCCAGCT
GAAACCAACTGGTGTTTGATAGATTCACACCAACAGTCTAACCCCCAAGCAGCACATAAATCCAGTTTGACCTCATGCATA
TATTCTCGATCCTATTTGTTGGTTGATCATTCCTCATATTCTAAACATCACATGTATCCTTCCTGTTTCCCTCTAGGCATC
AATCAAGGCAGCTGTTACAGAATCAACCATTTCCCTGATGACAATGATTACGACACGGACAGCTCTGAATACCTGCTGCGT
AAGTGCTCCCCATTCATTCTCTGTGCCGCGCATTGTTAAAACTGTCAGCCCTGTTGAAGGAAGTCCATTGACCTTCGCCGT
CTTATTATTTGGTTCTGAGAGCAGTCTCCAAACAGGAACAAAGATTCATGCAATGCAGATATTGTATCAGACTGTGAGCAC
AGCCTATGATAGTGTCTCACCTTTTCAGCAGATTATTTTGAAGCTGTTAATCACTCGTCATATGATGGGACAATTAAAGGT
AGCCTTGCCAAACCCGACTACAAGGTGCTGTATATGTTTTGTCCGAAAGCATTAATTAATTTTTTGGTATTCAAGAGTTTT
AATTGACTCTTATACTCGAATGAAATGTCCATGCTTGCTAATCAATTAGCTCCAAAGCCACAAAAGCTAGCTTGACACTGT
GGCTTCGGACATGGAGCTCAAACACAGAGAGAGGCTCATTATCATTTACTTATTAAAATTTTTAAAAAGGTGTTATAACGG
CAAACTGCAGTGCAAGCATTCCATAGTTAAGTGTTTTTTATTGCTTAATTGCTTTGTTGATGCTGGCGCAGGATCATATGC
TTATTTGCATTTTCCTCATTATTTATATAACGTGAAGCGTGTTGTGAATTGTGCTCACAGCAAGAGGAACTTCTTCACACT
GAACGGTGGGGAAGAGTGTTTCTTGGCTGTGTGGGGACAAAATCCTTGTGAAAATCCTTTTTCATTGAAGGTACTTTTCTG
TGAAAACAGGTTAAAAATGAAAATGGCGAGGCCTTCACTTAGATTTGCATTAGTTTGATATAAAATGGAGATAATCAATTA
TCAAAGGACCTTCATTTACTGCACTGCCAATTCAGCATTTGCCTAATATGCAGAGAAGAACAAAGACTTATCAGACTTTGG
ATAAAGTCCAAATGTAGTCCATTAGCCAAGAGGGCTTATTCAACTTTACAGTTGAATAAGCTCATCCATCCTTGGACACTT
GTAAAATGGCACAGTGATTCATTTCTGGGGAGGTGATAGTCAGTGAGGGCTCTTGTTACATCCCACAGCTGATTAACAAAG
CTTTGTCACAACAAGGCCCCAACGTATAAAAGGTAATGGAGTTTTAAAAAATGGATGGTAAGAGGTGAAGGGCTGGATAAA
TGAGGGCTCAGTAGGGTAATGAAAATTCAAATGATTTAATAGAGTCCATTTTTCTTTAACAGAAACGGTTGTTGAATGAAT
TTCCAGATCTCACACACATTTGCCTGTTATTGGTCTGTTTATGGTTGTATTTTGGTATCTATAAAATTGATTGAAATTAAT
CATGTTAAAACCATTTCGGTGATTAAATGATCTCTTTGCCATGTCCTCTTTCCTACATCCCTGCAGGTATAGTTCGTGCCT
CCAGTGTGTTCCCCATCCTCAGCACCTGCCTGCTGATGCTCGGCGGGCTGTGTGTCGGGGTTGGCCGAGTCTACAACAAGA
CAAACAACGTCCTCCTCAGCGCAGGCATTCTCTTTGTAGCTGCAGGTGAGCAGCAGGATTACACTGTCAACCATGCTCCGG
ATCATGTAAGGGCTAGGGGTTTATAATGAAGGAAGGCAGCTTGATGTGGCACCTGTGGTCACACGTTGTACACATGGTTTG
TTGGTTGGTTGGTTCTTCATCATGTCTCCATTAGGATTTGTCGATGTCTTCTGAATCTTTCTTGATTCTGTTGTCTTTTTG
TTTCTTGCTCATCAGGTCTGAGCAACATCATCGGCATCATCGTCTACATCTCGAGCAACGCAGGAGACCCCAGTGATAAAC
GCGACGATGACAAGAAGTACACTTACTCCTACGGCTGGTCGTTCTACTTCGGTGCCCTCTCATTCATTGTGGCCGAAACCG
TGGCCGTCCTCGCCATCAACATCTACATTGAGAAAAACAAGGAAGTCCGCTGGAGGGCAAGGCGCGAGTTCATCCGCTCAC
TCTCTTCCTCTTCCCCATACTCGAGGATACCGAGCTTCCGCTACCGCCGGCGGACGTCACACGCCAGCTCTCATTCTACAG
AGGCGTCGCGGGAGAACTCGCCAGTGGTTGGTTTGAAAGGGGTGCAATCTTCCAGTGGGGCTCTGGATGAGCTGTCCATGT
ATGCCCTGGCGAGGGACAGTGTGACAGATAATACGTACAGCCCCGAACATGAGGCGGCCGCTGAGTTCCTCCAGGTCCATA
ACTGTTTCCCCAATGATTTAAAGGACGGGGTGAATCGCAGGACCACACCGGTGTGAGGGGCATACCGCCGGGGCTCGAGGT

TCACCTGGAAGTGGATGTGGGCTAATCTGTTGGAGCCTGAGGGCTTGTGATAGCCTGCCATCATCACAAGAGACAGTCCTT
TCTGAAAAGACCGGAGCAAAAAAGAGGACAGAAGACGAGCCAGTCAGTCATTACCTGCTTGTAGAGATCATCGAAGCAGAA
TAGAGTTACATTGTCTGTGCAGTAGCCGATGCGAAATTAGTGTTATCTTTCAACTGTCAAAATGCTTAAACGAGCAGCGTG
TGGATCACAACCCTTGTTTGTGTATTAATCACTAAACATTGTGGATTAAGTGAGAGTGAAGCGTTGAGATGTGCTGATCAG
CAGTATTCTACCTGTAAAGTATTTAACAAGGTGCTAAATGAAACGTACTCTTCAACTTGAAAGTGTTCCATTTGTGCGTGC
AGTTGCTATAAAGTGTGGACCTCTGTCCGAAGTTTGTATTGTGTCCGCATAACCAGTTTGGTCTTGCCTTATACTCTAATC
AAAGGTGTGAAAGAGACAGCAGTCTCTCAGCTGTCCTTTAAAATTATATATGGCCTTTTAAACAAATTATAAATATTTTTA
ATGTATCATTTTTCACAACAGTGTGTTTTGGGAGTCAGTGTCTTAACAAATGACTGACTTGCTGAGTTGAAATGCATTTCA
TGTTTAGTATTTTGGGAGATTGTTCAAGTCAATCATCCAACATGAAAGGTTTTGTCTCCCTCGCTGCCTCTTCCTTAATCC
TATTCTTCTCTGACATTTGTCAGTGGAACAAAATGTCCGTTCTGTCAGTGGAACACTTGAATGGCCCCTTTCTGTTCACTT
CCATATCAGTCCGCCGTCCTTCCATCACACCCCCCGATGGATGTGACATTGTGTATGTGCTTGTCATGAGAGAAGAGCAGC
TGACAAAAAGGGGGTGCCCAGAACTGTGAAGACGGCACAGGTCAGGACTTCCTTTGGCTCTGAATGAAGCCTTGGCATCCA
CAGAGCTTAAAGGATAACGATCAGAGCCAGGCGGAGAATATTTGTCTGTCTGTCACACCCCTTGCGAGTCCATCTGGTCGC
CTTTTTTGAAAAGAGGACCCGGCCAGAAGCAGAGGGAGCTGTGTGTGGCGCCGTCCCTGAGGGATTTGGCTCTAATAACCA
ACCATGTGTGTGAGAGTGTGTGTGTGTGCTGTCTTTTCGCACGTCCATCACTGCTCTTTCTTTTAGTTCTCTCAGGAAAAA
CTGTGTTGTTATGGCAGGTTGAACACTTTGAGGTCCTCTCCCGACTTCGTTGTCAGCCCAGTCTCCTCAGTGTGAATACTA
ATTACACGACTTTAAACTTTCAAACTGCATGCAGTGAATACACCAAAGCCCAGTTTTGCATCTGGATCGTGATTCGGGTAA
AACAAAGCAAAAGTTTTTTTTATGATTCCTTTAGTTTGGCTACTTACTTCCTTATTCTCTGACTGCCTTCCGGGAGTTATA
GAGACAGGTGACCAATTGAAATGCACCATTACCTTGATGGAATTACAGATTTCTCTCGATTTGAACATTGTTGGAAATGTT
GTGCATTATCATTCAGATCTGTGGAAAATAGGGACACCCAAACAGGTCAGGTGAGGTAGTACAAAGAGCTGTCCAAGTTGA
GCGGAGGTTAGTTTTGATTGATACGTCCAACAAAAGTAGGACACTGGGAGACTGGTGTTTGTCTCCCAGTGAAACCTAAAG
TCATTGCTGACTTGTTTTAAGTTAAAGGATGTAACTTACATTACTTAAAATATGTTATTGATAACATTCAGCCACTAAATG
AGCCATTCCCTCTCCTCCCTACCATTGCATTCATGCCAAAACACAGCTGTTGTTCCAATCATCTGCCCCTCAAGTGGTTGC
CAGTTTGTTGCAATGCCTTACATTGCTAACTTTATGTTGCTAACGCTACCTAGCTAACGTGAGGCAGTACAGTATGAAGGG
AGCATCCTGTTGATGAAACACAGATGCCACGTGATACCAACGGTGTTATACTATTGGCTGACCAACCTTGTTAGAAGTTGG
AACCAACTGTCAGAATTCTGACACAGAGATAAAAGAAAACAATCATTTTGGACCCGACAAACATTTTAAAATCGTTAATTC
ACAATCATAGTAATGTTTTCATAAACATAACCAAGTATTTTGTTGCTTAAATCTAACCTAACTGCGAATGTTTCGTTTTGA
TTTTTGTTTCCATGATGACAAAGGTCACCTGACTATGGATTTGTTCTCCACAGAAGTTAATGGGAAATGATTGGGGTATCA
CCTGCATACTAAATTGAACTTTGGTGTATGCACTCTAAGCAATTTGAAAGCATAAAGTCGTGTTGGTTGTACACAGATTGA
TGAGATTGGGTTGCTGTGGCAGCGGTGGGAAATGGACCAGTGATAAAGAAAAACCTGGTGAAAATGATTTGAGTAAAAAAA
AAAAAAAAAATGCCATTACTGTATTTAGTAAATGCTGTGAGTATTTGCTTGAGCTTGCTCCAAGAATCAGCTGGGGTGGGG
TTTTTTTCAGTCTGAGGGCTATTCAGTTGGTTCTGGTTTAGTAAGCGAGCTCACTCAACCACAGAGGAGCATTTGATTGTA
TTTAGTGTGTTTGATATGCCATTAGAGATGCCACTTCATATACCAGGCTGAGCTCATGTAAAAGAAAGCCATCAGTATGAT
CCAATGTAGATCTTTTATATTACAGTTTGGGGGGGGAAAGTAAAATGTGTTTGTTTGTAATGTAAAGCTGTAAAGTTGTAT
ATAAAAGTACAATAAAAAAATACAAAAGAGAGAAAAAAAAACACTGAATGTGCAAAGACATGAAGACAAGATTCAAAGCATTA
TTTTACTTAACAGTGTCAATGATAATGACTTGATGCAGTCTGGTATTGGTCATACTATTAAATCTACCATTTTATTTTCAG
GTAAACACACACTGGTTTTGTTGTTATCAGTCAAAAACAATTAATCTACTGAATATTCTGTTTTGTAATGTGAAGTACAGT
AAGTTAATTTAGGAGATAAAAAATAGATTTTCTATTCCATGTGTATGCGGCAGCTGTACTTTACTGTATGTGCTTAAATTG
ATTTATGGCTTATTATTGTGACTTAAGCACATTCTCCAGTTTTTTGAAGAAACGGACATCTACAAACATTTGAGTACGGAC
GTTCAGAAGGGAGCGGCTAGTCGGAGTTGCATAATGAAAAAAATCACAAGGAAAGAAACTGAGGTATCAAAAAAAGAACTT
AATCAATCAATATGTGAAAAAGATCAAACCAACTGGGAAAACATCACTGTTACCTGCACCGATTCTTATTTTTTGAGTGCC
TTTTTTAATTTATACACTGTATTTTCTGCTTTCTATTTAATTTCTGAATTTACTTCAATTGTTCATTTCATCATCACATTT
TATTTTCAACAAAAATCATACTGTTATAGACAAATAAACTAAATCAGGGTTATATTGTTGTGCTTTATTTCTTTTAACAAG
ACTGAGAGTTTACAGTCATGCTGGCAGTACGGAAGTGGTGCTAACATTGACGTGCTGTCATCTTCACAAGAACAATGTGGA
AAAGGTGATGTTTAGCAGGTTCACAGTTCAGTGTGTTTGCTTGCCAACTTTTATTAATTAGCATTAAACACAAAGTAAGGC
TGAGGTTGATGGGAATGGTGTTTGTTATGTACGTATTTTGTAATAAACCAAAGTATTGGACAAATTAAAAATTTGAGCGGA
TGATGGCACTAAATCAAGAGGGATTACCACTGTTATTGAAATTTATCCAAAGGGGGGCATGCACATGTGTACCAAATGTCA
TGGCCATCCATTCTTTAGTTGTTGAGACATTTGACCGATTTCCACAAAAATCAACCTCCTGGTGTTGCTTGGAAAAAAGTC
AGGTGATCGCCAAAATCAGAAGATTATAGAAATGTTAACTGACATCACAATTACTAAAAATCCACACATCTGTTAATGTAA
TTTAATAGGACAACCCACTAACTATGTATAAGAAACACATGTACACATGATGTATATGATTTATGCGAAAATAATTACAGC
CAAACCCGTTCATCTCAGTGTAGCTTGATTTCCATTGAAGACCTTTGAATGCAGTGCTTCATTCACATTTCTGTGGCTGAC
CTTCTCTGAGTCCTGAATGCCTCTTCAGCTTACCTGCACAATACTCGTCTCGTATTGTTTGGAAAAGAAATAAATCAGTCG
GCGTCGTGCTGATAGCAGCGCTCTTTGATCAAAATGTTATAAAGCCCTCACTCTCGCTCAGAGAGATGCCACCTGATATTA
CAAGGTTATTTTTTTTATTTCAGCTGATTCATCAGTGAGTGGAGTGAACCTCTGTTTTCTGAATGTGTCTGAATCATATTA
TTGTCGGGATTGAAAAAAAAAATGGATGGTTGGCAGTTAAATGGAGCTGATCAGCATAGGTTCAGTTCTTGTGTTTTAACT

TCTCAATGCAGAATAAAGGCTTCTCCACATTACAGTCATAACTATTTTCCCACCTTCTTTTTAAAGGGGTCATACATGAAC
CTGCAAGAAGAATATGGTTGTTCCCACTGGTACCTCTTTTTTGGTTTATAACTCTTTCTGTAATAACAAAATAAGTCACAA
AAATGTTGTAATATTCCTTCAAAGGACGAAGCTGTTCAATAACCTGGGCGCAGCTGGGGAAACTGTTTTCTCTACTCATTT
TGCTCAGCTAAGTATTGGGCTACAAAAAAATTACAAATGACTGTCAAAAAGAACATTAAAAACAAACTAGTGGGTAGCAAT
CCACGGTGGCAGCAGCTAGTCTGCTAAATTTGGCGTTTAGTCCGCCAAAATCAGTGTTTGTTACCCCTAGATTCCAACAGA
TACATGTCCGGTCCGGCACTCCGGAGCCTTCCGGAGCAGATACGCAGGGTTTCTCTGCATATCTGAATCTCATAAAAGAGA
CAAATACAAGCACTTCTTCTAATCCTTTGGTTGGGAACACTTTGTGTTGTTGTGTGTATAACACATATGTATAACTGTGGT
CGCGCGTGATTATGTAATTACTGCGGGAACTCCTCGGTCTCGCAATTTCAGCTGTCCGATGGTGCTGCGCCGTGAAGGACT
CAAAACGCAGCCGGTGGGGGTTGCCGGACCGGAGCCGCAACGCAACGCACATGCATCCAGTGGAATCTTTGGGTTGTTGTC
GTCACTGCTGTAGTGCTTGCTCATGGTAATATTTCTGTAAATATGCAATTCACGTTCTTTTGGTTCCATTCTGAGTTTAAG
GACCAAAAAAAATCCAGGTACTGTTTGTAAGGAATTGTGAAGCAGCCAGTGGCTGAGTTTTGGCTAACGCTCAATCGAGACA
CTGAACAAAGTCCAGTTTCCTCTAACAACTTATCCAACCAGCTCATTACTTACATTAAGATATCGATTAAGTTGATGCCTC
GTTCAAAGTATCGGCGTCCACGGAGGGTTCAACCTCTTTGGCACTGGTTGCAGGTGTGCTAACGTTAGCCAGGTGTTTCCT
CAGGTTAGACACCCACCCAAACCTAAAGGAAAAGCACAATTTCTCCTACATTGCATTATCCTGTCAGATGTGTAATCATGC
TGGTGAGATTTCATTTTGAGAAATGAAGAGGAATGTTTCTGCAGCTGTTTATCTAACTGGGGAAATTATCTTTTGACAGTA
ATGTCTGATGTTGCGGTACAACAGCTACATCAGCCCCTTGTAATTTCCACCTAATTTAATCTCTCTTCAGTGTGAGCTCAT
TAACTCACAATTCACCGTCCCACATCACCTCCTGCTTCATGTAGATCAACGACCAAAGCTTTTTGCCTGAGCCCGGTCAAG
TGAGCCCGGACGACGTTGCAGGGGCGAACACGCGTGGTAACCAATCCCGTTGTTTTAGACAGGAATATTAACATGACCCTC
ATAGAAATTGCCACAGGGCATGGGTAATCTAATTTAGCTTGAGTAATGTTGCCTGTCATTAGCAAATGAATACAGTCGGGT
CCCTCTGAAAGCTATGCAATCTATTCTGCTATGGGTAAAATTAGCCTCCAGTTAAGCTTGTTTTTCATCCTGGACTAAAAT
TTCTAGCTGGCAGCTGTAATTGAACTTAATGACTGCTGAGTTTAGACGTGTCAATCCTGATTCCAGCAGAAGTGATTTTGC
ATGTTTCTGTCACTGCTCTGATTGTTCCGGTTCTGAGAGCTCGCCAGTGATATTTCTGCCAAGTGGCACTCACAGGTGAGT
CAGAGATGCCTTCTTGCCAGCTGCTGTCATCCGTTTTTCAGTGTTTCTGGCATGAGGTGATCTCTCCTGTATGTATGACAC
ACTCACGTCGTCGATTACACGATACCACGGTCGTCCGCACGGCTGCCTGTTGCTTAGAGACTCTGCAGCTGTGAGGTGTTT
GCTCTCTCCGCTGTCACCAGGGGACCATGAGCAAATGAAGCAGGTCAGAGGGTCACCCAGTAAGACGGGCTGTGCCAAAAG
ACACTATTATTACACACTTAGAGACAAGCGACGCTCTCACAGCCTCAGCGTGGGACTCGTCCTCTGGGAGAAGAGGCAGTT
TTGCTGCAAATGCATCCAGGTGGAACGTGATAATGAACACAACTGTCTCTCATCTCCTCTCAACCCCCACTGTGGGGTTTC
TTTTTTTTTGGTTTGTTTTGAAAGCTGGTGAGTGAGATGATTTTTCTTTAATGATCAATTTCCTGGTTCAGTTAGCTTCAG
ATGGTGTTTACAGAAAGCATTTCCTCTCTTGTTGCATTAATTTTCTAAAAAGTGTCAGACTCTCTGTGGTATTTCTGCTAT
TATACACATTTTGTGTAATTGGCGTACAGACTGTACAAACAAAACAGATTCTCTCTCTCTCTACCTCCTGCTAATGTCCCC
ACCATTTAGTGTGTTAAAAACCCAGAGCTGCGAGTGTTGTCAGGGCTGAAAATGGGCCAGAGTCCTGTCTAAAGTGGGGCA
CAGTTTATAAAAAGCATGCCCATTTACTCAAGTATTTCATAGGGAAAAGGTTGATTGTGCCTTTGAGATGTATCAGCTTTT
TACAGTTTTTTTTTCCCATCTTCAAACTCTGTGATGTCATTTATTAGTAAAGCCATCAAAAGCATTTGTGCGCTGAAACAA
TATGATTAACGATAGAATTAAAATTCTTAGGATGCTGAATCAAACAGTATGTCACAGCCACTTGTAGGTTTTGCTCGAAAT
GCACATTAAAGGTGCACTATGTAGTTTTGGGGAGACATTTTATTAAGAAGAGAAATGTCTAAACAAACTAAATAAACAAAC
TCTCTTTATTTTCATGACTAAATGAACTGAATAAACAAACTGACCTTAAAGGACAACACAGTTTCATACTGTTTTACTTTG
TTTATATTTGGCGGACCCTGCCACCTTTCTAGCTTCAAACAGTGTTCTGGAGACCTTATTTTCCTCTGAGAACAGTTTGTT
TATTCAGTTATGGAAAAAATAAATATTATTACCTCATTAATATTGTTATTAAGTTTTAATTTCTTCACCAAAAACTACACA
GTGCCACTTTAAAATATGACACAGGAATATATGACGATCTAAAAATCGAGAACTCTCTGTTCATGAACTGAACGTCAAATT
TTAGAGGTTCTATATTAAAGCCCTTTTTCTGTCTTCCTGAATCTGGATCAGCTGCCAGCATCAGGTGGGACCCTTGTCTAG
AGCCTTGTCTCAACTGCTGATTGGCTGAGTAAGTAGATCAGCTCATTCAGACGACAACTAATAAAGGAAATCTCCCCCTCA
GGAGTCCCCTGGCACATGGCGCCTGAGACGATTGCGCTGGATTGGACTGAATAAGAATATAAAAGAGACGTGACACAGTGT
TTCACATACATGAGAAGTTGATAACATCAGTTTTTGGCACTGCAATTAAATATTCAACAGCATTGTCAAAACTCACTCAAA
GTTTTCTCACATTGTCTTTGATGATGTAGTTAGAAGTGCACAACTGGAACGCTCACAATCACCTGCCTGTGTCCAAACTAT
AAATGAAAGTGTCTGATCTCGACCTGACTCTCGTCAAAACACCAGAGTGGTCAAGTAATGGGTGTAAAAGGCGTGGGTACT
TTCTAGTACATCTTAATCTTGTAGCTGAATGGCCTCGCCTCACCAAAGGCAACCAGGGAGGGAGAGGAGGAGAGGAAGAGA
GGTAGAAACTAGGGCACTGTTATCTGGCCCCTTTTCACCAACCACCAGGCACAAGGCAGAGAAAGAGAAAGAAAGAGCTGT
GGACAGGAGAACGCGAGAGAGACGAGGACATCAGAAGTGTAGTGAAGCTATAGACAAGACGGGGAAACCTCTGGAGTCAAC
ATCCGACGTCAAGGAGCTGAAGGCAAACAAGACGAAGGCAACCTTGAGTGTCCTACGCAGTCTTTACGATCTGTCACACGT
GTGCAAACCACAGGCACGCGCACGCACACACATCTACAACTTTGCACGCAACATGTTCGAGGAGCAGGCGACCAAGGTCAA
GATCACGTCTGTGGTGATCGTGGTGGGCATGTCGGCCATGCTGGCGGCCGTGGTGACCGACCACTGGGCCGTGCTCAGCCC
CCGGGTGGAGAAGCTCAACGCTACCTGCGAGGCGGCCCACTTTGGCCTCTGGAGGCTCTGCAAGAAGACCATCTTCATCGT
GGAGGAGGACCCTCAGGGCAAAGGCTGCGGCCCCATCAGCTTGCCTGGAGGTATGGAGCCTCTTGATCTCACTGTTACTGG
ATAAAAACCTGCTGTTTGGTGATTTTTGCATTTCTGTGCTCAAGTCTAAACATTTCTATTTGTTATCAAAGTGCTTTTTTT
TTTTTCTTGTGTGTTTGCTGACTGACAGCTCTCATGGCTCAAAATAATCCCGGATGTGAGTTTTTCCTTTTGCCAGATTTA

GAAAGACAGATGAAGAAAACAAACATTTGTTGTTGGAAAGCAGTTTGGAACCTGTAAAGAAATATTTATACTGCAGTTCTG
CTTTATGTGTTCGTGTAAACAAGCAGGTCAAAACCGGCAAAAACAACAGAAAAAAATTGACTTAAGAGCTGAATTTGCTGCC
GTGACTGCTAGCTCTCATCTACTCACTTTTATCACAGTTAGAATTAAGCCTCGACTTTGACATAATGTGAGTTTCAGCAGT
GCAGAATGGTATTTTTGCCATCACTTTCTCCTTGTACATCAAACCCGCAGATGCTCTGACATCTAAAAATCATTTAAGGAC
AGTCTGAGTGGATCTGTGTGTCTGTCCCTTCCTCTTTTTCCTCTCCTCTCACACACAAAGGCATAAACCAGCACATATGTT
GGCATGTGCATGTCCAACACACACAAACACACACATGGACATGCATTTGAACTGAATGTGTAAGATTTCTGCGGCCAGCTT
CTCACTGAACCATCATTATCTTCCACAAAGGAAGTCTCTCTGAACAGACTGCCAATTTAGTGCAGCCGAACATCAAGTGCC
TAATGGAAGAGTATGGAAGCTATACTTAACATTTAACAAAAACGTGAATCTGTTCTGTCAGAAGATGATGGACTCTCAATA
TGTTAAGAAGTAGGATTCATTTTACCTCACAGCGAAGACCTTATTCCTCAACTCCTTGAATTTCTTAACAGGAAGTGTTAC
ATAATTTGTCAGACTATATATACAAAAACGTAAGATGATAAGTTGCTATTTAACTCTGTCATCTCTTGTTATGTCTGCCTC
ACAGTATCGCAGCAGATCCGGGCTTAACTGGCTTTGAGTGAACCAGATGTGAATCAGACTGTCTAGCCTTGAGTCAATTTT
ATTAATGCAGCTCAATATCACCAATCACAATTTGCCTCAAGGGTCTTTACAATTCGTTATAAGACTCAATTCAGATCAGGA
AAAACTCCACACTGCTGGCTGTGGGGAGCCCTTTTTAGAGGATGCAGCTAATTCAAGCAGTATTTTCTGATACACTGATAA
ACCTTGGTTTGCCCAGTGTTTAAAGGGGCACTATGTAGTTTTGGAGAAGAATTTCAAACCAAATTTTAATCTTTACAATAT
TAATGAGGTAATGATATTTATTTTTTCCATCACTGAATAAACAAGCTGTTCTCGGAGGAAAATAAGGTCCCCAGAACACTG
TTTGAAGCTAGGAAGGTGGCAGGGTCCGCCAAATATAAACAAAGTCAAACAGAATGAAACTGTGTTGTCCTTTAAGGTCAG
TTTGTTTATTCAGTTCATTAAGATCTTTCTCTTCTGATTTTAAATTTCTTCCCCAAAACTATATAGTGCACCTTTAAAAGT
ACCAATTTCTGCGAAAAACTCTCACCTTATTATGAGACTTAAGAAAGAAAAACTAAAAAAGCTGGAGGTGCTGCTTAATCC
AAAAAGTGAAAAGTCAATCTTCCACTTACACATTCATTAGGAAGTTATTTCCTATCCTTGCATAACACAGCATACAGTAAG
TAATTCCAAAAAAAGTAAATCTGTCTCGCATTCGAAAGTCTACTTTTTCAAAATAGCCTTGTGGCTTTAATTACAAGCTCG
CTAAGAACAGAGCGAGCCCTGGGGTAAACACAGTCTCTCAGGCCTCTGGCTGTGTGTGCGTGCCTTGCAGCCTAACGGAGA
GATTTCAGTCTGACTCTATCATTGACTTTAGCCTCCAGCTCCAGCACCTCAGGATCAGTGCAGGCCTTAATGAAGTGTGGC
TTCCCGCTCTAGCAGATGAGTTAGAGTACACCTGGGAGACTCAGGAGGGATGAAGAGGATTGGGGGAAAAAAACTGGAGTC
TGCCTGGGAGGATAGAGGCAGGGATGCCTCTTTAGATGAGTACGGCGGGGGCACTTCTACTGTGTCCAAAGAACAGTTGGT
ACTGTCCTCAGATTTATGCATTACATGTCGGAGAAACCAGGAAGAGGCAGAGAGGCTGATGGAGAACAGTTATGAGAGCTG
CATGTCACAGAAAATCTGACAACATCGAGGACAACATTATCTGTTGAGACCTACTTTCCCATGAAAAAGTCACTTGTGGAA
CGTATGAAGAACATATTGCTGCAAAGAGACTCGATAGTCTAATTTGCAAAACGATCCAGTTACAACCTCCACAAGCTGACA
GCAGCAGTAACACGGTGCCCTATCTGTGGAGAGTCCCAGCTGCTTCTGTGACACGCGACACAACCTGGAGTGGACAGGAGT
GACTCGAGAGAAGCCACCTGACAGACTGAAGCTGTCAGCCGCTCACCCTTCTTACATCCAGCAGTGAGGGGAGTATGAGGC
AAGAAATATCAGCTGTTTTGACAGAGACCCCCATTGCTACATTCCCATGATCCCATGGTGCTTCCACATTTGGCTTATGAG
TGAAAATGAGTCACATTTTCATATTACTTGTAAAAGTCTAGTGATTTGGCGTGTGGCTAGGCAGCTCTGCCATTTGCTCCC
ACGCCAGGCTCAATGGTTCAGTTATACAAGGCCATCTCTCTCCATTTGTGGCCCCGGTGCCATCAGAAATGGATCTGTAGG
TGTGGTGTCGTGTCCCCAGTCGTTCTAGATGCAATTCTTGTTTGGAGAGTGAGTTTCAGTGACTGTCCTCACCAGACGAAC
TGATTGTTCTTCTCCAGTGGCCAAGAAAAAGCATCTACAGGTTTATTCTCAACAGGTGCTCTCTCTACTGGCACCCACTTG
GTCAATCTGCCAGGTTGGTGTAATTTGACAGCAGAGAGGAGCAGAGAGAAAGAGCCACTATCAGGATTCAGCCGCTTGCTT
TTCTCCTTGATTGAAATCAGCAACTGCAGCTTGGCTGAGTGCTAAAATCTTGGCTGGATTGCACACTGTGTGTCTAATTTC
ACATGTATGAAAGTGTTTCTCGTGTGATAAATTCACAGGTGTTTTTAAAGTGTTTGCAGTTAACAAGCCAACCTTCACACC
AACATTACCACCTCATGACAAGGCCTGCATAATGAAATAGTTTTGTTCTTTCCATTGATTTTGTACTAGTAATTACAACTC
AATTGCCAGAGTAAATGTTGGCAGTGTACATTTCTGCAAACCAAGGATGTGTTAAAACTTTATAATTCTTAATTTCGCAAC
CACCTGATTAGGAAAAGATCATGTTTTGTCTTACCTGATTCTGTCGCCATTAACACTCCTGGAAATTGTCCCTACATCTTG
TCAAATATGTCAAGTTTTGTTGCTACCAACAGAGCTTGAAAATGTTTGATGTGTCATTAAAAATATAAAATAGTTTTGGGC
TTATGAATGTTTAAATGCCATCTTGAACAGTCGTCTCTCATGAAACACTGTCTCAAACAGTGGTGTCTCATTAATAGTATC
CAGTGGTTTAGCGCATAGAAATGTTGAAACATCATCTTGCTAATAATATCCAATGGTTTCATTCTTACAAATGATGAAACA
CTGTCTCCAACAGTGGTGTCTCATTAGTGATATCCCATAGTTTTTGTGCTTATAAATGTTAAAACACCATCTCAAACAGCG
GTCTCCTGTTAAAGATATATGTAAAAGATATGGTTTCACACCTACAAATGTTAAAATGCTATCTCGAACAGCAGAATCTCA
TGAAACCCTGTCTCGAGCAGTGATCTTTCGTTAAAATATCCAGTAGTTTAGCGCTTACAAATGTTGAAAAGCTATCTCGAA
CAGTGGTCCCCTGTTAAAAATATCCTGTGGTGTCATTCTTACAAATGTTAAACCACTGTCTTGAACAGTCTTCTCTTCAAT
ACTATCCAGTAGTTTTGTTTCTTACAAATGTTGAAATACCATCTCCAGTAGCGGTCTGTCGTTAATAATATCCAGTGGTTA
GGTGCTTACAAATGTTGAAACACTGTCTTGAACAGTGGTCTGTCACTATAAATATCCAGTGGTTTCATGCTGCCAATGGTT
GAAATGCCCTCTTGAACAGTGGTCTCTTGTTAATGGTATCCTGTGGCTTCATTCTTACAAATGTTGAAGCACAGTCTCGAA
CAGTGGTGTCTTGTTAATAGTATCCAGTGGTTTCGTGCTTACGAATGTTGGAACGTTTTCTCGAATGGTCTTCTCTCATGA
AACGATGTCTCAAACAGTCGTCACTTGCTTTGAAGCTGTCTTGCCTAGCTGTGGTTTTTGCAGAAATTAACGACTGGGCTG
CTATTCAACCTAAAATACCATTGAGCACACTCAACATCTGCTCTTACTGGTATGCTGTCGAATGCATAAGCTTTGTTCTGG
AATTAAAGAAAGCTCTAATACCGTTTTAGGTGGATCTACAAAAGTTATGGAGTGGCTGACTGGCTAAAAGCAGCACACCAG
CGTTGCTTTATCTGCTTCTCAATTTAAAGGTGCGTGTAGTGTTACACAATGGTTAAAAAAACAACAACCCTGAGTTGATAG

AGAGTGCACTACCAGATTACATCCTTGATGAACGGCATACCCAAGCAAGCAGCGGTGATTGGACTTAATGACACATATTTG
CATTTTCCCAAACAGAGGGGGAGTCGGTCATCAAGAGGCGCGTACACAAACACTTTTTCATATAGGCACAATTACACCACT
TTTGTGATGGGGTTGCATAACAACAGCAGCAAAGCAGTGAACCAAAGTGACTTTTGAAATTAGCTTCCCACAAATGACGCA
TGACAGCTTGAGTGCTGTAAAAATCAAACAAACCCTTTATGGATCGCACCATCACTGCTCGGCTGGGAAATAAAGATCATA
ATTTGGTGCTTTTGTGTGCGCATATGTATGTGCGCAAAGAAGTCAGGAAGAGGAATTTATTATCAGAGATGTTTTAGTGCT
TCCAAATGACCGAGCATCTAAGATATTACCACCAGAGAATCTCAAAGAAAAAATGTCCTCCCCCACGGGTTGGTGCCCCCG
ATTCAAGCACCGATGTAACCTTGAAACACCATTTTCACTTCATAGACACCCAGTCATTTTCCTCTCAAGTCTGCCAGGTTC
CTGCTTGGAGTGCAGAGCCAGAACTGTGGAGGTGTGTCGCTGATTTGGCATTCAGAAAACAAGTTTAAGGTTCAGAATTTG
AATGAATCACTTCCCATTATGTAAATTTAAAATAAATAAAAGTAAACACATATTCAGAGATAACTGCTGGCATCCCTTTCA
GAATGAGATGCTGGGATGTAGTTTGTGGTGACTATCATGATGACAGACAAACTGAAGATGTTTTCGGATTGTGTGATAACG
GTCAGGCCACACACACACAAACACACATACACGCACGTACCGGATTAGGCTGCGAGGACTCTCCTGTGCTGCTGTGTCCCAGT
TATCAGAGCTCCTGGTGGAATAGCCATTCAAATACCACCAGCACCACCACCAGCAGCAGCACCACCCATCCTATTCACATT
GACCCAGAATTAGTCGGTCATTCTGCGTGGCTAGAAAAAGAACAGCCCCCACCTCCAATCACCCAAAAAACCTGTCTTTTG
TAACGCTCAGGCAAAAGATTAAAAAACCTATATTCAAGACCACTTTCACAGCTGTCACTGTAGCTAAAAACACGGACATAC
AGTTATTCAGTGAAAAACATCAGTTGGTTTTTGATGTCAGTTCATGTATTACCAAACTTTTAAGGCGTATTCAAATGGTGA
AAAAAGAGACTTCAGCATAGAAGCCAGCTGTAAAAGGAAAAAGAAAAACGAGAGGAATGCTGAACTGAAAGTAGAAGTTGA
GTTGGTAGAGAGAGACAGGTATAATGAAGGGGGGAAAGGGGAGGATGTAGAGGAAGGGAAGGGTAAGAGAGAAGCTGCATA
TCAAAGAGTGGAGGTGGCAGCACAGTTTAATGAAAAGAGGCAAAACTAAAATGACAGGCGATACCTCGTTCTAATCATTCT
CCCTCCCTGGAACTAAATATCCCCATTAGCCAGACTGCAAGGTCAATTACACGCACCCAGATGTTCTCTTCCTCAGTGGAT
GTTCACCATCTAAAGTTACGATACAAGCCAGATTTGTTTTTAACTGTTTCTGGGCAGCTTGTTTTACTTTCATTACAGAAT
ATGAGCGATCAAGTTCATAGTTTTGATTCACTCTCACTGCTCTCATAGCATCATGTTTTCAGCTAAAAGCTCTCAAACCCA
ACGGCACACTACCTGCTCAACACCAAACAGAAACAGACACAGGTGGCTACTAGCTGGTGAACATAGTGCAGCATTTGGCAG
CTAAAGAGCCTCATGTTTCTCTCATGAGTTGCTATAGACCAAAAACAGAGCTTAAGGAGCACAAGTATCGGAGCTGTTCAC
CAGATCGTCAGCAAAAATACTCCAAATGAAAACTAATGTCTCTCTGTTACTGCTGGAGGCATAAATAAGCAACTGCTGGCT
GACAAGTTCGCCATATCAACTTAAAAGGTGATTATGATTATGATTTAATCCAATAAAATCCACTGCCGTCACGTTGAAATG
ACAAACCTGCTCTTGTTTAATTCTCTGTGAAATCCAAAATTACTGCTAAGTTGGTTTAATAAATCTGCCACCGTTAATACT
GTAAACTGCATGCTAGAATGGATAGCTTGACTGGTCTCTACATTACCCTTTTTAATTCATCAGGATGAGGCAGATAACCGA
AGGCTCAGTCTTTTGGAGCTCCCTTTGTGTAAATCACATCTTTATGTCTCGTCCTTGTGTGGTGGGCAGATAATAGTCTTC
ATAGAAACTGGAGTAAATCTCGCAGCTACACCCTAACACGTATTATCTTAGATTAGCTGAGCCCCAGAGGTAAAGACAGCT
AATGAGAGAAGACTTATGAAAATAATATATTTGCTGATCCGAGCTGCACTGTGACCCCGCCTCTGGTTCTCCCCACTGCCT
GCCTGCCCATCCCCTCACACACACACACACACACACACACACACACACACACACACACACACACACACCGACTTTGACACT
CGCATACAAATACACACACATATGCATGCACACCCTCCTCTTGATATGCCGTTACATGGGTAAATAGATAGGACTAGGCAA
GACCTGCAGCAGCCTGTTGCTAGGTTACCTCTTTAGTGATTCTTTCTCATCCTGGATAACAAACAGCTCCTCTGTTGCCCG
GTACACACACACACATACACACACATCATATGTGCACACACATGCGCACATACAGACACCAACACACAAACATTGTCCACC
TCTGTGTGGACAGAGAGAGCACCTGTCTGCTTTATGGGTTTAATCTCAGCAAGAAAAGGATGGTGAGTGGAGGAAAGAGGG
GACTCTCACTTGGCCTTTATGTCCTCTCTCCACCCCTCCCTCTCAGCCCAGCTGCCCTCAGAGACAGCAAATAGATAGATG
GATGTGTGGGCAATGACTGCAGCCAGGAATCGTTTTTTTTATATATATATATATATATAAATGGGACTATATTGAAGATTG
TGGTCAAAGGACAAGAGTTTGGGCAAACACAGTGCAGAAATGTGTCATTTCTGTGTAGAAATAAGTGTGAACTGATTTCTG
AGAAGTAAACACAGCCAACATGGAGGACAGTGACTTTAAGACATTTAATTTTAGGATGACATCAAGTCACAGAACACTGCT
GTTATTTGGAGGCGGCGTTGAGCGTACATCTAATCAACCTGCCAAAGAGGAAAAGCAACAGTTGTGCATGCTG

SCAFFOLD 2 (123,998 nt); encodes pvalb3, pvalb9, and pvalb10

AAAATGCTGCTGTACACAATCTGATAGATTTATTATTTGGGTTACCTGAATATAAAACATGGAGTCTACTGTAAATGCTCA
GGACAGAGATCAAGTGTTCAAAGGTTGTGCCAGATTCTACCAGGTTCCCCTTGTTTCCAGTCTTTGTGCTAAGCTAAGCTA
AGCTAACAGCGGCTAGCAGCAGCCTTATGTATACCACACATACTTAAGAGTGTTATCAATCGTGTCATCTAACTCTGTGCA
AGACATGTGAAGCATCCTGCTCTTTTATGAGTAAGATTGTATATCTAAAATTGTTTCCTAATGCTAAGGTGTGTTTGTCTC
TCTTAATAGACGGGGCTGGTTTCCCTCATCTTACTGCAGACCTTTTACTGAGTCAGTGATATCAAATAGGTAAGACAAAAT
AACAACATTATGGGAAGAGTATCAATAATTTGTTTTACGGCTGATGTTTGGGTGCTGAACCTTTATGTCACATGTCTTAGC
AGCAATCTGGGCACGCCGGTGCGTAGACTGAGCTTGGCCAGTCTGCCTGAGCCGGAGGACGAAGAGGGGCCGGTGCTGCTG
CCACCACCAGACTACAGCGACGACTCCAGCCCAGTGGTCCGCTCAATGCTCTCACCACACAACACAGTCAGTATTCATGAG
CATTTGTAGAAGCATTCACTCACCAAATGTTACCTGTTGTTACCTGTTTCAGTACAAAGTCATCACTGGTGGAGATGGTTT
CAATGTGCTGTGTACAGTTTAATGATGTAATGTTGCAGGAATTCTAGTTGTTGAATTTCCTAGTTGTGCCAAAAAGTTCCT
TAAAAACTAATCTTAACACGGCTTTAATATCAGGGTTTGGACAAGTAACATACTGACGTACTGTTGTTTTAAACAAACACC
CTTTAGTACTTCTTTATTAATGATTGATTGTTGGTGTTTGTGACTAAAAGAAGCTGTTGGTTTGTTGTAAGGATTACATAA
AAACTACTAAACGGATTTCCTCGAAACATGGATGGGGGATAAATCTCGGCCCGGAATAGTCCCCATTAACTTTTGGTGCAG
ATCCGGAAAATTAGATGGATTCAGCATTTTTTTCTCACTCTTTAACATCGCGAGATACGCTGCTAAAAAATAAGTATCTGT
AAGTAAGTAATTGTCTGCTATGCTGTCACATGCTGTCTGCTATGCTAGCTAGCTAGCTGCTTTGAAGGTAACTAGCTACAT
CTTTGCCTCAGATGTCAGTTTCTGACCAGACAAGCACAGTCAGCAGTCAAATCCAACTAGATAAACACAGGGTAGTTTAAG
TCCTCTTCTTTGACAGCTATGATGTGCCTTCAGGCAAGGCACTTAAAGCTACATGTTTGACTGTCCTTTTACCAGACAGCT
GCCATGTTAAAATGTCTATGAAGTGGGTCGTTCCAGAAAGGTACACGTTTCCTCATTGAATATCCTTATTACCTAAGTCAT
TACCGACTCCACAGTCAAACCACATATAAAAATGCCATCACACGTCAAGCTTCCAGCCTGACTAATCCTGTCTTATCAAAA
TCTCCTAACTGCGTAGTCATTGCCTCCACACCCTTTAAACGGAGCAGTGCCAGAAACACACCTTAATTCCTCTAACCAGTT
TGAGGAAGTGTACTATATTTATTCATGCAGCTATTTCGCAAAACAATTTGTGAGCCTTAAGGTTGTTATGTTTACACAAAT
TTTGCCACCAATACAGACTTAAGTAATGTGTCAAGATGAAGAGAGTTGTTGGCTCGAAACCTTTCAAAAGACGATGTGAAA
AATGCTCCCAGCCAGGTTTTGTGCAGGAATGGAGCTAGAGGAGGAGGAAAGAGTCACAGAGGGAGATGTAAAGACAAGAGG
GTACACACAGTTTCATTCTGTGCCAGCTGATTGAGAATGAACTGTCCGGCCAGCAGGACAAGCCCGGGCAGGCACACTTCT
TTCTCCCTCATCATCACCTCTTCACCGCCGCCCTGTCTGTACTCAGTGTCCTTTGTATCTGCTGCCTCAGGGTTTTACCAT
GAAATAAACCACAGACAGGGATACACCCTCCCAACAGGAGGAACCCCAGCAGTGCTTTTCACTTTCACTTCTTCTCACATA
TATTCTTTTTAGAAACTTAGCCCAGCATGGACAAAGCTTTAATAATCCCACTATTATAGGTAGTTATAAGTAATACATCCT
CCTGCCTTTGGCAATCTGTTCAGATGTCACTGCTGACTGTGCGCTTTAGGTTTGTTATTGTGACTATGGGCCGAAGGTGGA
CCGTGTCACTGTTATTCCCTGAGTTGAGCCTTTCAGGCAGCATTAAAGTCCTTTTTCCTCTAGAGGACGTTGAGTTGCCTC
GAGTACTGTAGATACGTGACTTTCCGCCTGCCCCTTGGATCATCTATCTTTATCTTCTTTTCTTTTCTTTTAAACATCAAC
GTGAGAGGAGGTGCTTCCTGAAAGGTTTAGTTCTGGGAATAAGTCTCCCACTTGTTGACTCATGTGTGGTCACTTGTGAAT
GTTGTCTGCTCTCTAATGGAAGAGCAGGGTAATGCAATGACTGTTAATGTAATAAAATCAGTAGGTTTCCTCCCTTTATCA
GCCCTTGTTGTGCACACTGCGTAAATAACTGATAAAATCTTGGATTAAACACGTATTCTCACCTGATCTAAAATGAAAACA
ACACCCTAATCTTCATTCATGTGTTAACAATAGTTCCTCTGATATTTTTATTGCCTGCTGTAGCATCACCATAAACTTTCT
TTACATCATTTTATAAACAAAAATGTTCTGTAGTCGCCCTCAGGGTACATCGCTCAGGTATTTATGTTCCTCCACTTTATC
GTCCCCATGCTCACTTGGCTCTGTGGAGGAGATAAGATTATCGTCATCCTCAGGTGAGAGAAAAGGAGGAGTGTGTCTCCC
GCCCGCCTTTCTAGATTGTCTAGATTCCAGACTCGGTGAGGTCTGAGGTTTTAGCTGAAGTACGTGTCAAACCTCCACTGT
GCTGATTAGTAAGGTGGGAGGAGGGGGGGCGACTGTTGATGAGAAATCTTATCAAACCAGTTGGGCAACACTGACTTTCTT
CACAGCTGGAGGCAGGGTGTGGATTTGTGGTGACTGTGGTGGTGTGTGTTGAATGTTGTGTGTTATTTCTGGATCTTTAAG
GGTCTTTTCTTCTGTGTTTTTTCTTCAGGTTTCTTTAGTCAATGGGACTGCAAAGGCTCCCTTTCTAGGGTGAGTGACCTA
ACACACTAACAATAACTCACAGGTTTTCACTGCGTTCTGTCAATGATTGAAGGTTGTTTGATAATCAGCAGATGATTTTAA
ACATGTAATACCATGATAGTGATTTTCTTCCATTTTCTCCCTGCAGAGGAGGGAATCCTTTTGCGACGGTCAAGCTGAGAC
CGACTGTCACGAATGACAGATCGGCGCCAGTCATCTAACGCCGAGGAGGAGGGTTTTCACCGCTGTCATGCCTTCAGGGGA
ATCATGCAACATGTGACCCCTGCGACACACTGAATATCGCCCCAACCTGACATCTCACCAGCCTGCATGCTAGTCTCTCTC
AGTACCAGTCCGCTCTATCAAAGGGATCCATTGTTTCTTGGCGTGTTTAACTGTGCCAGTGTGTGTTTTACATTAGCCACA
ACAGGGAAACGTTGCATAGTCATTACACGGAGAAGCAGGAAATCTGAAAAGTTTTGCTGTCCAAAGAAAATGTTTTGTGTT
TGAGTCGATGTTAAAGTAATAAGTCATCCCCAAAATGAAAAGTTAGAGTTTGTCTTATCACTGAAAGTTGTTATCAAATTG
TCACATACTACTGTGTTTCCAGTGGCTGCTTCACAATAAAAGCCACCACGTTTCAACAGCTGAGCACTTTAAATTTGAAGC
AGTGAAGCAGTGGCTTAATACAGATAACACATAACTTAAAGGTGCATACGTGTCAAATTCATACTCAAAACAAATTGGGGG
CAGCATATCACCAGAGTAACCGCTAACTGCTGCTAACAAGAGTTTCCATTAGCTAGTAAACTCAGTTAGTCGTGCATCTGT
GGTCCAGACCGGGACTTTGGAGCACCTGAGGAGTGTTGGAGTTTACTAGCACAGGAGCTTTCGACCGGGACGGGCTGGGGC
TAGCTGGTTAGCATGCTAACTTATTCCAGTTTCCACGTTCCCACTAATACTAGTTTCTCCTGATGCTAACAGTGAGATAAA
AACCTGAATCTTCATTTTGGGGTGAACTGTTCCTTTAAGATTGCTCAGACACTTTTCAGGCCCGTCTGTGCTGCAGCCAGT

GCAGCGTTTGAACACAGAATGAAGAGCTCCAGATGTAAACAATAGTGACCTGATGCTGACGGGAAAAGCCCAGCACCCAGG
CTGTCTTCCTGACTTGCTAAAAAAACAATATATCGTCATTTCTATTTTTATTAATCATTCGTGTCTTTGTTTTTTTAAGAG
GCATTGTGATATACGAAGGTTGTGAGAATGAAGGGAGAGTGACGTGTTGGGGAAAAAAGGGTTTGTCATTCGAGTGAAGGC
ACAACAGTATGTTTAAAATGAGAAGGCTATTTATATACAGTACGTTTGTATGAGATATTTAAATAAAATACATATACAGGA
GTCCATTCTGTGAAGGTGCCCCTATGAATACGGTAGCCACTGTGCTCATGATCCCACGCTGATTTTGATTTGACTGCTGGT
ATGTATTGTAAATGGACAAAATGACCTTTTGATGAAGGGAATATTTTTCGCTCCTCATGGTTTCCCTGTTGTTTAATGTAG
ATTCTGTGTACGACGCACAGTTTATATGCAAATTAAAAATGTACCGTTACTGGTGAACACTTGCATTGTATGAGTAAAGTG
GGTCATTGTTTGAAATCGACAAAGTATTAACTGTTTTGTGAGGAAAATGACTTTTTCTGCCATATTTTGCTTTAAAGAATG
TAAATGTATGATGAGGACCTGATTGATGAAGCAGTGTATTGATCCTCAGAGACCCACTGATGTCAAGTGTGAAGCCATCAC
ACTGACATAGGTAGTGCCTCATGCTTTCCTCAGAGTAGCTTGCCACTGTGTTGACCAATGTAGTCTTTTTTTTTCTCAATA
AAAAAGGTAAAGTAAAAGTGTGTATGATAAATGTTCTGTGTGTTGTCAGTTTATCCCTCTCGGGCTTATGTAGTTGGTTGT
TTTATGTTTTATGTGTGTGTCCTCAGTATGTTGGGGTCAAAACTCGATGGTCAGAGTCCCTTAAACTATAAAGTAAAGTTT
ACCTTCTGCAATAAAACTGACAGTTACACATTATGACTTCTTAAAGATCCACAAGGTCTTTTAACATTTTAAAAGGGTATA
ATATTTTAGGAGGTTAGGCCTTTTAGTGTTTATAGAGTATAAAAGCAGCCTTTTATATGCTGCAAAATGTATATGAACGCT
ATAAACACTATAGTAGATGAGACATTGTTGCTTTAGTCACCTTCTGTATGTGGTTCAATTTTGTAATCCAGCAGTAATAAC
TGTAATCCCATAATATCATCTGCATTTGTGAACAATTAAGGAATAAGTGTGTCACAAATTCTCCAATTATCAAAGACTGTA
ACTGTAATAACTCTCTACGATTAGCACAACATCCATCTATTGTGAACCACAGTAACACGTTATAATATCCACCATTAACAA
AAGATACATTCATAGTTAATTAAACTTAAGTTAATGGTTAACAATCAATTAAATGTTTTATGAACCATTTATTAAGCAATT
ATAAAGGTTTATAAACTTCAGTTGCAGCTTTGTAATGGCCATCCAAATGAAGTTTATAGATGAGCTACACATTATTCATTA
ACCATTTACAAAGTATTGTAAACATCAGTTGCAACTTTACAATAAACAATAGTTTATAAAGCATTTTATTGTTTGTTAACA
GTGAAATTATTATAAACTAAAGTTTAATCAACTGTAAATATACCATTTATTGATTAAAATAGCAAAAGATTAAAACTCAAA
CTGTTCTGAAACATAATCGTCTGTATATAGGCTATTTTTGCTATTTCAGAGGCCACGTGTTTTCATGTTTAATTTCTACTA
TGTTTGATTATTCTGAATATAAAGGCTGCCCTTTAGCAGAGTGAACACTAGCCTGATAGGTCATAGCCCAAAATGAAAGCA
GTGTCCAGTGTAGCTACGTGATTACAAGTCTTTTCAGAGAAAGGCCTGGCAATTAGAAAGAGGAAGAGATGCCAGGGCAGA
TAGAAAGACAGATACTTAGACAGACAAGAAACATCTTCTTGTCTATCTCAGTATCTGAGAGGAGTGAACAGCTTCTCTTCC
GGGTGTACATTACGACCTGACCCTTGTGAGCGCCATGCTGTGCTGCTTTATGCATTAATAAAGTGTGACAATAGTGTGAGA
GATTTTCTTTTGTTTCTTCTGTCAGAATTGGAATTGCAAAATTGTCCCAACAGACAAGATGAATTTTCCTGCTTTTCTGTT
TGAACCCTGCAGTAACAGATTGTTGGACACTTGGGGGCAGCAGAGACTGTCTGTACTCTGAACATTGACCCACCGTCACCT
TTTAGAGGATTTGAGGAACTTGCTGTTGATTTTCACGTGAGGCAGACACAGAGCAACATGAGCTTTCATTTTGACTTGTTT
TTGGATTTAAGGAGGATTTCACAAGCTGATGAATGTACGTCCAACATTCACACGCTCTGGTCTCCACTGAGCCAACTCCTG
AAGGAAATATCTCGAGCCTCATCCAGCTCACAGCCTGGACAGACTGCCGGGTTGTGAGCTGGAGTATTGTTTGTGAAAAAT
GGTTTTGCTGTGAAAGGTGAAAAGACTTTTTTTTTTTCATGTTATTGAAAAGATGATTAGGCCTTTGTCTAGCCTCACAAC
AACACAACTGGCCACAGCCACATACATTCTCTCCAAAATGCTGGTGTGCCACTTGATGTTTTTTAATGAATTTGGTCCTTT
CTTTTTTCAAAATGCAAGAAATATAGGAATACATGTAAAATAGGAATTCTTACATTCTAGTCAAAGATACTCTCATGTGAA
TGTTAAAAGTCTGTGGACCTGAATCCTCCTGAATGTGTTTGTTGTGGATCTAGTGGGAGCAGAAGGAGACCCAACGAGAAG
CACAGCAGGCAGTTGTGGCCGAGTGGTTAAGGCGATGTACTTGAAATCCATTGGGGTCTTCCCACACAGGTTTGAACCCTG
CCAACAACGTGGTGACATCTATTACAATGTGAAGCTGACTTCACCAGTGAAGTGTTGTACCTGAAGCAGCACTGTATAGGA
GAAGTATGGGGCTCTTTGTCCAATAAATCATCATTTCTCTTTTGGTTCTGCAATCTCTACAGTAAAGATGCACATTGACTA
ATAGTTAGCCATCACATTTTACTTATTGTGGCAGTCAACGGATTTTAATGGGAATACTTATTTATATAAACTTGTTCCATT
GAAAGTTATTATCCACAGTAACCTGGACATTGTTGATGTAGAAATGTCAGTGCTGTGTCAGAGGTGATTTATCTTGATTAC
AGTTTCACAACTTGAATTTTACCCAACGTTTTAATATTAAACACTCTCCACTGATATTGTGGAGATGTGCTGTGTCATGCA
AACAGTGTAGGGGGATGTAGCTCAGTGGTAGAGCGCGTGCTTCGCATGTATGAGGTCTCGGGTTCAATCCCCGACATCTCC
ATCTTTAAAAGTGCTGGTATGTTAAAATACTTAATTGTACTGATGAAGTGTTGCATGTGGCCTCTTACTCAGTGGATCAAT
CACTGACGGTGAGATGCTCTTAAATCACAACTAAAAATTGTGTTGATTCCCTCCAACAGTGTTTCTCTTTAATAATAATAC
ACTGACATGTTGGTATTGTGCATGGAAGGTTGATCAATAGTTCTTCTCTAATTGAAAGATGGCGTTATTAACTGTTATTTC
AACTTGTCTTTCATTTGTATTTTACAGCTTGGAGAAGATCAGATGGAAGATCAGATGGAACAGCATGCCATGTGAGCCCAC
GCCTTTCAAAAGGTAAATACACATTAATCTGATGTTATTATCAGTACTCTGATATTGATCAGTAGTTCTTCTGTTTGCCTC
ATTGCCTCTTATCTGCTGTTTGTCTTTTAGAGAGACAGAAGACGAGCTGAGACGCGACCAGAGAAGAAGGAGACAGGAAGC
AGTTGTCCACATAAATCTGGACTCTGTCAAAAAGATAAATATTTCTAATATCTTGTTTCTGTAGAATCTTAGATTTAAAGC
AAAGGTTGCCTAAACATTATAGTAGAGGCACACGAAACAAGAAATGTGTGCTCACTGTCTTTTCTGTGCACTTACAGATCA
CCAAGAAGCTGTCAGGCCTTGACATGGGGACTGACCACTTTGTCGCTCACATCTGAGAGTAAATGTGAGAGAAAACATTCA
CCATCAGGCTGCTGTCAGCTGAGGAGTGTGGCACTCTGTCTTTATGGCATTTTTGTGCACAGAGGATGCACAATCTTGGCA
CCCCATCTTCATGGCCAAACTATCCACAAGTTCCTGCGTCAACACAGGTTGGCCCTACGGCTGTCCCCCCACCTTACTACA
GGACAGGAGACTAAATTAAATGCAGTCAGTGCCACCAGCCTCACAACAACACAGCTGGCCACAGCCACATACATTCTCTCC
AAAATGCTGGTGTGCCACTTGATGTTTTTTAATGAATTGGGTCCTTTCTTTTTTCAAAATGCAAGAAATATAGGAATACAT

GTAAAATAGGAATTCTTACATTCTAGTCAAAGATACTCTCATGTGAATGTTAAAAGTCTGTGGACCTGAATCCTCCTGAAT
GTTTGTTGTGGATCTAGTGGGAGCAGAAGGAGACCCAATAAGAGGCACAGCAGGTTGTTGTGGCCGAGTGGTTAAGGCGAT
GGACTTGAAATCCATTGGGGTCTCCCCGCGCAGGTTCGAACCCTGCCAACAACGTGGTGACATTGATTACAGTGTGAAGCT
GTCTTCACCAGTGCTTGAGGTGTTATACCTAAAGCAGTGCTGCATAGGAGAAGTATGGGGCTCTTTGTCCAATAAATCATT
ACTTCTCTTTTGGTTCTGCAATCTCTACAGTAAAGATGCACATTGACTAATAGTTAGCCATCACATTTGACTTATTGTGGC
AGTCAACGGATTTTAATGGGAATACTTATTTATTATAAACTTGTTCCATTGAAAGTTATTATCCACAGTAACCTGGACATT
GTTGATGTAGAAATGTCAGTGCTGTGTCTGAGGTAATTTATCTTGATTACAGTTTCACAACATGAATATTACCCAACATTT
TAATAATAATAAACAAACACTTGCTGATTATTCACCAGGTGTACCTGATATTGTGGAGATGTGCTGTGTCATGGAAATGTT
GTATAGGGGGATGTAGCTCAGTGGTAGAGCGCGTGCTTCGCATGTATGAGGTCTCGGGTTCAATCCCCGACATCTCCATCT
TTAAAGCTGCAGGGATGTTAAAATACTTAATTGTTCTGACTAAGTGTTTAAAACTGCATGTGGTCTCTTACTCAGTGGATC
AATCAATAAAAGTGAGATGCTCTTAAATCACAACTAAGACAAGTGTGTTGTTTCACTTTAACAATGTGTCTCTTTAATAAT
CATCCTTTAATCATAATACATTGAAAACTTCACCTTCTCACTTAATGTATGATGGTATTCTGCAGGGAAGTGGTAAATATG
CATTTAGGCAATTTGTTTATTAATTGATTCCTGTATTTATCTGAGGGTAGTATTTTTTAATTTTTTACTATTACTAGTATT
ATTATTATTATTATTACTATTTTTATTATTATAATTATTATTAATGCATATTCTGAACTTTGAATGGGAGCAGCTGTAACA
CAAACAATTTCCCCTAGGGGATAATAAAGTATTTCTGATTCTGTTTCTGATTCTGATTTACTTTTATACATTCATAATAAT
GATGAATAATCATGATAATTTGCATATGTCATTAAAAATGACATATGTCTTGATGGATAAATCATTTGAAGCTCCCACACT
ACGACAGAGAGAAAGACAGACACAGAAAGTCACAGTCAGAACAAAGTGATGTGTGTTTAAAATGAAGACGAAGTGTCCTCC
GTGTTGTCACATTGTTTCATTTCAGTCAGTTGTGTCTGCAGTGCATCATTTGACATCTGGCTTACACAGCGGAGTGTTGAA
TCCTCATTTTAATCTGCCGATGTAATGAAAAAACATGTAATTGACCTCATGTGCTTCCTACGGAAGCCTTAAAGGGCCATG
CATTCATACATTTTATTTCATACATTTTCCCGTGATAACGAGTTAATTTCATTTGTTTTCTCGAGATCTCGAGTTATTATC
TCGAAATAACAACTGCCGGTTTCCCGAGATAACGGCAATATTTTCTCGAGATCTCGAGATAACGAAACTTTGTTTTCCTGA
GATAACAATATAATTAATTCGAGATCTCGAGATAACGAAACTTTGTTTTCCCGAGATAACGATATAATTAATTCGAGATCT
CGAGATAACGAAACTTTGTTTTCCCGAGATAACGATATAATTAATTCGAGATCTCGAGATAACGAAACTTTGTTTTTCCGA
GATAACGATATAATTAATTCGAGATCTCGAGATAATGACTGTGATATGATAGGCCTGAGATTGTGGAGACGCCCGGGACCT
TGTAGCTGGTCAGCCAGTTAACAACGACATCAGGCTCACTTAGATTGCGCCGCCGATATAGCTGAAGCCTTGCTAACCTCT
TTTTAGATTACGCACACTAATGTGTATATTATCTGTAATAGCAAGGCATAATGCTATTTCAGCCTGTGTCAGGCCTTGATT
GAAAAGATATGTGATGCGATGATCGTTATGCTCCTGCTCCTCTGACATTGCTACACTGAATGATGTTTCCTGCAATGATGT
AATATACTACACTATTGTTTTGTTTTCTCAAGATCTCGAATTAATTGTATCGTTATCTCGGGAAAACAAAGTTTCGTTATC
TCGAGATCTTGAGAAAATTATCCCGTTATCTCGGGTAAACGGCAGTTGTTATTTCGAGATAATAACTCGAGATCTCAAGAT
AACAAATGAAATTAACTCATTATCATGGGATCCAAATGAATGCAGTGTGTTTTTACAGGGAGCTGTCAGTTTCTGTCACTT
GTCTCCTGTATTTACTGAACACCAGTTCAGTTGTTGCACATAACTCTAAAAATGAATGCAATAATTCATAAAGCTAGAAAC
ATAAACTACAGTAGCCTAACAATGAAATCTTGTTTCACTATCCCAGCGTGAACAGGTGCATTGAAAATTGACAATCGATTA
TCAAAATTGATCATTTATCTCAATCAATTGATGAGTAATGTTTCATCTATTATCAGGATTAAAGATGTTACATCTGAAATA
TAGGCCAAGCAAAATCTTAATTGATGTTAACCTATTATCATCATTATTAGTAGCTTACATTCTAGTCAAAGATACACTCAT
GTGAATGTTAAAAGTCTGTGGACCTGAATCCTCCTGAATGTGTTTGTTGTGGATCTAGTGGGAGTAGAAGGAGACTCAATG
AGAAGCACAGCAGGTTGTTGTGGCCGAGTGGTTAAGGCGATGGACTTGAAATCCATTGGGGTATCCCCGCGCAGGTTCGAA
CCCTGCCAACAACGTGGTGACATTGGTTACAATATGAACCTGTCTTCAACAATGATTGAGGTGTTGTAAAGCAGCTCCACA
CAGGACATTTATGATGCTTTGTAGAAAGCTAAATATTACAGAGTAAGTTCAGGGGCCCATCTGTGATAAACAAACCCTGAA
TCCTGTTTGTCCAGTAAACCATCATTTCTCTGTTGGTTCTGCAATCTCTACAGTAAAGATGGACATCATTAATAGTTAGTC
ATCACACTCACTTATTGTGGCAGTCAACGGATTTGAATGGGAATACTTATTTATTATAAACTTGTTCCATTTAAGGTTATT
ATCCACAATAACCTGGACATTGTTGATGTAGAAATGTCAGTGCTGTGTCTGTTGTAGAGAATGTATACAGCAAGTGTATAT
GTTGAAGCCGAAACGCTCAGAAAGTACGACACAGAGGCAAGGTTTGTAGAAAGTGGGTTCTGTTTATTCCTGGACTGACCA
AGCATGTATATGTCTCGAACTCTATTCATCTGTTTACACAATTGTATAATAATGACCAGGACCATCACCAAAATGTCACGA
TGATCTTTGCCTCATTTCCTAGAATTTCATGTGCTTTCCAGTAATCTTTCCAGAGCTTGTGTGCGTGTGCGTGTTTGCTCA
TGCATGTGTGTGTGTGTGTTTATCCTTGTTGACTTGACCATAAGTGCTGGTTTGAAGTGCAGGCTTCCTGTTTTGCACTGT
TTTGGAGAGTACTGGCATTTCTATGCGGTGTGGTTGTGCTTATTAGGTGTGGTGTCATTTGTATTTCCCAATCTTCTGTTT
ATCTCAGACCCCAAAGCAGAATAACATTTTCATATTCAAAGGTCAATACCGGTCACCTAGTCAGGCCAGATTTCTCAATAT
AATCCCCCCTCTGTGGGTTTACAAATCCCACACAAAACTGAACAACGTGCGCGAGGGCGAAAAAACCTTTGTTCTTATAAG
GATTAAAAAATTCCCCCACGGCCCCCAGATTTGCTTTTACTTTAAATGATCTGGACGACCACCTAAGGCTCACATTGTTTA
CATGAGTTGATCATACAGTGGTTGTACATATTGCATATACACATTTTGTTTGTTATAAATGTTATGTAAGCATGGCCAAAC
ATTACTGTTAGCGTTTGCCTGCGGTATGCAGTTATGTGTAACTTAGACGACTATAAAATGGAATAATATAATTCAGAGCAT
AGCATTAAAACTTAAATGACATATATGGTACGAAAACAACAACGTTTGTTCGATAGTAACATTTTCGTCTTTCGTTATTCT
TCGTATGAATCCAGTAACAGTGTTAAATAGGGAACATCCCAAACTCAGAGCTTGCAACCTTCTCATTCCTTCCATACACAT
ATTATAGTACTGATTAATTAAACACTTCTTGACTTAATATAGACTTCATAAGTTTGACAAATATTAAGTCATCATGTCACA
GTTGACTCAATATATAACTCGTGCACATCAAAACTTTGTTTATGGCAGGTTGTAAAACAGAATATGGTCCATTATACTGTC

ACACAAAAAATCGAACATATTTCAACTATAATTTGTTGACTAAACTTCAGAATATACATAATACATTTTTGGTAATACTGC
TTTTGTCCTTTTATACTTTTTGATCAGTCCATTCGATGGCAACTACTACAATGTCTTCATCACAGGTATCCAGAGGTCACA
GCACAAGCACACGTTGAAATCCTTTAATTCTTTATTTCGAAGTTCAGTCCTTGCTCACATTCATAGAACAATTATACCTTT
GATTCACTGTCCAAGTGCATGTAGTAATCAGGTATTGGGAACAAATTTGGATGTTCGATCACCATTTGTGTCAGCTGAGCC
TCTGCCACCTTCGTAGTTGCCATCATGGACATCTGTCAGGCAAAAGCTTTTGTAAGTACATTTCTCAGAATGGGTATGATG
CAACAAACCAATAAAATGATTATAACCAAGACTATTAGAACTGTTGTTCCTATTTTAGCAAACACTGCACCCCACTGCCCA
AACTTTGAAGCAAACCAATCCCCTGATTCTGCACTCCCTCCGGCATTTTCTGTTACTTCCTGTCTTAATTTCTTAAGTTTA
GACATTGCAGCGGTGAATGATCCACCTGTAGCTGTGTTTTCAGGTATGTAAGTGCAGCATTTTTCTCCAAATATGTGACAG
ACTCCACTTTTCTCAGCCAATATCCAGTCCAAAGCTTGTCAGTTCTGCCAAGCAACCCTACTGGTTTCATGCAACTGCTCT
CCAAGAGCTTTGAAAGCCTCAGGACGGCTTGGGTGGCCTCGGGCGGCTTGCCCTCCGTCGATTGTTGCTCCTGCGGTTGAC
CCTTGGATTGCGCCTTAGTACAGCGATTCAGATGGTGCCACGTCGTGCTTCCCTCCACCTGCACGGCTGTTGGCGTTGCCC
GTACCACAGTGTAGGGACCCTCTCGTCTTGGTTGATGCCACTTTCTTTGAAAGAACTTGATGTAATTTCTTATTTGTGAAC
ACACCACTTAGGCAACAGAGTAGGCTGGTCAGACTTTTACTTGATTCACAATGGCTTTCTTCAACTCACAACTTTTGTTTG
GAGAAAAAACAGATCACAGCGTTTACTTAAACATAAACCAGCTTTAAATGCATTTGCAACACAATTCTTTAAAATATTTGG
AATAGCAGCTTATATGTAATTATCAAACAATGTTATTGATCACTGGCATATAGGTATACTTAAACGCTTCAGACAACAACT
GTTATTCAACCGCCAATAAGACAGCATGCTGAGAAGATCTCGAAAAATCACAACAAAACCTCGCTGTACTCAAAAGAGAAA
AAAGGAAAAGTTCACTGAGCTCGGACACAGTCGACAGTGAGGGGAGAAGGGAGGGGGACTGCGTCACACCGGACGTGCAGT
AGAGTAGGGGGGCGCTGGGATCCCCTTAACACACACACAACAGGACACAGACAGTGGAGGGGCAATGGAGCCGCAGCTCAA
AACACAACTTCTCAACACACAACTTCTCAGCCACCAACGAGTTAGGAACTCGCTGTTAAACTAATACAAATCCCGCCCGGG
GATGTGTCTCTTCGGCTGTAACCTCAACTGACCGTCACTTGGATTCCTTATCCCAGCAACACTTTAAACATGCATTTAGAA
ATTCTTAGCCTACATTCATAAGCCAAAAACAACACGCACATGAACATAAATCACATCCAATATTAATATATCTATTAGCAC
ACACGTCTTTAAACCAGAACTATCACGGTTGTCTACTAACCTTCCACCATGTTTATCTACTGTAACATGCTTAACCACTTA
TTGACAGTAACACAAAGCTATTTAAAACAGCAGAAATCTCTCCACCATCTGACAGTTATATCAGCACAAAATCACACAACA
TTGCGAATAACACAATTCATTTGAGAGGACACAAATGTTTCCAGCAGATTGTTCAACATACAGTCAAGACACAACCACATA
CACTACTTCTTCACTTTATGAGACTCCCATAGGAATGTTCGAACATTTATTCTTGGTTTTCCTGAAATTGTTTTGGCTGGT
ATTCTATTTTGTTAACAACTAACTTTTATTGTTGTTTATTTCGGTTCAGATTGTTCTTTTCTGCAGTTAGATCATCGCATG
TGTTTACTCTTACTGTACTTAATTTGCTTTGATTTTGGCTTATTTATCTAATATTTGCTGTCCATGGACTTAAGGCTGTAT
TTTAATCTGCATCTCCCTGTCTTAAAATACCCTTTTTCTTTTAACCTGATTTGTAACTTATCCTTTTTCTTAACCTGGATT
GTCTTTACCTTTGTAAAGTACCGCCTCTATTTTGGAAGTCCATGGACTCAAATTCCTACTCTAATCTGCATCTCCCTGTTT
TAGAGTTGTAATTTACTTTAATTATCTTCCAATTTGCTCTATCTTAGAGTCTTTAAGACTTAAGGATTTGTTCCCTTTTGG
AACTTTAAGTCTTTTCTCTTGACACTACTTTTACAGCCCATCGGCTCAAAGTTTTCAGCTTTCTCTTAAAGCTTTAACAAG
AGACTCACTCTTCCCCTCCCTCTTGGAGCTGTAGGAGATTCTTTAGTAAGAGTTTTTATTTATTTATTTTTTTAAATTGCT
CCCAACGTTTTAATAATAATAAACACTTGCTGATTATTCACCAGGTGTACCTGATATTGTGGAGATGTGCAGAGTCATGTT
GACAGGGTATAGGGGGATGTAGCTCAGTGGTAGAGCGCGTGCTTCGCATGTATGAGGTCTCGGGTTCAATCCCCGACATCT
CCATCTTTAAATGTATGTATGTTAAAATACTTAATTGTACTGATTAAGTATTCAAAACTGCATGTGGCCTCTTAGTCAGTG
GATCAATCACTAAAGGTGATGATGAAGTCACAACTAAAAATTGTCTTGATTCCCTGATTTAATAATAATTCACTGAAAACT
TTACCTACTCACTTGATACATGTTGCTATTTTGCAGGGAAGTGGTGAGGGTTTTCAAAATATGCATTGAGGTAATTTGTTT
ATTAATTGATTCCTGTATTTATCTGAGGGTAGTATTTACTTTTATACAATAATGAGTTGATAAAAATGACATATGTCTTGT
TGGATAAATCATTTGAAGCTCCCACACTACAACAGAGAGAAAGACAGACAGGAGGTCACAGTCAGAACAAAGTGATGTGTG
TTTAAACTGAAGACGAAGTGTCCTCCGTGTTGTCACATTGTTTCATTTCAGTCAGTTGTGTCTGCAGTGCATCATTTTACG
TCTGGTTTACACAGCGGAGTGTTGAATCCTCATTTTAATCTGCCGACGTAAACAAAACAACATGTAACAAATGACCGGGAA
ATGATTAATTCTCAAAATAGATCATTTTATCTCATTCAATTGATGAGTAATGTTTCATCTATAATCAGGATCAAATATGTT
AAAACTGAAATATAAATTGAAAATTGCAGTCAGTGCCACCACCCTCACAACAACACAACTGGCCACAGCCACATACATTCT
CTCCAAAATGCTGGTGTGTCACTTGATGTTTTTTAATGAATTTGGTCCTTTCTTTTTCAAAATGCAAGAAATATAGGAATA
CATGTAAAATAGGAATTCTTACATTCTAGTCAAAGATAGTCTCATGTGAATGTTAAAAGTCTGTGGACCTGAATCCTCCTG
AATGTTTGTTGTGGATCTAGTGGGAGTAGAAGGAGACCCAATGAGAAGCACAGCAGGTTGTTGTGGCCGAGTGGTTAAGGC
GATGGACTTGAAATCCATTGGGGTATCCCCGCGCAGGTTCGAAATCTGCCAACAACGTGGTGACATTGATTGCAGTATGAA
CCTGTCTTCAACAATGATTGAGGAGCTTTATCTGAAGCACCACTGCATAGGAAAAGTATGTGGCCTTAGTTCTGGGACCCT
TCTGTGATAAACATTTCTAAACACATTCAGTTGTGGCAGTCAACAGATTTTAATGGGAATACTTATTTATTATAAACTTGT
TCCATTGAAGGTTATTATCCACAGTAACCTGGACATTGTTGATGTAGAAATGTCAGTGCTGTGTCAGAGGTGATTTATCTT
GATTACAGTTTCACAACATGAATATTACCCAACGTTTTAATAATATTAAACACTTGCTGATTATTCACCAGGTGTACCTGA
TATAGTGCAGATGTGCTGTGTCATGCAGACAGTGTATAGGGGGATGTAGCTCAGTGGTAGAGCGCGTGCTTCGCATGTATG
AGGTCTCGGGTTCAATCCCCGACATCTCCATCTTTAAAGGTGCCAGTATGTTAAAATACATAATTGTACTGATTAAGTGTT
CAGAACTGCATGTGGTCTCTTACTCAGTGGAAGTGAGATTCTCTTAAATCACAACAAAAAAATGTGTTGATTCCCCATACC
ACAGAGTAAATATGCAAGACTTTCCAAGTAAAGACCTCTATTGTTTCAAGTTTTTGTTAAGTTTTGTTTCAGGATAAGTTG

CAACTTAATGTTCATATTATAAGCATTTCTAGTCAGCCAGGTAACTGAAAACACCTGTGAGGATGTGCAGGGCTTTAAATA
CATAATAATGCATCATATTGTGTAAGCCATGTTTTGTATGTGATGTAAAAAAAAGTGCAATATTTCCCTCTGAAATGTAGA
AGAGCAGAAGAATGAGACAGGTCTCTTCTTCTTCTTCTTCTTCATGGGTGTTTGTGGGTCTACTTCATGGATCTATAATAA
GATCCATGGTCTACTTGCCTCCCTCTGCTCAGTCTCGGCTCTGAACTATCAGCCAGCAGCGCCCCCTGCTGCTCACAGCCA
CAAACACCAGCCCGCCGCTGAAAACAGCCAATAGGAGCGAAGAAGCGGGCAGCACTTCCTGTTTCACGTAGCGACAGGAGA
CGTTCAGATGTGTTCTGATGTGAACACATTATGATCCAGCTCAGCTGTTCGCTCGTCTCCAGCTGCTGAATGAACAGCGGC
GACAACCCAGCTGCGCCGCGTCCCGGTTTTATGAGAGGAAGCTCGAGGTTTGTGTGTTTGAGCTCCGCTTCTGTTTGCTAT
TTCCAGAGAGCTAAGCCTGTAAGCCCGCACCAAAAAAAAACGAGCTAAGTGTTGTCGTTTAAGCCTAAAGCTGGTTTTACT
GTACAGGGTCCGCTTGTTCGCAGCGTTTAGCTGACATTACACTGTCCTGTCAGTCACACCTGCTGTGTATTAGCTCCTAAT
TAGCCGCTGAAGCTCGCTACGTTAGTACAGAGCTGATACCTTGGTTTAACTGATACAAACCTACCTTGTTATACAGTAACA
AACGTCATAAACACACATATTACTGTGTATTTTACTCGACGAATCGTTATATAGACACCCCAAGGTGTACTGTATGTTCAT
TATTATTATTATTATTGTTATTATTATTATTATTATTGTTATTATTATTATAGATACGAGCTGACTGGTGGTGAC
AGAGGATACGCCTTTTAGCAGCAGCATCCCAGCTGGTTACGCAGTGTTGTGAAATGGAGACCACTGTACTGTAAGCCTTCA
GCAGCAGCTTCATTGTGCACTGTATTAAATGTCTTAAATGGGCTTAAATGTTGTGATGATGTGGATGTAGAGTCCGGGTCA
GTGACCGAGACAAGCCAAATTCGGTGTAAACAAACGAACAACAGAGTGAGCGTGGATCAGAGCCACCTGGACACATGACCC
TGATTCATGAGCACAGTGGGATTTTTTAATATTGAATTAGAAGTTACATGAGCAGCCTTTCAAAAACAGTACCCACATTGT
GCTGTAGATAATCTAATAAAAAATATTAGATTCCCAGTGAATTTTCTGTTTTAGTGCACTGGCGTCGTTTTAGATAATGAG
GTTTATTGTCAAACTGTAAAATATCCAACCCTCCTTACATAAATACACAAGTGTTTAATGACCACATCCGAGGGATCGTTG
CAAAAAGTCTGTGTTATCAGCAGATATATTGATATTGGACTTTCTAGTCCCTAATATTGGTATCGGCATCAGTCCCAGAAA
TCAAGTATCAGTCGGGCTCTAGTCATCAGTGTCATCTGTGCATTTCCTGCTATCTCAAGTCAGATTGTCGGCTGTACCGTG
AAGAGGAAGTTATTAGTTTTGATGTGTACTTTCTCATTTTAGCACTGTCATTATTTGTGTAACATGCTGCAAATGTATGTT
TCTATGCGTGTTATTTAATCACAGAATCAAATCTAATGCTTCCACTCGATTCTCTCTCTGATTCTTCTATTAATTTATCTG
GCCTAGTAAATCTGTTTTGTGGTCTGTGTCAGCCTCTGTCCTCCTTAACCTTTCCAACCCTGCTGACCCACTATTGGGTCC
ATTGTTGCAAACACCAATGTGCTGACAAGCATTTGTAAACCCTCTGAACCTAATTTTAAGATTCAAGACTTTCAAAGATTT
GACAATTTGAAATGTGTATAAAATGATTCACCTGTCACATGCAGTGATGCAGCTGTAAATGATGGTGTCGCAGCGATTATC
AATCAATAATTTGTCAACTATTGACTAAAATCTCCAATTATTTAGCTAATTGAACAATTGATTTGAGTAATTTTGTTAGAA
AAAAAGTCAAACGTTCTGATTCCAGCTTCTTAAATGTGAATATTTTCTGGTGGTTTGTGTACTCCTGACAGTAATCCAAAG
ATCTTTGTCTTGTGGACAAAACAAGACATTCATGGACATCATCGCAGACTTTGGGGAACACTTTGATATTTTTTACCATTT
TCTGACATGCTTTTGACTACAACAACTTAAAATTTAATTGAGAAAAGAATTGACAGATTACGTTGAAAATAGCCCTAATCG
TATTCGTAAATAATTTCACCTAAGATACACTGGAACAAACAGATACAGATTAAACACCTGATATTGTCAGGTGTTTAATCA
AACATTATGTAACTTTGAAGCTACATTGTCTTCATTCTCCTCCTCCTGATGCCCAGGTTTCTGCTGCAACCATGCCTGCCT
CCAGGCTGTGGGTGGTGGATGTGTACGGGCGGGTCTTCAGCCTGTCGACGGCGGGGCAGCAGTGGGAGCAGTGCCATGACA
CCCAGATGGAGTTCAAGCGGGTGACGGCAGCTCAGCAGTGCTGCTGGGGCATCGCCTGCGACAACAACGTCTACCTGAACC
TGCACGCTTCTGACCTGCCCATCCGCTACCAAGAGACTACCTACGAGAATCAAGTGGGTGTCTTCCACCAATCAGGGGTCA
TTGTTTAGTTTGCTTCATGGAAAAAAATGTTTTTGAAAGATGATGATTTTTCAAGTCAAGGTTAAAAACACACAGAAGGGA
AGCTTTGGTGTTTCTTCTCAGTATGCAAGTTAAAAACACCCTTATGTCTCTGTGTCTTCATTTCTTGCACAAACTGAAGTA
AAATCGAAAATGTTTTTGTTCCGCTGGACTTGATCTACGTGACTTTGTGACTTGCTCTATGTGCCACATCCACGTAGAGTG
GTGTTGCACTTCCTCTGTTGCTAACCTCAGCACGCTTTCAGCTGTGTGCCTTTACAAGCACATTGAGACAGTTTTTCAGCA
AATTACCAGAGGCAGTGAAAGTAAAACTGTACGTGGAACTTAAATGGCAGGAAAAAAAAATACAGAGTAGATTCAAGTTTCC
TGTGACTGATGGGAAGCTTTTTGTACCACCAGGTTGTTCTTACACAGCGGTGTCAGCTTATGTGTTTGAGTTTACTACTGT
TGTGGTTGTTCTTGTGACTTCCTCTCTTGGGTCAAGGTCACCTAAAGTTCACTTTTGGCAGTAAATCTTTCAGACACAATG
AAATTAGGAGGGGAGGCATTTATATTGAATTACAAGGGTTACTTTTACTTTGTTTGTTTTGTCCCTGTGTGTGCTCTAACA
TGCCATCTCCATTCATCTGTCTTTCTATCAGCGATGGAATCCAATCGGTGGTTTCTCGGACCATTTATTGCCAAGCGACCG
CTGGCAATGGAGTGATATCACGGGTCTGAAGCACCAACCTTTCGACAGCTTCGAGCTCCCCTCCAGCAGCTGGGAGTGGGA
GGGTGACTGGTACGTGGATGAAAACTTTGAGGGAGAGCCCACAGAGAAAGAGGTCAGTGTCTAGTAAGGATCATTTCTGTC
TTCTTTTAATCCCTACCTAGTTGTAATGACTGCGGTGTATGTGTCTGCAGGGATGGACCTACGCCATTGACTTCCCCGCCT
TCTACACCAAAAACAAAAAGATGACCTCCTGTGTCCGTCGCAGGCGATGGCTCCGCTACAGGAGGTACAAAGACATGGACA
CCTGGGCTAAGGTGGGTGTATTGTGTGTGTGTGTGTGTGTGCGGCATGTGTAAAGCTTTCAGTTTGTTCACCTGTGTCTCA
CTTCCTGTGTGTGCTGACAGATCCCCTCACCGCAGATGGAGACGCCAGATCCCTTCAGCGACATTAGCTGTGGAGGCTGGG
ATATCAGTGAGGAGCCCAGAGGGCGGCTGTCATTATGGGCTGTCTCCCTACTGGGCAAGGTACACACACACCCACACACAA
AGGTTACCATGGCAGTGTACTTTAAGTCTTAATCCGCTTATGATACGCACAAATTAAGTCAGTGACAGAGTGGGAACACAG
TATTTTACTGTCCTCTTCATACATACTGCAACACATTTTTTTCATCTTTTTAATCAACTGTATCTACTGTAATTCATTTGG
CTTTTCTAATATATATTTTTTTTAAATTATATACACGCTTGCACACAAATACGAAGGAAGAGTTTGTGTGTATGTTACTTT
TGACCTTGTCGCTGCCCATGGCAAGATCCCACACTTCGTCTTGTCACATCCGATAAACATTTGTATTTTCACTGCTTGACC
CGGATCACTTACTGTCCTGCTGTCACTCTTCTCTCCGTCCAGGTGTGGTTCAGAGAGGGAATCTATCACCACAATCCTGAG

GGCTCCATGTGGGAGGAGCAGTCTGTACCTGGGGAGGTGCTCCAGATCAGTTGTGGCCCAAGGGATCTGGTCTGGGCGGTG
CTGTGGGAGGGGCAGCTTGCTGTCAGGCAGGGCATCACCCGAGACTGCCCCAAAGGTATCACGGCTCACTAGTGGGAGGAG
GGTTTGGTTTGTGTGATCATCTCTATTAAGGATAATATTGTCTGCATGTCAGGTACCTCCTGGGTGGAGATGGATTCTCCA
AGTCCCGATGGAGGAGCCATACATGTGGCCGTGGGGATCAACACTGTTTGGGCTTTGACCAAGGACAACAAAGTAAGAAAA
TTCCGTAATGAACATTACAAAAGACACAAAAGTGAAACCTCTCCCAGCTATCATTTAATAAAATGGTTATCTTAATTACAT
ACTTTACCATTACCATTAACACACATCTCTCTAAACTGTTTTAGATTCTACAACATTCTACAACACTTTTAAGGAAAGGAG
AAAACCCAAATAGCATAATATGTCCCATTTAAAGCCTAAGAAATAAAAGTTTAAATGTAAGAATTTTCTCTGGCTTCACTA
CTAAAACCATCACACAAGTTATGGACAAAGTAGAATTTCCCTAAAATATGTTACTATAGCCCTGCAGTAAACAAACATATG
GTACAGCTAAAAATACAAAACAGATAAAGAGGAACTGGCTGCTCTGGTAAACTAAGGCTGTACTAGTATTAACAATAGTGT
TTAATCATATTTCTGTTATGCTAAAGAGCCCTTTTCCTCCCACTCTCGTGCTCTGCCAGGTGTGGTTCAGACGTGGCATTG
ACTCCCACAACCCTTGTGGCTTGGGCTGGATCAGCATGGTGGGAGAAATGATCACAATCAACGTAGGACTCAATGACCAGG
TGAAATACAGCCACATGACATCACATCCTGTATCAAACTGTTACGCATCAAGTACCATCCTCTCAGCTGGTTGACTTTTAG
TGTCAAGTCATGGGATGTGGACGTATTTAAGTCAACCTACTGCTGTTACTGTTGTGTGTAGGTGTTTGCAATCTGCTGTGA
GGATCGGGCCGTGTACTTCCGACAAGGAGTGACCTCCAGTGAACTGAGTGGGAAAACCTGGAAGGCCATCAGCGTCCCACG
AGATGGAGACCGATCACACTCCAGTGCCAGCGCAAACAGCCTGCAGAGGTGGAGAGGCCTCAGTGAACACACACACTTAGA
CACACATGGTGATAGTCAACATGTTAGGGAGAGGGCCGATGACATTAGTGGCGCCATCCATAACCACCTGAAGATGGAGGA
AGCAGCTATAATAAATTCCAGCTTCCAGTGCAGGCTGTCATGTCAACATATATGTGATGTAAAGTGTGTAAATAATAAAGT
ATAATAATAATAATGATAATGAAGTAACTCAGTTCCTCATTCTCTTCCTCCATGTCACAGTGCTGGATGCTTCTTCTCTGG
TGAGGTACGTGCTCAGTCAGTGGTGAGCGACGTGGAATCAGACACGGAGAAAGTATCCACAGATGGAGCCAGCTGCCTCGT
AACCTCCACCTCCCCTGAGTCTGTTGTTGATGCCCCCACAGACCACCCTGACCAACCTATGGAGACCCCCAAACCCCGCAT
CCCCAAGGTCACCAGCGACAGCTTCATCTCTGAACTCATCTCTGACCGAGAACCAGCCAAGAGCTTAAGGGAGGGCGGATC
TGCTGGTCCAGCTGTTCTGGAGGAGGAGGAGACCCTCCCCGGTGAAGGAGAGGTCATAGCCCCTTGTGCTGATGTAGCTAT
ATCCCCCAGCCAGTCTGGACGTCCTCTTGACGCCCAGTGGAGTAACGTGGATCTGGAGGAGGCGCAGGGCCAGCAGGCTCA
GACTGGAGTAGCACTAGAGGCAGCTGACACCAGCAGCTTGTCATCAGTGGCCACATACACTCTGGCCATGGAGGACCCGTA
TGGGACAGATGAGCATCCTCTGTGGGCGTGGGTCAGTGGAGGAGGCTGCTCCGTGGACAGTCATTCCCACCTCAACTGGTT
CAACTCGATGAACACTTCATGTGAGTTAAATGAGACAGATTGAATTTAGGCTGTTCTGGAGGTATACGACTGCTACAACTA
CAACCCATGTATGAAGTGGTGTAACATAATTAGTCAGTAACACCTGAGTCCCCGAACAAACAATAAAAGTGACTCTTCATG
TGACAGCTCCAAAGTCGAGGATAGCATGATTACATTTTAATTCCAGTCCTGCAGCAAATTTTGATGTCAAAACTCAGTTTG
TTTGCTTTTCTTCTCCCAGTTATCAATTTTTCTCGTTTGTCCTTCCCAGTGTTGGTCCAGTCAGTCCAGTCTGTCCAATCC
ATGAGTCCGTCTGTCACCCCAGCTCAGACAGCTGCCTGGCGGCGACAAATCTTTGAACAACTCAGCGAGAGGACCAAAAGA
GAGATGGATAATTTCAAACATTATGAACAAGCTATAGAGCAGGTAATGCAGTACAGAAACAGCTGTGACTGTACTATGTAT
TTGTTCTTTTTGATTTATCTCTTCTTCTGTCTTTGTGTGTGGTCTTACCTCTCTATTTTATTCTATTTATCATTAATTAAT
CATTCCTCTGCCTGAAAACTCCCTCAATGCAGTCTGTGTGGGTGAAGAAGGGAACCATGCAGTGGTGGAGGGACTGGAAGC
CATACAAGTGGATAGATGTTCATTTTGCCCTGGAGCAGTTCTCAGGATCAGACGGCAACAAGGACGGCATTCTTTTCATCT
ATTACAACTTTTATGAGGAGAAGAAGGTGAGTAAGAAGTGAGTGAGTTGGGGAGAATTTTCATATTTCTTCTTGAGCCAAA
TAATTCAGCGTCAAGGTCAGATAATGCTTGTTAACGAAATAGAAAGCTCTGGTGATGGATTGAGTCATTCAAATGATTCAG
TACTGCTGTTCCTCTCTTGCACTCACTACTGCTTTTCTCATCGTCTTTCTTCCTCTATTTGCTTTCTCTTTTACTTTCTAT
CTACTTCTATCTCTCCCTCCCTCTCTGATGTCACTCTCTCCAGTACCTGCATGCCTTCGTCAACGAGCTCACCATCCTGGT
TCCTGTGCTAAATGAATCCAAACACACTTTTGCCATCTACACTCCTGAGCGGACCAAACAGAGGTTTCCAATCAGATTGGC
AGCAGCCACAGAGCTAGAGATGCATGACTGGGTATGTATGGATACATCGCAGCATTTTCAAAAAAAATCGTATTATTACCA
TCGTGCATTTTGTCTCATCTGACATGAAAACTGAAGCCCAGCTCTTGCTGTCATGTGCAAAGATGTGTATTATACTAAATG
AATTGTTGCAGTCTCAACTTGCACCAAAATTTGTACATTGTTTCAGTCAAGGACCTTCACCAGACAGTAGTGAGGCATGAA
ATGTGATAAGTCAGTGCCTCTTAATAAACTGAAGAAACACTACATCAGTCTTCGAGGTGTTCTACTGTAGATTCCCACATG
ATGTACACACAGATTTCTGTGATTTCCTTTTTATTGCTTTCTTTCTGTCTTTTCGAATCACTTCTGGTTTCTCCCTCCCTT
CCCGACACAATTTGTCGACCTCCATCTCCATCCCTGTCTCTAGCTGGCGTTGTTGAGCGTTTCGTGCTGCCACTCCAGGGG
GATCCAGGGTCCTCCCTCCAAACAGGCCATCTGGTCAATCACCTGTAAAGGGGACATCTTTGTTAGTGAGCCCTCCCCTGA
TCTGGAGGCCATGCCTTACCCCACACCCTGTGACCAGATGTAAGAAAAACATTTCACTCATGTCAAGCAAAAGTTCTGATG
GATCAGGATGTAAATCATTGGAAGTAGATAAATACTCTTGTGTAAATTCTTGCAGACAACAGCAGAGCAGTTCCTGTGCTT
AGCTCTTTGCTTTGTGTTGGCCCTCGCCTAATTAAATATATCTGGTGGTTTCACACTTAGAAATGGTGAAACTCTGTGGTC
TGTCGTTAAAAATATCCAGTGAAAGCTGTGTTCTTTGTACCTTTCAAAACTTCATAGAGCACCGGCAGACAGTGAGGTGGG
CGGTAGAGGCGTGAATCTTCACTGGCTTCACGATTCGATTACAATTAAGCTGTCAACGATTAAAATACACAATGATTTTCA
ATGCATCTCACTGTATGGCATCATCAATATTTTTATATTAAATTCCCCTTTAATTTTGAATAAGTCTTTCCTGTACAAAAA
GCTACATAACACACTAAAGGAAAGGGAAAAAAACAAATACGTATAATACAAAATTGAAATCTTTTTCCAGGTTCTGGCGGC
AGGTTGGAGGTCACCTACGGATGGTGGAGTGTAACAGTGTTGGCATAGTGTGGGGGATTGGCTATGACCACACTGCCTGGG
TCTACACTGGGGGTTATGGAGGAGGTTTCTTCCAGGGACTGGCCAGCAGCACAGATAACATTTACACACAGACGGATGTCA

AAAGTGTCTACATCTACGAGAACCAGCGGTGGAACCCTCTTGAAGGCTTCACCAACAGGTAGAGTTCTCGCTCCAGACAAC
ACTGGGATTCAAGTACTTCTAGAGCTGGAAACTATGAAATACATGTGGTTAGATTTTCTCAAAGTACTAAAATATTTGATT
TCCTTTTTTTTTCTGTTGCATAATTGCATTGTTTCAAACAAAGAACTTTTCAGGCTAATATGTCTGACTGTATGATATGTT
AAGTGTCACTGTCAACATTGTGTCTAAATAAATAGAAATTTAAAATTTAACAACTGTGATAAAATTGCAGACTTAAAATTT
GTCTTTGTGCTACTTTTCACTAACATCTCATGAAAACAAAATGTGACAAATACTGTAAAGATGCCACCTGTCACTGCAATT
GTGTCTCTCTAAACATCTTAATCTTTACCACTGTGATAGACAAATATAAGGCAAACATTACTACTGCAGCTGAAGTACAGA
TGTTTATTTGAAGTGTCAATGACTAATTTAAAGTGCATTTGGTTGATTTCAGAGGGCTACCTACAGACCGCTACATGTGGA
GTGATGCATCAGGACTACACGAGTGCACAAAAACAAATATGAAACCTCCCTCCACCCAGTGGACGTGGGTAAGTACAGCTG
CATAGGGAAATGCCCTGTGGTGATGTGAAACCAGTCATGCGGTGCACATATTAACCCTCCTCCTTTCATTCTGGCCATTAC
TGAGGAAACTATAAATATTTGATCTTGAATTATCACTGACAAGGTGTATAGACAAAAAGACAGATGTGTTAACAGTGTTTC
ACTTCCATATTTGCTGTTTTCAGGTGTCTGACTGGACTATCGACTACAGCGTCTCCTGTGGGACAGACAAGGAAGGCTGGC
AATATGCAGCTGATTTTCCAGTGTAGGTTTTATTCATCAGTTGTCACCATTGTCACTTGGTTTAAACCACTCTGTCGTCAT
ATTTATGACCTCCATTTATTGGGGTTTCTTTATCGTTCTTCCTTCCTCTTCTCTATGTCTCCATGCAGGTCATATCATGGC
AGAAAAGGGAAGACGGACTTTGTGCGTCGCAGGCGATGGGCCAGGTACTATAAAAAGCAGGCTTATTTAGTCCCTCCAATG
TATTTGTTCAGGAGGTTCACAGTGGTCCCAGAGTGCACTTGACATTTTCCGTTTTGGTTTGTGTTTTTGTGTAGAAAGCCA
ACACAGACTTAGCAGAGGAGAAAGAGCAGAGTGTTAGACAAGGAGCACAATAACAATCAGTGATATTTCTTTTTAGCTTTA
CAAACTGTGGATAATATTAGTGTTGTATAAGATTGTATCATAATGTACAGGTAGTGTTTTCTATTCCCATACAGGTGACCG
TAGTCCTGGAACATGTGTACTTGTCATGTTGGCCTGTAAACAAAAGTGTGTTTTCTGGCAGGCTGGCTAGTTAATTATAGC
TGCAGATTTGGACTCTGAGTTATCCAGATGAGAATGCATGAGTCTGGATTTTTTTGGTTCTTCAAAGCTGGTTTAAACTGA
ATAATGATTTTAGATGAGAGCATGCAGGGTATTTATGTAATAAGAAATTACACTTTCATAATGAAGATGTATACATAAACA
TGAGTGAATATAAAATAAAATTAAGTTTTAGAGTCAAAAGATTTTGTCAAAGATTCGGACTGTTAAAAAACGGATGAAGTG
GGAACATATGGCTTTTGATTTGACAATAAAATTTAATTTCTTCTATCAAACACTAATAACACTCATAAAATGCTGCTTTTT
TAAAAGATTTTAAGACTTAATTTTAATACTATTCTTATTAGGGCTGTACATTTAATCGAAATATCATTAAAAATTAGTATG
GGTCAGTGCATCCAAATTAAAAGGAACTGCAATTTTTTTGTAAAATGTGTGACAAAAGAACATCATAACGAAGTACTGTAGT
GCTGCAGGGATATCCTGGCCTTCAAATCCAGATGTAAGAAAACATGTTTGTTTGGTACAGACTCCAACGAATGTCACACCA
TCGTGATTTTAACAGTTTTTTCAGTGGAAATGATAATTGTGATGCAAAAATGATCATTCCAAGTATTTGTGACTCTTATCA
AAATTATTTTTTGACTATATTGTGCAGCGCTAAATCTTATGATCATCACGAAGAGGTCTCTTGTACATTGCCCGTTTAAAA
CCCGCACAAATCAAACACCAATATCACTGGTAGTCTGACCATATTCACTGATAATCTCAACAGTGATCACTGTTTGGAAAC
ATTGTGGAGTGTGGTGTAGAACAAAATAACAGCAGTAACAATGCATGTATACACATATACCGAGTCCATGAATCTATTGCC
CCAGTGCAAGCTGGGAGGGGTCCCAACACACCTGCCTCGTGTACCTGCTATCACATGATCAACGGTAAATCAATTGTATGA
ATACTGATCATGGATTCAACTCTCTGTGCCTTCATCCTTTTGCTTCTGCTTCTTTGTGTTTGTTTTCCTGCACAGGAAGTG
TAAACTGACAACCACAGGACCCTGGCAGGAAATTCCGCCGGTAGCACTGAGTGATGTGACCATCCTGCCGTGTGCAGCTCA
GAGCAACGTGGATGAGGTTCATCTGTGGGCCATCAGCAACAAGGGAGATGTCCTCTGCCGACTGGGAGTCACCGCACTGAC
GCCTGCTGTGAGTCACATTTAGTGTTCACGTGCAGATACCCCATGAACAGGTTAAACACCACTTAGTGAGCCACCCATAAA
CATTCAGAACCATTTTCTTTTTCTCACTGCAACTGTGCACATGTGCTTCAGGCAGTGTGTGAAGTCCTTTCCTTATCTTAA
GTCTTAGTGGAGGCTTTTCACCGTTACCATTCACATTTTTGATACAAGAGTCCAGGAGTAAATCTTATTGATAAGTATGCA
AAGATACTTCCAGTGTAACTGCAACAAAAACAATTTTAAAAGTACTTGGGAGGTAATTGTTCGAATCCTGTCTTATTGGTC
TCTCAGGGATCCTCGTGGCTCCATGTGGGAACTGACCAGCCTTTCAAGTCCATCTCCATCGGGGCTGCCAGCCAGGTTTGG
GCCATCGCCCGGGATGGTTCTGCCTTTTACAGGGGATCTGTCTCTCCACAGAGCCCAGCAGGTCAGTGGGAGGAACTGAGT
CGTTCTTCAGTTTGATCATCATTCAGGCCACTGACAGATTTTAATCAAAAACATTAAAATGTACTTGAAAATTAGCAGAGA
TGGGGATGACCCTGATGAAGGCCACAAGCCATACACTTCGTTCTAATAATAAAGCTGTTTTTTATACTACAAGAGTTGCTG
GAGCTTTTTGACCTCTTCAGACTTTCTCCCTTCTCCCTTCATGCACCTTGGCAGTAGGGGAGGTTGTGCCAGAAACCCCTA
CTCCACTCCCACAGAGTTAAAGAAACTAAACATGTCAGCCTCTGATGCTGCAAATACAGATTATGTGTTGTCTCCCTCTGC
AGGAGACTGTTGGTACCACATCCCCTCTCCATCCAAACAGAAGCTGAAGCAGGTGTCTGTTGGAAGGACATCTGTCCTCAC
AGTAGATGAAAATGGTAAAGTGGTTTAATACCTGATATACCAGCTTCTTGGTGGACTAATTTTGATCATGTTTCTAATGGG
ACTATTTCCTGCTCAGGTAACCTGTGGTACCGGCAGGGTGTGACCCCCAGCTACCCTCAGGGCTCCTCCTGGGAACACATT
TCTAATAACGTACGCAAGGTCTCCGTAGGGCCTCTGGACCAGGTCAGTACCGGGGTGTCAGTACTGCACTTTAACCTTTAG
CCCTTAGCTTTTGTTACTGTCTACATTAAAGTAAATGACAGATGTGGATGTGACAGCCTACTCTATGTGCTCTATGTGCAG
CTTTGATTTTTTTGTTTTTGTGAAGCCACTTTTATCAACTCCACTGAGTTGGTAAAGCATTTGAACCTTGTACATCATTTC
TGGGTATTGATATTCAGAATTGACATGAATTGGTCCATTTTTGTGATTCGGAGCTTCTGACAAAGTAAGAAAATGTGTAGC
TTTAATGAATGATTCAGAATACATAGAAATACAATACAATTTATGAACAATCCTTGCATGTTGTTTGGTTGATGATTTCTG
TTGTTTTGCTGTTGTGTAGGTGTGGATCATAGCGGACAAGGTGCAGGGCAGCCACAGTCTGAGCTGTGGAACAGTGTGTCA
TCGACTTGGAGTCCAACCTGTGGAGCCTAAAGGAATGTCTTGGGACTACGGCATTGGGGTAAGACACCAAAGAAACTAATC
TGTACGACAGTGTTGTCAAGGTATCTGCAAAAAGTCTTAATAAGATGAGATTAATTGGCTTGTTTTTTGATTGCAAAAGTG
TTAACAGTGGATTATGTTGCATTATGATTCATTTTTGTGCTTAAAGCCAGATAGTGAATAAGTTGACCTTGTACTTGCCAA

CCATCCAGATTTTCCCATGAGACTTGGGGGGGGGGGGGGGGTCTCAAGGTTGAAAAGTATAGTTGACTTGCCTGATTTCTC
CCTTCCTCTCTCTTCCTGTCTTTTAGGGTGGCTGGGACCACATCACAGTGAGAGGGAACTCCATGGAGCCGCCTCGCGTTT
GTCTTCCCTCTTTAACAGACACGTCCGCCCCGGCCCCTCGGAGCCCCCTCCCTGTCAGGAACATGGAGGTCGACAGCAGTG
CTGGGGGCAGCTAGAAACACACATCGGTGCTTACAGTCTGTACACGGACATCCAAACCATGTTCCCTTTGTAGTAAACCTT
TGTAGATAATTCAGCTCCAAAGTCAATTCAAAGATGAAGTTGTGACTTTTTGACACTGAGCTGAATCTGTCTCTGAATCTG
TCTGAATATTCAGTCTTCTGGTCTCATGTTCATTTAATAAGCTTTAATCTCCTCTGTACTGGCATTTCTCTTTAATTTTTT
TGCTTGTGTTTGTTTTAGGTCACTAACACTCAACTTGACGGTCTTAATGCTTTTTTTTTAATTCATTCATCAGGATGTGAT
GTTTCTGTCGGTTTTTGTGTATTGATTTTCATATATACTGTGAAGATGGAAGGGATGTTTTTTAAAATATATTTTATGTTT
ATTTTTAAGAAGGGAAAAATGTGTGCACAACTGATTTGCTGATAAAATGTCAACTCCTGGGCTGGTAGTGTGAACAAAATA
AAAAATTACGTTACATCTTAGCCAACACTTAAACGCATGTTCCAACAGAGTACGGCCTGATTACTCTGGAAACCAGAAGCA
AGATTGATAAGTGTATAGAGACAATGTTTAAAGGTGCAATATGTAAGAATTTGCCACCTGTTGAATTCATGCTCAAACCAA
TAGGGGGCAGCATATCACCAGCGTAACCACTAGGAGCTCGGGGACCAGGGTTAGTGTTTACACCACTAGTACAGGAGCTTT
GGGCCGTAGCTATATGACTAACATGCTAACTCCAGTAAATATCTCTGCAAAACAATATATAGACTTCATAATGTCCAAACT
GTTATTTCCTCCAATTTTGTTGATAATTTTTTAACCAAAATACTTACATATTGCACATTTAATCCAGGCTTGGATGGCCCC
TTCTATTGTCTGATGCTGCTGTAGAAGTTCAACCACAGACCTCAGAAAAACATGTATGCCACATCTGTTGGTCTCTAATTA
TGTAGGTTTCTGACAGGACTTAATGACTTCTTCATGTTTAGAGCCAGAATTTTTTAAATTTAGATTAATGTGTTGAAACGT
GTATGAAGCTGAGCTTGGATGGGTGGGAAGCAGACTGAGTGTGATAGGCTGTGACAAGCACTCCAAAACAAACTTTCAAGA
CTTTCAAGTCTGTTTTAGTTATCCAACTGAGACGCTGAACATCTCGTGAAAAGCAAATATTGACTTTTTGGTCTCTTAGGG
AATTTATGAAATCGATGGTTTTACCCTTCAACACAGCTCGAGTGTTTTTTGGAGCCATCGTCTGTAGCAGCTGCAACAAAA
CTTGCACCGGCGTCATTCACCTGTGGTTTTTATAAGTTTTATTGTTTCTTTTCTCTAAAAGAATGGTTTGGTTTTTTATGT
CATTGTCTTGAAGTACGTGCAATGGTTAGCAATGTAAATAGGAGAAAAGTCAAGTCCTTGTAAAAGTCTTGTGGTTGAAAT
GCTACAACTGAACCTTCCTCTTTCTCAGGAGAATGTTGCATTTGTGCGCCGCCTACTTCCTTTGACTGATGATAGAGTGAC
TGAGCAGGTTTAAAAAAAAAAAAACTATATTTTTGTTATACATCTACAAGATATTAGTATTATACAGTGAGTAGAGTGGTGA
TATATTAAGGTGGAGTTGTGTTTGTATATTTGACAAAGAACAATTAATTAAAAATTCACTTTTCCATTAAGACATATTCAT
TAACCACACGCTGATGTCACGCCATGTCATAATGTGCTGTCTAAATAGAGAGAGAGATCTGTGTGTACTATCTGTGAATAA
CTAACACCATACTGCGTTACTGTGGCATTGTGGAGTACATTTTTTCAAATGTTGTGCATGGTCAAAATAACAGTCCTTGTC
AATAAATAAACGTGTTTATTGATAATAACCCCTCAAATAGCGCCTCTCCTCTTCTTCCTCTTAACTACAACGTTCTGCCTC
GAGAAACAAAAAGTCAAACAACACACACTTTAAACATCATCTGGGCCTTCCAGCTACACAGACAGTTAGAAAACAAGTTGT
CTTCTCATCTGAGCAAACACATTTTATAGCTTCATAATTTCAATATTTAAGCCCTCTGCTGCTTGCCTGCTTTTTCTCTCC
TGGATGTGACAGTTACGACTAGAGGCTACGCTGAGCGGGTTTCTCAACAGACAAGAAAAACACAAGAAGATCGCAAGATAA
CACGAGAGAGGATGACGAAGTTCCTGCTCGGGCTCGAAACCATTGAAGACAAAAAAAATAAGAGACAGAATGGCGCAAGAT
TAGCCCTTCCTTTCTGAGCACAAGAACAAATTTCACTCTTCACACAACACATCTGGTTTTCAGTCTGCAGTCTGTGTGTCT
GCTTGGTCCTCAAATGCCATATTTGGATGAAGTTCTCTTGCTATGGTGCTGTGGGTTTATTGTCAGAGGTCTATGATCTGG
AACAGCACCCAGTTCCAGTTGAGATTTGGCCCTCAAAGTCTACCCACGCCTCGGAGACAGCCTCCTGGTGGCTCTCCTGGT
CTCCAAACCACCTTGCAGCCACTGTTAGCTGCGCTGGCTCAAGCTGTGCACATGGGTGAGGTACTGGGCTAGATCATCCTC
CCCGTCCTCCTCCAGGGTCTGTTGGTAGCACTGGAGCAGCTTGGCGGCACTGGCCTCTGACGCGGCCCCGCCAGCAGGCAG
GCAGGCCCCTGACATGTCCAGGATGATGGTGGTTAAGGATTTGATGTAATTCTTTGCCAGGGTCAGAGTCTCGATCTTGGA
CAGCTTCTTGTCTGTCTTGACGTGGGGGATGGCCTCGCGCAGCGCTTGGAAGGCGTTGTTGAGCTTGTGCATGCGCTGACG
CTCTCGCTCGTTGCTCTCAAGCCGCCGGACGCTGCGTTCCTTGTTGCTGGAGCCGTGTTGCCTGCGTCGCCTGCCTCCACC
TGCTTGACGCTTGCCATCCCCACCTCTGAGTGACCCCCTCCAGGAGCCACCGATCCGGACAGAGGCCTCTGAGCCCTCCTG
CTCACTGGAGCCTGGTTCTGTGTCTGGTTCAAGTTCTGGCTCGGGGTCAGACCAGGTCCTCCTGGATGCTTTCACAGCCTT
CCCTTTGGACTTCATCCTGCTTCCTCCTTCACCTCTAACAGTGGAGATCTTAGAGTTTCAACAGGCCTGGAAGACAAGATG
GGAACGTTAGAGAGAGGAAGGAAAATCAGACCTTTTTTTAACATGTACTTTTTTACAGTGGTCTCCCAGTGAGCCTGTAAT
CCCCGTTTACTGGATAAACCTCTCTCCAATTCAAACAGCCTCGCATCCTGTTGTATCTAATCAGCCTGTTGCAGTGTGAAA
ACGCCATGATGTTGGTGTGTTAATCTACGCCTCTCTTATCCCCACGGTCCTGCGATAATGCTCATTTCAAGGTTACACTCA
CAAAAACACGGAGAGCAGCCAAGCAGCACTCAGATAACTCACCTCCACGGCTCTTTGGAGTGCATTTAAAGGTGTAGTTTT
GGAGAAGAATTTTAATACTTACAACATTAATGAGGTATTAAAACAAACTCAGAAATATCTTTTTTCCATAACTGATTAACA
AGCTGTTCTCAGAGGAAAATAAGGTGTAAAAATAACACTGTTTGAAGCTAGAAAGGTGGCAGGGTCCGCCACATATAAACA
AAGTAAAACAGTATGAACTTGTGCTGTCCTTTAAGGTCAGTTTGTTTAGTCAGTTAAGAAAACAAAGAGAGTTTGTTTATT
TAGTGTGTTTAGGCATAAAAAACAGTCATTGAAGATCTTTCTCTTCTAATTAAGATGTTTAGCTTAAAATACTGCATGTGT
GAAAGTCAGTATGTTAAGATATAATGTGATTTACACACTTTACTTTCAACCAATGATCAAATATGCTGATGTGCTACAACT
GCTAAAACGTTTCAGATAGAGCTGTTTTACATAGATTAGTGCACACAGTGAAGAAGAAAAAAACGGTAGTGCAGTGACTGA
TGCACACACTGCCACCATGAGAGTGAATGAAGCAGGAGGACATGATATAAAAAGAGACTTACCTTGGAAGTTCAGCTAACT
TTCCTCCTCCTCAGTGTTTTCACAGAGAGGTCGTCATCACGCAGCCGGTCATTCACAGCACCATCATGTCAAGTAAAGAGC
AGCCAGCAGCAGCAGCAGCAGCAGCAGAGGCAGCAGCGTCACATACACACACGTTCAGATCACTGTCACATGTGGGCTACC

TGTAGCTGACAGATGTACACGTAGCGCTGCTCAGCCAATCACAGAGCCCGAACACTCCCTCCAGCTCTGCGCGCTGCCCAG
ATGTCCCAACAGGCTGCTGCTGCTGCTGCTGCTGCTGCAGTGTTACAACACTACAACTACTGTATGATGTAGACTTAATGA
TCACTGCACAGTCCACATGGGGTTTGAGCAAAAATGGGTTGTATCAAATAGAAAATGTTTTTTTCGACCCTGTTATTTGAT
AGGCTGGTAAAAGTAGCCATAGTAAAAGTCCTGCATTGAAAATGTAGCTTTAAAGTACAGTAGTAGTGATGTAGTGTTGTA
GTGTTAACTAATGTTAGCTGCTTTAGGCTGGATGATGTTGGATTGTTTAAAGGTGCAATATGTAAGAATTTTAGTTTAAAA
AAGTTAAACTTAAAAAAAATGCTAGAATGATCAACACAGAGTGAATAAGTAACAGTTGTCATGTCATATCTTTGTATTTTG
TCGCAGAAGTTAGCATGCTAACCAGCTAGCTCCAACCCATCCAGGTCTAAAGCTCCTGTGCTAGCAGAGTAAACCATGGAC
GTTTAATAAGACCAGGATACAGTGTTGGTTGTGGAGCCACGTTCAGTCCTATGAAAGCTGCTGAGTGGCGTTTGAAGCCAA
AAAAGTACAACTTCCTGGGTATGAAAAATCGTGTTGCGTGGCCCATAGAGCGGTTTAGGCCCTGGCTAGCTTGAATAGGAA
TAAAATGATTTAGTTTTTGCTGCACTTCTAGACTTTCGGAACTTTTTTGGGCTGAATGGATCAAATCATGATAGTGAAACA
AGTCATTTAAAAAAAATGTAGTATTTAGTATGTTTTTTTTACAGGATGAATCTGGCCAATTCTTACGTACTGCACCTTTAA
CATGTAACAATGCATCATAATTTATAAACTGATCACATGTTTTGTGACCGTACATAGTTACTTTTGTGCAGATAATCAGCA
TAACAAGAAAAATATAAAAAATGTAGCTATACTAGAAACATCATCATATAAGCACCATCATATTGTTAAAGTCTCCACTGA
AAGACATTATAATAAAGATTGGTTTATGTGATTTGTCCCTTTTCAAAAGAAAATGTAGGAAGCTAAAAACATTTCCCAAGT
TTTGAAAGGATAAATGTCTTTATTGATGTACCAGTGGATGGTTAACTGGTATCAGCCCGTATTGTGAAAAAAATGTCTTAC
CATTTGAAACCACCACTACCAAATGACATTTTTAAATGTTTTTAATGTTTGGTCATTTGTCACATGTTTACTTAACTATAC
TATTTTATTCTTTCAGTGTTCCTCACTAAACCTTTTCATCAGTTTGAAGCAGCAGAGGCGAGCTGATGTTTTGGGCCTCAT
CTAAGTATAAGGCAGCTCACAGAGCCAGAAACACTTTGTTTTGGTTCCTCGTCCACTCCTTTAACTCCAAGCAGTTAACAT
AATTTCCATTGTGGCTTATCACCTTCCACTGCTTCATTTGAATACAGCCCTCCAGGCAACACCCGAATGTACAGAAGCAGG
GTCACGTCTCATTTAAACTCTATCACATTATTTACTTTTTACCAGACTTTTAAACTTTTTGCACTGTACTGCTGTTTTTGC
TGTAACATAAGCCACTGGATGCTGTCAAACTGTAAAGAACAAGTAAAACAAACAAGGACAGACACAATAGTAACTTAATTA
AGCATTTTTGAATATTCCCAACAAACACCTTTAACACTTATTTGCGCGATCTGCAGTAATTCACCTCGTCTGTTTGACCCG
AGGGCCCAACACGTTGTTGGCAGTTGGGATTATTGCCCTTTCCTGCTGTGATAGACAGTGAAATAGAGATAGGGTGGTGTC
ACTGTGGTGGCAGTGCGATACACTGAATGACCATTCATACAGTTAATCATGTTTCACAGGATGTTTTATATAGAGGCATTC
AGAAATACCCCACAGTCTTAAACAATCAAAAACCCAAATTTACGAACATCATCTTACAATTAACATAAGATCAAATGCAAT
TCTTGAAAGTAAAATTCTCTGAACAAGTTAAAATAAAATAAAAACAAATAGAACATTTCTTTCTGCATCTGTCATTTTGGT
TTTTATTTGTTGTCAATCTGATTTTTATCTTTTCCTTTTAAATGTTTCAGAAATGTATATAAATATTCTAAAAAAGAAAAG
GTTTTTATGCAATGGTGGGAGCTAGATAGCGCTAATCTTCTTCCTATGCGTTTTTATCACTTTTACAATAATTTCTGAACA
CGAAGTACGTGACTAAATCAAAACACATTCCTAAAATGTATCTCTTTAACAAATTTAAAACCTTTTTTATCCAAAAACATA
AAGTTAAGGAGCCACATTGACATACTGAGCAAAAGCCACAGGTAAACTAGCAGCTACCACCTAGCAAGCTCAGCTTAGCGA
GAAAACATAACAACAGTGAGTAAAATATTCAATGTAATAATGGGGGGGAAATGTGAGTTATGTTTTTGTGTGTTCAGTTCT
GCCAAAATAGAGTAATGATAAATAATACAGATGCTTTTCTTTTTAATGCAGTGTGACATTAGCTTAGGAAGGAAAAAGAAA
ACATGTAGCCAAGTAGCTTTAACTTCATTGAAACTCGAAGACACTTAAGTAAAAAAACAAACTAAAGTAGGCTTAAGTTAA
GATAAGACATGACCCAGTAGTATTTTTATGAATGAATGACGTCAAAGAACACTTGCAAAATTTGGCTATTCGCAACATTAG
CACTGAACAGGCTAACATGAATGTATAACAACAACAACAATAGTGTGCAGGTGTGAAGCTCCCAGCGCTTAGAGCAACAAT
TTTAGCTTTAGGTGTCCTTGAACAGCACGTGTGTGTGCGTGTGTGCGTGCACGTATGTGTGCGTGCGCATGTGTGTGTGTG
TGTGTGTGTGTGTGTGTGTGTGTGTGTGTGTGTGTGTGTGTGTGTGTGTGTGTGTGTGTGTGTGTGCTCGTGAACAGGT
CCTGCCAGGTTTCCTTTCCTCCTGCAGGTACACTGTATCTAATCAACCAAACCTCGCTGATGCTGGCTCTGTGGAAACACT
ATCTATTACTTTTAGTTTGTTCAGTTTCTGCCTCCATTGGCCGTAAGGCTTACATTGTTTATTTAGTCACAGCCGCTGATA
AATTCTGAGCTGAATACACTTCAGTAATGAGACAAGCAAACAGGGCCTCCACAAGCACGCTCTGTTTGAGCAAAACAAACT
GTATAAACAACATTGTATTAAGATTGACCTGGAATTTGCTCAACACAACAATTTAAAGATGAAACAGGATTTCAGATACAC
ATGACAATTTATTAAAAACATGTACTTTGACAACATTTCTGTTTCTATTTCACAGCTCGGGTCCAAGGTCACAACCTGGCA
GCTTCCAAAGAGCTTTGCAGCTGGGAAGGTACCAGCACTGTTATGCAACTGGCTGTTTTCTCACATCATAGCCAGCGATCG
TGTCATACCAACAACTGTGAAGGCTGAATGCACCGTCGCACAGCCCTGCGTGGCTGTGATGTCATGAGCGACAGGTGCTTT
GAATCCATTTGGAGCAGAGAGAGGGAAAAAAATCCATTAAAATCCCTGACTCATCATAAAAGAGGAGGGAGGTGGGGAGAT
GAGTTCGAGGTGAGGAGGACGAGATCTGGCCGATTACTCAGTTTTTCCATTAGGTCAAGGCTGGCTGTCTGATAGAAAAAC
AAGTGTGGATGACACATTAGGCCACGGTGGCGGCAGGATGGCCTTTGGCTGAAGGTAGACGATGTGATAGAAAGTCTGCCG
ACCACTGTGTCATGAAAATCAGCACCTACAACTCAATACGTTTGTGTCTTTTATCTCACAGCTCAAGATCCGACTCAGTGA
CATGAGTAAGAAACAGGCAGGTTTGGGACAGAAAGCGACGCCTGGACAGAGCAGAGCAGCTCCGTGTTGGCAGGGAGAGGG
TGTAGTGCTGAGGTACAGTGTGGTTGATACACTGAAAATGGCAGCTCTTCTCCAAAGCTGAGAAGTCGTCTTCCCACTCCA
AGCCTGCAGGCAAAACCAAGCAAACGCACAGTTAGTGATAACAAAGTGTCAACAGCCTGCAGCATCGGGATTTAGCAAGAC
TTATGAACACAGTTTGCTCAACCTTGTAGTGCCCCACCCTGATCGTGTTACATTGTTGGAGAGCTTTAGTCTGAAGTAGTT
AGTGAAATAATCAAATGACTTTTCCATGTTGTAGGTTAAGTTTAAGTGTCGACATAATAGCTTATTCAACAATAAGCGATT
AAATTATTATTTTTTGTACTTCTACTTCAAAGGAATACATCAGGAGTACATGTGTTTCCAGCCAATACCACCAAGTATTTC
CTGATGAGCAATCCCTCCGGACATCTTGGTTCAACATCCTGTTTCAGACAGAATTTCATTATAATATTGTAGCTAGATTTA

CTGTGAAATACAGGCACAAACAGAAACTAATGACTTTTTCAAAGTCTTTCATGTATATATATCCAATATAGCGTCTTAAAA
GAGTTTTACTGCACGAGTAGCCAATGTGCGTAAAAGTCAAGGGACTTGTGTAACTCCAGGCACAAGTCAGCCAACTTACAT
TTGTCCTTTGATAGGGGAAATCTGCCAGATCCAAAGTGCCATAAAAATCTACATTAAAATAGTTTCACCTGGATAAATAAT
AATAACTTAACACTCTCTTATGTTCTCTGCTACTTTTTAGTAAAATATATCTGACATCGCACAAAATGCAAATTTCTACAC
AATATGTAGTTAACAGGGTAAATCATGCAAAATCATGAGAGTGGTCATTTAACATAGTACCACTGTCCTCTAATGTGACAT
CTGGCAGGTTGCAGGAATGGCTGCTTGGCGGAGATGTGCAGGGCCCAGAGTGCAGCTCCACTGTGGGCCTCTCCTAAGAAC
AGTAATAGTGGAAATTAAACAGGAAACTAAGAAACAAAGCACAAAACCCACCACGATACAGTTAAACAGCCAACAGGAGAG
CAGTACCTGATCAAACAGGTAGACCATGACATCATCATCAAACGACACACTCTTCCTCCTTTTTACAGCCTTGTCTCTCTC
AGACAAACCTCCTGGAGCAGCGTAGATCTGACAGGATTTCAGAAGACTCTTCAGCTTGCATGTGTTAAAATTGTCTTTGCT
AACCAGTTCATGTTGAATCCCCTCAGAGTTGTTATTGTTGTTGTCTGGCGCGTGGGACCAGTGAGACACGTTATCAGTGAT
ATGCCGACCAGTATCCCCTATAGACGTCCAACAATCCTCAGTGGAGAGTGTGTCACTAAAAAGTTGTTGCGGTATGTCCTC
ATCACAGAAAATGATCAACCTATTTTGTTGATTCTTGGTAACAGCGTCCTGCTGGGTCACAATGTTTAAATCCTTTTTGAA
GAAACTGAACATGTCCTGCATATTTAAAATAAGTCTTGTCCATGGGGCCTCCTTTGTGCACTCAGAGACAGAAGAAGCGAA
AGAAGTGAGCGTTGAATGACGTGTGGGCTCCTCCAGAAGACGACGCTGTCGAGGGGGGATAGAGAGGGATGGCAGCTGTGT
TACCGAGCTTGATGTGCGGTCACAGTTCCTCCATAACTGGGGGATTCCTCTTCCTCTTTGATCAAGGATACAGAAGCAGGT
AATTGGCTGCAGCTCCTCTTCAGCCTGGATCATCTGACCTGAGGATATCAACGCCTGGAGCTCGTCATCCCTGTATGAAAA
TTATTACAGAAAATTATTACACACATGTGCAGCTCATTTTGGCTTCATTGAGTTTTGTTTGGGAATTTAGCAACATTAGTG
TCTCTGATATAGGCCTTTTCTGCGAAAGTAGAGAGCTGTTTTCAATGAAAAAGCTCTAAGAATCCACAATACACTACCTGC
TCAGCACCAAGCAGTACATAGACACAGTTAGCAAATAGCTGGTGAGCATTAGTGGAGCATTTAGCAGCTAAAAAGTGAAAG
ATATTTGCCTCAGTTGTTGGTAGAGACCAAAAACAGAGCTAAAGGAGAGTGAATACTGGACAGCTAGCTCTTGAGAAAGTC
GGGTGCAGAGCCAGAACTCCTGGAGGAATAAACTTCCAAAGTCTCGGCGGATTCTGCTCTGATCTGCAGCTTGTTACTGAG
TTTGGAGATTACTGTTTAACTTTTGGGTTTAAAAAATGCTTTTATCCTCACTCCTGTTTATCAACCTGACTGCAGCAGCGA
TCCGTGGACATGACCCCTATTATCTGGTGTTAATGTTCTGTAATGTTGACTGGCAGCTGGAGCTGGAGGGGAAATGTACTT
TACTTCATGAACACAATGCTACAGAGAGGAGAGGGGAAAAATGAGTCTCTGGTGAGTGCTGAATTCAGTGATTGACCTGAA
TGCAACTGACAGACACACACACCCTCAGTGCAGGTCACTGCTTGCTAGCTCATGGCTAGTGTACAGTAAAGTAACCTGGCT
GTTTGGGGCAAATAAACACTCGAGCTAGCAACGCAAATAAACACAAATGATCTAGAGGTCAAACTTGCCACAAACCTGTGA
GTAAAATCTGATGTGAGTAAGGAACAAATGTTCATGGACAAGGTTATATTTTAGTTGCATTTGCGGACTTCCTTTCTCACG
CTATTCCGGAAGTTATAGTGACAGGCTACCAAAAGAAAAGCACAGTTTTTTAGTAACTTGTGGATTTCTCTGGGTTTAAAC
AACATTGGAAATATTTGGGATAATGTAAGTAAACAACTTACAGCAAAATTCTGACATATTATATCTTAAAGGGACGTGGCA
AAAGGGCACTTGGTTATGGATGGAGCCTGTCACTGCTGAGTTTTGTCACATGGGTTCTGTAACAAGTGTGAAGTTCGGGCA
CTTTCACGAAAGATGATATGCCTCGTCTAAAGATAATCTCCATTTCAGTGTCTGCATTGTCTTCTTTTTTTAATCCACTGG
GTATTTTAAGAGTTGCACAGCGCAGCCTGCCCTTCAGTGTCTGCTAACAACAGGATGTACTAAAACTGATTTGTCAGCCTT
TGTCCAGTGGATGAAACAGCGCACACACAAGCGCACGCATGCACACAAACACATACATTATTTGGCCACTCACTCCTGAGC
CTGAATAAATATCGGGGCCCATCCATCCGCGGAGACAAAGACGCAATAGGAAACTTTGACCGCTCTGCAGTAACCAGGCAA
CCGATGGATGAGTGACTCAGGAAAGGAAGAGAGGAGCTGACTCACAGCAGCTGTGACGTAAAGGATGTGAAAGGACACACA
CACACACACACACACACACCACTGTATGGTCTCTCACACATGTATTCAGGAGGCCATGAAGGGTGTAATCTGCCCTGTTTC
AGGTAAATTAACACCTCACTGTGTAACAGGTGCAGGGGCTCAGGGAAAAGACAGATTATAATAACTTTTCCAAACAGCCAC
CACACAGACAGAGGCATCAATCACAGAGACAAATACCTTTTCTTGACTCCTTTGTCATTGCATCTCCTTTTAGACAGCCAA
TTTAAACCATCCAATGAAATCACCAGTGTAGCTCCATCCCTGAAGTATTTAAACTCTGTAAAAAATAAAGAGAGGAAGGAA
ACACGGTTAGAAACTGATACGCATTGCTTCAAAATGTTGCTGCATCATGATGCTGCAAAATGTGTTGCAACAAGCTTAATC
TCTGTGCCTCCAGCATGCATGACACCGGCATATTTCTCTTCCTACCTTGCATCGTGCGAGCCCAACTCTTTCCCCCGGCTT
TTGTCGCACCACAGAAACCTCATGGAGGGCACCAAAGGAGACGAGCTAAACATATGGGACAGAGAGAGCAGATAGAGATGC
ATAGAAAAAGAGAGGGGAGGTGCGGATGGAGGGGGATCAGGCGGGATAGAGAGGGAATGACCGAGGGGGGGAATGAAATAA
GGGAAGTAGATCAGAGGAGGTAAGGGGGGGATGAAAGAAGAGTGAAGGAAGGTGATGAAGAGGATGAGATGAGAAAGGATG
TGAGGACAGAGAAAGGACAAAGAGCTCATATATACATCGTCTCCCTGGAGGCTGCCGGCCTCTCTGCAGCAGGAGAGTGCA
TGATGGGGATCAATAAGTTATCTGGTCTAATGCAACACAGCCGCCTGAATGATTTACAGCAGAGTGGACGTCACAGAGACT
CTACATTATCTTAATTATTTGACCACACATACATTATTGATATTGACAACTGTTCCAGGTTTATGAACACACAATGAAGTG
AGGCATTCATCTGGTTTGTTGTGCATTCTTTGTCATCAGACAGGCGGTATTTGTCTTTTAAAGGATAAGTTCACCAAAAAA
AGCAAAAGTCATTCATTCAGTTATTATCTACTTATCCTCATGCCGATGCCCATTGTATGAAGTTTTGTGGTCGACAAAACA
GCATTGCAGCATTCTCCAACTGGCTTGTTTTAAAAATAAACAATGAAAAAAAAATCATAAAGGCTCCAATGGCTGAATAGC
CCTGTTGAAACTCCTGCACATGCTCCATAAGTGTAGAAACTGCAAACAAGTGCATTTTTTTCAGATTTTGCATTCTCTAGA
AGGCTTTAAATAACTCTCAACAGACAGGTGAGTTAATATAATTGTTAAAAGCTAATTCTACAAACAGTAATCATACAGCCA
TCATGAATGTCGAGACCCAACTGCCAGGTCTTGTGTCTGTATTTTTATTTCAGCCATGTGCCACTTAAGTTATTACCTGCA
AGTCCAGCTTGAAATAGTCTGTTTCCACCAGACAAATGATATTTAATATGGAGAGCAGTGGTTCCCAAAGTGGAAGGCAGG
GTCCATCAAGAGTCCTTCAGGGATGTTGCCTTGGGTTATTTTAGTTAGATGAATAATAGATTTTAGATTTTATTTAACATT

ATCACAGCAATTCCTCAGAGTTATTTTTAGTATAGTTACACTATATAACTTTTCCCACTCATTATACCCCACCTTTGATAT
GATGACTTTAGTTTGAATACATCTTTACTTTGGGGCTTGAGTGCAGTCTAAATGAAGACCACAGTCTTATCTGATTGTAAT
GCATCATTTTGGTCACATAAATTAACATGTGAAATGGATCATTTTGCTTCATTTAGCAACCTTTTTTTAGATTTTCCATGA
TCAATTTTGCATTTTTTAATCTGATATTTCATCCCTGTCTGATTTTGTTAAGTTAACATTAGCTCCCTCTAGAGGATTAAA
GCAGAACAGCAGCCAGCACTGTGGCAGGACACAGAACTGCAGCAGAGCTCTTAGCTTGTTCGACAATAAAGGCAATGAGAC
TTTAAATTATATTATTTGGCTTAAAGATATAAATTCACAACAGGTAGAATGTAATAATATAATTAATTAAATGAAATGAAA
TACTAGGCCTGCAGTTTAGACATTTAACTACTGTTTTTGGAGCCACTGATGCAGCTATTAGCCTACAGCCTTTACCTTTTA
CACCTACTGATGCTGTATCATTACTGTTATACATACTGCACACTATTACTACTGTTACTGGACATACTCTACATTATTTTT
AAGTATCTTTATTAGGAAGTTTCTGCTGCTATAGTCGTCCCACTGGCAACAAAATCACTGATGCTGGAGTTCTGCTTTAAA
CACCCAGGCAGCATCAGGATGTTGTTCCCAGGAGGCAACAGTGTCACCATTCAGTCCACCTCTGCTTCACAAAAGAGGGGA
AATCAAAGAGAAAAGAGCAGAGAAGGGAAAAAGGGACATAGAGATGGACAGATGGTGTTTCCATTCAGAATAATGAAGGCG
ATTGCATTTCTGTTCTTCTCCTCATTTAGCTTTTTTTGCTCTCTGCACCCGAGGATCCATATCAAATCTATATCAGGTCAG
CCAAGGTTCTCTGACTGCTTACTGTACAGTGTTTCAATATAGTCAACTCTTTTTTCACTGGACTTAGCAGGACACATGAGG
ATGTGATATAGTCGCTGAGCAGAAAACCTTCTCACTGGCACAAAATGTGAGCTTGTTCCGGTCCGTTCTACTTTCTATTTT
CACATCGGAGTTATGATTGATGCCTGCTGCCTCATAATCCGATATCTCAACAACTTCTGCGTCTAAATGTCAGATTAAAGA
GGGATGCGTCTTTGGTTTCAGGACCTCCTGACTGACGCTGAGGAAGCCTTGCAGTCGTATTTTATGACCTGCTGTAGTTTA
TTTAAGAGGATAAAACACTGAATATCATTTCTCAACTGCTGCGACATGTGCTGAGAGCCACCAAGCTGGCTCTTTTGGAGA
TCCAGCCCGCCAGCTGTCCCATAACTCCCTGCAGCACACATGGGAACCTGAACTCGCAGCGGGCATCAACAGGTGAACGAG
AAAAAAGAATGTGTTCGTTGTGCTAGAGGAGGTGAGAACAAGCCCAAGCACTGAGGCCACACTTCAGCTTGAATTCTTTTA
ATGAATTGCCACCTTCCCGTTGTGTTTTACTGCTCTGATTGAGTTGTAACGTGCACAGTGCATGCCAAAAGAACTGTGGAA
CTATCCTTTTACACTGCAGGCGCAGAATCATCATATCCAGACGGTGACTGACACCAAACAATCTATGAGAGGAGGGTAATT
GGAGAGCATTGGGCTTATTCTGAGCCCTCAGGATAGACCGTGCTCGAGGCCTGCCTGAGGAGGAACATATGGAGCAGGTCA
ATGTCCGACGCCAGTGGAACGGTGCTGCAAAGCTCTGTGGGTAATATGGGAGAATGGAACATCCAGTCAGGACGCTGGTGG
TGTTGTTTGCAAGCCTTTATTTGGACACAGCAGAGTCCCACGGCTGCAGAGGAGAGGGGCTGAGAGGGGGTGAAATGTGAC
ACTAAGATAATGACATACAGAGTGGTAAATATGTCACAGTTGTGTACATGTTTGTCGAAGGTGTTTGGTTTCAACAGCAAT
AAAACGTAAAGCAAGCCGTCACCGAACCTCCCTGCAGCCAGGGTGCAGCTCTGCATCCGCTTGTGAATTGAATGACTAATT
TGTTGCAGATCAGCACCAGCCTCTGCTGCGCAGCGGTGAGCCGTGCCACAAGCTGCCTTTGTAGGGAGAAAAGACTTTTCT
CTTAGGCGGTAATTACATAGTCTTAAAGGTTTGCTGACAGAGTGTCTTTAATATGATGACATAAATGTGTAATCCATCTCT
GGTCGCTGGCTGAGTATTTATGAGCCTCTGTTGACAGCAGTACACCTGCTCCTCCCCAGTCCGGACCTGGGAACAGTGCCC
GCCTGAACTAGCACCACCACTGCCAAGGGCACTGCCATATTGGGAGAAGTGTGTGTGTGTGTGGGTGTATGTGTGTGTGTG
TGTGTGTGTGTGTGTGTGTGTGTGTGTGTGTGTGTGTGTGTGTGTGTGTGTGTGTGTGTGTGTACATTGTTTTCACATAGT
GTGTCTGACATGGTTGAACCAGATTGTGCTAAAAGACTAAAATCTGAGTGTTTTTTGAAAAAAACACACTGACCAGAAGTT
CTGCTGGGTGCAAAATGTGTTCAGTTAGGTTGGTCTAAGCCTTCACTACATTGAAATCATCAATAAACAAGAGGGACATGT
ACACGCACGATGATCAAAATGTGAAACCAAAGTATCACCCCAAACTTTAAAGCCCCTATTTGGATGGATTCATTCATTTAC
TCTGTAAAGACACTGTGATCTTATGTGGTAAGTAAAACACATTAAAAATTAAATTATTAACACAATGTGAAGTTCTGACGT
AATGTCAAAGACGTATATGTGGTGTTGCAGAGGTGATGATTGAAGTAAGCATGCTAACCAGCTAGCCCCTTTGTACATTAA
AGGGATAGTTCGGATTTTTTGACATGAAGTTGTATGACATCCTCACCATCAGTGTCGTGCATCAGCAGTGACTTTTCCCCC
ACTGCATCCTGTGAGCCGAGGTCTGTCCCGCTGTTGTTGCTGAAGAAAGTAGTTACGGCTAGTTTGCAGGGTCACGAAGTT
AAAGCGTTTTGCTTCAAAAAACAATATCCGTTCAAAAGAGTAAAACATTTGCATCACAAAATCGTTTACGACAAAAAAGTC
AGACCTCACGATCACCTGGCACTACAGCAACAACAGCGGGACAGACCTCGGCTCACAGGACGCAGTGGGGGAAAAGTCACT
GCTGATGCACGACACTGATGGTGAGGATGTCATACAACTTCATGTCAAAAAATCCGAACTATCCCTTTAACATGTGATAGT
ATGATAGTAGACATCACCAGCACTCCCCCAATGATCTGAGCTCCCAGTCCAGGCAGAGTCAGCTAATACGACCAAAGTTAA
CTGAGCTTACTAGCTAACAGCAGCTACAGTTAGCAGCAGTTAGCGGCTACTCTGATGATATATAGAGTATGAATAGTACAA
TATTTGCACTGTGTAATTAAGTTTTCAGTTGTGTTTAACAAAACAAGATTTTCAGAGAGAAGGGTCGCTGCACCTAAATAC
TGATGGATTTATAGGACACATTTGCATTTTTTGCATAGTTTGCATGTACAGATTCTGATAAATGAAGACCAGAGAAGCAAA
TGAATCCACAAAACATGACAACTAAGTTGCTATTATGGTTAGTTAAAACCTTTTCTGTCGTTGCAGGCTTCTACCACTGGA
CGGCAGTAGAGTGCAGCAGTTATCGTCAGTCGGGGGAGTGATGTGGTTGAGGATTTCACACACAAGCACACAAGCTGTGAA
CCTTGTTAGGGAACACAAAGACAGCAGTGGTGGTGTTAAAAGGAGTCCTTTAATCAAAAAAAAAGCATTTCCTTTTTTCTT
TGCATTTTCTCCTGTCGTTTCCCGGTCACACATGCACTAGTTGTGAGCGAGCAGCGAGCAGCAGAAAAACAAGAACAGCTT
TGAACATTAAAGCATTATATCAGTCTGGTGCTAAAGCCGGTGGTGAAAGGCCTGCGCCTGTCTGTCACTTTATGTGGGCCC
AATTCATTTTCTCCACTTGTGCCATTTAAAAGGTTGAGCAATGACTGCACCTGTGTACGTACATAAACACTTTCCTCATCC
TGTCGTGTAGTCAAACCACAGCTGAGAGAGCAGCTAATCAGGGCGAAATGATATTTCAGCAATAATGTGAATTTCACATAG
TTGTCACGCACACACACACACACACACACACACACACACACAAGTGGGGAGGAATTTAACTTGAAGATGGTGGGAAACGTGTT
GAAAAGCGAGGCCATGACCTGTGCGAACACACTCAGGCAGCTGAGACAGCTGTGGGACTCCTCAACGGCTTTAAAGTGTTT
ATGGAGGAGCTGAGTTCAAGCTCTCGTTGACAGAGCTCTTATTTACTCACACTCACACACACACACACACACACACATAAC

CCTGATCAGATAAGAGCAGATCTGTTTCAATGGCTAATCTTAAATATGATGAGGTTCACAGGGGCCCGTGGAATATACATA
AATATCTTTATTTGGGAATCAGGCGCTGCCCAGGGGTATATCACACTACTTCACACTATAGGAACAGGAAACAGGATACTA
GCTTTCATGTCAGACTCCTACTTTGATATGGAGACTTGTTTTGGCTCAATCAATGTCAAATGGACACTGTGACAAGGAGAA
AAGGCCGTTAGAGAGAATATGTTTCAGGGAAGTTCCCAGCTTTTACTGTGTGTCCCGTACATCCATTCTGTACACATATAA
ATCCACACCAAGCCCCTCTCCCTGACACCACTATCTTTGACACCCTACTCTTCTCAGAGACGCCTGAGGGAGTCCCTGAGT
CCCCCCCTCCTCCTCCTCCTCCTCCTCCAAAACCCATCCACCCAGTCGGAGCCCCACGCACAGATCCTATCACCAGCATCT
TAAGCTGACTGGTATTCATCGGGCCGTGATCCAGTTGCACAGCAGCAACCACATTTAAGTCCAAAATGTAATGAGTCAGGA
TTGCCGCTTGGTTACGGCTCAAACAGATAAGAAATAGAGTGGCCGGGGCTCAGAAGATCGAAATGGTTGCACTTAATAACA
GGCATCTACCGGGCAGAGGAGGATCCACTTCCAGACAACACCAGATTATTGCTGCCTTCCAGCAAGTCTGCAACATACAAG
CACTTCTGCGATTGAGAGGAGTTTTTAACTTTAAAGGAAAAGTTTAACGTTTAGGGATTATTTCTTTTCGTAAGGTTATTA
CCACTGTCTTGTCTTTCCATTAACTATGAAGCTCCTATCCAGCTAATGTAGCTCAGCGTGAACAATAGAAACAGGGTTCAG
ACTGGTTGGGACCAGTAACTTCCTGGATTCTCCCCAGGTTACCCATTTTTTTTTTTTTTTATTAATTATATTATTATATTA
TATGATAACACATATAAGATACAATACAATGTGTGTAAATTGTAAATTAGTGAGCTTTAGAGATGTTGGTAGGCAGATTTA
TTTATGTTTTGACAGAACCTGTCTCACCGTTTACTCCAGTTCAGTTTTTGTGCGCAGCTAAGTTAGCGGTTTGCTAGTTTA
CTTTCATATTTAGCTAGCACAGACAGAGATTTGTTTTTAATCGTCTCGCCTCGCTTATGTCCCAGCAAGAAAACAAATGTC
AAATTAATTCATTAAATGTCAAATTTTCTCAGATAGCCTCCTCTTTGAGCAGTTAAAATGAGGACAGCTAACAGTTTAGCA
CAAACACTGGAAACAAGGGGAAACAGCTAGTAGAGGGTTTTGAAGGCTACAGGACTGTTTCTTGGCCGGGACCATTGACAT
CCCTGGAGTCTCTTTAGGTTACCCTTTAGTTTTTCTATGATTTAAGCATACAAGTTAAAACAATATCTGTGCAAATAAGTG
AGCTTTAAAGATGTTGGTATATACATAGTTTGTTAGCCTACCTTTTGACAGAGCTTGGGTAGCTGTTTACTTCTGTTTTCA
GTCTTTGTTCTAGTTAGCTGTTTGCTAGCTTACTTTCATATTTAGCTCGTGTAGGCTTCCCTTTGACAGGGAAAAAAGGAC
TAGCTAACAGTTTAGCATGGACACTGGAAACAAGGGGAAACAGCAGTGGGGGGGTTATGTAAGGGACTATTTCTTGGCCGG
GAGCAGTAACGTCCTGGAGTCTGCCCAGGTTAGGCCTTCACTTTGGTTTTTCTATGGATTAGACACACAAGATTTTGTAAT
GAAATGTTGAATATTTTAACGTTTTTTTCTTCTCAGTGGAAAAATAGATCCAGCTAACAGTTTGCTATTTTTGTTCAGGAG
GCTAATGAAACTCAGACTGGACTGGACCTCATCAACACTGAGAATGTGAACACTTTTTAGATCATCATTGTTTCCTGTGTT
GAACATATATTAAAATGTTTAATCCATTTTTCATTTAATTTGTGGGCCAACACTAGGTCGAGTAGAAATATGAAGGTATAA
GGTAAAAATGGCTAGCACTAGCGTAACTCCAGGCAAAGTCTAAAGAGACAGAGACAAAGTGAGCGTAAGAAGGCAGAAAAG
TTTTCTTCAAGCTGTTAAAGGTTGAAAGAGCAACCCAGCAAATATACTGTAGTTTCTCTTGGGGACTTTATTGGCCAATTG
ATTCAATGTGATCACAGTGCAAAAGTAGTTCATATGAGGAGGAATATTTCAGGGAACTGAAAGGCAACAGCATGACCATAC
ACCCTGTAGATACATGCAAAACCACAGGCACTGACATACAGTAGACATACACAAACTGACACACATCCAGTCTTGAGTGAT
TCGTGTCAAAACAGAGGACACACACATGCACAGACACAGTTAATGCCACAGACGACCACACAGATTGAAAGATTGGTGCCA
AAACAGTGGAGTGAGACAAAACCATTAGCTAGAGTGGAGGGTGGTGGTGGTAGGGGTGGAGGGTGGAGCTTGAGGGGGTAT
CTTGGCTGAATCTGAGAGCATCACTGGCATGCTCACACCACAGCTGGTCTGATGGAAATAGAAGAGGCAGTAACTGAGCCC
AGGAGAGAGGAAGAGTGACCACTTTCCTCTTCCTCGCTGTCTCTCTGTCTGAGAGGCCGAACGTTTCAGACATTACAGAGG
GACTTACAGATTTAGTTTAGCTGGACTAAAGTCGATCGGATGACATCATCATCCATCTGAATTAAAATGGCTTTCAATTGA
AATTGTTGCAGGCGTTACACACAGAGTATAAAATGACTTGTGTTGCTCTAAACACATTTTACATCTATTCTCATGAAAGGT
TCATAGCGTTTGATTTATTTATGTTAAAATCTTTTCTGTGCTGCAAAGATGTTCTTCCAAACATTTATTTTAGCATGTGTA
AGTTCAGACAAACAAGACTAGTCGTCTATATTAATAAGTAGCAGTTAATGTAAATTACCATACAGGAAACCAACGTTTCCA
GCTGAATTCATTAAAACAAAGTTTCTTAATAAATGTGCCAAACGATCCCGGATTGCAAAATTCTACCAGACACCCGCATCT
GGCCCACAAACTGTGTGGAATGATGGCACTTAATAAACCTGAAGTGGCTCAAGTTTGGGCCGGATCCAGCCCAACAGAATA
TCTCTATTTCACACACTACTGATGTAGGCAAAACTGTCATTAAACACTGCTCTGCTCACTGGGTGCTATTTGGGGCTTAGT
GTCTTGCCCAGGGTCACTTAAACATGTTGAGAACCTGGGATCAGCAAAAGCAAACCTATAATGAACTGACGACACATTCAA
CTACCTGACCTGAGCCACGCTCACTAAATCTTTTGAACATTTGGGCAATAATGATGCAACATATTCTGCAAGAATAACTTA
AAGTGTGATGGAGAATAGAAAGTGAGAAAATAAACACCAGAGGCAGTTTTCTACAGTGAATATCTCACGCTGCTCTATGAA
AACTTTGCTGTTGATCCATTCCACCTAAATGATAATTCAGCAATCTGTATGGTTGTTTATTACTTCTAATAACAAATTATG
AATGACTGATGCTAGGGGAGAAAAAGCACCCTGTAAGCCAAATCTCACATTAAAAATCCCCACAAAAGCCAGCTTGTGTGA
CTGTGTCTGTGATACATCCTGTCAAAGTGTCCTTGAACAACACGCTGAGTGTTGATGAGCTCACTCAATCACAAGTTCCTC
TAGATAAGCTTTGTATCTATATAACATAGATACCCAGACAGTGCAAATTAAGAGTATATAAGAGTTTCTGGTTCTCAAATT
TGTGTAAAATGCAACCTACCATCTGTGTTTGTGTAATACTTACCTCTGTTGGTTTTCTCGCCCTGTGCTGATTGTCGCGTT
GCTCAGTACTTTGCTCAGTCAGAGCAGTTTGTTTTTCAGGCATGTGAAAGTACAAAACTCCAGGTCCAAATCCCTTTATGA
TTTTTATAAATACACAAAATTGATTCCCTGTCTGCTTTGCTCACATTCTCAGGTGCAAGAGACAAAAAAAAGTCAGTTTTC
TGTCTCAGTAAAAGATTTGTACTCTGATCCCTCCCGTCCTCAGACTCCCTCACAGCTGCCAGGAAGCAACCAACGCGACAA
ACTCCCCCAAAATCACTACGTGCACAGGACCGTCACCCCATCCCCATCCATCTTTCTCTGTGTCTGTCCCTTCCTCTCTGT
CCCAGCCCCCTCTTCCTCCTCTCATCCACCGCTCCATCACGCAGGAAGCCTGGTCTTCCTGAAAACAAGCATTGGAGCGTG
GAAAGGGACAAGGCTGAGGGGGGCAGGTAGGTGAGGGGGGGGCAGAGGATGTTTTTGTCATTCCTCTGCCCCTCTGGTCGC
TGCAGGCCTCACTGTAGTCCATCCTGAGGAAACCACCCTCACGTCACTCTGGCCAGGCCTTGGGCCCACACGTGCTGCCTG

TTAGGGCCAGTGTGACGCAGTGGCTTCAGCGCTCCAGCAGCCCCTCGCACATGGGAGCCATAAAGACATATGTGGACTGAA
ACTGCGGAAAACACTGCTTTTCAAAACCATAATGGCATCAGCAAGCAGGAAGAGCTGATACAAAGTCACAGAGTGCATTTC
AGATAGACAAAAGAGGTCAAATGTGGCGTAGAGAGATATTGTGAGGAAGGTTTGTGCTTTGCCAGAACAAAAGTCTGTAAT
TTGGCCAGCAAACCACAATCTACTTCCTGTTTGTCGTAGTTGCAAACAGCCAAGTATGAGAATTCCTCAGCAGGTGGTTTT
CCAGAGATTCCCATCGACTGATTTATCGCAACAGAAAAAAATGTTAGAGGCTGTCTTTTGTTCGCAGTTACCATGACAAAC
ACTGTGATCATAAAAATAAATAACTGTGAGGAAAGCTTCAAATCTATGGAGGCATGGCTCGTGTCTATGTAGTCACCATCA
GGTAATTTGCCACAAGGGGCTTTACAATCTGTAGGCTGTAACATAGGACATCCTATCCATCCATAACTCCCCAAAGGGTTA
AAAAATGGAAGAAACTTCAGGAAGAGTAACAGAGGAGGGGTCTCTCTTCAGGACAGACAGACATGCAGTAGATGTTGTGTA
TAATATACACATGACTTCAACCCTAAATGCAGAAAATTGCATGTATTTAAAGCCACAACTGCATGTGTGGAATTTTTAAAT
GTTTGGCGGTTTAAGTGGTTGTATAAAAAAACAGTTCTACACGTTACAACTTTAATTCATTGAATACATTTCCTATTTTGA
CACCATTCACCAGTAATTCAAAGGTTCGGCTCTGAAATATCCACATTTTTAACATCATATCCTGATGAACGCATATTAGAA
AAGTGGTTGCAGCAGCTCTTCATAAGTTTAAAGTTCCCTATGTTAACCTGGTAATAACATCGGCTAAATGTTTACTTACAC
TTGCTGAGATCATTGAATAATGATGTTAACTGGGAAGTGTTCAAATTATAGTTCAAAAATTACACACCTCAAGTTACATAA
CATTATGGCAGTAACATTAGATGAAGGACCAAGGCTAATAACATAAACTAGGTTAGCATAATCCAATATATCACATCCAGT
CTAAACTAAATGAACGCTGCTGACTTTCAAATACATGCAATTCTTTTTATATGCAAATACAAAAATAAAATAAAACAAAGT
AGCATTTTTCTTCAGCTTTAATGGTGAAGCCAGATTTATTAAGATACTAATTTGAAAATAGCTACTACAGTAGTTAATAGT
TAAGAACGTATGAAGGCCTTTACACACAGACTTAGTGATGAATTTACAAATAACTGGTCCATCTAGCAAGTTAAGCATCTT
TTTTAAATAAAGAGAGCATATGTTAGAAGTAGCATGTGAAAACAAGGTGACTGTGAGTGGAGACAGCTCTATGGAGCGCAG
CCTGAAAGTGACAGAGGCCATAATAACAGGATGTTTACAGCCTGCGGAAACACTGCCAGGGACGACCACGAGCAGGCAGCA
ACTGTGGCTGGAAACACGAGCGAAGAGGAAATGCAGGCCAGCCAGGTTCCCTCCAGATCCACCAAATATAACCTCAGTGAC
TTGCTGTTTTGTTTACAGACAGACGCTTCCCTACCGCGGTCTGTTTAGGTAGATTTAATTTCCAATATGACCTCTGACCCC
TGGAGAAACCACTCTCCAAGACACAGTCGTTCAATAACATGATTCTCTGTCTAAAGTCTCACTTTAACACACACACACACA
CACACACACAGACACACACACAGACAGATACACACACACACACAGTTTGCTGAAGTGGTCAGCTGCAGGTGACAGCAGACA
GCAGGACACTGGTGGTTTCTGACACCCGGGGGGTTTAGATCTATTCACTGTCACCAGTAGTTTCTTTCTGCCCATCAGTGG
TCACATATTGCACCAGGCTGCAACATCCACCACAGCAGAGTCTCCACCACCATGGCTATTACCGACTTCCTCGCTGCCTCA
GACATCACTTCAGCCATTAATGCTTGTAAAGGTCAGAAGATGATTGTGTGAACGACAGATTTATACTTGTTCTAGTCGCAG
TTTGTTTTGATGCATTTCGCTGTAGTTGTGTGAGTCCTCAGATTATAGGACATCATCATTTAACTGACTGGATCTAATCCA
GTTTTATCTGTGATTTTGCTTTCAGCTAAAGATTCCTTCTGCCCAAAGATGTTTTTCAAAACAGTGGGTTTATCGAAGAAA
ACTCCGACAGAGATCGAGGGGGTCTTTAAGATTTTGGACCAGGACAAGAGCGGCTTCATAGAACAGGACGAGTTACAGTTG
AGTTTCAAACACAGTTTATATCCTTCTGCATAATTAATGAAACAAGATGGATTCCACAAGGCTCTTGAGAGTTTTCTGCAG
TATTATGTTTCCATACGAGATGCAGCACATTTAACATGTTTATCTCATTATGTTTTCTCAATGTGGTGCTTCATCCCTGCT
CTTCATCAGACTGTTCCTGCAGAACTTCTCCAAAGGAGCGAGGACCCTGACGGCAGCTGAGACCAGAGCTTTCCTGCTGGA
GGGAGACTCAGATGGAGACGGAAAGATCGGATGGGAAGGTAGGCAGATCTGAGGGGCACTCGGCCTGTAGGTGGTCTGCTT
TTTTCTAGTATATCAGGCAGGAATGTTTGGTTCATCGTGAAGAGGAGCAAGGTATTTCTCACAGACAGAGGTGATCAAGAT
CAGGGTCTGTTTTTATTAAACTCTTCTAAAATACAAAGGTACAAGTGAAAAAGCTTTATCAGACTATTTTAGTATTACCTA
GTATGGGAGTAAACCCTATAAATGATCACATGATTATGCTAAGGGCAGGAATATTGAAGGAATATATGAAGACACACTGAA
AGTAAATTCAAATAAAATAAGTGGCAGATTTATTTGCAATTTCTAAGTATTTTTAAGCCATAAAATGTTTGATGCATATTT
TTTTAATATAAGTTTGTCAGTAGGAATAAAAGTACATCATTTTTAATTATTTTACCGTAGTATAAAATGTACCTTTTATAA
GAAAGGACGAAACTCAACCAGCAAAACAGCAAAAACTGGGATAAATCCTCAAGTAAAAAAAAAAATAGCATCCTCCTTAAA
TGTAAATTTCCAGTTAATGGTGAATCACTGGTCTTAAAGTCAGCACTCTTCATCCCTGTGTTGTGTTTCACTGTGTTGCTA
GGCAGCTTAAGTATCACCATACATAAGTGTCACTGCCAGAGCCTCACACTTAATGTTCAGCCATGTTTGTAGTTTGTAGCC
TAGAAATAAGATGATGAATTTAAAAAGAGTAGAATCAAAAATGTCTGAAATTGTATCTGTCTTCCTTTTCGTTTTTCAGAG
TTCTCTGCACTGGTCAAGTCTTCATAAATCATCATCTGATCACATGTGTTTAGAGTAATAGTGTATGTTGTAGATTTAATG
TAATAAAGAAATTAAAGTAGACCACAAAATCAATGTTGCCTCTGTGTTATGTGGCTTTCCTTGCCAGAAAAGTGAATAAAT
AAACATACTTTTCTCCATGAGATGTCAGTGTTTTTTTTCTCCTGTGATAGTACCTAGTGATAATCTTATGAGCAGAATTCT
CTCATGTGCCTCATGTTGTGATGTTATATTGAGCACTGGGAGAGGTCCAGCCTGTGCAGGAGGCTTCGGTTTCTCTAATTA
ACGGCCTGTGCAACTTTCCGCTTTGCAAATGAAGTGTTTGACATTCTGGCAAACTCAATTGGACACCTTAGATGGAGTAAT
TTGGTGTAAATTATATGTTTACATTGTGACTGAATGTGTATAAATTTGAACTCATTTTCTTTGAAAAGTTAACAGATTACC
CTAATATTATATATTATATATCATAAAACCCAGTATGTTTCGTTTTGGCTCATGCTGGCTTAAACAAGCTCATTGAGGCCA
TAAAACAGTCTGGACTGTATTTGCTTTGAGTAATTTGACTTCTCTCAACCTCTCATCTTATAGGAAAAATGTATGACATTC
AATCTGCAGTTTGCACAGAACTAATGGGCTCAGGCTGAAGAGACAGTATGTCCCTCTTGTGTTTCGCTCAAAGACTGCATC
ACTTTCTGACTGATGATTTCTGTGGACAACATTTACAATCCATACAAGAGACAAGGATTGTATGGGTTCATTCAGGATGGG
GAGGATACGGGAAGAGTGTTGTTGCCACTCATTTTTAATTACAGCAAATAGCTCCATTTAGACAACTATGTTTAAGTCAAA
TGCAGAGGTGGAGAAAATAATAATAAAGTAGCAGAAGGTAAAGTAGAAGTACCTCAAAATTGTACTTGAGTACAGTACTTG
AGTAACTATTACTTTCCACCGCTGCAGGATCACAGTTATCTCCAGAAAATAGATCACACCTTCTGAAGAACATAACTTGTG

TTAAACTAATGTGGATGGAAACTGTGTTAATTGTTTTCTGAATGTTGGACAAAATACTTTATCTGTGTGGTGTAACTAAAA
AGTACTAGTCCTGCGTTTGAGTTTTTATTTAAGTTCAAGTCAAGTGTTAGAAACAAAATGTACTACTACTAATCTTTATAC
AGAGCAGTTTTTTATCAGTTTCAATTTAGTATCATATTGGATCATTACAATTCATTATGTAAGCTTCTTATCTACAGGATT
CACTGGATCAATACCAGACACAGTGCAGCTTTCTGGCAGCCTGGGCTCAACCACTCCAGCCCAGAAGTATTTATTGTAAAT
TGCAGATGGGCTTGGGGGGCACAGGCAGGACACAGTTGGATCAGAGGTCGGTCTGGCAGACAGAAAAGTAGTTCTGGCAGA
GCTTCAGCAGAAGCTGGCCTGAGGTGTACTTGCACTGACCCAGCAGAGGAACTCTTGGATGGGATCGAATTTCTGTGCCAG
TACATTTACAGGACTTCTGTCAATGAAGCACATTTATTTGCAACTTTCTCTCAGATGGCAAGCCACAGGGTCAGAGAATGT
AGGTCACTTCTTTTTCTGCTGCAGCACGAGGGAACAAACTCAGGTGGAGCATTAGAAAAACCTACAGAAGAGCAAAATAAA
TATCTGCTGCAGCTTTACACGGCAAAAACTCAAAATCTTAGTAAATCAAAGTATGTTTGTCTATTTATTAGTCAAAAATAT
CTCATTACACTTAATATAAGACATAATCACCTAGAAAGTAGCATTTCAGTGAGATATGGGACTTGTTTTTAGATCATTTTA
AATTGTTTTGCTTGATATTGAATCAGAATTTTTCCAGAGTAAGCAAATTAATACGACAGTCAAATGTAAGGTTCATATTTG
GTTGCTAATATCTTAAAATGTCAACGCAATTTCTGTGTTTACAAGTATTTATGTGCTCACTAAAGTATGGGAAATTAAGGT
GCAGCTTTCTGAATATTCCACAAATCTTTTACAAATGTATATTTCAAACATGAAGTGTGAAATATGATGGGGGATCGTCCT
TCGCCGCCTCCAATTGCAGACGTTCATAGCCTTGCTTGATGGGTTTCATCCCCGCTGTGAGATATTTGTAATTTAAACCTG
ATATTGAAGAAGGGCAGGAATAGTGACAGTATCTATGGCCGAGTTGTCTGAGGTGAGAGACCTCATTTGACCAGAGCAAAT
GTTAAAGGCCAAAGATTACAGAGCTTTTTTGGGAGGATTTGAAGTGAAGATGAAGCAGAGTAAAACAAATAAAGGACAAAT
ATTATAAGTGCTGCTGAGACATCACACGTGAAACCAGTGAAGTGAAGGAGGGGACTAATTAGGCAGCATATTTGGGATTTT
ACAGTCAGTTTATGCAGAACTGAAGCTGCATTTTATTAGATGTTTTTAATATGATTTGTATTTAATTCAATATTATCTCTA
CATTATATTAATCATAGGAACATCCCAACACTGCCAACTGATGTATGATAGGCACATAACCGCCTGCAGACGTCATGGGAG
CAAATATGGGCATGGAAAGACCCCTGGACTAGTCCCCCATTTAATGTCATTGTTTGGTGCAGAGGGAGAGCTGGGCTTCAT
TACACAACTGAAGTGTTAAGTTGACGAGGATAATGGCGACAGTGACGATGAGGAGGAGGACACCGGTGATATCAGTTTTTG
ATGCGTCCCAAAATAGCATCACAAATAGTTTCATCACCATAAGGAGAAATGGTCTGAATGGGTGTAAGAGGACCAAAAATA
AAACCTGGCAAACGGTTCTCGGTATGCGTGTGAGTGGATGATGACGAGGTGCTTTTAAATGAAAGGAGGTCTGGCTCTCCA
GTTAAACCCCCCTCCTCTTTGGCACTATAGGTCATAGTGGGATTTTTTAAACATGTCATCTTGTTTCCATAAAGTTTTTTC
ATGTTTCCTTTTTACTCTTAACACCTTCTGACAAAGACTGACTTTATATTCATACAAATGTATGACCTTTTGCAGGAAGAG
ACCTCCTAAATGATGCAGAAACATCAATAACACCAGAGGCCACGAACACTATTTGTTAGTTAAAGGGGCACTATGTTGTTT
TGGAGACGAAACTCAAACTCAGAATTTTAATATTTACAATATAAATGAGGTAATAATACAAACTCAGACATATTTATTTTT
TTCCATAACTGAATAAACAAGCTGTTCTCAGAGGAAAATAAGGTTGAAGCTAGAAAGGTGGCAGGACCCGCCACATATAAA
CAAAGTAAAACTGTATGAAACGGTGTTGTCCTTTAAGGTCAGTTTGTTTCTTCAGTTTATTCAGTCATGACAACAAAGAGA
GTTTGTTTATTTAGTTTGTTAGACATAAAAAAAATATCTTTCAAGATCTTTATCTTCGGATTTAAATTTCTTCCCCAAAAC
TACATAGTGTGCCTTTAAAATAATGAAATAGAATCACTACATTATTTCGATTTTCAGCATGAGCAACAATATGCATAATCT
CAAATATTTTATTGATTTTATGTTTTTAATTCTGTTAAACACCAGAAGAGTGCAATATTAGTCATCGGATGAATGTCTGTT
ACAGCCTGCATCAACATTTTACATGCCTATTTTTCACCTTGGTGAAATCGTTGCCATCGCTGGCGATAATCAATCACAACA
ACGAGACTTTTCTGACACTGGAATGCCAGGAAAGTCATTGCAGACATTTTACACACGCCGATCACGCACAGGCTCGTTTTC
ACCAAAACGGTTCTGTTCAGAAGGCACGTGCAGGGTAATTTATCACCCTAGGTGATTAAGATGTAAAAGTACAGGGTGTCT
ACGTAATCAGAAACCATAACATTGTATTTCATGTGTCATTTTTTTATTAAGGAAGTCTCACAGCACTTTAGAAGACAGGCT
AATCTGGATTCAGATGATCATTTAAAGTGACATTTTATAGGACAGAATACACAGAATATATTTTACAAACATCATAATAAA
GAGGACATTTAAAGAGCTACGTCTTGGCATTAAAAACATTTACATTTCTCATGAGAAAGGTCTGGGATACAAAGTTTAACA
TACTGATATTATGGCACAGTCTTTGAAATTAGGTTCATAACAACAAGGCAAAACTAGTCACAACTAAACAAATCTGTCTGA
TGAAAGAATAAGTGGCTGTAATAACTTAGACTATAGAAACCTGCTTATCTGTCTGACAATCTGCAACACACAAAGTAAAAG
TTTACCTCATTCTTCTCTCCGTGGTGCTCGGCACAGTCAAACAGATCAAAACTACATGGCCACAGGGTACGTTTCACCCAA
ATCAGCAGCGTATATTATTAGCCCTATGCAATATCAAGCACTTTTGCATAGACCTACACTTAGCCTACCATTTAAGCATCT
TCATTTATGTGCATATAAATAGAACAGCTCTGTCTAGTGGACACATTTTTCACCCGACTGCTTTGACTGCTTTGCACACAG
TGTCTATTACATAGCCTATGCATCATATTTCGGGGCAGTGAAGGTATATAAGCTTGCAAGAGTGGCTGAAGGCTTTACTGC
AGTGAGCAGAATCATCCTGAGCGAATCCACTCTAAGCCTTTAGAGAAAAAACCCCAAGGTGAGTTTCACTGAGGAGCTGCA
GACCTTTAGGCACTGAGCGGTTCAGGGCTTCGACTTCATGCAACGGGATACAAATCAAGGAAAGGGCCGCACAGGGCCATG
CAGATTTCTGAACATCAGAAGCTCAGAGTTTGGCTTTGGACTAACTAAGGAACCAAATGGGGGAAAAAAAATCTATTTGCC
AACCAAATACAGCTGCAAAGGATATTTGATCTTTTGCCTTGGAGGGCTTGGTTTTGGAAAAATTGGACTACCTTTTCTGAT
CATTTGGGGCTACCAGAGAGGAATACTCACAGAGAGGAACTCTAAAATATATTGCATGGCATAATGAAAGTGACACGTAGC
TCTTTAACTTTAAATGCTGCATTGTCAAGTGAGAAAACTTAACATGCTTAGATTCGAACTGCTTTATCCTGTCTGATGTGC
TACAGCTGAACTCATGAACTGATGACTTCCTCTTCTCTCCTCCAGCAGGTAAAAATGGCATTCGGTGGTATTCTGAAGGAT
GCTGACATCACTGCAGCCCTTGCGGCTTGCCAAGGTAAACCCCACCACGAGAATGACACACCATGCAGTATTACGACTACG
TATTAAGAGCCTACATGGTGGCTGCACATGCACACAATCATGCACTAGAAAAGGATCTGGACTCTAATTTTACTCATTTGT
AGTTGGATATCTTGCTGGAGAATTCAGAGTCACATCTCAAATTGTAGTTAAAAGATGCTCCATGCATCTTTTCTGCATTTA
ATACTAAACACATCTTGGAGGTGGATCAATAGTGCAGCAAGCAAGCAAGCAGAATGTGACTTTAATGTAATTTCTTTATGT

CTTAGCACAGACATTTACTCAAGTAAAGGTAATAACACAACTGTGTAGAAATATTCTGTTACAAGTAGAAGTCCTGCATTC
AAATGTTTACATAAGCAGAAGTACAGAAGTATCAGCATCAAAATAGACTAAAAGTACCAAAAGTAATTTTTAGAATCACAT
TTTATAATTGAAATCATTGATGCATCAAAGGTGCAATATGTACGAATTGGCCACCTGTGAAATTCATACTCTAAACAAGTA
GGGGGCACATATCACCACAGAAACCACCAACTGCTGCCAATTTTAGCTGCCAGTTAGATCAGTTAGCTGTGCAGCTAGCTC
TTCTATTGGCTAAATCTGCCCGGACTGGGAGCTCAGAGCACTGAGGGAGTGTTAGTGTTGTTAACTATTGGAACAAAGCAG
CTAGGAGCTAGTCACATAGCATGTTAATAATAATATTATTATTAGAAGCATTAGATAATATTAGTTTTTTTTTTTCATTTC
GCACATGTTGCACATTTAATGTGTAAAGGTGGTGGAGTAGAAGTATCATGTTTCAGAAAAAAGTGTTTCTTCCTAATGGCC
AGCAGGGGGCGACTCCACTGGTTTCAAAAAGAAGTCAGATTATATGTAAGCTTATAAGAAAAGGACCCTACTTCTCACTTG
ATTTATTCCCTTAGTAAAACGTTTTCTCATCAGTTTATGGAGTCAATCGCTACTTTCAAGTCTTCTTAAATACAACATGAT
GTTCCATCAGCAAATTATGTTGCCATTTAGAGCAAAATAGACGATAAAGCAGGGTATGATTTAGGGTGTGTCTTTATGTTC
TCAGTCAGATCAAATCAGTGTTGTCCCTAGCTCCCCCCTTTAATCCAAGTAGGGTCACTTCTGCCTTAAAAACAAAACAAA
AATGACAACAGCTGTAACACCAAACTCAAGGCTTCAAACCGGTAGTCTACAAGGTAATGGGTGACATGGTTTGTTTGCACT
TGGTCTGATATATTCCTTTCTGTGTCTTCTCACAGCTGCCGACTCTTTCAAGCACAAGGACTTCTTTGCTAAGGTCGGCCT
GGCTGCCAAGTCTGCAGATGAAATCAAGAAGGCCTTCGCTGTCATTGACCAGGACAAGAGTGGCTTCATTGAGGAGGATGA
GCTGAAGTGAGTGCCACCTTGAGATTTCACCCCAAAGCATCTGAGAAGCTACCTGGTTACTCTATGGCAGCTCTCTCCTCT
GGTGTTCGGTGACACTGAGCCGAGGAGAAAACAGAGCAGACATAGCCTTCTTGAAATTTGGAGCTAAATTGAGTGCAGTTC
ATTCTGAAATAGCAAATCATCCAGACTTAAGAAAATGGGAAGACTAAAGTGAGGGGTTTGGAGATTTTCTGAAAAATCTTC
CAATTCAATGTAAAAAATACACTGTGAAGAAATACACTTGAAAAAGAAATATTTACAGGTCTATTTCTGTTCATATTATCT
GTAATACCCACTCACCCTTTCTCTGTGATCGTTCTCTCCAGGCTGTTCCTGCAGAACTTCTCTGCCTCTGCCAGAGCTCTG
ACCGACGCTGAGACCAAGACTTTCCTCAAAGCCGGTGACTCTGATGGCGACGGCAAGATCGGAGTTGATGGTGAGAAAACG
TTCCTTTTTTTTCTCACAACAATCGCAACTTTGAGAATTTTGAGATCTTGAAATAACACTTAAAATTCTATGTCTCCCTGC
AGAGTTCGCAGCCATGATGAAGGCTTAAATGAATAAGCTGACCAACATCTTACTTCACAACTCTAAAGGAACAACTTGAGA
CAACTCTTCTTCTCCTCTTCCCTCTCACTTTCTCTCTCTTCAACTCCACACCACTTTTATACACTTGACCTTTCACCCCCC
CACCACCACCACCGACCCCTCTACTCCCCCACCACCCTCGACCACAAACAAACTGACCCCTCTTTCTGACTGTGCTCACCC
CTTTGCTGTCCACGATGAATGTACCTGACTAAGTGTATAGCGTGGTTGACTTGTGTCTTTCTCTCTCTCTCCCTCACTTCT
GTCTTCGTTTTACTATGTGAGTTTTGCTTACCGTGAGATCAAATGATGCACTTTTATGGGAAACGGACGGACGATGAGACA
ACAATAAAGGTTCTTTTTCAATAACACAAGTCTGACCCTTGTCTTCAATAGGGCTTATAAAGAATAGTTTGGACCAGACAG
AAAGCCCAGGAATTGGTCTTGATGTCTAACACCTTCACTTTTACACTACAGAAGGTGATTTACATGATGAATGAGGGAATA
GGAAACACATTTGTCAATCCATTAAATGACCATATTAGGAAAGTGATTCGAATGTTTCTTAAAATGCATATTTTCATGAAA
ATCTTCATCCACTGATGTCAAATCATAACAGCAACCACTGTGGCAACCACAAGTGACAGTCGTGCTAAATGCTACACACTT
GGCTAAAATGCTAATCCAATGTAAAAATAGCACAGGTGGAGAAGATTCATAAAGTTAGCAAAGTGACCCAGTAATTTGCAT
ATTTACCTAAAGTTTGTAAACATTAGCTGGAATTAGCCACCATTAGCTACTTATCATATGTAAGTCCTTTGCAAGTCTTAT
TACACGGCAGCCATCTTGGCAACGCCCCCAGGCAGACCCCTATCTAAATGAGTGGGGAAAGAGTCATTACTCTACTTTCAA
TGCTCACTGGGACATAAAAACTGCATGTAAAGAATCACTGGTCCATCTGATAAAAAAAAAAAATAATAAAAAGCTAAAAAG
TTGGTGACTTTGCCCCAAAATAAGGTTTAAAACCACTTTTCCCCACAGGGTATACCAACTTTTTTTAACATTAGAGAACAG
TTTACTTTAAGCATTCAGCTAATGTTAGCATTCAGCCACTTCCTAGCTAAAATTACTGTTTTATAGTGTTACAAGATGCAT
CATAGGAAAGGAAATACATTTATTGTAAAAAATCAAAGCACATTTTGGACACATTTATTTAAGCCTGGAGGTGATTCGCGC
TGGATGTAATCAAACTGTGATGTTAGCGAGGAAGTGTTTCGCTTTACTTTGAAATATGGTCTGATGTGTCTCCAGATTTTC
ACTATCCATCATCTTTTTATTTGCTGATCAATTTCCAAGTGGTGAAGTGATGGCAGTTTGTCAGATTCCTTGTCAGATTTG
CAACCTGGAACACAACAGCATAACATCCCAGCATTCGAGCTTCCCACCCTGAGCGTGACGTCAGAGGAAAATGGTCGCCTT
CTGAATATGGTCTTTTGGAGCACTTAAACAAATGTAAGTTTAATGCCTAATGTTTGAATTACCACACCTTCTTTGTAATCC
TGTGACATCATCTTTTTCAATGCGTATTCGAAGTCTGTTCAACTTTTTTTTGTAAGAAGAAAAATGCGCCATTCCTTTTTC
AAAACTTACATAGTGTGACTTTAAACGTTGCATTTTTAACGATGAACAGGGGGTTGGAAATCTTATCCAGTTGTGGTGATG
TGATATCTGGCACCAACGCTCTCGCCTGCACCCAGCTCCTGCTCTATTATGCAGCTCTCTCCTCTTGTACAGTATGTGGTG
AGGCTTTCATCCCTCATACCCCAATAAAAGATGGCTCTGCATTGATGGGAAGCAGCCACAAGGTGGCGCCTCTGATCTGTC
AATAGGTCACAGGGGTCTCAGCTGTCAGACAGTTTTACTTTGCACACTTGGAGGCTTGGAAATACAGTAAAAGTGTTTTCA
GAGCTTCACTTGAGTTCACTTTTTTAGTCCAACAAGTGGCTGTGAGTTCATGCTGTCACTCACAAAGTGTTAGAGCTTCAC
TGCTGGTGATATGTGGAGGAATAATGAGATTTATTCCCCTGGTTCTCGTTACAAAAAGAATCTCTCCTGTCTCTGCTAATG
CCATTAGTGCTGACTGGATTCACCAGAGACGCTGCTGTGTCAGAGGGTTTAGAGGGCCTCCGTCTGCAGACAACAAACTCA
TCAGTTTAGAGTGGCCAGTTGTCATGGTGACAGGTGCAAAACCCCCTTAGAAAACCTGAGGTGTACCTCAGTTCCTACCCA
GCTCATCACCTTATAGTAGGTCAGATGTGAAAGATGAAAAGCATCATAATGCACAAAACAGCTGTAGTATAACTCCACTCG
TATTGATTATTTTAACTGAACATCTTGGTGTTTTTGGTCTACTGATCCTCACTTTTTTTAATAAAATAATGTACTTTGGAT
TTTTCTTCACACTATTCTTACTTCAGCTTGAGTTACATCCATGTTTAATTTATCTTTTACCTTGTTCAGCAGTTTTATAGG
TAAATAAATTTCACTTAACGCAGTCAGACAGTGGGGTTTTGAGTCTGAGTGTAATATCAGTGTGACAAATAATGTCACAGT
TGAAGACAACAATCCCCCGGTGAATCACAAGATGATTTATAACCTACTGCTCCCTATAATCATCTGTATTTTGGAGATTAT

TCACGAAAACCAACAAGAGAAAAGAGGCCTGATTGAATTTGGAGATGTCTGTAAGCTTCCACGTGCACCTGCAGGGAGAGA
GAGGGGTGGAGAAGGGGTGAGGGAGGGGTAAGGGAGGGGAGAGGGGAGGGAAAGTTGATTGGACAAAAATCACAGTGATGT
GACAGCGAAGAAGAGAGGGGAGAGAGGAGAGAGCAGCGGATATAAACGGTGAAGGCTAATTGGCCAGGTGAGCAGGTGAGG
AACACGAGAAGGAGGCGGCTGGCTGCGGGAAAACTTGATCCGTGGACAGGTTGGAAACTCAAGCATCCGCCGGCCTATTTA
AGACCGAGCCAACTGTGAGCGGACACCACAAAGCCTTATCTGTTACTGCTGCTGCTGCTGCTGCTGCTGCTGCTGCTTTGG
ACCTGGACGGAGAGGAGACCGAGCCAACAGCGGTGAGTCCCACAGCTGCATGGGGAGAATACAGGTGTTGGCCAGATGTGC
TACTACCGTACCTGCGCTGCGGGAAGATGAGCTAGAAGTGTAACCATAGCCTTATATTCTCTTTCATAAGGTTTTGTTCAT
CATTTTTATAAAAGTTTTAGGCATGCTTATCCTTAAATTCTTTAGCCTACTAACAGTAGTAGGCTATTGCTCCAACCAGAT
ATCCACTGAGATGACAAACTGAGTGATGAATGACTTGATAAAAGATGTTTTAAATCACTAACAGGATTGTTTTAAGATCAC
CATCATCTTTTCCCATAAAGTTACAAACATAAGCTAAAACATGTCATGATAATATCTGAACTCCTCAAGCTTTCAGGGACT
GTACTAAAATGATTAGAGAAGTCAGAAAAGCTATGTATTTTTAATTTTTGCACAAGTGAGGGCAGTCTGTACATGCTTGGA
GAGCAGGAGAGGATTTTTTTTTATGATGCCAGATTTATATATTTTTTCATTCATTCTTTGGCAAAAAAGAGTCCATCAGCA
CTAGCAGATGTGGCTGACATTCAGTGTCTGACATTAGGGATATCCATCTCAGAAAATATTAGTTGCATGGTATATGGTACA
AAATATGTTGTTACACTGTAAAACACTGACTTCAGGAGAGTCAGGAAACCGATCACCTCAGAATTACCAAGTAAAGTAGAC
TTGGTTTCGCAAAATATTTTGTCCTGATCAAAATTTGATAGACTTCAGAGAATTCAATACTGTAGATAAGCAAATGTACAC
GGTACGATAACCATCACATTTTCTTAAAACTGCTGTAACGCTGCAACCCTTGTGCTATGATAGAAATTGGTGGCTTGCATA
TTCAAAGTTGTTTTTTATTTTCTTGGTGGTGGTTGTAAAATCACACACAGTATATTGTATATTAATGAACTATATCCCCAC
CCTTATTATTTTTGTACGGTACCAATCACCCTGCACAGCTCAGCCAACCCAATTAAGCAGTTCAAGGGCAGAAATTATCCT
CAGTTATTAAGAGGCCACCGGGGGAAATCTGAACAGGAAACCTGATGAATAAGGCATAGATCATAATATTATTATGAATTT
ATTAAATGTAATGAAAAACATTTTGAAACAAGTCGTACATCTTTTTTGAATCCCACTGATTCTTTCACTGACATTTTACTC
CCTTTTTTGACAACTTGAGCATATTTTGTCAAACATTCGTGACTGGATTTTTTGATGGGATCATAATATTAACCTAGATAC
ATCTCCAAATATGACAAAAGTTAATCCAGCATGACACAAAAATGGACTGGGAGCAAATCAAAAAGCCAGAATTAGGTGAAG
AAAATGTTTGTGTGTGCGAGAGGTAGAGTGTGTGTGTGTGTGTGTGTGTGAATTATTTACAGAGCTTTTCTTTGACTCTGC
AGGACCAACATGTCGCTCACCTCCATCCTTTCAGCTGAGGCCATTGAAAATGCTGTCAAGGACTGTCAAGGTAGCGTCTCC
ACACACACACACACACACACACACACACACAAACACACACACATTTTGGAACTGCCCTACAAATATTTGAAACTACTCATT
ATTCATTTAAAACCCTCCAATACAGAAAACATGGCTCATCTCTCCATGACCTCAACATTAAGATCACAGGATCAATAGCTG
TAACTTTAACTATCTACACACCTAATTGTGATAAAATGCACAGTAATCCTGATCCACGATTGGTAAATTGATCCGCTCATA
ATCTGTGTTCCAGATGTTTCTAACACACTCCCCGTACAAAGTGCATAATCTTCTCTACAGTTTCATTGTTTCTAATATTGT
ACAGCATCCTCTCCAGCTTTAACTGAATAGTGATGAAATCTGAATTTGAAAAGGTTTTCAATCCTCTTGTCTGTTTTAGTG
GAGGCATGTGTCGACTGGACTTAAAAGATAAAAAACGGGCTGGAAAATCTCCCACCTGGACAGCAGCCACACGCTGTTTTA
ATCTCCACATCGGCTCCCACCCTGCTAAACCTAATGTGTCAGCTTCTGTCCCTTTTATTTTTATTTTATCTATGGTTGTCT
GTAACTGTTATGTTTGTATGTATGTGTGTGTGTGTGTGTGTTGTGTGTTTCCAGCTCCGGACACATTCTGCTACAAAAAGT
TCTTCGAGCTGTGTGGCCTGTCCTCGAAGTCGCCCAAGGAAATCAAAGATGTCTTCCAGATCCTCGATGAGGACAACAGCG
GCTTCATCGAGGAGTCTGAGCTCAAGTGTGTGACACAAAGATCAAATATTTTTTTACCTCAAGTAACATCTTTTCATATAA
AATTATTTGCAGAGCTTGTATTTTCTATTTGTGCGTGTCTTTCAAAAAGGTTTACTCCTAATAATCCACTATGGGCAAAGT
GTTATGATTGCATTTGGTGTGTGTGTGTGTGTGTGTGTGTGTGTGTGTGTGTGTGTGTGTGTGTGTGTGTGTGTGTGTGTG
TGTGTGTGTGTGTGTACAGGTACTTCCTGCAGCGGTTCATCCCCGGGGCGCGAACACTGACCGAGGCAGAAACCAAGAGAT
TCATCTCAGCAGCTGATGATGACAGTGATGGCAGAATTGGAGCGGAGGGTCAGTATCTGATTTCTCAGATTGTATTTGTAC
AAACTCATGATGTAGGCAGGATTGATCTCATTTGTTGGACTGAATTTGAGTGGGTGGAGTCAAACAAACACAATCTGAGTT
AACAGAGAAGAGCCAGCTAACTTTATGCAGCTTAGAGCACTTTTAACATACCGAAGTAATGCATTTAGCCATTACAACAAC
AAACAAGTAATTTGCTAACCTGCTTAAATGCAGTATTATAGTCATGAATGATCACATTGATTTGCTATTAAAGCAACATTA
TGTCGAAATTGGCAACACTGTGCACTGCCTCAGGCTTCTTTACTTACAGTAAACAGAGGGCTGACTATGAGGATCCTCATT
TATGTATAATCTTACAGTTAAAATGTAAATGGGCATAAATTGACTAATGATTTCCATCAGTATTCTTCTATATGATCGATT
TAAGTATATATGCAACCATTACTGGCAGGCGGTGTTTTACGATAATGTCTTTGCATTGATTTGATCCTCAATATCAAATGC
ATTAAGACAAGTAACACAGACGATCTAAACAACTCAAAGGAGCAATAACTTTAACATTTTCAGCAGGGGTTTATTAAAATC
AATCTTACAAGCTCAGCTCTGAGGAATCATAAAATGTTTCTTCAGTGTTCAGTGCTTCAAATCAGTAGCCGAGGATCTTTG
TTCATTCATGTTGTTTCAGGTTTATCAGTGTAAAAACCATCTGCACTGATAGTCATGACCTCTCGATTATTGTCCCAGTTT
TGCCAACTGTCCAGTTACCAGCAATATCTGAGCTCAGCCGTCTTACATAACAGGACAAAGACACAGATTGAAGTCTTTATT
GTTATTGTGTAAAACAATGAAACAGTGAACCATCTGTCTGAGCTGCGTAGTGCAGAATACAGATAAAAGAGGTCATAAGAA
AGAAGAATTAAATAGTTACTTATTTAATAGATACCAAAGATAAAATGGTAATTAAGAATAAAATAAGCACTAAAAGGATTG
TCAGGGGAAAAAAATAGAAAAAAGATAGTTAGGAAAAGCACTTGTACAATTTATAAAACACAATTTCTATTCTGTATCAAT
TTTATGTGACGGCTCTGCATAAGCTTAAAATTTGCTTGATTAATGGCCATTCAACATGTTTTCTTTCTCCAGAGTTCCAGA
CTATGGTCCTGTCCTGAGTTTCACAGATGAAAAGAGCTCAAATCTTCAGGTCCTCAGTCTTCTACCCAGCTCTGGGCTCAT
CTGATCCCAGCTTCCATTCCCAGTGATTTATTTTGTTTTCTACTAAACTCTGTGTGCTCTAATTGTTTTTAAATGACCTTT
TGTCCATTTAAAGACGATAAACCTTTAATTTGTCTTTCCATTGTGTTCGTCCATGTTATTTTTCTGTGCACAACTCTGACA

ATTAATGAGATGAATAAACATGTGGAAATATAGATCACTTCTCCATGGTTATCTTTTCTGAAATCATGTTCGATGGGTAAA
CAAATGCTATAAAACACATTTGTCATATTATGTTTCAGCAAAAAATAAAATAAAATGAGGCGAAATGACTGTGTGTACAAA
TACCAGCTGATAGCTCAAGAGTAGAAACCACATGAATGACAGGAGGAATAAAAGCATCAGCTTCTCTGCTGGTGTTTGTGG
TGCTGGCTCAGGTGTATGTGAGCTGACCAATCAGAGCAGACTGGACAAAGAGAGGGGCTTAAAAACAGCATTTCAGACAGA
ATGAGAAAACTCATGTAAGCCTATTCTAGTATGAACCCAAAATAAAGTTATGAACCTGACAATGAACATTATATGTCTCCT
TTAGATTAAGAGGTTCTTCAAATACACAATCTTCCACAACATGGATTTGTCACTATACTAGAAACACATAAGGGAATATGT
TCACAAATAAACATGGGTGCAGACTGAAGTTGGTGACTGTGTATCTCATGACTACATGCAGTGTATTATTCATAGATCATT
TACTAACACATTTACAATTGAACAAATTGAATTAAGTATTTGTTTATTCAGGGCTTTTAAGGTTTATATTCGTTTATAGAG
AAAAGATGTAAGGTATAAAACATTACAGGGATTGGTGTGAAAATAAATGTATGGTATAATCTAAACCAAGAACTGAATGTG
ACGATTAAAGAAACAAATGACTTCCGAATGCATGCACTATACAATCCAAAGTCCAACCAAAGTGTCTCATCACTGTGCAAT
AAAGACTTGTAATGTAATGTAATGTAATGTAATCAAATGCAAGTCCAAAGAAGTACTTGATTAATAATCCTCAGTGTAAAA
TAAGAGCTAAAACTCTTTGTAAACAAGTTGTATTAAGTACACCTTAAATCAAATATTACTGCATGTGAACCCACATCTTTG
CAGAACCCGAGCACGTTTAATAATAACCAAGAAGTTATTCTAAAGTTCACAGCAATTCTCGTTCCCCTTGGACTAGGAACC
TTTATTTGGCTTGACATCGATTTTACCCGAGCAGTTGAACTTGTGACGCCGCGCTGTCAAATACAGGAAGTTAAGAATGTG
AAGTATAATGCGGCATCTCCTCTCATTGATCTCATTTCCTCATTTTTTATTTTTGTTTACGCAACCTACAGCCACAAGTAC
CTGCCCGTGTTGGGTGTATGCTTTGTAAGGTGAGTGTTACCGTCGTCTTACTCTGTTTTTCTCCGCCTGAATCTCTGCAAA
CACAACTCTACAGCTAGCAGAGCAAGCTAGCGAGCTAATAGCTGCTGGATCAGGCTCACCGTCGGCTTGTTGCTGTGCTCG
GTAACAGTTAATATGAAGTAGTTGTAGGCTACATGTTAAGTTACAGAGCCACAGACGACACCATGTGTGCTTAACTCACGA
AGTCATAATTTACACAAAAAAACAGCTAAGTCAGCTTGTGTGTTGTGGTTCCTCTCAGGGTTTTAGCTCAGTTTGCACAGA
CTTCACCGGCTTAACGACAGCAGTCAACGTTGCGAAATTTACATTATCCTGTACACAGTGGTAGCGACACAATGCAAAAGC
AGGAGCAAACATGCATCTGATAGTATTGAAGTATATAAAAAGCGCAGATTGAACTTCTTACTGAGTTTTTGACCGGCTGTT
GACGTTAAATTTAAAACATTTATAGCCTCATAAAACTTGAATGTTATGGTGGAGCAACTTGACACTAGTATGTTGTTGGGC
CACCATGACATGAACCTATTGCTCATATGTTATTGCATTAATAAATTTGAAATTCTTTATACGGATTATTTGTCCATCACT
TTCTGCTGTATCAATGAGTGTATAAGAAACACTGATCAGGATCTTGGACTGCCTTAGTTGTACCCAAGTACACTAAATAAG
CTGGTGAATATGAGTAAATATTAGTTTTGTGCTATTGTTTAATTTTGAGCACCGTCAATGGGATTTTCCTCCAGTATGATA
AAACACTATTAAAAGGTAGTATTATAATACCCACCACTGTATGAATTAGGTGTACACTGATTCCAAAGCCTCAGCCAGGCC
TCTACCATAAACAACACATTTACCATTTTTCCAAATTCTGGTATGTCTGTACCTTCCTGCAGGGATTAATAGGTATACTTT
TTTTGTAATGTAGTAACATAACGTCAGGGATAACTTTGGGAGTTAAAGCTGTATCAGTCGCCTGCCATGAATAAATAAATA
AATGCAGCTGTTATCGGAAACCCTAAATCTTGACATTGACAAAGCAGCTGTGGGTTGGTCATGTCATTGGCAATATCTGAT
CCACAGAATAAGGTCAGCATTTGGTAGGGATGTACGATCCATGTTTTTTTCAGTTCCGATCCAGTTCCAGTACCTGAATTT
GAATATCTGCTGATACCGAGTACCAACCGGATACCAGTGTTTAATTCATAAGCTGTATGTCTCACTGTGTGGATAGGGATA
ATTATTTTACGTGTAAAGCAGGCTTGACTTAAACATTTGTTTCCTTACCTTGTAAAACATAATGCAACAATAAATACATAT
ATATACATTCACTGAATTGTTATTTTTAAATTAAAATATTCAATAAACATGTAGAAACTTTAATTAACATGAATTACAAGT
TAAGATACAAACACATTTAGTGCAAATAAGCATATAGACAAAAACATGAAATATAATTGAATAGAGCAGCTCAATATCAGC
ATTGGATTAATGTTTCCCTACTATTTAGGAACATACTGATCTAGCATAGCATATCAGATCAGTGCATTTCAAGTATAGATC
CAACTTTGCCCTAATAAACATCAGCTGTACTAGTGTGTTTGTTTGTGTGTGTGTTTATCCCTGCAGTTAGAGCTTAATGAT
TCTCTCTAGATCTGTCATGCTACAGTTTGTGATGTACTTGTCTTCATTCTGCAGGCACACGGCATGCTAACCAAACTGCCT
TTGGAACAAAGGAAATCCAGACACAAGTTACACTAATGCTAATGATTAGGTAAAGCATACATGTTTTTGATGATGCAAGGC
TGACATTGACGTAAGCTGGAAGAAATCTAAAAGGTCGTTTATCTGGTTTGTGTTTCTTCAGCTTTCTCTTTGTATCTGTTA
CAGTCCCAGGATGGCCTCAGGAATGCAAGAGAAACAGTACACACCATCCCTGCTCAGTTTTTTCGTTTATAACCCAACATT
TGGACCCCGGGAAGGAGAGGTGAGGTTGATTCCCAGATATACACATACTTTACAGTTTGACCAGTTTTGTTTTTTTTTAAC
TCTGGTTATTAGGAACTTGTCAAAAGTGTAATTTTTGTTTTCTTCAGGAAGAGAAGAAGATTTTGTTTTACCACCCAAGTG
ATGTGGAGAAGAATGAGAAGATCCGAAATGTGGGCCTTTGTGAAGCCATTGTACAGTTCACCAGGTAAAGAGTTCAGTACT
GTAGGCTTGGCATTAATTTAAAAGTGTCCAATGTGAGTTATGTGGCGCAATTTGACATGCTGTTTTAAAGCAAACGAGAAG
GAGGGTGTCCTTTCTTTGAACCCGAAGTATAACAGAACAGGGAAATGTTGCAATAAGTTGTGATATAAAACACTGATAAGT
AACCACTATCTGTGAAAGCAGAAAGGATCGGACTGCACATCTTGTATCTCCTTTCTTTTCACGGTCATGTCTTCTGTCTTC
CAGAACGTTCTGTCCAACAAAACCAGCCAAGTCCCTGCACACACAGAAGAACCGGCAGTTTTTCTTCGAGCCCGAGGACAA
TTTCTGGATAGTCATGGTATTTTTAAAAGCTTACATATACTTCTCTTGTTTAATATTTGCTCTGTTTTTTTGACTGTTGAA
TTTCTTTTGGATGTTATTAAGCTTTCCTTCTCTAATCCTGCTGTTTGTATTTCAAATTCTTTCCTCAGGTGGTTCGAAACC
CAATGATTGAGAAACCAAACAAAGATGGTAAACCTCCCACGGTAGAATATCAGGAAGAGGAAATACTTGTAAGTGCTGAGT
GATGCTCAACATCTGTATTTCTTTCACTAAATATTGTCAGTTTAAACTTTACCTCACCTTACCCTCTTGCCTCTGTAGGAC
ACTGTTTATGGTGCAGTGGTAAGGCAGTGCTACAGTATGTACAAGGTGAGTGTGTACCCAGTATATGTATTTTAGACACTG
TCAGAAGTCCTTCTCTTCTAAAAACCATATAATAACACCCCTATACTACCAGTTTATGGCCTTTTATAATACAAAAAGAAT
AATTTTGTAACAGTGATTATTTTGCTTGTGCTTTAGCTCTTCAATGGCACATTTGGCAGAGCGATGGAAGCCGGTGGAGTG
GAGCTGCTCATTCAGAAGCTTGAAAAGTTCTTCTACAGGGTAAAAAGCTCTCCATTTGAAAGCATGATTGATTATAACCGG

ACAGTGTGCACATAAATGTTTTATTAAAAAGTCACTAGAGCCTGAAAAGCCGTGTTTTCACAAGGTCTTGCCTCCAGGGAT
GATACTCGTTGTACATACAGTGTGTCTTTGTAAAGAGTCATGTGACTGTGTGAAAAGATGTTTGCAGTCATGCCTGATTTC
TTCATAATTGTCTGTCTTTATTAGCTGTCTCAACCTTCATGCCACAGTCAGCTTGCTGGAACGATAAACACATATGCACAA
GTATAAAAAATTAATTTCTAATTAGGGAATACCCATCTTAAGTTAACACGGGCTTCCCTCTCCTCCATGTAACCCAAAGAC
TGAGACAAAGAGAGAGATGGCGGTGGTGTGCCTATCAGAGGTTGAATGTCAGGATGATGCAAGCGTCTCCACACAGCATCA
GTCAGAGTTTCTTATTCCTCTGTCTTTATTGCTCTGTAAGCAAGCACTTGTAGGCAGGTAAACTAACCCCTTTCACCTTCC
CTTCTCCTGTGTCTCTGGGCTGTCTCCTGTTATTTCTGTGTGGGTGTGTTCATTCTGGGGTTTTCAGTATCTGCAGACTCT
CCATCTTCAATCCTGCGACCTGCTGGACGTGTTTGGAGGCATCAGCTTCTTCCCACTGGACAAGATGACCTACCTTAAGAT
CCAGTCGTTCGTCAACAGAGTGGAGGAGAGCCTCAGTCTGATCAAATACACAGCCTTCCTGTATAACGACCAGCTCATCTG
GTACAGACTTGAAACTTTTTTGTAAATTGTGTTTTATTCCCCCAGCTATTGATACTGATATTGAATTTGGAAGCCCTCTTG
AACACACCACCTGCAGAAATAACACTTTTACCGACAAAATCAGCTCTTTACAAATCAAAAATGCTTGCTGCACACATAACA
TTAGCATCCTGAGTGTCTGGATCCCCCAGTTTCCCAATTTTTCCCCACTGATGGCTGTTGCCTATATCCACTTTTGTCTTC
TTAAGGCTCAGTTTTTCAGTTCAGCAGCTTATAAAAAATTAGTTCTAGGTTCTTTAACTTATTGGTAGTGCCTCTCTCCAG
CACACAGCAGCTTTTAGCCATTTTTTTGTTGTTTGCTAGCAGACAGAAGTTGCTAGGAAACACCTTCACACAATACAAAGT
TAGCAGAGATGATTTGTAAGGTGGGCGGCACTTAATGGCACTGTCTCTATCCTTGGTAAACAGAGGTGGGTCCTGTAATCC
TCTTTGCGGTTAACATGCTCACTGAAATGTCACTTTCGCTCTGGGAACAGTGGCGCAGTGTGTAAGCACCGGGGATTACAT
GATTTGTAATGAAATTACTCGAGAGCAGTGTGATCTTTCATTGTCAACAGGCAAACAGAAGAGCTGTTTAATTATTGTCTA
AATTAAATAGCATTGCTCTGGAGTATTCATAGCAGCACAATTTAATTAATTAATATAGCACCCGGCCCCTGATGCAAATTC
AACTGGAAGCACACTGAAAATACTGTCAACAGCCACATATTATTTGGATTTTGGCAGGGCGCGTTGTAAAGGTTGTAATTT
TGTCCCTGCGGTTTCGTCTGCAGGAGCGGACTGGAGCAAGATGACATGAGGATCCTGTACAAGTACCTGACCACTTCGCTG
TTCCCCAGGCACTCTGAACCAGAGGTGAGGAGGTGGGAGTGCACATGGGGTTAGGATAAAACTGTGGAATCTGATCCTGTT
TTTGTTTCACTCAATCAGTATATATACATGCAGCATTAGTCCACCACAAATTCATTGGTTTTGCAGATAGTGAGCTTGAGG
TGATTGTTGTTGAACATGTTCACTGGAAATAATTCACTGAAATCATACATGTCAATGTAGTGAGAACTTCTATTTTGCCAG
AAAATACACTTAATACCTTTTATCAAATTCCTTCAAAGTCTTCACTACATACAGTATATTATGTGTTCTGGACAGATATAA
TTGCAAAGCGCAACTTGACTGCTGCACAGAGGCACACAACCCTGAGGTGGTAATTCTTTTATGATGACAGCACCATCTTAG
CACTGATAGTGTGCTCCCGATACTATGCTCCTGCATGATTACGAAGAATGTAATGTGAGCTGATGTATTTTTATTTTTTTT
ATGTTAATGATCCAGACAGTATGGGGTTTCATTTGAGCCAACAATTTGAACTGCGCTTGTGTTGTCTTGTTGCACCCTGTG
TCAGTGTCAGTTGACTGTTTTGCATGTCGTAATGATGTGACGTAACAATTATATTAATTTGAGTAAATAAAATATTATTAT
TTTGTACACTATCACTATTATTTTGATAAATGTCTGACTTATAAATATAAACATTCTTTTTTATCAGGGAGGAGCTTAGTA
TTTGGAGCACACACAATTGGTGCTAAGATGGCCGTGTAAGAAAAAATAAATGCATACAATGGTTTTCAGTGACATCAGTTT
GGCACAAATGGTGCCTCATAAGACAGAAATTAATAGACGACTGTCCTGAATACGAGCTGTGAATGTTGCTTGCAAAGCTGT
GTCGAACATTTTCCACTAACCCACTCTTTACTCCTCATAGCTGGCTGGCAGGGACTCTCCACTGAGGCCAGAGGTCGCAGG
GAATCTGTTGCACTATGGGAGGTAAGTCCCCGATCACATGTATCTGTATCTGTATCTGTATCTGTGTTCGTCTGCAGAGGG
TCCCGTAAAAACAAGAGTGGGATTTTGTTGAGATGTCTGTCAGTGGAATAATGTCTTCAATGTAAAAGGTGTCCAACATTA
CACATCAACAGGCATACTGTTTACATCCACGTGCATGTGTGTGCACCAGCGTCTTGTGGTCTCGACCCACTGACGGTAATG
AGCTTTGCTTGCAGTCAACCAAATTATTTAAGGAGGCAACTCCCTTGGCTAGCACTTTGTATTTAAATAAGAGAGCAGGGG
GTAGAGTTTCACTGCACGCTCGGGGAAAGAAAATAAAGTCTACTTTCTATTCAGTTTCAGTGTACATATGTAACCAGAGAC
AGTTTGCTCAAGTGTTTTCCGTCTGACAAAACTTGTGTGTGAATGCTGCTTCAAGTCATTTGTCCCTGAAGGTGAAGTGGA
GCTGTTGCAGTAGTCCAAGGCTGACTCCTGGTGACCTATAAGTATAAGGTCTGATTGAATGACACTCGAGAATATAAAGAA
TATTAAATGAAGGACAGCAAAATTGTTAGAACTACGTTGAAGTAAAATAGGTTACAGATACACTTGGAGAGAGTTGGAGAA
ACACATGTAACACAAAGACAGGCCTCAGTGCTCATGGTTTATAATATGTGGCATCCTTGGAGTCTTGTGTATAAACAGTAT
AACAGTGAGTCCTTTGTTCAGTTTGGATCTGTCATGAAGGGATACATGGTGAAGAGGAATGAATCTTTTAGTTCTTTTATA
ATCAGTATGATATGGTCTGGTTCAGTCTTTTCAACACTTTTGTCCAAGATATCTTGACGACTACTGGCTGTGTTGCTGTGT
ATTTGGTACAAATATTCAGGAAAGCTGATGATTCATACCCGTTTTGGTAACCCGCTGACCTTACATCTAGTGCCACCATCA
GGCCAAACCTTCCCCCATGAGCAACACTTAGACTTGTCTTATGACGCCATAATGAATACCACAAATCCTCGCTTCCAATGC
TTTTTGCTGGAGATGTACAGGATCCACTTTGAATGAATCTTGAGCTGAAATGTCAATTAATTAATTAATTTTTACAGCATT
ACCGGCAATCTCTCCTAAATTTGTTTTGTAAATAGTCTCTCAGGTAGGCTTGCAGTTTTGACTTTTTGGACTGTTCATGCT
CTCCTCTGTTTTCTCCAGGTTTCTTACAGGACCTACTAACCTTAAAGACCCTGAGGCCAAATTCAGATTTCCAAAAATATT
TGTCAGCACAGACGATGGCTATGAGGAGCTGCACCTGATCGTTTATAAGGTAGAGTAAACATGATCTGCTATTTTTGTGAC
AGTCCATAAAGGAGTTTTTTAATGTATTAAAATAGGGGGGAGTTGTTATTCATTTTTATAATCTGTGACTGGAGGATTTGT
TTATGAACAGCAGATTTAGGATGAAATATTCCACTGCTCTATCACACGATATGGATTTTTTGGGATTAGACAGCTGAATTT
TAAAATGATGAAGTTTGCCCACGATGCTTATCATCATGTTAATGTGATTTCTAACCTGTGTGACCTTGATACTGCATTTAA
CCTGCACCCATCTGCTCTTTTCCAGGCGATGAGCGCTGCAGCGTGTTTTATGATCAGTGGTAAGTGTGCACCCAGTCACCC
ACCTGCCCACTGAATAAAAAGAGTCAACTTTCATTTCCCTTTTCCTGGTTAATTTATTCTGGTGCCGTTTTACTGCTCATA
TAAAGCTAAAATGTCCCTTTCTCTTCAGATGTCCCATAATGTCCGTCTCATCTCTTTTCTAGCCTCTGTGGAGCTGACGAG

GGACTTCTGTGAACAGCTGGACGGCTTGGTGGGCCCTCAGCTCACTTTGTTGGCATCAGACATCTGTGAACAGTTCACCAT
TAACCGCAGGATATCAGGGTCAGTTGACCGCTTAATTAACAATATTTCTAATTGCTGCCAACAATTTAATTACAGCAACAA
CTCATGCATATATTCATTATTAAATACTTTTCCCCTCATAAACCATTTAAAGTGAAATATACGACATTTTGATGAAATTAT
TCCTGCCATAGGCAGAACGTGTTTAAAAAAAAAAGGAAAAAGAAGAAAAAAACTTTGGTGACATTTTAACTGCCAGCTTAT
TAAAAATTCTCATATTTCAAACTAAATTCTTCAGTTTGACTTTCTTGACTAAATTATCAGTGCATTAATTGAAAGAAAAAA
TAGTTGGTTGTAGCCCTACTGTAGACTTATTTGTTCATCAGTTATTTATGTTAGTACTGCATCATAAATAGTCAAAAACTG
TTTCCAGAACATTCTCAGAATTAGAGAGAATGTAGCATAAGGGATCGAGATGATTTTAATGAAATGCAGTCCCATGATTAG
CCATGTTGTTGTCATCAGTTTGTATTAACTCTGCCTGGCTGCCAGCGTTAGATGAGACTCTAATACCACTGGATATCGTCA
GGGCACTACTTAGGGGTAACTCAAGCAGAGTAGCTGTAAAGACTGGCATCCTGGCCCGCTCTCCTTTAACCCCAGCAGTTA
GCTTCAGATTCGTCCGCGCCTGTAATCCAAGACTGGACTCCCTGGCACGGGCAATTTGATGGGCCCCTTTTTTAATCACCC
AAGTGAAGAATCCACTGAATAAATGATGGCGTCCCAAATTCCTCTGCTGGCGTTCAGGCCTGTGTCCTGATTTGGCCTGTT
GGAACTGTAAATAATTAGACGACGCAGAGATAAAATCAGTTGTATGGCCTGTTTGAATACCTACACGCACTCTTCCAGAGA
TGCTATTACAGTACTGTTGTCAGGTAAGCTGTGGTAATAACTGTATCCTCAGTTGACCTTAACGTGGGTGGACAATGGAGT
GGGCTTAATTAAAATGCACAAGTGGCTTTTTGGTGGATGTTATCATCCAAAGTGATTTGGCCTTCCTGTATCATCTATTAA
TAATGTATGTTTACTGAATGGCTTGCCACAGAGACACCCAAACTCTTTGACTGGACATCGTTAGTCTCCTACTGATGTATA
CAAGGGCTATAGTGTTAAAACAAATTAGCAAGATATGTCTTCCCTCCATATCTGCTCAAATAAAAGCTGAGGGCAGCACCT
CTAACAACCAAACAATGTCACCTTGAAAATGAAGTAGCACCCTTTCAAAAAGATGCATATCATAGACTTGTCCTTGTTTTT
CACAAGCAGGGGGTCATACAGCTTTAACATTTTGTGTCCATTCAGGCCTGAGAAGGAGCCGCAGTTTAAGTTCATCTACTT
CAACCACATGAACCTGGCGGAGAAGAGCACCATCCACATGAGGAAGACTGCCAGTGTTTGCCTCACCTCTGTCCACCCCGA
CCTCATGAAGATCCTGGGAGACATCAACTGTGACTTCGCCAGGTGAGGCAGGGAAAGGCTGTGGCTGTGTTTATCTCGAAG
GCCTCCACACATTTGTTCAAAGGTTTTTCCTAAATATATCGCAACATTATTTTCAGGCCTTATTGCTCCCAGCCCTAGCAG
GTTTAATTCCTTTTACACAAACCCTTCAGGAATAACTCGTAGATACAATGAGAAAAGCATCCAAAGCCAAGTATCCTTGAT
ACTAAGCATCCCACTCTCTCCCGAAAAGGCTGAACTTGTGGAATATGTGGGCGTGCTGATACACATTCCTTGCAGCGTCAA
CACTGGGTCCCAGCTAATTTGGATTTCTGCTGAGGGGTTTTAAAAAAACCAAATCAAAAATTTACTACAAAAATCCCCCCA
CGTCAATGATTTAGTGGAGCTACACTGAGTTTCCCGGAGACATTTAGCCTCATCAGGGATTTTCATCTCATTCTCCCTCTC
CCAACTCTGCCTGACATGATTTGTTGAACTAGCTGTGTCATTTTCATAGATAATGTGATGTCTGTAGGAACTTTGTAAACT
CACTCAGTTTAGATGCAGTCATGAGACTGTAGTCATGTGAAAGTCCCATTTCCAGACTGTGGGACATAAAGTGGCATCTAC
ATTCATAGCTTTGATGCTGGTATCAATATCAGTTCCTCTTTTTGCGCAGATGTTGTACAGATGTTCTTTCCTTGTCTGCAT
ACTCAAAACATACACACTGTTATCTGTGCAATTTAGATATTGAAATGTAGGTAATTTCTTGCGGATTTCTGTTGTGTTTTA
ATGAGAAATGCATTCACTTCTGACTCAACCCTTGAGAAATATTCAATTATAGGACATTTTTTCATATATATATATATATAT
ATATATATATATATATATATATGAGGTTTTTGAGTCAGTGTAGCTAAATACATTATCTTGGAAGCACAGTTCCCCTCAGAT
ACAAAGCTGTAAGATAACCTTCTGTAAATGTGATAGTTTCCTTCACTTGACGTTCTATTTGTCTCCCAGGGTGGATGAAGA
TGAAGAAATCATCGTAAAAGCTATGACAGATTACTGGGTAGTTGGAAAGAAGTCAGACCAGAGAGAACTGTACGTGATCCT
GAATCAGAAGAACGCCAATCTGATCGAAGTAAACGGTAAGTCTCCACCAATTGGCAATGACTAAATCTGTGTCAGTCATCG
GTACTCCAACCTTAATACCCTTTTCTCTTTTCTCCCTTACAGAGGAGGTCAAAAGGCTTTGTGCAACACAATTCAACAACA
TTTTCTTCTTGGACTGATGCAGCAGACAGACAGAGACTGTTCCACCACACTAAAAACTGTCAGCGTTGCAGGGTATCATGA
CAGCATCAGCGTAATGCAATAATTGCATTGTAAAATAAGAAAAAAATAAAAAGCTCTCTCACTAAGTTTTTTTTTATTTTT
TATTTAACCTATAAAAATTGTAATTTTCTCTTGTAATTACATGTAAATCCAGATTTTTACTGTACATGTACTTTATTTTCC
GTCTTTCTTTGTACATACTTGTAGGGTTTTGTTCACTGGACATATTGATTTCTTTGAAATTCCAACTTCTTCATGTTTTTT
TTTTGTTTTGTTGTTTTTAATGACCACATCATGATAGTGTCTCAAATTAATTTAAAAACTGACATGCACAGAACAGTTTAC
TCCAGGTTCCTTTGATGATCAGTGATGGTTTTAAAATAAGATGTCATTATGTCAAAACTATTGTGGTTTGTAAACAACTAC
GCTAACTTTAAATAAATTAAGAAGTCATTTTGCGTAGTGTAATTTTTCTACTTGTTGTTGAAGATGCACATTAAGCGGATG
TTTTCCTTCCTTCCAAATAACAGCATCGTGTTTATCTGAATATTATGGGGCCCATTTTCCGATCACGAGAATAAACTTTAA
TTTTAATTTTAGATGAACATGTCGTCATGGAATTTGACCCTGGAACATGGAGGAGATTACACGTTTATTGTTTCAGGGACC
TGATGGAATAACTTTGTGAGAAATGTGAAATGAAAGCTCCTTATAGTAGCTTTCTTATGAAAAATCTACAAATGTAAATCT
GGAGGACAAAAACAAATGGTACATATTTCTTAGAAGAAATGTTAAACAGAAATTGAGACTTATTGGCCTATATTAAGCAGA
CTGCAAGATGAATATATATATATATATTTACTGTGACATATATCGAGAAAAACGTGTGTTAATATTGTATATCGAATGAAAAA
CATGACAAAACACGAGTAACAGTAAATTGATACATTTGCACTTCACACTACAGACCAATAGATGGCGTCAAATCATGCAAT
TGTAACCATCGAAAACACCTGAAGAAGAAGAAGAACCAATCAGAGCGCAGTGTTCCTGTCTGGTTTGCAGCCAATCAGCGT
CACCGTAGATTCATTTCAAAAAGTCACCCGCCATGATTGTTGTGTTTTTCAGGCTCAACTGAGTCCGTCCGTTGTGTAGCA
AGTTGTTTTTACTGGACGGCCCGAAGAGGAAGTTTGTTTTTATACGTGTTTTTTAAAAAGAGAGTACGGTAAGATGGCTGT
GTAATTATACATTTTCCAAAATAGTTGTAAACCTTTGGTTGCTAAATGAACCGTTACAGTGGCTCGTGGCTTCCTCTTCTT
TCAGGATCAATATTTAGCTAGTTGAGTTGGTGGCTAACGTGTTAACATACAGCTTGCACCAATCTAGTTTTTCGCAGTTTT
CACGGACTTAAACATTAGCAAGCTAACTTAACTTGGTTACGGTAAGTGGTCGAAGTGAAATTGTTTTAGGCTTTGATAAGT
TAGCTAAACTTGACGTTAGATATTTGAACCACTGCCTTTAAGCTGTCGTTAGCTTCGGTGTTGTGTAACGTGACATGGTTC

AGGAAAGTTGTTTCTTTATACACACAGAAGGTTATAACATGTGCTGACTCACACAATGGCCCACTTTCGCCACTTAATGCC
ATGTTGTTTGGTTTGGTTGATATAACGTTAACATTAGAGTATTACGTATTTCTTAAAAAAACCTTTAATCCGTCAACGTTA
GCGTCAAACTTTTAGGTTTGAGTCAAACCTCCCAGGACGTAAACAACAAAGCAAAAAAGAAATAAAATTGTGTATAGTTCA
CATATTTGACATAGCGTTTGTCGTGTCTGCTAATTTTATTTATTTTGATAGGAAGCTGCTTGTTTCATATATATTTTGATT
TGCCATCAATGTTTTGAATTTTGTTGACTACAAAAAGTATAAAGCGAATGACATGACAATAAAAGTCTGTTAAAGCCGTTT
GTTTCATTTTTTAAATCGAATTCCTATACAGTCACCAGATACAAGAGCTGTAATGGTTTACCACAGTACAAAATAGGTATC
ATTATGTTATCTAAAACCCCTTTCCAAAATGAGTACCTCCATTATTGTGTAAGTTTCATGGTGGCTAGCACATCTAATATG
ATGTGTTTCGATACCTCTGATAACCATCTCAGGCAGTGTGCTTATAATACATCATCTGTTCCTCTTCCTATTGACTGCAGA
TACCTGAACATGGAGTCTCGATTTTCTCACCTGCGCCAGAGGGACACCAGTGTGTCTATGCTGAGGGTAAAATTGTCCCGT
AGAAGGTCTCAGTCCCAGAAGGAGAACCGAGAGCGAGCCGTGAACACACGCAGGCATCTAGACAAGCTCCCAGAGCTGGAA
ATCTCTTCCCTGGATGCCTCCATTGCGATGGCCAACATGTCGACCATTCATGAGAAGACGATGAACAGTACAAAACTTGAT
AAAAGTAAGGCTTTATTCGCCATCACTGCTAAAACTGTGCCACCCTTTTTTCATGCCCATAATTCTAGCTAATTTGTGACT
CACCACACACCCTTTCCTCCACAGAAATGGCCTTAGAAGAGAGGATGAAACAGCTTGAGCGCTGGAAAGAGCGTAAGACTC
TTGAGAAGGAGAAGGAAAGGAGAGAGAAGGAGCGTAAAGGGATTTTCAAGACTGGCGTGTATCATCCGAAGGACACTCTTA
TCATTGTCCCAGCTGCCTCAACCAGAGCTAAGGAGGTGAAAAGATGACTCGCACGTAGACTAGTATTTACTTTCAATATCT
TCCCCAAATGTAGGACTCCAATGATCAACATGCAATATTTGCTTGTCTTCCAGACAAAAGTGAACACAGCTCCACCCCAGA
GTACCAGAGTCACTCGTTCAATGAAACAGCAAGTGCAGAGGGTACTACTTTCTCTAAAATATCTTTCTCACATGTTCTCAT
ATTAATTTCCATAACGCTAAAAGCTAACATGTGCTTTGTTTCTTTTGCTAAAATTTCTACAGCCTCTCAAGATTCAGGATC
CAAATACTGCAGCAAAGAAAGGTAAATTTAAGTGAACGTGCCTTTACTTTAATAGAAGCAGACTGTGGTGCTTTTTAGATC
TGACCTCTGACTCCTCCTGTAGCTCAACCTGCCGTGGAAAGATCAACTAGGAGCCGGGCAGCCCCTGTCAAGCCTGCTCCT
GCACCTGCTCCAGCAAAGCAGACCAAGACCAAAGTTTGTGCAGGTAACAAAATAAGAATCGAAATCCTCACACATAACTGT
ACCTTCCGACGAGCTATCGTGTTGGTTTCACCCTATTGTGTTCCCTTATTTGCATCTCAGTCTGTGTCTCTGTGTCATGTC
TTCCTGGTGTAGTGGAGCCTGTCGTAAGACCTTTGTCCACCAGATCAGCCAACAGGCCTCCTGTTACAGCAGCTCCTGTAG
TGAAGGACAAACCTAAGGACAAGCCTGCAGGTACACACAGAGCAATGGCCATATTTGAAGCAGATTATTATTATTATTATT
ATTATTATTATTATCTTTATTATTATTGTTATTTGCTGTAAAAGCGCTGGAGATTCAGAACTAAACGGGGAGAGACACTTT
TGTTTGAAACTACTTGTAATATTTTTAAATTCCTCTTTCCCCTCAGATGTAAGACCTACAAGAAGCAGAGCATTTGTCAAC
ACTATGGCCCCCCCTTCTGACGAAGAAAAGAACTGTGAAGGTATCGTTAAATGTAAAAATATCAAATATCCTTTTTGTTTT
GCCTGCATGGACTTTTTAAATTTCTGACACAATGTTTTTGTTGATAACCTTTCCTTTTGATATACAGAAACTGACAACAAC
TTTGTCCAGCCTGCTGTCCCAAAGGTGAGCATCTCTGGGAATTCTACACACGTTGTTGTTGTTGTTGTTGTTGTTGTTGTT
GTTTTGATTAATGATTAATTTGTTTCTGTCTTGCGTAGGAGCCTGAGCTGGATCCTGTTGAAGTGCTGACCTCCTTTGCTC
CCCAGGGTTTTGTTTTCCAGGCTCCCACTGGCTTGTCATCCTTCAGGTTTGAGCCTCTCACTCCCCGCTCCGCAGATGCCT
TCTTAACACCAAGGTCTGTCTGTTTTCTATCTTGTTTGTTTTATAGTCATTGTTGAAGATATTTAAGACACTTTAAAGCTT
TTGTATTATTGCACGTAAGCCCTATTATGTAAATAGTAAATATTAAACCCATGCTAAAGATTTTTGTCTTTGACGCCTCTA
AGAAATGTAGTTAAAACTTGCAATGTTTTCTTTCCTTTATGCCATTTTGTTACTCTATGCGACTTTAGCCCCACCTTCTGT
ATTCCTGCGGCCCCTGCGTTTGACACTGACCCTCAGGCTGAGACAAGCGAGCCCTCTCCGCCTAAGTCCCCCCGCCGCTCT
CCTCCTCCCACAGTGGCTCCTCCAACTCCTGGCAGCCCCCTGGAATCAAAGCATGACGTACCATACTTTAGGTGACATACC
TAACTTTTGCTGCATTTCTTGTTCTGATTTTGCAAAGTCAATGTTTTTAAATCATTTTCTTATTTGTATATTATTCAGATC
GGAAATTGCCAATGAGACAGACAGACTGATGAGTCTTTGTGTCGTCTGGGAGTCTAAAGTGGAGGATGAGTCCATCCCAGA
AGAGAGTGAGTTTATCTTCTCACCATCCTGAATATGTAGCCATGTTTTAAATTTAGTGCTGTAAAAGGTGGTGATCTTAAT
TCGGGAAACATGACCACATCCATTTAACAAATTAGTGTAAATCGATGTCATTCTTGCTCTTGTCTGTTCAGTGAGAGACCG
TATGCGTACAGCAGTCGGCCAAGCGAGGCTGTTGATGAAGGAGCGCTTCAAGCAGTTCAGCGGTCTGGTGGACGACTGCGA
GCTGGGCCGAGGGGAGAAGATCACCACCTGCACTGACCTGCAGGGATTCTGGGACATGGTTTATTACCAGGCGAGTGCCTC
ATAAGAAGAGAGGGACATCACTTTGTTTCAGAAACCAACCACACAAGGAAGAAGAAAGATTAATATTTTTTCTGCTTTTTT
GCCATCTTTCAGGTAGAGGATGTCAACAAGAAGTTTGATGCTCTGAGAGAAGCAGAGAGTCGTGGCTGGGTAGAGGAGCAC
AAACCACTGCCGCGACAGAGGAAAGTAGTGAAGGTGAGAATGGTGATAAAAGGAAAAATAATAATACAAAAAAAAAACTGT
CCATCCTGGGGCCCTTCTTTATACTCTATACACTTTAGTGTGTGTGTGTGTGTGTGTGTGTGTGTGTGTGTGTGTGTGTGT
GTGTGTGTGTGTGTGTGTGTGTGTGTGTGTGTGTGTGTGTGTGTGTGTAAGTGAGGTCTCTATTTCTGAGTTAAAATAACAGT
TGACCAAATTATTCCTGTCCTGTCCTGTCCCAGAAACCATCAGCTGCACCCACCAAGCCAACAGGAACCAAAACGGCAGCC
AAGTCTCGCCTGGCTGCAGCCAAAGCAGCCATGAAAGCCAGACAGCAGGCAGCGGAGGCAGAGAAAACCGAAACGGATGCT
GGTACTGATAAGGACGACACCAGCCACAACTCTCAGGAACCACAACCCCAGGCAGAACCCCAACCGACTGACTCAGTGGTC
TTTGATGGAGGCTTCTTCCAGGTGGAGAGTCCAGCCAAAACATCAGGTGAGAGAAAAAAACTTGATTTTCATTCATCCTGC
TGCCTGATCTATACGATTTAGTCTTAAAGGTGTTTTGTATGTGTTGATATCTGTAGGTACAGTGAGGAGATCCAGCCGTCT
GAGTGCCGCCGTGCTGCCTCAGGCCTCTCCCTGCTCCAACTACCTCTCTCCCAGAAGAGTAACTCGACGATCCCTAGCACT
GGCACTGACTCCTGTTAACGCCGCTGCCGCCTCTCCTGCTCAGCCCACCCAAACTCCTGCCCAACTCCGCCTTACCCTCAA
CCAGACCCCGGCGCAAGCGCCAAAGTCTCAGCATGGCACTCCTCAGTCATCCCAGAAGAGAAAGGACACTGTAAATGTCTC

TCTTTGTTTTTCACCTGTGAAGGAAGTGCTTTCAGACAACACCCAGCCTGAGGGAAGCCCTGCACACCAGTCAGAAACGGC
TCCTGAACTGAGCATAGCAACACCTGTACATTCACTTCCATCAATTTCTGTAGTTGAGGAGCAAGATGAAGCAGCTGAAGT
TGTCGATATTGACCTCCCTCTCTCTCCAAGACTTTCCCCCTCTCCGTGCAAAGTACCTCTTCCGGTCTCCCAGGCCCCTGA
GCTGTCAGCGTGTCTGAGTTTCACGTTGTCACCCTGCGTGACTCCGAGTCAGCCTCCAATCTCGTCCCCTCCCACTGTGCA
GGGCCCAGTGCAGACCCAGGAGTCTGTGTGTCACACTCCAGACAGCTCAGTTGTCGAGGTAAACGCATTTTCAGGTTCACT
GTACTGAAAAATCTCAGCCTCATAGGATGCCCTTTTAATAAAACCTCTAATCCAATCTTGCTAAACAACAAAGTTATTCTC
TTGTCATTTCAGGAACTTCCTGGGTTGGACTTTGAGCGTTACCTCCAGCCTTCACAGAGCTGCAGCTTGTCACCCATGGGG
GAGCCAGTTGCCATGGAGACCCTCTCACCTATGGCAGTGGATGTCGAAATGGAGAGTCCCAGAAGTCAATCTGAAGACCTG
CTTACTCAGCAAGAAGCAGGTATGATTGATGCCCATAGGTACCGTTCATGCACGCACAATGTGTACGCCTGGGTTTCATAG
CTCAGCACCACTAATGCATCCGGTCGGAGTTGGGATTTGTTTTAAATGAGCAAAACTAAAATTCATACTGAAGGAATTGCA
GTAAAAGTTGCGAAAGCAAGATTATTTTGGCTTCGTTCTTTCTTTTTTTATTTTAAAGAAAATGTTGTACTTGCCTGGTTA
TAAGGGAGTTACCACACTGTATTCTTCTGGCACAGGTTTATTTTGAACAAAGTGCACACTTGTATGTCTTAACTCACTGTT
CTGCTCACTTCTCACTGTCATCACAGCACTGCCACCGGTGTCTTCAGTGTTAACTCCTCAGTCACCACAGGTCAAAGTTTT
TCCCTTTTTTTTCTCTTTTCTTTTTTAAATTATTTTTTACTTCCAAATATTCACTTTTAATCCACACTGAATAATCAATTA
AATTAGCTTTTATTTCTTCTTGTATTTCTTTATTGTGGCTTTATTATTGCTGAATATTCTTTCTGTATTTCTCAGGCCCAG
ACAGCAGACTCTGCCCTGCTTCTCTTCACCCCGGACCTGAAGGACCGGATACGCCAGTCTGTCTGTCCAAGTGACCTCATG
GTCTTCACCCCTCCTCTCTAATGTACAAAAAATATATTTTCTTTTACATTTATAACTTTGACATATTTACTACTATTAACT
CTTTTTGGTGAAAAAAAAGTATCTTTGTGATTCTGACTTTTATTTTTAGATCAATGGAAAATAGTTTTGTCTGCAGTTTGA
TCTTATTAAAGTTTTAGTTACTACAAGGAAATGGTCAGTACTCTTTATTCACATCTGAAGGCACAAATGTACTCGGTGCTT
TATTACTATTGCTATTGACACAGTATGAGATTTTACTGTATTCAAAACCTGGATAAGCAATGTACATGATATGATAACACT
TAATTTTCCTTACCATGAAACTGGTACCACTGTATTTGAAAGCATTTTGTTAATTTTTTATACTGTTTGGATAGTTGAGGT
ACAGAGCAATTGGCTTCATCCTGTGTTCGTTACTGCATCATCATTCTACCTGTAACACAATACAGCACAACACTAACCACT
ATACATGTCTAATGGCACAATCATGTGCGGAGCTGATATTAAAAGCTACATGGAGGGAGAGTGATGGGTGCCATCTGGTGG
CTGTGTTTATGACTAAGGAGTGTGGCTTGAATTCTTGATGCAAACAAGTTGAAATACAGTAACATTAAGCCATTAATTCTG
CTTAGACTTTATCTGTGTCAACTAGTTAACTAAACAACTCTTGTACTTGGTTAAATCTCATGAGTGGTATAAACTCATACA
TAGACTGAGCCATTGACTATATAATGCTGCCTTAAGTGTGCTACCTGGCTCTTTATTGGTTCTTGACCAGAGCAATGCATC
TCTTGGTCTCCAGGTGGAGCTTTTCTTCTTTCATATTTCTGGACACTAACTGGTAATGTTCAAACGCTGGGAAGAAAAAAT
GGTTGAAGAATTGATGGATGGTCTTAATCCAAAGGCCCAGTTCATGGTCTTGGACCTCCATTCCTCTTGATTGCACCCCAT
GTCCTTCACCTCTAGGTTCTTCTGTTTGTTCTACAGGAAATGTGAGATGTGTGTTTATAATATTAGAAAGTATTTTTGAAG
ATTAAAGCCAAAAACTAAACTAGTAAATTACCAAGAAAACAAACCTGCAGACTGAGGTTCCTCATTGACTTTAGTCTTGAC
ATCTTGTGGACCCTCCATGTCCTGGGTGTTGGATAATTCTGCTGTGTCACTTGATTTCTCTGTCTATGATAAGATGTAATT
ACATGATTATAAAGATTACAGAATATTTCAGCATTATCAATAGGGGTGGGACAACACTAATATCAATATTGAATATTTTTT
TAATCTTTTCACATAAAGATGAGTGATTTCTCAACAGATAGTCATAACTTGTGTTGGCTGTGAAAAATTGTAAATGCAATT
CATTGGAGCTGCCACCAGGATAGAAAAAAACCCTGTAAATTGTTTTCTTGTGCAGAATTCTCAGCTTTACCTAGTAGTATA
AGTATAAAAGTCACAGTTTGCTGGAATGACTACATTCTTTACATAGCGTGGTAAGGTGTGCAGAAGGCATCAGTGACTCGA
GAAAGAATCTGACCGACTGGGAAGGGCTGTTTGTAGTCTGCAAGATTTTCTCGCTGGCTTCCCCCTCAGGTAGATCCTTGG
GGCTGAACAGTGACTGGCCCTCCTCCTGGCCTACAGAAACCTGCTGACACTTCACCTCAGCGGAGCCCAGGAGTGCCTCAA
AAAACTCCAGGAATGTGATCTGCAGCAGAAGGTGTGGGACAAGGACAATGACACAGAATGTTAATCTTTAAAGGACAATGA
GACATAACGAATGAGTCTGTAGACACTATAGGTGGACCTCCAAATCCAGACAGGAGGACAGGTTGCTGGGGTCATGGCTCT
CTGCTGTGATGATCTCCATTAATTTCAATGTGGTCAGGTTATTGTCCAGCAGATGGAGATCCTAGCAGAGAGAAAAACAGT
CGACCACTAATGCAAACTGAATGTTAGTAGAAGTTCCTTGTATTTATAACCATACAATACATTTAATTCTTTAATCGAGTC
ATTCACAATTGGCCAACCCTCTGGCTTTTTCTGTAGTAACAAGCAACAGGATTTGCATCCTTTTCCTATGGTTAGTTGGGA
ACAATAATGCAGCAGTTAAAACATATGGTTATCTTAATGCTGCAGTGTGTTGCACCTTAAACAGCCACAGCAGGTGCCGGC
AGGTCATGCTTTGATCATCTCTGGCGGCCACACTGACTTCGCAGTAGGTCTGATAGATTTCCCAGCTCTTCTTCAAATAGT
TCACAGCCACCACAGCACAGTCTGGCTGCTTAAACAGGAAGTCTGGATCAACAAAGTCATAGTCAACAAACACAGCTATTA
CAGTAGGTTCACTGTAATAGGTTCACAGTAGACGGACAAGGAACCTGCACACTGATGTTCTTTTCATCATTATTTTGCTAA
ATAATGCTTAAATATCTGGTTATGCTGCCATATTTTCCACATTTGAGGCTTCTGATGCTGTAAATTTTCAGTTTGCAGGTT
CATTTGACATCTAATACGTGTGTACACATGAGCCTTTAACATTACAGTGTTAGCACCTTTTACATTCTTAGCATTAGGAAG
GATGTTGTCTGTCATCAGTTTGGAAAAGCAGGCAGCCAGAAGGTTATTTTGTGACCTGTGAAAAGAGAAGTAATGTTTCTT
CACCAAGGCTAAAAAAAAAGTTTATACTAAGTTGTGTCCTTACATTGGTAGTTTTTTTGTGTCCCCATGTTGCTTTTTGAG
CAAAAGCCTTGAGCTATCACCAATAGAGCACATTTATAGTTTGAAAAATATCTGACCTCTTCAGCTCTGTTCAAACTGGAA
AATATTTAATAGAAGTACTCTAGCACTACAATGAAGCACTAGAGCCATTAGCTGTTATGCCTTTGAAACGATATGCTAGCA
TGGCTAGAAACTTGGCTGTTTTTCTTAGATTCTTCATCAAGAGTTACAAGTGTTTCATCTAATGTTGAAGATCTATTAGGA
GTCATCCCTTACACCATGTCCTTATGGTAAATGTGGTAGGCCACAACCACAAGATAGCTGAGGAACCTATGAAGAAGTATA
GTGGCAAACGGAGAGTGGATCTCAGCTGTAGTGACATCCTCTGAGACACACAGAGAGAAAAACATTAAGCAGTGTCCAAAA

CTACTGTAATTATAATGCCATTGATGTCCTCTCCTGTGTCTGTGTAGGTTCTCACCTCTGATAAAACGGTCAATCTGTGTC
AGAGTGATGTCTTGATGGTGGATCTTACAGTCTTTGAGCAGGCGCCAGAACTGCAGGCGTGTCAGCAAGGAGGTGTTATTT
GGGGAGCGCGACCGGCCAAGTCTGCTGTAAAAACTGTAGATGGACCTCAGCTCTGTGCCATGCATCATTACCGCAAACTCC
ACCTGTTACACATGAGCATCAGGATCCACATTGAAAATAAGGATGTGGGGAAAAGATTTTATAGTGACGCAGGACCTGACC
TTTGTGCGCTCGGTGCCCCGCTCTCTCTCAGGGATTATATCCAGCAGATATTCAATGCTCAGAGCCATGTCTGTTGAGATA
GATGAATCACTGCCTGACAGAGAAAGTGCACCTGTAACAAGACACAAAACTGTGAGCCAGTGTACCAGCATACTGTAACAC
TATTAATGATCTCCTTCATGTGAATTTTGAAAATACTGTAGGTGTTTACCACATGGAGGGATGGGAGCTCCATTTCCTCTC
AGATTGTGTGTCATCATCTGATCATCCACAAGCTCTCCTACCTTAAAATTGAATTTTCTTTAACATATACAGTATGTGTAT
CATAGGTGTGTTACTGAATTTGAGTGAGTCAAACAAGTGGCAAAAAAGTAAGTTCTGAGTACGTTTTGATGCAAAATGCAC
ATACAGTACGACACTCTCTTTGGAAAACTTGTTGGATGCTTGATTAACAGCTGGATATGTTGATGTAAATAGTCTGTAAGA
AGTCTAAGATAATACTACATATAAGTGTGCATGGTTTCATACCTGAACTCCATTTTGCCACAATCCAACATACTGCTGTCC
CAGTATGAGCCACCTCATCGTGCCCTCTCCATGTCTCTGGTTGTTCTTCCACTCACCAGAGTAAAGGTTACCAGAGGGGTA
GCTGTTAAGACACATATGTCACCTTTAGGCATTTCAAATTGAGGCCTAATCTCAGGCTACAGATTAGCCGTGCACTCCATA
CATACCGTCTCTCTCCCCTTCCCTCTCTGCTGTTCTTCACCCAGTCTCCTTTGTACCAAGATGTCTTATCCTGATTGTAAT
ACACTGTGCCCTGAAACAAAGACAATCAATTTAAAGAAATTTTTTAAAAAAAGACTGTACCTTAATTATGAATTGGTAGAT
CATACCTTTCCGTGCCTCTTGCCCCGATCCCACTGCCCTGTGTAGTATACACCAGTTTTTGCACATTTGTAGGTTCCAGTC
CCATGTTGTATACCATTGTACACTTCACCCTCATAGGAACTGCCATCTGGCCAGGTGTAGGTGCCCTGGCCCATGGGGATG
TTGCACACAAATTCTCCCTGTTAGATAAGAATATACTTCAAGATTTTGTTTGCATTTATTGTTAAACTATTTTATTATATT
CCAATTTTTTTTTTTACCTATTCTATTTGAATATGTATGTATACAGTGGTATGTTTAGAATGGTTTATGTACACATGGTGT
TTTAAAATTTATTAAGCACAAATTCCTTAATAAAATTTAAGTTATTTTCATTATTGTGCCCTACCTCATATTTCATTCCGC
CAGTCTGTGTGAAGACACCGTGTCCATCCATAAATCCCTTGGAAAACATCCCCTTCAGTGAAACAAAGCAGTGACTTAATG
ATATAGTTTTGTTAATTCTTACTGCAAGTGAAATTTGTATTTTTGTCTTGGTAATACACAAGAGTACGATAAATTCATGTA
TTTAAAGGAAAACAATAGAAATACAATTTCAAGCAGTTTTAAAGGTGCGATACGTAAGAATTGGCCACTTGTTGAATTCAT
ACTCACAGCAAATATAGGGCAACATATTAGAGTAACGACTAACTGCTGCCAATTTTAGCTGCCGTTAGCTAGTTAGCTCAG
TTAACCGTGCAGCTAGTGGCCAGGGCTGGAAGCTCAGAACACCAAGGAAGTGTTAGTGTTTACAGGGCTAGCTGGTTAGCA
TGCTACAATACACAGACCTAATAATGTCAATACTGTGATTCCTCCACATTCTGTTGATATTTTTAGTGAAGTCGTCAATGT
TTTGACTGATATTCTTACATATTGCCCCTTTAAATAAATTGAAAAAGAAAATAATATTCTTACCGATTGAAATATTTCTAA
ATGTTTTTTGTTTTTTTTTAAATTGACAATTGTTTGCCTAATGTTCTACAATAGGCTAAAATATACATAAAGTAAGTATTA
ACTGTAGGCTGCTGAAAAATCAAAGATAGTCTTTCCCCCATGGCATCAAAATATGACGTCATTCGCACCGTACCTTGTACA
TATGGCCTCCTTCAAAACAGGCGACTCCTTCTCCATGAAACAGACCTTCACATGTCCCCCCTTCGTATCTGTAAATGCGAC
ACTACAATTTACACTTTCAGCTACAGCCAGTTTAACGTAAGGATGCTCTAAAAATAATTATGTAATGAATGTTATTGGTGT
GTATTTACCTCTGCACAACTAAGCTAAAAAGTGTTGGAAGTTTATAAATGTCTTCACTCTCGGGCTGCTCATCACTGACAG
GACGGCTTTGGTTCTTTACCACATCCGCTGAATGTGCCTTTAAACTGACAGAGATGCTCACTAACTTTCTTTTTTGTTTCC
CCCCCGCTGTCTGTGGTAAATCTGCGTTTCCGCACGTTTGCTCGGTGTCCTGTTTATCGTTTTGTATCGTGTTTTTTTCCT
TTTCTTCCCCCTGTCTCATTCTGCGCTTTATGTAGGCTATCTGTCGTTCAAGCTAGGAATCTCTCCATGGTAACAGTCTCT
GTGGTAACGAACGCTTCCACCTACGGCAACCTTCATCAGTCAAAAGGGCTAATGTACTACTTTTGACACTTTAGGTAAATT
GGGCTGCTAATGCTTCATACTTTAAGGTTTAAAATGCAGGACTTTTACTTGTAGTGGGGTATAATCACAGTGTGGTATTAG
ATACTTCCTCCACCACTGGATTTAACCATACATATGTTGTTTATTACCAGAGGTAGAAGTACTCAGACCTTGTACTTCAGT
AAAAGTAGCAATACAACAGTACAACAGTAAACAAGTATTAGCATCAAAATATACTTAAAGTACCAAAAGTAAAAGTACTCA
TTGTGCATTACCATTTATTGATGCATAAATGTCTCCATCACTTGAATGTTGAATCTGGTTAAAGTGATTCAGCTTCTTTCT
ATTGTTTATCTGCTGGGCAGCTTTTGAAATTCCCCTCAAGTCAATAAGGTTTTCAATCTGTAATATCACATCATAATTTAT
TTTTTGATTATATTTTGTATAATCAAACTATATCTGCAAAATAAGTAGTAACTCAAGTGGTTAAATCAATGTAGAGGAGTA
GAAAGTGCAATATTTCCCTCTGAAATGTAGTGTAGGAGTAATAGGTAGCAGAAAATGGAAGTACTCAGGTAAAATATTTGA
GTAAAGGTACTTTGCCTCTTTCCCCCACTGTTTATGACACCCATTAAAGTGGTATTTTACTGCTGGAAAGATGGTCTTTTC
ATAAAACTGGGCTGTCTCTGCAGTACAAAGATGCAAATATTTTTTCAATTGTTGCTATATGACTAGAAAAACTCACCTTCA
ACAACCACTGTGCTGCACCAGACCACAAAATGCTCCTGCTGGTGGGTGACATGACAGCCCAGGACTACAGATGGAAATCTG
CTACAGACAAAGCATCTTTCCTCTTTTTTGAGATGAAAGTATTTTGCACATGTTCTCTGATATATATTTAGGTGGATGAAA
AAAATGGCAGCCTCGTCTGTCTCGTATTACAGCTAAACAGTTTGATCTGAGTTTTGTGAGCTTCCCCTCACTCAATCCGAC
TGTCAAGACAGGCATACAAAAAACGCAAAAGTAATCTGCAGTTTAACGTTTTCCTTTAATTAGGTGTTAAAATATAAAAAC
TATGATTTTGTGCTACAAAATACAAATAAAATCAGAACATCTATTACCGAGCATGTGAACCATACAAATATACAGTATCTA
CAACTCTGTCTTCTCTCTGCGTCAGAGCATCAGCAAGGTTGATGTTTAACAGAAATAAATGAAAGGAACGAAAGCACATTC
CTGTTATAACAAGCCAGTGTAAAGTAGGATTTTCCAACAGTTCCTTCCTTGACATTGTTAGTCTTGTGAGATGATGTCCAG
GTTGGCGAGGTGTCTCATGGTGGGCCTGCCATGAGGACAGTTCCATGGATGCTCAATCTCCCCCATGTGAACCAGGAGCTT
CTTCATCTCGCTGACACTCAGAGCAGTGCCAATCATCACCTTGAGATGGACATGAGCAAAACAAGAGATCATGTGTCAGGA
ATATCACTATGTTAGATAATAAGTCTATAGCAAAACAGAATAACAACTGCCCTTATTGTAGGTATTTAAGGACCCTCAGTG

TTTTTACACTTTAGATTACTCAAAATCACACCTTACTCTACTCTACAGATGTCTTGCTGGCTCGTGTAAAGGCACAGTTTC
TGAAGATGGGCTGTGCGCAATTCTCTGTGGAGTGTTTTACTTTCACAGTGTTGTACCTAGCCCTGCTTTATAACAAAAAAA
AATATGGAAATCCCACTTTCTACCATATGGGACCTTCAAAGTGTCTGTTTATTTTCATTATAAACTCACAGATTTTCGACA
GGCTCGTGAAGCAAACATCTGCCTGACGCGGGATGGTCGACACATGACCCCTGGGCTGTCGCTCAACATGAAGATCAGCTC
TTCAATGTCAGCTGGGCCAAATGTCCAGTTTTTACTGGTGGGCAGAGACACCAGCTTCACCCTCTCCATCACCTGAGCTGC
AGTCACAAATAAAAAAGTTAGTTTGTGTTTGCAATCATCAAAATAGGGAGTAATTGTTTTGAACTTCATATAAAATTATAT
CATCAAAATAGGGAGTAACTGATATCATCAAATATCATCAAAATAGGGAGTAATTGTTTTGAACTTCATATAAAATTAACT
CACTCAACTAACTAAAATTAAATTACTAATTGCTTGTATTCAACAGTTAAATGTTCGTACCATCCTCATTGACAAGGAACT
CAAAGCCATTCTTTCTGAAGATCTCAATGTTCTCCATGAGTACATTTTCACTGACAGCAGTGAGGTGAAGCTTCTGAGGGC
TGCAAATGTAAAGGGAAAAATAAAAAACAGATCACTTATTAGTAAAAAAGGTCATTCTAAACGACAGAAGGCAGAATTTAT
CAACAGATTTTGATTCTTACGCAATGAGTTTCTGTCCTTGGAGCACAGTGTGCTGCTGCAGCATCTCAAAGTTGTATTTCT
CATCTGTGGCATGCTGGTCGATCATGAAGATGTCCGAGTTGAGTTTGGTGATAATGAAGCCCAGGTTAAACTGACCTATGA
TCTCCATCTGTTTGAACATGTCTTTGCTGTATGGATGAAAGAAAGATAACTTACAGTTGTGGAAATTACACACCAAGATTG
GTAAATTCAGTCAGCAGCATTTACAGGAGCAAAATCCTACCTAATCTCCTTCTTGAGCTCATCCTCTGCACTCTGGTTTTC
TCCGGGGTTGATCTTGGCCCTGAAGCGTCGATAGCGCAGATTCTCCCCGGCCCTCTGTGCCTGCGTCTCCTGTAACCTTTT
CATCTTCCCTGCTAGCTCTTGGAATGAGAACTTGAGCTGCACCGTCCTCCTCTGACGGCAGACAGACGTGTCCACCATCAA
GGACGATTTCTCTGAATGGTTTGAAGAAGAGTTGGATTTGTGCTCTTTAGTGAAATATGGTTTCTCTGTCCTGGCCCTCTT
GGCATCCGGGCTCACAGGACAGTCCTCGTTGGAAGTGATTGGACCAGTCTGTAGCTCTGCTTCTTCAGGAACGGTGTGATT
ATCGTTGGACGTTTCTTCAAATGTTTCATCTTTCACACTGAGTCTGTCAGCTGCTGGCTCAGGAGAACTGGACTCTAGGCC
TGAACACTGAACATCTGGTGTTCCCATGGTAACATCACAACTGGAGTCTGTATCCCTGACAGTCGTTCCGTACCTGAACCC
ATCTAGCACAGAATTCCTCACTGGGGAGCATTTTGCTAGATCTCTTGAGGGTTTCAAAGGAGATCTTACACCACTCAGGGT
GCAGTTAGTAGGTTTGACAGTGTCCTTAAAAAAAGATTGTAGTGTTTTCTGTGTGGGGCCGATGTTGGCAGCTTTTGCAGC
ACTTGACTTGTTCCCAGAAGTGGAGCTGTGATGACTTGCAAAAGCAGCTTTTAGGCCGGCCAGGTTTAAGGACGACTTTGG
TCTCTGGGTGACTGTTTCCTCTCCAGATTCTGGTGTATTCTCATTAGATGGGACAACTTTGCAGATTTCAGATGCTGCTGT
TGTATTTAGAGACAAAAAGCACTCTCAGTATTACTCTGCCATAATATAATAAATAAATATTATGTGTTGCTATAAATGTTA
GAAGTAATGATCTGCACTTACTGGTGCTGGTTAAGGGTGTATAGTTCAGGCTTATCTTATTGACTCCTGCCTCAAACATGT
TGATAAGAGAGGTCTTAAGAATAGCCAGCAAGAGTTTCTCCTCCTGAAGGAAAATCTGTCGTTTGTCTGGGGTGACGTTCA
CATCCACACACTCTGAAATAACACCACAAAAACGCTCATGTAGAACAGAAAGTCTGTGGTTTGTTTGAGGAATAACTTCAG
TTCAGTTATCTGCTTACCTGAGGAAACAGCTATATTCAAGGCAACAAATGGATATTGATGTCTGTTGTACATGTGATACAC
TTCATTCACAAGTTTGGTCACCTGAAAATTGAGGTTAACCAGAAATTAGATGTGTATTAATGTGTTTGTCAACAACTATAA
ATACTCCTATAGAGAAAAGTAAATCACTGTTGTAATAATACAGAAAAAAAGGTCTCAGTTATATGTGCTAACAGCTGCATT
AAAATGTGCTATTACCCATTATTAAATGTTTGTATAAATTACCTTAAGGGGATCACATGGCCGGTTGTTAATGAAAAAGAA
CTGCCTGTCTGTGGCACTTCTCCCAACGCCATGGTCTCCTCGTGACACAAACCCTGTGATGCTGCCAAAACAGAGTTTTTC
ATTTAAAAAAGTACTCTTTTGTTTGCACAGAAGGAATGAACAGGACTTCCATAAAATGGTGACACTCACGAAAAGAGCTGT
TTGGGTAGATCGGCTTCTTTGAGGCCGTATTCTTCAAGAACATTTTCTGTAGCGAACGCTTGCTGAAAAGGCAGAAGACTC
TGCAGCTGCAGAAGACACAACAGACACTGAAATTAGATACTTAGAAAACCGAATAAGAGAAATGTTTTCGTCAACTAAGGA
ATTACACAGTTCCTCTAAGGTTTGGGGAAATTCTTTAACTCTTGGATTTCAACTTAAGAACTTAAGAAAAATGTTTTATTT
AATTCAAGGCAGCATTGTGGAGAATCAGACTGACACTTTCTGCAGATTACAGATAAACAGTATAGTCAAGAACAGTGTGAA
ACCAATGTTCCAACCTGTTTTGGTCCAAATATGGCCCCTATGTTGTCTCTCATACTCTGGCTGCCGCTGGTGCTGAGGACT
GTGCTGCGCTTTCCTTGCCCATTTTGGTTGGAGCAGGTGATACGCACTCCTGTGGAGATTATACAGTAGGACTGCAGGATA
TGTATCATTTTGGCATACTCCTGAAAATACAGAAGAATGAAATATTCAATTGAAGACAATGACACTGAAGAGGTATAAACT
TATGCATTCTATGGTACTAAATGAAAAATGACAATTACAAGGCATTACAAGTCATATTATGTTCTAAGAAAAGAGTTTTAT
TTCAGAATTAGTTTGGATTATTTATGTGATGGTTAAACTCTTGCTGCATTTACATTGGTGCTTTTGTATGCTCTTGCTCAT
TTACTGTGTTAGATTTCAGTTTGAAATCAACTTGCAAAGTTATAGAGGTGTTTTCGGTGGTGTGTTCATGTGCTGATGGGA
AGCTCAGACATTCAGGATTTGATGCGCTCCTACATTAATAAAACAAATAGGTGCACGTATGTAAATGGGAAGGGTCAAACA
TATGATGTGAATTTGGTTGTTTTTTTGCTTTCAGAAGGTTGAGTTCAAACCACCACATGGAAACACAACTCGGAGGACCTT
TTGATTGCAATCCACACTTTCAAATGTATTATAACACAATATTTAAATATTTTTCCAAGTAAGTGTAAAGTCATTGCAAGA
CAATCTTATATAACAAGAATGGAAATCAATGGTTGAGAAGATAGTAGGAAAAACTGTTAAGTAAAAAAAACACATTCATGT
TTGGAAAACTATCTGGAGTTTCAGAAAATGTATGTGAGAATGATCCTTTGATGACTGACCTTCTTAATATTGCGTTGGAAC
TCCTTGTGTCGAACAGGCAGGGTGTAGAAGAGCTGCTGCAGACTGACTGTGGTGCCTTGCTGCCGGGGATGAGGTGACTGC
TGCACAAGGTGGCCTTTTTGGTCAAACACCAGCTTGGTCCCCACCTGGCTCAACTCGTGGCATGTCACCACGGATAGGTTA
CTGGAACACGGGGGGATAAAGGGGAATTTTAGTCGATGAGATAACAGTTAATATAGGTTTACGTGCTGCGTTAATGTGGCT
GTTACCTCAGAGCGCATAAAGAGCTGAGGGCTTCACCTCTGAAGCCAAATGTTTCCACATGGATGAGATCAGAGAAGTCCC
TCAGCTTTGACGTGTGATGCTTCAATGCTGTGGAACGGTCAGAAGAAGTTTCACTTGACCAAAAAGATCAGCTAGACTTTG
ACTATCTGGGTGAAGATAAACAAAACTGACACTTACTCAATCCTTCAAAATTGGCTTCTTCTACTCCTTTGCCATTGTCTG

ACACTTCCACCAGTTCAGCTCCACACTCCTTTAGCCTCACATCTGTAACATGTAACAATGTGGTTCAGAACTGTAGCACTC
ATGAGCAATTGGTGGGCTATCAAAACCTCATTAGCAAACAGAAGACCCTAGTATCACACTGTGTGCATCAACACTTACCAA
TGCTGGTGGCCCCTGCATCTACGCTGTTTTCCACCAGCTCTTTGACAGCGGTGGACAAAGTCAGCACCACCTGTCCAGAGC
AGATTTGATGCACTGAGTGCTTATCGATGGCCTTGATGGCTTTGGCAGGTTCAGAGCAGCTGAGAAGAAAAGCATTATTCA
GTCACAATGATGTATAACTTTATCTTCTGTTGAATTCATATTTGAGCAACAGATTTAAACATGTACAGCTATTTAGAAGAG
ATTAAAAGCTCCTTAATGGTGCAATACAGTATGTATGTACTACATGTAAGATTTAAGATTGAAACCCCTCAAAAAAAGAAC
TAAAATAATCCACAGAATGTGAAGAAATCACAGTTTTGATATTACGACATCTATGTATTAGGACGTAGAGATATCTACTGA
AGTTATCATGCTAATCAGCTAGCCCTGCCCTGTCCTGGACCAGCAGTATAAACATCAACACTTCCCCTGTGCTCCAAGCTC
CCAGTCTGGACCACTAGCTGCACGGCTAACTGAGCTAACTAGCTAATGACAGCTAGAGTTAGCAGCAGTTTATGGTTACTC
TGATGATGTGCTGCTCCCTATTTGTTTTCAGTATTAAAGCTGCTGTAAGTGTTGCTGTCGTTTTAGCAAACTTCCTGATTA
CTGTTGGAATATAGAGCTATTTAACACTTTATTTTAATACAAATAGATCACTTACAGCAGCTTTAATTCCACAAGTGGCAG
ATTCTTACATATTGCAGCTTTAAACCCTGTGTTCTCTGATCTGTGATAAATACATGAGGATTGTGGGAATAATAATAAAAT
GTGATGTCTCACTTAAATGTATATTGCTTGTGGAGTTGATTTTAATCTTATTGTACTATATTGTAGAGTGGCTCCATTATT
TTAGTAAGAACATGTGTTTTAGGTGTAAATAAGAATATACACGAGCTTTTCTAACGGTGTGGGTTCCAAATGTAACACACG
TCTACACTGTAGATTTGCATATTGTAAAAACAAATACATCTGCAGTCAAGATACCGAAATAATTACTCTGTTAGCTTACTT
ACCAAGCATCAGACATCTTCAAAGACTGAAGAGCTTCACGAGGTTTTTTACATGCGACTCTATCTAACACGGTGGAAGGAA
GAGAAACCCGCTAGTTAGTAGTCAACATTGTCAAATACAGCAAGTTTGTTTTGAATTGTAAAATTTCAGTGAACACGCTTC
ACGGCATCATGTTGTAAACACAGAAGTCGAGCTGTACCGCGCGACTGCGCAGATCGTTCGCGAGCGTCAACTTTGCGCAAA
ACTGCGCAGTTTGTCTCTTAAAAGTAATAATATAAAATGAAAATGTGTGGAAGAAGCAAATTCACAAAACAGACACAAATA
TAAGAGTGGCGACAGCATCAAACAACTGACACACGTGCGTCAGGGTTATAAAAACAGTTGCCCAAAACTTTGTAGCATTTC
ATCACCGAACCTACCGGAAACGTGACGTCAGGGCGCGTGTACAAATACCTCATATTATAAAAAAACGAATGAATAGCTTTT
TCGTGCAGGGTCCTTCTCAGTGGTTTGGAGCTTTACAATTATATTTATGTTAGAACATAATAGTTTTGTTGCTTTCATACT
ATCATTTTTAGCATAACTTTTGTTTAGTCAGTTAGTCTTGTTCTTGGTCGAGCACGTATATTGTCAGACGGTACCTGACTA
TTTCTAATGTGGAAACATGGCCGAACATATGGGACTTGTGAGAGAAACCTTCCACCGTCTCTTCTGACATATTTATGCGAT
GTAAACACTTGTTAAGCCAGAATTCAGTGAGTAGTTTTGACGTATCTCACTGCTGACATGGGATAGCTGCGGTAAATTGAG
CAGCATTTTAAAATACGCACTTTTTTTGACGAGTTATTATTCTTCATGTACAATAATGTACAGTTCGACATCCAACCACGG
ACTCCTGCGGTCGGAGGAATGTTCTGGTAGCAGCAGTAATATTTTGGTCCACCACAACATTAAACCCCTCAGGACACATGA
GGGTGAGAGTAACGGACTGATGAGTTGGGCAAGCGAGGAGGACGATGAAGTCCAGTTTGACAGTAAGCAGCTTTACTTAAG
GTTTATACCTAACTGAACAGTGTTTACTGGTCCTTATCGACCTGACTTTGGTGGTGGTGGTGGTGGTTCACCCATATT
ACCTGACCCAATGATACCAGAATAAAACGTAGATCTGAGTGGTTCGAGACTTTCTTGCTTGTGTAAATAGCCAGGGGCGTA
GTTGAGGGATTAAAGGCAATGTCAACACATGACTCTCTGGGCCCCCCTTCTTAACTGTGGTACAGTGCCCACCCTACCTTT
ACTCTAGATATCATAATATTTTAAAGAATGATGAGTAAAGCTGCTGTATCCTGTACTTTTAGAAATGCAATGAAAAGTTAT
GACTTCAGTATGTACCCATGTCCATTTTGTTAAATGGCATAATAATAAAACATTACGCATTTTTATGTGGTTGATAAAAAC
GGAGTGCAAGAGTTCATTTCTGTCCACACCTGTAGGACATAACATTTTGCTTAACGTAGGGCTGGGCGGTATGTCCTCAAA
ATAATATTGCAATATTTTTAGGCCATATTACAATATACAATATATACCACTATATGTTAGAATTTATCAAAAGCATTTTAT
AAATGTCCGGACTGGGCGACAAAATCAAATATCAGAATATTTTTAACCAAAAAGGCCCTTCTTTGATATAGATTTGATATA
GATACAGCAGTATTGTAGGGAAGACTATTGATACATTCACAAAATACCAACACAATGAGATTGCTGATCAATAATCATCAT
AATGTGGACATAATGACTAAGTGGGTAAAGGCAAGTGTTAGAACGAGTAGAAGAGTCTGGTAAGTTCAGAAAATGACATCA
CTTTACTGTAATGGTGCCTTTAAAACCAGGATAAGACAACACTTATGCCATATAAAGATGTAATGATGTCCAAAATCTAAG
ACGATATATAGTCTCATGTCACGATAACGAAATTTATATTGTATATAGTGCCCAGCTCTACTTAACATATCAAACTCATAA
CACAGTTTATAGCTGTTATAGCTTTAACCCTGTCAGTACCAGTGTAGTACTTGTTATTACACTTTTTGAGAAAAAATGCTT
TCCATTCACACAAGTTTTAGTCTCTTCCTCAGCCTAGGCCAGCCTGTCATCCTATACAAGCCATGGTAGATTCCCTTTTTT
GCATAATATAAGCCTCTGTATGATGTAAGGTTCAAGACTGATTCTTAGAAGTAGAAATAACCACTCAAATAAATATACTTC
AGTCTCAGTCCTGGTCTCTATGAAGAGAAAGTATGCCAAAGCTGTTAAAGGCCTCTAAAGTTGTCTTACTTTATAATGCAA
TTACTAGTAGATTTTTCCCAGAATTGTTGCAGTCTAATGTTATTAACAACTAGACAGTCTGACATGTTTTTTCCATTTCTA
TAAAGACATATATATCACATTGCGGTATAAATCAATATTAGCAGAAGGTCTTACAGAGATATTTTGCTCATTATCTTTATA
CAGCTTAGCTCTAATCAACAGGCTTAAAGGGGTACTCCAGCAATTTGATACTGCACCTTCAGAAAGTAAGAGGCCTTGCGC
TGGACAGGCTGAAAATAGAATGGTTAAAATCGAAGCGACAGGTGCATGAATATCCAGACTTTTTGTTTATTAGTATGAGTT
TAGGTTCAAAATAGGTGGATCTTTTTTTCTCTTCATTGCCCATAATGCACCTTAACAGTGTTCTTTCATTAGACCCCCCCG
CCTGTTAAATTTAACCATCTTTCAAACTACATGCCTCCAGTTTATAACACAGTTTTTTTATTGAACGTTTGAAGTCTCAAG
CCAGATCTAGGATTCCCCTGATGACATCATCAGGGTTGTTTTCACAGTCTGAGAAGTAGCCTCATGGCCCTTTACTCGTAA
CCTTACATAAACTTGCATTTAACGGTAAGTTGGCATGGTCTAGTGACATCATCAGGATGTTACATCAAGTCGGATCCTAGT
TTAGCAATACATTTCTACTTCTCGTTTTGCCCTATGTTGTCATTTTCATTTTGTTTTAATCAGTGTAGTTGAAAAAATGTT
CAGTACAGATCAATTGCTCTCTGCCTGTATTTTCACCTATACATTTTTTTTTACATAAAGAAAAACAAGTCAGGACTTGCC
CCTCCACACTTCCTGCTGTACAGTATGGTGAAATGAGAGTAGATGAAACAGCCCCTCCTTTCCATCCTGGCACAGTGACCA

AGTGTCTCAGCTTGATCATGTGTTATTGGAGTACAGTTAGGTGGAGGAGGCTGGGGTCCACTGGAACTGGCAGATAAGCCT
TACTGCTGTGCACGCCCCCTCATGTGTAAACAAGATGAGCTAATTTTTTGCCTTGGATGTGAGCAGGATAATTATTAGTAG
AGCTCTGAGTGCTTAATGTCGGACCAAAAGTGTTTTTATACAGAAAAATGGACCTAGGAGACAAAAACCTTGTTGAATTGA
TAGCAAAGGCTATTGAAGAAGGTAAGACTAAAGAGAAGAAATTTTGTGGTTATATGTGTATTTGATCTTAGCTGACAAAGT
CAAATATAATGTAAACTGTTGTTTTGTGCCACAGTTTGAGCCTTTTAAGAGGATTTTTCATTGTAAAAACAATTTCATCAC
TTCCTCTTTCATTTTTCTATTCCATTTTGTGTGCTCAGCTTCAGACACAGATGATAACCATGAGGTGTTGATGGCTGGCAG
GCATTACCCGTCCATCCAGGAGTTTGCCTCAGCAATCCCCAACCACCTTCTGCTGGGCTCTCTGCTGGAGCACCTGTGCTT
CGTCTACGAGAGCAATCCAGCACGCTCACGTATGCTGTTTAAAGGTATGTACTGAGATGAAGTGATAGCTAGTTTAAAGAA
TAATACATTATACATACGATTTGTCATTAATTCCTAATTCAGTTATGAATTGTTTTGTTGTAACTCTGACAGTCATTGGCC
AGCGTTTAGCCGCCATGAACCTCCTCTCACCTTTGGCCGTAAGTGATGAGTTCAGCACCGTCAGACTGCAGCACAACCGGG
CCTTCACTGAGCTGCTTCATGCTGCCAGCTCCTCGCTGTATCCACAGGTAACAAAATCATCAGTATGTCTTTGTAGTAGTC
AGTATGTTGTCAGTTTTAATGTTGGGACTGACTTAATTTTAACTAATTATGTAAAATGAGTACTTGTACTTAGTGTAATAG
ACATAGTCAGTGAGAGAAGCAGCAAACTCAGCTTGCAGTTTTGTTAATTGGGAAGTGCTGTAGCCACTAACTAACTGGTAC
TTGTTTGTCCTCTTTTCATTTCACAGGGGCAACAACTTTTCAGTACAAACGCACACAATGCAGCAGTAAGGTAGGCTGCAC
TTTGAGTAGTTGACTTGCTTGAAAGGAATTTTTATTTAATCTTTATTACATTTATTTTTAGTTGCGATGATAGGTCGCTCT
AGCAGACACCCGGTCGATCAACCAGTCGTGCCACTTCCAATTTCCCCACCTTTTTTTTGTTCTTGTTTGTAATTATTATGG
TACGTGGGTCAAGTGTGTGTGTGTATGTGTGTGTGTGTATGAGAGAGAGAAGGCGTGTGTTTGTGGTCATGTCAACTGG
CTAAAAGGGAGTGTGGCAGTGGGAGCGGCCTACAACAAAATACGCATAATTTTGGATTAACAAAGTTATTAAGTTATGTGA
GTAATGGATTCTGGTAACATGACCAAACTTTGTAGAAACATCCCATCTTGCCATGTAATGAGGTTGTGTACATTTAAATGC
ATGTACGTATGGATGCCCTTGCGTACCTGGTTGCAGCTGTTGTGAACTCACATGTTTTATTAATATAACACAAGGTCAAAG
AAGTGAATACACTTACACACTTATAGAAAGGAACAAACAACACAAAAAAATACCACCTGCTGAAAACAACAGCACATTATA
AAAGAGAAGTATTTAATAAGAGATGTTTGTTTTTGTCTTCCCACAGACCGAAGGAGGGCCTGTTTCAAGCGCAGACGTCTC
GGTATCTTAGTGAATTTGAAGAAATGTGTAGACTTGGAAAAGGATCATATGGAAATGTCTGGAAGGTATTTTACTCGGTTC
TACTCCATACTTTTTAACAAACTCCTCCAAAGTAGAACATGAGTTGAGTTGATCAACACAATCATATTATGTGTGACTTTA
TCTTGTAGGTTATGAACAAACTGGATGGACAGTGCTACGCTGTGAAGAAAATACTCATCAAAAAAGTCTCAAAGGATGACT
GCATGAAGGTGGGCCATCAGTGTTTTGTCAATAGAGGCTTTATTTTTACAACCAAATACAGACAGGTGATAGACGGATAAT
TGTATGATCTGGTTTTGCTTTAGGTCCTCAGGGAAGTCAAAGTGTTGTCCAGCCTGCAGCATGTAAATGTTGTGGGCTATC
ACACTGCCTGGATGGAACATGTTCAGCCTGCGGCACGTGAGTGTTTAGCTATTTAGCTACGTTTGTGAGTGAAGTTACTTT
TCAAGGACTTTAGAAGCTTTTTGTTAAAAACATTTCTGTTGCAGAGCCTGAGTCTCTCCTGCCTGCACTGAGATCAGCTGG
AGATCAAGAAAGGTAGGATCTCACACTAACTGTTCTTACATTCATTTATATTGATATTTTTCACCAGTAAATTAATTGAAA
TGTAACTATTTTTCAGGAAAATAATCAGTGTATTAATCAATAATGGAAATAGTCACAAGTTGCCCTAGTATCAATTATTAT
ATGTCCAGAGCATATGAAACTGTATATGGAAACTGTATATGCCGAAAATATGAGCACACGTGTTGTTGTATGGAAAGTATG
TGAATTAAAACAGTGGCGATGCTGGGGTTAAGCGCTAGTTATATAAAATCGATTCAGGAATTGTGAAAAAATAATCCTGTT
AATTAAAGGAATAGTTCCTAGTAATAGAGGGTGAGTCGATAATGACTGAATTTCGTTCTAGAGTGAACTATTCCTTTAAGG
GAATCAATATTTTCCCCCACACCTGTTATTTATAAGGTTTATTGATATCAGAATCAGAAATACTTTATTATCCCGAGCGGA
AATTGTTTACACTACAGCTGCTCCCATTCAAGTTCAGAATATGCAGAAATAATAATAATAGTAATAGTAATAATAATAATA
ATAATAATGATAATAATAATAACAATATCTATATATAACAAAAACAAAACAAAAATACAAGAAATGTACACTCCACTAGAA
TAAATATACACATATACATATACAATAAATAATGCTTACTTATGATACTTGTAATTACTGTGTATGTTGGATGAATTACAG
ACTTGATTAACTGCACATTTTTATTGTTGTCGTCAAGTACTTTTTTATTAACACTGTGTTGCAGGTCAAAAAATGAAAAAA
TATTCCAATCACTATTTATACCAGTGTATTCATCTCATGTAGATGCAATTATAGTTTATACAGTAAGTAAATCATGACTCT
TTCATTCACAGTTCTGATGAGAGCCCTGACAGCGGCAGCACCAGCTCCATTGTTTTTGAAAGCCTCAGTCAAGCACCGACA
GATGCAGCCCCAAGTGCCAACGCACACCCAAGAGAGACCCAGTCTGTCAAAGCTTTAGTTCCAACCCAAGAGAATGGCCAA
GTGGTGTGTCCCAAGACGATGCGGCGCATCCCTAAGAACTACGTCCCCTGTGTGTTCCTGGGACAACGAGATCCCATGAAG
GGCACCAAATGTCCTGCCACAGGCTGGGACAGTTCAGCACTTCTAGAGGAGGAGTCTAGCAGGAGTAGCATCGAACTGAAC
AACAACTTCTGCATCGACAAGGAATGTCAGCAGTGGGCTGACGTTCCCACAACAAAGCCTCCCAAAGAGGTTTGTGCTAAA
AGAGACTTTCAGTGATCAAACAAGAATTTGAAACATAAAGATTACCATGTTCTATAGCTTACTGAAAATGAAAGATTTGTT
TTCAGAATTATTTTTAAAAATGTTACAGTTTTTAGTTATAGTTTGGTTGTATCTGCAGGTCCAGTTCCACCTGATGCTCTA
CATCCAGATGCAGCTGTGTGAGCGCTCTCTGAAGGACTGGATCTCTGACAGGAACAACAAGCCCAAAGAAGAACTGACCTC
AAAATGTAATTGTTAAAAACATGCATTGATTTAAATGATTACAGATTAGAGAATCACAGATTGTTTAAAATGATTATGTGT
CAACTTTTGTTCTGAGTTAAACTAAAATACAATTTTGGAAAATAGATTAAACGTTTTTTTATTTTCAGGTCCGTATGGATG
TGTTGATACTGAACACACACTGAGCCTGCTGAGAAGGATACTTGAAGGCGTCGAGTACATTCACTCCAGGGGAATCATGCA
CAGAGACCTGAAGGTAAGCTACTGTGATTTGTTTTATTTAAACAGAAGCAGCTTATGTGCAGTTTGCATCGACACACAGGT
GTTAGAGAGGAGCCATGTCAGTAAGGCTATTGAATGAGTCCTGGTCACCATCACTGTCGGTCTCAAAGGAAATGCGTGAAT
CCTCCCAGTAGTTGAATACAAAGTTATAGACACTCTCAGGCAGGACTTTTCTTAATTCATCTCTCTTGTCTTGGACGTATC
TTAGATAAATTGCTCTCTTGCAAATATCCTGAAGGCTCCTTGGCTGCTGAATGAGTTTTAGTAGCTGCTGTGTCTGTTTGA

AGAAACATGGTAGATTCAAGGTGGAGGGCAGGAGGCCTTTGTGGTTGTAGACAGTAAGCGGCGAGATTAGACTGAAGAGTT
TCACCAGCAGAGACTTTTTCCTCACATTAGCCCTGCGGTTCAGGAACAGAAACAGACAGTTCTCTCCTGTTTTGCTCAGCT
CGTTGATGTTGGCTCCATACCTGAGCAGCAGGTCCACCAGCTCATTGTCGCCTATGCTGCACGCCTCCTGTAAAGGTGTCA
TTCCTGCCTTGTTTGTAGCGTCGACCTGGGCTCCGTGTTTGACCAGCTCTGAGATGCAGCCACTGAAGTCCACTGGGGTTT
TAGCAGTCTTCATAGCCGTTGCCACAGCAGCTATGTGCAGAGGGGTGTTCCCAGAGTTCTTCACACCCTTGAAAATAGTCA
AGAATTTACTTGAATTTAATACAGGACATATGTAGAACCTAATGCACATATTTCAACAGAATAAAGAGCACTACAAGGATT
GTAATGTGAGGGCAAAGTTTGGTGTCACTTGAATTTCTATGCACAGAAACGTTTGCATGTGAATCAAAAGATCTTTTGTGT
TGTCTAGCACATTTACTTGTGATTAAGATATCATCAGGATCACTAATCAACAGTTATGAATCATTAATTATTTTTATTAAG
AATCTATGATTGTTTAAAAAAAACATTTAAGTACTTAATACAGAGACTGACTACATAAAATAATTCCAAATATATCTTGAA
AACTCATCTCTGATGCAAAATTCACAGGAAATAATCTGCTCGAAATTCATTCAAATGGCTCGTTATACATAAACGTCTGGC
AGTTATTGCAATTCACTATTTGGGATGATTGGACAGTTTATCATTTGTTCTGCACTGTTGTTATTATTATTCATTGAATAT
GATGTGAAATACCCATAAAACAGGTCAGTTTACAGAATGATAGAAAAGTGTTCCGTTAAGGGAAGAGGTTTATAAAGGGTA
TTTTCAGCATTTTTTTTTATATATATTGACATTTCATAGACTGAACGATTAATCGATGAATTGGCAGATTAATTGATGAT
AAAAATAAGCATAGGTTGCAGCTTTAGACATTTTGACTCACCATAACAGGAAAAACAGAGGTGTTAACTAAACTACAAAGA
TATATGATGGCTCGTGTCTGTTCAGGTGTCCCAGTAAGGCATGGAATGTAGCCAGTTGGAATGTACCTTTAGTTAATGACA
GCTGTGCTTCACCTGTTTTGACAGGTCAATATGACTGCTGTGAAAGAGTCTTCAGTATTTGTCTTTTTTGTTTTATTGCGA
GTCTTTAAGCAAGAAGAAATAGATGATAACAGAAGAACATCACAAACCATGTTGATATCTGCTCCCTCTTTAATCAAGCTG
GCTATAATGTCCTTACGAAGGATCCCAGCGGCCATATGCAGGGGCGTCATCCCACTGCCGCCCACTGCGTTAATATCAGCA
CCGTAGCTGGCCAGAGTATGGATGGCAGACAGAGCTGTGTAGTGCACTGCAAGGTGGAGAGCTGTCTGGCGAATCTTGCCA
TCCACCTAGGGAGGAACCACAAGACACAGTCGATTTTACATTAAACACTATACTCACAAAAGCAAAGAGGCTAAACATAGA
TTTTGCTATATGAAAAAGTTTTACCTTTGCATTGATGTTAACACCGTGCTCACAGAGGAGCCGTAGACAGGCTTCTGCCTG
TGTAAACCTGCTGGTAACCACAGTGCAGAGCTTGGAGCCTGGTTTTGGCCAAGTAGTCTGGAAACTAGGCCAACTGGTTAT
CACCAAGTGTAGAGTGGTTCGACCAAGCCCGTCCCTGTGACAGACGCAGGGGAAGTGAAACTGTTGCAGATATACCAAAAA
TGAAATTAGACACTTGCTGCATACACAATCTAACTTAACTATCCCACTGAAATGAGGGGCTCCTCCTGAGTTGTTGTAGTT
GTTCATTCTGATATGAGAGGCTTGTTTAACACTGTAGTATTGATAGGCGGGATGTTTGGTGTTTAGTGTGTGTACAGGATG
TGTTTGTCCAGACCTCATCTCAGGGTCTGCCCCTGCTGACAGCAAGATCCGCATGCTGTTTACTCTTCTGTAAGAGGCAGC
GAGGTGAAGAGGACGGATAGCACGACCCTAAGGCAAGACATGACATGACATGACAAGACAAGACAAACTAGACTAACCCTT
CAAAAAGTACTGTCTAGTTTGCCTTCTATTTTAATAAATCAATGATCACATTAGAGCAAAGACACCTGACAGTTCCGAGTA
ATTATTGAATTTTCTTGTGGTTATCATTAACTTACATGTATCATGTTCCAATTCTTACAGGTGATTTGTAAAAGAAAGAAA
AAGGAAATACCAAGAAAATGGCTGCATTGTTGCGTTTACCTTACAGAAAACCTCTGAGGGGGTGCCGTCTTGTATCTTGAG
GAGATAGTTGCTGTCATACCTTTTTGTCATTTCTTCCATACGTTCCAAGTCTTCATCCCGAATAGCGTTATAGAATTCGTT
GCCCTGAAACTTAGAAATGTTACTGTTCCGGTCCTGATGTTCCTCTCTCTCCTCCGACATGACTAGGCACTGAACTTCACT
GCTGTCTGTATTTGGTTTTGCTCATGGTGAGCAGTGCTTAAGAGCTGGTTATTCAGGTTTGCACATGCAAGTCTGTATTTC
TTCGAGGTGTGTCAGTTCATTTTTGGCTGTGGAAGAAAATTAAGGACACTACTTCTGTTTTCTGTGTTTGGTAGTTTCAAC
AGCTACTTCTGTTTTTTGGGTTTGGGGTTTTTCGTCAGTCTGATTATATCATGTAGGTTTGAGTGTTGCACGTCTAAGTTT
GATGTGTGGGCTTCCTGTTACAACTGATAACTTCGTGCTGTGTGTGGGGCCAATTATGGGGACTGGTGCGTCCCTTCAGAG
TAAATTAGAGGTTTTCCAGTGCTTGTGTGACTTCAAAGAATGCACTTTGCATAAGCTCACATGCTTTGGAAAATACATGAG
ATACCTGAAATGCTTAAAGTCTTGAAATCTGCCCTGAAAATGTTGTTTGAATACTATGATCTCATATGCAAAAACATCTGC
ATCTTTACACCCAGTCACATATAAAACTAATTGTGATATTTTTGCATTTTCCCACAGCCAAGGAACATTTTCCTCCACGGT
CATGACTGCCATGTTCGGATTGGGGACTTTGGTTTGGCCTGCAGGGATCTATTAATGGATGGCCATAAAAGCACCACCTCT
CCCAGCACTGGTAAATACTGCGCACCTCTTATCTTGTTGATTTCAAGTCACAGTCCCTCACAGAATCACCACCAGATGGGG
CCATTTGCTTTAAAGTCACATTTCTGGTGTGTTTTTGCAGATTCCTCACATACCACAGGTGTTGGCACATTTGTATATGCT
GCACCAGAACAACTGAAGGGCTCCCATTATGATTCAAAGGTAAACAAATTAATAATATTCAGACAACTTCAGACTTAATTT
TTCATAGAACGCAACATATTTTCTTGCTACACTTTCCATTATTAATATTCATCATTATCATCTCTTCTATGACTTTTTGAA
CAACTGTGAGTCTAAAATGTATTTGTGTTTTTCCCCTCCCAGTCAGACATGTACAGCATCGGGGTGCTCGCTCTCGAGCTA
TTCCAGCCCTTTGGGACAGAGATGGAGCGGGTCCGGACCCTTGGGAACCTGAGAGAGGGGAAAATCCCAGACTCGTTCTGC
CAAAGATGGCCAGTCCTGACTAAATACATCATGAAACTGACGAGTAAAGAGCCAAGTGTTCGGCCCCACAGCCAGCCAGCTT
CTACAGAGTGACCTCTTCTGCAGTAAAGACATTGTATGTAGTACATGGATAGATCGTAGCTCTATAAATAAATTACCCCTT
CATGACTTTCTGTGCCATTAAAGGTAAGAGTATTTTGGGATAACTGATCTATGTAAGTATGTCACAATTATTTTTCCCCCC
CAGGTGATCCATGGCTTGCAGAAAAAGGGTTGAACAGCAGGAGGAAGAGATCATGCAGCTGAAGAGACAGATCAGCCAGCTT
CAGAGCTCACAAGTCACAGTTCACATCTCTGAGCCGGACAAAGCCTGACCCTTTAATCTCAACTCTTCACAGACTGTCAAT
ACTTGATTCATTCTACCACTGCTGTACTGTGTTTTGCTTACTTGGTACAAGTGGTAGAGTGTGCTTGTGTGCTTGTGTGCG
TGCATGTGTAATCATTTGAGTGTGTCTTGTGCACCTGGATGTAAGGGTGCTATTATTTATCTTCATTCAGCTCAATAAAAC
CCATTTCTGATATGCCTTGCCAGATTTACAAACAGCAACATGTGATTATTGATCATTTAAGATGTTTGACACTTAAAACAC
ATTTTTACTCTGATTTTTGTCTAGCTTTGTGAGCAGCCCATTGTCAGCAAATTGTCAATCAATTGTGGACACAATTAGCCT

TCCAGTTTTGATTGTATTGGGACATGGGCACATATATTTATGTGCACAGATGTACAAATACCCTGCACATGTGGTTGATGA
TGTTATATGCGGTTTAGTTATTGATCACAGGGGATGCTCCCTCATGTTTTAAGGACTTTAAATTGTGGAGCTACTGTGTGC
ACTCGAAAATGTCTCTAGAAATGGGCATTACAGCATAGATATGGCAGTCTGACTGTCTGTCTATAGCCAAATAAATATGCT
GGTATCGTTTCTAGCATAGATTTCTGGGTATATAGCACACATTTTTATTGTACATCATGTACACAAAGGTCAGAGGTGTGA
ACATGCGACGGATCTTCCCGAAACACGTTCCTCACAGAGAAACGGTCGAATGAGCCAATCAGAGACCTCGTTTCATCGCAG
GTACCCGGATGTACCCGTGGGGCAATTAGCTACGTCATTGTTCACCGCCGGACACCCGCTGGAAATAAGGAATGGATTTAC
AACCGCTATTAGTGTATTCATAACAGATTTCAGGTGAGTGTGTTGTTGCTATTATGATTAAGTGGCACTTTAACTGTGCAG
CTGTGATATTTTGGTAGGCCTGAGCCGCTTAGATTTGTTGTAGTAGCCTTACACCGGTTCTCTGTCATGTCGGAGACAGCG
GCGCAGTGAGCACACTCAGCCGCCGGTGTCGAGCCCCTCCTGTCTCAGCTAGCTAGACGTCCCGTTACTGGGGCCCACGCT
TGTAAATCAAATCTACTTCGAAGTAGAAACAACAGAGTGCCCTTCGACATCGATAGTACGTGGTTATGTTACTCCCCGCCA
TGTCACTCAGCCTCCATCGTGCCGAGCTAGCTAGCACCGTTAGCATCAATATTGCCGCACGAGCGCCGCCGCGCGTAATGT
TAGCCCCGCTCGGTAGCTATATGTGTTACGTTCGCCGTTTCCATGGTGTCGCTGCCTTCACGAACCGCTCTCTCATCATCA
GTCTTTCCGTCCCAGTCTTCGACAAGAACGGTGAGCTTTTAATCGGTGCTACGTCTCCCACAAGTGCTAAAGGAGGGTTTA
AATCGTGTTTTAATGAGGTTTAGCATCGCTGCGTGTTAAGGGCCTAGCTGCCGTCTGTTAGCCAACGTGAGGCAGTGTGCC
GGAGCTGGTGGGAGGGAACAGCTGTTGTTGTAAACGTGCTTCCGACCAGGAAATTTGCTCTGTCGGGGTTTTGAACTAGTA
ACGCTCACCTGGGAACTTCAGCACTGCGAAATCATTTCCATGTTTGCTCTCAGCAGTAAGTCGATTTGAACATGTTCAAAG
TAGAATTTAATACCTTGCCAGTGTTTTCTGATGTTTGCCCAGTAAGTAAACCGTGCACGGTGCCCACAAATGGCTATCACT
TATCGTAGAGCTATATTGACTTAGTTTTTAGATCCAAGCGGAGGTTGAATGACAAATGTGATAGTGAAGTCAGTCAGCCGA
GACTTTTCTCCCACGGCAAGCAATTGTGACCTCTTGTTTAACCATGCGATATTATGACTGAGTTTGGATACTTTGTTACAT
AAATAATCAGCTATTCAGTTTATGGCGCGTTGGTGTACTCTTGCATTGTGTTTCTCAAAATAAAATAAATTGATCTTACAT
AACATTCTTGACTGTTTACTAAATGTCTCGGTACTGTATAAGCGCGCGAAATTATCGAAACGAAACACTGCAGTGTTTTTA
TCTGCACTTTGCAAGTGATCATTTGTGAAAAATAGTGATTATATGGTACTTAGTGGTTGTAATCAAAATGTAAGTAGTTTG
TGTTAACTGGAACAATCAAAGAAGTTAAGAAAATTGTATCACTATATTTGATGACAGCCTCTCAATACCACAACATTTTGG
TTGTGCCACACACACAGATTCCCCTCCATAAAAGCAACAACAAAGAGGGAAAATGCAAAACTTTTGATCTGTCTTTTCTGC
AGCTCATAAAGTTGTATACTTCTGTGTTTCTGCCCCTCCTACAGCAATGAACAATGACCATAGTTGACAGATCTTCAGAAA
AGTCTGACCACGAGTCAGTCGGGTGTAAGCGATCTCCCTTCACCAGTGGAGACGTGGGCATGGACAGCAGCTGTTCCAGCA
GCTGGGCAGTGGGTCCCACCACCATGCCCAGTGACTCTCCGAGGCTGAAGGCTCCAGGAGGCTGCCTGGGCCCGACACCTG
GAGCTGTAGTCTACAACAGTACGCCCTCCTCTGTTGATCGGCCCAAGGAGCAAGGTAGGCGCAAGGACTCTTATCAGCTGT
GGTTTTGTGATGCATGTGTCCTGAAAGCTGAGAGGAAACAACTGAATGTTGGCAGGTTACACTTACTCCATTGTGTACATA
CCCCTCAGCCAAGCTATACTGTGACTCTATTTAACCATTAAACAATAGACTGATGGAAGAGTTTCCTGACTTATGTGGTCA
AGCTGTACAATTGAGGAAGCATTTGAAGATGCATGCCAATAGTCAATATTTTCCCACCGATATTGGAAATGGTGATGTACA
CTTCCTCAGGTCTCATTACAAATGCCAGTTCAAAGCAGACATGGATATCTTAAGATGATAAAGTAGTTTAATAAAAAAGAC
CATGTTGCTAATTTCCATTTTGTGTTGCTGAATTCTGTAATTGTGTGTCATAATCTTGCCAGCAATCTGAGAACTGTTCCC
TGGAAACTTTATACATAAATAATACAAAGCTGCACCAATGAAGCTAAACCTATGGCCAGTGTTTCTGTACGAGAGTGTGAC
ACAACTGGTTTTGTGGGTGTTGGGTTGCTGCTTCCACTCTTCTTTCATTCATCCAATTACATAACCAAGCCAGTTTTGAAT
TGAGAGCATGATCAATCACTTGGGCCTGTGACGCAATTGTTGCTTTGACATAAAAAGCAGCAGCCTGACTTAGACAGAAAC
AGAAGAGAAAGCAAACTCTGTTTCCTTCAGCTGCTTGACAGCCTTATTCAGTAGCCAGAACCCCTCCCTATAGCTGTTTGG
ACTGATAGCTATGGCTGCAATGTCCTGGTGTTAGTTTATTATGTGCCAGTAATATTTATTTTATATAATGCTTTTCAGGGT
ACACAAAGACACTTTACAGGTTAAAAAGCAGAATAATAAAACAACAACACACTAAAAATACAGTAATCAACAAAGTACATA
CTTGCATAGACAATGTAGCATTTATACAGATACTTATTGGTGTCCTTGACCTGGAAAATGCATTGAATCTAATTTTAGTAA
TGATGTTGTCTCTGTGTGTCCAGTGATAAGCAGTGATGACGGCATTGACTTGCCCCAGAAGGTCCTCTTCTCTCCAGAGCG
GCTCAACCTGAAGTGGACCCAGGTTCACCGCATCGGTGCAGGCCTGCAGAACATGGGGAACACCTGCTTTCTCAACTCAGC
CCTGCAGTGTCTCACCTACACCCCTCCCTTTGCCAACTACATGCTGACACGGGAGCACTCCAAAACATGTAAATTTATCTT
GCATGGTGCTTCTTTAAACCAACAAAGTTCTTGTGTAATTGTACAGTCACGCACTGTTTATCACATCAATCTTGGTCGCTT
CAGCAGTATGTATGAGTTGTTTTCTCGCCTGATTCATATCTTACTCATCAATGTTCTGTCCATCTGTCTCTGCTGCAGGTC
ACGAGCCGGGGTTCTGTATGATGTGCACCATGCAAAACCACATCATTCAGGTTTTCGCCAACTCTGGGAATGTCATTAAGC
CCATTGGTGTGCTCAATGAGCTCAAAAGTAAGTGTCTGTACAGCTAAAACATTGAAATTATGCCTTTTAAACATTTTTTTT
TTTACCTGAAATGAATGACAGGAGGCGCTGTGTGGCTGTTTACTTTTTAAATTTTGGAGGGAAATTTTCTGGTGGGTGTTT
GTATACTGCTGTAAAAGAATTTGAACTTTTCATCGACTTATAAGTGTGATATAATCATCTATATCGACATTTTTTGTGTTT
TTTCCCGACACAGGGATTGCAAAGCACTTCCGCTATGGAAGCCAGGAGGATGCTCATGAGTTCCTGCGGTACACAGTGGAT
GCTATGCAAAAGTCCTGCTTACCTGGAACCAAGTAAGTGTGTTCCATTTTCAAAACCAGATAACGCTCAGGATAATAAACA
ATCATACACTTGCTTAGAAAGTTTAATCATATTTTTGGCTTGATGAAAATAGTTCATAAATATTGATATGGGCCATCGATT
GTTTAGGGAAATATTACTATGTTCTTTGTATTCTTTATTGTGATCTGATGATCCATTGAACATTCTACAAAGGTTTCCAGT
CTAAATCTTCCATAAAAGCTAAATGCACATACATTGAAGTATTCACGTTTTTCTGCTGAAATGTATGAAATCAGTGTAGAG
CCATTTCCAAGTAAGTCATGTAAACACACAAAGCATACATGCATTTTTAAAAGATACGTTGGGCAGAGCATTCAGGATCTC

AGGGGACTTTTAAAAATGGATTTTAGAAAAGTATCAAGTAGGTACTTATTCTGTAGTAACAGGTGGCCTTTCAACTAATTA
CTCTTGATCTAATTCCTCCCTTTCTTACTCCTGCACTTTTTGCTGTAATATGTTGTAACTTGGCAGTGCAAAATTCTGTAA
AGGGATTCTCTGTGCCAATATAGCCAGAAAATATGTGTCTCTGTGTTGGATAAATAGGTATAAATAATAAATCAGTCAATT
ATGTCATTACACTATTTGGTTGTTTTACATCCTTATTTTTGTGTTTGTAACAGATTGGACAGGCAAACGCAGGCAACCACT
TTCATCCATCAAGTGTTTGGCGGGTATCTAAGGTCCAGAGGTAAGTTTTACTAGTGTTGATTTCTTTTCTTGATTTATTCA
AGTTTTTGTTTTTGTTGTTGTTGTTGTTGTTGTACAGCTTTGGTAGACTAAAAGAAAATTGTTGATTTTTCTTTTTCAGTC
AAATGTTTAAACTGCAAAGCAGTCTCGGATACATATGACCCTTTTCTGGATATTCCTTTGGAAATTAAGGTAATTAATCAT
TATTGTCACTTGTGGGTTTGAAGTGTCTATTCAAGTCAATGAATTCAGATTTTTGTAAGGATCTTAAGAACAGTACAAAAA
AAATATTTTTCTTGCCATGCAGACGGCCCCCAGTGTCTCAAAGGCTCTGGAGCAGTTTGTCAAGCCAGAACAGCTGGATGG
AGAAAACGCCTACAAATGCACCAAGTAGGTTGTTTTACAGACAAAAGCAACTCAGAAAAGATGACGAGAAAGAGACAGTGC
TCTGTTTGTGTTTTTCTCTGTCTGTCTGTCAGCTAAACAACTGAACATTGTTTCACATAGGTGCAAAAAAATGGTCACCGC
CTCAAAGAGATTTACCATCCACCGCAGCGCCAATGTGCTCACGCTCTCCCTCAAACGCTTTGCAAACTTCAGCGGAGGCAA
AATTACAAAGGTACCATGACATATGCAACTATAATATCCAGAACAATGATAAGACTATAGGACAACACCCAGCTTTCATTA
ATTGGATATTAATCAGCTATTTTTTGGTCCTCAGGATGTGAAGTATCCAGAGTACCTGGACCTGCGGCCCTTCATGTCTCA
GACCCAAGGGGAGCCCCAGGTCTACGGGCTGTATGCTGTGCTCGTCCACTCTGGATTCAGCTGTCATGCTGGACACTACTT
CTGCTATATTAAGGTACTGGCATTGCTATGGACCACTGTTCTAATCCATCAGGACACTGTTGCCAAGTCGCATGCACACAT
TTGTCTCTGCAACACTCTATCAACAACTCACTACTAACAGTTTAGTGCAGGATTTCCTACAACAAGTCTTGCTCTTACAGA
GAAAATGTTTTTTAAGTTTTTCATGGTTTTATACACTTCAAATGAAACTGAGGAAAGTAAGATGGAAGATGTGAAATGATG
ATGATTCTGTTAAGCAAATATGATCAAACCTGACAACTGTTTTGTTGTGTCTCTCAGGCAAGCAATGGGCAGTGGTATCAG
ATGAACGACTCCTCTGTGTCTGTCACTGACATCAGGTCTGTTCTCAACCAGCAGGCCTATGTCCTTTTCTACATCAAGTGA
GTAAACCTTTTGAAAAATGATTGTTGGGTCATAAGTGAGCTTGAATTAAGAATCAATTTATAGAACAGAACAGAAATGTAA
TCAAAGCAACAATTTATTATAAGTTTGTCCTGTTGTGCTAAAAATGTAAATACATTTTAAATGTGCAATATGTAGGAGTTT
TAGTTAAAAACATTAAAAAAAAAAAAATACTAGAATTATCAACAAAATGTGACATTGAAGGTTATGTCAAAGAAGTCTATGA
TGTTAGTATGCTAACCAGATAGTGCCAAGCAAGGGGAAACTGCCTCATTCCGGTGTTTAAATGCTCCTGTGCTAGTGCTAT
AAACACCAACACTGGTCACATTGGTTGTTTCAAGTGTTTCCTGTTGCTAATTTCCATTTTTTTTGTGTCACTAGGTCCAGT
GATGTGAAGAAAACAGGGGACTACAGCCACATGAGCCATAATCCAGGCATCCCTGGTCAGTCGTCTCCACGACCAGTGGTG
ATACCACGCATCAACACCATCCACCACAACAACAACATTGGCTTCATAGGTCCCCAGCTGCCACCACACATGACCAAGGTA
CTGCAGACTTGTTTGCGTCTTAATTCAAATTTATCACAATTTATCACAACAACACTAGACACTCATACTATGCATTCTTTT
TTGTTTCTTCCTCAGAGCGCACTTCATGTTAATGGGAACGGATCTCTGAGGGACTATCCCACCAACTCTAAGCCCAGCACC
AGCAGCAGTGTGGTGGGAAAACCTAGCCATGGACTGGCCTCTTCCTCTTCTTCCATTTCCCACTCAATCAGCCGGCCCACA
ATGATCCCAGACCACGACAAACGGCAGAAGCTTTCATTCTTCATCGGGCAAGGCAAACAGAGCCGCCCGTCCTCGTCCTCT
TACAGCCAGCCGTCCTCTGCCAGCAGCAGCTCCTCCTCCAGCTCGCTATCCTCCTCACAGTCTACCTCAGACGTCCACTCA
GACATTCGCTTTGTTCCACGTCAGCTTAACCACGTTAACGGCACGTCTTGCAGTAACGGGGATCGACACCCTGGAGGGAAT
GGAGCGTCGTTCTTGGTGCCGTACGGCCAAGAGTCCTCAGAGGAATCAGACCAGGAGAACTGTGGCACCGTGGATAATGGC
TGTTTCGCCAAGTCTCACCTTAATGGAAACACCAGAACAGGAGAAGTTTTTGATAAATCTCCTCAGGCCACCAATGGAGAG
TCAGGGGTGCACCACAACGGTAACAGATTGAACGGACCAACTTATTGCGTCTCTAAATCCAGTCAAAATGGGCACCATTAT
GGACACCACAAAGTGAATGGACACAACACCCCTGATAAGGTAAATTATATGCTTTATGTTTTGCTATCCTATCATAAAAAT
ATATTGATAAGTGAATGAACAGTAGTTGTGTGTCTTAATGAATAATTGTGTCTTGATTTTTCCCTGCAGATCTCTGGCAGT
AATGGCAGTTCATCCTCTGTGGCTACTGTTGCTACTAATGGACTAGACAGTGAGCATAGTCAAACAAGGTAAGATACACAC
TTGGTTGTATTGACATTTTCATTGTTTGGTTGTTTGTTTTCACTTTTAAACAATGCACAAAAAGCAGGGTTTCTGTGAGTC
CTTTTTTCTGCCAGTATTCACATGATATAGGTCTTAAATTGCATTCAAAATGGTCTTTAAAAAGTCTTAAATTTAACGTGT
TGAAACCTGCAGAGACCCTGAAAGTGTGATATAAAGCAGTATTTATATTGTATATTTTGTTTGTTTTCCAGCAAAGAGGCA
CATAATTCTGCGTCATCCCAGGCCAGTTCCTCTCAGAGCATCCATCCTGCTGCCAGTGAAAGCCAGCACCTCTCCACTCCT
GACACAAGGGCTAAAACTGTGTCCGAACCCCTGCCTCAGCATACAGCGTCCTCCGCTGTTAGCTCTCTACCTTCAGCTACT
GCTACAAAGACTCATTCTGTCACATCTAGAGAATGTGCTTCCTCTTCTGCCCATCATGGCGACATCAATGCGGCCTTATCC
ACCCCGCCAGACTGCTCCCAGAGCACCAATGGGCCCTCTGCAGCGCCACAGAAGGTTAGCAGAGGTGGGGATGAAGCAATG
GATCTACCAGAGACCAGTGATCCGTCAGCAGGGACTGAAGGGTGTACAGATGTTAAGGAGCAGCAGCAGTCCAAGCCCAGT
TCTAAAGGCAATGAAGAGATGAAAGAGTTGAAAAGAGACAGACCGTACTCTGACTGGAGCAGAGACCG

SCAFFOLD 3 (109,998 nt); encodes pvalb6

ACCATAAGGAGAGAACTGGCGTGGAATACCAACTAGAGTTCGAGCAACAATGTGTTTGATACACGCGGTTTATTTCCAGAG
ACACATTTACTCGCTCACACACAGCACAACACGTACGCAAGTGGAAAACCATACTGCAACCCAGCCTTCATGTATCCTCGG
CGGAGTAATTCCAATCTTTCTCTGCGTATACATTTCCTCCCTCCATCCATCTTTCATAACCTCGCGTGCACCCCCTCCTCC
CCCCATCCCCCTCTTTCTCTCTCGTCGTCCATTTCGAGATGACAGATTGTGGGATAATCTAATCCCGCATACCTGCACCAT
GGGACAATCTGTCCTCAGCTGTTGCTCTGTGTGGTGATCTGTGTCGTATATAAGCACCATGAGAGGAAGCGTGGGGCAATT
AAAGAGGAGGAAGGGAGGATGGAACAAAGGACGACCCCAGCTTGCCTTTAGAGTAAGACATACAGTATAGTGAACGCATGA
ATTATGTAAAGACTACCCATTCATCAAGCTGTGTGAATATGTGTCAGTGTGACGGGGTCTGTACAGTGTGTGATTCCACTC
ACCATACAGTAGATGTTACAAGATCATTAGCTCACATGCGTTCTCTCTTGTCCTTGGTGGCTGTGCAGCTTTGCCAGTTAA
TTAGACATATCCCGAGGCACGTTATGAAGCTCAGCACGCCACACAAATTTATTGACTTCATATAATTCTGTCCCTGGTATG
AACATGATCATGCTTTGACTCACTGAAAGACTGCTAGAAGGAGGAACGGGTTGATGTTAAGCATTTTACAGAGCTGAATTA
TATAACATAACAAACAAGCTAGTTAGCCATTGTTTGGAGATAGCATGTTTATATTTTGCGGAGTGAACTATGAGTATCCGA
CCTTGAGACTGACCAGTACACACAGAATCACAACGGTAAAAGTCCCTGGTGAGCCCTCCATGGATCGGACTTGTTCCAGCA
CGTCCCATGAATGCTCGATCAGATTGGAATCTGGGAATTTGAAGACCAGGTCGGCCATCGTGGATCGAACTTGGCTCTGGC
CCACCACAGCATGAACGCAGGCCCTCTGGAGGTGTCCTGTGGTGTCTGGCACCTAGGTTTTTGGCAATGAATCCTTTGACT
CCTGTGGGTTTTTGAGGTGGATACTTCATTGATCAAACTTGGGCAAGGGGGCCAGACGGAGGGAGGTGGTCCCCCCACACC
TTATGGCCCTGGCCCATATTCGTTTTGATACAGTCATGAAACTTCACAGGTGCGTAGTTTAGATTAAAATTAAGGCCGATT
TTGGAGGTCAGTGTGATCCAAACCATCAGTACTGAGTACTCATGTGTTGGCTTCTGACTTTGCCAGATCATGAGAGCAAAG
GCCAAAGTTGTTAAGCTCAGCGACAGGCATAAATGTTGTAACTTGCATAAATGCTATTACAAAGTAGAAAAAGTTAGAGCT
CAATCATGGCAAACAAGCTAGCTAGCAGGTGCTAAAATAACTAGTCCCACGCCAAAAGTGATATAATACTGATGACATCAC
TATGACATCATCAGTGCTGTTTTTTTTATATTTGGCCCGGCTCGTTCACGGCCACAGAAGAGCTCAACCAATTTTCACATG
CTGTAGTCTATAGTCCTCCCCTTGATGGAGTTTTAATAACATAGTCTGTCTCTCCAAATGAAAACCTGCCAGCTGGCAGCT
CTGTGAAGTATTAAATTGCTTCTGAAAAATTATATTTATTGAAAGTTTTTTTATTGTGTTGTGTTATTGATTCTGTTCTCA
CATTATGTTTTATATGCTGTGTATTCTATACGCTCTGTATATTCCTGCTGTTGTCTCCTAACGTGTTTTTAATTATCTTTA
CGCCTGATATTTTACTATTCCTGCCTGCCAATTTTATTGTTGTTCCCTGATCTTAAATTCATCCTTTGTGTTTTTTGAAAT
TGTTTTGTCTTATACTCTTACACTTATTTGTTTTATGTTGTTTTTCATGAAGCACTTTGTTATTGATAACTATCAAATTAA
GTTACTGGAGCTGTCATTAGGCGTCCAGTTCCCCAGCCAAAGTCGGAGAAACAAAGATTTTTGTGTAATATAAACCCAGCA
ACACCAGGATGAGTAATCAAAAGCTACATTTAAATGCACCTTTGTTTCAGATCTGTGCTGTTTTATCTTTGCAGTTGTCAG
TCCGCTCTTGCCTCTCCGCTCACCAGCTCACATGCTAACTATGCCATGCTTGACTGGAGCTGCAGCGTTAGATCAGTCCCA
TTACAGCTGTTCAAAGTGCGTCATCAGTTTCATTCTATTGTATTCTGGTTCAAATGTCATTCGGTGTTGAGTCGGACTCGC
AGCCAAGCACTGCTCCTGGTTGCTTGTTTTTCAGTAGAGCCCTGTAGGGACAGATTAATACCACAGAGCTATGAGAGAGGT
CAGAGGTCATCACAGCTTACAAACAGAGCCTCAGGTGGTCTGAAATGAAGCAATATGTAGACCTTTGAGGTTGGTAGCTTA
CAGCAGCATGACACAGTCAGTATGCGGATAGTGGCACTAGATCTGGCAACCTCCTACGCTAGTGATGACTCTACATCCAAT
AGTCAGCTGTGATTGTTTGTGACTGTCTCACTAAACCTGCAACAACTCATTCAGTATCTTGGTACTAAGCCCTCCCCTTCA
ACACCATCAGAGACATTTAGAAAAGGACACGAAACAGCCTTTTATTACCTCTTTTATTTCAGATATAAAATGTTTACATGT
TAGATTATAATAGATTTATGTTTGGTGCTAACTAATGCTTTGCTCTGGCTCGTCGCCATATTGGTGCTGTGTCACACTACG
TCATGAGGTCGGTTAGTTGAAAAGCCGGCCCAGGAAGACATCAGTCGCTGAACATGAATTTAGCTTTATATGGCCAAAGAC
TAAGCTGAAAGTTATTGTGAAGATGACGATTTTGAGGCAGTGAGGAAAATAGACTGAGGTACTAATACAAGTTAGTTTAGA
AGAGCGGCCATTGGTTCCCCGAGGTGCGAAGTGGGCGGGCAGCACCACCTGTTGCATGTGTGACGTTACTGAAATGTCGAC
CTGTCATGGATTAACCACATGCTCTGCTGTGGGGGACGTCTTGAAAGTTTCCTTGAAAGTCTGTGCTGAGGCCCTGGTGCT
GCCCATCAACAGCAGGGATTTTAACCAGAAACAGGAAACAGGACAGAGTACCTCCTGTTAGAAACATCAGCATGAATAAAA
CAGTCTTATGGCTAAGCCATTAAATAAAGTCGATGTTAAAATCCACAGTCGCTGAAACTGGTAACCTTTTTGTTTAAGGTG
ACATAATATGACACGTTACATATCAACAAATGATAGACAGCACTGTTAATGCACAAGGTATATTTTATTATTTTTCGGTAT
AGTTTTGTACGTTTTCCTACTGTTTAGCTTCGTGTAGCTGTGCTGGACCACAACCATGACATGATCAGACAGACTGCGAAG
ATCAAAGAGGTGGAAGAGCAGATGAAGCACCATTTCTCAAAGGCTGTATGTGTGCAAATTGGTTGTAGTGATGATGGGCTG
AACTCTGTTGCCGGTTAACCGTAACAGTGATCACGGGTGAAGACTTGGGTGAAATGGAAATCGTCTGTTTCAGTTCCTTTA
TTGCTGTACTGCTTAGCCGTCACTGTCATTACTAGAGTACTCACACTGACCCTTTTCCTTAGTTTCCTGTTGCTCCTCAAC
ACTGGTGTCTGTCTCTGTCACACCACTGCTGCTGCTCCAGTGTCAGCTTCATCCAGACATAACAGTGCAAAAACAGTGTGT
GACGTGTAACTGCACATGACTCATGAGCGGCAACAGTGTCGTGTTGCTGATACATGCTTATAATTATTAATTGATGGGTTT
TATTTTGAGTGATTGCGTGGCGGATTTGGAGGATCCATTACTCATGAAGTCAGACTGAAGACGTGGGTTGTGTTTTTTTTT
TTTTCTCCACTAATTTTTAAACAGTGATGAGGCGAGAGCTGAGAGCACACATGAGGACAGGGAGAGAGGGAGAGAGAGTCA
CTTTTAATTTTCTCAAGAAAGGGCTGTCAGCATAAAGCCCAGACTGCAAATTGCTTGTGTGTGATCTTTTTTTGCTACATT
TATGGGGTCCCAAATCCGGGAGCTCACTTACAGGGTCCAACAGCTTTGTGGGGACCAAAATGCTGCACTCCTTAATGTCCA
TCATTAAGTGTTGGGTTAATGTTCGATAACATTTTGGGTTGGAGAATTAGAAGTCATGGTCATACATCGAGTAAGGCTCCA

GGAAATGAATGTAAACCTATGTAATGTCCCCACAAGTGACGAAACCAAGATTTCGTGTGTGTGTGTGTGAGCGTGTGTGCG
CATCCATACCTGTGTTTGTGTGTATCTTTCTCCCAAACGGAAAATTGATTTCCAGATGGCATGAAAAGCCCTCAGAGGACA
CAAGTGTGTGTGCTCGCTTAGAAAATATTTGGAGGACAAAACACGGAGATGGGATACATTTTTAATCGGGCTGTTCAGACA
CACACGCACCTTGAAATGCATGAAGAAGATTGGAGGGTGACTGAGTGCACAACACATTTATATTTCAGTCGAGGTGATAAA
ATAGACTCCGCCGCTCCTCGTTAGCTCGTCGACTGATCTGTGGCCTGTGCTTTCACGAGGCGGCTAACAGGTTGACAGCAG
CACACATGCTCCTTTTTATGTGCTAACGACGCTGAACTCTATGTCTAATCTGAAATGGCTTACAGAGACACACTAACAGCA
GACACATTTGATTCATTTTCCTTTTTGTTCCTTTTTGTTCTCATGAAATTGGACACTTTCGACTTACTCTGCATTTCTCAC
CCTCCGTCGTTCTCACACTCATTCGCTGAATCATCTTCTGACACTCATTTGTCTTCACACCACACTTCTATTATCCAATTG
TTTATGCAGCACACAAAACAGGGAATACATGTGCATAATGATAAATAATGAAAAAAAAGACTGTATTTTGAAGATGAATGC
CGAGATTGACGGATTCATACAATTCAAATAGCTCCAAAAGCTGCGAGGAGAACTGTTATCAGTAAGATGTCAAAGTTAAAA
CAAAATGCTCGACAGCCTCTCGCCTGGAGACACAAACCACTGCCATTTGTGTTTAAAATCACAGACATGCAAAGCGTGAAG
AACACATTTGCGCAAGTTTAATTGACGACTTTAAAGTACAAAGCACAGAGGGAATCATTCGGGATGTGTTTTATAAATAAA
TGAAGGTGCAAAGTGGATTGTTACAGACAGTCACGACCAGGAGCCAAGCAAGCTGCGTTTGCTAACACTCGCACACACACT
TTCTAATGGAGCCAAAGCCCTCTCTGAATTAGATCACATGCTTCAAAAATTATCATGCATCTGCTCACCCCAGCGCTAAGA
AATTGGAGGAAGTGAGAGACGGAAGGTTGAGAAATAGGAGGCTACAGAGACGTAATAAGCAACAGAGAGAAGGAGGGAGAG
GGGGAAGGATTATGTTAATGTGGAAATAACTGATCAGGAAGGGCCGTGTTGTCTGCTCAAGAAACTGATTAAATTCTAACT
AATGTGGACAAGTAGAGCTCTGAGCCATGTGGGGAGACACTGCAGGTGAAAATGCTGGGTTTTCCTGCACTAGTCAGTTTT
TGGGGCCAGTTTGCTTCAATAACATGTTCTCTTAAAAACTAGTAGAGAGAAAACTTGTAACATACACATAAACGTATGTGT
TTTAGTTTATGACGCCCCTAAACTGCCGCACACTTTTACTCATTAGCCTTTAGTGTCGCCTAATTATGTGTCTAATTTGCC
TGTGCCTTCCTTGCAATTATTATTACTATTATTGCGACTGTTGTTGGTGCACATCGCTGCCATTGTTGTATATTATCATCA
CTCTGTTAACTCATTATTTTTTAATTATTATTATTATCACAGCATCGTCACTCTAGTTTCCTTTTCCTCTCAAGTGTTTGT
CTCATTCTTGTTTTATTCTTTTTCCCCTGTGATCTAGAAAGCACTTTGGATCAACTGTGTTGTGTGTGATGTGCTATATAA
ATAAAGTTGAGTTGAGTTAGAGTCTGTTTAAGAAATGTATGCAAATATAGTGTATTATAATTTGATAACCCAATTAGCATA
TTTATACATAAACGTTCAGAAAACGTGTAATACAAAAAATGTACTGTCTTAATGTGAGTAATCAGCTAGGGAAAATGTCAT
GGTGATATCAATTAGTTAAAATCACAATTTTCTGTCTAAAAATAGCTGGAATTATACAATACATATCTTTAAAGGCTCTTT
TCTACTACTGAGCCAGAAGTCTCCACTTCAGCACTTACACGCTCCTTCTAGCTTTATATCAACAAAATGAAACAAGCATTT
TGAATTTCGGCTTCATCCAATGTTCAAGTTTGTGCATATTTAATTGGATAGTGCGTCATTTGCATATTTTAATATAAAATG
TCACAAACCTTGTAATATCGAGTCGGGATGTTTCATATCAGAGAGTTGCCATTGTATGTGGGATGATATAATACTTATATA
AACCTCATGGACAGGTGCCAGAGGAGCTGCTATTAAAACAAATGAAAGTCCTATTTGAAAATGTATAGTAGTGCATGGTAG
TGTACAGTTTACAGCTGTAATAGAAGCTTCATGTGATAAATCCTCACATTAACAGAGCAGACTGTGAATTTTCAACATGTG
TACACAGATGTTGCGTACATTAATGTATTTCTCTGTTGTGAATGTTTACAGTCGTGTTTGAATGACAAAACAACAACATGA
TAACTCACAGACACTTTGTGATTGCTGAGTGAATAACATGTAGTTAATAAGCTGCAGCCTGAATGCATATGATTGTATTAC
ATAACAACAAAATGGCCTCCTGAGCAGAAAGATTTCTCCCATTTGGGTACTTGATGTCATCTTTCTGCTCTCTATTCACCA
TCACCATCTAGAATCCAACAGGGGGATTGAGTCTTGTCTCTCTCTGAGCTCCTGTTTCATAAAGCTGCTGCATGTTTATGG
AGTTTAATCGCTAAAGATCTTCTCTGTAATTATGTAAAAATGTATCTCCACGTGAGCAGGAACATTGGCGTGAATGTGAAT
GTGAGACTCCTTCCACTGACTGCCCAGCTGAATGTGGGCGAACGCTGCATACTTGAGATTTCTTTCATCAGAAGACAAAAT
AACCACTAAAACAATTACCCATGGGAAAACGTGTGAAAGAACGCTGTTTCCAAAGTCCAAAATACTGACTGCTGACTGAGA
TAACACTCAGGCTTACATAACCTCAAGAGGGTCGATACTGCTGGATTATTTTTACCAAGCACACTGTTTGGGGTGCATTTT
TACAGCAGTGAGTATGTATACATTTTTAACATAAGCAGCCTCGGAGGAAACAATCCCATTGACTTTGGCAGTATTACAACC
ATGCTCATGGAGTTATACAAGGCCACCTACCTGCACTTGAAAGAATATTGTATTAAACCGTGGAGGATAAGTGAGGGAGGG
AGGGAGGGAGGGAGGAATTTGAGATGCAAAGTGGGTGACAAAGACGGAGGAAGAGCAAAGCGTGTTCCTGTCCAGATGGAG
CCTCCGTGGGGATTTTGGACCGTATGTGTGTGTGTGTGTGTGTGTGTCTGTGTGTGTGGATGAAGAGTAACGATTCTGGGA
TACTGCCATGTGGAGGCTGAGTAGGTGGATTACCTACTCTGGTTCAAAAGGGAAAGTGATGAGTGGGTGGATTTAAGGATT
TTGATAGAAAGCAGTTATCATGATGACCGCAAAATGTCAAAATGCATCCGTCTGGTGTGAATACTACTCTGACAGAATGTG
AGCAAAAAACACAACAATTATAATTCATGAAATCAAACCCAGTTTTTTAAGATTATTTCTGGTCTGCATTTTTTTCACTCA
TAGAAGAGTAGATTTACTTTTATGTGGGAATGCTGTAATTAACACGTGTAAATGTACTTTTCTTACGTTGAAATATGTATC
AACCTGGGCTGAAATGTTGAGGATAACAACTTTAACAGTAGTATAACCCCCCTGAAAATCTTGTTTATCTTCTCACATTAC
TGCAGTGGTGTCCAGGTGGACTCCAGGACCGGGTGACATCACTGCTGAGGGTGGCTTCTGACCACACCCACCCGGCTTGCA
AGTTTCATTTGGAGGGGGCAGCAAAACACAGCTTTTCAGCCTGGTATTACGTTTCTCTTTAACATGATTTGCATCTTAACT
TTCAGCATGCAGTAACATTTTTGTTTTCAAGATTTAACACCCCCCAAAACAAAATTATGTTACTTTCCAATGGGACACTCC
CAGCAGAGCAACATGTCATTTTGTGATCAGACACTTCCTGGAGCTGCTCATTTGACAAAAAAAAAAAAAAAAAACAGCTTTG
AGGCTGAATATCTGCTGGAGCATATAAAACCTAATGAGTTTGGATTCAAACAGCTTTGAATCAGAAAATGTTCCCCTCATC
CAGACAAGAGCCAACATATCTGGTCTCTCATTAACAGTGAAGGAGGCTTGAACACAGTCGAAGAGAAGCAGAACATCACAT
GCAGTGACATAATAAACTGTAGACTAATGGACCGTCATCTTTTCGCATGAATTAGTGTTAACTGTGCCTGAGAGTATTTGA
TTTCAGCCAAAGTCTCCGCTTGTTGGTCCATCCATTTCATGGATTATATGGGATTACAATCCCAATCCATTCCTAGATGGA

ACATTATGGATTAGATTGGTAATCCAACAGGGAATATCATCTTCTGGCTCTGGACGCTGTTTATTGAAGCCTGGTGGAACT
GAAGGGTCACAGGTTAAGTCCCTGCTGCAGCCAGTACATCTCTCCCTGTCAGATTGTCCTGTGCCCTCTCCACACACACTG
CTGCAGGCTTCTACTGTTTTGTATAAATTATCATGCCTCAAGGGGAAAAATGCAAAGAAACAAGTTCCCAAGTGTGATGCT
TTTGTTCGAGGTGCTAAAACAGAAAGAAACAAAGTGAGAAGAAGCCATTTGAGTTGATGTGTGAGAATCACACATCGAATT
TGTGTCTCCTCTCTTCCGTTCGCTGCCGCTGAGAAATGTTGAGAGCGCCAGGAAAGAAATTAATACCCGACAGCCTGTCTG
AAGCAGTTAGAAAGTAGCTGTGACAGGCAAGTTTTGTTAAAGCTGCAGTACCATAGCAGGTGTGAGACACTTAGCCAAAAA
TACGCTTATAGACCTGTTGCATATTGCATAACATAGCTCAGATTCGTGTGTCTGTTAACCAGAGAGATGCTGCACAGCCAC
ACTCAGTGCATCAGTGTATTGGAACAGCATGTTTTAACCTCCTAAGGCTGAATCCATCACAACATGTAGGGCCAAGAATTA
TTATATTATAATTATATACCGGATCTGATAATTGGTCAACCTATAGTATACAATATTTACATACATTTAAATATATGAATA
ATTTACTTTTACTTGTACTTTTGATAGTTAAGTACATTTATTGCCAGATACTTTGAGACTTTTACTTGAGGTCTATTTTCA
TGGGTGACTTTCACTTTTACCAAAGTAATATACTATTTGCTGACAAACCTCGTCAGAAGCTGTTCGAATTCTTACACAGAG
ATAAACAAAACAACAATCTTGGACTTGAAACACATTGTACAATTATTAATTCACAATCATTATAATGTTTTAAAGGAAAAG
AGATACTTTTATTCTGCACCATCACAAGCTCTGTAGATGTATTATTTTCTAAAAATGATTTGTCAACATTAAATATTATCA
ATATGACATTTAAAATTCAACATCCAAATATATCAGATGAGATAGCATTTAAAATGATAACAAGACACCTTGATGGTTCCG
TTTTCTTTAAACTTAATATTTCACTCATTTAAAACACGACATAGCTTTAGTTAAACATGTAACATCAGTGTTGCAGGGGTT
AATATTATGTTCTGAGCAGTTCTTACAGACCGCAGGCTGCACGCTGCACACTAATGAGGTTAATAGGACTTGACTTGGTTT
GGATCCAAGTTGTGTGACATCAGCCAGCTGACTCTGACTGTCTGGCACGACCATCAGAGCTGTTGATGGTCTTAAGTCGTA
AGTCCATTACTAAGAGAGAGGATAGCGTGAACAAACGGTGCTCACCCAGTCTCATCCACTGGAAGGATTGAACAGATTGAC
TCATGCTAATTAGGGATTACGGATACAGGTTGTTCTGTTCTGTGTCTGTCAGTTGCTGTACTGGCTAGTAGGCTCACCACA
GGCCCACAGAAGAATCACGACTCCAAAAAATTATGTTGCATGACTTATGTCTTGAGGCGGCAAAGCAAAGATAAACATTTG
TTTTTAAATCCCCTCTCATAGCTATTTGTTATCACAAGTCTCTTGTAAGCTTGTTCTCTTCAATTTTTTCCCCAACATATC
TGTTAAAGTGTCAGTTTTTCGTGACAGCAACCTTGAAAACTCAGAGCCTCGTGCTGTGCACGCATCTCCTCTCTGTCAGTA
GACATGCTGAGGGTGTCTTTGGCTGAAGGGATGGTTGAGAAATGGGACACGAGGCATGCAGGGATTGAGTAAAGAGCAGGT
GACAGTTGACTATGGAGGAGGAGGATGAGGGGTCTGATGTAGAAATGCCACTGGGAGGAGGAGGAGGAGGGAAGAAGATGA
GAGGAGGGTGAGAATGGAGGATGACAGAGAGGTTGGGAGAGTGGAGGCAGAGTGGGAGTTGAAAAAGTCATAAGTATCACA
TATATACAAACTGGAGACACAGAGGTTACACAGTAATGGCATACAGCGATGCACAATTAGAAAAACACCTGTAGTCTGTAT
CAGACTGCTCATAAAATGTCTTTGCAGGCCTAAACATGAATACAAATATGCTCTGTCCATTTAAGATTTGGACTGAATGTG
AGGCCCTGAACACGTTATGCCTCTCAAACACATGCGACAGAGATAGAACCAGTTTTTTTCATTACTACACGAGAAGCAGAT
ACACAGCAGCTGCCTGACAGGGTCTGCCAAATCCTCATTTTACTCTTAACTCATATCTACACCTGCGGCATCTGAAGGAGA
AGACTCCAGGCTTCTCTCCTTGAACTCTCTCCTTTAATTTGCTTCGAGGAGGATTGTTTGGAGGAGCAGGAGAACACAGAC
ATATACTTTATTTTCTCTTGTTGAAAAAATGAATATGTTTTTTTGTGGAGTCTGGAGAGCACTCTCACTGTAGGAGGGATT
GCTTTCACAGCAGGTTTTGCTGCCGCTGTGCTTAAATTACAAGTGTGTTCGTGCATGTCCTTGAATAAAAGAACAACTGAA
GTTCTAGTACACTTATTCACTGTGCTTTTTTCTCTGATGCAAATATAGTGTCAGATACCAAGAAAGGAATCAGATTCTTTA
CATCTGAAAATTAGGACTTTAATAAAGATTTGAGGTTTGTACACGGCACATATGCAGAACAATACAACACCATACAAGACA
CGGACGTACAGTATTTATACAAACAGTCGCGTGAAAACAGACAAGATACTGATGCATACAGTGAAAAAAAAACAACAGAAG
TCATGTAAGAAAAGACAAACATTGCCAGTAGAGTTAAAAAGCGTTCAATTTCAATAACTGTAAACAGAATAATGCCACGAC
ACTATCAAAAAGCCCAAAAAAGTTGCTGAACAGACACCAGGCATAATAGTATTAACAACACTGAAGACTGAGAGGCATCAT
CAAAGACACTATTAGTCACAGCAAGATCATGCTGACCTGATTTAGCATCAGTTTGAACGGCACTGCACAGAGAAACTGCGT
CTGTGTTCTCAAATGTTTTATCAAACCTCTTAAATCTTAAAAAGATCCCCGGGGGATTCACAGAAACACTGACGCTTAATT
AGCTCAAAACATCTCGCGGTGTGAATTCTCCATCAGGATAAATTTGACCATTTGTTTACATGGGCGCAAACACCCCGACAG
GCACTTTCCACTAAGGATTGTTTTTATGCTATAGGGAGAGCCAGCGTTTGTAGGAACTTTGAAATGAACAGTTTGTATTTC
ACATCATGTCTCATACAACAGTTGAGAATAATCCTCAAAATGACGAGGTATGTTGCAAACATACAGCACGACTCATGCATG
GAATGTAGGAAGGCTGGAAGAGTCGGAGGTTTCCGGTTGTTGATTCATCAAACCACACTGTTGACTGGTTGCAGGTATACA
GTTGCAAAGAGTGCATTATGTAAATTTGAAACTAATAAAACCATTAAATGTTATGTATGTTTGAAGGTTGCATTTAAAGGA
GACATATTATGCTCATTTTCAGGTTCATAATTTTATTTTCCTGTCTCCCGCTCCTGTCTCTTTAAGGCCCCCCCTTCTGAA
TACGCAGTCTCCTCTGATTGGTCAGCTCACACACGCCTGAGCCAGCACAAGTAACAACAACAGAGCAGCTGTGACTAGGTG
CCAAACTAGCTGCTGGTCATGAATCATGCAAATGCGTGACATGGTGACGTGGTGTGATGTCAGAAAGTCACAGAATTAAAG
GCAGTACTACTGACGAGGCGTTTCGGGAGCAGTGTTTTCTGTGGGAGAGAGGAGCTTCTGTTGGTGCGGACTTTGAGCTTT
TTAACTTTCAAGATCTTTTAGATCTATAAAAAACACAGAGGGAAAGGAAAAAAATGAAAACGCATAATAGGTCTCCTTTAA
GACCATGAATTCAAATATGTCCTTGATGAGAGCCCATACTTTGATGTTGATTTTCTAAATCTGCACAGACTGACAGAAATA
GGGCAAAAGCTTGCAAAAACAAATGTAAATGCACCCAAAAGAAAAGCAACATTCTGCTAATGGTGGTGACTTCCTTAGGGA
ATAAGACTCCTTGTTTTAGATTGATTCATAAACTTATTTAGGGAACAAAAGAGACCCTCGGGCCTTCAGAGACACAAAGAC
AGCCACTGATGCAGGAGGCACTGTCCTCTGTGAGCAACACAGTGCAGTGGCAAGACATTACAGGTATATGAACACATCATG
CAGAGGTGAAAACAATGTAGCATCGCTCCTTAACAGCACTGGATCCATCAGTTTCCTTTCTCTTTCATCATCTACTCCTTG
TTGCCATCACCTAGTACTTAATATTCCCACCACTTTCTCCAACATTTTTCATCACCTTACGTCGACTCCCTGCGCCAAAGC

TGTCACGGCTCCTAATTAACATCACCCCGACCCTGCTGAACTCATGGAGGGATTTCAGCTGGCTTGACACAACTTCTGGCA
GCCATAGTGGAGGTCAACAGCTCTCGACTGTCTCAACACAATAAATATACAGTGGGGATTTCTGCATAGTTTTAGCTGTTA
AATGACAACATCTCCAGTGATCATATTGGTTTAAAGGACAATGTCAACATTTTGGGGAAAAGGATTCTTTGCTTTCTTGCT
GAAAGTTATCACTCACATTGATGAGCTGCTAACTTGTTTAGGTTTGCACCGTTAGCGATGGACTTCCCATTTCAGCACGTA
GGTTTCTAGCTCGCAGGTATTCAGATAGCTGAAGGGTGGAGAATGCAGATTGAAATAATTGGCCGATAGCAGACTTATAGG
CCCACCAACAGCCTACATCTGGTGAATCAGAATGCTACATGCTAAGTATGAAGCTACCACCACCAGCCAGCTTTTGATTTT
TTCTGTGATTAAACAAAAGAACATGGTTAATTGTTGACCTTAGAGGTGCTGGTAGGCAAAATTTGCTGCTCCCCCCTGTCT
AAAGTGTTTATGCTAAGCTAAGTTAGCCAGCAGCTCAACTCTCAGCAATAAAGCAAATCAGCGTACTCTCCAACATGTTTA
ACAATTTCTTTGAGGGTTAGATAATGTCTTTTTTTTTTTAGCTAGCTAATCATAAGCAAGTTTGGTCAGCTAGCCATCACT
GTGTCGTTGCTAGCATTTTTCCACACTTATTAATAGCTAGCAGAAAAATAAATTGTGTGCTTGTCTTCTTAAAGTGGACAT
GTACAGTAGTTGCTTTTACTAATTAGATTCCTAGCTGGTTAAAAAGTAGGAGCTAGGACCAATGCCAACATAGTGTGGCCT
GAATCTGGCAACACATCTACAATTATAGATTATTTAATAGACTATAAGTAGCGTACAGAATGTGATCTATACCCATACTAC
AGCTATATTTATGAGTAATCTCATCTTGTATTGCACATTTCGCCATGGACCCTTCATAATGGGATCAGCTGTGGTTGCTAG
AAGTTTGGTGAAGTAACTGCAAAGCCCTCTACTCAGGCCATATAAATATATATCATTAACTTTCCATTTTCACATACTTGC
CAGTCCATGCCACATAGAATTGTTTAGAAAATCATGTAGCAGTGTTAATATTAGGCAAACTGATACCTTCTCGAAATCGCT
ACAACTTTACTCCTCCACCCTCCCTTCTTTCACTTCCCCTTTCCTCAAAGCCTCCTCCCATCAGTCATTGAGAGCTCCTGG
TGTGTTTACTACAAGGCTCCCCCGGGTCCTTGGTTGCTCCGCCGTGAACCTCAACTCCGTTCTCGGTCCAGGGGGCCACAC
TCCCCTGGGTTACTCTGCTGCAGCGGGCCAAGCTGGCCACCTGGCTAGAAGACAGGACCCGGTCCCAAAGACCAACCTGGG
ACAACTCTCCAACCAGAGCCTGGGATGAGTCAAAACCTCCGCCCAGGGAGTCCTGGAGAGAAACAAGAGTAAAGGTAGATG
GTTTACAGAGATGAAGCTTGAAAGGCGTTCAAGTGATTAGAGACAAAAACGCAGAGCTCAGCTTCGACTGCTCTGCTGACT
CGGGCCAAACTGTGGATTTGCTTTTGGGGAGATGAAGGAGATGTAGGACTATGGAATAAAGACACCATATTGTAAGGGTTC
TGTTGGCTCTGGATGAAGGTCACCCGGGCTATTTTATTTTTATACTTGTTTTCTTTATCGATTACACAGGTTTGCGCAGAT
ATCCTTGTTACAGTAGGACTTTATGTTTACTTCCATTCATATGTACAGCCTAACCCAAACCCTTACCCTAAGCTCTATTCA
TACCTTACCCCTAGAATTAACCAGGACATCAGAGATCATGTTTTGCCGCATTAGGACCGGTCTTTGGTCCCCATGAGGCCT
ATTGGCCCTGACGTGGTCAGTGTTTATGCCACTGTCCCCAGTAGGTAACCAAAACACACACACACACACACACACACACAC
ACACAAACACTCAACACTTTAGCTAGCAGTATACTTCAAACTAAATCAAACTCCTAGATTAACTGCCTCCATTTGCATCAA
GGCTCTCGAAGTATTTCATGCTTTTATTTACTACTTTTATTTACAACTCCAGGAGAAGTCAGTGAGTGAAAACACCCACAG
CTGCAGAGGGTAAACTACTATGTTCTTGAAGCGCTGTAGTCAGCAGTTGTCTGACGGGAATACCAAATTAGATTTCTCCTG
GAGTACCCCGGCAGGCTGACTTGACTCTAATCAGTTAAAACTTCATTCCTTCAGTCTTCCACACCCTCATTCGTGCGCTCA
CCTGCTCTTGCCCCAGTATGAGCACTCCTCCAGGTCTGATGTGATGCCCGGCTGCCAGCGAGTGACCCTCGCCCCTCAGTT
TCCCCCCCTGGTAGGCCTGCCAGGCTCCTCCCTTCTGGCTCCAGCTCACACAGATGTGCTGCCAGCTGCCTCTGGAGAGGT
TCAGAGGCAACTGCGCCACCTGGGAATAGGCGAGGAAAAGGGCAGTTCAGATGAAGTTTGGATTCTGATGTTCTTGAGACA
CATTTGAAGGTGACATCTCATTTCTCTCTCAAGGCTTCAGGGTACAAAAGCTGGCTATTATACAGCATTTCCTCTGTCCCA
AAGACATTGTGCTGCCTCCAAGGACACAGAACTTTTCCTCCAGGCATCTATTACCATAATGACTTTATTGAATTATTCCTA
CAGCCACCGCACCGCACTGCACAAGCCTGTAATGCTGTCAAGGTGGCGTATTAGTTTGTCATTTGAATACATGAATGAGAC
TGTTTAGCCTGTCCGCTAAATTGGCTCTTTGGTTTGATGTGTTCTGCTTATTTGTATGCTAATACAGCGCACTATTTCTTC
TGTAAGAGACATTAGAAACTCTACTCAAGACTCTAATGCTTTTTATAAATAGAACATTGGGTAAAAGTGGGCGAGTCGTGT
AGAAAGCAGGAGTGAAACTCTTCTTAGGTGAAACTATGGTAACTTATGTAAGATATGTACACTGTAAGTCTCCTCTACACT
CCATCCCAGAGTAGTGCTGCAGACTCTTGGATCTGTCTTGCATTTTTATCCCATCCCTAATGTTTTTATGAAAAAGGCACT
GAAATATTATCACAGAAAACCTTTGAATGATTTGTGAGCACATACATTTACCGTAACAGGTTCTTAATTTGTAGCAGATTT
CTCATCACATCTAATTAACAAATTCTACATGACTCGTCCCAGTGAGCACCAGGTCCGTCTGAACGTCTTAGAGCTCTCAAC
CAGCTAAAATGTTTTTACAACAGCAACGTGTAACAAAACACAACTTTGTGTCATCATTTTGTTCACTAGTAGGTTGAAAGA
GATTAAAGCGATTATGATTTAAGTGAAGGTAAGTCATATTGCACCGGTGGGCAAAATGAGCTCTATGCAAAACGTAATTTC
AACACAATTAATGTTTCAAATAAAATGTATAGAAATAGAAACTATAAAGGCTGCGAGCTGCTTACATAAAGCTTCCAGGAG
ATCATAATGGGAGCAGTGTTATACATTAGTTTAAAATAAGACATTGTGCTTTCTGCTGTAAAGCGCATGTGCAAGAACTTA
AAGCGTTACAGGTTTAACTACTCCAAACTATGAAACAAATATGAAGAAATAAATGTGACAATTCACCCACTGAACCTACAT
GTGGCCATTGTGGCTAAACGGTAAAGTAGCCATTCATCAATCAGAAGCTCTGCGGTTTGATCCCTGACTCCACAATTACAT
GTGTTGCTGCTTAAAAAAACCTTTTTAATCTATAATGTGCAGATAAGAGCATATCGTCATTTTGGAGCCCGACTAAGAAAC
CACATACAAAGCGTCAGTTGTAGCCAGTCTTCAAAGAGTTGGTGAAAAAACGTTCACTTTGAAGGTCTTTTCACAGGCGAC
CGTGGACGTTTTTTAGGCTGAGGGTTAGGGGGCGTCTTGAACAATAAAATGCTCACTCACAAACTCACATTCGTCCATTAA
AATACAGAATATATTTGAATGAACAGAGAGACTAACATATGTTGTAACACGTGCACGTAAATATATATCTTCTTGACATAT
TGTCTAATATATACTGCCAATATGCTGTGTCATTACTTTGGCGGGTAGCTGGATTTGCCAGATCAAGGGATCATGTGAACT
CGTTCATCTTGCCAGGCAGACCAGTCGGCGTCCTATGAGGAAGTAGTGGCAGCTATGGCCCTGGTTTAGGTGTTACAACAA
GAGAGAAGCCACTGTTCGTTAGAAAACAAACGTAGGGTTTAGCGTAGCTGCTGCTGTAGCATCAGTTATGTCAGAGCTGGA
GAGTATGTTTCATTAAAAGAAGAGTAAAGAATGACACTGAAAGCTTTTCGCGATGGGAAAGATGTTTTCGCTCCTCTCCCT

ATTGGCTTTGGCAAGAGTTTGATTAACTGACTGGCTCCACTGGTGGTAGCACTCTCCTCCTGATGATCTGCTTAGTCAAAG
TATGCCCATGATAGGTGATGATGATAGTCTAATTGCCTGTCAAGTGTTTTTTAAAAGTGACAGCCCTTTTCCAAACTGTTT
CTAATGCCTTGATTTCACCAGGAAGGTCTCTATGACCTTGCTTGTGCGACGTGGTTTTGTTCCATCCTGCACGCAGCTTCA
TTTCCCACCTGGAGCCTGATACTGTTGAGTTCATTTCATCGTTTCCCTTGATTATTATGCTTCCACCCTTGCTGAGTGTCC
TATACAGTTCAATTATTTATTTTGTGTCTATTTTACTGTACTGTAGATCCTGAGTATTTTGAAAATTGACTGGATGAGCAT
GCTGTTTCTGGGTCTGCATGCTGTTCCAGGGAACTTAAAAAGAGGGGCAGCTCATGAAGTCTATGGGGGATGCGTGAAGAA
ACGCTCTCTGAAGTATGTTCTTTCATTTTCTGGAGGCATTTCATCATCAGAAGTTATACGTTGGGTTTTAGTGTGTTTCTC
CATGCTTCTCTCATAGACTTCAATACAGCTGACACGACACCACAGCCTGGCACCAAAACAAGAGTCAGTGACGTAATGATC
CTTCTGTTTTATGTTCCTTGGGTCTGACTTCCTGCCTGGTTCTGTGCAGCTTGACGTGGCTTCTGTCCACTTCTGTCAAAA
ATAGATTATTGGCATATTGGTGGAACCACAAGGATTATCTACATTATCTGAAGAATGGCCGCAATCGGCTCCGGCAGCTGG
TCATGTCTGGAACGTAATGGCGACTTCCAAGATGTTTCTATGTAACAAACCATCTGGCGCGTCATGTTGGACAATGACTGG
GTTACATCAACCCTCCTGAGAAAAGCAATTGCACCCAAGCAGTGGACAGTTCAGATACACAGTACATGCAGCTTGAGTGTA
CCTTGTCATTGACGAGCAGCTCAGCGGGGGTATGCAGGCCCTGCAGCAGCACCAGTTCGTTAGGCTGCGCAGGAACAGCGT
AAGACAGAGGCGTGCCGATCCCGCCCTCCGCGGGCCGCAGCCACAGGCAGACGGTGAAGGCCCGCAGTGTCGGGATGGAGT
GTTTCACAACAGCGTACATGTAGTTTGTCCGAGTTGGGAAGGACAGTCTGTAGCCCTGTGGGAAAGAATGTCCGGAGATAC
CTGAGGAAGAAGCAGAGAGAGAGCATGAACATGGAAACAAAGGACAGAGAGTGCAGAGAGAAATCACTGCAGTGATTCGGC
AAAAGGATCAGATGGATCTGTACTTTCTTCACATCAAAACATCTGATCTGTGTTTTTGAAGCAGGGCATTGATTTCATAGT
CTTTCTGATCTTTTTTTTTTTTACATAAGTTAAAACATAATTTATTCATCATAAAGAAACCTCATGGATTGATTTGATCGA
GATTAATAGAAAAATGAAACATCTGCTGTGGTTTTTATGCCTGATTATTTCTCCTCATTAGTGAGATGAGTCATCACAGTC
CATTATTACAATTTCATATTAACCCGCACTTAAACACAAATCCTGTTTTATTTCTTATCAGTTGTATCTGTTAACACCCAC
CTTCCTCCAGTCCTGAGATTCGGTGGTGGAGTTTATTGATGCCTCTGTCGATTTCCTGTCTGTGTTTCTCTGTTTCCAGCC
TCAAGGCTTTTCTCTCTTTGTCCAGCAGCTCAACCTTCCTCTCCAGCTCTCCCTCTAAATCTTCCATCCTCCAGGGCCTGT
CAGCCCCAGCTCGGGGCCCAGGATCGGCCATCCTCTCTGGGCCGCCAGACGTCCCGCCTTCCTCTGGTACTCCTGAGGTGG
TGCTGTCCGTCTGGTTGTGAGGAAATGGGCCAATGTCTGACTGTGGAGGAGGGAGACAGCGGAAGAAAACTGGTTCACTTT
GTGGATTTTACAGCCTTTCAGAAAGTATCGCTAAGTACAGTTAAAGCAACATTATGTAGGAATTGGTATTTTGTGCGATTT
GGTGCCCACATGGTTTCTAAGGGCTACACCACTGTCATAAATACAAATCCCAGGTGTCTGTAACAGTATTGTCAGATGTAA
TGTCACATGAGCTCACTCTAAGAAATTTTAATCAGAAGAGAAAGATCTAAATAAACAAACTCTCTTTGTTTTCATAACTGA
ATAAACAAACTGACCTTAAAGGACAGGACAGTTTCATACTGTTTAACTTTGTTTATATGTGGCGGACCCTGCCACCTTTCT
AGCTTTAAACAGTGTCCTGGGGACCTTTTTTTTCCTCTGAGAACAGCTTGTTAATTCAGTTATGGAAAAGATAAATATTTC
TGAGTTTGTATCATTACCTCATTAATATTGTAAATTTTAAAATTCTGAGTTTGAATTTCTTCTCCAAAACTACATAGTGCC
CCTTTGATATGCTAAAACTTCCACTCCAGCTACATTTGCTGTAAAAAGTCATGATCACAGTTGACATCCACGGCTATATTT
GTCTCTTTTCTTACTCGATAGTCCCTTTTAGGTTTTCTGGGCACGTATGAGTGCTTTATGAAATAATGGCCGTTCAATTTT
GAATGAGGACAAGTGAGTTATCCGAAAGGCTTTATGTGCAGTATTGGATATTTATGTGCGATAGTGGATATTGGCAGCCAG
ATCCCTGTTTTTTCTGTAAACAACATCAGTTTGAATTTCTTCTCCAAAACTGCATAGTGCCGCTTTAAGGTGAAATAATTT
ATAAATGTTGCTTTCGGGTAAAAGAGTTTACTTACTATTTAATGGAAACGAACGTCCCAGTTAAAGCAGTGATTCTCAACC
TTTTCTTGGTTGCATGCCGATGTAAAGAGAAGTTATTAGACCCTATTAATAATTGATTTTGCTGAAACATTACATCACGCC
TGGTGATGGATCTAGGGCCTGATTTAATAAGGTTTCACTGTCCTTTAGTCAATGTAATGTTTTGTCCTTAAAGGAATAGTT
CAACATTTCTCCAAGGACTATTTCTTAGCTGGGGGCAGTCACTGTGAGCTTGCTAGTCTTATCTAATAGTTTTGCACAAGA
GTCCCTATGTTGGGAAACAATGTTAATTATTAGTTATACATTTTGAATTTTGGATGGATTGCTCAAATGAGATATGGTGTG
TTAATTAAAGAGCTTTAGAGGTGCTGGTAGGTTGATTGTCTGTTACCTTCAGGCTGTTTTCTGCTAAGCTCAGATAACTTG
ATGCCAGCTATAGCTACATATTGAACGGACAATGCGAAAGTCATATCACGCTTCTCATCTACCTGCCAGCAAGAATATGAA
TAAGTATATTCATATATGGTATTTGGCGGACCCTGCCACCTTTCTAGCTTCAAACAGTGTTCTGGGGACCTTATTTTCCTC
TGAGAACAGCTTGTTTATCCAGTTATGGAAAAAATAAGTACTTTTTCTCCTTTATATTGTCTCTTCAAGCTTAAAACATTT
AACTGCTGCATACATAGAGCATTAGCATCCTTTTGCAACATTTCCTAGAATCGATTCTGTAGTATTCTGGGTTTATATTTG
TGGATTTCGCCATGTTTTCACATTTCTGTGGAATCTGTGTGAGAAATAGTGCGGCATGATTAAAGCAGGATCAGTACTTTC
AAGCTCTTCTTTTGAATTCCACAGAGGGAATGATAAAAAACGTCTGTATCCAAGTGTCACTCATGAAGTTTTCGGTATGAA
CACATCTCCTTAAAGCTGAGCCATCTCATGTCCATAGGGATTTGTTTCAATTAAGTATAACGCAATGTATAATTGCAAGCC
AGAAAAGGCCGCCAAGATTAAAAAATATATTTCAGTCAACAACAGATTGCCAGGCCCTGCTGCATAATGGTTCACTGAAAT
AACCAGTCATACCACAGGGGAGGGAATACAGACTCAACAAGATTCCTAAAACCTGCCTGGTCCTGTGTTTCCACCAAGGAT
ATTGTTTAATGGGATGCAGTAGAGATGACACCCCGAGGGAATTTATACAGAAAATACAAACCCATAAGCCATACATTGTGG
ACTGTGCTTACCTCTATTGTATGAGATGATGCTGGGGCTGTGCAGCTTCCTATACATAATAACCTGACTTGATAACACATG
GTGTAAAACAGGACAGCAGAGGACACACGATGGAGTAACATGTATGTTTTCCAGCAGACTTAACAGTCTTTTATCCTGTAA
TGTGGTTGCATTTTGCAGTAAAATCAACAGCAGAATTATTGTAAAATGCCATTATCGTCACATTTGATGTAATAGCTGGAA
ATGATAGGTTTGCACCTCGCTACAGACTGCACTTACGCATCTGCTTAAATCATGCGTATGCTAATCGAGAGCCTTGAAAAA
AATACAATCCTGTGAGCCTTGGACTGATAGGAAAATATATTTGACACTTGTGTTATCGTGCCTGGGGTTAGGCCCCGAGGC

AGGTATTTACATGTGCAGGCGTCAAACATTTGTAAATAAAACAACAATTACAGTTTCCTCCAATACATGAATGTGTGCACA
AAAAAATCTAGAGAGGCATAAGTGTGGTGAAAGGCATCATTATGTGCTCAGTAGTACATGCCCCAACTTTAAATAAGCAAA
CTGAATGTGTCAGTTTGTTGGTTGGTCCATCACTTTGTCCAGACTTAGATATCTCAATAACTAACAGATGGATTGCCGCGA
GAGGATGAATGATAAAGACTTTGGTGTTTGCCCTTCCATGTAGTGCTAGCAGCAGGTCAAAAGAAAAAGAAAAAAGAATGC
AACATTTACTTTTATAGACATTCGTGGCTTTAAGATGATGAATTCTGAAGACGTGGTGATCCCCTGACCTTTCTTCTAGCG
CCACCATGAGGTTGACGTTTGTGGGTTAGTCTAGGGAAATGCGATAACATTGGTGATCCTTTAAAATTGTGTCTACCTATC
TAGTTGTAAGGTATTATTCAGTGAATGTTAGAATGCTGTCACGCTTTACTAAGATGGAGAACATTGTCAACAATACCTGCT
TAACTGCTAAACATCAGCATGTTAGCATTATCAATATGAGCTTGTTTTCATGCAATGCTGCATGAAAACAAGAGCATCTGT
TCTTTCATTTGATCAATCAAAATTTCATAATAGTTCAACAGGAAATTAGCCACATAGTAAAGATTAGAGCCGGGGTTTCCA
AACATGTTCATGTCAAAGATCTCCATAGTAGATACCGTTGTCTGAAACAATAACATCGGAGTATAACTGCATGGCTAACTG
AGGTAACTACCTAATGGCAGCTACAGTTAGCAGCAGTTTACTCTGGTGCTATGCTGCTTCCTATTGGTTTTGAGTATGAAT
TTGACAGGTGGCCAAATAATGCATTTTTAATTAAATATGACACAAAAAAACTTCATAGACAATCAATATTTAAGATACTTT
CGCATTAAATTGCAATCAATCAAAAAAGCCAACTTCAAAATGTGATTAAAGTTAAAATATTATCTTCCTCCTTTCGCTGGG
GACCCACTTAAGGACCCTGTGCCACTGAGCTCGAAGACATTTGTTCCTCACACTCATTTCCAGCACAGCCTGTACTGAAGA
TAAAGCACAGAGCGAAGGATCAACACACTGCAGCTGGATCAGCACGATATTACGTGGCTCTTACTACCATTGATCCTCTGG
TTATGATGGAAATCTACTGATGCTGCAGGAAAATCACGGCTCAAACTGCTATCGGAGCCCGTGGTACTGGGAGGCAAATGG
AAAACCAGCACATTTGCACAAATGCAGCCTGTCCTAGACATTTTTTCTCCTCTGACATGTGAAGAAGGGTTGGCGCTATAG
GATTTCTGGCTCTAGAAGCAAACATGGACATCTAGCAAGTGCTATCAATAGCTGCTGGAGTGTAGTCATGAAAGCTGTGAG
GGTTATGAATTTAACACAGTTATTTGACCTGACTAGTATTCCCTGATGGGTGCTCTTCTTGTAGATTCACCAGCGCAGAGG
ATCTGATGTAAACACCACACTGTGAAATAGGCCAAGTACCTGCTGTACACAGAAACGTGACTTGGATGGTAAAAGAAAGAG
AGTGGGGGTGAGAAACAACAGCTCCAGTATTATGTCACCCTGAAAACCTGTGAAATCCAACTCGGATCTTATTCTGTCAGA
GGAAAACGTGAGCACACATGGTTCCTGAAAAGAGCCGCTTTTTTATCTGACAGTGTCGATATGGAGAGGAGACAAAGAGAG
AGTGCATAATCCTCAAAGAAAATGACCCACACTGACATGTCCTACATAAACTAAAGCAGAGTGACAAGCGCTATACTGCAG
AGAAGCACATTTACTCTGCTTTCAACAAATATAGGCGACTCATAAATTATGGATGGGATTGCTTCAGCCTAAAATGAGGTT
GTACATCATCGGAGTCTGGTCAGAAAAGGGCATGTTTCATGTTACTCTGCTCTATCGTCCCTCCTCCTCCTCCTCCTCCTT
TATCACCCCCTCCTCACTTTATCTTTATCCTCATCTCCTCTCTCCCTCCCCCCCTCTCCTCCCCCCCTTCACTGTTTCTCT
TTCCATCGCTCTCTTTTCTTTTCCACCCTAATTTCTCATTCTGTTTCCCTGGCATGATAAATTAGCCGCCTCAGCCAGGGA
CGAACAGATGACGGGTTTTTGATTATGCAAAACCTCTAAATGTTGATTTTATCGGCTGTTAGCTGCAATATCATTTCCAGA
TCGTATCATGTGATCCCTGTAATTGATTAGAGTGCAAGAATGCAGACAAAGCAGCCATGTGTGGAAGCTTTTCTCTTTTTT
TAATGCTTGCAAATGAGTGAATGGCTTCAGCTGATGTTGTTGTTGACAAAAATGGGTATCTGGCTGCCAAAATCTGATACC
ACACATAAAGCCTTTTGGATAACTCACTTGTTCTCATTCAAAATTGAACAGCCTTGCTTCCATAAAGCACTCATGCGTGCT
CAGAAAACCTAAAAGGGACTATCGAGTAAGAAAAGAGACGAATATAGCCGTTAATGTCAACTCTGAACATGACTTTCCACA
GCAAAGTGCAGCTGGAGTGGAGATTATAGCACATTAAAGGGGCACTGTGTAGTTTTGTAGAAGAATTTGAATATTTACATT
ATTAATGAGGTAATAATACAAACTCCATGACTGAAAATAAGGTCCCCAGAACACTGTTTGAAGCTGGAAAGGTGGCAGGGT
CCGCCACAAGTATAAAGGAAAACAGTATGAAACTACATGGTGCACCTTTAATATTAAAAGCTTTCTATCAGAACATGAACA
TCTTTCTTCAACAGGGAGATTCTTCTTTATTCCTTTAAAATCTTATCATGTGGGACCAGAGATTCCTCCTCATGTCTGGTT
TTCTTTCTCTACATACCTCCAGTTTCTCGATGCGGTCTTTGAGCTGAGTGATCGCCTGCTCCAGCTCATCCACAGCCCGCA
CGGTGAGCAGGTGGACACTGTCGGTTGTCGAAGCCGGGCTGTCCCGCACCATGATCCGCTCCAGGTGCGTCTCCTCTCCCG
CGCTCTTGCCCCCCAGCAGCCCCGCTGCGCCCCCGCGTCTGTCCGCGCCGCTGCTCCTGCCGTCCAGCCCCTTCTCGCACT
CGGTTAGTTTCCCCGTCAGCTCCCTGATGGTCCGCTGGTCCGTTAAGATCTGGTCCTTCTGCTGCAGGACCGTCTGCCGGA
GCTCCTCCGCGGTGGTGCGGAGGTAGCCCCAGTCCTCCCCCGCGTACAGCGACGGGTCCTCCGCTTGTTGCTGAAAGTTTT
TCGGGTTGCACTCCCCGGCGGGGATCGGCGTGCAGATGAGCCGGCTGACGGATGGGTCGTGCCCGGTGAGTCCAGTGACCC
CGTCCAGCCCGCCGAGCCCGATGTTGAAGGTTGGTGCTTCTGAATCGGGCGTTTCAGAACCATGGAGAGCGCCCAGAGATC
CGGCCCGGGCCACGGAGCCCCTTGAAGCGGGGGTCTGCGCCGCCGCGTCCGGGTACAGCGAGTGGTTGTCGGTCGCTGGCT
GCTGTTGCGCGGCGGCAGCCCTGGAGGATCCGGTGTGGACTGCCGCGATGATGCAGATAACCGCTCCGATGAAGGCCACCA
TCCCGGCGGCCAAGATGATAACAATGAACTTCAGGTTGGCGGCGGGGGCGATCGATTATTAGAAAAATCTAACAGCTGATA
ACACTCAGATGAGAAGAAACTAAAAAGCTGCAAAACAACAAAAGTGTCCGATTATTTTCACGCATGTAAAAGAAGAAGAAA
GAAAAAACTCTCCACCTCCTCAGTTTGACTCTTTCCCACAAGAAGAGGCGATTACAAGAGGAGAGTTCCGCACCATAATTG
AGTGATCATGGAGTCAACACACCAGGAGCAGCCAGCTTCTCTGCTCCACCTCCTCCTCCTGTTCAGCGCGCATGTCCTCCC
CATCAGGCAGAACCCAGCCTGTTAACACTGAAGCAGCAGAGTGAGGAGGAGAGAGGAGGAGAGAGGGAGAGAAGAGGTGGA
GAGCTGCTCCCTCCATGCAGTCTGATAATGAAGCACTTTTTGTACATTTTGTCTCCCTCCTGTCGCCTACAGTTAACTTTC
CCTGCAGTGTTTAAACAGTCATCTGTGCTTGTGGACAGCAGCTGAAAAATAAAAAATGCTGCATAAAAATCCCATCCTCAG
TCACGAGGATGGTGCAACATGTGCTGAAAAAGGCAGGTAGCCTCACAGTAAAGGGTCTTTATTCCCAGGCTTTCCTTGAAG
ATCTTTGCTGCATGCATAACAGTCTGCAAATGAGTTTTTCCCTCCCCGAGGCTTAATAATTTCTTGCTCAGCGCCTACTGT
AATGTGTTGTGCGGGCAGCCATAAGTCAGCCTCAAGGCTCCCTATGGACCGGCTCTACGGCAGCAGAGATGACAGAATGAT

GGCTCTGGTGGGGGTTTGCAGAGCATGATACAGACCTGGTGCTATTAGCGGGTTTCATTAAACCAGGCTCGCCAGTAATGA
TGCATGTTAAAGGAGCACTATGTAGTTTTGAAGAGGGGAACTGTTGTTTTTTATGCCTAAACAAACTAAATAAACAAACTC
TCTTTGTTTTCATGACTGAATAAACAAACTGACCTTAAAGGACAACACAGTTTCATACTGTTTCACTTTGTTTACATGTGG
CGGACCCTGCCACCTTTCTAGCTTCAAACAGTATTGTGGGGACCTTATTTTCCTCCGAGAGCAGCTTGAAAAGCTACATAG
TGCACCTTTAAAACACTGAATTTGAATTTCTTCTCCAAAAAAACTACATAGTGCACCTTTAATCTGAGCGTAATGCGTATT
TACATGATCAAAACACCCGGAGACGAAGACAGGTGTGAGAAACTCTCCGCTGTGTGTATTAAATTAGTGTGTGATGTGCAT
TCTCACACAAGTATTAAGGGGGATTTAAACTAAGACCTCTCAAAGTCGCAGCCGCTGAAAGCTCCAGTATGAGACAGCTGA
GGGGAAACAGCGCGAGACATCTGGCATGTCTGCAGCACAAAATAGCTCTCAGTCTTCAGCCGACCACTGGAATAATAAAGC
AGGTTGGTAAGTACTCCTGGAGAGATGGCTGGGAAAAGTAAATACAAAAACAAAAGGAGAAACGTCTGAGCTGCACACATT
TGTATTCAGCCTCTACACTTGAAGTCACTGGTGTAATTTCTTGGCTGTAAATAGCTTTGCAGCAGCAGAGTGGGTTATATC
GCTACCTAATGTTGATTGGAGAATGCTTGCAGCTTTAAATGGTTTTTATTTTAATACTTTCGGATGATGAAAGTGTAAACA
CCGCCTCTGTACATGAATGCATTTGGGGATTCAAGTATGAGTTCTGTTTTCTTTGGCTGATTGGAGCTAAATTGTTCAACC
CCACAGCACAGTCAGTCCACGCACAGTGAAGTGAGTGAGCGATGATAAAATGTGTGCAGGTGCATTAAAAGAAGGGCAGTC
TGGGTGAAGTATTCTGGACACTGCTGCACAGTTAATGATCGAGATGTACTGCCGACGTGTCCATAAAAGCCTCCGTGGCCT
TCGAGTCTGAGTTCAGTTTATCATCCTAGTCACCCTTGACCTTTATGTGTGTTCTGAGCTCTGAGGTTTTCCTTTGTTGAG
AATGTTTCATATTCCTTAAGGCGATTTTTCCACAGGATTTAACTATAATCTGTGCTGGGGGATTTGGTGAGAGCTGCTCAG
TTACTTGAAAGTCTAAAGATTTTGCTTCTCCCACCAACAAATGTACTTTTCTTTGTGGGTTGATGTTACCTCTTTACTGAG
CTGTGAAACAGATGAAATGTGGCAGTGAAAGTAATCCTTTTAAACAAAGCTGAGTGAAAACAAATGCGTGATAATGCCTCA
TTTCAAGCTCTTTAGGATAATTGTTTTGTTTATTACTGTTGTTTTTCCTTGTTCTGTAATATTTAAAGGGCCTCTACATCA
CAATTTGTAGCCATTTACACAAATTAAAGGTGCAATACGTAAGAATTTGCCACCTCGTCAAATTCATACTCAAGACATACG
GGGGGCAGCATATCACCAGAGTATCCGCTAACTGCTGCTAACTGTAGCAGCCCTTAGCTGGTTAGCTCAGTGCAGCTAGCG
GTTCGGACTGGGACCCGGGGCTGGAGGGTAGCTTTGGACCAGGATGGGCCGGGGCTAGCTGGTTAGCATGCTAACTTCCGT
AGACATCACTGCAACATAGTACATAGACATCATAATGTCAAAATTGCTATTTATTTTTGAAAATTTTTGAACATTTTCAGC
TAAAGTTCGTATATATTTCACCTTAAATCAGTTATTTGTTGCCCATATGTGAGTGGATTGTAACGTGACATGAAGATGAGT
CCTTCCCCGTATGTTATCCAGTGCCTCGTCTCAGTACCTCTCTCTGCTCCGCTAACATAGAGCAACAGTAGCTAACGTTAG
CTTAGAAATGGAGTCCGCTAACATAAACAAACACAGAGCCCCTCTAATAGACAGTTTAAAGGTCCACTGTGACAGGAGAAA
TATATCAAAGAGCTGCACATGGCCTGCAGCAGTGAAAAGTTGACCAGAGAACTAGCTAAGATCTGGAGATGGTCCCTGGGT
GCCGTATGTTGGCTGCTACACTGTACATTGTATGATTGTATGTGATCAGTTAATAGCGTATGATAAATTGTTACAGATTAA
CAGTATATAGTTAAAGGGACAGTTCAACCCCAAATCAAAAATAACCTTCATGTTTTTCCTCTTACCTGTTGTGCTATTCAT
CCATCTAGATTGTTTTGGTGTGAGTTGCTGAGTTTGGGAGATATCAGCTGTAGAGACGTCTGCCTTCTCTTGAATATAATG
GAACTAGATTGCATTGTGGGGCTCAAAGCACCAAAAAAAACTCAACAGCACTGTCTCTTACCAGAAGTCATGACTCAGTGT
AATCCACAGACTTTGTTGTGAGCATTTTCATGTTGGAACTATTTTATTTATAACAATTTTCTTTTCCTGTTAACCATATCA
GCAGGTAGAGGGAGACTTAGCTTGCTAATGTTGCAGCTCAGCCGCGGAGGACACGTTATGCTTTAAATTCAGCTATATCAT
GACCACGAGTCTCGAGTTACTACATGAAACTGCTCACAACAAGGCCTGTGGATTATCTTGAGTAACAGGGTCATGATGGCT
GATATCTCCAAAACTCTGCAGCTCACACAAAAACAATCTAGATGGATAAATAGCACCACAGGTAAGAGGAAAAATATGTTT
TTTAATTTTGGGTTTAGTAGTTATTGTTTACTTGCTACCACTTGTGGCCCATTCTCGAAAAAAAGCCTCTAAAGTGCCCTC
TTGGATGCCCCTGAGGTAGATAAAACATGATAAATGTCCTCTAGGGTAAAAATGTATCAAGACTTCCTGTGTGCTAATGCC
CCACCACATATGCTGCATAATAAATCCTTCTACTTCTATTAATAATATTGTTGTACTTCTACTAGCTTTTGAATGCATGAC
TTTTACCTGAAATGCAGTATTTTTTTTCACTATTTTTACTAAATTAAAGGATCTGACTCCTTCCAGCTCTGTCTGTGATCG
AATAATATTTTACAATCAATAATGCTCACTATGTTGCACTAAACATATATATATAAGCTTCTTGTGTAAGAAGGTGAAGGC
ATGCAGTGGGGATGTTTGTCCTCACACCTGATATACTGACCCAGAGCTGGCAGAATTGTATTAAAAGCAAAGAAAAGAAGA
AAAGAGGGAGCAAGAAGAGATTAAAATAGGTGTGTGTTATGAAGCTGTTTAAAATTCCAGTCATGTCTCCAGTTAGTTAAT
GGTGCCATCTAGTGTTCAAGGCTCCCTCTCTCTCTCTCTCTCTCTCTCTCTCTCTCTCTCTCTCTCTCTCTCTCTCACTCT
GTTATCTATTCATCTATAAACAATTAATCTGGGACTTCAGAGGTTTCACAGTCTATTTTGTTATTTCCTGCCTCGCATACA
TGCATCGATTAGACTGAAATACAATCATTGCCCTCACCACACCACCCTACAGTGTCTTACATGCATTCACACGGGGAATAA
ATCACCGTCCACACGGACCACACTCCACTCTCCTGGGAGTTCACGGTGAGTTCACTGACTCGGACGTGTCAGAGCAAAGAA
AAATGCACAGTTTGATAATAAATCCATCAGGTGTGAGTCGAGTGCAGCTCACAAGAGTGTGCAGTTCAGCTCGGCAACAGG
GGGTCGGGTGCCAGGCCCGCTGCCAAAATATGATGGCTGAAGCAAAGAAAAGAAATCAATAACTTTCCCTTCACTCATGGC
AGGTTTTGATATAACACGTCAGAGCTCCTTTTTTTGGGGTATTTCATGCTTCTTTTTTGCCTTGTTCAGTCTGTGCAAGTA
GGATTTTAACATCTAAGGTGATTAAAGTATCAGGATTAAGGGATTTTTAGCACATATGACCACTTAATTCTACATTAACCA
ATCAAAACCGAGGAATATTAGTGATATTACAGTATTAAAGGATTGGGGTTGAGTAAATCAGATTTTAAAATATGCTCATAT
TTAGCAGTCAGTTTACAATCTGCACAACATGACACACTCACACATCTTTAGCTCCTCCATTTGAATGAGGAATTAGCAGTT
ACTCTGGTGATACGCTGCACCCTATTTGTTTTTTGAGCATGAATTCAACAGGTGGTCGATTCTTACATATATTCTTACATA
TATTCTTACATATTGCACCTTTAACAGGGGAAAAAAGAGAAAGAAATCCCTGAAGGAGCAACAGAGGATTGATCTCTCTCA
CAGGATGGACTGAAATGTGATAAAAGTAATGTGTACCAGAGTTGACTGAAGTAACAATGGTAGAATTATGATATGGAAAAA

CAGAACGACGGTATTATGTGGATTGTTCAGATATATGAAGAATAGGTTTCTACGAAGACGTGAAGCAACTGCAAATAACCT
CTCAGAAAGAGGACAGAAACACACAAGGGAGAGAAAAAAGCTCATTCAAGGACATCATTCACAAAGAGACAATATTCTTTC
TTTTTAACACAGATAGTGCAACAAACTGACACTGGTTTCAATCGCTTTTCATTTCCATGTGCTTAGAAGACACCTTTTTCA
CTTTCGCTTTCCTCAGAATTATTGTTAACTCCATGACGACCTTTGCAGATTATTTTCAGGCATTTAAGTGACGACAACATA
AATACTAATGAATATTTTGTTATATAACACGTTGTTCAGAATCAGTTGGACACACTCCAGATTTCTCTGTGACCCACCTTT
GGGTGCAGAACTGATATGAAATGATAAGACTGGTGATATTCTATATTTTTAGTGATTAAATGCCATGAAAAGACCCAAACC
AACAATGAAGTGACTCTACAGTATTGTTGACGAAGGTTCTCAGGTAGTTCTAAGTGCTGTATTGTAGGCAACTGGACGTGT
TCAGTTTCCTGAAGACGTGATACGTTTACAGAGTCGTTGGGGCCACTTGAGGGTCCTTGATCCAACTGGCCTTCATGTGCG
TTGCTAGGGCTAGGTGAGCCCAGGTGTGAATGGTTGTTAAGCTGTCTGGGGAGGGAACTCAGTACTTCATTGTAGGTGAGT
GATAAAAGCCTGCAAGAACTGAAGGAGCCTCTTGGATGAGAAGTGAAACGTGTTCAAGAAACTGAAACAAGTCCAGTTGCC
TACGATACAGCGTATATTTGCATCCGAAACCTGATATACAGTGGTTAATACCTCTGAGACAAAGTCTAGCAACTATTTATT
ACATCTAATCTGTGAGTCACACCTTTGCACCGAGAGACGACTTCCTTACGATACAGCAGAACCATAAACTTAAATCATCCT
GTTGCTGTAAATGCTGAAAGGTGCATCAAAACCGGAAAATGGTCTCCAACATATAATCCCTTTACTCCTGTTTAAGTAACG
TCTGCAACCCAATATAGCATTAATAAATACCTTATGAGTTTATAGTATTTGTGTAGTTATAACAGGCCAAGAGCAGCATGT
CCTCTAATCTTAAATTAAAAAACAGTTAATAAGCTTTAAATCTCGTAGATGCAAAAAGCATAGTTAACCGTTAATAGCTTA
AATTCAGTTAACAGCTAATAAATGCACAATAATGTATGTTTTAATCTGAATAAAGCAACAAAAATGTTGAATAAACCAACA
AAAAATAGCTGATTTTAAACCGAATGGGTCCAGCATGATGTACTGCTCTAAAGTTAGCTAGCTAGCATTAGCAACATTTAC
GTTAGGTGGCGTTGGCGACGATGGCGTTAGCTAGCCCTAGCAACGTTAGAGTTAGCTAGCTCTTGTGATGTCAGTGTTAGC
TAGCTAGCTTTAGCAACGTTAGCCAGTGCACAAACTGGCAACCACTTGAGGGGGGAGATGATTCGAACAACAGCGGCCTTT
TAACATCAATGCAATGTGAGTGAGTGTTATCAATAACACCTTTAAAGCAAAAAAGTTTCTACTGACAGTTTCAGTTAAACT
CAAGAAATGCATTTGTAATAAACAATTAAACTCACAAGGCTTTTATTAGCGTTCTTAAGCAGCTTATAAACTGTATATAAA
CTGTAACATATATAAATATTACATTATATTCACTGACATTAGAGAGTTAAAAGAGATCTCTGTTGTCGTCTTGAATAAAAC
ACACAATGTGGCACAAACACACACGCACACGCCCAGACAGTCAAGGTTTACAGCATCTGTCAGTGTGTTTTATGGTTGAGC
CATGTTTAATTATTTACTCTGGTCTCTCTCAGGCCACAGCGGTGTCGATTACCAGCACACTGAGCTGCTGCTCCCTCTCAA
CTACACGTCAGCTTCAATACGATTAACTCTCCTTTCTCACAGAGGCTTTGGCACAGTTGCTGGATGATAAAAAAGCTGCAG
ACCCTGTCAAGGTTTCAGTGACAGTTGCACTGTTATGGATATAAACATGTCGGGGATGAATAGTGATCAGCTGCATCGCTC
CACCTTGGGGATTTACTCGGTAGGTAACAGGTACTGTGTGTGGGGTAGTGGCTCAAATTAGACGACAAACTTGCATATCGT
TTCTGGCGAGCAGACATGATGTGAATCCACACAGTCAGTTTTGAATTCAAGGAGTGTTTCCTTTGCTTGGTAACACCAGAG
ATTTGAGTCTGAGGCTGTCTGTGCTCCAGCGTTGGGCGGGAGAGTTTGTGCTCATAAATACAGATTGAATTAAATAAGTTT
TATTGATTCTCCTTAAGGTGGGTCTAAAAGTTTGTCTGACTTTCAAATATGTGTCTGTGTGTGTGTGTGTTTCCCAAACAG
GACTTAATGGATGACTTCAACCCGAGCCTACAGAAACTGGTTTTACTTGGTAGCAGCTATATGCAGGCTTTCCAAGGTAAG
ACATAAAGAAAGAAACAAAAAAAGAAAGACAATCCGCTGATGACAATTTCTCACTTCTGAAATTCACACACCTTCCTTTAT
AAGTGCTATAAACTAAACAAACTATAATGGCGGTACACAGGATATGGGAGCTGTCTATTTCACTCCACCTAATTCCAAAGT
CCAAACGCTTTGGGAAAACCTTCCTGGTGCAGTTAGAGCCTTGATAATGGGCCCGTAAACACAGCCTGCAGAGGTTATGAG
TACATTGAGAACAGACAAACAACGGCCCAGAAAGATATTTGATTTCCAAAGACTGAGGCAGGGATTTAGGATATTGAAAAC
AGTCTGGGCCTCATGTTTTTTAATTCATTTCTTTGCTTTATTTTTGAATGTGCTTCTGTGAGAAGAGGTCATTTCAGAGCA
TGGATTAAACTTTTAATTTGAATCTGAACCTACAGTCTACATTTGATGTCTTAATTATCCTCCTCGTAATCACCTCTAATC
CCTGCGCGATGCGTTCGGTGTGAGCGGCCTTTAAAGTGCTGAGATGGAAATGAAATTCAGCCACAGAAGCAGTTTCACATG
GTGAGCCTTTCACTGTAGGTGCTTGTTTAATATATGCACGAGCACTTATGCGACGTCATCAACAACTAATTCACAGTCGCA
CTGCTGCACCAAAAGCCCTGCAGACAATAAAATACAGATGATGATGTCTCTGGTTGCCACCTGCACTGAGACTTTAAATTC
CTCCTGCGTGCTCTGTGGAGCCGCGACTTCACCACGTTAATGAGGTCAAGACCAAGAACTGATCGGACTTAAGAGTCCATT
TAGATTCTACTGATGCAGCTTCTCCTTATAAATGAAGAAGCAGTGGATGCTGCCCATACTGCCCCTATTCAGTGTTTTTAA
AGAGCTATGTGTTTGTCAGTGAAATCCATAGTCTCACAGCCTCGGGGAGTGTGTGTTTGATTGGTTAGCATTGGTTGTAGC
CCCATCTTGACAAAAACAAGGCAACGTTGTCTCCTCTTTTTACTGCTGAAAATGCAGCTAAAGCTCACTCAAATAAAAACA
AAAACCCCAGGCAACAAACTACTTCAATAAGATGTAGGTTAGTTCATCAAAACGTTTACGATTCAAGTACAGGGAACAGCT
ATGTGGGAAAAGTAAGTCAATTAAGTTTGAGCTGAGGCTAACCATGGCTCTGTCACTATTCAGATTTAATGTTGATGACGG
TTCTCAGTCATCTGGGTCATGGTAGTTCTAAGTGCTGTATCGTAGGCAACTGGACGGGTTTCAGTTTGTTGAAGACGTTCC
AGCTCTCATCCAAGAGACTTCTTCACTTCTAATTAACTGGAGGAGTGACCTTACAGAGTCGTTAAGGACACGTGTGAGCTC
TTTATACGTCTTTTCTGGACATTGTATAGATGTTTATATGTAGTTTGTCGACTAATTCTGGCTGTCCGTGGACATCCAGAT
CATGGCCTGGCCCGACGGACATGATATCAACCTGTTTTGGACGTCCACAGACGTTGCCTGCTCAGTGGGGAGGTTCAGCTT
TTACTGTTTGGATTGTAGTTTTGTTGTGACTGCAAATTCAACGTACTGCTTTTTTAATTATACATTTCTGTGACGTCAAAG
AGGCCAATAAAAAGTGATTAATTCATCTAAGTTCCTACATATTTTAGCATAGAACCTGTTTAAAAGTTTTGTGTGTTAAAA
AAGATGTGAGTAGGTCAGATTCTAGTCTTAATCTTTTGACTAGATACTGTACTGCATTTTGCCGTTGACATTATAAAAACA
AAACCAATATCAACAGCAGGCAAAATGCACTGTTATAATTGAGAGTGAAGAGGGACTTTATGGTGATTGCTTATCAAGAAG
AATGTGATTACACTGTGATTTTATGCAGCTGTTATCCAAGTAACATATTTCCAAATGATTGCAGAGAGACATTTGCGTGT

GTTGTAATTTGAATTATTGAATCGTTTCCATTCCATTTCCGCTGTTGTAATTAATCACTCTGTTTACCTGCCTCTGTACTT
GTAATCTGGGGAAATTAATTGTGCCAAATCAATTTGCATTTCAAATAACTGTTGTGCTTGTGTGTGTTTTAGCTCTCGCTG
TAACAAGCGAGGCCTACTTCAGTGCACTTTCAAGGATCGGAGAGAAGGCTTCCCACACCGTGTCCTCACGCTCCCTGGGTA
TGTATGAACACATGCACAGTCAGCTGGCTCGTGCGATGTCGTGATTACTAAGTGGAAACACAATGAGGTTATTTCCATATC
TACAATTCAGGCAGTGTTTTGTCTTTTGGCTAATTAAACAGTGTTCTTCTGTGTGGCGAAGACCAAAGTTTGCCCTCAGGC
AGCGCTTTATTACCCCTTTCACAAATACCAAGAGCATGTGGAACGTGTAGGTGTGCGTCATTAGTGTACTCTTTGATGTAC
AGGTTTGTAAGACATACTGTGATACATTATTGAATGAATGTATTTTATGGTGTAACATCCACAGCTCATCAAGACTTGTAG
CTCAGACTCAGATAACATTTGTGTTGGGCTCGAGAATGAGGCAGAACCGTCTTATTAGATTTCTGCTCATGTCAAGAGGCA
GCTTCAAAGTCAGATTGCAGCACAGTGTTCGTTTGAGTCTGTTTTTATATGAGCTCAAGCCAAGAGTGTTGGCAGAGTTGG
GTATCAGATGCGAGCAGACATCATGAATAATAAACGATGACAGGCAGCGGGCCGTGCTCCGCTCACTTAACCCAGCCACTC
TGAAAGGGAGTCGTGTTCAGTTTGGTAGCTGGAGTTGAAGATATTGTCCAAACTGGATCATTAGTGTACTCACGCTGTGAG
GAAATGAATCCCAGCATGCATTTCAAGCACGGTGTCAAGTCAAACTAAACGAGTGCAACGTTTTCAGAATATGATGTCATG
GCGTGGTGAATCCTGATGGTTAAACTGCTTTGGGGGTCGTTTTGTGCTTTTATCAAATGTTTAGATCCCTCACAATGCAGG
TTATTGAAAGCTAAATGCACTTTTATTAAAGGTAGAAGACAGCAGTTGGCCTAAAATACCATTTGGCCAATAATTCAATGA
TCCCTTCAAAGAAAATAATGAGACTCCAGACTCATCATTATCGGTCATTAACGTCAACATTTCAAAGGTTATTGTAAAGAT
GAATTTGCACAGTGGCCTGATAATGAAACTGGATTTATAAAAGTGATTAGCTGCAAAATTAGCTGTGCCTCATGTCAGGAA
GAAACATCACAGATGAAAGATTAACAATGAATCCCTGAAGAAAGTACAGCACAGTAGATTCTCCAGTAAACACAGCTTCAC
ATATCAGAGTCCAGAGTACAAATGAAACAATAAAATATTTAACTGTTAGCCCTAAAATGTCAACAGATTTACACATCAGAG
CTAGCATAGTGGCTGGCTTGATTAGCAGCAAACAAAATCACAAAAACAAACCCGATCAGACTGTGTAAACTCTGTAAACTG
GTGGACGGTAACAGAAAGAACCTGAGTTTCTGGTGAGTCCTGAGTTGAGTCAGAAGCTGAGCAGAAACACAGGCACAGTCC
CTCAAATGTTTGGTCCGTTTGTTGTTAGCTAGCTTACAGCAAACACCTGTGTAGTTGGTACAATGGCATGATTAAAGACAA
AAAGTCAATCAGAATCAGAATCATAAATACTTTATTGATAACTGAGGGGAAATTGCTTGCGTTACAGCTGCTCCTATTCAA
AGTCCCAGAAATATGCAGAAGAATAAAAACTATATATAAGCAAGAACAACAAAAAACAGAACAAAAATACAAGAAATGTAC
ATTCTACTAGAATATATCCATATATACAGTGTAATAAATAATGCTACAGTTGAAAATATATGGGTTATTGCACATTGTCTT
AATTATTGCACATTAGAGAGAAACAAAGCGTCCAGAACCCTCCTATTGACTCAGAGTGTCAGACACTCAGAGCGAGGAGTT
GAACAGTTTGATGGTCACAGGCAGGGATGATTTCCTGTGGCGCTCTGTGGTGCGTCTTGGTGGGATCAACCTAGTGCTGAA
TGTACTCCTGTGGCTGACCACCACGTCATGGAGTGGATGGGAGCCATTGTCCAAGTTGACATGTAACTTGGACAGCATCCT
CCTCTCTGACACCACCGTCAGAGAGTCCAGCTCCACTCCCACAACGTCACTGGCATTGCGGATCAGTCTGTTCAGTCTTTT
GGCGTCCTCTACCCTCAGCCTGCTTCCAACGCTGATGGGTAATTATCTTTCAGGGAAACTTGTACTGAGCTCTCTGACTCA
GATGAAATCCTAATGAGAAAAGAATTTGAAAAGGAGATAAACAGTTAATCTGACAAAAGGATTAATTTAACAAGAAGTGTA
TTTCCATTCACCTACTTTCATGCAATCAGGAAATGCTTAGACTTTAAAAGAAAAACCCTCAAAATGTGTTTTTTGTTTTTA
CTCTTGGAATCGATAAGAGCAGTGCAAGATAAGTGCAACAAGGCGGAGCTAAAACAGAATTTTTATGCAGACAAAAACATC
AGGAGTGTAACGTTCATCAAATAATCATCAATGTTCATCATTTTTTCACATCATTTGAGGTTTGACTGGTGGTCTTTCTTT
TATATCATCTTGCTGAGACCCTATCTTCTGCAAATGGTATAATGGCACCTGACTGGAGCATTAGCGCCACTAACTGCGTAT
CTCAATGAACCAACTTCCAATATTTGTAACTATTAATAGCAACATTTCAATAATCATTGAACAAATTGTTGTAATTTTCTG
ATTGATTAGAAACTTAACTAACAACTTATTTCAAGGTAAAACAACATATTTGATAACCTGTTAGCTTCCTTTAGACGACAG
CTAATGTTAGCATTCAACCACTTCCTAGCTAAAATTACTTGTTTGACAGTGTCACACAGTACAATAGGACAGGGAACAACA
CACATCTTGGACACATGGAAGCGATATACACTGGATGTAATCAAACAGTAATGTTAGCTAGGAAGTGGTTGAATGCTAATA
TTAGCTAATAGGTTATCAAATATCTTTTTTTTTACCTTGAAATATGGCCCGATGTTACTCCAGATTTTCAATATCAATCAT
CTATTTTGTTGCTGATCAATCGGGGAGCAGTGAAATGATAACAATTTGTCTACGTTTATACGACTTGCGACGCAGAAAACA
GCAGCAAAACATTATCCCAGCATCTGAACTTCCCACCCTGAGTGAGACTTTAGAGGAAGAGATGCTGAAGAGATGAAGTCA
CAGGTTTCCATTCCTGTTAAGATCATTGGTTAATGAGTACATAGAAATCAACAAAAGGAGAAGGTTATATGATGCTGCATG
ATGTCATTTTATTGTTTTCCAGTAATACATTAAGTGATTATAATGAAACATGTCGTGCTTTTTTCCTGTGCAGGAGATGTC
CTGATTCAGATCTCTGAGAGCCAAAGAAGACTCTCCTTGGAGCTGGAAGGACTCGTAAGTGACATCATCACTACACCTAAA
ACAGCTCTTGTGTATGAGTGTGTTATCCCTGTGTTTGACATGATGTTGTGTGTCTCTCTCCAGTTCCGCTCGTTCAGTGTG
GAGGTTCTTCAGGAGATGGACAATAACATTAGACTGGACAAAGATTACATATCAGTGAGTGAGCCCCAGAGCAGGGGCACC
ACAAGGTCAAGAGAGGGCTCTTACAGAGTGACAAGCTGTTATCTCGTAATTATAGGAAAATGAATAACTGCCGGGCATCAT
GAGAATAGTTATGGAGTGAGTTATCTGATTAATTAATATCTTGATAAATAGCTCACTTACCTCGCAGATGATAAAGAAGTA
ATAATCTGTCATTAAAACTGAAGAAGCCATTAGAAAGAACGGAAAAGACGTGACATGAATCGTGTGTCGTACAGGGCAGCA
GGAGGCGCTACGAGATGGAGGTCCACAGCCAGGCGACAGCTCTGGATAGGCAGCTGAGGAGAGGAGCCAACCAGGTACGAG
TTTCTGCACCCTGAGGGAAAATTCAGCACCATGTGCACGAGTGTGTCGTGTAAAATATGTAAATGTGCGCTGTTAAATATG
TGTGTCCTCAGGACGGCAGCGAGTATGTGCAGTTCCTCAGGAGGAGCCACGACGAGGCTCTGAAGGAGGAGGAGAGACGAC
ACCGCTTCCTGGCTGAGAAACACTGTGGTCTGATACAATCCATCGCCAAGCTCATGAATAAGGTATACAGCTCACCTCAGA
TAGCATTTGTACATTTAATATTTTATGGTCTACAGGTGCCAAATGGGTCAGGTTTGTGTTCTTACCACTCTTACTGCAATA
AGTGTCAGAAGGACTCTAACAGCTTATTCACAATAATGTGTGATGATTATGACTTTATCAGCCCCTGTAGAGGAGTTCCCC

TCCTTTATAATGAGGGTCAAACACAGATCAGCAACACAGTTTCTCCTGCAGAACTGATGTCTCATTAACAGGCTGTCTGTT
TGAACTCTGGACTTTCTGCTCACCTTTTATCACACCACTGTACCATCACAACTGAACATTTTACTGTTTTCATATGAGGCA
GTCACAAAGCCCTTTGAGACTGGACGTGTAGTTAAGGACGGATCTGACATGTTTGTTTAACTCTGTGCTCACGGGCTAAAA
AGCAGCCACGACAAAGATATTTGCCTCTCTGAAAAAAGTTTTCCCTTTTAATAGTTAACTTGATCTTAAATAAATCTACTC
TAGATAACTATGTAAATACAGGGCTTATTAAAAAAGAGGGGCGTGCCTGCACACTCACACCATACCACAGAAGTTTAACCT
GTTAAGAACTGACCTCTGTTTTTCGAAAATGACATGCGGAGAGTTTATCTCTGCAGCTACAAGGTCTATTTGAATAATCAA
GGTGTCATAAGATGCATTCAGATGTGTAAGTATAAGTATATATGTCACCGGGTCTGAGTAAATGAAGTTGGCACACAGCAC
GAATGAAAGATTTTATTTCCACCCTTAAAATATCTCTAGCAAACCAAAGCTAAACATCTGAACATCACCTCTGCCAACTTT
TATGCTAGTAAAGTCAATCAGGTCAAAATTAGAGAGGTTTTAGTACCATCTGAGTATTTGGGCACCATCTGTGCTATTTAA
AACCTCTCAGGTACCAAACTATATATTTCACTTACATGTTACCAAGTTGGGCTCTCAGCTCGGGCTTAACAGGTTAAAAAA
GACAACATTTGGATCCTGCTTGGATCTTCATCAGGTCGGACTGTAACCAGCCTGCTCATCTAATACAGGCTCATTCCAATA
GATGACATCAAAACATATAAGTATGAGTAAATATGTCAGTCAGTTATGACATATTAAGTCAAAGTGTTTAACTTTTTCTTG
TATTTGAGCATTGTTTGATGGCATCAATGAGCTTCCATACAAAAACCTTTTAATTCTCAGTTGTAAAGCTTTAACCTGTTT
AAACAGACTTGTGTCTCAGGCCAGCCACTGTTTATCATGTGTGGATGACCGTTTACATCCCTGCTCTCATAGAAAGGAACA
TACAGTATATTTGTGTACATGAGGCCTTTTTTTTGCCAAAAGATTATTAATATAAGCAGCTGTGTGACATAAGCCCTAACT
CTGTTGACCCTTTTTTTAATATTTGTATGTGTGTGTGTGTGTGTGTGTGTGTGTGTGTGTGTGTGTGTGTGTGTGTGTGTG
TGGCAGCTGGGAGTTTCCCTCCAGCAGAGAGCTGATGCCTGGACGGACGAGGTGAACGCCACCAGAGGACTCCAGGCCAGA
CGACCCCCGTCTGTGGAAAACTTTGTGAGTTCAGGGCAGAGTTTACAGAAATCAGCTGCGTGTATTGAATTATTCAAACAG
CGCTGGAGCTGGATTGCATTTGAGTCTTTCAAAACCCCAAAAATATGCTGCTGGTGTGAGATATAATCCGGTTTCAGGGGA
AAGGACTGAGGGAAGCTCTGTAAGATCTGTTTGGGGAAATATCTTGTGAAATGGATAACAGAATTTGAGCCAGTCAAAACT
TTCATAATGTGGTGTCTGAAGTCGAGATGATTAGATGAGAGAACATAATGTGATTGAACTGGCTGTAAGGGATCATACACA
GACAGTGTGTTAGGCAGAAGCCAGTGAATTATTGCCATTATATTTCTGTTACCAGGGTGCCCTCTGCTGGTTGGATCCGAT
TTGAAACCATCAGAATGAATCACAGGACTGACACGAGACATGATGCTGTTAGAGGGAAGCTGCCTGAGGAGGATTAAACCT
GTAGCTGACATAGAAGCACAGATTACATGAGAGCTGCACGATTTAATTGATTAGTCGGTTGAAATATAAAGAACTGCTAAC
TACTTTTCAAGCAAAAATGTCATTTGCAGCTTTGTGCTTCTTAACTGTGAGGATTTGCTTATTGTCTTTGTTATTTCTGAT
AGTAAATGAAAAGTCTTTGTGTTTTGGACTGTTAGTTGGACAAAAGAAGAAATTTGTAGACTTCACTTTGGGCTCAGGGAA
ATTGAGCTGTTTTTCACTGCTTTTTGGCAGAATAATCCATAATGAGAATCAGCATTAGTTGCAGCCCGGGATAAAATTATT
TTCTGGCGTGAAGGAAATTAATATGTGATTGTGATAAGGCAATAAATCTCTCCTGTTCAACCACTGGCTTTAATCAAAATG
TGAACCTTATACATCATCTGAATAAACCCAGATGTCTCCACATCTTAAAACAGTAGGATTAACACCATGGATAATTCACAA
GCTGACACCATATGGTCCTTTGCATGAAAGCTGCAATTAAGATGTGAGTAAACAACAAAGACGGCCCAGAGCTAAGCCCAG
TCAGTTTCAGTCTGTTCCTCATCACAAGGTGACATGCCAGTGAACCCCACAGATCCCACATCCTACAGTAATAACCATCAG
AGGATTAAGTGGATCCTGTGTTTGTTTTGGTTTGGCGTTGGATTTATTCTCGCTTATAACAGAGTGTGAGCCACTTCAGAC
AGATATTTGAGTTGCCTTTCCACAGTCACTCATCATATACAGGACATTACTTGAGATAAAGGTTCATTTATTTGTCATTTT
GCAACACCAGGGTTGTAAAACTAAACTGAATTTAGTCATTTTATGCTACATAACAGAAAAAAAAGATAAAAGAAGAAAGAA
GCTGCTCTGTAGTCTGGTGGTACAGCAACAGATACTTCTGTATCTTTTGCCAGATGGCAGCAGGGTGAAGAGCCTGTGGCT
GGGGTGGGTGTTGTCTTTTAGTATCTTTTGGGCTCTGTGCAGACATCTCACTTCACTGATGTCAAAAATAATGATCATAAG
CGTTTTTAGTTGGCCCTTGGTCTCAATAACCATGCCCCAAGCCCCTGATGTAAGTCGTTCTTTGTTCTAGACCAACAAAGT
GTTGGTGCCGGTCCAGAACCAGTTTCCCTGGACTAGAGCCACTTCTTTGGCTGTAGAAATGCGAAGAACTTGTTCCAGAGT
AGGCACTGGCTCTGAACCAGCACCTGAACTGCCTTGGTCAAAAAGTTTACGTTGATTCCCAGGAGCCTGAAAACTGTTCAC
CTGCTCCACCTGAGCTCCACTGATGTAGACCAGGGTGTCATCAGCTCCTTTTTTTGGTAAAATCATCAATCAGCTCCATCA
GCTTTTTTTGCCAATGTTGAGCAGTAGATTGTTCTCTGTGAACCACTATGCAAGATTGTTGATTTCCTCCTGGTATGAACT
GTCTTGGCTGTTTGTAACGTGGCCGATGACAGTGGTGTCGTCTGCATACTTAACAACAGCATCAACACAGTCTCACCGAGT
GCAGTCATGGGTGTACAGTGTGAACTGGAGGGGGCTGAGCACACAGCCCTGGGGAGCTTCGGTGTTGAGCACTAAAGTAGA
GGAGGTGTGACTGCCAATCTGAACTGTCTGGGGTCTGTTTGTGAGGATGTCTAATATCCAACTGCAGAGTGTTGGGCTCAA
GCCCAGCGCAAAGTTTTCCAGTCAGCTTCATGGGAGAGATTGTATTGGATGCTGAGCTGAAGTCAACAAACAGCATTCGGA
TGAAGTTGTCTGTGTTTGTGCATGAACTGTGCTGTATATTCTGGGTTGTGCAGATGGGGATGAAGGAGGAGGAGTACAGAA
GGAACAAAGAAGAGCCGGCCCTCGGCAAAATACCCTCAAGGGGTGAGTGGTTACTGCGAGCCATTCAAAAACTCTGTATTT
ATTTTGAGAAAAGATATCACTTTACTGACAGCAGGAAACCTAACCTAACCTAACACAGACGATAAATCCTACATGTCAACA
GCGATATCATAAAAGGATTTCGGTGCCACATATTTCATGTTATGTTTAGAATCAAAGTATGTCTGTAACATTCACAGCCCA
TAATGCCTCCGCCTTGAGCTCATTTTGCCTTCACCACCCGCTTTATCATTCTTTAAGCCTTTTATCTGTGTTTCTCTCCCT
CCACATACTTCCTCATTTATACTTGCCTCTGCAGAAAGTCTGTCTAATTGTTTGCCTTGCTTATCTAGAGTAAAAACAGAT
AACGGTACCATAAACAATCTCTAGAATATTGAGTGGAAGTTCCACTAATGTTCCACTTTTGCTCCTGTGAAACATTGCACG
CACCACGCTCGTTGTCTGCAGCACTACAATGATCCTCACTAGAGAGTAAATAACACTGCCCCCCGTTTTTTGTGTAATTCA
GCGTATTGCATCCCCTGCCATCCAAGCAAAATAATTACACAGAGAGTGAGATACTTAGTAGTCTGCCTACTCTCTGAGGTA
TGAATACCAGAAACATAACGGATAAAGAAATAAATCATACCCTTTTATATTTTTTAATGAATCATTTCACCAGAGATCCTT

CTTTGCCATTTAAGAGGTTATTTGTGCGGTAACAAACAGTTCAATTTGTCATCTCTCCAGCTCCGTCTCCCCAGGAAAGCG
TTTATCGCTCTTCAGGAGAGTCACTGGGAGGTGGCAGCGGAGCGAGAACCTTTAGGGCGAAGGTGGCCCACCAGCCCGGTG
GCTCCAACCCGACCTTGCTGCCCTTCTCTAAGGGAGAGATGATCACTGTGAAGGTCCAACAGCCAAGGAACGGCTGGCTGT
ATGGACGAACTGAGAGCAGTTCGCGGTGAGCTAACCCTAACCCTGTGTGACTGCTTGTGCATCCACTGTAAATGTGTCTGC
TGCTCAAGCGAAATACAGTATATGGTGGCAATTTTGCTGCTGTACTGTCTGTTGCACACTGATACCATTCAGGAGGTCAAT
ATATTCAGGTGGTTTTCAAACATTTTCACATTTAATGCTCCAGGTCCCTTTTCTTCAGCGAAAATCAGCCGGTGAGAAAAA
GAGTGATGCATTTTGCATTAGAGATGCAAACATTTGTAAGGAATTTATCGCAAATAAAAGGATATAAGAAACAATCAGTAT
CGTCAGCAAATAAATGGAGAGAAAATAGAAGAATAGTATGTAAAAAGCAAATAAACAAACAGAAAAGCAAAATAAAACTTT
AATTGAAACAACAATAACAAGATAAGGAGTCTGTAGGCTGTACTTTAAGGTAAGTGCATCATTGCATAAAAAATCAGTGCG
ATCATGTGCACAATGACAAAGCTCACATGCTGGTGTTCAGCATGTGTTCTATGCTCACCATATGTTACAGTGTTAACATTT
ACTAATTAGTGCTAAACACAGATTCATCCTCATGGTGTCCATGAACGTCTGAACAAAATTTCATGTCAGTCCATCCAGTAG
CTATTTCAACTGTATTAAAAATGTATTTGTCTTTTCTAATTCTTTTGAATATGTGACTCTTGACATTATTCACGACATTAA
CACCAAATCCACCCACTGTCATCTTTATTGTCTGATTCTTTGTTTTCAGTCAGGGATGGTTTCCATCCACCTATGTGGAAG
CAGTGGATGATCCACTGATGATGACCAGCTCTCGGTAAATATCCAATATAATGCATTCAACAAGTATGACTGTTAAAAAAG
TTTTTGTCATGCTAAGTGCTGGCTACAGGTTTTGCATGTGTTCTGCCTCAAATTAATTTGTCATGGCAGCTGTTAAAAATA
GATGGAGAGACTTTAAACCAAAGACAAACCTTCTGCATAATGTTTCTTTCATCCCTGATACACAATACACTTCGCAGTTCC
CCCAGGCCGTCCTTATTGTTTTATGGGTCCAACATTTTTAAAAGAGGGTCAGCATGAAACTCAGGCCTGGAATAATAACAT
GAACTATTTAAGAAAAATGGGTGACCCATAGCTGTGAGGAAAACCAGTAGGAGGTTTAAAACTAAAAGTCAGCCGCTGTGA
GCACATGGCACAGCTGAATGCCCAAAATGTGAATCTTAACATAGATCTTCTGAACGTATGTATTTAACCTAAAATCTCATA
TACTTTAAAAAGCAAAATAAGATTTAACTCATATTCTTTATGTACAGGTCTAATATCATTTAACTATGAAAGACAAATGGC
ATTTTTAGGAGGACATTTTTTTTTATCTGGTTAGACTCACAATAAGGACATTCAACCATACTCTGCTTCATTTACATATAA
AACCATTGAATTTTATCTTCGCTTCAATAGCTCCGCTTTCCGAAGCAGCAGCAGCAGCGGCAGCATCAGCAGCATGAGCGA
CCTGCTCGACCAACCACGAAGCAGGAACCAGAGTGGAGCCCCGCCCCCTCCACCTCCACCTCCACCTACATATTCAAATGA
GCAGTCTGGGATGCGACAAACCACGCCGACTTTTGACAGAAGGGCTGAGTCCTTTTCAGAAAGTAAGGTACGGCTATCAGT
CAGTCTGTCTGATTGAGCGATCAGTGTGGCTCGTATCAATCAATAATCTCTCCATCAGAAGATTAAGAATGTCTTTTTAAA
TATGATCACAAATGGACACCGAAAAGCAGTTTTCTAGGAACTTTCTTTTTTAACAAAGCCTCTTTCTATTGTCTTTCTAAA
GTCTGTATAAGTTGGACTAATTTTTTGTTGCTGTTATAGACAGACAAACTCAGAGAAAATAGGGGAAACTGCTTACATACA
TACCTGTTTATTAATGGCTGCCATGACTGAACACATTTGTATTTTTCCCACTTGAGATGGAGAAATGATGCGGGGCAGCTC
TGATGAGATACACTATGTAAATCCTTGATGGTCTTTCCCAGTCTGTCCAGCAGGGGCACTGTGGCAAGGTGCTGTTCTCAA
TGGCACCACACCCGTCTTTCTGGTGGATGCAGGGAGGGAGCTCCAGCTGCTGACGGTGTATGGGCGAGGAGGTTGTGTGCA
CGTGTGTGTGTGTGTGTGTGTGTGTGTGCGCTCCTGTTTTAGCATGCTGTTAAAGTGAAGAGCTATTTGAAGTCCTTGTGA
CTAAATAGGTCCTTCAGGGTGCTGCAGAGAGAAAACACTGTGTTCTTTGGTAGCTTCAGTAGAATTTCTGTTTCTGCATGC
AGAGATTTTGACATTTGAAAAGGTGTGATTCAACAGTTTGTGTCCACTGAAAAGTGTGTGTGCTTGTGTGTGTGTGTGTGT
GTGGCAGCTTGAAACAACACATCAATGAGCAGCTTGTGGGAGGACCGACTGAAAGCAGAGGGGTCAGGGGCCGGAGCCCGC
TGAGCCTCCATCAGAGCATGTGTCCACATTTTGTGGTTTGGATCATCCTTCACTCACCTTTCTCCTTTTTTTCTCTACAGA
TATCAAATCCACATGGATCAAGACCAGAACTCTTCCCGAGGTAAGAATAATGTGCTGACAGGTTGTGAAACGTTCAAATGG
AGGCCACTCATGTCCTCTTCCACAGAAGCTAATGATATTTTTTGAAGTATATCACGAAGCCATTAAATCAGAGAACAACAG
ACGATGAGGGCCGCGATCTAAATGGCACAGAATCGCTTTCCTCCCATTACAGTTTGAAGTGCGGTTTAGTACTGAAACCAT
TTCAGTAACGGCCATTTGGGAGCCATGACTCTCACTCTCGCTCTCACTTCAGGTGAGCAGTTTGCTAGATGATGCTCGTGC
GGTTTGAAATGCGGCGCTCGTTTCTCGTGTAATAACCTTTATTGTCGGGCTCAACGATGTTGTTTAGGCCACGGCATTTTC
TGATATTAGTTTTCACCACTCTGTTGCTGTTTTTTCTCTCTTCATCCAACATTTTTTTCTTCTTTTATTTTGAAGGGGTAC
CAATCCATTTGCCACAGTTAAGCTGAAGCCGACGCACACCGACGACCGATCGGCCCCACGTTTTAACCGACGATGACATCG
TAACCTTGGAAACGATGCTTTCATCGAGCTCGACTGTTCATTCTTGGCTAGTCTTGACCAAGTGGACCTTGAGTGGAGGTT
GTTTTGTCGAGTGTTGTCATACCTCTCACAGTAGTCTGTTTGCCATAGTGTCTTTCTGCACACAAAGTTGGAGAAAGAAGA
CAAAACTCCTGATTAATATTTTAGAATTCCTGATTTCGCACAAGATAATATTAGATAGATAACTTCAGATAATATTATTTT
GATTATTTTGAACAACCAACAACCACTCATGTGTTTTTGTAAGCTTTTTCATAATCAGCTCTGGCTTAGTGATTTATAAAA
TAGTAAAACTGAATCACTGTGATACATAAACTGATAAGTTCATTGATCTTACCGTCTTAAATCACCAGTTTTCTCTGAAAT
GCTTGTTTTATTGGCACTGAAATATTATATTTAATATTAGAAATAAATGATTACTGTCCATGATGTGTATATAGTGTACAG
CTGATGTTATGAATTGATGTTGGATCTGCCAGCTCAGGGATGAATGCACAACGCCATGTTCTGTATGTAGCTGCGTAAATA
ATCTTTATGTTTTAAGTTTTTATTGCCATTTTATTGTATCTCTGCCATTTTTCTCTCTCTGTTGAATTAACTCCATTAATA
AATATTAATTGCATTACTAATTTACTTTGGAAAATGGCTTGTAGAAAATGAAAATGTAACCTCTGGCATAAAAAATGTAAT
AACTTGTAAAAAAGAAAATATGAGAAAATGTCAGAGCCAGAAATGTAATGAAAAATGCAGTGCATTCATGGATAATGAAAA
CCCATCTTTATAGCATCATATAATTACTTTAACTAACGGGTTTCACTGTATTAGCAGGTGGTGTTTTTAGCTGCTGGCGTA
CTGATAAAGTAGATCACAATATGTGAGGTATTACATCATTAAAATGCTGCTGTTAAAGCCCCTGACAACTTCATCTCAATT
CTTTAGTATTTGTCTATAACATTAAATATTTACTCATAAAGGGGCACTATATAGTTTTGGAGATGAACTCAGAATTTTTAT

ATTTACAATATTAATGAGGTAATAATACAACCTCAAAAATATTTATCTTTTCCATAACTGAATAAACAAGCTGTTCTCAGA
GTAAAAAAAGGTCCACTGAACACTGTTTGAAGCTAGAAAGGTGGCAGGGTCCGCCGCATATAAGTTTCAGTTTCTTCAGTC
ATGAAAACAATGAGAGTTTGTTTATTCAGTTTGTGTAGGCATAAAAGAAAAGTCAATGAAGATCCTTTAAACCTTTAAACC
TTTAAAATAAACAATATAAGTTAATGTTCAATAAAAAAACAATCTGCTTAATGAGCATTGTGATTGATCAGGTCAGTTATC
CATCAAACCAGCAGCTGTCATCAGATTTTGACTCGGACGCTGCTAAATCCTGATTGGAGCACAGAAGGAGGTGACTTCCAC
TTTTCCTGACGGAGCCCCGCCCACCTCTGATTAGTGAGAGGAGCGGAGACAAAAACAATACAGACAACAAAAAAGACAGAA
AACTTCTTGAGAAATAACCCAAACATTTCAAACTTTGGAATAAAATAGTGTTGTTTAACCACGTCACGTGTCATAAGAAGC
TGGATTAGAAATGTTATCATCATACTGTGTTTATTAAAGGAACAGCTTCTACGTATGGACAGTTTGTTTCACTGCTTTTGA
ATTGCGCTTTATCACGTTAAACCTGCCGTGTGGAACTTTGTCTCCCTCTTCTGGCAGTGACAGTTATCACACAAACACTGT
GTCAACATGTGCTTGCAATATATGGTTTGCAGGCAATATACATTTCTTATATTTAACTTATAATCCTTCTTCGGAAAATTC
AGAGTTTCCTATTACTGCTCCTGAAACGCCTCGTTAGTCGTCTCACCTTTAATTCTGTGACTTTGTGACATCACACTATGT
CAAAGAGTCACACATTTGTATTATTAAGCAGCTAGTTTGGCACATAAGAAAAGATTTAGCACCTCTGCTCTGTTGTTGTTA
GCGATGCTGGCTCAGGTGTGTGTGAGCTGACCAATCAGAGCAGACTGGGTATTCAGGAGGGGGGCCTTAAAGCAACAGGAG
TCAAAAGTTTCAGACAGAAACACTAAAGCACCTAAACCTATTCTAGTGGTAACCAAAACTAAAATAATGAGCCTGAAAAAA
GTAATTTGAGGTGGCTGCAAATCTGCACCAGACTCCTTTAAAAATTCACTGACTTTTGTGAGTTGCTGAGTTTGAAGAAAC
TCTACAAGCTCTGTGAAACGTTGTCTTTTAATTTTTCTTAATTATTTCTTCCCTCTTGTGAATTTGGCATATGTAATACAT
TAGGTTATTCCATTCTCTGTATTAGAAATAGTGTATCAGGTCATCTTGGTTTGGTGCCGGCTCCTCTGTGACTATATAAAA
TATAAGCCTTGTTACCAGAAGACCCGCTGCACAGTTGAGCACAACACGCACCCCCTGCACACACAGTTATTTCTTACTGTA
TATAATTCATTTTCCACCCAAATTGCTTCAATTCTTCACACCGCTCTGTTGAATAATGCAAATTTTGGCACTGTCATGGCA
AAGAATAGGCGCATAATTAGCAACCAGAGCCGCGTGACTTTCAACTTCCTCTCGATATCACTATATCTATATTTGAACAAC
ACATATTTTACGTTGAGCTCGATGTGAGCCAGACTACATCCAGATGTGTTTAAGCATATCTGATCACTTTCCATTCACAAA
AAAATCCAGATATAGATCGTATTTTAACACCAGGTGTAAACAGGGTGACGGACAGATTTATCTGGCGGTGATGATGCCACT
TCCACAAGCCACTTAACCCATAAACCCTCTGACGCTTTCATTAACAGGCCATTTCAACCAGGCTACGCTGAACTCACCAGC
TCTGTTCCCCCGTCTCTTTAAGTGATAGCCTCAAAGCTTCAGATCTGTGTTTATTTTCTCAGGCGGTGAGATGGCCTACTA
AAGCAGATCAGGATTCCCGCGTTGCCTGTTTGGGTCCTCCGCTGGGGATACTTAACCCAAATTGAACCTCAGTGTGAATCC
AATTCTGCTAATAGCTTGACAGCATGGTTCTGTTAAACTAGCACGGCACTACTGAGTAAAGACTCTTTGGCAGGTGGAATA
ATACGTCCTCCACCTCGTTTGTTCTCTTTCAGTGACGGTGCATTCTTAATGTAGCGAGGACTAGAGATGACGCAGTCGGTG
CTAACGAAAACTAACGAATGACACTGGAGACTAATTTCGACTTTACTTTTTAAAGTACACCCTTTTATTCATGATTAGGCT
CCTGTCAGGGTACGCTTCAACCGCTCTGCTCGATAAGAGTAAATATGAGAGCGAAACAGGGAAAGGAAATCAGAGAGAGGA
GTCAAAGGAAGGAATTAACAGACGAGTCTTCATGAGTCTCAAGGCTTTGTTGTTTTTTTTAAAGTTTTTATTTGAGTCTTT
CACAGCTTGAACTCTTTCTTCTTTTGGTATGTTTTACAACAGCTCATTACCAGAGGAGAAAGGGATGGATGGATAGATAGA
TGGATGGTGTGTGAAGGACAAAGGGGAGCGAATGCAGGAAGGGAACCGTGCCAGAAGTGAAATCGACAGAGACATGGGGGA
AAACGGAAAAAAAAAAGAGGGGTGGGGGGGAGAAAGAGGGAAGGTGAGCGGAAGTGTTGGCTAAGAAAATGAAGAGACATGA
ACGAGAGTACAGTCAGAAGTACAGAAGGAAACGAGGCTCTTTCTCTTATATTCTCTGTTTACTTTAAATATCTACTTTATG
CTTTAAAGTGTGAGGTTTATTCCTGCAGGCATGTTTTATCTTTATCTATACATAAACAGAAACAAGAACAGTTTGTATAAA
AAGGATAAAATAAATAGGATGAATATAGCTGTGGGGACATGTGTGTGTGTGTGTGTGTGTGTGTGTGGGGAAGGGGGGGTG
CCGCGCCACAGCTTACTTCCCATAGTGCAGCATTGCACTTAGCGAAGACAGCAGCGTCGGGGGGGGAGAGGGGTGTGTGTA
GCAGGAATCAGCTGCAGTCTGGTGGGCTGTGGGAGTCAGGGTGGGGCGGTGAGGTGTAGGTGTTGGGGAGGAAGAGGGTGG
GGGTGGGGCAGCGGGTGTCAGGATGTAGCGGTGGCTCGGAGGTGACCTTGGGAGTGCTAGTCAGTCACTCGAGGTGACTGT
TTATTCTTTCACCAGGGAAACAAACTCTGCAAGAGAGGAAGACAGAAAAGTTGTCAGGATGTCAGTTATTTTCCCTTCCCT
TTTTTTCAAAGGGGCACTGTGAAGTTTTGGGGAAGAAATTTTAATAAGCCGTTTTTTTTTTTTGTTTATATGTGGCGGACCC
TACCACCTTTTTAGCTTCAAACAGTGTTCTGGGGACCTTATTTTCCTCGGAGAACAGCTTGTTTATTCAGTTATGGAAAAG
ATAAATAATTCTGAGTTTGTATCATTAGCTCATTAATATCGTTAATATTAAAATTCTGAGTTTGAATTTCTTCTCCAAAAC
TGCATAGTGCCCCTTTAAAGAAACATCGTGGTAAAAATGTGTTCAGCTTTCAGTCACATACAGTAATATAGTGTGCACAGT
TTGTAGTTAGGGACTGTATGAAAACATGGCAACATGCCTTGTGTGCATGGACCATAAAGGCATGTTTTCACATATGCAGAG
GACAAAAATCACGTCTCCTTCTTGGGATTTTTTTTTCTAGAAGCTGTAGATACACATCTACTGTTTCTGGACCTTTATATT
TCGTAGGGATTCAACATAGGTCAAAGGCTCAATCCCAGTGTCCACACCAACAGACTCAGACTTTGAGGCGCCTTCCCGGTA
AGTCCATGAGGGTTTAGAGCTATCCCACTGTCACATCATTACACTGACACAACATGTGCATGAGGGCTCTCTCGCAGACAT
TTTGAGTGCTTTATGGACACTTTCTATAAGTCCGCATATCTGCAAACCTGCACACTTCATGGGTTATGACCTTACAACACA
GGGTTATGTGTTAACTACCCATTATATAGTGTTTCAGTCAACATTCACTAATAGTAGTTCAAAAAATAAAATGAACCTACA
GGGAAGCTGCAGTCTTAATAAATGACCTATGAATAAATGTGTGGTGAACCGCAGTCGGCAGCACATGATGACAGTCCGCCT
GTGTTCATCCGACTCTCTCTCTACATTCATGTTGTTTGTGTGACCTCGCGACACTCCCACTAATAGAACTTGATGTTTACC
ATCGACTCCAATCTTGCCGTCTCCATCCTTGTCGGCTGCTTGTAAAAATGCTTTGGTTTCTTTGTCCGTCAGGTCCCGGCC
ATCTTTGGCGAATCCCTTCAGGACGAATCTAGCCGGGGACAGAAAAGCAGAAAGGAATCGTCGTCACAGGACAATGATGTT
TAATGTGCAGCAAAAACATTTGCTGCTGGGTTTACATGTGTGTCTGTCTGTGTGTGTTTGTCTCACTTGAGCTCCTCCTCC

TCTATGAAGCCGCTGTTGTCGGCGTCCAGCACCTTGAAAACCTTCTTCACGTCGTCAGAAGACTTGGCCTTCAGACCCACC
ATGTCGAAAAACTTGTTATACTCAAAGGAGTCTGCAGCTGCACACACACACACACACACACACACACACACACACACACAC
AAGGAGGCACACAGGCACGCAAACACAAAGATGTAAAATGTCATTACAGTACTTTACAATCACTTTTAAACTCCAATATCT
GAACATGGGATGCACTTTTTTGACACATACTACAGCAGTCAGTGGAGGCATCTGATGAGACTTCTCAAGAAGTTACTTTAA
GTTTTTTTGTCCTGTGTTGATCTGGGAGATGATGCCCAAAGGCATAAATGTAATTATAATAAGGCAGCCTCCTCACAGTGT
GACAGTTATAGATTGAGAATAACCAGAGGACCCGTCTGTTCTGGGTCACTGAGCTCTGTGTATTCTCTATCTGCCTACACC
ATAGTACAGGTATGCACAGAAACTGCAGAATAACTTCGCACAAATATAACATGAGTCACAGCTGGAGCACTCAGCATAAGA
GCAAACAACTTTGTTTGAAAACATCTGGTTATTTGATAAAATGTCGAAAGGTCGAGCCAGCGTACAGAAAAACAGCGTTGC
AGCATTCTCCTAAATAACTGAAGTAGGTGGGGACTTGTTTGAAAACAAAAAAAAAAGGAACCACAAAAAACAAACAAAATG
GGTCCACACAGCTCATCTGACGCAATCCAAGTGTCCAGAAACCCCAAGATCCCAAATTGTTTGAGAAGATGTTATTTACAT
TGTTTGTTAAGCTGAAATCTTCACTGTAGCTGCTAGAAATGTGTGCACAAGCTCGACTGTGCATAGAGGGTGTTTTTTTTT
TTTACGTTTCTAAAACATGTCCCCTTCTACTCCAGCCGTTTATGAGAATGCTGTAACGCTGTTTTGCGTTGAAGCTCCAGA
AATGTTTTGTGTATCACCAACTTTCCATCGTCACTGGGGTTTTTCATGAGATTTCATTTCAATCATCATTTTTAAGTGAAC
TGTTCCTTTAAATAAATTTACTTTTGGATTCTGAGACTTTTGGATTAATTCAGGAATGTTGGCTCCTTTTGTTCTCAACTG
TATCAGCAAAGATTGTTACATTTCCTGCCGCTCTGATTCACATCGCTGACCAGCTGAGGCTCATGGTTATTGTAGTATTTT
GATCCATCCACTGACACACTGGTTGGCACACTGGCTCACATTTGGTCCACTAGAGACAAAACACAGCTGAGATCAGCTCGT
CACACCAGGTTCCCTCCTAACCCTGCCGTTCATCCATGACAAGTGCTTTACCTGCAAATGCATCTAGAGCTTTCTTGATGT
TATCAGCGTTGAGGATGCTGCTCATTGCCATCTTGGCTGTTAGAGAAAAAAAAAAAGAGATAGAAAGAGAGAGACAGAAGAA
CAGAGAGGGGACAAGGTCAGAAACAAGGTCGCCAGAGAGCAGAGAGAGAACTCGGAGACATTCTCGTATTACACTGGTGAC
ACTCTAGAAATGGAGCAGGAAACCAGGACATGTGAGAAGGAGGTGATGAGTGACAGACTGAATTAGTGACAGTCGAGGAGG
AGTGACAAAAGGAGGGAGAGGAAGGAGATGAGACGAAGGAGGAGGGAGAGGAGGTTTAGCTGCAGGGACGAGGCAGCAGAG
AGAAATATTAAAGGAGGCACACATGGACAGCGTAGTGTCCATAACAGTGTCTCCCATTTCATCTGTGTGATATCATTGAGT
TGTTACAGATGAGAGAAAGGCACGGAGCCAGAGGCAGAGTCAACAGGGGTTTAACAGGCCATCTGAGTCTTCTGCTGTGCC
TGAAATACCACAGGAGAATTCAGTTTGTCAGCCCAGTCCTCATGGAGAGTCCTCCTGTACCTCACGCTATGAAAGGTGAGC
CCGGCACTTAACGTCACTGTGGCTTTTAGATCTGTGTGTGACTGAACCCGATCGCTGCTTGTCAAATTCCTGAGCACAGAG
GGAGGCCAACGCAGAGAATCTCCACACGAGCAGTGGAAAAAGACTGGAGTCCAGGGAGCTGTTGTGTTGAGACGGAGTCGT
TGTGGTCTGGCAAACCTTATTAGGGATTTGGCTGGGACACGCATCATCTCAAGTGGGAGAAAGGAAGACGCTTATTGAGCT
GTCAGTTGTCTTCGCTTCCACAGACGTCAGTTTGTGAGTACATGTGGGACGAGCATAGGCCATTTAGCACGTACAGTATTT
TTAATAGTTGCTCAGTAGGTTCAAGAGCTTCACGTGTGGCGAGTCACGTCTTTAAACAGCATTCAAGCTGCCAAGCTCAGT
GGGGTCTCAATGGATGTTGTCCAGACGAAGACTAAATGGGAATCCATATCAAAAGTGTGACATCAATAAGCACATCAGCAT
CAAGCCCTTTCAGCTAACTGCGACTCACATTCAGCCATCAGTGTCCTCATAAACAGCCAAAGCCAAACTGTCTTCAACAAG
GCCGACTTAGATCAGAAATGTGCTGTACAAACACATTTTTCAAGTTGGCTCCACGAGGAGGACGACAGAAGAAAACGGCAG
TGATTCATTGATTTAATTAAAACTGTCCACCACAGGAGGCAGAACTAATGCCTATAGTGAATTCAAAAAGCTCACAGACAC
ACTAGGGGCTCTTTATAGTTACCATTAATAAGTTCACAACATACTGTACGCTGTAGGCTGAACAATGAGATTTAAACTGGC
TTTGGAGAATATGCTTCATGAAGTGTACCAAAGACAAAAAAGTAACGTGATGCTCCTCTCTTAAGTGACTGTGTATCTGTT
TCTACGAGTCGGACGAGCGAGACGGAGAACACTTTCTGTGCAATTGCCCCTCAAGAGTCTAGAGGCTCAATGTTACCATGC
TGATCTAGCATTTGCATGCTAACATTGGCTCAAACCAAAGTATTGAAAAGTTGAAGTTTTGACGGGGCTAAATGAAAGCAC
TGTTACAATTTATCCTGAGTGGAGCGTGAGTGTCTGTACTAAATTTCATGGCAATCCATCCAATGGTTGTTGCGATATTTC
AAGTAGTAGACTTGACAGACTGACGTTGCCGTCCCTAGGGCGAGGCTAAAAAAAAATTACCTTGTGAGATGTTGTCTGACT
AAAAGCTGCGGCAGTATCCCGTGAAGGAGAGCTCACTGTAAACAACTGAGTAACAGTCTGAGCTGACTGTAGTTTATCTGC
AGCGGGCCCTGGAGCTCCTGGATACACACAGATACTGTAGATAGATGCACCTGTCTGTTTGTGAGGGCAGCTCACTGTATA
CTGCTGCATAATACATGAAACATCCTTCAACCTTCTGACTGCGATGATGATGCAAAGCGTGGAGAAGTGAGATGTCTGGAG
AAAGGTCACAGCGATGACACCGTATCACCATCATCCTTGTACCTTCATCATCCCCGTGTTCCCTCCGTGCAGCACATAATG
CTGAGCCGGCAGATATCATTGCTCTGTCCTTCTCAGTGGAAACACAGTCTGAGTCGTCTGTACGTTACAAATCACTCATGC
TCCAGCGTTTTTGCTGCATCATACCTGAAGCAGTGCAACTATTCTAATAGCATCAATATCTAATAGCATAACAATATCACT
GAAAATGGACATAAAACACATGGTTTTCTGAGGAAAATAAATTTAAAAAAAAAACACTTTATGACTTTAAAATACCCATCT
GACACTAACTTTGTACATTCAAAATGGTCATAAATAAGTGCTTAGGAAGCCGATACAAAGCGCAAATTGGATGAAATGTCA
TAAACGTGCATAATTTCCAAGCTTAAAGGAGCAATATGTAAGAATTTTAGTTGCAATCATCCAAAAATGAACAAAGAGAAT
GTGAATAACAGCTCAGACGTTATGACGTCTATGTATTGTGTTGTAAGCATATATACTGAAGTTAGCATGCTAACCAGCTAG
CCCCAACTCATCCTGGTCTAAAGCTTCTATGCAAATGGTGCACAGCTAATTGAGCTACCTAGCATAACGATATGCTATCCC
TTTTGTTTTGAGTATGAATTCAACAGGTGGTCAGTTCTTACATGTTGCACTTTCAAAGCTTCAAAACACAATAAAATTATA
TATCTATGGATTTGAAATCTCCTTGGCTACACAATGTGTCAATCATCCAGAGGCTGAAGACAGAGTTGGTCTCCTGTCCTC
CTGATCTATCGTTGGCTGTTCCAGGCCACGGGGCAGGAATAGAGTGTCAATCACTAGTTCAGTTTAATGGCGTTTTGCTTT
GAAATAGTGTTTTTGAACAGACAAAACAATGAAAAAGCAGCGGTTTGTCACTATTTTGTCATCATTTAAGCATGGAAAACT
GTGTTTTGGACTAAATACATTACTGTAATGGATCTACTGGACGTCTGGGAATAAATAACATCCTGCACTGTCACCAGCCCG

AACCATGAGTAGATGCACGATTCCCTCTACTCAGTGATACAGTTGGCACAACAGGTCTGTGGATTATCTTGAGTAACCAGG
TCATGATTTCTGGAAGGAGACTTGAGTTTTTAAATGTTTTATTTTGCTCTTTGAGCACTGCAAGCTGAGCGCCATCTACTT
CCATTATATTCAAGAGGGGGCCGACATCTCTACGGCCGATAGCTCACAAACTCAGCATCTCACACAAAAACAGTCTAGATG
GATACAGAGTAAATATGTCCCCAGTTATAAAACTGTAAAATGTTTCCTGGCGAGATCAGTGATATAAACTCTCAGATAAAT
GTAGTGTTTGAGGTAGTTCATAGACTTCTTTCCCCGGTGTCACACTCAGCTTCAGTGATGTATGAGCTTCTTATTAAGGTA
CGCTGTTATCTGCTACTGTAACATACTGCTGCACGCACAGATCAATACTGAACACCGCTGAACTCGTGTGGCCACACATTC
AATCTTCTATCATTATATCAGCACAACCTCCTTCTCTGACCTTTACCACAAAGGTGTCCACAGGGGAAAGAAACAATCGGC
TGTGAAATGAGGAGGATAAGATTGATTTACAGAGATACCTCAGCCAGTGGAACTGTACAACATATGCCATGTTGATTTATT
TAAAGGGAAGTATAAAGACCGATTGTGGTCGGGCTGATCTGTGGTCTGTCTCTGCAGGTTCTTAATCTCGTTCAGTTATCT
GAAAAGCTTCTGTTTTCTCTCTGTCAGTGAGGTTAACCTGCACCAACTTTTGTTTGTAGCCACTGGGGGACAGTGGAAACC
ACTTCTGAACACAACACTGACATATCATGACCTCAAAACAGTAGCTTCTTCGTACATCCAGCAGTTATGGAGCTAAACTAT
CATTCATACGAATCATGTTTCTCTCCACCTGATGAATACAAGTTCAATATCTGCTCTCTTTTAACTCTGCTTTTGGCCTCC
AGAGGGAAAAATCTGCCTTTTTAGCTCCTTTTTGATCACCGGCTAACTTTTAAGTGTGTCTGCTGAGCAGGCGGTATGAAG
TCCTGTTTTTAGAGATTGTTTTTGCTAAAAATAGCTGGCCACTTCAAAGCTCCAAAAAGCTGAGCTGCAGATTTGGGTGAT
AATCTTCTGTGGGTTCATCAATACAAGGGACACCTTTTACACATTGTTTTTACAACTTCAATCGATTGTTCCTGGAGTTTC
TACCTCTTTGGTCTTTTTCCGCTTCTCCATGCACCTTGGAAGTTGTGCCAGAAACCCCCATTCTGCTTTTAGCTGCATTGT
GGCATCTACAGCAGAATGTAGTGAGACCTTAATAAAAGTCACATGTCCTTCTGTGAAAGAGTAGTTTGGCAGGTTAGACGG
GTAGTCTCGCATTACCGGACCTATCTTCATAGCGCTGTGTCAGCGTTAGAGAATGGTCTGTCTGCACCGATTCTCATTCTG
GTATGGAGCAAGAAACACTTTAAACCAGTCACAATAGTCTTGTCTTGCAGGGCTAAACAGATAAAAATGGCTATATCCCGG
CGAATGAAAAGGTAAAAGCAACAAGCAAGCTATCTTGTTAACGTTTGTACCGTTAGCAACCAGGTTAATGTTAGAGTACCT
TGCTATTTCCAGTGTGTGGGTTGTTAGCCTGAAATTGGTGCTCAATGGTAGGTTTTTTTACCAACAGTCTACATCCGCTAG
GTCATAGTGTATGGGAAGAATGATACCAGTGTGTTTACTAATGTCGACAGTAAATATAATGCTACCACCAGCTAGCTTAGC
TTAGCTTAGCTTCGTGTAAAGAATGGAAGTTGGGTGGAAAGCTTGCCTGGCCTGGCTCACCTAAAGGAATCAAGGTAACCT
CCACAGCTCACTAATTAAACGTTATATCTCATTTGCCAATTACTACATAATGTAGCTTTAAATGCATCTTTTCTACAGTAC
GTTGTAGGCTGAGTTGTGGAAGATTAGATTATCAATTTGCTTTTGTCATTTTTTTCCCCAACACACACATTAGGAGCAATT
TGGGGTTCAGTATCTGGGATCGATCCAACAACCCTTCAAATAATGGCCCCCCACTACCTGAGCTACAATATGTTCATGTGA
AAATATAGGACAGTGTGGTTTGACTGTATTGGATGTAGATGGATGATATAGCAAACCTCAAATGACCAGAAAAGAATAAAG
ACATATTACAAGTCTCCAAAATCAATATTTTTCTGATTGGTAATATGTTTGTAGCAGTAATGTGTAAAGGCTCGTTCTGGT
TTCTTATTCTCATATATATTAGTGTTCAGTACTTCAAGCCTTCAAGCCTTTCACACTACAGATAAACAATGTGCCTATCTT
AGAAAAACAGTCAATACTTCACAGATGCAGCCGATCCAATCTTCAAACATACTGTTCTTTAATCTAACAATAAGTTCGTTA
ACCATCTTCCCCTTTCAAACCCCCATCTGAATTTAAGCAGGTGTGATATGAGCGTCAATCGGATCCCACCAAGGTTATCTG
CTGCGCCGCACTGCTGATGTCAAACGTTCATTGTTTAGTCGTTGCCATCGTCTATAACCACTATCTCTGCTAGATAAAATG
TGTCCACTCCAGTTGTGCTACACTGACTTCTGCAGTCTTGAAACTCCGTCCCTGAGGTGTGAAGTTTACCTCAAATCAACA
TTTTTATGACTGGAGCAGAATGCTGAGGCAGGAGGGGCAAGTTATGATGTACAGCGAGCCTGCTCAGAAGAGGCTCCATCC
AAAAGTAAAATAAAGAAAGCTTGAGTTTTTTATGCTACAGTAGACAAATTAAACTAACATGATTTATACAATTTTTATAAC
AGTATTTGTATTTCGAAGTCTTGGTAAACCTGCTAAAATATAAGCAGTTGAGTCCTGTCCAGTTGCCAGTGGACACATTTT
CTGATTCAGCATCACGAAACGCAAAACAGGGAACATTAGAAAGCAGCATGGAGGCCGGTGAAAATGTTACTGTGGTCACTT
CTTGGTATGAGGAAGGAATGAGCGCTGAAAAGTTTCCCAATGAAGACTAAAGCCGTGGATGTTAGTGGGTGTTTTTGGTTT
TTGTCCAGTGTGAAAGGGGGACAAGACGACGTCAGGGGTGTCTTTGCACCCGGCACACCTCACCGTGTAACTTCCATTTTA
GAGGGGTCACCTTGATTTTACAGACGACTTAGATTAAAAGGCTTTACACTTCATGTACAGTATGTAAATGACGCCCCTAGC
GATCTCTAAAGCAAAACAAAATGATGTAAGTAGCATGGGATCATGGGAGTTGTTGTCTTCATTGTTAAACAACAACAATTG
CTGATTGAACTCTATCTACGTCACTCGGGCAGAACCGTTGGACGAGGCAATTTCTTTCTTTTGTCGATTTTGCCAGATTCC
TTCCTCACTCTGACGTATCATCTGATGTTTTTGATGACGGGTGAAAAAACGAAATGCACGATACTGATGGCATCTCTCCCT
CATGTGGTCGACCTGTGAGCCATTTGAAGCAGAGTAATAAACTAAGTGAACTGGGGTTTCCTTGGTGGGAAGCCCCTTCAC
CCTGATGGTCTCTAGCTGTGGGAAACCTAACCACGCTGCAGGGCTGCCAACTCCCCAGTCTACAGTAACAGCTGCATGAAG
GTGTGAATATGTGTGTATGCATGCGAGTGTTTACAGAGGGTGCCAAACAGGCTGGGGTGAAGAGGTGGAAGGCTTAAAGAA
GAATGAACGTGCATCTCAAGAAAGCTGATTCCCTTATGTGCCTTTATGTGCGTATGTTTGTGGGGGAGTGTGTAGAAAAGC
AGAGGAAATGTGTGTTTGTGTGAGACGGAGCGAGACACAGTGAAGGGGAAACAGAAAAAACATACCGCAGAGAAATATTCG
TCTTCGGTGTCAGTGCATGCCATTTTTGAACTGGAGGTGAACTCTTGTCCTCCACAGAAATAAGCTTCTGAAGGTATTGTG
TGTTGAGTTTGTTTTCCTCTTTGTGCGGCTGTGTCAGTGTACCGAAACGAGGCCTTTTACAATGAGCGTTGTTTGTATGCA
ACTCATTCGCCCCTTGAGCTTTATGCTGTTTTAAGTTTAAACTGAGGTCGCCCAATAATGGGATGTGTTTTGTAATGAATG
AGTAAGAAAGGATTCCTCTGTGCGCGTCTTTGCGTTCTTATATAAGGACGTGTGGCTGCCATAGAAAGAAAACACTAATGT
AGTAGGATTGAGGTCATTATTTTTTTCTACTCTTTCAATTCTGGCTGGTTCACATTAAAAATACAAAGTAGTATGGTACTA
TCTCTGAGGGATTTCTAAAATGATTCATTTGAATTGCACTGCAGGAGAAACCGAAAATATTGAACTGTACCATCAATTTTA
AATGTCCCATGTTGTAGAAAGCGAGATTATCATGTCTCTAGTTTATAAAGCAGGTCTAATGTAAAATAAACCCACAATACG

CACAATATATGAGTGCGAACCATCTTAATATTGGTGGTCTCCATTAGAAATCTATCCTTGGTAGAGGTCGTTAAAATGAAA
GTAGTTCAAAACTGAAAATACGTTACAGAGCTACAGGTTAGGACAGATCTTTGAAGCCTCTCCAACAATAGTCACATGACA
AAAAAACAAAATTGTTCTCATTCAGTCCAAAATTTGAATTGACATTCTGACAAATTGCTTATTCATTGTAAGTTTATTCAC
ACAGGACGTCCAGTTTTAATATCTTTGTTCTCCAGTTTTACTAAAGCCACCCAGCTACCATTAATCTGAAGAATAGGGTCC
CCAGTGATTTTCTTCCTCCATAATAGGTTTTTATCTGAATTGCCACTGATCTGCACAGCTGATCTTTATATAAGCTCGTCA
GTTGAATGCAGTCAGAGCCGTCTTTTCAGTGTGCATGGCGTTGTCTATAGATTTTATAGAGCGGCTGATAGAGAGAGAATG
TACAAGTCGTGGAAAGGCCTGGGGATCGTCTGGCTGTTATCACTACTGTCCACTGTCTAATCCTCTGGCAGGTACAGGACA
GGGTGGCTTAACCACACTGTTATAGCCTGGCACCAACAATGGTCCTGTGTCTGTGTGTGCGTGTGTGTGTGTGTCTGTGTG
TGTGTGTGTATGTGTGTGTGTGTTCATAACTATTTATTATCTAATGGTCTGAAAGAGTCTACAGCAATGCCAGCCACTCAA
TGAGGCTGTGCTAAAAGGCCGATGTTAAGCAGGTAGTATTTTCACCAATTGAGATTAGCATAACATTTTCCAGTTGCCACT
TAAAGGGTAAATCCACCAATTTGACATTTTAAAGTGTGTTTGCGGGTCTTTGGGAGTACTACTGCATATGTGAAAAGTGAT
GTTACTCAGTGGCATTGTGTTGCTTTGTGTGTTGCATTGTGGGTAATTTAGAAAAATGTAAAACTCATGGTGGCACTAGAG
GAAAAGCGGGGGATTTATTATTACTTATATAGTTAAATAAAATGTGTGTATATGCGTGAAGCCTCACCATGGTTACACTTC
CCCACTTAACATACTGTAGGCACCACTAACATTCATAATCTAATAAGTCACAAACTCTAATGAGATGACTTTGCGAAACAC
ACGTGTGATGACAGCAGTCTTAAACATGCACACTCAACACCTATTAGCATAAAATATTAAAAAACAAATTAAAGATGTAAA
TTCCTTTTAATCTGCTGTTGAAAGCAACAGCGTCGAGAGCGTTAAAAGGCATTTCATATGTCATTGTGACACACAAACAAA
ATATTGTGCGTCCCCCTCATTAATTACTTGAGAATGCATCCACTTAAGTATCGTTCTGACGATGAATATTCATGGTATATA
AAGCCCAAGAATCAGAAGTACGTTGAAATTAATTCTCAAGAGGAGCTGCATTTTTCAGCATCTTGACAAAGTTGCGTCACC
CCGGAGCACGGATGAGACAGAGACTGCATTCAATCTCATTATGTGCAATTAAAATTTGACCTTGACAGATATCACAAGTAG
GCGCTGCAGAAAAAAACACGCCTGTGCCTCTTATTTTGGAAGCAAAGTGTTGCTATCAGAAAGTAATTACCTTATTGATAT
ACTCTCTGTATCTGAATCTAATCTCAGCGAGGCCCGTAAACCAAAGTAAATTGCATTTCTCCGCACGTTTCTCTATCACCT
GTGCTATTAGTTTCATATATCACTGCGTTTGATAACAAAGTAATATCGAGAATTGTACTACTATATCTCAAAAACTACTAT
CTTAAGGAGGCAGAACACAAAAAAACTGAGATCCGTCGCCTTATCTTTCATCTGTCTGTCTCCTTACACTTCTATAAAATA
TCTCATATGTAGCATTTCTCATCTTACCTGGTTACCGGCTGACAGGTGTCGAGGTGGGAGAAGGGCCAGACAGGTGCAAGA
GAGACGTCCAGTAAAAGATCAGAGTGTCTGCTCTTTCTGTTTTATACATGTCCTCTCTAACTATTTTGGGATGAAAAATGG
GCGCCCGATCCAGCATGGGGTCAGTGATGTTAGGCTGCTAGCCCGGCCCGTATCACTTTCCTGACTGTACTCATGCTAATC
AAACATTACTATTAATACTCATCAATCTCCACCTCTCATACCAGCCAGGCTTCTTTTCTTATGCTCGCCCTTTCTCGCCCC
TCCCTTCCTCCTTCCTCCTTCCAGCCATTTGATTTCCCCACCTCATTTTCTCCCACCTCCCACCCGAGGGACCTTGCTCTG
CCCTCTAATCCCCCTCTGATCCGATCTCTCCCCTTCGCTGTTTCTTTCCCTCTCTCTCCGTCTCTCTTCTCTTAAACTTTT
AATTTTCACTTTTAATCCTCATCCATCATTTCCTCCTTTAAGGCATTCTGCATCATGAGTGACAAATTAAAGCCCAAACCT
CCTCTCCCCTGTACCTTTGTCACTGTTTTGTTCTCGTACCATAATCTAAAATATAGACGGTAGTAATCTATACAAAGCCTG
TTCTGACTAATTATTACAATCAACAATTGAAATACACAAAAATGTGGGACTATAATGCACAATTCAGCAATAAGAATGTAA
TATTAGTGAGCTAGTTAGTTAGCTACTGAGACATCAGCAAACTGGTCGCGATTGTTTTATGCTTGTTACAAAAAAGGACAG
TAATAGTTTCTCATAACCCTCAGTTTGAAGCGTACCTATGAAGAATCAGTTTGTAGGCCCCACAGTCCACCATACCCCTCT
ATCCCAACAAGAACAGGGACACCCTACGCCTGTACGTGAACATACAAAACAGAGGGATAGTACTGAGTGGTAGGGGCAAGG
GCTGCATTGGGATTAGGCCCTGGTTGCTGTTGCTTCCCCTGTCAAGACTTCCATTCCTGCGTAGTATCGTTTACAGTGATA
ATGGAGCAGATTTATCTCATTTCTAGTCATCGGCCACTTATTTGACTGTTGCAATGACACTTGTTGCTGGTAGTAGTTGTA
AGTTGTAGCTAGCTAGCTAGCTAATTAAGCTAATGTTAGCTCTATGAGTTGGTTGAAAACAGTCTGACATGTATTGAATGT
TTCCATTTGAATTGAAGGGGGCCTTAAAGGTGCAATATGTAAGATTTGACCACCTGTCGAATTCAAAACAAATAGGGTGCT
GCATATTGCTGGAGTGATCGCAGCTGACTGCTGCTGACCGTTGCTGCTGTTAGCTAATTAGCTCTGTTAGCCATGCAGTTA
GCAGAATGGACTGGGAGAACCACAGGAGTGTAGCTTTTGACACCACAAGCACAGGAGCTTTAGAAATGGGTAAAACTATTT
CATCGTGTGGCTCTTCCAGACTTTCTAAATGTTATTGGTGTGAATGGATCAAATTCTGATTCTGGGTTGTGATGCTAAAAA
ACACGTGACGTTGTAATTACATGGTTTGGCCACTATACCGAACTGGCTATAAAGCATGGCGCTCTTCCTGTGGGCTAAGGA
AACCAGTTCTACTTTACAGTAGCTTGGTTACTAACTGGGTTAGCAACATAATACAGACACACTGTGAACCAATATGAGGAA
TGCTAGTTAGTCGGACATGCTAACCCTCGCAGCTTATATTTGAGCAAACATACACTGTTTAATCACTCATTTGAATTTTTG
CTGTTGTGTTTTTGGTTATGTATAGGCTTAAAAAAACTATTAAAATGTAGAGTATCACTCTAAGTACCGACGTGCGGCCAC
CACTTCGTCCTGATGGGAAATTAGAAGCTTGACAGACCTGAGATGCCTCGTTAGGGCTGCTGAGTTATGAGTGGATAATTG
TTCAGGGGAGTGGTTTAGCGAATGGTTTAGTGCTTTCATTTGGATCATTTTGCCTCGGGGGTTGGATTGGATTAAAGAAAA
TGTTTTTGATATATGTGACAGCAGTAGATAGAGTAAAACGTGCAGAGATCCAGAAAACCTCTGTGGTCCGCTGTGCAGGCA
ATAATGACTTCGCAGAAAAAGCATCTGCAAGAGGCTCTTCATCACTAACCCCAATAATAAAACACTCATTGCCATTCTGTA
CATCCCTCTGGGACAGTGCAGCACTCCCTGCCCAGGATTTGTCCCTGAGGCTCACTTATAGATGTAAAGTGGTTATAGATC
ACAGCAAATATCAGCTGTTTCCACCAGTGGGATCACAGAACAACCTCTCAGGCTGACCTTTAGGAAGCGTTGGTTGGGAGA
GGCCGATACCAGACTTAAGGGCTACTGTCGGTACGTGAAAGCCATAAACAAGTTTTCTTGGGAGGGATGGATTTATGTGTT
CCTGAGTCTCCGTGCACATACTTTGAATGGTATGGGTGGGTAGGAGGTGGAGCTACTTTCAACAACAGGTGGTATTATCTC
GGTTACTGTCAAGGTGTCGTATTTATGAGGCCGCTTTCAGTTTCATGACAGTTTTATAGATGTTGGGTTGGTGTTTTTATT

TTTTTTTAGGCCAGAATCATTTTCAAAGTGGCAGAGATTTATTATCTTTGCAACTTAATCATGAAATGTTTTATAGACGCT
CGTAGATGCTAGGGACGGTCAGAACAGGAACAAGATGAGTAATTTGAGAAAATGCTTAAAATGATAGATGACAAAGCCCTC
TTGATGAGTTAATAGAGTCTCTCTGGGGAGCAAAAACGGAGTGTGAGGTGGTTAAACAACCACAAAACCTCTAATAGGGTG
TTCGGACTATAAATAACGCACACTGGAGACATTTTAATTTTGAGACACAACTGAATTAGTTTTAAGACTTATCAGAGGCCA
AAACTGTTTTACTTTAACAGGTCTATAATACTTTGCAGGAGGATATTACATCTCATGAACATCATCACGGTTCAATCATAA
CAGCAGTCTTTTATGGGGTGGAATGGGCGGGGCTTGAACTGGGAGTGGGTGTGGTGTTGGTATATTTATTATATATTAGAA
AACTATTTAGACAGTTTTTCTTAAACTTCAATATAAAAAGTTCAGTCAGCCACCCAATGAGGATTTTCCTTCATCAAGAGC
ACAGATAAACATAAATGTATCCACCAAATACATTCTCTGTACTCGTTTCAGTTGAGCTCTCGCCCTATCACTTGTAGAAAT
ACGGCATTTTTATAAATACACTGCTTTTCTACTCGCACTACATATTGGCACCCTTGTCGAGAAAGCCGCTATTTTGTATAA
TAGTCTGTCCAAAAATGACCTGAGGAGATGGTTGGAGGTGGAAAGTTGGGCAAGATTCCGTCAGCATCCTTCAAGCCCCGT
CTTTTACGTCACTCAAACCACAGTCGTTCCTTCAGTATTTTTAGTTGCAGATTGGAAAATGAGTCATTTTGGGAACAATCA
CAGGGGTAACTTTGTGAGACATTGACGAAGGACCTCTTGGTATGAGGACAAATTGATCTGTCTAGCTCCATGAAACTTGTC
TCTTTCTGCTCTTGTTTCCCCCCTTGGCATGACTGCACAGGGACATCACTCCCAGTCCAATTCACAAACCTTCCCCTCAAA
GTCCTCAATCTTTTTTAACCGTTCCAAGCCCTTCGTTCCTTTTCATCTCTCCCCCAACCATTTTGATGCAATCTCACGCCG
AGTGTTTCTCTCCCTCTCCCTCCTTCTATGCATTCGCTAGTACACTCCTCTAAATCTATCTCTTCTCCAATCCCCAGCCAA
AGGGCCGCAGAGGGGATTGCCTTGATCCCATGATGTGCCGCGGTGCCTCTTGCAGTCCTCTTTGCGGCAACAGGGTCCTCA
GCGGCGGCGGGGGTGGGAGGCAGAAGGGAAAGAGAGAAGGAGGTGATGGAGTAGTGACAAGCGAAGCCGGGAGGGGGAAAA
TGGTAAATGATGAAAGAGGAGAAATTAAAGAGGAATGGCGAATGAGAGATGAAGAGAGGAATGCCAACAGAGCATAAGAGA
GAAGTAAAAAAGGGAGGGATGAGGGCAGCGGAGTGGTTCGAGGCTCGAGGTCGACAATGGATCTCACCGGGGAATGTGGAT
GTTAGATGGGGTTGTGGACCGGGGCGGAGGGTCGGCATGAGAAAGCAAAATTCAAAATAAGGAAGAGAAAATTCAAATCAT
GGGTGAGGTTTTATCTCACCCAGAGCCGGCACAGCTACGGCCTGACAAGGAAAACCATCAGTGGCTGCTACCAGAGACCAA
ACGGAAGACTATTTTTGTCTTCTATTCCCTTATCGACGGAGACACGGCGTACTTAATAATCAACACAAACGTATCTAAACC
AAACAGCATCAGACTTTCAGTCCCAGGAGTCCCACTGGCTTCAAACTATTTGATTATGTCTTAATCGCTGTCATTAAAAAG
ACTTTCAGCCTGTGTGCTTGCTTTGTTTGAGATCGTTTCAGATGGATACGAGAGTTAGTTGTGTGCCGATCGACTTGTTCA
GTTACAGCGTGTGTTATTTGCCGTCGGGGAGTTCTTAGCAGTCATGCAAACCATCACCAGATCTTAATCACACAAACAAGG
GCGGCTCATTGTGAAGCTCTCCTGAGGGTGTCTCACTTCAGCTCGTTAGTCAAATGACAATCAAGGATTTGTGCTGCAGAA
CAAGAATGTAGCTCAAAATCCAAAACCATATGCTAATTATTTCTCAAGTTTGGCCTTGCATTTATCTGACTCTCCATCTAT
AATACTACTGATTAGCTTTGTCACTGTCTTCTTTCAGCATACAGACTTCTGATTTAACCTTCTCCAGCACAGTGAAAGAGT
CAAACATGAGATTACATTCATATGACATCCTATAACTTAGAACAAAGGGAAGTTAGTCTGGGATCAACTGAGAGGCTAAGA
GATAGTATGAGAAAGAAGTCCAGGGAGGACAGGAACAAAGAAAGAGTTATGCTGATAAGAGAAGGCAGGAGACAGGAGAGA
GAGGGAGGCTTTTGAACCTGAGTCCAGGTAAGCCAACATAATGCAGACACATTGTGAACCTTTTTGAAGGTAGCATCGAGC
GACAGAGACACAGAGTTCGCTCCCAGCCATCTGCTCTCTGCACATTTTGAGTCTGCATGGACATGCCAGACTTTTGATAAA
ACACCAATCACAAAGCGTTACATTAGGCTGACATGTACCCATCTTTTTTCTGTCTTCTCTCCTTTACACACTTCTTTCTAA
ACCACTCAGACTTTCATCTATTCTTGTCTTGTCACATTATCGCCGTTGTCACCTTAACATTGTTTTTTTTCTATAATCTCA
GCAATCGAGTTTCATGGAGCCTGTATGGGCCAAAAAACTGCATAAAATAGTGGACAGAGCCACCATGATGTCACCCATAGG
TTCCTGAAGAGCCAGGATGAAGCTCAAAGTGGGTGGTACTAGCTGTCTGCACCTTGGCGGTGCCTGACTCCACCTAACTCC
TGGCTAATCGAAAATGGGCAAAGAGGTGGAGCGTGGGTGGAGCTGAAGCGATCAGAACTCAATGCCATGCTTCGTCCAAAA
AGTAATCCATTCATTGCAGTAGTATGTACTGATAACTGATCTGACTGCTGTGGAGAATCATCACGGTTCTTTTTTTTAGAT
AACTCAACTATCAAAAAACGTGTTTTAACATAAAGCATTTAAACATTATCTCGTAGACACCCAAAATAAAAGTATGAACAT
GAAAATGAGAACAATATGAGGCTTTTATAATTATTAATAGGTGCTTGAACGTCGTCCAAAACAGATATTAGCTGCTCTCAT
ACTGCTGCACTGACTCAACTCACCTTGTACTTGGCTTCTATTCACACTGGGACAGTTGTTATTCATCAGACTGCTGATACA
GACTTCTCTTATTAATGTTGTAGCACTTATGGTTTGTACAGCACATTAAATTACCAGCCTACTTATTAAGTGCAGTGGATC
CACTTATAAGGGCATCTGCTAAATGCCTGGATAGGTATGTGATGGTGGACTGAGTTTCCAGGTCTTTGACTTCACTTGTCC
TCCTCACAGCAGGAGGACTGTGAGAATGATGGATTTTTGAACAAAGTCCCAGCATCAGCATCTTTCTGACAAGGTTTTGCA
TTCAGATAAAGTCAGTGCGCTTGATTTATGTAGTTCAGAGAGGGCTGGCGTTCGTGTTGATTGACACGGACATGATAGAGG
GAGAGAGACACGATGAAGAAAGAGATTGCACGGACAAAATGCTGCAGTAGAGAATAACTTCGGTATAAGTGCGTCTAACTG
GAACAATACCCGAGGACTTTTCAAAGCAAAAAAGCATTTAGATTATGGTGTTGTGCTTACTCGTAAATAATGTCATGGGCC
ATTTGGCGATCAATATACCAATACTGATGAGAGGAAATAAAAGATTTCACATTGTACTATCAACAAATCAACTCTATGTGC
TGTGACAGCCTCTGCAACGAACGTCATCTCCATAAAACGTGAAAATATGTTCTATTGCCGAGGTGAAGACACACTTTTTCT
AAAGGGCATCATGACAGACACTCTGTTGCTGTGAGTGAGAGACGTGCACAATAAAAAGCTGTTATATGGCATCATCTTTGT
CCGCCACGTACACTATTGTATTACTCTATCTCAGCATTTTATTCCCGTCCGTCTTGCACTGAAGAGGTGCAGAGTGAAAAA
AAAAAAATGAGTGGACTAAAAGGACGAGGGAAACATGAAAGACAGAGATGGATGAAAAAAATCAAGAAGAGAAAATGGAAA
AGAAGATAGATAGATAGATAGATAGATAGATAGATAGATAGATAGATAGATAGATAGATAGATAGATAGATAGACCTGCTT
CATCATCATTCTTTCTGGTAAGTATGAAGGTGTCAAGTCAAATCCCCCCTCTTACACACACTATATAATCAGACACTTTTG
GTGCAAAACACTCCTCGCTGCAGGGCTTTGCAGATATTGGACCGAACTCAGCCTCTATCAGAAGTACAAAGATACCAAGAG

ACCCCGAAGACTGAAAGAATGATTGACCTGTCTGTATATACACAGAGGAAGCCGTGACAGGGAGAAGATAGGATGATGGAG
GGCAAAAGAAGAGGCAAAACGCTGCACAGAGGAAAAATTGATTTGTTGGATGGGATTTAGGTAGGAAGATGTACGAGCGCA
GTGACAGGAGATGAGTCCGTCTGTTGCTCCGGACAGTGTGGAAAATGAGATCTCTCAGGTCAGTGGTGGAACTACCCAAGC
CGTGAAGTGTCAAGGTTGTTCTCATCAACGGCGTCTTTATCACTTTCATCGCTATATTTCCACTGTCATCACTGTTGAGTC
GATAAGGTTGTCTTGCTGCAGGCTGGAGATTTCTGTACTCATTTTAAAAAAACTAGCATACAAACGTACAAGGAAACAGAT
TTGAAGGTCTCTACAACAAAGGTCTCGTTGACTAACTCAAGTTTAGCAATATGGTTGCACCGTCTTGTTTTCTCTTTTTTA
ATTTTTTGAGCCAGAAGTGAGTATATTTGGATGATAGGATGGAGCCTGGGAGGATACGCATTGATCACATCTTTATCCTGA
CAGGGAACCCCAGGACACAGCATTTATTGCCTTTCTTACTCTAAATGGGACCTTAATTTACAAAATGAACATCATGCTGTA
TTGAAGAAGACTTGCAACTAGCCATTCAGTAGGAAACTGTTTACTGAGGTAATAAATCAAGTCAAGAGTAGGGTCATTTTC
TCATAAGCTTACATACAATCTGACTTTTTTTGAAACCAGTGAAGTTGCCCCCTGCTGGCCATTAGAAAAATGCAGATTTAA
GGCACCTATTGCATTAGCGACAGTTTTCAGATCAGGAAGCTATTTTTTCAACATCATCAACATCAAAACCAGATAATCAGC
GTTGTCATGATGGCATGAATACACACAGACATATAGTAACAGTTCCCCCCATCAAAAGGCTATAAAACACAAGTGGGGTGA
TAGATATACTTAGACAAACACACACACAGCAGGAATGCTGAGGAGAGCTGCAGTGTGAGGGGCGTCTATATGAGAGACGGG
GGGTCACTGTGTGATTCACACAGTATCTGGTGCCGGCCTTGGTTGGCAGAATAGTGTGAACTCGGCAACAGGTGTCTGGGA
ACAGTTGACACTGACTTCAGAAGCGATATGAGTTCAAGCACCATGATATGTGAGTCGATGAGTATTCAAAGACAGAGAGAG
AGGAGGGGAGAAGAAAGGGAGACTCAACTTTTTAACCAAGTTTGAGAAAAGTTCAGTTTTCAGCCTCAATAAAAGTAAAAG
TAAAGTTGGGGTTAGGGTGTAATTGATGGAAGGGGTGATGAATGAGGAAGAGGAAGTATTTGTGAGACAGCATGCTGGGAG
GCAAAACTATTAGAGAAGACAAGAGTTCAGGAGGTGTGAGAGCGAGAGAGAGATGAATGTAGATGTAGTGGTGGCGCAGGG
AGGGTTATCACTGAGTAAATGAGGGTCTAAACACATCGTTTGTGGTCACTGGCGTCCCCAGAGGAGGTTTACAATATCAGC
AGCCTAATCGCCGGCAAAATCTCAGCTCGCTACTTTTATGTTCAGCAGTTAATTTAAACTGGGGGAAGCTATTTAAATTAG
ATTTTTATTTCCTCTAATGGGAGGAAATGAAAAAATGCTTTCTGAAAGATAAGCTGCTCAAAGTGAGAGGAGTTCATAAGG
GGGTCATATGGGACAATTTTCAAACTTTATTTCATATTAATTAGAATATTAAATTTAGTTATGTGATGATATTTGAGTTAT
GTGATGTGATAAAAACTGGCATAATGTAAAAAATGTTGTTAATAAGTAATTCAAAGGAAATTTAAAGCAACATTATTTAAC
TTTTAACCTTAAAGGAACAGCTTCAAAATAATGTTGATGGCACAGTGTGTTGTAACAGGGTGAATGGTGTCTCTGTCTCAG
CCCCCCATCACTTGTTTCTGCACTATGTAACTTCAGTGAGAGGGTAGGATCACAGCGATACATACATGTTTTCAACACATA
ATATGCAGGTAGAGTAAGGAGGAGAAACACAGGTTAGGGTAATAAGAGGAGAAACACTGGGAACACAGGAGGAGCTGATAG
ACTAGAGAGAACACTAGGAACATGGCTGGGCTGATGGAAGAGATGCAGTGCAGGTGTGGAGGGAAAGCAGACTGGGGATGA
GCACAGAAACACAGGAGGGAAAGAGAATAAAACACATGACAATGACCCAGAAAACACACAGAGCACAATTCAAATGGACCT
GAAATGACATGACCACAACAGTGTGTATTCAAACGGAGATCACATTGGCTGCAAACATGATGTAATTCATGTGCAGTCAAT
GTGACGGGTGGGCAGAAAACAAGCTGTAAACAAGTCACAGAAACGGGATTAAATGTTTGAGTTATGGTTCATACGACATTG
ATCTGAATTTAGACGCACATGTACATTAATCTGAATGGCAGTGTGTCATTCCTTTATGTACAAGCAACTGTCGGGCATGGA
GATTTACAGCATTTAAAGAAAATGGCAGATGGCAAATGCACGCATGGATAAAGACACAATCGCACACTTACATACATTGCA
TACATACACATAACCATCCTCCTACTCTCAGATGCAAACACCTACACAAATTGGCCAACATATACATAAACATGTAAGTAT
GCGAGTAGACCAGCACACATACATGTTATATTCTAACACTGAGGAAAGAAGTGTTTGACCTTGAAGTTTTTTCTATGGTGT
GCGTGGGGTGTGAGCAGTAAAGTATATCGAATTCCAACAAATCACTAGTGTCAGGCAATAAAAAAGAGAAAATCTCTGTAG
ATTATTCCCCCCCGTAAATCCTCTCCAGCTTGAATCACATCAGCGGCACATGGCCACATCTGCACATTACGATAATAACCA
ACAACGACTGTCTGTCACAGACGGCCTGACGTTCATGTGTTGCTTTTGGTTAATCCAAGGGCAACTTGGTGTCACCATGCT
GCTAAATGCTGCGCATTATAAAAACATTAGAGCGATAATAAGGCTGAAAACAGCACCTGCCGTGCACTCTCATCCTGCAGA
TGTGCCACTTTATTTGCTCTTTATGGACAGTATACGTTTAAGAGTCATTCGTAAGTGGATGGTCCTTAACACACATTATCC
TAACTGGTGTGCGCTGAAAGCCAGCCGCATTGCAGTTAAGATCTAATTTAAGCCAAGGACTCACTCGGGTGAAGGATTCTC
GTGGACAACACTGCAAGACTGCAAAGCGCAAAGGAGGAACCTTAATCATTATACCCATCAATCAATTAGACAACAAATTGA
AGCCATTAGTGTACATTAGATAAGGCTAGTGTATGTGTCATTATGGATCCCAATGCTTTTCTTGGCCTGTCCTCCCTCTGC
CTCTGATTGGCTTACCCTGATATTCTCACCCCAACCCAAACCAATCTCCCTCCTCATGCCGAAACCTAACCAACCCAACCA
ACGAAGGCAGCCAATCAGAGGCAGAGTAGGGCGGGTCCTAACATGAGAAGCAGTGGGATCTATAATCACGCTTTGTGTATG
CGTGTGTGCACGTGTGTCTCCAGTTTAATTGTGCGCTCATCCATTAACGTTCATGATGAATGCACATAATGAATGGTCATG
TCTTCAGCGCATGTTAATATGTGTTGCTGTGTATGTTTGTAGATTTAATTCAGACTATAGAGACTGTGTCATGTGTTGTCC
CGAGTCAGAAACGTCCTGCCTGACGTGTTTTCTGGTCTTTTTGCAGGGATTTTTATCGGGGCGATCCCCACTTAAGTGTAA
TTGTCTGTAATTTATACATGTTTAACAAGCTACATGTTACACTTCTGTATGTGTAGGCTACATGAGAATGTACAGGTATTA
TAATGAATGTTTCTTTCATCAGAGAAAGTACTAAGAGCTTTTAAGTCGCACTTGGGAGCACTATTTTCCCTTTTTAAAAGT
ATTTTTGGGTAATTTGGATTATCTTATTGTGAAGGACAGCAGTGATGGTGGTTTGGCCAGTCAACGTACAGCCAGATAACA
ATATCTTACCTCTCCTTTTATTTAGCTTAACAAGGCTGTACATGTTACAGGCCGGCTAAGTAGCCGAGCTACAAAAGGTTT
AAGAGTGTTAGCTAATTCAGTGTGGTTAATTCAGACCACGACAGCATTTTTCAGAAGTTTTTCAGAGTCGTCTACCAACCA
GAGCTTTCTCTCATTTAGCCTTCCCTCATTAACATACAGCAAATAGGGTGGACAAAATAATGGAAACATGCATCTGTGGCA
TTACAATACAACTGCTGACTCCAAAATGGCGGTACATTTCTTTCTCAAAAGAATTATGACCTTCATGAATATATGATTGAT
TGCCGGAAAGTTGTATTAGATGGAAATATTTAAGCTTACTTGACTGTACCTAATACACTGAGTGTATCCATGTGTGTTGGG

TTGGGGTCACGAGAGAGACACCATAATTAAGTCAAGTTAATGCACTGGCATCTCGTGTGCATCGATCCACTCCCTGCTAAT
GTCCCACGGCGGCATGACTTGTTAGACTTATTAAGTGCAAACCTACAACCACATGATCCGATTAGATAAAAAAGCATTCAC
TCAACCTGCATGCTGAGCCATTGGTGCTGTCTTCGCATGCAGCAGCGGCGGTGCAGTAAAGATGGCTAAATGAAGTCAAAC
GATGGGAAACTTTCTCTGTTCTGAGGAGCCCATAAACGGAAAGCCTTCTTATCTTTTTGTAATGAGAAAATGTCACACTGT
GTTTCATTTTAAGCCCAGCAAAAACGAATTGGTTTTATTTTAATTCCTGGATTGAAATGAGTTCACCCACAACAAGCTGCA
TTGCACTCTATGGAGGAGATAGTAGCAGAGTGTCGTTATAATTTAGCAGTCTGGGTGAGGTGTCGCCAAGCTTTAGAACAA
ACTCTGCATCATTCATGACTTCTTGGGAAACAGATGTGATTAATCATTCGAAGAATGGCTTGCTCTCATTATTCCCTCAGC
AGAGATACATGAAAGTCACAATTCACCGAGACTGCAGTTAAGAGTCTCACCTGTATCAACTTGGATTTAATCATACAAGCT
GACAAATATAAGGTCAAATATTAACTAAATGCTAAATTATAATGAAACAGTGACTCTAGAGTGTATGTAGCTGCATGGTCT
GATGTTGCCGTGTATTGGTTTTGGCCGTGAAAAATCTAAATGAATGTTGCCACAGGGCCATCATTGAAACTGGCCACATTT
TGGGATAAGTTATCATTATTATTATTATTAGTTTATTTCATAGGGACAGTGCACCACTAATCAACAGAGTTAAAAGAAAAA
CTGTAAGTGGGCCAGAGTTAGCCAACAAGGCTAGTTTTCGTCCGTTGTCCCTGGCCAGATGTTAAAGGCAACCTAAAAGCC
AAAAAAAGGGGCAACAATTAAGTACTTTCAAGACACAACTTACATAACAATCTAGTAATCTATCTAGTAGTAAAAGCTCAT
AATAATAACAAGTAAAACAGGTAAGAAGTCTGACATACAACATGACAAAAGCTCATGGCATGCAGACATAACATGCAGACA
GAGAGAAGAAGAGCGAGAGGAAGCAAGAGAAGAAAGCACAGCTAGAAGCACATGCAAGTAGCACACAGAGCTAAAACATTA
GCACAAACAAACAATATCAGACATTACAAGACAGGACATGAAACGGCATTAGGCAAGAGAACCAAAGCTAACAAATAAGCA
AACAAACAGAATAAAAAACAAAAACAAAAACAAAAACAGGACATATGATTACAAGCATTGAGAAACAGCACAAATAGCCTA
ATGATTGATTAGTGATTTACATGATTAGTGATTTATATGATTAGCGATTACATGATTAGTGATTACATGATTGATTATTAA
TTAACCACTGCTTTAACTGTCTTTTAAAAGAATAGTGAGTGTTTGATTCTCTAATAGACCCTGGTGTTGAGTTCCAGTCCT
GCACTGCTTTTACTGAGAAGGCAGCTTGGCCAAAGACACTTTTTCTCATGGGAACAACACAGTCTCCTCGAGCTGATCCTC
TGGTTGCCCTGTCGTTGATTGATCTTTGGGTTATGAATGTTTGGAGAGGAGGAGGAGCAGTATTGTGCAGTATTTTAAAGA
CCAGGCAGAGATTTGCATACTTTATTAAATTATCCCAACTCAGAAGATCATGCTTTTTTAAGATGAGGCAGTGATGGTGGG
AGTTAGGTTTATCCTTTCAAACATGTTTAACCCTAACCCGTGTTTGTGAAAGGGTATATTTTAACCCAAAACCTGTTTTCC
TAAACCTAACCAAGTATTTTTGTAGCTGAAACCTAACCAAACAGTGACCTAGAGGGAACTTCACGCAAGGTCACACTAGGG
CACTATCATGTTTTAAGTACCACAGGGGCATCCAAGAGGGCACTTTCGCATTTTTAACATGCTCGAGCAGCATGCTTATAA
ATGGAGTTATGTGAGTTGAAATGATGGTGCTGGAGCAACATTAATTATAAGGATCAAGCAACAACACCAACACAAGCTGCA
AAACTATGACAAAAGAACAATGTGATTGGTCAGTTTCAATGAACACTAACACAGCAATATCAGATACACCATATGTAGGTA
TACAAGTTAATGGTTGGTGTCGATGTTATACAGACAATGGAACGGTCTCCTAAAACGACAAATTTTACCGTTCCCAAAACA
ACATTACCGTTGCAGCGTACCAGTGCGATGTTTGGTTGTGTTTAGGTAAGAAAACTACTTGGTTAGGTTTCGGAAAACATT
GTGGTTTGGGTTAAAATCACTACATTACTTCTGTTTCTTTCCTTACAAATGTACCTATGTCACCTAATTAACACAGTTACG
TTATGTATGTGACGTAACTTCAGTACGTTAGAACGTGAAAAGAACTCACCGTTGACTTCTGGTTTTACACGGGACATGAAC
AGCAGTCTCCAGAATTTAAGTCCTAGGAACTTTTCACTCTTTATACTACTTCTCTACACTTCCTCTTCTGTAGCAGTAATA
ACTACATTCCAAACGTACATATGGGTCGTGATAGGCTGCCTTCATTTTCAACCTATATGATCGTTTTTCTGATGAGGACAG
GCTCAGAAATCCCAGCGCAACAAGTTGGCCAATGGGCACTTAAAATATTAATACAGTGAGATATTGGATTGAAATATAATT
ACATACTAATTAAATTTTATGGATAAGAAGATGAGAGAAAAGCAAGAAGAAAGACGTGAAATGAAAAGAAAAGAAGAAAAG
AGAAGGGTTGTTTAAGAAAACTGTTTTGTCGGAGCTCCTCTGTGGGGAAAAAAAGAGCATCCAACAGCCTCGGGATTCAAT
TCCACCTTTTTTTTCCTTCCATGCTTTCATGCGTAGCTTGCTCTGGATCAAACAGACTCCTCCAACTGTGACTTTGTTTGC
CCCTGGCTTAACCTTTGGCCTGTTCATCCCTCTACACAAACACACACACACACGCGCTCCCATCCACTGGTACTTGTCACA
TTAGGTTCATGACTCAGTTATTATACATTTACACCAACCGCTCTGACCTGCTACACGCTTCACTACAATAACACATATGAA
GCGCACACAAATACAACAAACGGGCAGTGACCTCATGGTGCTGATGTGTAAATCTGGCTTCTATCAGCCTCGGGGAAATAT
TACATAATGTACCGTATATTATATACGTTTCCAGCAGGATGGTGGTCCAATTAACTGTTCATGTGTGCATGTGGGCATGAT
TGCACGTATACAGTACATGTGATTCTGGTTCAACAACAGTGTCATGCCCGTGTGTGTGTGTGTGTGTGTGTGTGTGATGCA
TCTTTCATGTGTAGGCTGAACAATGATCACAATCCGTGTATTGGAATCCATTACTCTATAACCTCAAGTGCAACGATAATG
TAGGAGTGAGTCAGTCAGCAGAGCAACAAAACATCACACTGCAGTCCTTCCCCTGGCCATCACGGTGTGTGTGTGTGTGTG
TGTGTGTGTGTGTGTGTGTGTGTGTGTGAAGAAAAAAAGCACAAGGCTGCGTTCACAAACTCACAGAGCTCGTGAGAATGA
ATAATGATCAATACTTTGACCTTTGTTGTTTCCACCTGAAGGGTATCTGCTGCACCCGAAGTAGTCAGCGCTCAGCGGGCT
GCCTCAAAACTGAAACACCTCGCTATTGCATGTCAGCATTTACACACTTGTGTGTTTGTGTGTGTATGTGTGTGTGTGTGT
GTGTGTGTGTAGGGGCAGCACACATAAGGCTGCAGGCAATACTGTACTTCCTACATACTGAACAGCACTCCAAAGACTCTT
GACTCTGAAAGAGCTGAAGGGAACGCAGATCCTATAAGACGATAAGTGAAAGAGAAAGCGAGAGCGGGAAACAAAGGGAGT
GAATGGCAGAAGGAGGAGGAGAAGAAGTGTTGGAGTAAGAGTGAGTCCCTCTGATCGCTGGGCTTTGGTTTATCATTCATT
CAGTGGCGGTTTCTCCCTCTTCCCTGCATGCTTAGCAGCATTATCAGCAGCTGCCAGGAGTTAGGACGCACACACCAGGTC
ACACACACTCCCCCCCACCCCCCACTCTCTCTTAAATCACATCACACACACACTCAGAGACGAACTCACACAGAGGATTTG
GGTTGTAGTAGTTGTTGAGCCGGGCCTGCCAATAAGAGCTCCTGACATGGAATCAGAGGTGACAGATGAGGCAGGTGAGGA
GAGGAGCAGGGAGGCGGGGGAGGGGGAGACAAGGAGACAGACAGGGGGAGTAATCAGGAGAGGCAGGTGATGCTGAAGAGG
GAGGAAGCGGGACACAGACGGTGGAGATGAGACATGAGAGGCGAGAGTGGAAAGGTGGAGGTGAGAGAGGAAATATAAGAG

GACTGGTGTCCCCTCATTCAGTGGGGAGAGATTACACTGCAGAGAGGAGACACATGACGGCCCTCTTACACTCCCTCACTT
GCTCTTCTTTTTAGCCACTCCTGCCACATCACTCTAGGGACGGCAATGATGGGCAATCGGTGGACCACTTTCAGACTGAAA
TATCTCAACCACTATTGATATATCTAGCGCCACCACCAGGTCAAACTTTTACTTTGACAGAAAGCGGGATCCCCAGCAGGA
TTCGACCCCAGGACCTTGAGGACCTTGCTGTGAGTGCTGACCACTGCACCCCCGTGCTGCCAGGAATGTTGATGTTTAAGA
TGGAAAACAGAAAGAATCCTGCGCAACATTAGCATATTAGCATTGTCATTGTGAGCATTTTCAACCTGAACCATATTTTTC
AATGTTCTAGTGTCTAAATTACTGATGGTGACAACAATTTTTGAAGGATTTTTTTGTGGACACAAGCAGCATGTAGAGCT
AGAATATTTTCTCATCTAACGTGCTGGACTATGTGTTTATTTTTGACCAATCCAGAGTGATTTTTGGAATAGTGGAAAGAT
GATTTCTTGAGTTTTGTTTTGATTTACTGTCGAGTTTGAATGAAGTGTTATTGACAATAAAATGACTGGTTCTGCAAATGG
AGTCAGGTAGGATCTTACTCTTTTAAAAGGTTTATTTCTGTAGCGATCCTTTCCACGATGTTGTCAGTTTAACGATCTGAG
CCTGTTTGTGGCAAAAACATACACCATCTTTTCAGACACTTTCAAATGTACAATTTACGTTTTGTTTTCCTGTCACTTGTC
TCCCTTAAATGGCTTTTTCTTTGAAACTCATCCTCAGCCTTTCTTCTTCTTCCATGTCATCCCATGCCCCCCCCCCCAACA
TCTGGTGCTATAAGGCCCTAGGAATGTGTTATTTCTTTCTTTCTTTCTTTCTTTCTTTCTTTCTTTCTTTCTTTCTTTCTT
CCTTGGAAAAACATCTGCCTCTCTCTCACACAGCTGGGCACAGCCAGCTGTCAGTTTCATCAGTGTATATGACGGGTTTCT
CATTTCTCTCATTTCATCATCAGGCCGTTCTCTATTCAGGTGAGACGAGACGAGATCAGATGCTGTGCCTCGACGGGCAGG
TGGCTCTTTGCATCTCAAGTAGCTCCTGGATGCTTCCAATCAAGCAGATACCACCAAACGGTCAAGTCTCTGATGTTGGTA
GCATTCGCATTAGCCCATTCTGGCTAATTGTTTCAAGTCCTCTAATACACTCACAATGCAAAATATGTTCAATGCTAAAAA
TCAAAGTCAAAGATTGCACACCTCCTCTTGATTTACATGAATTTTGAGGCCCGTCGTCCTTTAAATAAACTCTCTTTGATT
TCATGACTGAATAAACTGAATAAACAAACTGACCTTAAAGGACAACTTTGTTTATATGTGGCGGACCCTGCCACCTTTCTA
GCTTCAAACAGTGTTCTGGGGACCATATTTCCCTCTGAGAACAGCTTGTTTATTCAGTTATGGAAAGGTTAAATATTTCTT
ATTTTGTATTATTACCTCATTAATAATGTAAAGTTTGAATTTCTTCTCCAAAAAAACACATAGTGCCCCTTTAACTTTGAC
CTCATCTTCAACACAGTAGTTGTTTCGTTTGACACCAAAGTAAAAGAATCAACCTGAAGAGGAAACTTTCTACGAGCGACT
CTTCGCTGTCTCCAGATTAGAGAGTAATTACACACTGCGTCAGTCCGTCTCTGCCAGCTACACAATTTAAAAGATGCTTTC
TGGGGTGAGTGAGTTTGTGAGGGGGGGGTGTGTCCTTGGATAACTTTTATCCATCCAATGCCAGAATGATGTCAAGGTACC
TCCAAAGTAGTGCTCTGATATCATGCATTGGGCCTGCAGCCAGACAAGTCCAGTGGAGTGAAAACGTTGGACACAATGAGT
CTTTCCAACACGTTCTTTGTTTTGTGTCTTAGTTCAATCCTCCATTCTTGTTTTATTCTATCTGTTTCTCTCTTTTTTTTA
TAATTTTATTCATCCCTTATTTGTCTCTTTCGGTGGATACTCTCCATCCTCTTGAAGCTGCAGAGACAATATGTCCCCGCA
ACCCAAATTAACACCCTCTGTCTCACTGCCTCTCCATCCATCCCCCCCCCCCCCCCCCCCCCCCCCTCTCATTTGGCCATC
CTCCCCTCAGCCTCACTCTCTCTCTTCTTCCCGTCGACGACTCTCCTTATATTAATTTCATCCACATCTACTTGTTCGCAT
CATCTCCCCCCCTCTCTTCTCCTGGCACATTTTGCATCCCCCATTCTCTCACCCCTAAATCCTCCATCTCATCCCCCCCGC
CGTCCTCCCTTTTATCTCACTTCCTCCATTCCTGCGTCCCACCACCCCTCTGGCAGGATCATTTGGCCGCAGCAGAAGGGC
TCTCATACTCCAGGTGTGGTGCATTGAATGATGACCACAGGAGGACCGGGAAATGGCTCGTACACACACACACACACACAC
ACACACTGAGCAGCCATCATTGCACCAACCTTTCTGTGCCCAGCATTTCCCCCTTTTTCCTTTCCTCTTTTTCCAGGAAAA
CTATGTCATGTTTTGCCAGTCGGCCTCCGCTGAAGCGCCTGATTTAGACAAAAGTGTTGTCACACTGTACTTAATAAATGG
GATTATGTAATATTAACGATATAAAGTTAGTAAGCTAATGTTTTACATGAATATACACGTGCAAACACACACCCAGAGACA
CACAAACAAGCGCGGTCCAACCATGTGACTAATGTGTTTTCCATGCCATCTCTCCGTCCACCGGGTCCTCTGCCAAGCCAT
TACCATCCCCTCCTCTTCCTCCTCTCCTCTCTCTTCCTCTCCTTCCAAATGGATTAATTCATCTAGCCGATTAAACAGACA
GCAGCATCAATAGTTTCCAAGACTGCACTATCATAATTTTGCCTCCGGGCTCAATGAGGTGTAAAAATGGCTATTAGAACT
TGTTGGGTGTTACAGAAGAATCACTGTGTCTGAATGGGAGTTTCTGAATGGTGGTAATCTTTCATGGTGGAAATCAGTGGG
ACGCAAACCGCAGAGAAGAATTTCTGAATTAATTACTTTTTCTATTGATTTTTGTGTGACGCTGACTTGAGGGGCAGATTT
ATGCGGTTTCGGCATTTATCACAAAATGGAAATGGCTTCATCGTTAATAATTACAATCAAGCAGGAGTAGATAGAAAAGGT
TTTGAAACAATGCAGACATAATGAATCAAATATCTCATCCATGCAGCAGCCCCTTTACTCCCAACAGCCTTTCTTCGTCAA
GTTTATAAGGTGTCCTAACATGGAATTATTTGGTATTCATGAGGAAATCATACTTAACCAGATGTGTGAGATGGCTCTCTT
TCAGTCGTATCATGTACTTGCAGTGGCATTTTTTCTGTGTGCGAGAACACCAGTGTAATGAAACCACGCCTGAATGAACAG
AAACATTAAGATGAGCCTCTTCGGTTGCTCCACCGAGCAGAGAGGAGAATGGGTCTAAAATGTACTGCGTGTCAAGATGCA
GAAAAAGTTGAGAAGCCATCTTCGCTGGAAAATGTGACACTTCAGAAATAACTCTCATCATGGGAACCAATGACGCTATAA
ATGCCGCTGACGATACACCGGCAGAAATAAATAGAACCTCTATGTTAACTTTATAATATGGCTCACTGCTGCACTGGATTA
TATGTACTGGACAATCTTGCAACCCATCTACCTGTCTGTGATAACCATCTGTGTGACGAAGATTTAGCCTCTGGGGACAAC
AGGCAGCAGATATCCAGATAACAGGCATCCAGGGCAAAGACTTCTTCCGTGTGGCAGTCCGATTAGCAAGTAGCCGCGAGA
GAACTGAGAGTTGAGAGACGACTCCTATTTGTACATGAATGGTGATGGTTTAAAAAATAAAAAAAATAAATAGCTAAATTG
TTGCGAGACGTCCAAGATTGCATGTTGGTCAATGTGTGCCGTGACTGTTTCACTACATGCAACAAAATCCAGCTCAAACGT
GACTGGTCAATACTACTTGGACTACAAACAGACTACAAATGGAAACAAGAATGCTTCTGATGCGATATCTAGTCCCTTTCC
TTGAGATGATGCATTTGTAGGATGTTTATAGAGATTAAAGGGGGCAATATGTAAGAATTGTAGTTCAAAATGTTCTAAAAT
TTACTAAAATTATCGACAGAATTTAAAGAAAAAGCAGTTTTGACATTATGTATATGTTGCAGAGATATCTACTGAAGCTAG
TATGCTAACCAGCTAGCCATGGCCCATCCTGGTCCAAAGCTCCAATGCTAGCAGTATAAGCTTTAACCACTCCCTTGGAGC
CCGAGCTGACTGAGCTAACTAGCTAAGGGCAGCTACAGTTAGCAGGAGTTAGCGGTTACTATAGTGATATGCTGCCCCCTA

ATGGTTCTGAGTAAGAATTTGACTGGTGGCTAATTCTTACGTATAGCACCTTAATATGTGGATAATTTGTTGCACTTTCTG
ACTAGTACTTTCCTTCTCATATCCAACAGTCAGTCAGTGTCGACTGGCCTGCCCACATACATTTTAAGCCCTTGTTTTATT
TTCTCATTTGCTTGGTGGAGCGGTCGAGAAGGCTGTGAATGATGTGCTGCACGCTGTTGATGGTAAAGTCTCAGCAACTCC
TCAAGGTAGATCATAAAAATGTTGTTACACAGTTGGTTTTAATTCATCTTTTGTACTCACTTTACCCTTCTCAGGGTCACG
AGGCGGGGCTGGAGCCGAGCCAGGCATGGGTTGGGCGAGAGGCAGATGATTCTGGACAGGTCGCCAGTCCATCAACGGGGC
TGACACGTACAGTGAATGACGGACAAAACACCTACCAGCAATTTAGAATGATTCATCCAACCTGCCTGTTCAAGCCTCTCT
GACACAAACAAACCGAGGTACAACATGTTCGTACCACCACACAGCTTTAAGCATGCTTCTGAGTTGAATCTGAGCATGTTG
AGAACAAGATGTGATAGAATTCATCTGATGCAGATGACACACGTGGCCTTGTCTTTGACTGTGAAATGTGTGTTTTTAAAG
TGTTGCGTGTGATCGGCGTCGCCGTCCTGCAGGCTACACGTCAGTCAAGGCGATCTCCCCTCCTCTGTCTCTTCACTGCGC
CGCCTCATCCGTTTTACTGCCTCCTTCTCTTGTTTTATTCTTCAGCATTGCAGGACAGAGAGCCCGGAGGCCAGGGTGCTG
CCATCACACACACACACACACACACACTCGCAAACATCCACCCACCCACGCACTCACGCATGCATGCACTCTTAGAGAGCA
GGTACAGCGCACACACACAGATACAAAAAGGCACACTTACTCTACACTACACACTTGTGAATGATATAACTGCTGGACAAG
GCAGTGACAGGAAGAGGCACTGTCTTATAATTTATTTCAACGGGAAGCAGGTCCTTAAAACCAATGTCACGCACGCTGGGG
TATTACTGTTATTCATAGAAAAATTGCATCACAAATAACATTTTCTTTGATGAATTAATTCAGGAGAAAAACTTAAAGGGG
CACTGTGTGGTTTTGGTGAAGAAATTCAAACTCAGAATTTTAACATTTACAATATTAATGAGGTAATAATACAAACTCAGA
ACTGTTTCATTTTACCAGACATGTAAATGAATAAACAAGCTGTTCCCTGAGGACAATAAGGTCCCAGAACACTGTTTGAAG
CTAGAAAGGTGGCAGGGTCCGCCAAAGATAAAGAAAGTAAAACAGCATGAAATTGTGTTAAGGGTCAGTGTGTTTATTCAG
TTTATTCAGTCGTGAAAATTAAGAGTTTGTTATTTACTTAGTTGTTTGAGTTTATTTATTGAGTTAAACTACATAGTGCAC
CTTTAAATAGTGAAGCCAGGTGGAAAAGCATCAAAGTGTAACTTTCACCTTCATCTGTGACTGCACCGAGATCCACACTTT
ACCTCTCAGAGTTACACCGCGCTATAAGAACCTCTAACTACTGTTATTTGTCACTGGAGGTGCGACGTTATAGTTTACTGT
GGTGTCTTGTGTGAGGGCTATAGTGTTTGTGTGTGTGTGTGTGTGTGTGTGTGTCTCCCCGCTTGAAAAATACTGCAGTCT
TCGTCATGCCTGGCTGGCTCGGAGCAGGGTGATGTAAGGGCCTATTTTTTCCTTGGCTGCTTTAAAAGTGATGGAGTCCAG
TTGGGCTGAGCTTTGTTTAGGGAGGAACACTGAATCAAACGAGACAGATGTCGATCCGTGTGTGTGTGTGTGTGTGTGTGT
GTGTGTGTGTGTGTGTGTGTGTGTGTGTGTGTGTGTGTGTGTGTGTGTGTGGGAGCACTGGGGCCAATGCAACAACAAAAACG
GTTTGAGTCCTCGGTAAATGGGGTCAGAGAGAGTCATCACAGTCACATGTCTCCGTTTTGACTCACACACCTCTTTCAAGA
AAGTGGACCACACACACATAAAATATGAAGCGTTCACAGACACCCCCGGCTCTCTTGGTGACGATCCTCTCCTCCCTCTCG
TTCTTTCTCCCGGTTCCGCTTTGTTCCCTCTAATGTCGTCGCTCCGTCTTGCTCTCCGCCAGCCGCCCCACGCAGCGGCCC
CACCTGAGTGAAGCATTGACAGTGTTTACTCTTCCATCCAGCGCGCAGAGCGAGGGATTCTATGGGGTGAGTGGCAGACAG
GCCCGTTTGTGTTGATTTTAATGAGGGTTGATGGACTCGGAGGTCTGAGTCACTGCATAATGTCCGTGAGGGACACAGGAC
AACCTTGCCTAGTAAAGAGTAACGGCCCACTTGCTACGGTCGGTACATCACAGGTGTCGTCCTTGTCAGGAGGGAACAGCC
TCTTTTAATGTCCTGCTCAAAGGCACTTGAGGCAGATGCTTGTTCATGCAGAAAATACAGTCTTTCTACTCTCTATGTCAT
ACTGATGCCTTACTGGCTGAAGCAGGGGTAGACTGTAGTCACACACAAGATCACTGATGAATCAAACCAATTATCTTTCAT
TCAGAAGAGCTTTCCAGACTTTGAAAAAATAACACTGATGATGTCATCAGGCAGACAGATGCTACAGAGTTGCATTATGGG
AAACACAGGATGCAATTTTTTTTGGAGCTTGACCCATACCAGGGGGTCACTGGTGGACTCGACCACCAGTGACCTGACTTG
GCACTCCGAAGTTGGAAGAACAACTTCCCAGCTCAGGAAGTTGGAACATCCGAGGAGCACATGAATGCAGCATAAAGCGTG
ATAAGGTTTGATCACACACCATACTTCCAAGATAAAACCCTGAACCATAAATCAAATCGTAGGTGTCTGCAGCGGAGATCT
GGTTGAACCCCTCGCATCAAATATAAATTAGGCTCTCCACACTTGGTCAGAGGCACTATTTCCTGAATACAAAGCACGGCA
AAGGAAAAAATACCTGCATTGACGTCACAAAATGAGGTCAGGCAAAGAATAAGAAGTTTGCCTGATTTTGGGAAGGTGCAAC
TCAAACTCAGAGAGCAGAGAAGGAGTCAAAAGTTTTGAAATGAATCGCAAAATGAAGCTTCATACCTTATCTAAAACTCAA
ATAAAGGTATTTATTACTATTTCTCGAGGTGATGCTACATATTGATGCCACCAGTAACATCAAAAGATGTCTCACGTCCAC
CAAAAAGTAAAAAAGAAAAGTTTGATTCTCATTCAAGTGTGAATCTCTCACAGCACACTTCTAATTGCCCTTCATTTTAAA
GCAAGAGCCCGTCTGATGTACCCTGTGATAGCAGACTCAGACTGTACTGATGAGTTGTATCATCTTACAGGGAAAAGTCAA
AGCTCCATTGATCAATATTTCTGATCTGAACATGGAATCTAATGACTCGCTGTAATGCGAAAGGGTCACTCGTACTGCCGA
TCCCACTGTGAGTCGACACCCACACTGCAGCACTGAGCAGCTTTTACGGGAGAGAGAGTACGTCGTATCATTGAACAGCTA
AATAACCACGTCTTTGAAGACTATTCAACAGCCCGTCCTGTATCGAACACACACACATCTCCAGTAAACCCTTTGTACGTT
GTATCTTCCATTCATCTCTGCATATTCTCTCCCACTCTGTGTGTCTATCCCCACCACTCTTTGTTTTCTCGTCTTTTTGTC
TGTAGAGCAGCTTTACCCCATCTATTGTTGCCTCTCTTTTCTTTCGGCGGGACACATTCACTCCCGTCTCCCATTCTGCTC
TGGCCTACAGCATTATAGGCTACCCAAATAAAGAAAGGAAAAGCACAATTTGTATTCTGGACCACGTCTGTGATGCGGGAG
GAAAAAGGAAAGGGGGGAAAAAAGGGTGGGGAAGGCAGCGAGGGGAGGAGGGGTTGTTAAAGTCACAGTTTTGAAGACTGG
TTAAATACACTGGACGTAAAGTCAGGTGTCTCTGGGCTTTTCGCTTACATTGTCGATTTGTTTCTCCTTAAGGAGGGAAGT
TTGACCTTGAGTCAACAAGACAAAACACACTCATGCAATAGGAGGTACTTCACCACACTGTCATCCTCTTTATATCATAGT
AATGATTCTCATTTGTGCTGCTGCTCGCAATTAGGCATTCATCTCGTAGAGTACTATCAATCCATCTAATTTTATGCATCA
TTACACAAGCGGTTTGTATATTGACACATGACTTTTCTGCACTGCGCTTCAGTTCGCTGGTGGTTCTTCAGGATTCTCCAA
ACATAGCCTCACAGTGTATCCTCGCAGTTGGATAATGATGCGTTTAGCAGAGAGGGAGCGGGGATGGCTATCCACACATCC
ACACAGCGCTCATCGATACACTTTTTTTCCAGCGCTGGCGGCGTGGTTTGTGTACATGCATTTGGATCTGTATCCGCGTGA

GCGAGCAACGCCAGCCCGCACGCATGAGTGTGCACATGAATGCCAATTTGTGCCAGTATGTGCGTGTTGCCCCGTATCCCT
AACCCCGGCAGTGATGCCTGGTCAGCAGCAGCAGCAGCAGCTGACACCCTCTTGTCACTGACAGCAACTCCTGCAGTGCTT
AGAGAAAGCGAGAGACGAAGGGGGTGTAGCTCTCCATTGACCTTGCAGCAGAAAAGCCTAACAGTTACAGCAGTAGTAGCA
CACTCTCGACTGCTCATACGCAAAGACACCGGAGGAGCACAGACACACACACACACACACACACACACACACACACACACA
CATTCTCCAGCTCTCTCATGTTCATTCTGACACTCGGTCCACACGTTAGTGCTCAGGACTCGCCTCCGGCCTGTTTGCCTC
AGCCGGTCTTCTCACAAAGCACGAGAAAGCGAGCAGGATGGAAAATGGGTGGAGGGCAGGGGCTCGAGGATGGAATATGCA
CATTCACATAAGATGAAGAAATGTGCAGTCATTCATAAAGCCACAAAAGACAGAGACAGTGTAAGTGAGTTATAGCTTCAG
GGGGAGAAGTGAATAGAGACACACACACACACAGAGTCATGCACCTTTTTATGGTGGTATACACTTAGATGCATGTGTAAAGA
CACATATAAGACCATGTGAACAAAAACACCCTGACCTCCGTATGAATACAGCCTCTATGTAAGGTGATGTGTTTCACTTCC
ACACATACATGCACCCCCTCGACAAAAGTCGCTGTAAAAGTCTTGTCTCATAGAGGTGCAATATGAAAGCAAAAATAGCCT
CATCATAGCGTCATCTGTCTCCAATCTATTCCCTCTCACCGATGATAACACAGGCCTCAGCCTGGCGTCACCAGATTGAAT
GTGACACTGGAGTGAATGGGTCTGGTGCAAAGGGTGCAATATGGGATAAACACTGTCGCTGAGTGTTGCTTCTGCTGCTGC
CTTTCGTTTATCATGCTGGATCAGATCACTGAGAGGAATCCTGAATGAAAACCTCACACTATTTAAACCCCCGAGATGGAT
TTTATTGCTTACAAGCCACAGAGAAATACTATGCAAGTTCGATCATGTCTTGTCTTTGCTGTGTTTCACAGAGGTGTGCAG
CCTTAGTCCTCCATATTGATCTGTGACATTGAGACAAAATGTTTACTCTGCAAGCCCACAGGTCTGTAATCACAAATGCAC
ACTGTGAACCAACATTTACATTTCGGTCAGGTATCTGAGGGCATCCAGGGACATCAAATTAAATCCAGTGCAGGAGAGGCA
GATTCCACCACAAACAGTGAAAACTACCATGTTGAGAGATAATTACAGTATGCAAATGACTATCGCTAAAAAATGTTGATG
ATGGGGTCTGGTTTCATAACTTTAATGCTGCAGGATTAAAAACTAGAATGGCATTCAGTAGAGCGTATACCTCCACTAAAA
CCCAACAGTCGCATTTAATTCAATCAAGCCCAAACTCAATAGCCAGGAATCAGTCTACATTTTTAACTGCCTAATGGCTTA
AATATACCTTGATTTTTTTTTTCATCAAGATCCATCCATTATTTCCTGAGAGATTAAAGAAAATGTCCCAAAAAAAATGAA
TGGGGTCAATTCTGGGCTGAGACCCATCCACCTTCCAAGTTTTGTGGAAATCCATTAAGCAGTTTTTGTGTAATCCTGCCG
ACAAACCAACAAACAGACACAGATGAAAACCTCTTTAGTGGAGGTGTAGTCAATCAATCAAAACTTTATTGCAGACTCTGT
ATTGTGTCAAAAACAAATAAATTCACTCAGTTCATGTCAATTCACTCAAATAGGTCTGAAGACTACAAGTTTAAAAGAGGA
AAACATCTGGAGGGGTGGAGCTCGAAAGAAGTGAGCTTACCAGGCCCCTGTAGCCTGCCACTCATCTCTACTTTAGGCTAG
CAGCTCAAGACTACATTATCCCCTACTAGCATTAATACACCTGAATCTCCGACCAAACTGTTGGTGGCTCACTTGCATTGT
GGGTAATGAGTGCACCAGGTTTTGACAAGGAAGAAGAATGTGTGAAATAAAAAACTATGACATGTCTGGTTCTGCTGCATT
AATTTTGATCCTTCTTCTTTTTTTTTTTTTTTCAAGATATCCATCCAGTCCTGGAAAGTGGTAGATAAAACATGATAAAGTG
CCCTCTAGGGTGCTCTTCCAGGGCCTCTTCTACAGCAGACGCCCTTTTTATTTTTTCCACCGCTGCCCCTTTGTAAACCCT
CGGTGAAGAGCCAAATTATTCAGAAATGTTTTGGTACCCCGAGCTATTGCACATCTAAACAAAGTTAGCACGAAATGATGC
ACTAAACCCATGCACAGCACTTTAAGCTTTTGAGTGAGTAGGGGAGGGAGTGTGTGTGTGTGTAAAACCATGGAGTCGTGA
CCTTCTTAGAGCTCTAATACTTCGATACTATTTTTGCTACGCTGGTGATACTTTTTTTATGTCCTTTGAGTCTTTATCATG
GTGATGTGTCGTTTTAGCTCTCGAATGCTGAATCGTCAAACTGAGCAACAAACCTACCCTCATACTAATATTAACCTTGAA
CTGTTAGAAGAGTGCAATGCTGAAATGGTTGAGTATAACTTTTTACAGTGATTTTCCTCCAGCGTGGCATATGCACAAATA
TTTTGCTCCATTTATAAAATATTCCCAGCTAAAAAGTGTGAAGTGTTTAAAGACAAGGTGAAGTTTCTGGGGAAGATTGTA
CCCAAAGATGGCTACTGCATGGATCCTGCTGAGTTAGCGCCAGTACAAGCGCTCAAAGACCGCAAACCTGAGACTGTAGGA
GACCTGAGGAAAATGTTAGGATTCCTATCATATTACCTTCCATACATCCCAAACTTCTCACGCATTGCCAAACCACTGTAC
AATCTGCTATCCACTGAGAAGACTCCAGGTAGTAAAGGCAAAGAAGCAAGCACAAGTAAGAGTAGGGGGAAACGTAAGAAG
TCGGACCAGCTACCCTCGAGCCAGCCGATCACCTGGACAGAACAACATCAGGAAGTGCTCTGCCACCTCATAGAGTACCTG
CTGCGTCCACCAGTCCCCAGTTACCCCGACTTTGAGAAACCGTTTGTGCTACATTGTGATGCCTCTCAGGAAGGGCTTGGT
GCAGTACTGTACCAGAGACAACAAGACAAGCTGGTTGTTGTTGGTTATGGTTCAAGAACACTAACTGCTCCAGAAGCCAAT
TACCACCTCCATTCAGGAAAGTTAGAGTTTTTAGCAATGAAATGGGCCATCTGTGAAAGATTCAGGGAGTATCTGTATTAT
GCACCCTCATTCATTGTCTACACTGACAACAATCCTTTAACCTATTTGACCACAGCGAAGTTGAACGCCACAACGCACCGG
TGGATTGCTGAGTTAGCAGACTTTAAGTTGAGATGGAGATGGAGCCAGAAGTAATAACAACTCTCACACAGGCACTCCAAC
TTGAATCTCATGAACATGAGCCCTGGATGCCCTGGATGTGTCCAGTCACCATCACTGCAGCATGTACTGATGTAGAGCAAG
AACAAATAACATCCCCAGTGGCAGAATTCACCATAAAGGACCTAAAGAAAGCACAAGAGGATGACCCAGTCATTGGCAAGG
TGCGAGAGTATGTCATCACAGGACAGTGGCCTCATCTGAAAGGAAGAGCCCAGCGTGATGACACCTCTGTTCTAGTAAGGG
AGAGAAACAAGTTGTATGTCAACGAAGAGGGTATCCTGTATAGGAAGTCAGTGGCTCGAGTTTAACTGGTGCTACCTACAA
CATTCCACCCGCTGATCTACAGAGAGCTACATGAGGAAATGGGACACCTCGGAGTGGAACGGACACTCAGCTTGATCCGTG
ATCGATTGTTCTGGCCTCATATGCAAAGAGATGTGGATCATTATGTAACCAAGGTATGTAGCTGCCTGAAACGGAAACGCC
CTAACAAGCCAACTAGAGCACCGCTGGTTAATGTAGTGACTACTTACCCTTTTGAGATGGTCTCAATAGACTACCTGCGCC
TGGAGAGCTGTAAAGGAGGATATGAGTACATTCTGGTTGTTATTGATCATTTTACTCGTTTTGCACAGGCATATGCATGCA
CCAACAAGTCAGCAAAAACTGCAGCCGAAAAGATCTTTGGAGACTTTGTGCTCAAGTTTGGATTTCCTGCCAAGCTGCATC
ATGATCAAGGCAAGGAGTTCGAGAATAAGCTGTTTTCCAAGCTGGTAGAATATTGTGGTATTCAAGGCTCACGCACAACAC
CTTACCACGCAGCTGAAAACGGTCAAGCGGAGAGATTTAATCGAACACTCCTTTCCATGCTTAGAAACCTGACAGAGGAAG
CCAAGCAACTGGAAGAGCTCCTTAGCAAAAGTTGTGCATGCGTATAACGGTACGCGCAGTGAAGCAACAGGGTACGCCCCA

TACTAGCTCCTCTATGGTAGAAACCCCCGGCTACCTGTGGACCCAGTGACCAGAGCAGTTCTCACAATGAATATGCCAGTA
AGTGGAGAAAGCAGATGCAAGAAGCATACAGGCTGGCATCAAAGACAGCTCAGAGGGAGCAAAACAGAGGAAAGAAGCAGT
ACGATCGCAAGACTCATGGTGTGGAACTACAGCCAGGGGGCAGAGTATTGGTGTGGAACTTCAGGGACAGAGGAGGACCCG
GGAAACTCAGATCTTATTGGGAGGAGCAAGTCCACGTGGTAACTGAAAGGAAGCACCAGGACAGTCCTGTCTACACAGTAT
GACCTGGGAGGGGCCCAGGAAGGACTAGAGTCCTGCACAGGAACCTGTTACTGCCCAGCAATTTTCTGCCTGTGGAAGAAG
AGGATAAGCGGGAGGACAAGAAAAAGGAAACGAGAAAGCCAAACACAGTGAAGAGGAGCAAAAGTCAGGCAAAGCAGAAGG
GGAAAGACCCTGACAGTAGTTCAGAGGATGAAAGTGACTGGAGATTCATCACCACGTGACCAGTGGAGCCATTGAACCTGG
TCAGAAGCTAGCTGAGAGTGGAAGCGGGGGAATTCCAGCCGCAAAGAGCTGACGGAGTACTGGAGCAAGGTGTTGAGGAAG
ATGAGTGTCCAAAACAGGATGAGGAAGGCAGGATGGAACCAGAGGTGGAGAATGGAGAAACGGCAGAGGCAGAGCAAGATG
CGTCATCTGATGAAGCAGATGAAGCAGACAAGATCAACTCTGAGCGGGTGTCGTCATATGATGAAGCAGACGAGATCAACT
CTGAGCGGGTGTTGTCATCTGATGAAGCAGATGAAAGGGGACCCGAGCCACAGTCATTCTCCCCAAGAAAATATCCTTTTC
GACTAAGACATCCACCTAGGACACTTACATACAACACATTAGGCCAACCATCAGTCACACACAGAGACAAGTGATGTTTAC
AGTTAAAAGTAAAAGACACACATTCATAGTCACATGTTAAGTTTATTGTCTTGACAAAGTTAAGAATTCATGCAGTATAGT
ACATTTAGTCCTGTTAGTTAAAAAGTAGTACCAGTAGAAGGGTACCAGAACTCAGACCGCTGACACATAGAAGAGTACTGT
GACATATGTGAGGCATACAGTAATATATGTGGGTCTGTGTCATGGGTTATGGGGTAGAAAAAAGGGCGAAACCTTTCAGTC
TGAGATACAAAGCCCACATAAATAGTGGAGTAGCAATTCAGATTTCACCTGGCCAGTGTCATCTGGTGCTGACACATGACA
TAAAGAGGGAGATACATGTCATGTCAAAGACTGTGGCAGTACAGAGAGTATCCCGAAAGACTGGCTCCCATATAACCTACC
ACTATAACTCACCAGTTGAAGACTGGAGTACTTTGGGAAACTGATAATGTCGGGACGACATTCATTTGGGAGGGGAGAGTG
TGGGACCCACAGTAATGTTGGGCATATTAATGGACAATAGACAGTGTACACCCCCCATTCTGAGTTACTATTATTTTGTTT
TTATTTTGTTCATTCATAAACTGCGATATGTCCATGTACAACAGCAACTATAATATTTTAGTAGGTCAATGTGTGGTTATA
ATGAGTGACGCTCACGCAGGCGCTTTGGGCACCAGGGGTCCTTAGTGTGTGGCTCAGGAGGGCGTGTCTGTTGTCTAGGCA
ACGTTAACAGCCAGCAGTATGTAGCCACGGTGTGCAAATGTACCTGTGTTTCTGTATTTCCAGGTGAGCAAAAGTGATGAA
ATGATCTATAAACGTAGCGACACACATGTAAAATGAGTGTGTAAGAGTAACGGACCATCAGTAATGCAAATACGAGGGTTA
TATTGGAGTTTTGCAAGTTAAACGAGTGTTAAACAGTGCTTCACCGAGTACTTAGCTCATGAGGAAAGCAGGAGTGTAACC
ACCTTTACCCTGCTAAAATATTCGCTGTGTGAGTGTGCAGAATATTATGAGCGCGTTTGTATTTTGATCATTGTATTAAAA
GCGGACAGTTACGTTTATGTTAGAGGAAATGGTTATAGTGTCTGTTGTCTAGGCAACATTAACACCCAGCAGTATGTAGCC
ACGGTGTGCTAATGTACCTGTGTTTCTGTATTTCCAGGAAGAAAGTAAAGGAATTTATTTTACACTCATGCACGCCTGGAA
GTTCCTTTGTGTGTCCAAGTGTGCAACACTTGTATGAACAGACTTTGCCTGGTCGCACCTTTAAATCCGTCCACCCCTCAG
CAGTAATATATTCAAGAGAGAGAATGAGCGAGGCAGGCAGAGAGAGAGACGGGAAGTTTTGAAGCCTTTCTGTTGGGAATG
TTTCTGCTTAAAGCTGAGTAGCAAAGCAGGCGATAATGCAGAGATTGAGGCAGGGAAGAGAGAGTATGGGTGATGGGATAA
GGATTGCGGATTAAATTAAATGTAGAGTCAGGGAAATGAAGGTCAGACGAAAGGGGAGCGCAGTAACAGGAAGCGATAAAT
GGAAGGAGAAGTTCCCCGCAGCTGTCCTGGCTTTTGTTCCTGAGGGCCGGGATGTGGCCCGGGACGTGACATTCAAAGGGG
ATTGATGGATATTGTTCACAACGAATGCTGCCGCGCAAGGCCAAAGCTACGGCTCATCTCGCCTAATCTCTGCAACTGAGA
TGAGCTGTCATTCACTTCGGGGGCTGATAATGCAATTCCAGCTGACCATTGACAGTTCGTTGCTCCACATTAGCTCGAGCT
GTAGCGGGATAACTGTACTTTGCATTTAGGGCAGCGGGCCTTTAACGCAGATTTTCCTTTCCCCACTTGTTCTCTTCTGAG
CCAAGAAGTTTTCACTCACTTTGAATAATCCTTTTTGAAAATATTAGATGTGGAACAGGAACGGGGAACAGGTAAAGAATT
TAAAGTGGTGCTCCATTTTTGGAAATTTCTTTTGCGGATTTGAACTTTTAAGGACGCAACTAAGGTCCTATGATTAAAAAC
TGGGAAGGGATGCAGCTGTCATGTCACACTGCCCCTTTTTAATGGCCACAGTCTCAGATGTGAGTCATGCTGATTTTGAAC
TGCCCGTGGGAAGAATAAACATTAAGCTCTGTTGTCACTGCCTGTTTTCTCCGACAGTTGTCTCTCAACTCGTCTGTGTGG
ATCCCTTTGACTCGAGTCACACTGCCTGACGCAATGCACAGATTTGATTGGCTGATGGCATCACGTCAGTGATTAACAATG
CATGCAATTGGTCTGTGAGTTCCCTTTGCTGCCTGTGCCGTAAAGAATAGAAACACTGCGCCACTCTGTAAAAATGTAATA
ATTCTAAAATGTATTTATTTGATGCTGTTACACTGTTGTGTAATTTATGTTTTGAATGAAGAGTCTATGTATCAGTTCTTT
TGTTATCTAAGAATTATTTTTTGAAGCCACAGAAGTATTTTAATTTTAGTTTAACCTTGATATTATAAACCAGCTGAAAGT
TACCTTAAATTAAGAACCAATGAAAGAAGAATTTGTATCACTTGTGTTATCGTGTTATTTTCTCATCTGATATGATTTAGT
CTATGTTTTTATCTTGTGTGTTTTTATTGTATTGTATACACTTTTGTCATGTACAGTTTGGTGTTTACTTAACTTTGTATT
TGTATTTTTAGTTTGTTATATAAATAAACTTGACTTGACTAACACTAGCATGAGTGCACTGATGTTGCACAACCTGACACG
GAACAAAATCACATTTGTCGACCGTGTAAACGTTTAACCTTAAGATTGGTACTATTCACTGCTTAAGGACCGATGTAAGTC
TATATATACTTTGACTTTGTTCCCTAAAGACGGAGGCTTTAAGTGGCTTTGTCAAATTAGGTCACATCATCACCCTGTTTA
ATTTTCCATTTCTAAAAAAAAAGAGCGGACCAAGCTCTAAAATAAGAAAAGTCTCAATCCATTTTTGCTCGCTCTCTATCC
CACCTGACCACGGGTTCGTTAATGATCTCATTTAAGAGGTCGTGGGTTTTCTAATTGGAAGTGGGCTGACTTCTTTTGCAC
CGGGCTGACCTCTGCGACAGTCTAAAGTTAGTCTCGACTTTTTTAACCTCGCTAAAAATACTCGCACTTGTGTCACCGCTG
TCCTCAGAAACACACCCGACCCTTCAAATAGCTCAAACAACCTTTGGACCTGAGTTCTCCCTTAGGAAAAAGTACTTTGC
TCGAAGAACAGGTAAGGTAAGGTTTTATGTGTGTTTTCTTGGCATTACTTCAACATATGATGCATGAGAAAGATGGACACA
CAGGAGGTGAGAAAAGCTGCAGTGAGGAAGCTTTTACCAAGAGGAAGCACACATGAGCTACACAGTTAAGCCCGAGGCACT
CAGGGTAGTGCAGGTATTGCAGTTGATCCGCCATAAGGCAGCATAACACCAGAGACCTTTCCATTCATTGTGATTATGTGC

AACACCTTCACATGACCTCACAAAGTCCAGCTGAGCACCGTCTGGCAGAACACTGTGCACCGCCACAATCCCATCACTTAT
TCAAATATTAGCTAAACCTCAACATCTGATGTTTCAATAGATGCTGTTCACTTCACGCTCATAATTCCTTATTAGCGGGTG
GTGGCTCGCTTCCAGCATGGTGCCACCCACACAAACAATGACCCGTGCACGTACATGCACAAACCTTACACAACTGTCTGC
AGGCGCACACACATTATAATAAGCCCCAAACCAGCAGCGTCGGACACGTTTTTCACTGATCATCCTGACCATCGAGCTCTC
CTCCAGATGCCAGCCACCTCACACAACATCTGTCGTTGACCTTGGGGATTAGGACTCACTAGTGAAGACACCAAAGAGTAC
ATGTGCCTATTGCAGCCTTCTGCCGGGGTCCATTAAGATATTATTTTTTAATGTGTGTGTGTGTTGTGTAGATGTGATTGT
GTGGTTGGTGGAAGAAATTCATGTCATCTGTGACCTGCCCTGCATCGGGTACACAGGTCGTCCGTCTGTGCACCGGTGTGT
AAGTATCAGCTTGCATCTGCTGGACAAGGTTGGGTGACCCGTGGTACTACAGCGTGTCATGAATTTTGTGTTCTCGGCCGA
GCCTGTTCAGCAAATGCAGTACAAACTTTACTAGTCGAACTGTACAAGATGAACACTCTTTCTGAGTCCCCACTCTCCTCC
CCTTCTTTTCTTAAAACTCAGCAAAAAGTTAGACATCTTCTTCTTCTTCATTCTCTTTTATCAGGATCTGCGTTTTTAATT
AATGCCTCTTAACAAGGGCCCTGAACACTGGGTTAATTGTGCTTTTCATGACAAAATCAGCGCAAATGCAAATTGTCTCTG
AGCGACCTCTGCGCCGTGGCTGGGAACGGTGTTTTAATTACCCAGACAACCTCTTAAGTGGTGGAGGAACTGTAGTGACGA
GCTGACCATATGCTCAGCAGTCGCAACTTATCTTGCAGTTATATATCAGCTGGCAGGTGGAGGAGGACGTGAGGCAGGAGA
GGAGGGGGGAGATTAATGCTGACATCAGATTTATACAAAAGGAGGAAAAGTAGGGAATGGAGAGACGGGAGACAAGAGGAA
AAAGATGATGATAATGTTAAAAGAATTCAAGGTCAAGAAGATGTAAAGAAATGCACCATAGAAGAGGTATTTCCATTTCCC
AGGGTGCACCGTGCCTCCCTCTGACTACTCACAACTAACTGACACTTTCCTGACACTCTCTCACCAACAATAACAAGCCCA
CACAAACAACCACCCCTCTATCCTTCCCAATAAAATGGCATTTCCCTCACCAGTCCACCTACTGACAGGCTTCCATCGTAC
TCACTCACACTCACATTCTCCACACAACAGTGAGCAGCAGAGGACAGAAAATACGCAACCAGAGGGCAATTGGCAATGCAG
GACAGAGGTTGGGTGACCCAACAATACACAGAAATACACACATGGATGTCCACACACAACCACCAGCAGTGATTTCATTCA
TTGTGTGTGGTCTGAATACAGTTTTGCACAATGATGTAAAGGGTTTTAACAGATAATGAAATAATTTCTACATGTTTGATA
ATACATGTGCAGCTGCTGGTTGCGTGTGTGTCTGTTCTTTTGTGCATTTTTAGCAACTTTAGCAGTGTTGCTCTAAGAATG
GTCATTCTGTCTGTCCCACCGCTTTGTTCCTGAAATATTTCAACAACAATCAGATGAAACGCCATGCACTTTGTTATGGCT
GTCATTTCTTTGTTTTGCTTGGGTTATGTAGTTTAATGTCTCAACAACTACCATATAAGCTAATGGATTTCAGTAATACTT
GACAAGTCTGGATATTTCATGAGAAGTAGGTGGATAAAGCTAGCATGACTCAGCGATCCATCTTATTTTATATTGAGGGTA
CTATACAACATTCAGCAACGTATAGTGAAGAAATTAATAATATTGGTAACAGCTAATTAACTATCATGCCTATTCTAGCCT
GTAAATGTACGTGCCCATTAAGGTATTTGCAGAAAAAGCAGCTCCGAGTCAACTTGGAGCCCTTTAGCAGCTTGTAGTATG
TAACTCATGAGCCCTAACAAGCACAGGCAGCCAGCACGGCTACAAGAACAGAAGACCTTGGATTACAACACCCTTAACGGA
AGTGAGACTATTCTCTGGTCAGGATGCCATTAATTCTGTAACACTGTAATATTATTTTATTATTATGTTCAAAAATCTCAT
AAAGCTGAAATAGGCGATTCTGAAAAAGATTGGGTTGGTTTGCTGACCTGGGAATGAGACTCAAAAATCAGCTATCTTTCT
CCAGAATCACCTACTTCACCTTTAATGCCATTTGAAAGAGCCGGAAATTGAATCAGTTGGGATTTAAATTTTAAAAATCAC
CCCACATTACTGCTAATTATATTTTAAATGATTTCACGGTTTTAGAGTGTTTTTTAATTTGTAGTCACATCAGAGAAGATA
TCTGCATGCCCCATTAATGATAATCATTAATTGTAAGGACTCAGTCACACATTAAAACAGTCTCATTGAAGAACGCCTTCT
CTGGTTCAATCACCTCGTTATTGACGAAATCAGGAGCCGTTAAATGGTGGTTTACACAATAGAACAGTTTATCACCACCTA
TATGTAATGTTGAGGGAGAAGGCCCTGCTGTATTTAGTACTTTAGTAGAAAAAAACAAAAGAATCCAGAGGGAGTCTTATT
TCTTCTTTCACTCCCAGTCCCCAGTTTGTTATTTTTCCTTTGAACTGACAGGTCACAGCAACCTCAAACCAGTATTTAGAG
ACACTTTAATAGGACCAACTGGACCACATATTACCAACACACACACTCGCAGGCTCAGTGAACCCACCAGTCTGACTGTTA
TGGTATTTGAACACCTTTCCTAGTCCACCTGCCCCAATTCCACTGACACCAGTCTGAAATTTCTCCTACACTGGTTCTCCA
TGCTTCCCTTAACACCATCAGCACTGCACAGCTCCACACACGCACATCACTTTTTTTATTTCTCACATCCACACTTCCACT
TGTCCTTCCACCTGTCTGTCTGTCCCTCTTCTTGTCTTACTTTCCCAGTGCATAACCATTTTTCTATCTTACTCCCCCACA
CAAATGTCCCATAGGTCTTCTCCCCCAGGAGCGTTATATCACACCTGGGTATTTCACACTTCACAGAGCTCCATGCTCATT
AAATTACTGAATTCCCGAGGAATAAGGAGGCGATATACCATGCATCACGCTTGACTTTTATATCTAATGGAGAAGTGGTAA
TAAAGTTTATGGGAACATATAATGTATAGGTCTAAGAGTGGAGAGCGCAGGGGGCTGGTGTGAAGGTCCAGAAGGGTGTGC
AGATGGTTAAAGTACGGCATCCTGCTTGTTGGCAGGAAGATGAGCTTTTTTTGAACAGTAATGGATATTTATGTACGTACG
TAATTCTTGTCCCACCCTTATTGTTTGTTCCATTTAGTTATCTACAAAACATGCTTGCGGTGTGCTTCAAGGGTCCATTCT
GGGTCCTATTCTATTCCCTTTTCACGTGCTCACCCTTTGTCAAAATATTCTTTTGTGTATATGCAGACGATACACAAGTAT
ATCTTTCTGTCAATCCAATTGATACTTGCAGTTTGGCTGCCATTATGGACTGCTTTTCAGGATGTTTCCAGGTTCTCCAGT
TTATTTAGGGCAAGATTCAGATAATTATATTTTCAGCTAGCCTGAAAAATGTATTTTCACGTGCTTACCCTTTGTCAATAT
ATTTTTTTGTGTTTATGCAGATGATACACAATTAGTCTTGATTCAAACCAGAACTTTGACAACCAAGTGCCAGGAAGTATT
CAGTCTTGATTTTATCAGTTGAGAAATATCTCTAAAGTAAAACCATTTCTGTCTGCTGTTGACCTGCAGAAAGTTATTCGT
CCCGTCTGGATTTATTGAAGATCACTCAGTAAAAAGACACTAAATCGACTTTTGTTTGACCCAATGCAGATGCTCAAATTT
TAGCTGGAACTACAAGAATAAAACATGTGACCCCAATTTTAGAAACCCCTCACTTAAAGAATGCAGTTCAAAATTCTATTA
TTCACTTGTGAGGCATTAAATGGATTGGCTTCGAGCTACATCTCTCGTACAAGCATGCACACTAAGGTCAGGACAAAAAGC
TCTACTTGCTGTTCCTATAGTCTAAATTTGAAACCAAGGGTGACATGCATTTTCTGCAGTGGTTGAAGGAATTACCGTTCA
CAAGCTGCCTTTGTAATATATTCCAACTTGTAAATACATGGCCTTGTATTTCATTTATTTTATTGATTATTTAATTTCATT
TATCAGGTTAACTTTTTAGCCATGCTAGCGGCACTGTCAGTTAGTCAGCCCACCATTTTGGTCAAGACTAGAATATCTCAA

CAACTAGTAGATGGGTTACACCACAAAACATGATCAAGTGCCCGCTATGTTACTCTGCCAGGTCATGATAAATGTATCATA
CAGCTGGTGACCTTTTTATTTGCCCCCACCCCTCCAACATCCTGAGTCCACCACTGCAGACAGTTATGGTCTACAGATTGC
TTCTGATTTTGGGTGTCCGTCACTTTTCCTCTAGTGCCACTGTGACATCGACATTTGCTGTTCAGAAAAAAAATATTCTGAT
CACTTTTGGATCGACTGTCTTGAACTTTGCTACAGATATTTAAGGTACCTGGAGTACCAAGAGGTCAAAGCTTTCCCTTAT
TTGGGAAATATCTCGTCATCTACTTCCGTCTACACTTTCAGCCTTATTTTCTTCCCCTCCTTTTTGTCTTTTTCTCTCTAC
ATTGTGACCTGTAAAGGTAGCTGAAGTTACCCTTCTCTCTTCCTCCTCCTCCTCCCTTCTTCATCACCTCCCCTGTCC
TCCAAGCTTAACGCACCAACAGCTCCCCTCCCCCCGCAGTTCCCCGCACAAATGTCACTTTCTAATTGGCCGGCGTTGACG
AGTCAGGAGAGCATTGCTGTTTGGACAGTTGTTAATCTCGGCGGGTTGGCAGTGGCGGTGAGATTGTGGTGGTGGCACGGC
GAGGGATGACAATCTGTAACAGCGCTGAGAATGACGCAATGAGGGAGAAAGAGTTCGGGGGAAATAAAGAAGTTTGTGTCT
GGTGCAAAATCAGCAGTCTGTCCAAACATATGTCTGTCACTGACATTATGTTGAGCCTATGATAAAATGAATCCCCAATTT
AAAATGCAGTGTGCATCTCTCACCGTCTCTACATTCCTGTACAATGTGGGCTGTTTAACACAGGTAATGTTAATGTACAAG
CGAACACCCTGCAGCTTCATTCAAGCATCTGCTCCGAAAGCCTCAGCGTCACCCGCACAGGAATGTAATGGGTTTTCTCCC
CCCCTCTTCCACCTCCCTCCCTCTTGTTCCATCTTTCCCTCTTTTTCTTTCACACACATACACACACACAATGAAGTACAC
ACAGACACACAGCGCTCAGGGAACCAATCACCATCGCCTGTTTTCAGGTCCTGTTTGTGAATGGGATTTCTACGGGAGAGG
AGGAGGAGGAGGAGGCAGGCACCGCTGCATTTGTAGGCATGAAGGGAAACATCACCATCCCCATCATCATCCCATTCTCAT
TTCTTTCCCCCTGCTGAAATATTTCTTTCACTTTCTGCCGGCTGCTCCCTCCAGTTAATTTTTCATGGTTTGGTTTGTTTC
TTCTCACGCCCTCCTTTCCATTTTTGCCGCTCTTTCCATCTCCGTGAACTCTGTCTGACTCTTTTTTGTCTTTTTTGATGC
AGCATTCACAATCGACTGACTGCATCAGAATTGCCCCTCTTTTCCCATGCTTTTCTTTCACTTTTCCCCCTGACATGCCAT
TATCACCCTCATCCAGTGCTTTTTGCCTCTTTTGCATCTTTTCACTCTCTTGTCATGCCACTGCAGAACAGCTGTCTTCCA
CCACTACATCTTGTCCCTTGTTCCCATCTTTTTCACCAGTCATCCCGTCTGCCTCCCAAAGCAGCAGCTGATGCAGGTTAA
TGACCACCACCAACACACACACACACACTGTAGACACTTACCTCCAGTGACAGAGTCTTCGTCCCGAGTGCTTCTGAATCCTC
AGTGGATGAAGGAAGAGCGGCGAGCCTGCAAGCTTCTGGCGGCCGAAGAGAAGTCGAGGACAGAAAGAGACAGAAAGAGGG
AGAAAGACAGATAAAAGACAAGAGAGAGAGAGAGAGAGAGAAAGTGGATGAGAGCAGGCAGGCTGCGCGAAGAGGAGGAGGGT
GCTCCGGCTGGCTGGAGGTGAGATGAAGGAGAGAAAGAGAGAGGGAGGGAGAGATAAAGGATGAGGGAGGGAGAGAGCGAG
GGTGCGCTGGATTGGGATAGGAGGGGATTGTGTGATGTTTGAGAGAAGGAGGAGGGATTGGAGGGAGTAGAGAAAGAGGAA
AGTTGGAGAGAGGAGGAAAGGGAGGAAGGGTGAGGATGCAGAGGCAGAGAAGAAAGAGAGGAAGAAGGTAAAGAGAGAGGG
GTGAAGAAGCAAAAAGGGACAGAGAGCATAGAGGCAGATATGTTCATGTTGTAAGAGAAACCAAGCCTGTCTTTGCTCCAC
TACAACTGTAACCTTTTGCAGCCACCCAAAAAATACAAGTACAACTCATGTCAGGCTGTCACACGCTGCCAGTCCCTGGAG
ACAATCAATTTCAGAGTGGAGAAAATGTCCTTCCATCCATTAAATCACACTGTAATCCAATCCTGTGTTGGTGTGTTGCCG
GACGGATACTGCAATTAACTTTGGAGCAATAACTTACCGGAGCCGAGGGGACGAGCAGCTGGGAGAGAAAGAGGGAGGGAG
GGTGGTGAGAGGAGACGAGGAAATAAGACTTCTGTGAGAGCCGGTGACCAAAATGGAATCGGGGTAACGTGGCATAATAAG
GAAAAAGGGATCGTGTGATAATGACAGAGGGCATTAGACGTGACATGTGAAGAGTAGAGGAAGAGGAGCAGCATATTGGCC
TCTGGCGAAATGCATTCAAGGATGAAAGGGTGGTGAAGAAGAGGAATGGAAAGAAAGAAAGGAAAGAGAGAAGAAGGCTGA
ATGGAGGATGATGATATTATGAAAATCCACTGATCTGTCATATGCCTTTCAGTAAGTTTAGGGCAGGTAATGGGTCACATG
GTAACATACAGTACAGCAGCACTGAAAGCACGCAACCTCAAAACACTTAACAGTCATGGGCGGATTATAAACCAGCAGGGC
CCCGGGCACAGGCAAGCAAAAGGCCCCCCCGCCTCTTACCCAGGAGCAAGGCACCCAGACCGAGAGAGATGCAAAACAGTG
GCTGCATTTTCTATAGTCATTTTGCATCTCATGATTGGATTTTGTGCCCTTTTTTGGCCATTTAGCATCCCTTTGTTGATT
CTTTGTAGTCATTTATTGTCTAGGTTGGTGCTTCAGCACCTCTATGTAGTTTTACGTTTCTTGTAGTAATGTTGCATTTCT
TTGAGGTTGTGTGTCGCTGTGTCGTTTTGCATCTTCATACAGTCATCATCTTGTTTCTCTTCTTTTGTCTCTTTGTGGTCA
TTTTGCATCTTTTGGTAGTAGTTTTACGTTTCTTTGTAGACATTTTTGTAGTCATTTTGCATCTCTTTGCTTCGTTTTGTC
TCGCTTTGTAGCTGCCTAACGACTCTTTATGCGTGTCTGTGATTGTTTTGTATTTGTGGTCATTTTGAATCTCTTTCTGGC
CATTTTGTGTTTTCTGTTGCCATTTACGACTCTATGTTTTCTTATGTGTCTCTTCGTAGTCATTCTGTGGTTGTTTTGAGT
TTCCTTACAGTCATTTTGTTTTAGTTATCTTGTTTTTCTTTTCTTTGTGCTTGTTTTGTCTCTCTTTGTGTCGTTCTTCTG
TCTCTCCTGTGGGCAGCTTTGGCTCAGGAGGTCGAGCAAGTCATCCAGTAATCATAAGGTCGGTGGTTCGATCCGTTGCAC
CTCCAGTCTGCATGTTGAAGTGTCTGAGTTTCTGAACCCCAAATTGCTCCTGATGGCTGTGTCATCGGTTTGTGTGTGTGA
ATGGCTGAGTATATAAATATCATGAGGCCCTTCGGATAGAAAGCGCTGTATCGCTCCTGATTAGCTGAAGGGCACCTTGCA
TGCCAGCCATGTTCAAATGTGCATGTGAACAGGGCATGGACTTGGCCGTGTATTAAAGTGCTTTACAACGCTTGTTGACCC
GTATTCATTCACACATTCCTACACTGATTGCAGGGGCTGCCATACGAGGTGCCGACTTGCTCATCAGGAATGAAGCAGCGC
TTTACATCCAAAGCACCCGACAACATTAATATTGTTAATCCACTCACTTAAACACAGATGACCACGAGCAATTTGGGGCTC
AATATCTTCCCTCGCTTTGCTTTGCGTCTCATTGTGGTCATTTTTGTTTGTGACTTTGTGTCTCATTCTGGTCGTTGAGCA
TCTCTGTGTAGTTGTTTTGTGTTGCTTTGTAGTAACATTGCTTTTATTATTGCATCTCTTTGTTGTCATTTGTATATTTGA
AGTTTTTTTGTGTGTCTTTGTAGTATTGTTACATTTCTTTGAAGTTTCAGTGTGCATCATTGTGGTAGTCATCTCTCCTTG
TGTCTCTCTATGAGAAATTTAGCATCTCGTTGTAGTTTTTGTTTCGTTCTTTGTTGTTCTTTTTTGAAGTTCGGTGTTTCC
CTTGGGGTTTTTTCTTTCATCTTTGTGGACGTCATTTGCGGTTGTTTTGTTTTTCGTTGTCCTTCTCTTTGTAGTCATTTT
GCACCTGCTTTTGTGTGGTGTTGTAGTCATTTTCATCTCTTTATGACTTATATTTGTTTCTCGTCTCATTGCAGCTGCATG

TTTGTGGTCATTTTTAATTTGAAAGAAGTCATTACAAACAGAAGCTCAGTCTGCCTATGCCCAGTGGGACCATTCAGTAAT
TAATCCATGATTACAATGTGGTAAAAGGAGCCCTAATTAATTCCAATTAGCTTGTGTTTTTTGGGGGGGGTTTCTTGCCGT
GTGGCCATGTCTGAACGTTTCACCTTTACAGGCGAATGAATCCCTGCACTGAAAAAGTGCAGCATGGTAAAATACCTTCCG
GACATGCTTTAAAAAAAAAAGGGCGTAACAATAATAGAGATGTGATGCATTTGAAAATCTCCTTTTGTATTCAGTGTCAGA
GGTGTGGGTCAGCTACCTTACTCTGGAGCTGGCAAGGAGAGATTCATTCATTTTCTCAAGGACACTTCAGCAGAGTGGATG
CTCGCCAACAAAGCTTAATCCTCCCTGTCCCCCTTTAAACAAAAAAGCTTTACTCAGCAGTTTAAATGCATAACTGACAGT
GGCAGTCCATTTTCATAAAAGCCTGCTAAAATCAACTATACAGATCTTCAAATACAATCTAAAATACCCGGAGGCTTGCCT
GTAATGTGTTGTTTTCCTGCACTCTAATGTGCTACACCGTGGGATTCTCCCCTCTGAGACTCTCAGGCAATTCACCATCCA
CAATGTGTGTTGTACATCTGAGTGTGCATGGGTTTGCACTGCAAGAAAACCCCACCAACATATTTTGTGATTTTAAAACAG
ATGGACAGCAAAGAGAGAGACAGAGAGAGAAATATGGGCGCTGTTCCAGCTTTGAGGGATTTCGTTTTTACAAGCTGCTAC
GTTTCTGTCGACTCCAGAGCTGCAGCTTCAAACCTCCCAGATCAGACTCCAGCTGCCAGTCCTGCACAAGCACCACTGGCA
GTGAAAGAGGAAGTGAGCGCGCTGCACACAGAATTGATTTATTCAGATAAAAATCACAGTTTGATTTGAGAACTTGGAGGA
GGAGAAACAGAGAAACATAAGGGAAGAGATTGTACTATCATTGCCCCCGGGGCTGTTACATGCATTGTTAAGTGGATGCTT
CTTTAGTGCTCATGTCATTCGAATGGCTGCGTGCACCACCGCTGACACCAGCAAAAAAATTCAGTGTGATTTCTGGATTTC
TGGTCTTGCCTTTGATTTCATTGTCCTCAAGTGATTTGGGGAAACAGGCAGTGACGGAATGCAACTGTGCTGTGACGGTAA
AAACCGGGAGAGTGTGTGTGTGTGTGTGTGTGTGTGTGTGTGTGTGTGTGTGTGTGTGTGTGTGTGCGTGCGTGCGTGTGT
GTGCCCTGTGATAGAACGAGACGGCACAAAATTAATGAGACAGGAGAGAGAGAGAGCACGCCATGTCTCCTCCAGCTGTTT
ATCAGCCCTGAGCACTCTGCTCAGATTAAACTGCAACTACTTTATAATTATTATAATGTATTCTTATTGGACAAGTATGCC
TTTCTTAATAAAGGGTCCGATAATTGACTCTCTGCCTTATTTCTCCTCCTCCAGCCCCTTTTCTCTCCTACACACACTCAC
TATTGATCTCATTAATTTACGTTACATTCTCATTAGACTCAAAGAGTATTTAATTTTACACCAGCAGCATGTTGGCTGTGC
AGTCTTGAAGGCAAGAGCCATTAGTCAACAACAATAGATTGGTCCAGTCATTCAGATCATGCGCCATTAGCACAGATTTGG
CAGTTCAGATGTTCTTTTTCTTTTTTTTAGTAATTTAAATCGTATTACTCTTATTGAAAATTGTATTTTTTCTTTTAGCCT
CTTCAGATAACCTGATGGAGGTCTAAGTACCGAGACGTTGTTGTCAACTTAATTGCAAGTGAAGTGGACAGTGTGCGGGAG
TCTTGTTCCCTCTTTTAGCCTCTAAAAATCTCCCAAAATCTCTCCAAGATAATTATTTCACTTGCAATACAATACAATCAA
AAGCGGAATTCCACCATAAAACAGCACTTTGCAGATTATGCTTCATCCTCGACTGTGTAAATCTTTGTTTACTGTTGCAAA
TTGCCCAAAAGATGAATATGTCTCTGTAAAAAACAACCCCAGGCCTTTAATTGAGAACATAAATTCAAGCTCCCATACACT
GTTGGAGCTACAGGAGACAGGATTTATAATGGCACATTTCTGATCAGTGGAAATCACAAAATGGGACAGCATTTGAAATTG
AAATTTAACCGCAACGCACAAACTGTCACCTCAGCAGACAAAAGAATAACTGTACCATACAAAAATAATCAGACTCTTCTT
TTGAATGTTTGCCAGCGGGGCTGAACAGGATGCATGGTGGTGGAGCTGCTGGTGGTGCAAAGGAAATAAAAGCTTCCTGTT
GGTTCCCACTCAGGCATGTAAAGCATGTGAACATATTCTTAAACTTATTCTGTAGTACTGCTGCTAGCACTGTTATATTAA
TGCTTTTATTGTTCCTTAAACTGTTCATGTAAGTACTTTGAATGATGCCTCTCACTATTGTTTTGAGACACACATGCACAC
TTTCATCCACAAAGATTTAAGAAAGTAACATGGTTTATTTGTCTCTGTTTTGATTCTCATCGGCTGCTAAAAAAGTGGCAT
CATGATCCAGTATAGTTTGCTTCATAATAGGTGCATTAAAGGGACAGTATGTAATTTCTGCCACTCGGGGTCCGCTAACCA
AACGAAACAAAAGAAGAAAGTGGCGGAAGATCGTAGGAGTTGTTGTTTTTATTGTTAAACAAACAATCTATTATTAAGATT
TATAGTTTTTAATTTTATTTTTAAAGATGATATAGACAGTGTGTTGACAAGATACGCTGCAAAGAATAAACAATGCATTAA
CAGTTTATAACCTTTATAAGGATAATGCATTGAAAAAAATAAACTTGTCTCTGATGAGAAAAAGGGAGACATGAAAAAATA
GACAATGCTTACAAAACTTAAATAGTTACAAACAACACACTTTCCTCACTCTCTGACACAATCCCTGAGATTTCTCTGGCT
TAATCATTGTTGGGATCAAGTACACAACTCAACAAAATATATATAAAACACGATGTAGTCGTTTTAAAGCAGAAATGTTGC
ATATTATATCTTTAACAATATACTTTATTGTATTTCTGGATTGTGATTTTAATAAGAATGTGTTTTATTTCTTGAAATGTG
TTGTATGTGTCAGGGCTGTGGTGTGTGTTTAATGGATTGTGTCTTCACTGAGTTTAAGTGGCAACACACTGTGGCCGCTGA

SCAFFOLD 4 (110,000 nt); encodes pvalb7

ATTAAAGAGTGCAACGCAGTGTAGATGCTTATTTGACAATAAGTGTTTTCTTCTTTTTAAAATGAGAGATAGCACCATGAA
TTATTCATGCTGTGCTTTTCTTGTTGCTCTTTTTTTTCTTGTTTTTCCTCTCTAATTTTATCAAATATTTTTAGTTAATGT
GAAGCACTTTTGGTGTTTGTTATTTGGCTTTTATCATCTGAAAAATTGGAGAAGCAGGCTACATCAGTCATTACATGATTA
GCGTGGTGCATGACAGTTTACACACCAAAGAGCTCCATCATGGCCTGATAAAAGAAGCTGTAGATTCAGCTGTGTCACTGT
GAGGCAAACCACAGAAACCAACTCAAACACATCTCGGTCAGGGGGCCCAGATACTGAACCGCCCCCAAACGACACTAGAGG
TGTGAGTCCTTCAGTAGTTTATCCACACAAGGCCCATCAAGGGCAGCAGCTCTTTGTATGGGCAAACCCTAATTACAGTTT
GGACCGTCATGAAAAAGCTTCCGCTCTGACATCAAATACAAAGTCATTGAAGTACAAAGGCTTTCATTTTTTCCTTTGTTT
CAGATGCCAGCACAGCAGAAGGAGTGGAGGACCGAGGAGGAAACGGCGGAAAGATAACGGGAATTAGAAAGAGCCGGCAGA
GAGAGCGAATCGGACGGAGACAATAGTGGAGACAAAAATGGCACAGGGAGACAAGGAGACAGACAAGAATGAACAAAAAAT
GGAAGCATGATAAGAATGATGGATGTCGAGAAAACACACACGCGTGCACACACACAGACACACACACGCACACACGCATAC
CATTAAGTTTGGATGTGGACGAAGCGCTAAAAGATGAAGCAGATCAGAACAATGAAACACAGAGATAATTTAACAAACTTT
ATGAGCAAATTCTTTCCACTTCATGACTGTACTGGTCCTACAGTGTGTGTGTGTGTGTGTGTGTGTGTGTGTGTGTGTGTG
TGTGTGAGTGTGTGTGTGTGTGTTTAATTTTGAGGGACTTTTCCTAATAGTTTTCCTTTCTCTGTCAGTTAAGGTTAACAA
TGGCCTATTCTGTGTGGCGGTGAACAGGAGACTGACACACCCACACCCATGTCTGAACACCGTCCAGAGTGTGTGTGTGTG
TGTGTGTGTGAGTGTGTGTGTGTGTGTGTGTGTTGGATATTTTTGGCGCGGTTATTCCGATGACAGTGATGGTATGTTGTG
ATGATAACAGTTTATATTCATGTGTGTGACAAAGAAATTTGGCATGTGTGTATGTGTGTTCCTGTTTGTCAGTAGATTGGA
CAGACAGTTTACAGTTTATACTACAGAGGTGTGTGTGTGTGTGTGTGTTTAGAGAAATGTCCGTGTGTTTATGGGTGCGTT
TCTCTCACATAATATCCGCTTCTACCTTTGAACTCTGCCTCCAAATTTGTCTCTGTTATGTCTCCAAAAATACACATTTGA
TGTCTAAGACTGTCTTTCTCACTGTTGCTGCAAATGTAAATGACTGCATTTTATCCAACGTGGTGTACAATTCGCCTCACA
TTCATCCATTCTCACCAGTACATCAGGGAGATGCTGACCATCAGGACCAATTTGGGGTTTATATTGATATCTTTTACCCAA
GTTCCTATACTAACAACTATACTACTGATAAATACAATTTAATATATAGATTTTACCAAAATATTATAACAATAATGATAT
ATAAGATCGACTCTACATGTCTTTTATAATACAGCACTAAGATATCTTGATTTTCCTTCTAGAATATCTTTATTTAATGTA
ATAAAATATATCAAAAGGCATCAACAGTCAAAGTCCTAATCCAGCACAAATTTCAACCTAGTTTCCCAAAAATATAGGACA
CAACACATTCAATTCACAATGACACCTTAAAGACTGGTGCTAAATCCTTAATGTAGTCAATAAGACAATAGAAAGACGAGG
ACAGCACTCCAATTTGACCGTGTAGTATTTCCAGGCGGACATTTCAGGCGAGTAAACCTTTCTTCAGTACTTATTCTGGCA
ATTTTACGATCAGTATATACAGCACAAGTTCATCCCAAAATGGGGAACTACAAACTTTCTAAGCACAAAATCTCAAAATGG
TGTTTCTCAAACATGAAAAACCAAATAAACCCAACTCAGATTAAACTCACATCACAAGAAAGGAGCAAATGAATCATCCTC
ACTCATCCCACTGGGAATGAGGGTATCAAGGAAGTGAATTCAATACGCCTCCCAGAATTTTTTTTTCCTGTTACCACCCC
TCTGCAGCATGTCGACTGTCTCACATGTAATTTCCCACAGAAATGAAGTCATTTAATTTTGGATTTGGCTGAATATCTGCC
CAATTACATTGAGACACAAAACAAGGGGACTGTTTTTCCTGCACAAAAGAGCCAAAGATGTCACATTCTTGCTGTGATAAA
TGTGAAGAGGAGAGAAAAAAAAATCACTTTCGCCATGTCTCAGCTAATTCCTACAGTTTGTACCATGTCACAATAAAGCGA
TCAACTAAACTTTTAATGAGAAGTCGAATGCATTTTGTAATTCGTCCAGATCAACATGTCTTCAGATAAAAACTCTAGTAG
TTAGTTTATTTCAGGCTGACACTTCTGGCGTGTGTGTGTCATCTTCGGGAAAGCAAATTAGGGACATTAGAGACTCATTTG
ACAAAACAAAAAAGTTCAGAGGGACGATGATAAAAGCCAGGAAGACAGCAATATCCCAGCAGGAAGCAACAACGTTTGTAG
GAAATGTGGTGTTAAACTAGACTAAACCAAACACTCTAACCTAAAACAACAGAGGCCAGTAATGAGCTGAACTTGTTTTTT
GGGGTAAACTGCATCTATATTACATTCAGCAGGTCAAATAGAGGCTAACAAAATCAGCAACAAGAGGAGAGACTTTTTCTT
TCTGAGTACTTTTCCTGGATTTTAGCCTTCAATCATTTTGCCTTATTTTTTGCTGATCTTTGATCTCTTTCGAAAAAATCC
CCACCAGCTCCATCACTCATGCACAAACCCTTCCCACGCACACACACACAAAATAAACAATTTGCCCGGCTTACTGTAATC
CAGGAAAACAGATTTGTTACTGTAAATCTCCTGGCGCAGCCTCTTTTTATTGTTGTCCTGCACATCAAAAGTCTCTGCTGC
TTTTTTAACCCTCCCTCTCTTCTCGACCTCTCCCTCCACCCACCTGTCTCAATCCCTTACCAACTTGTCTCCCCCCCCCTTT
CATATCGCAGAGAGAGAGAGAGAGACTGATTTAAGATTCGGGCGGAGACAAAAACACACCCCCTGACACCAACACACACACTG
TCCTCACACCAACATCTCAGGCCAAGTTCTTGACAACAGACTCCCAGCAGTCTCTGCCTCCTCCACAGCTTCATAATTGAC
CTTCTGTGTGTGTCTGGCCATCTGTGTGACTCCAGTCGCCCTGTTTGAACATTTTCTCTGTGAGTGCTGCTCACTGGTCCG
TCTCCACTTATTTTGTTTGCCCTCTCTTTCTCCTTCTCTGTCTGCCTATCTGTTCAAAAAAACCCTGCAGGCCTAAACGGA
GATGACAGTATCGACCAATTTTTGCCATGAAAACTCTCCTCTCTCCTCCTCTTCCTCCTCTGATGGAAAAAAAAAAAATCAC
TGCAGTGTTTTGCAGTTTTAGCCAGTCATGCTTCTGCAAAATGGTGTCAATCACATGTATTTTTCATTTGTGACATAAAAG
AGGGTTTTTGTATCCCAAACCTGGAAGAAGATCAAAAGTAGAAGAGGCTTAAATTACTACAAACATTTAAACAAGTTATTA
ATCATACAGGAGTCGTGTGCTATAATGTTTTTCTCTGCTACCAAACTCCGGGAAACACAAATAAAGCAGCACAGCGAGCGG
TAAAGGGCAATTTTCTTTTCTTTTCTTAGAATCTAATCGGCAGGAAGCCGCTAATGTTGATGATGGTAACGGTCCTGATGC
TGACAACCTTGATGATGATCATGTTGATGAAGATACACTGTGCCTCTGCAGCGTTCATTCTACTGACAACTGCACCGAATA
GATCAACAGCGCCCCCCTGTGGAGAGGAGCTAAAAAGTCAAGCTTTAACAACTGTTCTTCTTACATTTTTCTTTTTGTTGA
ATTACTAGCAATAAGATATTTTGTATTCGTTGAAAATTAGATTAGTTTTACTCAAAGTTGTTATTTTATTTTCAGTTTTAG
AGGCAGTTTCAGATGGGTTCATCCCAGGTCCAATTACTTCCTCATTTAATCTTTCCAATCAGGTAATTCTTCACTTTTTTA

AATGACCTGGTGTTGCTGGTCAGTCCCAGTCTAACTCATTTTATTAAATGTTGACCATAAATACCATTTTTAAACCTAATA
AAGGGTCTCGACATTGAAAACCTTGACATTGATCTAAAGAAGGGCAGCCCAAAGTGGGGCCCGCCAGCAAAAGTTGCCCCG
TGCACCATCAGGCTCAATTTGGCCATCCAGATGTTTCTCGTGAGATAAAATGTTTAAGTTTGTTTTTTGTGAGGTGAAAAT
TCTCCTTAAAGGGCTAATTTGAAGGATACTTGATTGTTGAGTCCCATTCCCATCTCGTCAAATATCGATGCTTTGCGTTGG
TGGACAACCCGACCCTACCGACCCAAAGCATCAGTATTTGACGACCTGGGAAACCCAAAACTTTGATACTTGACGAGTTGG
AAATGGGGCTCAACAATCAAGTATCTTACACATTGGACCTTTAACTAATCCCTAATTATTAATTCTTTTTGGGAACTTCAC
TGAGCAGGAAGTCAGAACTTAGGGTCCTAGTACCATCTTGTATCTTAACAATACAATAGGTGTTTGCATAACAACATCTAA
TGTGTCAAAATGTGTATGATTTTTCCTGAAACATTTTAATTATGATTAATGCAGTTAAACAGTGCCCCCTTGTGTCAGTAA
AATAAAAAAACATGAAGCCAAGCTCACAGAATCAAGTCCAAGTAATGATCTGATGAATCCATCTGTGAGAACAAACCACTA
ATTACTAAGTGGGACTCCAAGGAAGAATTAAAAATAGTCTGTCTGAGAGATGTTTTAACCAGCGTTAATAAGTCTGAAGAT
CTGAGGCATGCGGTCATATGTGTGTGTGTGTGTGTGTGTGTGTGTGTGTGTGTGATTGTCTGTATGACAGATCTCTACATG
ATCATAAATGTGGATGAGGAGGCTGAATGTCTGCTGGCTGGAACAGAACGAGATAAAGATGGATTGAACTCATCACTGAAG
ACCGGGAGCAGACGTACAGATCCATCTCTTACCAGCCAGATTGCGTGTCTGTGGTCATATTGTATACATTTCCAAATTTAG
ACACTAAAAGAAAACATGTACACATGTACTTATATATCTTTGTTTGGCTCTGTGTCACCATTCATCACTGTCTTTCATCAG
CAGAGATACAACACAACACTGTGCTGCTGCTGCTGCTGCTGCTCTCCTGTAACCCGCAAAATCTCACCAACACACAATCCT
GTAAAGTGTGAATTGTGAAAAGGTTACTGCAGCAATTAAAAGCAACAATTACAGTCTGTGCTGTGGGCCGACTCTGCAGTA
CCTCGTAAAGACCAAGGAGTGGCAGCACTTCACAGCTCTACAGCTGATCTGTGAGAGGATCGATGCAGAGGAGAGAGGGTT
ATCTTTTCATACATTTACTGAGACTCCTACTCATGAGATAAAAGATCAAAATTCAGCCCCGGCACCTGTGCAGTCAGTGTT
CCTGAAAATATACAAATCTATAACACACTCTCTGTGAAAGACATCAGCAGGTAATGTAACTCGGTGTGTTTGTAGACTCAA
GTCTAAGACTGGAATGAAGCTGTAAGGAGCAAAAAACACCCAAACTGGCTTAAGGCCTCAGTAAAAAAAAATCGGATTGTG
GTTGTGCTGCGATCGACCAGAGGTGCAGAGGCCAAAAGTAAAGCAGGATTAAAACTTCTCTCTTTCTTTATTTTTCATACA
ACTACTTCCATCATGTTCAGTGTGTGACACCAAGTGAAGCATCATGCATGACTTTAAGGAAAGAAAGAGTCCAAAGGTTGC
ATCTCAACCTAATTCTTTTGCAGCAGATCTTAAAGGGACACTCCAGTGGTTTAGTATTGCATGTCAATAAAAGGTGAGGGA
CCTTGAGAGAGGCAAATTAAACAAAGAATGGTATAAATCAGTGTGGCGGAGGATGAGATATGCTGACTTTAAGTCCCTAGT
TTGGTTTCAGCTTCAAAAACTGAATCCTACAGCTGCCAAAATGCAGCTCAGCAACATCTTTTTTTTTTTAGATCCTCCCT
GACCAGTGAATGCCCACGTGTTTCAATCTGCACACTTTCAGCCTGTAACGCAGGCTTTGTCTCCAACCACACACTTTAGAA
AGCTCCTCCAGGACAAGAGAAGACTTTTCAATCAGGAGAGACAAGTGAGCGAGGTAAAGACAGTGTTTATCACAGTAGATG
ATACATGATAATTGAGTCTTGTCTTGTTTGTGAACGTCAGTAGTGAGCAGCGGGAAGATTAATCCCCCCACAGAGTCCAGA
CAGTGGCAGTGGCTGATGACTCTGCTGAGACCGATAGGCAGATGAAATAATGAAGCTGGAGTATCTGCAAAGGGCCGTGAT
GGGGAGGTGGATGGGGCACGTGAAACAGCGGTGAGGGGACTGATGTGAACAGCTGATAACAATCGACAGCAGCAATGAGTG
AGCATGCATGAGAGGAGTGAATGACTGTCTAAGCATGCAGAAGAACAGTTTTACTGACTGAACTTTCATGAGAGCTTCATT
ATCGAAGTGTATTCAGAGGTTTCAGTGAACCTGATGAGTAAAACTGGTATGGTGACACTGTAAGTGCCTAATAACGTTTGC
AGGCTGCGAGCATGTTGGCAAATAAAGTGTAAAAATCAAGAATGCTAACAAGAATTGATATTAAAACCGTGTTTCTGAAAT
CAATCACATTTTTGGTAAGGATCTGACAAATGAGCCGAATCCACACGTTTTTTCTTTCTCACTTTCTTTGACATTGCGAGC
TAGGGCGACGATTTTGTTCATTTAGTTAACTTCTCAGGGGATAATGCATGTATCAGGCTCGTATCTATGAGAAAGCACGCT
TAGATTCAAGGTCACATGCATGCTATTGTAAAAGCTACACGTTCAAAGAAAGAGTAGCTCACCCAAACTATTTTAAGAAGT
CACATGAGTAAAACCTATGGCCCAATTTACCTTATCAGCAAACTTTAAATGGATACCCTCCTCACTGCGATAGCTCTTCCA
AAAGCAATTTGGATCGTAGAGTAATGTGATTTTATATAAAAAGCAATCTCCCAAAACAACTGCAGTGAAAGACAGCCGGAG
CAGGAGCAGTAACTTATCAGGGTGTGTGTTAAACTACCTCTTGTGCTATTCTGCTGTTAAGTGGAAAAATGTTATTCCAGT
GAGTGAAGATTTATATGACTAGAGTCGCCAAATGGGAGAAATAACATGGGAGGCAGAAGAAGAAGAGAAGGTGAAAGGAAG
GTCAGGGAGGAAAAAGGAGACAGGAGCAAAATGGAGGGAGGCAGAAGGCACACGGGGACCCGGTGAGGGAAATTAACACCA
TCCACCAACCACACGAAGGTAAATTTAGGCCGAGTCTGTTATAGTCGTCTGTTTCAGCAGTCACTCAGGAAGGTGAACAAC
CACAGATTCAAGGCACCATTTCAATCACTCTGACATGAAACCCAGTATCTGCTGCACACATGCGTCTCCGAGTGCCGTTTT
TCTCTCGAGGTGCAGTTCGACCTGCTGTCCGCCTTTTATTTCCTGCCTCAGCCCCCCTCAGACGTGTTTTTCAGCCGTCCC
CTGCTGCCAACTATTAGCCTAGAACATTAGAGGAAGGTTAACTGCCTGCTAAACTTACCCGATGAGTCCTCTTGTCTGCAT
TAGAGTCCAGTATGTTGCCTGTTCCTAGTGTTTTTCCACTCATTACCCTCACCTGTGTTTCTCTGCCTCACTCCACCTGCA
CGTCATCCCCTCGTTAGTTTAGCTTGTATTGAAGTCCAGTCTTCAGTTTGCTCTTTGTCGAGTCATCTGTTGTATTTCCGC
GAGCCTGCTGTTGGGTTTTTTCCGTTTCTGATGTTCCACCGTGTGTTGCCTTGATCCTGAATAAAACTTCATTCATTGCAC
TTTCTACCTGCCGTCTCCTGCATTTGGGTCAAACATCCCTGTTTCGGTGTGACAAAATTAGTTAGATCATAGCATTTAATG
TTATTATTTCTACGATTTTTGGTTCTCTTTCTTTTATATTGATTTATATGGGTCTGCAAAGTTTATAATTGATGCATCAAT
GCAATGAATGAATACTCCAACAAATGAGCCCGATCTGTGATATGTGAGCAAGTTTGCTTAAATGTGTCTTCATATGTGTGC
CATACATGAACCCAATGAACCCCAATGCTTTGGCTTAAAATACCTGTTTTGGTCGCCACGATCACGGATGGAGTTGGTCCG
ACTTCCTGTGAAAAAAAGCCGGCTTTGGTCGCCAGAAAAACGGCATTTTTATTTGTATGTCACAATTATCATTACCTGCAA
GACTAAAGACATTCCCATTAGCTCCAGCTGTAATTTAGAGCTAATGAGCAAATGTCAACATGCCAAACTGTCATGCTAGGT
ATAAATCACACATTACCAGAACAGCCATCCCTCTACTTTACATTAGTACTTTACCTCAATCAACTTGAGCCCTGCTGACTT

TGCTTACTCACAGAAATGCTAAAACTGGCTGCCATTCATACATTACAGTACACAGAAACTAATTTAAAACACAAAATAACA
ATTTAGCTCCTTTGTTTCCAACTGCCTCCTACTTGAGCATGTGCCCCGCGTTTCATCGCTGGTTCACGGTACATGGCAAGT
TCGCAGGTCGACGTGACTTTTCGCCCTAATGACATTCATTATGAGCTTAATTAGATGTAGCTACAACAGGCCGCCGTGGCA
GCAAAGACGAATGCTGATAATTAAATTCCGGGCAAAGGTTGACTGGAGAACATGCGACTTAGATAGCGTCTGCCCTTTCTC
TGTGGCTGGTATGAGGCATAGAGAGACTGAGGATGACAAACTGAGAGAGATACAGGGCGTGAAAGTGGCAAAATGTCACAG
ACAGATGGGCCGGGTCAGATTGACAGAGAAATATCCACCAATACCGGCACAAAATGTTCTCCGAATGGTGATGCTGTCGCC
CCCGGTAATAAGATTAATCATTCCTCGAGTTTCACAAAAAAAAAACTGACTGGTACTCTTGCAGTTTTTTCATAGTTTAAA
ATCTGATTTAGTTTCAGTCAGACCCATCAGTGTTTGTTAATACCAGAAGATCGTGCTCCAGCATAATAGCTTCCCTCCAAG
TTTTGTTTCTCAGAACAGAACAGTTTGATAAACAGCCCATGGCTATTTATAAAATGTACTCATACAGACATCTAGATTAGC
CAGTTAGCCTAGCTGACGTTTACAGGTTCAAAGCTGCCTTGGCTGGTAATGTAGGCACTTATCTAAATACTGATATGACAT
GATGTGCTATAATTTGGCAGCTAGGGAAGCAGCACTTCTATAAATTGTTGTTTTTTTTTACTCTACCAATTAAAATTGAAC
TTTTAAAAAGGGCCACTTTGAGTCTCATTCCCAACTCGCCAAACACTGAAATTGTAGGTCAGGTTGGTCGCCGTCCCAAAG
CATCAAAATTTGACGCGTTCGTATTTGACATGTTGGGAATGAGAGTGACTTTCTGTCACCTTAGAGGACCTTAAAGAAGTA
CTTGAACATTTTAGTAAATAAACTTAATTGCATTTGGGCCAAGAGTTACTGTAGATGGAAAGAGGAAAAGCACTCTCATGT
CTGTATGTCTTCATGGTAATAAAAATAAACACATTTTGGTTGGGTTTAGGCAACAAAACTACTATATGTTAATGAAGAAAT
TACACTACATGACCTAAATTAAATACATTTCACGCTGGACACAACCTCCACCCTGCTTGGACTTTCCTGACATTATTCATG
TGTGGTTTTTGAACTCATCTGTTTAGTACTTTATGGGAAGAACTGGAGTATCACCTGCATGCAACACTGGACTTCGCCATA
TGTACTGCAAGCAATTTGAAAGTCTAAAGTTGTGTTATTTGTAGATGGGTTGAAGCCTATGATGCGACAGTATCAGCGACA
TCGACAAACTTTTTCACTAGCAGAGTGTGTAGAGCATTTCCAATCACAGCTCCTCTTAAAGTTATTATTTATGGTTATGAT
TGTAATGTTTGACAAACATCACTAGGAAATTAAGTCTTAAAAGCTTAACCTATACAAGGAGATTAATTCCAACTCTCAAAA
AGCCCCTTAACAACAACTGTCCATCAGTAAACAGTGCCCTTGACCAGGACCTGAAGCCTCGCGTGCTCACTCAGCGTTAAG
TTGCTGTGGCTGAAAGCATCGGCTAAATGTAAGTGGAAAATGTGAAAGCAAGTTAAGACCATGGCGGCGGCAGCGGCGGCG
ATGGGCTCTCCCTTTTTATCGCAGCTAAAGCAAAGCTGTGACTGAAAGCAATCACAAAGCCTCCAACATACATGCAGTAAA
GAGTGATTGTGGTCATGAGGCATGGCATCACAAAGTACCACATTGTGTTTATTTTACTCCAGCGCCGCCAAGGTCACACCA
GTGTCACAGCGCTTGTCAGTCACTGGAAACAGGCAAATCTCACGCGCTCTTTAGAGATGAAAAAGGCAATAAATGTAATTG
GAAACAGAGTAATGTGTAAAAATCACACAGACAAACTGGTTTATCAAGGACATGTCCTCTTTATCCATTTAATTCCCCCTG
TACTTTAATTTAAACTCACATGAATTGAAGTGTGTGTTCACTTGTGTGTCTTTGTGTATGCAGTGTCCGTTCGCTTGTTCT
GTTTCCAGTGCTTTCAGCCCACAAATGCTACTAATGCATTCTTCCACTTTTTGCATACCAACATAACATCTGAGTGAGAGT
GAACTGAAATGATGTTCATCTACAGTATGTCAGTGACATACCGGTTAATGTGCTGAGTGTCATAGGAGCAGATCATCACCA
AAAAACCCATTAACACCTGAAAAATCACCATTTACACCAGCAGGATGTGTCCAGGGAGCGTTTCCACTGTTCCATCATAAC
TGATGTTTTGGACACTTTTCTACAATAAATTAGATTTCTCCCTTAAACTGGCAATAAAATGTCAGATGCGTTTTTAAAATG
TGCTGTCAAATCTAATCCATCAGAGAGTCTACATACATATGTCCCCTTCAGGTTGTGTCATTTCTCCAATGATGCTGACAC
ACAGGAAAGGAGGAGAACCAAACACCTGAATACTCAATTGAATAAGAGTGTTTACACTTGAGTCTGGGCAAGTATCAGTCG
CCCGCGGTGCGATAAGCAACGTTTAAAGATGCATAACTTCCACATTGCCTGGGAGAGGTTGTTAAAAAAAAAAGGAATATG
AGGGGAATAACTTTGCATTAGAGATGAGGAAAAAACTGTCCTGCCAGCAAAGGCGCTACAGTATTTGAATTTCCTGAGGCG
AGTGTGAATGAAGTGCAGACACATGGATAAAATGTGAATGCAGGGTGAGAGCGAGAGGACGGACCTAAGAGTTCAATCGTA
AGTTATGATTTGCCTCTTAAGTACTAAAGTAACACTAAATATCTGTAGTTAGTTAGTTGGCAGGGTTGATCAGTAGTCAAG
TTGATTTATAGGCAAGAAGCAGTTTGACAAGTTATGTTAAAACATGTTAGTTGGGAGAATATGCTGTTGATTGGGTGGCTG
ATATTATGAGGATCCATTGACAAAAAGTTGGAAAAGTCAAGCAATTGCTAATAGGTTTAGGTTTTATTTTCATTGGCCAGT
CATTCTCAAACTACAGGACACATCTATGGTAAATTATAAACAGTTAACAGTCATAACAGAGAGAAAAGCAGCAAATGTGTA
TACTCAAAGAATAATGTAAATAGCCTCTCATTAATAAGTGAAACGTATAAGTATTTATCTTTAAGATTCAGCTCCACATCT
TCAGGTTCGCCTGAGGAATCTTTTCATGTTTGACATTTACTAAACTCCCGACGTCCCTGACATCTTTAAATGTGTGAGATT
AGCTGCAGCTCCAGGTTTTCTTTGCCCCGAGCTAATGCTCTCAGGCTAACACGGCCGCACCAGGCTAGGTACTCCAATGTA
TAGAAACAGATACCAACCAGTTCCAACAAGAAAAAAACTCAGGTACGCAAATTAAATGTTATTAAATTAGTTTATTAAAAA
GTGAAATGTGTCGGCCATTATGTGAAATAGGAGTGACAGTTTTAACACTAATGAGGCTGTAAATAATTGGAAAAGACAAAC
CATAAAACATCATCAACAACATCCTGGTCAAAGTTCATTATTCTGATAAAATAATAAAATAATACTAAATATCTTCACCAC
TTCCTTTGATTTTCGCTCTCTCAGGCCTCGACGTTGTAAACAGAAAGGTCAGAAGCCAAGGTCACGTGCAGTTCCCATGGA
AACTGATTGACGGGTCTCTTGACTTTGCCCTGCTGACCTCTGCTCTCCTTGATCATCAGCTGGACGTCGTCGTCGTCGAGG
ATACGGATGAGGTCGCCTTCGGGATCGCGGTAGTTCACGGCGATGTCGTCCACCTTGAAGACGTTCCTAAAGAGACACACG
GACAGGGAGAGGTTGACTTTAAACTCACTTTTTCCCCCTTTTTCTTATAATCATTTGAGTATCATCATCACAGCAGTCAGA
CTCAGGCTTAAACCAAAACAATACTGCCATGACTGAAGAATATCCTAAATGACTGATGTTGATATGTCCAAAAGTAAATTA
AAAGAGGTTTAAGTTCCTTTGTGAAACATCAGAACTTAAGTAAAAAACATCACATTTTAATAACAGATACGAAAAAGCCTG
AGAGACCAGTAGAAATGTAGCTGTGAAATGTACTTGAACCTAACATGTAATTAACGAGTGTTCTCTAACGCCACATATCGG
ACACAGGAAGTGACACGGCGGGACTGCTGACAGTGTCCAAAACACACGAAAAACGGTCAACGTGGCTGTCGAAAGACCCGC
TGCTTGTGGGGGTGCAGGCCCATTCAACACTGCTAACACTTTTAATTAGGTTTTGTATTTTTATTATCATCATATTGTGTG

GGGGCTCTTTTCTGGCTGCTCTGTGTAAAAAAAAATGCTTTTATATGTTACTTGTATTTGAGTGCTTGTTTACTTTAATGT
TAAAAGCCATAAAGATATTTAATGACGACATTATAAGAAACCTTTGCTCCATATTTCCAGTGAAGGAAATGACTGCCCCCG
CAAGGCTTTTGTTGATTTATTACAGCTTAATTAAAATTTTCACTGTGAAAGTTATCACCAAGAAAAACATGTGCTGGACAA
AAGGAAATGGGGTAATAAGTGCAGATAGGAAGTTTGACAACTGCACCACCCTCTCTATATTCACTTTTTTTTGCATGAATA
GACGGTTATATTTGTGTTGAGAATTAATAAGAGAGAAAGAAAAAGAAGAAAATAACAGAAAGAGACGCAGAGTTATAGTTT
ATGAGGTTCTCTATAGGCCCTGGAGGCACAACATGATGAAGTAATACATCTGATGGAGGACATGCAGAGAGGTATCCAAAG
TCACTTCCTGCCAGCAGCAATGGCAAGACTTTTTTTAGAGGACATGCATTCTCCAACATGAATCTTGTCGAGAAATGAAGC
TTAGTATTGAATTATTTCTTTCTTTTAAGAAGCAGTAAAATCATAACCACATCTTCTGAATATAAAAATTCAGTAGTATCA
CCACCGGCAGCACTGTTTACGTCTCTCTCTGCCCGGATACTGAAAGTGTGAGTGTGACAAAACGCCAAAATACAACGTGTT
AAAACATCCTGATGATGATCTTGTGAAATCAAAATAACACAAGAAAAAACATCTGTAATAATCACAAGTTAGTGTGATGTG
ATGCTATGTGACAGCTGATCAAATTCACTGGTTTTACTCGAATTTAAACTCATTCATAGTCGAGGCAAGGTATATTTTCAT
TCAGTTAATTATCATTTTCAGACTCCTTCGCCATCTTCCTCTTTCCTGTTTCACACGACGCAGTCCTTCTCCTCATACCTC
TGCTACTCCTCTCCTCCCTCCTTCTCTTCCCTCTCCCCCTCCTCTTTATCAGTGCCAAGCCATATTAAAACTCAAGACCAG
GGTCTCATCCATCCTACTCCAGGGCACAATAGGCCTGAACACAGAAAAACAATAATGAGCAGATAAGCGGAAGAAATGAAC
TGATTATGAATGGGGAGGGCTAATCACCACGGCGCTCACCTATCTAAATTACCAGATAATGAGAGATAGAGGGAGAGGGAG
AATGAAAGGGAAAAAGACGGAGAAGAACAGATATGATAGTGTAATGAGCATATGCATCCGGTGATGGTGGTGCAGTGGGAG
GGAATGGGGGGAGTTACGGGGGGAGTCAGAGGTCCCACAGGAACTAATTTGACGAACGGTGATGATTGGCAAGTGGCAATT
TGCAAGTTTGCTGTGCACAGTGGCTAAATATGCTGCCCCTGAGGGCAGGTTGGTGAATTAATGGACTTTTACTCATCCTTG
TTTGCTCTGGAATTAACACAGAAAAATTACTGAGGGAGTGAAATGCTGAAAGTGTGGCCACAAAGGTCAGCAATCACCTTA
GTTCAGCAGCACATCTCCAGGACAAAAACACTAGATATGGAAATCTGCTCTGCTGTGCAAGGATTTGAGAACAGGATGCTT
TAATTTGTGATTTAAATCACCATCTTTGAAAGTCTAATTTATGAAAATGGCTTCAATAGAACACTTTACAAATGCTTTACA
AAGACGAGGTCAAACATTACCGGTCAATTTGTACTTTTTTCAGAGATGTAATTCAGTGACACAAAGAACAAGAAGTAGGAA
ACTGGGAAAAAGGCTTACGTAAGTCTGCGATTATGAATGTGAAGATCTGAAGGTTTTAATGCAAAAAATTGCAACATGCTT
CACCGCCTTCTCTTCAAGCCTTCGTTAAATATGTCAGTTTTATCTTCAATTATCACATTTTTGGGAAGGTGTTGTAATTCA
CTCACAGGTGCGAGGTGGCCTACGATCACTTCTCCTTCAAAAGCGAAACAACAGTCAGCTCTCAACACCCACAAAAATGCT
CCTCACATTTCTCTTAATCACCGTTTCCCACACGAGTGTAAATGAAATTAAAAACATGACACAAAACACCGAAACATAACA
CAATGTCCGTCAGATCTTTTGTTTCTCCCCTAAAAATGGCCACGGGGCTACTAAAATTAAAAATGATAGCAAGTTAAAGAA
AGGTCTAGAATTTAGTGTACACCTACCTCATACGAGACAGAAGCTCTTTGTATGTAGGCTGGATGGTGAGGTCCTCTTGCA
CACACACGTCTCTGAAAGAAGGAAAGCACACCTTAAAGCAGATTTAAAATGTGTTTTTACAAGTTACAATCTACATTTTCT
AAGTGATAATCACAGAATTCAAATAAATGTCTGTTGAGTCACTATCTACCACTGAAGGTGACAGGATGGTGATGGAGCCTG
ATGCATCATGGCCCATGAATCGAAGTATTGCATAAGGTCATCCTCCTGCTGCTCATGATCCAATATGACTCTGAGTGTCTA
TTCCTTCAGAAGGTCTGGCTCTGCACCTGACGCTCTCCTCCCAACTCTAACCCAACTCTGAGCAGCTGGTCAACGGGTCTG
CCCCCCGCTCCACCTGGCCCTCCCACACAATACTCCACCAACATTTCTATATGCTTTGTTTTGAGTGAGAGACCCCTGGCT
GCAGAAATTGCATATTGTACACTTAATTTAGTAATGCAAACTGTACATTCTGCTGCAGCTTCACATCTAAAGCATTTCACA
ATGGCCATTTTTGGCATTTTTTGAGCGCTTGCAGGGAGTACAGTCTATGGAAGTACTGGAACAAGAAGGAGCAGCCAGACT
GAACCGTTAGATAAAGAGCTGCAGGCTGCACACCTCCACCTTCTCAGCGAGGTAACAAGCTCCTTTCACTGTGCCCTGCTG
TGTGCTGCGGCCAATTATTGCCTGGCTTCCTTTATTAAAACGACAAAGACTGTTTTGCTGCAACTGGAATTCTGTGAGCTG
TAGTAGTTAACCACATGTGATGTTCCCACCAGTAACTACAGGTGTTGCCAAATCAACTAGGACTGAAATCTTATTATGCTT
TTTAGTTTGTATCCTTTCCTTCAGCGTTTTAAAGATGTTCTAGTGCATGTCAAAGATCTTCAATGTTAAAAAGGTCAAAAT
ACACACCAACAGAAGCTCCTCTCTCCTACAAAAAAACACTGCTCCTAAACACCTCGTCAGTAGTCGCGCCCTTAATTCTGT
GTCTTTGTGACATCACACTATGTCGCCATGCCACACATTTGCATCATTTATGCCTCTTGGCTAGTTTGGCACCGAAGAATT
GATTTAACACAGCTGCTCTGTTGTTGCTAGCGGTGCTGGATCAGGCGTGTGTGAGCTGACCAATCAGAGTAGGCTGGGTAT
TCAGGAGGGGGGGCCTTAAAGTGACAGGAGCTAAAACAGAGGGGGATACAGAGCTGCAGCACTGGACAGTATGAGGAAACT
GACACGTTTTTTGATCACTAAAGCATGTAAACCTATTCTAGCAGACATTATCAGAAGCTGACACCCCAAACAATTCTCCCC
TCTCAAGATTTAAGGTCTAACTTCTGCATTACAAATTGTTCTTAGTTTCTATTTTTGTAAATAAAAGAATAGAACCATCAA
CAAAAGACATCCTGTATACTCGTCAGTATCACATTGATTACCACACGCACTCTTCTGAACCGACCTGGTGTCGACTCCAGA
GGGGGTGAGCAGGAAGCAGCGCAGGCAGCTGTACGTGGGGCATTCACCTTCACCTTCTGTGTCACTGTTTGGGAGGGGCTT
AATGATCTTCACAAAGGATTGTGGGAAAATACCCGTTTGATTGTTCACTGTGCCCTGAGGAGAAGACAGGAGGACAAACAG
GGACAGGAGAAAAAAAGAGAAGGGAAGAAGGATAAGGAGATGATGAGAAATAGCAAATGAGCAGAGTCCCAGATGATAGAG
AGATAAGGGACTGATGTGATGAGATGGGCTGATGATTTAAAGAACGCAAGGAAGCAGCATGGACGTGCATCTGGAGAGAAG
TTTCACAGACTAATTAGCCTGTTCATAATATCCTGCTGATGTTACAAGCACTCGACACAACCATACAGCCTTTTTACTACA
CCCCTGCTGATGAATGAGTGTGTGTGTGTGTGTGCGTGTGTGCAGCACGAGGCAACAGTTTTTTGTGCGAAGATCCACGTG
ATCAGGAGACATTTAGCCTATTTGCTATTAAGATACTATAATCAATTAGTTTAAGCATTTGGTAAAGAATGGGCATAATTT
TTTTTTTTTATGAAGATACTTTTCATCAGCATCAAGTGAAACAATCAGAATAACAGCTTTTGTTTACGAAGAACTGTGTTT
AACGCAGAACGCGTCCCATTTCTGGCTCCTACATTTCCCATAATCCCATTAATAATGGGGTTTTTTTCCACATCGTGTCCA

AACCCAGTTTTTAACACAGGCGACCTGTTATAGATCTGCATCCCATACAAGCCAGCACGACTCAGGTGATGTCACTTAAGT
ATATCACTAGATACTCCAGAGCCATAAAACACATTATTCAACTGTTTTTTCACAGGCTGAGTAGTACTGCTCATGACCATT
CGGTGTTTGTTATGTAAAATCAGTGGGCTTCCCTTTTAAGGCTAAATGGATGAACGATCTGTGCACACATGTTTTTTTTGT
TTTCTTATAAAACCTCTCTCTGCATTAACACCTCGGCGCAGCTCGCTCTCTTTACTCAGTGCAGCGATGGGAGAGGGAGTC
TGTTTGTTATTGGCAGCCTTTCAGGGTGAGGGTGTAGAATATGTAGTTAATTAGTGCTCCGAGGAAATTATAAAGTATAAT
ATTAATAGTTACAGTGTTTGTTTGGTGTAAGGAGATCCAAATACAACTTTGTTCTCCCACTTCTCCATGTATGGTGCATAG
GAAAACCTAATGTCTATAAACAGATATCTGCATATAAATGCAGATTCTGCAGGCAAGAGACAAAATTCTGGTATCGACCAG
ATTACAATGTTCGTTTACGATGACTTCCGGGTAAAAACAGCTGCATTGCACATCAGCTTACATATAATTTAACAAATATAA
TTTATTTTTTCATGATGTTATCTATGAATTATGTTAAGTGTGTGTAAAATGCATATTTTATCCTGTCATATCTAATTAATA
GCAACACATTCATTGTGTTAGTTTGGAGACCTAATTTACAGACTACAGAAAATGACAGTTAGCAGTTAGCATAACACGTCG
CTGTTGTGTAGAAGACGGCAGGAAAAGCTATATAGAACAAGAAACCACAACAGAAACGTCAGATGGGTTTGACCACCGTGT
TTAACAATGTATCTTTGCAACTTAGAGTCACGGCTGAAAAATGACACCAGAAACAAACAAGCGCAACGATGATTCAGTCTA
TTGGAAGTGTCTGGTTTATGTTTGTAATCCGAGCAGTATTGACCAACCACGTTTGAGCAGGTTATAGTTGTATGTAAACAG
TTCATGCGGAGAGCATTGGCCAAAATGTAATCTTGGAAACCATCAGCTGTCGTCTCACAACCATTTAGCTGTTTATTAGTT
ATCTGTGATATCTGTTTACAGAGATGAACTAATCTGCCGTCAGACAAAGCCGATGGCTTCAAAGATTGCTGATCGGACTGT
AAACAATGACAGGTGCAAAGACGACCAAGACTCCATATCTGGCTCCACCGAAAGCCACGAAACATTGGCTGTTGCCGCCAG
AGGCTAAATCGTTATCAGATAATAGCCGATGACTCCGTAGGATTTAGGACAACGTGACTGGTATATGTAGAAATGATGAAC
TCACCGTCTATCTGAACCCAGTGAGAAGTTGCAGCAGAGGGGTTAGTGTCTCATTTCTACAACTCTCAGTCAGGTTACACA
TAATTCATCATTCTAATAAACTCTGAGTTTAAATTAATCCTTTAATTCTCGGTTTTTGGCCACCCCTCACAGAAAATGCAA
GCTGCTCCTCACATACTGCAGAAAGCATTTCTCATTTTCTCCTCAAACTGTTCAGGTGTCTCTCCACTGGTGTGTTTGAAA
CGTTTACCTTGATGCACCAGTCTGCCAGTTGGATTTGTCACGTTTTCCACCCACAGCCGCTCTGTCCACCGACTAAATTAA
AAGGCACTCAGTCAACTTTTCACAAGTTTTAAGTGTTACATTAACCTCGCCCACCCCCAAAAAAACACAAAAAGCTCCATC
CTATCATGGATGCAGGGTTGCCAAAAAAGAACAACAGGGTAGTGTTCTGCAGTGTTGTGTAAGGTTATTATTATCAGCCTT
TTCTGTTACGATTTTTGGATCTCTCTAGAAAGCTAGGGGCAGGATAAGTGCACCGGCCTCCTCCTCCATGTTGATTCTCAC
TCTCTCTCTCTCTCTCTCCCACCACATCAGCTTCCACCTCCTCCCTTTCTCTCTCTCCCACTCAGCCACTCACCCACTCCC
TGCTCCTCTCTCACACTCCATCTCAACACCTTGCACGTTTTAACTCAACATGTTTCCTGACCTTTTCTTCAAGTACTCGTG
TGACAAAGCAGAATTTTCGGTGACAACTATTTGCCGCCACCGATGTTCCAGAGTTGAATTTTAATGTTCAGCAGCTAATTA
AAATGTCCCACATCCTGGTTTTTGGACGACTCACACGTGACAGTTGCAGTTCGTATTCAGGACATTTTGTTCATTAAGTAA
TTGTTCCTCTGAAGGAAACTGGAGCAGGGCACCTCCAATCGCACCCGACGCGATGCTCTGTGGTCGCTGTGCTCAGCTCGC
TGCCATGTGGGGATAACAGAAGTGTTAATCTATTTTTAGTTGTATTTTATTCTGTAATTAACTTTTTGACTGTTGCAACTT
GTGAAGGGGCTGTTTTGTGTACTTAAAACATTTGGCGAGGTAGAGATGCCCAATAAAAAAATATAGAATTAAACAAAATTC
AATCGTTAAACATAGCATATTCATCTGCTGCTATAACAACCTCCACCTTTAAGATTTTGGGACCTGGCTGCAGGGATTTGT
CCACATTAAGCCACATTGCAGAGATCGGGCATGATTCTGAATGATCGAGACTGGCTCACAACGTTCCAGTTCTTCCTATAG
GCGTTGGATGGGGTTGAGGTCCAGTCTCTGTGCAGACCAGTGAAGTAATATTATGTCAAGCAGGCCCTCGTGGTCAGTAAG
ATTAGGGGTGTGAGATAGAGTTACTCATGATACGTTACTGTCACAATACGTTGTCCAGGATAACAACAGTATCAAAACAAG
ATTGTTAAGCGATGCTTGATACGTTTCAAGACAATCATCTACGACACCTCATGATGTCTTTAACTGAAGAAGAAAAAAGCA
GTATTTATGCATTTATTCCCTGCATTTTACAGGTGAGATGAAACAATGTTCAAAAACTGCAGAGTGTAAGTTTAGTGCCTC
TTAAAGGGCCCTGACACTCTGGGCGGTCGACAGTCGGCCGATGTCGGGGCCATCGGTGAGGGCCCCAGTTGTTGCGGTTTC
TCCCGTACCGTTGGCACTTGTCGGTCTTTGTCTGTGGCTTTTTGGACGTGCAGAACTGTCGGCAGAGTCGGTGAGAGAAAT
CACTCTGATTGGCTGCTCATCGAAGCCTATCAGTGCCCCTTGAGCCTGTCGCTGGATTATCCATAATCTCACCCATTTCTC
CTTGATTAGAAGTGGCTATATTAGCAATAGAGTTGTGATAAAATGTTGCTTTATCATAAAAATAATTTTTACATTGACAGT
CCCGCATCTTCTGGTTTCCCTTTTTGAGTGACGACTTCAGACTACCGCCACCTGGTGGCATGGAGAGGTACTTCCTCTCAT
ACAGGCGCAGAACGTCCAAGCTAGCTGGCCTTAGGCTGTAATCTTTGCGGTGCGTTCAAGTGCAGCTTTTTGGCCAAGACA
CAGGCTACACAACAGTCAGCCTTTATCACCACCAGTTTTTTGGCATCGGTTTGGTGTGTCACACACATACACAGTTTTGAT
AATATATTGCTAGAGGAAGCGATGCGATATATTCCCGTATTGGTATTTTGTCATCTACTTCAGATATCTTTGTCCATATAC
ATTTGGCCACCTTTTGGAGGAAATGTAGAAAAGGTGCAATTCACTCATTGAGGTGGCAACAAAATCTCCTCATTTCTCTTT
TCATTCAGTTTCCATGAATTCATCCCTTCACTCTTCATCCCTCCTACTCACTTTTTCTCTCTCCCCCTCTTTCCCTCTTTT
TCTCTCCATCCTTCCCACTGTGTCTTCCTCCATCGCTCTTCTTTCCTCTTTCATTCTTCCTCCTCAGTCCTCCCCTTCACC
ATCCCAATCAAGATACCTCCTTCTCTCTTTCCATTTTTTCTCTTCTTTTCTTTCTTCTTGGCTCAAGTCTCCTCTCCCCCT
CCACCTCCTCTCACTCCATCTATCTCTGCCTTCCACTCTCCCACTCTTTCCCTCCCTCTCCCTCTCTCTCTCTCCCCTCAC
TCTGTCTCCCACTCTCTCTCTCTCTCTCTCTCTCTTTCTGTACTTACCTCCAGCCAGTCGGCGTTGACTCGTTGCAGCAGG
AAGATCACCTCTCCCCTCTTCAGGTTCAACTCCGCCTTGCCGTTGCCGCGAAAGTCAAACATCGCCTGGGACACACACGCA
AACACACACACACACAAAAGAAACCACACACACACACACAAGTCAGTGAGTGGAGAGAGGAAGGAGAGAGGAGAGAGACGG
ACACACTCAACACTAAGAGAGAAGAGACGGTGAGAGCGGACAGGTAAGACTTCACACACTTTTTTGCTGACGGAGAACAAC
GTGCGAGGATGTAAAAGTGAGAGAAAGAGATGGGAAGAGAGAAGAAATGCTATGTTTTTCTCAGTTTGATGTAGTTAAGGC

TTGTGAGCAAATTAGAGCAAGTGTAAGCAGATCTGTGTGGATGTGTGGGTTTTTGAGAAACAAAGTGAGGGGATGCGAGAC
AAGACAAGAATGAATTCATCGATCAAGTTGTGGGGAAGGCGGAGAACTGACCCTTGAGATGGGTTTGTGTTTGTGTGAGAG
TGAAAGAAAGTGAGAATGATAGAGCAGCATCATTTCAAGTGTTTGGTTATGCAGCTCTTCTGTCTGTGACACCAAACATCC
TCGCAATGTGTTTTGTTTAGTATTTGACAGCACTGGTGAATTTATTATAAGTCTCTGAAAGTGTGTGTAGGAACTGCAACT
AATGATTATTTCATTATCATTAATTATGTACAATATTTCATTAAAATGTAAGAAAATTGTGAAAAATGCCTATCACAGTTA
CCCAGATAGATGTCTTCAAACGTTTCGTGTTGTCCAAAACCCAAAGATGTTGAATTTATAATGATTTAAAACAGAGAAAAG
CAGCCAATCCTCACATTTAGGAAGCTGAAACCAGCGAATGTTGAGCATTTTCTTGCTTGATTAATAACAGAAACTGTCAAT
CACCCCTGACTAACTGATGAACTGACTGAACATTCAGTCAGCAGCCCTTCTGTCTGTTTATCTGAGCACAGTGTACTGGTT
AGGAATAGCCGAGTATTGTTGCATCTGTGTGAGAGTGTGAAAGAGTTTTTTTGTGTGAGTGCGCGTCTGTGGGCGGCGAAG
TAAAAAAGCATGGACGAGGAGTTTTTTGCAAAAGTGGTACAGCCTGAGGAGATGCCACGCTGCAATGCAAGGGTGTGTGTG
TGTGTGTGTGGGAGTGTGAGGGGTAGGATGCTCGACCGTGCTGGTTAGCATCGGTGCGTCTGGAATAAAAGCCTCTGCCAG
AGTCGAATTTGCATTTTAAGTAGAACTCAGTAGAGTCATTACTGATGCGTTAAACCTTTTTCTGTCCCGCTGCAAAGAGAC
TCCTCTAACAGACGGATGATAAAGTAGCCTATTACTCACACAAACACACACACACACACACACACACACACACACACACAC
AGGCTTTTGCAAATGCACAACCATACACAAGCACACACACAAACTACAATAATCTCTTCCCTGTAACAGCACATGTATAAA
TGTAATCTAATGTGCACATGTAGATCCAAAAGCAAACATATGAAAGAAAGAAAGAAAGAAAGAAAGAAAGAAAGAAAGAAA
GAAAGAAAGAAAGAAAGAAAGAAAGAAAGAATGAAAGAATGAAAGAAAGAAAGAAAGAAAGAAAAGCAAGAAAGAGGATTA
ATAACGTCGGACTGAGAAATTGCGGAGAGAATTACCGCCACAGTAGGAGAGCAATCACAAGAGACGCTCAAACTAGGAAAA
GAGTTGGAGAATCAGGAGACGAAAAAAGACCGAGGAGCGGTTAAAGCAGTGGGAGGGAGGTGAAGAAGAGGAGGAAGAGGA
GGAGGAGGAAGAAGAGGAGGAAGAGGAGGTGGAGGAGGAGGAAGAGGAAAGAAGAAAGTTAAACAGAGGTGCAGCGCTGCA
GAGGGCTGATAGATCGCTATCCACACAAGTGGCCGTGTGTGATAACCCTCACAGGACAGTGAGTGAGTGACGATGTGAGTG
TAGAAGAGATTAACTATCAATAAATACATAGTAATTACAGGTCAAATGATGCAATGTGGCTCATAGTAATGAATCCACAGA
TTATCATGGACGCTACAGTGTTTTCCACTCATTTAACTCATTTTTGTGTCTTGTGCAGCCTGCTTTACTGTTCTGGTTCAC
TGTCACCACTCTTTGCAGCGAAAAAGCTCCGATAAACACACTGTACTCCACTTGCCGTGCACCAAACAACGAGACAAAGTT
CATGACCAACTGGTGACCAAAGTCAGCATGTAGCACCTGAAGACTCAAATCAGAGCTAAAAGGAGAGTGAATATTTGGCAG
ATGAACGGAAATATGACTAAATACAAATGCTAATGCTAACACGTTGGCTATAACAACTTTAAAAGGTGAGATAATATGTCA
GTGTTGTGTTTGTAGCTTGTTGCCCCCTAGATGCCCCTTAAAGCAGGTTGAATATTTCATCCTGGTTGATAACCTTCGCAA
AGGAGGTTGTGTTTTCACCCGTGTACCTTTGTGTTGGTTTGTTGGTTTGTCAGCAGGATTACACAAAAACTACTGAACAGA
TTTCCATGAAACTTGGATGGAAGATGGGTCTCGGCCAAGAATAGACCCCATTAACTTTTGGTGAAGGGACGGATTTTTGCA
AAATTTTCTTTAATTTCTCAGGAACTAATGTACGGATCTTGATGCAAAGAAACATCTGGTGTATTTTGGTGGCGTACAATT
TGATGCAGATCCTCCAGTTTGATATTGGATCAGGCTTGATTAAATTAAAGGGGGACTGTTGGGCCTTAGCGGAGGTACAGT
ATGCGCTCTACTGAGTACCAATCTAGTTAAGTTCTGTAGTGATAACAGCAAGTGTGCTCGAATAAATCAACCGCTGGGGGC
AGCAAACACACTTTGAACAGCTACTCAGAAGAGAAGAAGGTCAACTCAACAACGCATCTTTCAAATCATGGGCTGAGGGGA
TTTATCACATCAGAGAATCAGCCTTTTGTCCTTCACATCACATCCAGTACCTGCATGCGTACTCTCTTTCGTGCATCACAG
GTACGCAATTTACATTTTGTGCAGAAAAGGCGGACTCAGCCAGTAAAACTTTAAAAACTACTGTATCCAGAGTGAACTGTT
GAATACATCAAACGGCTCGCGCTGAAAAATTGGGGTTGACGATTCGAAAATCTGAAAGGATAATACTTTTAAAGTAAATGT
TCAAGTGTGTGTGCATGCATGTGTGTGCGTGTGCGTCTGCAGAAAATGAGACAGAGGCTGATGGCAGATTAATGAAGCTGG
GGAGTGAGGGAGCTGGCTGGATTAACAATCCACTGAGATTACCCTGACCCTGAGGACACACACACACACGCACATGCACAC
ACGCCAGCACACACACACACACACACGCACACGCACACACGCACACCACACACACGCCAGCACACACACACACACACACAC
TCTTTGTATCCGGAGCTGCTATTCTTAATGCATTATTCCAGACGCTGGTTTAATATAGCGTTGACCCGCCGCTGGCTGTAA
TTTATAGATGATGTGAAAATGTGTTGGAGGTGATAAGCTACTATCTCCAACACAAACACCAAATATGATGTGACAAAGCAA
TTCCCCCTCACTTTGCACCTCCTTTCATCTCTCCCTCCATCTCCTCACCATCACTGTTCCACCTCTCCATCCCTTTACTTT
CACTTGTCGTTTTCTCTGGTCTGGTTTCTTTCATCCAATTTGTTTTTTTTCTCCCCTCTCTGTCTTCCTCTGTGTCTCTAC
TAACTAATTAAGATGTTGACTTGATGAGTTAGCAATAACAACTTAGTGCCGTGGGTAATACGGCAGATGAGTGTATGCTAT
TTAAAAAGCTGCACACAAAGAGACAGTTGGACGTCGGTGTTTTTAGTCGAACATTAAGCATCTGGCAGGCAAAAATAACTT
AACTCTGAGATAAATAAAATCAAGTCCCAAAAGGCTGACAGGGAAATAAAATCAAAACTGGGAATTTGGGGAAAATTTCAA
TTATTCACAAGGCAAAAAACCTTAGTTTTCTAAGTGAGCTAACTTAAAGAAACATAAAGACAGACGATGCCAACACAAAGT
CCATCTTAAAACAACGCCAGCCAAGTTCTGATTGCAAAGGTATTTTGCGTAATTAGCAACAGTGGACTCAACTCATTGGTT
TTTCTACAGTTTTGTTTTATTCAAACAGAATAAGTTCACCACATATGTTCAGAATGTTTTTCTACACTTTGCCGCCTACTG
GCTCTGCATGCTTGTCTGAGCATTCATAATCATTTCTTCATTCTCTTGTGTACAGAGATATTTTGTAAAAAGAAGTCATTT
TCACAGATTTTTTAAAGAGGAAAAATATCCTCTTATGTGTGGACAAGAAAACACGAAGATGTGGGCAGTAACTCTGAGGTC
TGAAACCAAGTTATCACAAGATGTGGCAAGATGTTGAGATCTGTTGCTGCAATTTGTGCACCAGAACCAAAAAACAATGTA
ATTCTTAGGCAACTGCATCACCCCGTGAATCACAGAGGAAGAGCTTGACTCTGTGATTCACCATTTTGCAATGTTATTTAT
CGTTATCAGGCAGGATGAAATCTGTCATAGAGGAAGCAAAGACACCAGTTTCTTTACCTAAAGCAGCAGCATCAGAACAGG
GACAATCTAATCCACTATTTTTTTTATAATTCAGCATGTAATGCAAAGCAAACAGATCATTCTGTCTCCTCAAGCTAAAAA
TATACTCTGTTTTATCCAGAGCAGGTTGATGTTGGTGTACCTCCTGTGCCCAGTAAAGGGCCAGCCTCTAAATGTAGCCAC

ACAACCCTTGGGTTCAGCCGGCCCCGGGAGCAATCATCCGGTACAATTAGACTCGCGCCGACCCCGCCTTAATGAGCTTGT
TAAACCCTCATCTCGTTAGTCTGGCAATAGGGAGGTGGAGATGAGAATTAGGTTTTTTTTTCTCTCTCCCTGACGTTTAAT
GAAAGTGTATTTTTTTAAGTAGATGGCCCACTTCCTGGAAGGCCTGGAGTGAAGGATCATAAGAGGACATGCTTGAATATC
CATATAAAAATCAGCAGTGTGTGTCTGTGCAGGTCGCTTCTGCTCTTCAGCTGTCAGATAGAGAAAATACAGTATGCATTA
GGGGTTAGCAGATCCTACACTGGGCAGTGATTCAGACTATGTTTTGTCTTCCTTTCGGATTGCAAAACCTGCTTTTAAATC
TGGGGACTTTTGCTATATTCATGCATGTTGCAAGCAATTTTGCAATCATGGAGCATGACAGGCATCCATTTGAAGTGACCT
GATGACTGTTAGTCTAACATTTACTCTTACCAACTCCTGAGAAAAAAAGATCCGGGTCTTTAGCTGCTAAATGGTCCACTA
TGCTCACCAGCTAGTCTCTGTCTTTGTGCTTGGCAGGCAGGGTACAGTGAGGTCCATCAGAGCTATTCTGCTGAAGACAGC
TTCCTGCTGCTGCTTCATACGAGGCTAATGGGAGTGGTGAGAGTGCACCAAACGGTTCATTTGCTTGGAGACAGAACAGTA
AGCAACAACTAGGGCTGCAACTAATGATTATTTTCATTCATCCATTAGTCTGCTGATTATTTTCACATATTTTCAATTAAT
CGTTTCGTCTAGTCCAAAACCCAAAGACTCTTCATTTACTATTAAATATGACAAAGAAAAGCAGCAGAACTTCACATCTTA
AGAAGTTGGAACCAGTAAATGCTTGACGTCTATGCTTTGAAAAGGACTGAAACAATTAATCGATTATCTTAACAGTTATTT
AATTATCGTTCTAGTGATTCATCAGGCAATCGACCAATCGCTGAGCAAATCTGCTCAGTAGAGCTGTAGGATTAAGAGATA
ATTCTCTGCAGTTACATCAGTGTGACTCACCTTTTACATTAGGAAAAGCCATTTGATATATTGTTTGTATAAAAAAAATAT
AGTCATAGAGTAGTTAAGCCGTTTAAACCCAGTTAACTGGAAGAAAAACACACACAAAAGGTTGTATAATCTCAACCTTTA
TAGTCACTGTTTAAACTATATGCTTAAAACTGGTAAAAAATACAATGCCATGATACTGCCGCTCTCAAATCTTGCATCAAA
TCTGCTAAATTTATACTTATTTCATCACAGTCGTTCACATTAACATACACAATAACCTTCAGCAACAATAATGTCACTTCA
CACCACAGATAAAAGTGAACATTCGAGTTTCATGGTCATACTTTCTTCGTGGTGAGGTTAAATACAAACTGTACTTGCGCA
TCAGCTTTTCTTGCTCGGGTAGGTTTTTATCTAAAATAGATCTAATTTCTGCTCTAATGGCTCTCAGTGCAAAGGAAGACG
GGCTGAGCCTCGGTCGATAAGCACCTGCTTGAATTTCAATTTCCTAATTTTATGGGTTTTTCTTTCATTTTTGCACTCCAT
CTCTCTGCATCTCCAACACACTCTCTGCCTCTTTTCTCTCTTTTATCTACCCCTGCCTCTTTGTCTGTCACTCCCCAGTTT
TTTCACATTAGACAAGCCGGCCCTCCAGCTCGTGAACCGTTAAAATCATGTTAACTAAGAGCAGCCTGTCCCATTGACCTT
GTTTGAAGCTCGCAGTGCTACAGCCACCAACAATTACCCATCACACTGTTTGGGGCTAAGCCTGAGCTAATTCTGCAGTAA
GTGGAGTGGAATGAAGTGTTGTAGCGTAAATGCTAACGCTTGCACCAGCTCTACTGTGACAAATTCTTGTGTTTCCCTCTC
ATCCATCAGTCTCAGACCAGCCATTGATCATTGTTTGGACGGACTTTAACAGCCACTTCCCTGCACGGAAAAAACCCACAC
GGCAGGACAAACACGAAACCACACAAACGCACAGAGAAGCACTCATTTTTCTCAGAGAGAGAAGGAGAGAGAGGGCTAAAA
TAAATGTTGAGTAGGCTGGCTGCTGCTTCGTGAGTAGAGTACACCAATGTTTTACTTGTGTGTGGGAGAGAGGGGAGGCAG
AGAGAGAGATACAGAGAGTAAGACAGAGAGAGAAAGAAAGAAAAAATAAAGAGAGAAATTGAGTTGTTTGTGCGTGTGTGT
GTGTGTGTGTGTGCGTGCATGTGTGTGCGTGTGTGTGTGAAAAAGAGCTGTACCTATTTCTGTGCAGTGCAAGGTTAAGAA
AAGTTAAACGCTTTTGCGAGGGGATAAAGATCATAAGAGTTCAAGAGCTGCAGAAGCGCGCACACACTTCACCTGCAGACA
CGTGCTCACATTATAGGCTGCAGTGTAAACACAGCCACTTACAGAGAAGGTGCAAAAGTGCATCAAACACACAAACACACA
CACTGCATGGAATCACAAACACAGCTGCTTTCTAGCAAGCTTCATTTTCCTAGATCAAGTTAAAAAAAATCTGCCACTACA
GAATGGATTAGTGCTGCAACTATTGATTATTCTCTTTACCAATTAAACTGCTAATCTGTTATTTTCTCAATTAATCAATTA
ACTGTTGAATTAAAACCTAATAGTCTGAAAAATCTTGTCCAACAGCCCGTTATCCCGAAAAACAAACACCTGCTCTGAAAA
TACACAATCTCCCTGCAGTTGTTAGCTCAGTGACCACCAGGGGGTTTGGCCTTTAATGGACTATATGTCTAAGACCGTGTT
TTGTCCAACCGAAAAGATATAAAGTCTACTATGACATATTTTGATTGCAAAATGACATAAACAGTTAATCGGTTATCAAAA
TAGTTGCCTATATTTCTGTCAGTCAACTATTAATTAATGCAGCTCCAGTATGAATATTTCTGCAGTAACTGTCTCCAACAG
AAAAAGAGAAAAGACTAAAGTTTTTATTGGAATAAAAAGTTAAATCTGATTTCTGAAACACTTGCATGAGCAGATTTGCAT
TGGAAAGAGTTTGAAATTCTCTTGCTGCACTGCCACCAACTTATTTTTAAGCTACATGGCAGTTTCTGCACAAATCTGGAT
AACTGGCAAGTGAAAAAAAAGACTATTCCTAATAAACATGTAATGTTGCAACATCAGAACTGGACTAAATAAATGGAATAT
GTGGGCACCTTTCTCTAAAAATGTCTCAACGTGGGCTGAACTACAAACAAAAATGACTTCATAAGAGAGGATGCTTTGTAC
AAAAATAACAAACATAAAAAATTCAATATATTTCTTCACACCACAACGTATACCGATAGAAGATCACAGTAAAGTAATGGC
TCTCATCCACCAGTACTATCAACATGATTACTCCCACACAAACAGTATTACCCACAGTTACTACAAGGCACTATTTCTTTA
TTTTAAAAATACACATTTATGGTGATTGTAATTGAGCATGTGCACGTGCATGTGTGTATGTCTCTTATATTTCTCTTCGGC
TTTGAGTTTTAGACGTAAAGACTCAGGACATTTTTGGAAGGAGAGGTCACTTTGGCTGCTCCTCACTTCTTCAGAGAGCTG
TTTGACGGTCAGATTTATAGTCAGGCCAAGGTCAGGGCAAAAAATGGGATTAGGCATTCTTATTGTTAGTTTATTTGTATA
GCATTTATCAAAACCGAGGTTACAAAGTGCACCAAAACACACAAATGAAATACACAAACAGAAGATACTGATTATAGCCAG
CTGTAGTTGCTCAGGCAGGCACGAACAGCAAACGTTCGGTCTCTGAAGCAGACTGCATGCTGGGTGGTTTTGGGACAGCAG
TTACAAAATATATATTCTAGGCCAGGTCATGAAGGGCTTTAAAAGTAAAATGGTTTTTGTCAGGAGTCTTGAGATCGGGAG
TCTGGTGATTAATTTTTGCCTTATAATTGTCAAAAGACCGTCCCGATCATCCAACTGTAAGGAAATAAACGCATGAATGAT
AGTTTGACTGGCATTAGATGGTCGTAACACGACTGGATAACCTTGCTGGGATGTTTCTCAAAACTGAAGTCGAAGAGAACA
CCCAGGTTTGTGACATGTGGTTTAACGATTGTTAAAGTTAGTGTTAGAGGATACTTTTGTCCTCAAAAGAATAGAAGTACC
AACATGTGTGCAAAGGTCAGTGTAGGATTGTGCTGATTCTACAAAAGAATAAAAAAGCCCGTTTGTGCCTCCTTGGAGAAT
TAAATTGGCAGGTCATTACCACTTTAACCTCTGCCGTGACTGTAACCACACCAGCTACACAACTTCACAAACACACATCTT
AAAACGACCACAGAATATAAACACTGTTTCCCTTCCCTTTTTTTAACGGTGCTGGAGCGGAGGGTGTGAACGGAATGAAAA

TGAGAGGATGCGAAAGGAGACGAAGAGGAGGAGGAGGAGGGGGAGAGAGGGACGAGAGATGGTCGTAACCTTGAGAAG
ACGCAGAGCGAGAGGAAAAGGGAAACAAGGCCAAACTTAAGGGTTGTTTAAAGGGAATTGTGGGGAGGGGGGAGAGAAAGC
GAGGAGGGTGAGGGAGGCTAATACCGAAGGAGGGATGGATACAGAAATTAAAAAAGGAAGTGAAGCGAGGCGGGAACTATT
GATGAGGTCAACGGAGTCACAGGCAAGAGGGAGGAGAAAAAGAGCGACGGACATTTGATAAATGAAGTCATCCTCATTGAT
TTGTCACATCAGTCGATATGTTTGTGTGTGTTGTTTCTTTTCCATGTTTGTCTGTGTGTGTGTGTGTGTGTGTGTGTGT
GTGTGTCTGTGTGCGTGTGCGTGTGCGTGTGCGTGTGCATGCCATGAGTTGCAGTCACGCCAAGGGTGCTGACAGGATCAC
ATGGTATTTCTCGGGGTGATGAAGTGCATTCTGCGTGGTTGTTTGACTAAAAAGTTGTTGCTCTGTGGGTGGCCATGGAGA
TTGTAACCAAAGCAACAACAATAAAAAAAAAAATGTTGCTTCACATACCTCGGCCCTGGGGGAGGAGAAGAGGTCCATCTTT
GGTTTGACTTTTCTCCTAAAAGACATAGAGATACAGAGATAAGATGACGGATATAAGTGAACATAAATGTTGCAGGTGTGA
TGAACAGCAGGATAGATTAAGTCTTGATGGGAGATCAGAGTAGTGGAGCGCTATTTGCTTTGCATAGTTACACATTTCCTG
TAAAAACAGGCAGCTAAGGAGAGACTTGTCGCGGATATTGATTGGCATTTACAGCAGCCAATAGCGCTGAATGTGCGTTAC
GACTTCCTGCCATGGAGCTAACGGTATGCCGACTCTTGCCCAACTGGCAGCTCCTAATGAACACAGATGGAGCAGGACTGA
ACAACACAGCCCGGTACCAAGGTGGTTTTCTCAACAGGTTCGGGTCCCGACACCCCAGGGCTTAGTCAGCAAATAGGACTG
CACGGCCTAACTTTACCAAACAGCAGCTACATTTAGCAGCAGTTAGCAGTTACGTTAGCTACAAAGCTCCTGGTGTCTGTC
CATCAAAAAACTCCTTTTATCATGTTGGTACTGTAACTGGCCTCCGTCATTCTCTGAAGGCTCACACTTGGTCTGGAGGCG
TGGTCAGTGGCAAAAGTGATAAAGTTGTGTTTTTGTACAGTTATCCATGATGCCTGGTCCCCAGAAACACAAAGGATTAGT
ATATGCGGGGGGGAGTGCAGGATGAGTTATCAACTGTATTTTACTGTTTTCCAGGAAATCACAAACTCTTAAACACGATTA
ACAATAAATACAAAAAGACTTACTTGCCATTGTCGGGCCTCATACTGGCGTTTAGGTGAGATATAGAGCAAGACTCACACA
TTGATTTTGACTTCATGGGAACTCATTTGAAGTCTAAAATTTTGACCTTTAATTATAGTTCCTGTCAGAACCAATCAGTGT
AGCTATTATTAGCCATGCTAGCGGCAAAGTTATAGAAATGGCAATGTTGATCTAGCACAGATGTTGTTGGTTCACAAATGT
TTCAACAACTGTTGGATGTATAAAGATGCACTTTACTGTAGACATTCTTGTTCCCCTCAGCGTTAATTGTAATAACTTCGG
TAATCCTCTGACTTTTGCTGTCATCCGTCAATTGCGTTGTACAAAGAAAAAACAAAGTACAAACGGACCAATTTCAGGTCC
AAAGTTTAAAGTCGTTTTGCAAAGATTATGGAAAAAGAAAATGCAACCTGATATAGATAGATAATACGGCCATGGGTTTAA
CGATGTAAGGAAAGACTTGCCAGGGTTGAATAAAGCTCACCATAAAAGGTTTAGTATGACCTGTGTAATATTGGGACTATG
TGACAAAATGCATCTGATTTCTAGGACAATGAGAGAATCTGAAATGTGGCAACATTTGAAAGAGGAAATATCACAGTTCCT
TATAAAGTTCCAACACACAAACCAATCATACCTCCATATACATAAAGGCAGGTTAGCAACACGAACAGAGGAAGTGATGAG
AGTCTACATTTGCATCATTTCCTGTTGAGAGAGTCCGTCCTTACCACATCTCCTGGCTGGTGTAATCCTACCCAAGGGAAA
TGGGCCAATCAGAAGCCCGAGTGGCCGTTATGAATGGGGAAGTGGACCATTAAAAGGGTAATAAGTCTCTGTGGAGTTTTC
TCAATGCCAAATTTATGTATCCTTGCCTCCATCTTTTCCTCGCTACTTAACTGCATCCATTTATGATGTGAGGGAGACAGG
CTGAGATAAGATGGGAGGAATCAAGGCAGCACTTATTTGCCAAATGGGACGTCATAACTCAGGGACTGCCTCGTTTTAATT
TAACATAAAATAGTTATAACTTTTTGCACAGCTGCATCACTTTTCACCGGGCTGAGGGTATGACTGTTCTGTTTTTAGGTC
CTGGTTTTACCATAACACTGCAGGACACTGTACCCGATGATATATCTCTAGATGCTGTGTCTCGTAGATCCACTCATATCA
TTAATCAATATACAGACTTAATAGCACCCTCGGTGGCTTTTTCATTGCTACATCTGGCTATGTCGAGTCAACCCGTGCCGT
GCAGATTTGCGGTTCCATCCGAGTTTAAGCATGCCACCTCTCGGACTGGCAACAAAGTGTGCTGCTCTGCCTATAAAAATG
GGCTTCACTGACGTGTCGTGACCACAGCCAAACCATAACGACCCCCCTTCTCACCCCAAACCAAGCGTATGTTTAGAGACT
CAACTCGTGTCTCTGCAGGAAACCTGAGAGACAGACTGTTTTGTTCTGACATTCTGATATCTGCTTTCTTTTACCATTTCA
TCATCTTCACTTTCAGCTGTTTCAGAAGTTGTGTGTCATTTTTTTCTGCATCATTATAAAAGCTTTTAAATAACTGAAAAA
AGGAGGATTTTAGTGTGAAGAATCTATAGGAAATAAAACGTTTTTATCACCAAATTAGAGGAGCTTTGTTAGCCGCTATTC
TTCGGTTGTACTGTAAGGAGGAAGATTGAGAAATGTGGAGATAAAATGACGTGCTGGACTGACATGCTGAGTAAAAACGAG
TCAAGCCAAGCTAGCACATGTGCATGGAAAATCCCCACCGGAAAACCCCAAAGTTTTCTCTTTTGTCTCACAGCATTCATT
AAGAGCCACAAAAGTTAAGTTCTGGAAGCCTACAGGCATTGAATCCAACATCCAAGTACTGTATATTATAATAATAAAAGC
AGAGAAATTCATTTTATAAGTTGCTCATTTCAGACCGTGTCACTGTAGCTAAATGTATAAAAGTTTCTAATACCTGGTTAA
TAGATTTCTCTGGTTCATGGGGTTCAATTGGTTCACTTGTTTGGTTGAATATATTTGTGCTTGTCTTTTTTGTTCGTGCTC
TTCTTACCGGAGGAAAACTGACACTCGAACGGTAACACATTCAAGTAACCAGTATAATTATTAATAGAATTTCAATAGTCT
ATACTTTTCAAGGTTTATGCTTTTTCTTTCCATCCTTTTCCTCTTTTTAGGGGCCCATCATGCTGTTGGAGGGCCCTCCCT
TGATCTTATCCTCCATATTCAAAAACAAAACCTGAACCCCATCTGCTCCAACTACTTCAATTTCAAAACTTTATGTAATTC
ATTCGCTATTTCAAGTGGTGAAAACTGCTCTTTAAAAAGCCTAATGGCACTAGATTGATACTAATTGTGGTACCATTTAAA
AGATAGCGGAGGCAATTTTACATCTCCATGATTAAAAAGCCTTCGATATTTCACAGACTAGGGTCACATTTTGAGGGCCTC
AGGAGACCCTGCAGGATTTCTTAATCACATAATAATGTATGCAAATAAATTAGGGCACAAAACAAGCCCTCGAACATCCAC
AAATAGTGCCCTTCATCATCACTAATTGAAGGTGCTTCAGCTTTAAACACCAAGGCTGCCACCTTTGCACCCTCAAACACT
TTGTACAACTGAAGTATCGTCTCCTCTGATTCAAAGCTCTCATGATAAACAATCTGCCTGTTGTGGATATATTAATATATT
CATATTTAGTCAAATCTGGCCTACAGCTCAAGGGATTCAGCAAGATGCTTTGTAGTAATACCAGTCACTCTCTTAGTGCTT
TTAGCACTGTGTGCAGAGGGGCTTACACTATGCGGGTAGGTGGGCGGAGACGCCTGAGGGCTCGTGGTTGATGCTGGCTGT
CCTGGTCCGTCTGGTAGAAAAACATCCTGAGAGCCTCTTCAAGCAAAAACCATGTTGGCAGACCGAGCAACCTCTGGTAGG
GAGGAGAGAGAGGGAGAATGAGGCAAGAGAGATGGGAAAAGGGTTGGTTAACAAGAAAGAGAGGAAAGAGAGATCAAAAGA

AAGATAATAAGGTAGTGCTTGAGGGGAAGATGGGTGGAGACGGAGGAGAGGGGGAAAAGGAGGGAAGTCAAAATGAATGGT
GGGAGAGGTCCGAAGAGAAAAAGGGGCTGTAGGGAGAGTGGGAGTTTGATGGAAGAGGAGAAGGAGAAGTAGGGTGGTGGG
ATATTTGAAGAGAGAAGGGTGCAAGAAGTGGATGAAAACAGAGGAGATGAAGCGAGAAAGATTACAAGTGCAAGGCAAGCG
AAGGCGAGAGAGTGTGGCAAAGTGAGAACCACGTAGGTTGCGGGAAAAAAGAGTAAAGCAGAAAAGAGAAGTGAGCGAGTG
GGAGAAAGAGAACCTGATCGTGTTTTTAGCGATGGAGCAACGCTGAAACATGTCAAAAGTCCCACAACTACAGTCAGTCAG
ACGTGATGTGGGCACAGGGCAGAGCAAAGAGAAGAGAGCAGCAACGCCTACATGTTGGGCTCCATCTGCAAACTACTGTTG
AAATATCCGAGTCAGTGCTTATCCACCTACAGCATTGTTCAGCCCACCAAGTTGTAGGCTTCTGCATTGCACGCTGCAGAA
GAAGCCGAGCATGCCAACAACGCAGCAACCTCTGCACAGAATAAAAGACAGCGCTGTGCATAAAACTAGACGTGATTACCT
TCATGTACGTGTTCAGTTCAGGGATTCTGCTCTCTGCAATCTGCCTCTTGTTGCCCATGAAGACTTTTCCTGCAGAGAAAC
ATGTTCACAATTAAAAAAGGTGCAGGAATACAATCAGCAGGGGTCAGATGTACAGTATAATCAAGGCCTATATAATTTCCC
ACACATACTGTAAACTGGTATTTATAGAAGCACAGCATGCAGAATGTGCACAGCTCACACATACTGTGCCTCAGACCAGGT
GAAGAGATGTGAGCTCAGAGTGCTATGGGGAAGTAGAAATGGAAAGATAAGGAGATCGACTCAATATAAGATGCAATTTTA
TTCTTTTAGGGTCAATTTCACTCGCTGAATATGATCGTTTACAGTCTACACACACGGAAGATTGACAGCTGGTGGTGGTGA
CGTGCAGAGAAGCGATGCAACGCCACAGGCAGGGAAGAAAAAATCTACTAGCAGGCGACACGAGAACGTGGAAACAATACA
CAATTAAAAAATAGCTTGAAAGAGCATGTGACATGTTTGTTAGCCTTCAAAGGTACTCCAATGACTCTATGTAAAGATGGA
CGACACACCTCCACTTCCTCCCACTGTATAAAAATGAAACCGAAATATCCCAGATACGGAGCTGCCATCGTGTACTGGTGA
CTTCATTGGGCAGTTATCAGGGGATGGAGCCACGGTTTCGAGGTCCTGCCCTTACACCCACACAACTCAATCGCAGGTTAA
CAATCAGGACGTTACGTCCCCTTTTTATAACACCAAACAACTGATTCAATCAAACTTATCATAAACATAACACTTCAACAT
GATGAGAAAGACTGTTAAAAAATAATTTGACTTGCAATTTGAGTTTTTAGTTTGTCCCACCTGCTAACACAACGGGGGCAG
GATTTATAACCTATACTGCATTAAGCCATGAGGGGGCGATTCTGCGATTCTGTCACTGGGGTAAAAAAAACAAAACACTGA
ATGTAAACATAACTTTCAATAAAACTTAGGACAACTTACATTAAATCATGGTGGGATAACGTGCATTATCAACTTTTTACT
ATTTTTACAGCAATTCTGTTCTTTATATGTATTCTACTTTAGTGTTTTTTCCTTGTTTCATCATTTCCTGTTTATGTCCCT
TTCAGTTTATGCTTTAGTGTGCTGTAGCAAAAAAAAAAAAAAAGACAACAAACAGAAACTAAGAAGCTTCTCTGGGTCCAAA
TAATGTTTGCCATAACATTTATCTGAGCATCTAATTATGAGTAATTCAATGCACCAATACACTTCAGATATTTTGATGCTT
TTTATACCCTTATTCTAAATCCGGATTGATTACTTGTTCAAAATTTGACAATGATGATACACTATCTCTGCACTAATAGTT
TGCAGGTACAACTGTGATGATTAATTGAATTACTGAAGCTACAAGTGGTTTAAATACTATATCAGCATGTTATGAACTTGT
TGACCTGGTGGAGCAGTGCGAGGAGGTAATTCTACGAACAGCTGATTAAGGGCATGTCTACCTCTTTGTAGTACCTACACA
GAGATTTGACCCCAAACCATTTGGAGACAGACATATTATAGACTGCTAATAATCCTCAGTTTCAGCCCCTTCAGTCCTAAA
CCCTTCTTGAGAGTGTGGACACATCCACAGACCAGTGCAGTCAAACGTTATGAAGCACAGTGCAGACTCCTAATGCTCTAA
TTCAGGCCCCTCAACAATCCATCCAAGGCCATCTAACACTGCTGTTAAATCAAATTTAGCACAGTCTTAATGACCCTCAGC
CACAATATTCAGCCCTCTGTCCTCTCTCAATTTCTCTCGTCTCATTCACTCTCGAGCACGATAACTCACTCAATCAGCCGC
AGACACAAATTCACTCATTTACTCACTAACTGATGAATCATTCACTCATCCGCTCATCTACTAACCATCTGCTGGGCCCTC
GGTGGATAAAATGCTCTCCCGACCTTGACTCTATTTTAGAGCAGCTTTCATTTCTTTATTCTATTTTAAGCTCACAGGTTG
AAGCACCAAAGGCTACACCTGTGATGAAACAAGAGGATAAGCGAGGAAAATTTGTTTGATTTTTTTAAATTAGCACGACAG
AAAGAAACTAAATGCATCAGCATGTCCATTTAAATCTCTTTAAATCATCAAAGTGAGAAGTGAGAAAGAAAGAGGGAACAA
AACAGTCTGTCGTCGAGATATTTTTCATTAATTTAAATTAATAAAACAGTTTCTATTTGAAAAAACAGACCTATCCGTTTC
CTTTTTTTTTTTGCTCCTACCCATCACCCATCACCCATCCTCCCCTCTGTCTCCCTCCTCTCTCCCTGCTGTCTTTCTTAT
TCCCTTTCCATCCTGATGAGATCTGGCACTGCAATTAAACCCATCAGATCTAATATTATCCAGCAGACTCCTCAGTGGCAG
CTGCTAAATCAGAGTTTGTAGAAAAGTAGAACTTTCCCAATATAGAAAGCGGCAATGAGAGAAACAGCCTTGGGTGTGTGT
TTAAATTATACATATTACCACATATGACTGAAATGAATACCTCTATAGTGGGTTACTTTGAAAAACACATTATAACAGATG
CACATTAAGCAGTGGTGCATAAAAAACATCTTTACTCCAAAGATCTCTGTGCTTTACCGTATTATCTTTTTCTGATGTTTA
ATTCCAGCATCTTTTAGTCGAATATTTACTGTAAATCAAAATACTTTTTTTTTTTAAATGTTTTTCAGCCCTGAGTAGTGT
CTGCAGGTCTTCCCCTGAGTCGCCCACATCCCAAACAACCCCTCGCTATATTTTAGGGCAGGCAAAGCTCTTGCTGATTGT
TTTGAGCGCACAGTAATAACTGAAGGCGAGTCAGATGAAGATATCAATGTTTTTTTTATCTCTCTAACCTGATATGGTGGC
TGGACTGTCACAGGACAATGATCAGAGAGATCATTACAACATTACAAACATTACAATAAGGGACAAATGACTCACCATCTT
AAAAACTCATTTTACACTTCATTCTGACCGTGAAATGCATCTCTACAACTGCTGAAACTCCCCGGCACACACACTATCTGG
TAAAAACCCTCACTCCTCTCCTCCTCTGAGCAGGCTGAAAGAAATGCCAAGAATAAGAAGCATTTGTAACTTTACCCCCTG
CTTGGCTCACAGCACATACGGCGAACATCAAGGGAAGGAGGCACTTGGGGGAGGACAGAGGGGAGCTGAGAAGAACAGTAA
AGAGATCAGAGAACAGCACAAGTCTTGTTTTAATGGTTCGGACTGGATTCGGTTCTGGCAGAACTCCAACCGCACAGGTGC
AGCATGAAGATACAACAGATGAAGTTACTTTGAAGATCTCACTCTAAAATACTATTACATCAGCGATACTGTATTATGGTT
CCTAATCAGTGGCACCGAGTCGGTAATGTTCGATGATGTCATGTCATGGGGAGACTCTGCTTCCTCCAAATGAGTTTTGGC
TGTTGTGGGTGGTTATCACTATAAACAGCCTGCGGGTGTATGAACGGGTTTCAGTGTTTCTGCTGGTAAGATTTTGCCACC
ACAAGCAAAATAAAATCATAAAAGGAAAACAAAACACTCCACGATCTATCATCTGATCCACTTCATGAGCGTTTGTGGTTT
GTTTGTGCTGCCAACACGTTTGTTACTTCAACCACAAAGCCCCAGTCTGTGATTACAGTGACTGCGTATGTTATCTTTCCA
GAACTGTAGTCGGGAGACCTCTGGTTTTACACTACTGTTATTAACTTCAGCCTGGCTCATAGCTCTGACATTAAACATTAA

ACCAAATCTAATGTTTCGTTAGTCGATAGCTACGCTAAAGCCACTCGAGTTCCAACAGAAATAAATCTGGATGTGACTTAA
GTGTGATTACAGAGCAGAGCAGTGTAACCTGACTCACCATCTATAAGTAATTTATACACTTTCGCACACACGCAAACACAC
AAACACCTCACTCTGTCCTCATCTCTCCTAAATCGATGGACCGAGCTTCCCATTCAAAGCCTTCAGGCTTTATTGGTGCAT
ATGTTGGTTTCCAGCCTCCGATAATCAATACAGACACTGACGGCGGCTCCCAGACAAGACTGACTTATGGGAAAACAATGA
GCTGCAGAATAAAACCGGAGCCACCGGGCAAGATGAGGACAAGAGGACGAACACAGACATACTGCAACACAGCGACTGATC
GAGGCCAGAATATATGTTGTGAAGTGAAAATCTTGTGTGTGCAGACTTCTATTTCATCTTGGATTGAAAGATTACAGAAGG
AAAGCATTAAAAACCCTAATTAATAATTCTCTGCATCAATGTCACAGAGACAAACTTGTATATTACTGTATTTAGCAGATT
AAATGAGTCTGGGGCTGGTTTCTGGTCTCAGATCTGCTGGCATCCGTCTGCTCAGCCTCACTTTGAGCTTTAAACAGCACT
CCTTAGTCTGGCCCTCGTTACTGCACTGCTGACTCGTTCTAATTTAAAGTTTCTGTTTGATTGTGGTCTGTCCAGGTGCTT
CCCACCCACACTTAATCTTCTTTAATAACAGCTCACTTGCATGGGCCAATGACTTTGCACTTTCCATTAACTTCGATTATT
AAACACAGTCTCTGTTGGGTGGATTTTTTTCTCTCTTTCATTCTCGTTCTTAATTCTCCTGACGCAACTCAACTCCTTGTC
ATCTTAAAACTTTATCCTACGAAGTGGATGAATGTATGAATGTCTCTCTTTCATCTCTTCCAAACCTCTTCCCCTTCGACG
TGGCTGTTTTCACTTACAGTTTGCATCCTGTATTTTCCCCGTGGCTCTCATCTGTCCGTCTTTCCATCTGCTCTTTTCTGA
CTATTCTGAATGTCACTGTGGATTACACTTATTTATTACAGCCCTCTGATATAAGGTTTAACATTGACCTTGTAAGGGTAT
ACACCTCGTGGCACTACGGTGACTGATGGCAGTGCAGAGTTTTTGACATTAAGCCTGACAACATGACACTTGGCTACAGGC
ATATCTAAAGGTTTTATTCCAAAACGATTTCAAGACCTTTAAGGGGAAGAAATTACCCTCAGTTCATTGAGCTCAATAAAA
TGACATTTGGAAAGATGATAACGATAACATTTAATCTTTATTACAGTCGTCCTGAGCAGATCCCGAGGATCATATCCAGCC
ATATCTTAATGGATCAGTGTCAGCCAAAATAAAGCCTGTAAAGCCTGCTCTCCTGTTTGACAGCCTACAGTGCGAGCATTT
CACTGCATATCCAAGTGAAAAAATAAGAGCCTGTGAATGAGAAAAACGGCACGACTGACTCTGAGTTTAGACATCGGTAAA
ATACACAGAGTCGCTAAACTCTGCTGCAAACATCTGTTATTATCTTGTCATTTTTAGCCAGCACTGACTGTCACTCTCACA
ATGACAGACATCACCAGTGACACATTATAAAAGCTGTCACTATTTCTAATGTAAATTTTGTGAGTAACGTGACATCAGACC
AAAATCAAACTTGTTTATCTGGTTTGTGTGCGCTCTCTAGACGATGGTATCTGTCCTCAGCCAAAGATTATTTGTGAGGGA
AGAAACTTCACTCTGTACCAACAAAATCTAAAAGATTTGCTCTTGGATTGTTTGAATTTTTGCAAACACTTTGATGGTTTC
GCTCTTGTTTTTGCTAAATCTCTACAGCTGTTATGTCAAAATTTCAGAGACATAAAAAAAGGTAAATTTAAGTATCGGGTC
AGACTCAGGATAAATAATCAAGATTAAAGGGCCTCTTTACTCCTTTTCTACTGCCTCACAAAGTAATTCAACTTGAACTAT
TTAAATTCTACTGAATAAATATTTTGAGTGTAGGAGTTAGACATCAGATATCTCCCTGAAGAGCTTTTTGTTCAGTATATC
CAAGTGGCTCGAACAGAAATTAATTCAGTAAGTTCACATGAAACTGAAAAATAAAACAAAACATTATTCGCTTTTCTTCCA
TGATTTCATCTGCAACTATTGTGTTTTTGAATTGATCGGGCAAAAATCCACTCATTTCTCAATCCACTCAGAACAATACAT
GAGGGGCGTATGTTTGATATATGATACCTGACAGATACGTAGCTCTGTCCGACACATGTAGCAGATCCTCGCTTTTAAAAC
CGTGTTCAGATGGGTTTCAGTTCTTCCAGCCACAGGGAGAGATATCAGCCAGCTGTCACCATAAACCCAGTCTGTTCCTCT
CCATATGCATGAAGTGGAGCAGAAATGAGCATGCACGTGAAGGTGAAGGTTACACGGTGACGAGGCATAAACGATGCTGTA
AAAAGTACAATGTTCCCCCTGTTTTCTGTCGGCAGCAAAGCCCCGACACAGCAAAATCACTGTCATCACTGTGAAACTGGA
GCCCTGCTGACTGCACTCTATTTTCCTCACCATTAGTATTAAAGTTTTCATGATAGTGAAGCATCTAAAGGACGATTTGTT
TGGTAACGCAACCTGAAACAGAACAAATACTAAGGAGTGAAATCAAATGGGATCACAATGAACAAAACTGTTGTACTTTCA
AGGTGTCATATGTGAATTTATAAATGATAGAACCTGCTTCATCTGTCACAAATAACACTGTGATTTTATTCAGGTTTTGAT
GACTGAGGATATGACCAGGGTCAGCACAGGAACGCCCATGAACTTATCTTAGGACATCTCGTTTATCTCATAGCAGCGTTC
AACGTTAAATAACTTGAGCGCTGGATTCGCATACATCATACATCATTTGTTCTACGGTGGCTTTTCAATCTCACTTCGTCA
TTAAAACTGTAAAGTTGTCCACCAGAATCGGTTGCTGTAGCTAACAGCACTAGCACTGGTGACTCACGAGGTGCCACTAGT
TTCTCATTCAGTCATTTCAAAGCTAACTGACACTTGTTAATAGTTAATGTAGGCTGCTGACAGTGAAAGTGCTGACAGGGA
CCAATAAAAAATACAACGTCTGGAGTTCACGTTACGTTTGGGGCTGCACAAAATGTTGCCGAGGTGCTGCCATTGGCCCGC
AGGGTCGCACTTTGAGAGACTATGATTAAGATGAAAGGCATTTGCCGTTGTGTTACAATAGTACTGGAGTACTTTAATGTA
TCATCAAGACAGTGACAGGTTCTAGTAGCTAATAATATAAAAAACTACTGTATTATTTACTACAACACTTCATATACAGTA
GCTTTTACTAAGACACAACTAGCTGTATGTGGATATTCTGCACTGTGGCGTATGTGCTGATCCCATCAGACATGAAAAAAT
GAACCTGAAAAATGAGGAAAACTCACTTTTATCAATTACTAAAACAATTAAGACAGAATGAGTGCAATAAAACACAAAAAC
ACTGTCTGTGTTATAGCAGGTAGCAAAACAAAGGCTTATCACACATGAAACACTGCACAATGGTGCAATTGGGCAGAAAGG
GTGTGCTTGCATGTGACTGATTGGCCAACACAGTGCATGGATGGATGACATCACAGTGTGTTGCTGCAGAATTTGAGCAAC
GTTCAACTCATTAGCAAAACACTTTCTTAGCTGCAGTTGTCTCACTGAAATTATTCACAGGTTGTGTGTTTTTGCATGCAG
TGCTTAAGTCTTCTCAGCTGAGGTTGTTTTCTCAGCACTAGAAGTTAGACTTGCAACGTGCGGACAGTACAAATCAGAACC
AGCTTCAGAAACATTTTTGTGTCCGGTATGGTAGCCCGAGGCAAATCATCTTGCCAGGGGTGCAACTTAACAAAATTGGAA
ATCAGAGACCTCGACTGTTCACATAGTTTGGCTCAGAGGTTCCCTCATACTGTACCACTCAGACTTGAAAAAGCATAAAAT
CAGATTTGAAGGGCGTCATGAATGACTTGTATTTGAACTATGTGAAACCTGTTGCGTGTCGACGACTATGACGTGACATGT
CTGCATGCCGCTGCTGCAGTTATCAGTGCTGTGATCTCACTCACCTGGAAGAGCAGGCAGGACGCAGGTGTTGGGACCAAG
TTTCTCGGGGTTCTCAGGAGAGTATTTGGACTCGAGGATCTGGTGGAGGTTGAAAAATTCTCTGTACCTCCTGTAGATGAG
ATACTTACTCCCCCCTTTAGTCCTCACCTCAACCACAAACATCTGGGAGAGAAACGCAAAACTTGAGGCGGATTTGTACTT
TTTGTTGTCAAAGCTCTTCGGCTTAGCTGATGTTTTTAAACTTACAAAGTAGTCGATGAAGCCTTTCTTCTCCTCGATATC

TGCGATGGTGGCGCTGACAGCGATGTTGTGAGGCAGCTGATCAAAGTCGCTAAGTAAAAGAAATAATGTGGTGATAATATT
TTATTCATTTAAAAGGAAAAGTTCAACTCTATCATTATCTACTCACGCCCGTGCAGATGGAAAGTCGGGTGAAGTTTCATA
CTCCAAAAAACATTTCTGGAGCTTCACAGCAAAACAGTGTTGCAGCATTCCCCTACACAAGTAAAGTAGATGAGGACTCAG
AACGTTAAAAAAGAAAAAAACATAAAATGGCTCCACACAGGCTTGTCCGGCATAATCCAAGTTTCTAAGAAACCTTGAGAT
CCCGAATTGATTTGAAAAGACAGACAGGGCATGTGCTAACGCTTAGCTTAGCAACTTTCATCTTGAGCAAAGGTTGTAAAT
TATGTCTTTTCACATTAATTTAGGGTGCCGGGGCCTCCCAGAGACTTTGATTTCACCGGCTGAGCTGTATGGAGCCATTTT
ATTCTTTTTTTTTTTTTTTTTTGCTGTTGTTGTTTTACGCTTTAAAACAAGTCCCCATTTACTTCAGCTGTTCAGGAGAGT
GCTGCAACAATGTTTTCCTGTGAAGCTCCAGAAAAAGTTTTGCAGACTACTAAACTTCACCTGACTTTCCGTCGGCATAAC
GACGAGTAGATAACGACTTTATTTTCATTTTTGTGTGAACCGTTCCTTTAAGCCCCATGTTCCTGTTTTTATTGACTTTTA
TTTACATATTGTTTACACTGAAATTGGTTGTCAGCTGTGCAGTCTTTTTTTCAAAAGTTACAAAATCTACTGTTTGTATTT
TAATGCATGTGCTCCATTTATGGCAGCAGCAGCAGCAATAACACAATTAAGCAATGCAATAAACAACAAATGTATTTTTTC
CATCGTTTTTTAACAATGAAGACCAGAAAACCACCCAACTGTGCAGCCGCTGAACTTCTGCAAACACTGCGCAATGGCAGG
AAAGTCCGCCTGTAATCTCATTACCCTGACATACTGAACATAACAGGATAACAGAAAAAAGTGAAGTGACTAACCTGATAT
CATGTTGTGATATAACCACTAAGTTAACAGACACTAAAACACAGCTGCAATCTGCGCTATAAATGAGCACCAGCCTGCAGA
CTCATTAACCCATTAATGAAGTTAGTGGTTAGTCATTTTTATTAAAAATGTGAAGTGAAGACAAACTCACCTCTCGTCTCG
TAACTGCTGCGGCAGCGACATGACGGTGGTCACAGTGTTCAGCTGCAGACGACCGGAGGAACTTGACCTGTCTGTGCGGAG
AGAAAAGCGCCTCTCACAGAAGTGTTCGTGGGAGAAAACACTCACTGACACACCGCAGGCTGCACACACTGACTCACCGCC
TCTGAAACTGTCTGTGCGGAAACAGCAACAGGAAGCGAAAGTTCCCCAAAAAAGACCGAGCGAAGGTGCGTTCAATTGACC
GTGCCGGGCGGGGCGGGGTCTCAGCGGGGTGCAGGCAGGTGGCACATGAGAGGGCAGGTATGATGTTCAGTGAGCAGCAGG
TAGAGCAACATATCTGGAAACAGTTCTGGATGGAGATGAGCTGAAGTTAGGTGTACATGAAACAGGTGGGATTACATTCTA
ATGGGTCAGTTCATCATCATCTGAAATATCTGCTGCCACACAAAAACAAAGTGGAATACTGAAAAATTAATAAAAACTTAA
AAACAACTTAGATTTCCTTGAAATGATTTTATTATCTATTATGGTCGAGTACAACCTATGCAGCCATCATCACACATAATC
ACCAATCAATCAGGACAGCATCCTAATTGGCAGCCTGTAGTTGTCAACCATGCATGATCTGGCACAGGCAGCATGTTTCAT
TTTGTGTGCCGGAGGCTGTTTAAAGAGAATAAAAATCAAGATGCCAGAGGTGGAAAGCTCCCAGCTCTACTGGGCGTGTCC
ACATAACAGCATAACAGCAACCTGCATAACAGGTGAAGTCGGCCCTCCTGTCACCTATGATCAAATTTATTTGTGTTTGTG
CATGCACATGTGAGGGAGGGCCCACATTTTACCTGCAGGAGCTCACAGTCTCACCGCTGGGTGTATGGGTACTTTTGGTAA
CACTAAAAATGCTTCTTCACCTTCAGAAAGTGGTGCAAAATCACCTGCGAGTTAAAAACATAATCAAACATTCATCAAATC
TCTAATTTCTATAATCAGAATAATGATATTAATTAACGTTGTTGGACTTTGGAAAAAAACGGACATTGTGATACTTCTGGT
TTTGTGTGACACATATTGTGAGTTTTTACCAGATAACTTGAAAAGCTCTATTTGGAAAGAATTAATCAATCCAGAACGACA
GAATGATTTTGTGGGGGAGTGCATTTTATCAGACAACCAGAGCAGGCCCTGCTGTGTTTTGTAAACTGATCGAGAATGATA
GTGATTGTTGGTTTGCTGTGTATGCAGAGCCCGCATGTCACTCTCAAGCAACTCCCATGTATCCAAAAACCCTTAAGCTGG
ACTAAATTAGACAAACTTCTCCTGCACAGTAGATTAGTGATGAAAATGGGAACCGTACTAGTTTTGCGTTTGTATTAAGCA
GCAAAAATGTTGTAGCGTGATATGTTTGAGCTGATACACCTACTTGCATATGCGTGCATTGCATTGTCGATGCTGAAACGA
TATATTACGGAGCCCTAATAATAAAGCTAAAAGTAATGGATATCAATAAGTGTTTTAGTTTCTTATTGACAGTTATTTTGC
ACCCAGTTTTGTATGTTTATTGTTATTTTTTTCTCTCTTGTTGTAGAGGACACTGTACCAAAAAAAATCCCTGTAAATAAGT
AACTGCTCCAGAGTCTGTGCACACATGCACCATCAATCCCAAATCACTCCCACATTCAATTTGACTGATTAGTGTAAATAC
TGTGAACAAGATTGTTGACTACTGGCAGCCTCTCCCAGCCTCCCACACTGTATCACACAATGGAGGATTGTTAGTTTCTGT
CACGTAAACAAAAAGCACAACCTTGCTCCAGTTTAAGCGCTCTCTTGTCAACATGGTATTAGCTTGCACAGTCAACATGCG
CGCTGGCTAAAGACAAATACAAGCTGTTATCATGATCTACTGCAAGTTTAATATGAATCAACTGGGATGATGGACGTGCTA
AAACAGCCAAACTAATCTAACGAGACAAAAAAAGTCTGATTTAAAGAGACTGAGCTGAAAGAAATATCTAAAATGGAGACA
AAAGTTACACTGGCTGTTGCACCAGCTATGTGTTAGTCCAAACATAACATGAGTTTGAATGAAGTCTGTAGCACGATACAA
GTTAGTTGTAGCAACGTAAAGTTTAAGTAAAAGTTTACACCTCCGCCAAGCAGGTGTAAGTCATTTCTTATCTAAAGGTGG
GAGAGACGAATGAGTGATCCTCATCCTTTTACATTAATGACGACACTCAGTGCTGTACTTGGGTTAAACAGCAAAAGTGTG
ATGTCATGCTGCATTTACTTGAGTGAATGCAGCATGACATGCATTTTGTCTCCTCAAACTCTCTGATTCATTTACCTGTCT
TGAGGAATGTGTGAATCATTCAGCACCACTCTCAACATCTCTTCTCTCTGTTGGAAAGACACTTCGGGACCTTGAAACTCT
CTAAACTCTTTGCTGTTCCAGTCCTGTAACTGCAGGTCATGTGTGAGTTCAGTCTTGAAGAGAGCTGCTAAATGTTGGTCA
TCATCCGAGACCTGAAGCACACAAGAGAGATGGCAGAGTCCAATGAGCAGGACATGTATTTTTAACTCGGTCTTTTCACAT
CCACATCTCAGTTACATTGAGTACCTGCAGCCTCGAGGAGCAAATCTGAACTGCAAAAAGAGTTCAGATTTTTTGGTGGCA
GCAGAACAAACTGTAAACACACCACTGACACATCTGGAGGTAGAGCAGGTTGTCCACTAATGGCAGGGTTTGGCCGTTCGA
TCCCACCTCGACACTGCTCCTAATGCCAAAGTGTCCTTGATGGTGAGGCCGGCACCTTGCAAGGTAACACTGCGATTTGTG
TGAGACTGTGTGCAAGTGTGAATGGAAAGAAATTACAAAAGAGCTACAACGATGCAGCCATTAACAATTTAGTCTGAGTCA
TGTTCCAAGCAACCTAACAATTTTAAGTCCAATATTCACTTCTCCATTTTGGCTTCCACCGACACCTGAGGAAAACACCTG
TCAAAACACACTAGCCGCTAATGACGTCTGTGGTGTTTGGTGCTTTTCAGGGAATGTACAAATGGTTTATCAGAGCTTTTT
TTGTTATAAAATCCTGCCCGATGCATCATCAATCAATCATTCGTCAATAAAGTAAACTCACAAGCCATAACACCAAAACCA
AGAACCAAGGGGAACAGGAGAGTCTGTAATAATCCTCTGTGGGTTCGTCAGCGAGAGCGGCACCTGTCATATAACACATAA

TCATTGACCCCTTGTTAATATATAAATATTGATTAGAGCAGCTTTAAATAAAACTGACACCAAACTTAATTTTCAGTTGTT
TTACTCAAACTTAAAACTACTAAAATGCTTTTATTTTGTCTCGTCAGTTTGTCCAACATAGCTGAGATAAGCATGGCAGCT
TTATGTCTCCGCTATTCTTTAATTCAAGTGAAGGTTTAAATGCATATTAGAAAAAGCAGGTATAATATAAATTGAATGTAA
ACATGTGTTAACATAACAAAAAAGTGTTATCAAGGATAACTATACTGCTGAAATCAAAATGTTCATGTGCATTATCCTTCA
AACCGTGTTCAGGAAATAATCTGTCCCCTCCACCTGTTTAGTCAACTCTTTTCTTGCAGTGTGCTCCGTCCTTCCTTCCCT
CACTTCTTATCTCACCTGAAGTATAAAGAAACACACGCCCTTACACAAACATTAAGGGCCACAGAGTTGACCTCTCAAACT
GCTTTTGAAGTTGACAATTAAAGGTACATACAGGTGAGGGCTGGTGACTGGTGACTCTGAGGGGGGAAAAAATCTGGAACA
AAATCTGGGACACAGTTCTGACATCAAATCTGAGGTGATTAAAACGTATAACACTAGATGTCAGCAAAGTTGTTGCTATGC
TGGAGGTGTGGGTGTCTGTGTGTAAAAGAGATAACACTGATTTATGGTCTACAGTTTCCTGCGGGCTCAAGATCAAATGTA
GGTGACTGATTGAGTTAAAGTTTGTAGTCAGTCTTAATCCAGCTGATCTCTCTTTGAGATGTCAGTTCTACTTCAGTCTCT
CTGAACACTTTCATACTGTCAGTTAACATTAATGCACACAATATCTAGACTTGCTTATAGTTGGGATTCCCACCAAAAGAC
GGAAAATCCACAACCACTTTAATCCCTAGCGCTGATGTTTGCTTTGTAACTACAAGAGCAGAACCACTGCATCACTGACTA
AAGACACAGACTGACAAAGATCAGAGGGGAAAGGAATCACAGCAGGTACAAGCTTGAGTAAATCAAACAAGGTGTGCTGAA
GGCGTTCTCCCTGTGTATTCAGTGTGATTCTTCCCTGTGACTTCTTGTGTGCACTGGGATAAAAGCGTTTACAAAAATGTT
TTTCAGATGTTACTTGGTAACCGAGTAAAAAACTTAGTCAAGTACAAGCAAAGAGACATAGAAAGAGAGAATGTAGGAGTG
TGTATGAGGAGAAACTGTGTAGGTGTGAATATTTGTTTCTGTTTGTTACACAAAGTCTCAGTTACAAAAAAGAGTGCAATA
AAGTGTGAGGCTGCATAGAGGATTGTGTGTGTGTGTGTGTGTGTGTGTGTGTGTGTGTGTGTGTGTGTGTGTGTGTGTGTG
CAGCACATATTGACTCCATATCTATTTTAATTCAGCGTGAGATGGATCCTTTGAAACGCTGCTCGTATAACAGACTATCGC
CAGCCATGAATTGCAGAAACACTTTTCAAGGGTGTATTTATCGTCAAGGTCAGGCCAGCTGCTAGAGTCTGTAGTGCGTGT
CTGCGTGTGTGTGTGTGTGTGTGTGTTTAAGAGAGAGAATGTGTTGATTGGGAATGTGTATATGTGTGAGAGAGGCTTTAT
GTGTGTGTTTTTGTGTACTGTGGCTGTGTTTGTACATAGTTTACTGCATGCTAGCCTTGGAATGAAACACAAGTCCACTTT
AAAACACCTGCTGATGAAAGATAATGAGCCATTAAGCTGACAGACAAACAGGTACCAAAACACACACACACGCTTATAAAC
ACATACACACAGAAACAGAAGTGAGATTGACAGAAAGTCAGCTGGTTGCCATAGAGACGTCATAAGGGAAGTAGAAAGAGG
AAGAGGTGAACCTGAGCACCGAGTTAATCGAAGCACTGAGTGACATACTGTGACTATAATCAGTGTGAGACAGCTCAAGAG
TTTTTTTGTCCAAATTTCGATATCCAGTGCTTTAATTTATGGCAAAAACTACTTGTTTTAGATGTACTGAAGAGATCACAC
AATTCTTAGATGCATCAGGATTCTCTCTTGGAAGATTATGTCTTGATGCAGTCAGTAATCTTATATTAATAAGCTTACGGC
TGAACCACGTCGTGTTTACGTTCCGAGGCTACTTCTTGAGTTTTGGTGAGGATCGCTGAGCATGAGCAGAGATAATAAAAG
TAAATTCATCCGTGTATTTTAATTATGAATCAAAATAAAAGTTCCAAGTTGTCACACAGTGAGAAAACATACATCACGTTT
AGTTGCATCGAGCCATCCGAATCGAAATTGAATTGCGAGGTGCCTTGAGAGTCCCACAGCAACATAAAACATACACATTTG
ACATCTGACAACACTTTTGAGCAAGAAATATCTACTGCCTTTGGAGGAGACGTGGCAAGAAGGAGAAGAAGAGGAAGAGGG
TATCTCTGGGGAAAGCCTGTTATTATGTGAAGTATTTGCTAAGCGTGCATGTACATTTTCAGATGAGACAGACATTCATGT
GGTTGGCAGTGAATGTCAAATGTGAAAAGTCAAAATGAAAAGTAGTGATGAATCAAGGTAATAACCTCTAACAAAAATAGT
TTACCTCACGATTGAAAGGGTTTTATCACTTATATCACCTGATGAGACCTCCTCTCTCATCAGGACAGCGTCATCTCAATC
ATTCCTACTGGTGATGAAGTGTGTTTGGTGAAATCACATAACATTTGAGGGTTTGCAGGGCCTTTAACATTTATGGTAGCA
ATATAATTTACTGTAGAGGTACTTACCATGGAGTAATTCGGCTGTAACTGTAAATGATATTAAGCGTTTATTTCCAGAGAT
CGGGGTTTAGTTTAAATACATCATGTTTTAATGTTCCCACTGCAGAGATAATGCTCGTCAAACAATCAGTGCGACTAACTG
GAATCTACCAGGGAGATGTGGCCGGTTCAGCTTCTCATTATTTAACATTTTGGCTGCGTTACAAGCCAAACTGCCAACAAC
TTGGTGCATTCCTTTTAAAAACTCACCATCTCCCACACACGCCCACACACACCAGCCGTAACATTAGCACTAAATATACCA
ATATAACCAGTGCCTGCAGTGCAGTCATGCACATTTGGCAAAGAGCGGGGCTTCCTTCTCCCACTCTACTGTACATATTGG
CCTGAAGTAAACTCATGAGGGTGTGTGGGGTACTGTAGTTAAGTCACCAACGCAAATGAGTACAAACTGAGGAATTCAAAC
TCCTTAATGTCTAAAACTGAGTCCACATCTGAGCAGCTCTGAACAAGTTACTCGGACATCTTTAAATACAATCCAAATGTA
GTTGCATTTTTTTGTTACTCAATGCAGCGCTGCAAAGGTTCCTTTAAACAACTCCAAGGGCATCGACATGTCATAACTTCT
GGCTGCTGGTCCCATCCTCACAGTAAAGCCACACAGTTCACACACCCTCTGACTCTGAACAAAATCAGGTGATTACCGATT
GTAGTCTTGCCAGCAGCAAATGTTAAGCCAGCTAAAGTCACACAACAGTTACCTACCTTGAGTTGCATTTGTTTTTATTTG
ACTGACTGCCTCCCAACCATAGACTGTATAATAAATAGTGGATGGAGCAACTATGACATCACCCATTGTTTTGTGGACTAA
CATGAAGCCTCAGGTTTAGCATTATTGCTATTTCTTCTTGGGTTTTTTGGAGCTAGATGTGACCATATTTGAACAAGAGGG
TGCAGTTGGGGAGGACATCCGGTTTGGATCTGACGAAGAAACCGCCTTGGCAGCAACTCGTCAGTAACAAGGTGCAAGCAT
ACCCTGGGTTGTCATCTGTTTTACTCTAAATGGGACCACGATTTACAAAATAAACAATATGCTGTTTTGAAAACACTTGAA
ACTATGGACTGAAAGCATAAATCCATTAGGAAACTGTTAACTGAGGTCATAAATCAAGTGAGAAGTAGGGCCTTTTTCTCA
TAAGCGTTGTAGTGAAAAGCTGTGAACCCCCTATTGGTCACCCCGTGTTTATTTTTAGGGCCTATATCTGCTAATCTTCGG
GTGAGTTGACAAGGGACAACCCAGTGAAATCGGTACTGCACACACACACACACACACACACAGACACACACATAAACATAT
TCACACACACAGCTGTGGCTTGTGTCCCCCCCTGCCATGACTTAATGCACCACCTGTTGTTGAACTGCAGGTTCTGTGACA
CGCTCTGTTTTTCAAATATCTATCTATGCATCTATTGTATGTAGGCATCCTGTCTGTCCATCTTTGCTTTGCAGAGTTGGA
CAGACAGGTCCAGGCAGGTTATTAATGGCTGCTTCAACTTTAAACACACAGACAACAAAAGACTTCTGATCCTCTGCCCCC
TCTGCTCTACACTCTGTTCACTCTCCTCCTCTCTCCAGTGTTAATAACATTGTTACTCTTTCATTATCCCCATTATTTCTC

AGCTTGTCTTGTCCTTGACAAACTCCCAACCTGCCTCAACCTCAATTATTGCTATTTTCCTTTCTGGACCCTCCCGGTGTG
TTTTCCTCACCACCTCACCCTGCCCTCCATCGTCCTTCTACAATGCTGGACAGTGCTGATGAGGCGTAATTCGATTGAGAG
GAGAAAGCTGGAGAGAGCAAGACACTGAGAAAGCAGAGGGTCGACACAGGACATAAAGAGACAGAGACAGTATTTGTCTCG
CATAACGCAGCTGAACAGCATTATTCAAAGATTGTGTGACAGAGACATGATCTCATATTGTTTATGTATAGGAGTATCGAG
CATAGAGTCTTCCTCTAAAGGAAAAGATTATGTCTTTTTGGATTTTGGATTATTAGCCAACACAAACCATTAATATTATCA
GAAAATACTCCTCACAACAAAGTGAGTCTACTGTCTCAGGACCTTTTGTTTGACTGGCTGCAGCCTTGTACCAGCGAACGT
GTCTCTGTCGTTCTATCTCTCATTCTTGCACCTACCATTAATTAATTCCTCTCTTGGCCTTGACTAGCATAAGGGATAAAA
ACAAAAGATAAGCGGATGACAAAAAGAAAATAGAAGGAGACGGGAGGAGACAAGGAGTCACACAAGCTGTCAGGGCCCTGA
CACGATAGAGATCGATTCGCGATAAAAGGAAGAGGCATGAGATGATACGGATGCATAGAAAGAGGAGCAAATTTGTTCTTT
GATATGGAGAGAGGAAAGTGGAAGGGGTGGAGACAGGAAGGAGCGATAAGGAACGTAATGGACAGAGCTCGAGCTGAAAGG
AGAGATACTGCAAGAGAGAAAGTGAGGAGTAGAGTTTTTTTAGGGTAAAAGAGAGAGGGATGAATACATACAACTTTAGTA
AGGTGTTCCCTAAGTGGGAATCAGAAACACAGAATCAGAAAGAAGTCTGATTATTTTTAAGAAAGGAGTGTAGAAATGTGT
GTTAAAATTTAGGTTTGTGTGAGTTAAAAGGTGGTCATGTTAAAGCTGCATCAGTTTGTTTGACCACTCGGGGGCAGGACT
CCACCACCAGCTAAAAATCTCTCAACAGTTGCCCGTTTACAGATCCAGCGACACATAGAAACATTATATTTTATTCATTTT
TGTCTCCTTTTCTTCCTGCTTTGGTGTCCGCCAACTACTGAGGGAAATATCTGGCTCTCAAGCTGTTAAGTGCTCCACTAG
TTAGTCACAAACTTTGTCTGCTCATATGTGCTGAGCAGGTAGTGCACAGATATACAGTTTATCAGAGACACGGGCTGTATA
ACATGAAGACGATGGAATAAATAAAGGCAGATAAGAGTATAAAAAGGATCGAAACAAAAGAGGAAGAGGGTCAACATGGAG
GAGATGTGGACTGAGGGCAGCCCGATGAAGTGATATTATTAATAGGCCTGTGTGTGATTTGTTATTGCTGCAGAGAAAGCG
ATCGCACTCCTGGGCTGACGATGTTTGTGTCATATCTTGACAGCCACTCTACCTTTCTCAAAATAGAGAGAATACTGAGAG
GTGGGGATGTGGGGTAAGAGAGTCTGTGTTTGGAGGCAGCTATAAAAGTCTTATTGTGTCCTCCCTCCCAACTTCATCTTA
CCTCCATCTCCAACCCTGCTCTCTACTACATTCTCTTTCCATCACCTTTCCTCTTGCCCTCTGTCTCAGCTCAGGTGCACT
CATCCACCCCTTCATTCAGGTAAGACAGACAAGAAACATGACATGTTGTAGCAGAGAATAGTTTTGGTCAGTGAGGCTATC
GTGAACAGGAAAATCTAACTCAGTAATTGCTGGGTTTATCTCGAACAAGCAGATAGTCAGCAATTGAGTGAAATACCTTTG
CTAGTTGCATGAATAATTAAGCCGCACGCAACATGATGATGAAATATTTTGTAGCTGTGGAGAGTACAGCAGGAGTGCTTT
TGGATAAAACAGAGTCAGAGCTCTGCTAGAAACTGAGCTGACATCACAGATGTGAGTTTAACCAGGGTTATAAAAGAGCAT
GGTTTTAATTAAATGGAATATGGGGGAGTAATTTCAGTATATAACAAAGAAAATCAGCAAAAACAGGACAGAAGAGTGCCA
TGTTTATTGTGACAACTTCCAGTCAGTGTGAGAGTTTTAAGTGTGAGTTAAGTATTTGTCTGACACCAGTTTCCACCTCCC
TGTGAGGCTGTCCAGCACAACTATACAGGTGGTGGGCTCAGATGAAGGCAGGTTTTTTTTATAGTCTTCAACATTTGTGAT
TTTAGGCTGTTGTGTGTAAATCAGACAGACTAGAAAAGTTGCATAACAATGCGTCAAAACAGAGTAAATCTACAGAGTCGA
CTATCCAAGAAAACTCAGCATAAAACCCGCAATAACAATGATGAACCAAGTAAAAGTCCTTATCGTTAGCATCCACAACTT
CAACACCATGTCAGAAGAACAATGCCATATTAACATCTACTGTAGAAAGCACCACTGCATGTCAGTCAGCTGTTTTCATTT
TTTAATTAGCATTACCATTTAAAAAAGTGGTGGGGACATTTCCTTAGCGCCCCAGTGTAAACTGGCACCTGTACATTTTTG
CTTTTTGACAACACCTGGCTCACGTCTCTATTATAAAGCAACTTTTAGTCCCTTAAAAAACAAGGTTCTCTTTAAGGGACA
CTTACTCTATTCTGGTCTGATGTTGATCACTTCTAACGTCCCCTGTGTGTTGCAAACTGCTGTTTCCTTTAATTGTTTTCG
TCTTTGCAGTACTGTGTATCAAATTAAAGGTAATGTTGGAGCAAGCGTTGCTCTAATGGTTGTTTTCTTGGCTTGTGTCTC
ACCATAAACAGGTGTTTATGCACAGAGACACGGCACAGTAGCCGAGAAGTTGAGTGCTGTAATTAATAGTAATGCATGTTT
TAGATGTTCTTCATGGTTGTTACTATTTTAGCTGTGCCCATGTTACAAAGGGGGGCCCAGATGCTCAATGAGACTGCCTGT
TTTAATAAATTATACTTTATAAATAATACACTTGCTGTGCCTCGACTCACTGCTGACTGTGTTGTCACCAGAATTTCAATC
TTTAACTCGCAGAGATCTTTTGTGCTGTAGGCTGATGCAGTTTTCTGGAGTTCTCAAGCAATGCTGGCCGGATCTTTCAAT
CATTTGCATGAAATTCTTATTATTTCTGCCTCTAGAAGTAGAACTGAAGGCTTAACATGTTATTTCTTTTGCATTATTTAT
GACAAACTCAGAGCCAAGAACTGACATTCAATCTGCAGTGAAAGTCTGTCATTTGATGAATAGATGGTGCTAAAATTTGAG
CTCAATAATCCATCACAGCGGTCATATTTGTTCACAGCAGGGCTGATAGCAGTCATTGTGTCTGTTTTGCAATTTTTCACA
TTTTAAGCTACCCTGAGGAGAAAGGGATGAGCTCAAGGACACTTTGGGCTCGTTCGCTGCAAACGGTGACAGAAGCTTCGG
CCTTTTGATTACAAGACAGATTCTTTCACCACAGCTCTCGTCTGCACACCTCAATGTCAAGGCTGTTGTAAAAGAACCTGC
AAACTTAAGTCAAACATTTGGTATGACTGAGACGCATATGATCTGATGTCGGATGACATTGGCAGGACATGAAGATCCAGA
ACAACTTGTCATACCAGAACTATCTGTCAAAGGCTTGTTGAGGATGGATAAAAGATGAAATCAGGTATCCAAACGACCTTT
CAGACATCAACAAACCACTAGATGATAACCTGAGATATCTTTAACAGTGAGGATAACTAAACTACTCTGCTGAGGCTCTTT
AAGTCTTATATCTTGAGATAGGGACTAGGCTGCTGAAGACACAGATCTCTTATAGACACGAACACATTCATCAAACATCTG
TCTTGACAGCTCAGCGTGTGTTCCCAGTGCTCCAAACAGGCAAACAAGGAGCCTGAGAGCCTTCAGCTCAGTTCTGACACC
TATTGTCATCTACGAGGTACTGCAGTGCAACTGAGGCACACAATCCCATCCACCTTGGGGGAAACATGACATGACAGATAC
ACTGCCATCTGTTTCAGCAAATATCAGCTTTATATTTATACTCTATGCCCAGTTTTCCTCTGTGAACACTGAAAAACAAAT
TTGTTTATTCATTCAACCTTATTTAAACCCCGATTTAGAATAGAGCCGAGTATAAATAGAGAAAGATCCTTGACAGCAAG
AAACACAAACAGGAGGCTTAGATAAAAATGGCTATAAAATAAGCCCAAACTAACAAAAAGCCCAAATTAGTAAAACAGTAA
ACAAAGCAATTAAATTTTAAACAAACAACAGCTGGAAGTGAAATAACATGAGATTAAAAATGGCAAAATGGCCAAAAACTG
TATTTTCTTGCTTGTAAATTGGAAGCAAAGGCCAGTAACAGGCGAGCTAGTGGTGCAATCCTCGAGTCAGATGTGCAAGTG

CTGGCATCTTTAACTGCGTGTCCATTGTGTGATGCAACGTGGCAAAAAGAAATGTGATTACAAATAGATATTGTTTTAGAG
GAATAGAGTTGACATTAGTAAAGAAATGTATGATATGTGAGCTTGTGTGAGTCAAGTATGCGTGTTAGAGGTGAACGGTTG
GATAATACGGTTGTGTTTGTATGTGTGCAATTGGGGTATTATGTATTTGTCCCCTTAGTGTCCGCCAGACTGTATTCATGC
CAACGTCTTAAATACTTAACTATGGCCTTGAAACATGGGTCTTGTTGTGCCAAGTTCCACTGTGGCCATCTTTTGAATGTC
TAAATATAATTTTACCAAGCCCACTAGAGTGTGCATGTTTGTGTATATGCCATCTCCAACTAACAGACTATATTAATTTCC
AATGAACCAGTCTTTAAGTATCTCTAGGTCAGATCTGTAGTTAATGGCCATCAAGTTCTCAGATCAGATCCACACTTAACA
GTTTAAATTTGGCTTTTAGCTTTTGTACCAAACCCTCCACTGTTGTACTTCTGCTCTGGCTGTTGTCTGCTTTGAACAGCT
TGAAATACTTTTCTTTCATTTCTAACTTTGCACCTTTCATGTTCTCTGTTTCTCCCTCTCTGGCCCTCTCTCCAGCTGACA
AAATGTCGATGACAGATCTGTTGAAAGCTGAGGAGATCAAGAAAGCTCTTGATGCCTTTGCAGGTCCGTAACAGTCGCCCA
GTCCATGTGGTATTCTCAGCAGAAACAGACATACAAAGTTCCAGAAGTTAGTTTAGACACTGACAGATACAGTAAATGCA
CACACATTTTTTAATTGTATATTGCTAACTGTCTGTGCTATTAATTTAAATCCACAGTTCTGTGATAGAATTCATTTTATC
CTTTGAGGTTGATTTTCATTTTCAGAAGATTTCTGGCTAACATTAAGGTGAACATTAGCGTTGATTGATTGGTTCTGTCTT
GCCTTTTGTAGCAGAAACATTCGACCCCAAAAAGTTCTTTGAGATGGTGGGAATGAAGGCCATGACAGCTGAAAACGTCAA
GAAGGTCTTCAAGGTTCTGGATGTGGACGGTAGCGGCTTCATAGAGGAGGAAGAGCTCAAGTGAGGAACACACACAGACAC
AAAAACACACTAAAAAAAGGAGGCCAGGCCTGGATAACAGACTGCATACTGTAGGAAGCCCATGTCATGTGTTCATTAACT
CATTAACACTGTCCTACAGTGATGCTTCTCTGCAACACCCTGTGCTTTTCTCAGGTATGTACTGAAGGGCTTTTCCCAGGA
GGGCAGAGATCTGACTGACGATGAGACAAGAGCATTCCTCAAAGCCGCAGACAAAGATGGAGATGGCAAGATCGGCATTGA
CGGTAAGAGAACGGAGAAACATAATGAAGTTTATGATAAAGAAAAAAGAAAAGTAGGGGTGATGGAAGGTCTAGTCGGCAC
CAGAAAGTTTGTATTTTTTTATGAAGAATGTATAAATCTGTTTTTATCACTTTTTGACTCTTTTGCCCCAAGCCATCTGTT
TGTACTTTACGTATAAGTCTTTAAAAACAGATATCTAAATATCTGGTTTCCTTTTTTAAAACCCGGCTAAAGCATTTCTTT
AACAGCTGGGAGTGAATAAATACATATCTGGCTGTTTAGATAGCAGCAGGGTGTGAATGCCGTATTGACTGAAAATAAAAT
GCTCACTGATGTGTTTTTAAAGTCCAGTGTGTTGGATTTAGAGGGACCTTTTGGCAAATAAGGAATATAATATTCATAATT
ATGTTTTCATTGGATGTGGTGACAACATATAGAGAATATAATATAAATTAAAGCTGCAAGCAGCGATGAACGGGCCCTCGC
AGTCCACGCGCGTCGGGGCGTGCTGCTGTCGTTACATGCTGCTGTCCGGGCATGCTGCTGTCGAGGCTTGTTCATGCAACA
ACTTCCGTAGAGGAAACAAAACTGAAGGCAGTGAAGCTGCTGTTTCGTCATTTAGCAATAAAAGCGCCACCTATTGGTCGA
TTTGCACAAAATTTTGTAGAAATGCTCATCTTGCCATGGAACACAACTGGGAAAAGTTTTGTGTTCATCGGACTGTATTTC
ATCAAGATACACAAGAATGTCTGTTTGACGACAGTGTGCAGGAAATTGTTTTATATTTTGGGCGTTCTGTGGACTAGCACA
TTTCTTTTAATAACTTTTTGTCAGGAGGGTCCACAGATGCTGCGTGCAAAGTTTGGTGCAAATTGGACAAATTGCCTGGGA
GGAGTTCGAAAAAGTAGGTTTGCGACATTTGGCGAATTTGCGAATGGAAATTCAGTGCGAACGTGGGCGTGGCCTACATCA
TACAATTCAGGTGAATTCCGGGAACACGTAGATATAAGGTTTAACAATGTCCGACAAATTATGTGGGAGTAATTAGCAAAA
AACGTTTTTTCTTGATAATAGCGCCACCTGCTGGTCATTTTTGGTGTGTGAGTTACAGGGGCCGGTCTATACCATCCCTAT
GAATTTCATATCCATAAGTGTTATGGTGTGGGCACAGTGGCAAATCTAAAATGAAACTGCCACCAGAGCGCCACCTAGTGT
CATATCGGTAACCCCTTTGTCACCTGTCCTCAGGAGGCCATTGGCAATGAGTGTACCAAGTTTTGTGTTAATCCGACCAAC
CGATGTCGAGATATTAAATACTTCAATTTAAAGAGCGCCACCTAGTGGTCATCGGCCAAAATTTAGCACAGAGCCTTAGAG
CATCATGAGGAAGTAGGATCCTGAGTTTGACGTCATTTGGACAAACCAATGTGGAGATATGCAACACTTCCTGTTTGGAAC
GAAATCTGCAGGAAGTTGTTATTTAATAACTTGAGTATTTTTTGGAATATCAAAATTCTTTTAATAACTTTTTGTCAGGAG
GGTCCACAGATGATGTGTGCAAAGGTTGGTGCAAATTGGTGAAATTGCCTGGGAGGAGTTCGAAAAAGTAGGTTTTTCGAC
ATTTCGCGAGCTAGCATAAAAACGGTAGGCGGAAATGGGCGTGGCCTATATCAGGAGATTCAGCACAATTCACTGAACGCG
TGGATACAACGTTTTTGAATGTGCGACAAAGTATGTGGGAGTTATTAGCCAAAACGCACTTTCCTTATTACAGCGCCACCT
AGCGGTGAAAATCCACAACGACAACAGATTATAAAATTTTTCGCCAGGTTTGATCTATATTCCAAGTTTGGTGACTTTTGG
GGTATGTTCAGGCAGTGAAAAATGCGATCATTTTGGCCGAAAAATAATAATAATTAAAGCTGCAAGCAGCGATGAACGGGC
CCTCGCAGTCCACGCGCGTCGGGGCGTGCTGCTGTCGGGGCGTGCTGCTGTCGTGACATGCTGCTGTCGGGGCATGCTGCT
GTCGTTACATGCTGCTGTCGGGGCTTGCTAATGCATCAACTTTTCTTGAGGAATCACAAGTGAAGGCACTCAAGCTGTTAT
TTAGTCATTTAGCAATAAAAGCGCCCCCTATTGGTTGATTTGCACCAAATTTGTCAGAACTCGTCATGTGGCCATGGAACG
CAACTGGGAAAAGTTTTGTGTTAATCGGACTGTATTTCATCAAGATACACAAGAATGTCTGTTTGACCACAATGTGCAGGA
AATTGTTGTTTTATATTTTGGGCGTTGTGTGGACTAGCACATTTCTTTTAATAACTTTTTGTCAGTAGGGTCCACAGATGC
TGCGTGCAAAGTTTGGTGTAAATCGGAGAAATTGCCTGGGAGGAGTTCGAAAAAGTAGGTTTGCAACATTTCGCGAATTTG
CGAATGGAAATTCAGTGCGAACGTGGGCGTGGCCCACATCACACAATTCAGCTGAATTCAGGGAAGAGGTAGATATAAGGT
TTAACAATGTGTGACAAATTATGTGGGAGTTATTAGCCAAGAACTGCTTTATGTTGATAATAGCGCCACCTGCTGGTCATT
TTTGGTGTGTGAGTTACAGGGGCCAGTCTATACCACGCCTATGAATTTCATGTCCATAAATGTTATGGTGTGGGCACAGTG
CCAAATATAAAATGAAACTGCCACCAGAGCGCCACCTAGTGTCAGATCGGTAACCCCTTTGTCAGCTGTCCTCAGGAGGGC
AGTGGCAATGAGTGTACCAAGTTTGGCGTCAATCCGACCAACCGATGTGGAGATATAAAATACTTCAATTTAAATAGCGCC
ACCTAGTGGTCATGGGCGAAGATTTTGCACAGAGCCTGAGGGGCTCATGGGGAAGTAGTATCCTGAGTTTCACGTCATTTG
GACACACCAATGTGGAGATATGCAACACTTCCTGTTGTGACCCGAATCCACAGGAAGTTGTTATTTAATAACTTGAGTATT
TTTTGGAATATCAAAATTCTTTTAATAACTTTTTGTCAGGAGGGTACACAGATGCTGTGTGCAAAGTTTGGTGCAAATTGG

TGAAATTGCCTGGGAGGAGTTCGAAAAAGTAGGTTTGCGACATTTTGCGAGCTAGCGAAAAAAAACGTTAGGCGGAAATGG
GTGTGGCCTATATCAAAAGATTCAAGACAATTCACTGAACGTGTGGATATGAGGTTTTTGAATGTGGGACAAAGTATGTGG
GAGTTATTAGCCAAAAAGCACTTTCCTTGATTGTAGCGCCACCTAGTGGTGAAAATTCACAACGACAACAGATTATAAAAT
TTTTCGCCAGGTGTGACATATATTCCAAGTTTGGTGAGTTTTGGGGTATGTTCAGGCAGTGAAAAATGCGTTCATTTGGGA
CAAAGAAAAAAAAAAAAAAATAATAATCACTACAAAAACAATAGGGACCTCGCAGGTTTCCTGCTCGGGCCCTAATAATAAT
AATCATTCGAAAAACAATAGGGTCCTCGCAGGTTCCCTGCTCGGGCCCTAATCATTGCCAGCCTCGTCTTTAAGGTGTATG
TCAGTAGAAGTCACCTGTGCAAATGTTTTCATTCTTTTTTTTCAGAGTTCGAGGTTTTGGTGCATGAGTAGGGGCCAGCTG
AACAATCCCCTTCTTCTTCCTCCTCCTCTTCATCCCTGCATTCATATTTTGTAACTGCAACCTTCAAACTCCTCTCACCTT
CCTGGGGACCCCACACATGGAGTCAGACGAGGCCTGCTCGCTACAAAACACTGCAGAAGCAGGCCAACAGGGAGTCAGCGT
GAGGAGGAACATACAGGCATCAGTTTCATTTTTAATTTTTTCATTTTTTATATATATTTAATCGCACATGCTGTATGGGTT
TCAGAATCATTCTAAATGATTTTAATGAAGATAGTTTGTGGTCTGAAAACAGGTGGTACTAGTGCTGTAGATGGTGTAGAT
GTTATGAAGACTTCTTAAATCTTTATTTCCTTCTTTTGTGTATTTCAGGTTGTGCATTTTGTCTGATTGATTTTACTTCTA
GATGTTCACATATCAGTGCACTTTCCTCCCACTCCCCTTCCTCATCCACCTTCCCTCACCTCACCTCATCTCCTCTATCTC
CTCCTCCTCACTCCCTGCCTCTGATCAGATGATGTAGTAGATAAAATGATATATAAGACGAGCTGTGAAGAGAGAAAAGAA
ATGATAATAAAGAAAACTTGAGTAATAACTGTCTCTTTACTTATTTTACTCACAACGGACACAAAATATTAAACTCACAAA
ACAACAACGCACTAAACATTTCAAAACATGTCAATCACAGGAGGGTGAAGAGGATTACAAATTTACAAACACTAGTTGAGA
ACCTGTGGCTTTCAAGGAAAACTATCACTGTCATGTGAGAGATCCTATATTCCTCATGAACCAAAGACTCTAATTTGGGAC
ACATTAGCTAATAAAATAATCATAAGCTATCAACAGCTTAAAGGAATAGTCTGAAATTTTGAAAAATATGGTTGTCCATTT
CGCTTAGTAGAAAGTGTGTATGCCAAATTTGTTGTTGGATTTAGCAGCCAGGAATCTTGCTGTTTTTAAAGAAAATGTGCT
GGTAGGTGGATTTTGTTACCGCTTGGCTGACACAGTCTAACTGTTTACCCCTGTTTCCAGTCTTTATGCTAAGCTAACCCG
CTGCAGATAGATATAAGAGTGCTATTGCTCTTCTTATCCAACTCTTGGCAAGAAAGCAAATGAGTATATTTCCAAAAATGT
ATAACTAATCCTTCAATATCTCTTCTATAAGAAACAAACAGGCCTATCTATAAAAACTCCAATACTGTACAAAGAGCTGAT
GAAATTGGTTTTGATATTAAATAATCTAGATGTACTACAGTCTCACTTTTGATTTTGTTACAAGAAATACTTCACCTAATC
TAACACTGTACAAAAAGCCCACCAAACCTTCCACTGTCCCTCCAGCTAAAAGCCATCACATTTCAGAGCTGTGATTACATT
CTCCTGAAACAAAAATGTATATCCCGCCTCCACAACCTCATCTGGCAGATCTGGGTCACAAACCCTGCCTGCATGACAAGG
ATGGATCAGAGGATCCGAGGAGAGGATCTGTCATTGGTCGTCGTGGGGCGAAGCTTTACGGTGGCGAATGGGTTTGTGCCT
CTGAAACGAGAGGATGGCGAAAAATGTAACACAATACCTGACATGCTCAACCTCTGTCAACACAAATGAACAGATACACAC
AGGTGCACACACTCCAGGATGGGAAACTAACAGCAGACCCCAGCTAGAAGAAGGTAAATTTTGACTGTGGATAGTGAGCAT
GCTTCAGAGGTTTGACTTTGGATTTTTGGTTTAAAAATTTGGGTTGGGAATTAGCAATCAACCTAGGTTTGTCTAGATAGA
TGATCCTCAAAGGAACAGTTCACCCCCAAAACCAAAAATACGTATTATTCCTCTTATCTGCAGTCCTGTTTATCCCTCTAG
ATTCCCTTCTATGCAGTGATACGGTTGCTGGGTGAAGTTCAATAGAAAGAAATACTGTAGCTCCAATGTGAAACTACTAAC
AAAAGGCCTGTGGATTATCTTGAGTAAGCGGGACATGATTTCTGGAGCACCACGAGCAATTAGCCAGGCAGACATCTCTAC
AGCCGATATCTCAAAAAATCAGCAACTCACACCAAAAATACACAAATACACATAAACAGAATTTATGATTTTGGGTGTGTC
CCTTTAGAAATATGTCAAAAATGTACTGGAATTCATCAAGGAATATAAAATTATTTTCTATCAACCCATACACACTAAGAA
AATTACCTGGGAAACAGTGGATTTTCAGGTGGCCCATGGGGTGATAATGCCTAAGAAAAGAAGATATGCCAGTGTGAACAA
CCAAAACAAGTGAAGTTGGATTACATTATATCGAACAGATCCAGAAAATTATTTCCAGAATTAAACCTCTGGTACCTGGAC
ATCAGATGCTGATTCACCCTTCCTGTCAGGGAGGGGAGAGATTGGTCGAAAATCTACAGAGCCCCTTCTAAGATTCTGATT
TGAGGGAGGAGGAGGCGGTGGGGGAGGAGGTGGAGGCAGGTGTTCATTGTAGCTCTTTGTCTGACCGGGCTTTGACTCCAC
GACCGGCTCCAGCTTCTTCTCAGGGAGAGGAGAGACCGGCCGGAAGTCTACAGAGGCTCTGCGGTTGGGTGTGGCTGGTGG
TGGGACATCTCCATAGCTCTTGTTCTCTGATGGGTCGGTGTCAGTGGGGTCCAGCAGATTGTTCATACTGTGGCTTCTTAG
GGAACCACCACTGGGAGGGGTCACATATAGGATCTTTAAGTGATTCATACTAAGTTACCTGGTACATTTTGTGAATAAATA
GAAAATAAAGTCCTCCTAGAAACAGCAGTAAATATGCATTTTAGGCGTTAATACAATCATTCTGTGCAACAAAAGAGTGCT
CAAAAAATGTAATACATTTGGTCTCTTGCTTGAATAAAAATTTAGCATGTCTTATGCTGACCAATATATAGAAGTAGGTCT
ACTTTTTCAGGTGAGGAGCTCTTTAACTCCTGGGGTCAACCTGTCCCTACCTTGTTGCTAAAGTGTTGGAGAAATCCTCGA
TTGGGGCCACATACGCAGCAGGGAACCAGCCCTGACTGAAAGAGATAAGACAGATACATATAAGAAAACATTACATGTTCT
CATGACAAACGGCAATATTGTTAACATTTCCACAACATCTTTTTTCACATTTCTAATACAATAATAACATTTCTATAAACT
TTTTCTCATCTCCGTTGTGGTTTCACTGTCAACTGTACAAGGAGGAGGGAGAGGGCTACAAGGCAACAGTTATGCCAGAGA
TTTATCAGTTGCATTTCCTTCTGGCTGCTGACAGTCATAAAAAGCATTTAGATCGATTAATTATCAAGTAAAAATGCTTAA
AAACAAATCCTCCTCCTATCCCAGCCACCCACCGCAGGCTGCTGTCAGTGCGTCCATACAGCCAGCCGTTGCGTGGCTCCT
GGACCAGTACGGTGACAGTCTCTCCCCTGTTGAAAGGCAGAAGCTTTGGGTTGGATGAGGAGGGGTGAGACACCAGGGCTC
TCATGGCTCTCCCTCCACCCAGACCCAGAGATTCCCCCACGGAGCTGGAGCGGGAACGGCTGGGGATGGGAGACGGTGCTG
GAGGGGGGAGAACAGACAGCACAAAGCATATGTTAGTTTACACAATGCAAATGTACTGAGCAGTATCTATTTTTATGGAAT
GTTGCTGAGGAAAATACTGCTGATGTTGTTTGGCTTCACTGGTTATCAGATCGTAATCACATTCAAGTACAGTCATCCTCT
CACCTCTAGAGGGCACTCTGCCCAGCGCCTGCTGCTCCCTCCTGGCCCAGGACATATCTTCATCTCTGGCTACAGTCTGCA
GCAGGGAGCTCACTGAACCTCGCAGCTGATCAGGAACAACACACGCAGGTAAACTGACTGCTGTTCCCGCTCAAATTCTAT

TTATTTGTCATTGTATCTAAGCTTAAGTTCTGTGCAGCTGATAGACCATATACAAACGTGTGAAAAGTATATCTAATGTCA
TGCAAACATCTCTCAATACGGTGCAATTCAACACCACTACAACATCAATAATAAATATATGGGTAAATTAATACCTGTCTA
ACAGTGTCAGTGAAAACTAAGCATTATTATCTTAGTAAAATAAAGAATTTATGTCTGAGCTGTAGCATTTGATTACATCCA
ATATCCATCAGGGTTCTTCCTACTAAAAGTTAATAAAGCAGTATACTGATAAAACAAGGGCTCAAGGCCCCTATTAGATAA
TTTATGGTAGTTTCAGCACAACACAAGATGTGCTGATCGACTGAGTATAATCTTCATGTGGATCAAGATAATTGACGAAAC
GACTTGATGCGTCTGTACCTGCGCCTCTTGGTCCACATGAGTGGGAGTACGAGGTCTGGACCCTCTGGTGTCGTTCACTTT
CTCCTTCCATCCATCTGCCTTTTGCTGAAGAGATGCACCCGTCTGAGAACAGAAGGAGACACCAGCGCCTCATTAAATCTC
AAGCTGATCAGGCAGCAACACAAATGAACAGTGAATAAGACAGTGGACGCTGAGACATGAATTTCATAAAGCAACCAACTT
GCTACCATGTTGATAATAAAATGTCTTGGCCATGGTGGCTCAAAGAAAACACGTAAATGTTGCTGAGACGATGATGATGAC
ATGGGTTAATTTGGCATCAGCTACACACACCTTCACCTGGGTTTTTTTAACTTCATTTATGCAGTGGTGTTTCACTGAGA
GCACCGCTCCCTTTTTTATGAATGCCTTGATCACATTCACACAATTACACACATTCATACCTGGAAGCTGCTCAGCAACCA
CGATCTGATCTGCTGTCTCTGTCTGCTGAGCAGTGACAAGGGCCTTTCTCAAGGGCACAACAGAGGTGGTAATGAGGGAGG
GGTAAGTGCTGCCTTTCAATTTCCCCACCCAGATTTATCATTAAAGTCTGACACTCTCATCACTTTTTATAATTCTATTTG
TCCATCAGCTCTAATTGACTCAATAATACCTTGCAACACCTAAATCTGAGTAATTTCATTTTCCTCTGATGTATACAACAA
GGAATGTCCATCAACCAGTAGGTAGACGTTTTTAGTGGACCGTCATGTAGATTTTTCTCATTAGTTAGCAATTTTGTGGAA
TTGTTTGCTTACTTACTACTGAGCCAACTTCATAAAAACAAAAAGCTGGGGTCAAGCCCAAAGTTAGCTCACGCACGTGTT
TATACCTTGTTTATAAGGAAGAGCAGTGACTGAGTGAGGCCACAGTGCTTCTCTGCTAAGAAGCGATACCTCCTCTCCTCC
TCCTTCAGAATCTCCTGCTGACTTTGCCTCAGGTACAACATGTACTCACTGTTCTCCTGAAAACACAAACACATATGAGAA
ATGACACATGGTACAGATGTGTTACTGCTGGACTGTGTGAGTGCACAGATGTACATTGTGTACAGTATATCTCAGTGTGTG
TGAACCAGTGCAGTGTTTATGTGTTTGTGTAACTACCAGAGAGTCTCTGTAGGCTCCTCGTCTGAGCTGTTTCTCCAAAGA
TTCTGCCTGGTTTCTCACCTCCAGCTCGTACACTCTGCGACTCCCCTGTGGCACAGGAAACCGTTTGATTTAAGAAGCTCT
GATGTGATTATCAAATGTTTTCCTGAATTTCTTCACAAACAGACATTTTCTACAAAGATCTAAACAGTTCACTCTCAACTG
AATCTGGCCTTACAGTGTAGTTCTCTTGTCTTATATTGGAGGGCATGTAAAAATAGTTTTTGTCATACTACTGTAATGAAG
TAAAATTTCAGGAGAGATCATTCAGAAAACTGCACACATCAGTAGCTTGAACCACAAAGCATGAAATAACAGGAAAGTCCT
GAAACTCAAATAGTAACTGCACTAAACATTTATTTATTTAAAAATATACTGATAAATATTTTACTTTTCTTAACTTTATTG
TCATAATCATTAAATGCAGAACATCCACGGAGTATATTTAATATAACAGCAGCCTAATCATTACAGTGAAACATTTTCTGA
ATCTAAATAGCTCAGTAAGCTCTTCAAAATTCTCCTCAACTCCACTCGTCCTCAGAAGGGTACAAATGAAAAAATATATAC
TTTTCCCTTTCGTGAACTCACATCAATGTACTCCTCATCCAACTTGACATTCTTCTCCATGGCTTGCAACACCTCTATCTG
GAACCATCGAAACTGTAGCAGGAAGAAAAACACGTGTCCCTGCGGTGTAGCCAAGTCATTATGGAAACCTTCCCTTTGTTC
AAACACAATTTTTCATTATCAGGTTGTTTCGCTTGACTGCATCTATATGTAAATGTGCCCCGTCCTCAGACCAACTATTTT
ATCAGAGGCCACCTTTTTCTTATTCACATCACTGAAAGCAACTGCAATCAGGCCCAAGGGTTTGAGTAGCACCATGAATTT
TTCAACAAGCAGCAAAATGGAAGCAAATGTCTCCCAAAAGCTTTCTGCAGAGCTCAGCTTTAGTCATTTAATTTGTACTGA
GTCAAAAGATCAGACTCCTTCACAGATTACTTAATTAAGGACTCAAAAGTCTATTTCCAAATATGAAATATTGAATAACCT
GCTTTTTTCTATGGCTGTGCAATTTAGCATACTCAGGACAACAATGTCTATGATGTGTCAATGCTATCCTGGTTTCCTGGT
GACGTTACTTGGGGAAATGTGTACCGTCAGGCAGCACTTTCAAATCTAAGAAGCGTTACAAATAACTCTAAGAAAGAGGAT
AAGCATCCTGTTACCATGGAAAACGCTGGTTACAATTAAGCAACAGAATGAGACGGGAGTCGCGGCAATAATTTGCGCAAA
GTATCGGAGCTTGGAATAAGTTGGATAAGATTATGTTAAAAGTCAACTGGGTTTAATGAAATAGCAAGACACACCTGTCGC
ATGGGACATAATTTATTGTGGTTCATATTTGTTCAATATTTGTCTATTTTAAGATTTAACCAGCACTTCTGATCCTGTGAT
TTTGATTAAATAACATGAGTATTGACTGAATACAACTACTGACCACTCCTTCCATCTCTGCGGTGAGTCTCCTCTGTGTTT
CTGATATCTGGATCAGGACATCCCCTGTTGAAAACAAAAGGACTTGGGTAGATCAACACACATATCAAATATCTGCAGTCA
GAATTTAAAACATTGAAAAAAGTCTACGATGCTTTGAACTATTTTTAAATGTTACGTAGTATTATAGTGTCATATTGATTG
TTATATCATGTATTCAATTAAATTCAATGTATATTGTACGCACCTGCAACAAAACGTTTTTCATTAAATGTTTAATTCTAT
CATTTTGTGTGATCTTACGTCCATTACAAAAGAGAGGCACAATATTTTATCAGGAATGTCAGTAATTTGATATATGAATTT
AACACATTTCAACACAACTCAAGTCATCTACGTTTCGATCAAGCCAGCTGGACTGGTGGAAAGTTAGCCAAGCCCTTTATA
TTATCAGTGCCTCTGGACTTGTCTATTGTCATGGATTGTGGTTGGGCTAATGAATTGAGTGCAGCTGAAAGACTGTCCCCA
TCTATAATTTAGAGGCTGTCACTCAATAGGCTCCCTGGTCACGCTGTGGCTCCCTGACCTTCTCAATAAGCCAAACTAGCA
GAGCCACGTGCGTATTTGTGTGCTAGCACTGATGTGTGAGCCCACACACTCTCATTAGGGATGCGTTAGCATAATGCTAAT
TACCGTTAACAATGCTATCACACGTCCCCAGGGTCAGAGTGAAAGTGAAGCTTGTGGAAAATATGCACTCATGCATGCACT
CAACAGTCTGCGCACATGCACACACAAATACACAACCACGAGCACACACAGAGATATTTATAAGAGAGGACGAGGTGTAAT
GTAAGGGTGTAATCATAAGGTTACTGAGTGAGCGTGTGGTATTTTACAATCTGAAATGCAACAGTATGACCAGGCTTGACA
TTAACTCGATAGAGCTCTTGGCCATCTCTAGAGGACAATTCTGTTGAATTAATGTTAGAAATTATGGGCGAAACCTGTAAT
AGAAAGACTCAGTAATCAGTCGCACATACAACCAGGCACACACACTTACCAAGAGAGCGGGATGAGAGCGTGTGAATGGCC
TGGTCACCCATCTTAGCCACAGCGCTGAAGTAGGCCTCACTGCAAACAGCTAAGGCTGTATGGCACACACACACAAAAATA
CACAATAAACTAAAAGCTTCTCAAATCTTATAGACTGATAAGCTTTGGAGTAGCATAACAGATAGTTTAACTACTCCTAAT
TGGGTCAATCATTTCTGCCTCTATTCATTATAAACACAAGCCTCGCAGTGCTGACATGTGATATGTGATTTTGGTCGAGGA

ATAGCCCCATGCCAGTGGGTATAGTTTTACCACGCTGCCTTGTTGGCCTGTGACTGACGGCCTGTTCTTGTTTTATCAGGC
AGAGAGGTGCATTAATGATACATGCTGTTCACCCAATTACCAACGTAACACACGATAAAGATTGGGGATACGTAGGCAGGA
AGGACTAGTGAGGCAAAGACTGGGAGATACGAAAAAAAATTCTGTTCAAGCTTTGATGCCTCATGTCTGAAGAAAACATGA
GGCATCAGTACTGAAATATAAACTTAATTTTACATATTTTATTTGTACATGAAGAGGAATATAGTTAATTTTGTCTTACTA
CTCAACATGTCACTTTACTGTGCGACTCTAATGCCCAATACAAGCCCAGTCACTGGACTAGCTGGTGCAGTTAAAAACGGC
TTAAAACCCAGATAAAAACTTTCAGTGCAACAAGTAAGAACTGCAACAGATTTACACATATTGTGACAGCAGGTTTGTGAT
CTTCCTCTACTTCTGCAACTAAAACAACACAATCCTTACAGCACCTACAGTATTAGGCCCTCTAATTAACATTTGATTGTA
TATTACAGCGTGTGGTTTTTATTTCTAGCTCCTCTCACAGGAGATTACACGACACCATTCACATATTTATATGTGTAAACT
GACAATCAAAATAACAATGACAAAATCTACCCACCAGTCAGTCACATGGTCACTCACCTTGGAAAGCTTTGACATAGCTGT
TTCCTAAAGCGACGAGCTTCTGCAGGCCAGGATTGAAGTGCTCCATCAGGTTCTACAGTAACACACACACACACACACACA
CACACACACACACACACACACACACACACACAAACACACAGACACACAAATAAAGTGAAATACATGCAAACCAACACAC
CACTGAAAACAGTTGAACAACAGTTCCTTCAGTTTGAAATACTACATTACAGCCAATGTGTCCACACCACGACCAGAATCC
AAATGTAGGGAACAAGAAGACAATATAAACTCACCACATAGACAGATAAAGTTGATCTGTGCAGCTGGTCACTGGTGGCTC
CGGACATAGTGACCAATTTCAAACAGAATATGAACCTCAAAAAACAAGAAAGCCTCAAAACAAAGATCGTCAGAGACAGAA
CAAACTTCCGTAAAGAGTCTGTATTCCCATTCAGACTTCTGAAGACGTTTCCTCGGGCGTTTCTCGATCTTTGGATATCAG
CGTCTTCAGGCAGTGAAAGTCACTTCACTGCTGTCCTTCTGGTCCTGCTGTTGTCCCAAAGACCGATAACACCTGTCTGAT
AGAATGATAGTGCTTTCCTCCCTCTCTCCCTTCTTCTGTCATTCGTTAGTGAGCTCTTTTACCTGCTTGTCATCAACAGTT
TTCGTGGAAAGAACGAGCAAGCCAGCGAGGGGAAGAGAAAAGGGAGTAGGTGCCATCGATATGACCGGGCCAGGTAAATTA
TTGATTGAAACTCTGGAAGGAAAGGAGAAGTAGGACAAGCAGCTGCCTAACTAGCTGTGACGTAGACTCACACCTACGGGG
GGGACCTCAGCTTTGGGCAAACAAACTACTGCCTTAGAAAATTTACATATTATTTACTCATATACAGTTAGATCCAAATAC
ATTAATCCAGAACCTGAGAGAAACACTTGCTTTTGAACGTTTCAAACATGATGAGAACATTTCCCCAAGTCAGTATACGTC
TGCTTCATAGGACTCATCATCATTAAAGAATCAGAGGGTGCAATTCAAAATGTATTACGTGCTTCTGATGTCTTTTACTTT
ATACTGCAGTTGAACCACAATGCACACACTCTACTAAAAAATATTTTTCATTATTTCTCGTGTAGCAGAAATCCTAACAGA
TAATCATCATTTAAGTAATAAAATTCTAACTAATATTGCCAGTATCAGATATCTATTAAGTTCTAATTATTATAAAAATAA
TAAAGTTTATCTGTAAAACTTATTTTATTGTCAACAAACATCATTTTTCAATTTGCTTTTAACAGTCAGTTTATGTTTATG
TTTCAGTGTGATGACAATAGCCAAGTTATTTAAATATATCAAAGGAACAAAGTGCAAAAGGTATTTCAAATATGGTGAAAA
TTATTTGGAAATACAATCAGGAAAATTTCATTGGAAAGAATCTACTATATAGCATCTGACATTATCTTTAAGTTACCTTTG
AAATAGCTTGATTCAGATCCAGACAGTCAGTGTTAGTTTTAAAAAATATTATTGTCACGGGACGACAAAAAAAAGCAACAA
AACTAAAATGTAATTGTTTGTTACAAGACAAAATAATCTGCATCTCACATGTAGTCACAAATCCCCTCCCTCACTACACAC
AGACACTTAACAGACAGCCTTTCCAGTAAATGACAAAATCTGCCTCTTCTGTATTTCAATATTGTATCTATCCTTGATAAT
AATTATAATATATACAATACATGACTTCCTGGGAAATGGTGCAAGTGTCTTAAATCCAACAATCCGTTCCATTGTTTCTGA
CTATTTTCCTAGGACACTGTTACATTACAGTGAATTTATGTTGTCGAATCCAAAATTTCTGGAAGTTTAGTGTAGCTATTT
AGGGTTCCCTTCTCATGTTTTGTTCTGTTTATACAACCTGTTTTACTGTAACTGGAACTATTCCAAGACACTGTCAAAGTC
CCTAAGACTTTAACAACAACATTAATGTAACAACATCTTGCATGTGCAAGTGTGACATGTAGTCATCAACATAACAAAATT
AATAGACTTTCCTACCCAGCAGGTGTGACCGTGTCTTTCAAGGAGGCCATGTCATCATAGATGACATCATCATCACCACCG
TCTTCACTCTGTCTCTTTTGGCACCGACTTACAGTCTCCAGCACAGTTTTCAAGCGCCGGTCGAGAGCGGCGAGGTGGGGC
TCTGACAGCAGTGGAGCCACACTGTGAAGGGGATCATGGGAGAGCGAGGCCCTCATGACGTCACTGAGACGATACTGTGGT
AGAGAAAGCAGACGCAGGCGTAGCCAGGTGGTGCGACGGATTCTAGAGCCAGATAGAGGAGGAGTTGGAGAGAAAGAAGGA
TAACACGGCGTAGTTAGGGAGGAGGAACTAATGGAAGAGAGAGGAAAATGGTGGTGATGACTTTCATGTGATACCAAGGTC
ATATTGTGTGTGATTTTTTTCCTAGTCTCACAAAGCACCCATTATTATTCTGGTATGGGTGAAAAAACACTTGGGCTTGTT
TGTATTTCTTTAAACCAATTCAGTCTGAATTTAACTGAAGACAACAAAGTCGACACAAAGAAAATAATGAACCATATATGA
TCTTGTTCCATGTACTTGTATTTACCTGCAGCACTGTTCTAGAGGAGCTAGGATAGAAGGCTCATCTTTGGAGTGACGCCC
AAATCTGCAATCGGAAGAGACCCGGAGTCAGTTAAGCCACATCACAATTACAACGCCAGTGACCACTATAAAGGAGTACAT
TAACAGAGACACACACAAACACACCAAAAATACTCACGCCCTCCCGTTGTCCAGGTGTAGTAGGAAAGTGTTGTTGCCAAA
CCTCTCAAAGGTTTCATAATGGTGCCGGTCCATGTTACCTCAAGGAGGAGAAAAAGGAATTGAATTACTATTATCAACAAA
ATTCACACCTCCTCTGTAGAGCTCTAAAGTGCTATGTATTCTGTTGTTACCAGCTATGCAAACCTAAAACTTGTTTGTGTG
CTAAAACTAAATACAGATATCCTCAAATAAACAGTTTTTTTATGTTTTGCTTCATAAATCTGGACAAAAAGCTACTTACAC
ACATAATTCAAACAATGATACGCTGAACAAAATTGATGGCATTTTGTGTTTTCACACGTGGGTGAAAAATATCTGAATATA
CATTAACATTTAAACAGCTTTCAAAACTGAAAACCAGCTGATATGGAGGGGTATTACAGTCAGTACATCATGGCATTACTT
TCACATGACATCGGGTGTAGTTATGGGTGCACAAGACCACACAAAACAGTGATGCCAGGTGGATCATACAAAATGCTAGAA
ATTGCAATTATGACATTGTGTGAAATCTTTAATAAGAAATTCAAAATATTGCAAAACTCAAAGGATGAAGAGGCATTTTAC
AGCAACAGCAGTGTCACATACATTTATGTGAACAGTAGTCCCACTGACAAATATATTTACAAAAATAATCATGTAGAGTGA
GGCCTTTTTTTATCAATAAAGTAAAAGCATCTGAGCTTCAGTCTGGTACATCACAGCAGGTAAAAATGTGGTTTCATCAGA
GCACACTCACTAGCCTCACCCTTCTCAAACTACACAAAACAGGATTTAACTTGGCACGAAGGGGCAGCATAGTGATAATGT
TAACATCTGCCAGTCCCCAGTTTCCACTCTCCCGCTTTCCATCGAGATAGAGCGGGCAGTGATAGAGTGGTAACTGTGGGT

CACAGTTTAAATGTGAACTTTCACAGTGGGAGGTATCCCGGGCTGTATTTGTGCTCCTGACTTGAGCCTGGACACAAACTA
GGGGCAGTGAGCATGGCTGCGGAGACACTGTGTCCTCTAGGGCTCCTAGCTGTGTATCCCATGATGCTCCTCTAAGCTCCA
GGCCACACAAATACACAGCATCCACAGGAGGTGAGGCTGGGTATGTGCTATCGCTCAGAACCTGGAGGGAAGAAAACAACA
ATCAACATGCAGTAGGAACTATTTTCATCCACAGAGTTCCAAAATTTAAGGCTATTTAAATATGATTTTATCATTTGTATG
CTTTACCTGAAAATGCAGGGCTATATCACTGGTATATTTGTGATTCACTTGAGCAGCCTGTCTTATCACCGCCACCAGGAA
GCCTCTGGCATTTTTAAAAGAAGATAGTCGGTAGCCAACAGGTGGATCTGTGGTGTTGTGGTGCCAGAGATAAGCACTGAG
CAACTCTGCCCTCCTCTCCAAGTGAGACAAGTCAGTGAGCTTGAGGAGGGAAGCGGAGGTCGGCGTGCTGTATTGAATGGG
CTGTTGGAGCTGTGAGAGGAGTGAGGACACAGAATCAATGAGATCATCCCACTCAGCCTGGAGGAAGTCACGTAGGGGACT
GTGAGAAACAGCTCCTGCATTTGTATCTGGGCTGTCATTCTGGTGTTCAAGGTAAGTCTTCAGAGTCTGCAGTCGGTCTAT
AGCGTGGCTAAAGGCTGGCAGTGTGGCAGGCTGCTTTAGCTGGGCGCAGAAGCTCCTCGCTGTTCCTAGAGGAGTCTGGGA
GGCCTGCAGCAGTATGTTCAAATTGTGGCTATTGATTTTGATTCTCTCAGCTGCCACATCCGCACTGAAGCCGAGCACAAG
AGTCAAGTCATTGATGTTCGCTGAATCTTGAAAACCCTGCTCCAAAATCTGCAGTAATCCTGTCAAATCTGACGACATGAA
ACACAGCCACAATCAATACATTGTAGTTACAAAGAATATCTGACAAACAGAAATCCCGGTATTGGCTCTAACATAAACTAA
ATGTCACCTCATATTAATGCTACGGTCGTGCAACTTGCTCAACAAAAGGTGTCAGGTAAATATTGAAAAGGGCAAAGAAAA
AATCTTTGCATGATCCAACTCCTCTAGTTCACTGTCTCATCAGTTAGCTTCTAGGATATTTACTATGCAAAACACAGCACT
TTAAAAAAGTTGTGTGGTTACCAAAGTTGCCAGGATTGCTGATAATATCTGAAAGGATTTGTGGTCCTTTGCCCCAGAGAG
GTGACAATGTGCTGAGGCAGATCTTAGCAAGGCTCTCCACCACCTCCAAATCTGCAGAGTCTAGTACATGTCCACCATGCA
CGAGATTGACTTTAAATGAAAAGAGCAACAAGGTTAAATCAGACTGTAGAGTAACTGAACAAGAAGCATAGCTTTATCTGT
AAACTGTGAAGTTGGATATTCATACAGCAGCTGGGCCCTTATTTTACATATTTTGGTATGTAATTGACACATAGGTCCTGT
AGATTGCCTCAGCCAGCATCTGGACATGGCTGCAATGGCTGCTGAATAATCCTTCAAAGTATGTTCACTAAAAGTGCTTGT
TTTTGCCACTGACAAGCATTGTCATTTTAAATGTCTAACCACATTATGGCAAAGATTCTTTGTGACGGAGTAAGATCCTTT
TGTTTAACCAGAAAAAGCTGCAATGTTGCCCTCAGCAAATTTACAAAAATGGTTATTTTATCATCGTAAAAAAAATAAATT
CATTCAAACTTGCAAACAAAACAAAATAAAACTCTCCAAAATCATCTTGGTTTGTCTATCCACTGTTCCAAAAATCATCAA
CAAAAAGGTTTATCTCTTTAAGCATTCTTTCCTTAATGTCACACACTTGCATTAACAATCTCAGCCTGTCATTGGCAAAAA
CAAGCACTTTTACTGGTCTTACTTTGACAGACAAAGCTGCCCTTAAGGATTACATTGCAGCCATCACAGCCCGTGTTGCGG
CTGCCCGCTGAGGAGTTCGACTGGGCCGATTCCAAACTTCTTAGTACCTATTAGTCATTTACACATGAAAACATGTCAACA
TGGAGCCCATCTTTCAAAATACCAGACATGCTGCCTCACCTGCTATATACTGCAAAGCTTGAGTCTTGTCATGGCAGAGAC
CAGCAATGCTAATGTGTGCATCCATCAAAGCCAGGAGGTCGTCCTGACTCCTTCGAAAGACAAACAATCCAAATGCACAAG
AATAAAATCTAAAATCCATCATTAAATTTCCACCATTTGCAGAAAGTGCCTCGGTTTTAAAATATTGAGACATTAGTTCAG
GTTGTATTTGGTTTCACTGTAAACGGATTTAACAGAAAATTATACAAATATTTTTTACAATGAGACAACACCTCTACAGTA
TGTGAGCCCAGTTTCTCACCAATTGTAAATCCTCCCTTGGCCTAAGTATTTATAGGTCTGTCTCTGCAGTAACACAGAGTG
AAAGATGGCACAGCGGAGGAGGAGCTCCATGTTGTCTGCTGTGACAGCCGACAGTGACTGACACCGTATGATGGAGACCAC
CTGTCGCAAGGAACAGCTCAGCTCCTCCGTCAGGTTCCAGGGAGAGTCACAGAGCAGAGGCAGGGCACACATTCGTACTGC
GGCTGTTTAATAATACAAAATAATAATCGGCATTAAAATCATTATTACAACCTGAGTAAGACAAACAGAGACTACGCTTTT
GCTTGAGGCTATAAATTAGCTCCCTTAGGATAAGATTGTGTCTTTTTGTTCTTGTTTTATTGATGATAATTGATAGCTTTG
GCCCTAAATTGCACACCAATGTACTGTGTATTGTTTCTAAAAGCTCATGATTAGACTGGATCACTATAATGATCGATACTT
AGCAGTAATGGCAATATAACAGATCTACGACCTACCTGGTATAGAGCATGATGCATATTCTTGAGTTATGAACCACAATCT
GAAGCACGGGTGAATCAGGTATCGCTTCTCTGTGCGATGGGTAGGTAGACAGGCAGATAGGTAGATACATAAGTAGAAAGA
CTGGGCTGCAACTTGGGTTGTAAGCATGTATTAATGGCAACAAATGTTACAAATACAATTTAAAGTGACGCATGATGGACT
ACCTACCTCTGAAAGAGGAGATCAACCAGCTGAGGTGAGTCACTACTTTATCATCCCATTGTTCCAACAGGTGACAGTTGT
TGAAAACCAACCAGTGGCCGTCATTAACAGCTTGGTCCAGCATTGAGAGAATGAACTCTGTGTCACACGGAGCCCCAAAGG
AAATGACTTTCACCTAAAACATAAGAAAAACATTAAGCACTGCATTAATATACTCTGGTTACAGTATTAACAACAGAAACT
GTGTGTGTTACCTCCTTTTTGTCTGCTACACAGTGGGACACGCTGTTTATTAAGTGAAGAGGCTGGATGCTTGTCCACTTA
TTTTCTCCTGGACTGGGCATTGTAAGAATGATGGGTCCCTCGTGTTTGACGAGATATCTTGAGAGGGCCTCTGGGTTCCCA
GCGTGAGGGGCCTCATTCTCTGAGGTCTGTCCAGACAGGCACAGGGGGTAGACGGCCATGGCGTCAGCTAGTCCCTCCAAA
CAGTCTGGAAGCATGGTCTTCCATAGCAGAGCCCGCTGTAGCAGGGATAAATGGGAGTGAGAGCGACAGGGAACAGCCCCC
GCTACAGACGAGGAAGTGAAGTGCAGGTACTCCTGCCACTGTGTGGGACAGGTGGAGAGAGAGGCAATCAATCCTTTGAAG
ACTGGGATCTTCTCCAGGCAGAGGAGCTCTGGGTGGATATGAGGTGGTATCCAGCTGGGCAGAGCACTTGTGGACTGTGAA
ACAGGATGCTCTGTGTCTTGAAGGCCCCTGAGGAAAGCTGCCCTCTCAGCCTCAGGGCAGTGCTGGTTGTGTTGGAGCAAA
GCCAAAGACACCAGCATCTTCAGAACAGCAACATGGCTCTTGGAGAGACAAGGCCTGTATTGCACCAGCAGCTGGGCTACC
ATCCAGTATGTGACCTCTGCTATGACGCCCTCTGACACTTTCCCACTGCTATATGACAGTAATGGCCTGCCCTTCACAATG
AAGGCCTCTTGCATAACTATGATGAAGCCATGCAGGGAAAAGCAGTAGGCAGGGGACAGACGGGACACCTCCTGCAGAGCC
TGGTAGAGGGTAGCAGCCAGCCTCATTAACTGCCGGGGGACAGCCAGCAGGGACTCATGGTACTCCAGCTCCTCATTCAGC
TGTTGGATCTCTGTCTGCAGTTTCGTCATTGCTTCTTGACAAACAGCCACGCGGGGGAGAAAATTAGGGTCCTCCAGCAGT
GGGGTGTTAGTCTGCAGGATGTAGTCCATAAGAGCATTCTAAGACAAGATTAGATAGAGTCAGTACAAACAACAACAGATA

ATTATGATACATTATGATGTATAGAGTAAGATTTTGCATCAAAGGACTGACCTCAGATTTAGTTTAACTTAAAGGTATAAA
GTAACTTTTCCATTCAGTGACTACAACAACAGTTTGATTAAAAAACACTTGAAACCACACTCAAAAGCCCAGAACATGATT
GATGTACAGAACTGCAGCCCAGGTAATAAGGTTTACTTGGTCCATAACCTAATATAGATAAGGTGAACATGCAAAGCCAGG
CTACAGCTTGTTGGCACAGCATTAATTATATGGAAAAGGATTACTCGATAATCATTGAGATCAAGGGATATCAACCAGATC
ACCAGGCAAACCTCTAATTTTATTAAGTTAATTTTAATTTTTGAAGATACATGTCGGACTCTGAAACTAGCTATTTTGGTG
CATAGTTAGGAAGGACACTGACAATAGCAGTTGCCATGGAATCAAAAGCTAATGACATAATTAATATAAATTAATCAGGTG
AAAGAACAGTCAACGCTGATGTGTGAGAGGAATGAGAGAGAAATTTCATTCATCTTTTGTACAGGCTACATGAAATGATGT
AAATCCCAACCAAACTGACCATGGCTATGCCTCAGTCAGTCAGAAATCATTTGTATGCCAGTTGCAGATTGGGTCTTTTAC
ACATTCAAACCGTACCATTTTGTTTAAGAGAGAAGCACCCGAATGTCCCGACTGATTTCATTCTACTTCACTGAATCAAAT
GAGGCTATATAAGCAACAGAGACAGTATTTGCTTGATATGATGGTAAATGATGGTATCGTCACCTCTTCTGAGACCAGTTT
CTCCTGCAGCGTCTGTTTGTCATTCTGCAACTGCAAGTGTCGATTCAGCAGCTTTTTACAGTCAGACTGCAGCAGCTGTGT
CAGCATCAGCTCCTGGATCTCTTCTGAGCTCAGAGAAAGGTCAACCACTCGGACCTGAGCCAAAATGGAGGGATGAATCTC
TAAGGAACAAAAGCAAGAAAAGTGAGCTACTGCTTGCAGCTGAGTTAGAGGAGTTAATTTCTAATATGTCCAGAGTGTCTA
CAGTACTTCATTACGTTTGCTGAGTTTTGGCGATCCATGTTTGTAATGTTTGTAAAGATCCCATTAATCTAACTCTTTATG
CTCAGAGTAACACAAACAGTATAGAGTAGTATGCGGTCTTAATACTGACTGTGATTGTGATCCGTGTAAAGTTATTAGTAT
GCCTCTGTAGCAGTGATAACTTTAGCCAGTCAAACCAAGCCAAGACAAACTTGTTTACATTTATTAGATTGGGCTGACAAA
TGAATTTGGAAATTCTGTACTTCTGTTGCAAGATAAATGCCAAGTAAGTGTTTAACAGACTCCACAAATCCTTCTTACCAC
TGCCAAGCAGTTTGACAGGCAGATGGGTGGTGAAGAAGAGGCAGAATTCAGGGTGGGTCGGCTGAACATGTTGTTCTATCC
CTGGAAGGCAACATCCAGCCGGTCGAACCAATCTGGCCAGAAACTGTGGGCTGGGAATTGCACGCTCAACGTTTGTTACCA
GGACACTTAAGCCTGCAAAACAAAAGGTTAAAGCTGAGAAAAAAGCTGAGAAAGGACATATAATTTGGTAACAAATGGACT
ACAACATGATGTAAAAGTCTCATACTATTGTGTGGTTTAAGTGGTGTGGCATTTCTAATTCACCTTCCTCCGCAGCTTGGT
CGATCTTTTCCATAAGCTCTGGATCATCAGCACAAACCACCATGTCACACCCTGCCTCCATCTGAAGACTGGCTTTTTCTC
CTGCAGAGCAAACACACAACCGGTTTGCTGTTTTTTAATATAATTTGCATTTCACACAGGCATTGTAGAGATTTTAGGGGG
ATTTAAATGGCACAAGGTGCAACAAACAGGCAAACCCCAGCAAAAATGTAAATCTAAGGACTTACTGTACCTGTGAGCCAG
CTTTGTGAGCTTATATCTAGATGTTGCTGGGTGTCTACCAGCAGAGGCCAGCACTGAACCCACGCACTCCTGCAGCCCCAC
AGCAGCAGCTTTACAAACATTCTGGCAGATGGAGCATCCTGGAGAATACCTAATGCCTGACCCACAGACAGCTGCAGACTC
TCAGACACAGCGATGGGAAAACCAAGAACAGGTAAAGAAGGTGAGGCATCAGAGTGTGTAAACAGGGAGGTTCGTGGGTCC
TCTCGGTTTATATTTATGCTTCCAGTCTGACATAGCTCCCTCCACTTGCTCAGCAGTTCCGTGCGCACATCAGGCCCAAAG
GGGCCTAAATAAGAGGTGATTGCTGCCAGGATAAGGGCATCTCCTGGTAGAGTTTGGTTATTTAACTCTGCCTCCTGAAAA
AATCAAAAAAACATTATCATTTAAAAAAAGATTACCAAGAAATAACACCAAATTTAGTGGCATGTTTAAATAGCCTGTACA
GAATATTTGCAGATTCTGTGTTACCTGAGCAGCAGCCCTCCAGTCTCTGTAATGTGTCTCCAACTGCCTTGCAGCTGTAGT
AGCTTCTCTTTCTGCACTCTCAGCCGAGCGCAGCTCCAGCATTTGCTCCTCCAGTTCTTTTTGTACCAACCAAAGCTGAAG
CTTGACATCCTCTAGATGATGGCATGCGTCCTCCTTGTGCTGCTTGGCCAGGTGCAATTGACCTCGTGCCTCTTTGGCCAT
CACCTCCAACTGCCGCTTGACCAACAGCTGATGTTGTATACAGCAGCACTCATACTTAGCCTGCACCCATTGACACAGGGA
CTCACAGGCCTTGCTCACCTCTCGCACAGACTCCGGCACAAAATGGGGACTGTGAACTATCTGTCCGAGCTGCTGCAGCTG
TTCATTTGTCAGAGTGTAGCGGTCAAAAAATTCCAACTCCTAGGAGAGATATTAGAGAGAAAACAATCCCAAATCAGCCTC
TTAAGCCACTGTATGCCATTACTCAATCAGTGCTGACCAATATAAACTTCTGTATGAAGCCACTCTACTACTTTTACTATT
ACTATTTTCCACACACCTGGAAAAAGTTGGTTTGTCCAAGAAGCTGTTTAGCACTCTCCCAGCCTGGTGGACGATTAAACA
GCAAACAAATGGCATCCATAATTTTCACAACTCCATCAGGTGGATCTCTGTAGTGTCGGACCTCCTCCAGGTCAGATGGAT
TTAGACAGTTGAGAATTTTGAGGCCTGACAAAAACACAGGTCTCACCTGGAAAAAAATAAATACCATATTAAGATCCTTTT
CTGTGACACTTCAGGTAAATCCCCTAAATTGTTCTCAGGGATTTAGTCCAATTTCTAAACAGCCTAGGATACTATGATTAC
AAAGCTAATAAGGAATGTTTTATAATGTGATCCGATATGATTTAAAGGATGCAGATTTTAATAAGACAGGAAATCTGTTTA
CCTGTTTCTGGGCGTAATTAATCTGCTCTTCCAGGTCATCGAGCTTGTTTGCCACTGCTACACATGTGTCCCGAGCTTCCT
CAAGCAGGCTTCTCTGGTGATCTGCAGCTCTGAGGAGCTCTTCCATGCGCTTAGGGATGGCAATTTCACAAATAATGTGCG
TTATAGGGCAAATGTTACTGAATATGAGAATTTCAATATGAACTGAAGGCCACTTGCTTACTTTAACATAATCTCCTCTTT
AAAAATTAGATGCAACAAAAAGAAACTTGCTGTTAAGAGTTTCTTAGTTTAAAACCAATATTTTCTCTGGTGCGCAGAACG
TTCTGAAGCCTTCACAGGCATCTGAAGTGCACTTGATATATTGTTTACAAAGTTATTCAAACCATTTTTGACTGTGGTGAA
AAATAACCTCTTGTGGTATAGAAATTGTGGCAGTCGATACAGTTGCAGCCACATACAGAATACTGATCCCACTGGAACTGC
ATATGCTGAATTGCTATATATTACCTGCTGTGTCTCAGCAGCCTTCTCCTGCAGTCTTATTAAGTGCTGTTTGTACTGCAC
AGCTGTGTTGTTCATGACATCCACGTGAGACAGGACACTGGCAACTCTGAAAAAATACAATAGTTAGTAGAGCAGTGTGTG
TATTTCTGTTTTCAATCCAAATACTCTTTGTGAAGGTTTATTTATCAGATAAAGCTTGCTCACAGTAGCATAAACACAAGT
GTGTTGGTAAATCAGCAGCAGCACATTAAATACTGAAGACACAGGGAGAAAAGAGGAAAAAATGCTAATTATCCAATGGTT
TCAGAAGAACTACCTGACAGCTTGGCTCTGCCGTAGCTTGTGCAGATGGTTGCAGAGGTAACCAAAGTGGGAAATTAACTC
CATATAAGTCTGAGGGCTGAAAGGCTGAGCTCTTAGAAGGACAGCAGCATACTGACACGCAGACTGATGGATTCCTGCCAT
AGCAACAGACAGGCCGGCCTCAAGCCCCTCTCGCTTCACTAAAAAAGAAAAGGGGAAAGGATATGTCTTGCCAGTAAGAAG

AAGTACAAATTAAGTAAGTATAGACTAACTTTGGCGGTGTGACTCACTTTTGTGGGTACTGGATTTTAGGCACTGAGCAGC
AACTTCCACTAAAGACTGGTGGGACCAAGGCTGGTACACCTCCACACAGCAACTGACACTCAGGGCCTTGGTCAGTTGTGC
CTGGATCACAAAAGATAAGAAGTCATCCGTTTAGATTTACTGTATAAAATTACTTTATAAATCTAACACACTTAATGGCAC
AACGTGTGCTATGTATACAGAGCCCTTTACACCCTAAATATCACATTACAGTAGAGTGAGCATGGTCTGCATGTTGGGGTG
CTGCTGGGGTACAAGGATCCCACACCAGAATCAAGGGTTGATCCTGTAAGTATCCAACTATAAAAGTATGTGTCTAAATAG
TTGTTGCCTGGATCTTCTCCACCTGGTCTGAAAAGTGCATTTTGTGTGTTGTTGTAAAATACAGTGACAAGTGTTGATACT
CAAAGAAAATACTTATTTACATTCCATCCATGGGTTGTTTTGCTGGCAGGTATCTCGCTGCTGTCTGACACGGTGAGCGGA
AGCAGCATGAACACATGCACATTTCTGTGGACTTGGCTCAAGTATCTTTGTGAAAAGAAACAGAACAAATATGGTTAACTA
ATATTTTCTTCAAATATTTACTTTAAGTAGTCACACATTTTCCTACAAGTCCATTCTTGTAAATGCTGTGATATCATCATC
ATCATCATACAACTACAGTATAAATTAATGTAGTGATCAATACCCAGATTACAAGTGCATCACTTTTGGATGTGAGTAAAT
ACACTAAATGCATTTCTTAGTAATTCAATAACACAATTTGATGCCTTTTAATAGTTACCATTACCAGTTATCTCTGTGGCA
AGCTAGTATGATGACCAATAGGTTTTCTAGGTATTATCCTCTTACTTCTCAAACATCCAACTGTCCATAAGGTATCTTCTT
GAATTCTTCACAGCAGTCACTCTGGAAACGAGATTTCTCAGCTCCTCGTCTGTGTAGAGCCCTGGGTAAGTTCTGTGAGCC
ATCCCCACCAAGAGTTCATCTCTGACAGACTGGCTAATTCCTTCGTGGACCAGTATGATGACATTAACTCCATCCGCTCTA
GTTTGATTCCCAGCCTCTTTAAGGATTTCATGCAATTTATCCTCATTCCCAGGATGCACCTCCATCAACTGGTAGCCCATT
ACATAGGCAGCTAAGCACACAGTGGTTTTACGGCCTGTGCCTCTGTCTGAGCCAATCAGCACTCCGTGACCTCCAGGTATG
AGCAACGCCCTGAGGATATGTAACAGCTGAATCACCCTCTGTCTGTGTACAATGACTCTGGTTGTAAAGTTGTAGTCATTA
TCATATTTCTGTCCTTTTTCTTCTGCTTTTCTGTCAATAAAATCACACAGCTGTTGCAGTTGTACATCAAGGTCTCGGTCC
TGATAGGAACAGGTGCGTCTAAAACTGTGCTGCTGATTCATTGAGTTTAGGGCCTCTGAGAGGTCAATACCATAAACAAGC
GTAGTTATTATGTGCTCCATACGCTGAAGAGTCTGGGGTTGCAGAGGGTGAGTTTTTAGATGCTGACTGTTTTCAAACAGA
AGGGGAGATTCAGTCAATGTATGGCCTATGTTCAGGTCTGACTGGTCAGCTGGTTTTGGCTCTTGAGGCAGAGTTAATGTA
TCTACACTCTGATCAGTTGGCCTGCAGGTGCTTGCTGTGTCCATGGGTAGTGTGGGAACAGCAAGGCTTGTCACAGCAGGT
GGGTTATCTACACCAACAGGCTTGGCTTCCTCAACCAATCGAATTCCATAGTGTGTCGTTGCTGTCTTGGCTATGAGTGAC
ACGAGAGTTTTGCGTTCGTCCTCTGAGCATAACCTATCACAGAATGTACGCATGCACTCATGCATCCAGAGATGTACTATG
TTTAGCACTGAAGCTGCAGGCCCTGACAGGTTAGGGAGAAAGCCTGGAAGTGAATTCTCCGTTTTCTGCATTGTGTCTGTG
ATGTTAGGCTGCCACAGGCACAATCCTGAAAACACCTTTTCAAGGTCATGATGGGAAAATATGAAGTGGGGCCTTTGCAAA
GTAGGCTGGAATTGGTCACATACTGCATGATACAGACTTTTAGTTGCAGTGATGATACAACATGCCATGTCAGCACCACTC
TGCATAAGTGACATGTCTTTGAGCCAGATCTTCAGCCGAGGAGAATGGATAGACAAAATAACGTCCCTAGATAAGCTAGGG
AGCACAAAGATGGAGAACAGGCGTGAGAGCCTGGAGGATATCACGTTGCTCTGGTGATTGCCCAATCCAAAGACACAACAG
GTAGCCATGTAGCTGATGGTTTCAGAACACATTGACTTGAAGTGATAGGTATCAAATGTGAGAATCTCTCCCTTTGAAATA
CTCTGTCGTAGTGTCTCTAGTGATGCTGACATCTTCCCATAGACATCTATCAACAGGAAGACAGAACAAATTAACTTTATA
AAGCTTCAAGCGGTGCAAAATCAGATTAAACTTCCATTTTAGTTCTCAATAAGGTAAATTCTTTTGACTCACCACACGGTG
CTTCATGCAAATCATCCACAAAAAGAAGCACTCCAGGCTTTTTTGTCATAGTATCTTTACAGTTCTTCTGGCAGCTGATTT
TATTCAGTATAGTGCGCAGGTCTCTGGAGCCCAGCAGTGGACTGGCTGGTAGGCTGATATGTGGCTTGTCAAAACTCAGCA
AAGTTTTGCACAGAGTGGTTTTTCCAGAGTTAGGCTCTCCTGCGAGCAACACTGGCTGGTTTGCCTCCGACATGATGTTGA
GGAGATAGGTATGTTTCCCAAACTATGCAAACAGATTTGCAACACTTGTTAGTTGAAATATTTAATTGCAGTAGCCTAAGC
TCTACATTATTGTACTTTCAATACACAAAATTGAATGCTCAGGACAAACCTTGGGAGAGATGTAATTCGTCAACAGTGTGT
TCTTGGGGCATATCTTGCTGTCGATGTTAAAAAAGTGATCAAACACACTCTCCTCATCAGGAACCACAATTTTGTACCGAC
AAGTAAACAGCACTTGACGGGCTAGTAAGTCAAACTGTGGCCAGTGTCTAAAGTAAAAACATAAAATAAAAGAAGAAAATT
AGTATTTTTTGTATCACAAAAAACAAATGATGATGTGTTCAAAACTTAATACATTTTTGACAGTTGCAGGATTTGTCCCAG
TATACCAGAAAGCCAGTACACCACTGTATATGTTAACAGCAATAACATACATACTTAATCGTTTGCATAATTATAAGTGAC
TACATATGCAAGCTCCATAAAGATTCTCTTACCGAGGATGCAGATGTCCACTAAATCCCCAAATGTAAGCCACCAGAAAAA
GGTTCCTGGGCAGCAGCTCTTGTTTAGTGTCGGGATCTGTGGCAGTTCTGTGAATATCTGGTGAGAGAAGAGGTGGTACAG
CATTAATGAAAGTACAGTCATTTCTTTAACTAGTGTGTACGTTTACGAGGTAATTTGTAATGTGAGTAGTGGTTATACCTG
TTTGTTGTATTGCCTCAGCTTTTTCCACTTCCTTCCTGAAATGGTGTAGGAGGGCACGCAGGATCCGAACAAATGACATGA
TTTCTTGCAGTCCATAGGTGACACTTTCAAAACATTGTCCTTCATTATGGATTGCAGAGGTTAATGCCTCTTGTCTGAGCA
AACTCAGAGTGCTGGAAAAAAGATCTCCAGCTAGACGAGTCCACATTTTCACAGTTCCTTGATCCAGTTTGTGTTCAGAAG
AGAGGGCATCCATTTCACTCTTCCACACAGCCTTCCACAGATCAGTACCTGTGAAATAAACAAGGCTACAACGGGTCACTG
CAGAGGGACTTGCGCCATGTAGGTTGGTGGTCTCCATAAGTAGTTTAAGGTGTGATTGGGATGGTAGTGTTTCACCTGATG
ACAGGCACAGAAATGGGTCCTCAGGACTGCAAAGTGTGGTTAAGTAATCTAACCAGCCAGGCTGTCCCACAGGCTCCCCAT
CCATTACCAGCCATTTCACTATTGGTGTCCGATTACTTTTCTTGTTGCAGACTTTAGACCTTGTTGGTTCACGTCGTTCTA
AATCTCTCAGCACCTTTGCAACAGCTCCATCTTGCCATCCTCTCTTTTCACAGAAGCAGCCAAATAACTCTTCATGGACA
TGGCATTAGGAAATAAGACCACAGTGTCAACAGAGTTCCAGGTTGAAGGAGAGATCTGTGGCTCTGCCTGTGAAGTATCTC
CTTTGGTCACATTGTCGTTTTCACTCACATGTTCCACTGCCTCGGCAGCCAGACAATTCAGTGCTCCAGCTAGAGCACAGT
AACAGGTTGTCTTTCCACTACCTGAGGGGCCTAAGAGCAGAACTGCCTGAGATATTTTAATGGCCTGGTAGAGTGTAAGAG

CACTGCTAATTGTTTTGGTGTCAGAGTGACACTGTTTCCTTTGTAAGTCTTCTGTAACTGCATCTTTAAGTTGGTTCTCTT
CTTCTTCCTCAATATAGCGTTGGAAAAGAGGAAACTGACAAGCTAATGGGAATGTGTCCCTGAAAATGATGTAGAACTGTG
AGGCTTTCTTGTGCTCATAAAGAACAGGTAATACGACAGACAGAATGGCTTTAACAATGGCTGTCTCCTCCATTAATCCTT
CTATAACTGACAAATGAGAACGGCGCAACCTGGAAAGCTTTTCAGCTTCTTTTCTGTCCTTCTCGACAAATCTGGCAGTCC
CATTTTGTGAAGATGTATGATTAGTTTTTTCTGCAGCCGATCCTTTAGCCTCATCCGAACTTTTCCGTTGCCTTACAGTTT
GTTCGAAGTGTAATTCAGAGGCGGAGATGATCTTTTGCAAGACAACGAGATAGCAGCTTTGGTTGTCAGTGATGAAATCAG
GCAGACAGAGGGAATCCTTGGCCAGGCTGATGAGGGACACCAGACGCTGACTTAAAGACATGGCTTCTGTGAAACCAATGG
AAGTCAGCATTACTTCTGCGATGATCATGTAATCAGGATGGGTTAGTGCAATGGGTCTGGTTGCATACCGCAGACTCTCTG
GAACCTCAGATGTAAACCCCTTGGATGAGATGAGAACACACCCATAGTGTAGGCTGGCATGAATGCTTTTCCCTGCAAGTA
TCATTTGGCATTCTGAATCAACATTTTTGTAGCCTGTGACCCTCTCAGCAGTCCTGTCATTTGGGTCCTTATTCTCTCTTT
GATTCTTCTTTTCTGTCAACCCAGAGAACGATTGGTGAATGTCTACAAGATGCTGTGCCAGTAATGACAGGACCCCTTGTG
TTAGTAAGTCTACAGAGTCCAGTAGCAGCCATGCTCCAGTCTGGAGGGCACCCAACATCATCCGCTGAACAACACCTGGTC
TCATGCTTGGACAACACTGTATGCTGACAAGCTGTCGGCCCAGCGCTTTTCCTAACTGGACCACAGTCGTTTTCTTTCCAG
ACATGCAGGGTCCACTCACAAACCCACATCTATAGCTTGTCATGGCAAGGAGAATCCCCAGTATTGCCCGATCTGTGGACG
GAGAATGCACAATCACCCAATCTTCTGGACCAAAATACTCATAACCATATTGGAGTCGATGGCCCAAAACATCCACATAAC
ATGTCGGTCCATCATTGCCTTTCAGACTCCAATTTTCTGAGTTTATGTGATACTTCATCAGACTTTGCCACTCAAAAGAAG
ACTCCAGAGGTACACCTTGCACTTCCATGAGTTGAGATAGCTGCTGCGAATGTTTCATTGTAAGTTGCACTAACACACGCA
GACACGTCATCGTATACTTAGTTTTAGCCCCTGTGACTCCATCCCTTATGGACTGTCCAAGGTTTTTCAGCTCTGCAGAGG
TGTGTGCCTTTATGTTGCTCAGCTTCACTGGGCTCAATTCTTGGAAAGCTTGTAACACAACGTTGCACCAAACTGCCTCCT
CAGCCACCAGCAGACACTGAAGAGGATAGTCAGACAAGAGATCCAGCACAGGCATTGCGATGTTCCTCTTATCGGCAATGT
CGATCAGGACGTCTCCAACTTTCTTATCACATTCCAAATCTTGACTGGATGGTTCAAGCTGGTTTCGCATAAGAGCACATT
CTTTCATGAGCTGTACCATGGTCATCTTCAATTGTTTCTCGAACAGACAAAGCCAATCCAAGGCACTGAGATTTGGTTCCA
AAGGAGACAGAAAGGTAATGTGCTCCTGGAGGCTGCCAAAAAATCCCAGCACCTTCATCTGTCTGCGGCTTTGAGACGTAG
CCTCAGAGTACTTTACATTTCTTGTACTAGATGGTATTTCACGATCCACTTCGAGCCAACGAACCCCTTTAAAGCATTTGC
GTACAACGGGTTGCAGTGTGAAAGGCGCTGGGTGAAAAGAGAGTAGCTGTATCACCTCCCTGTCACTCAGAAACCAGAGTC
TTGGAAACTGTTCACAGAGGGTGTCCAGGAGATCCACCATCTGGTTGGAAATACCCTCCATTGTAGACAGGCCATCCATTA
GTATTTGGCAAAGGTTATTGCCATGGAATCTGTCATTTGTCTTCTTGAAATGAACAAAGTTCAACACATGACGGTCACTGG
CTATAGAGAGCATGATTTCCTTAAATGTCTCATCAACTGGTCGGAATCGGTCCAGCTATGGGGAAAAGAAATACATTTATC
TGAAACAGAAACTTAAAGAAAAGCTCTGCGAAATACCAGTTTTATAGCAATTCAAAAGGTAGTTATATGTGACTTGTGATC
ATGAAACAGGGACATACCAAATCCACTTTCTGAAAAGTAAAGTTTTGATGGAACATCTTGGTCAGGAAGGCCCATATTTGC
TGGTGTCTTTCAAATAAAGAAAGCAGCTTCTCTATTAATTATTAAAAGGGGAAGAAAAACAATACAATGTTCATACTATCT
CTACACTTTGTGGCAAATTTTCAAAATGTTGATTTCTATATTTTATTCCTGAATGTCCCCCTTACCAAGATCTTGTAGTGA
TTGCACCAGATTTTCCATCTGCAGCCTGAACTCCAGACTGTCGGGGGACTTCAACATGATGGACAGAGTCATCAAATCATT
CTCTATCTCAGCAAAGTGTATCTCCAGACCTGAATGAGAAAACACAAATCGCAGATATTTCGAAGCAGAATATTGATTTCA
CACGGAATCAAAGTAGTAACTGCTTGATTGATGATTACATCTCTATTACTAAGTTGTTGTCAGGTTGTTATTCAGTCTCAC
CAATGATGGTGAATCTTGCATCATGACAGGAATGTTGACTGGCCGTTCGGAGATTTGAAACGGTGCCCTCTGTGGGCTTTT
CTGTCTCTGCTAATCCCTGTTGTGGCTCACAAACAGGGAGAGTGAATTTCTCCAGCTCCAAGATTCTGGCTTCCCAACCCT
GTTGAAGTTTGTGGAAGGTCTGCTCCATGTTACACTCTGCGTCTGCATCCCCACATATCTGATGGAGTAAAAGGAACCATG
AGAAGCAAAATTAATCAAGTATCGTGTGTTAATGACATATAAAGATAGTTACGCAAAGGCAATTATTTCAACCCCTATTTG
TTCTGATTTTAGTAGCGCTGCCATAACGTGCTCAATATGCAACATCTCTCCTGCTTTCACCTTGTTAATGAGTTGCTGGTC
AATTTGAGGTTGTTGAGACATCAATTCAGCAACAGTCACCTCCTTCTCTGGAACATAGCCCATGCCTGCCAGATGGAAAGC
ATTTCAATCATTTTAAATATCCTGTAATGATATGCATGTGTTAATATATGGGAATGTCTCTGACCTTCAAAAATAGCTTTC
CAGTGTTTATGTTTGAGCGTGGGGCTCTGCAGCTTGGCCATGAATGTAAGCTGATGGCTTAAGCTGTCCAGGTTTCCTAAA
GTCTCCTGCAGCACAGCATCATCAGTAGGTATGATACTTGTTAGAGACAGAGCTTGCTCCTTCCACTTGGCAACTTTCCCT
TGAGCTTGTGACACCCACAACCTGACAGAAAAGTATTTCAAAATATTTGTTGGTTTAACTCCCATGGACAATAGTTATAGGG
TTTATTTTAAATCACAAAACAGAATATTCTTACTTTTATAACTCTTAATGGTTTATAAACAGTGATTAGCATCTTTGCATG
AATGTAGTGTGAACCAAGCTGTGCTGCCGATACTCCAGCTCTTAACATCACTACTTTTTTCTCCAAAGATGGCAATTAATT
AAGGAGAGACTGAATTCAATTTTAAGTACATTTTAAATTAGCTTTAAATTTCTCAGCACCATGAATGCGCACAAATACAGT
AATTACCTCCTTGCAAAACATATTTAGTCATCATGTTAATAAGCAGAAAGAGGTCAGTTTGACATGTTACCTCACTGAAAG
AGAGCTGTTTCCATTCTTTCAGCCATGCTATGAAGGCATCTCTCAGCCCCCACAACTCTTTACGGGCGTTGACCTTCTGAA
CATCTGTTGCCAAAGTGGTCAAATCCATTGGATGTTCTAATGGGAGGCAATAAAACACAGTCTGATAAGATTAGGGTTTCT
GTAACTACATATTATGACAGTTTTACCACTGCCAAGCTTCATTTTTTCTTTGTTTAATTTCTCATTTAAAATACATGTTTA
ATCCACCAGTGGTCAAAGCTAAAAGCACAACATCAAAAGCATTTCTTTCAAGCTTCAACCCATATTTTGTACCAACTCACC
TTGTAGGTCTTGACTGTTCCTACTAAGTTGTTCCAGCTTTGCACTGAGATTGTGCACATGTGAACACATGTGGTTCAGTGT
GGAGACCATCTCTTTTGCATTCTGGGTTGGGTCAAGGAAGGGTCCAGATGTAGTTTTAGACACCATATTTTTAAGGTCATA

CACCAAAAATGAGAACATTGTGTCCAGTGCGTGAGCCATTGTAGGAAGACGCTGGCACACAATGCTATCTGCTTTCTTCAA
GAGGGGAATAAAGCAATCCCACATGTCCAGCATCTGAATATAGGACACAAACAGAAGCACACAGACCGCAGTGTAAAAAAA
GCTATAAATGAGACATTACAGCTGTAATGTTCCAAAATAATGCAGGAAACTGAATTGTACAAATATAATGTGGTTAATACT
GTCATCATTTTCACTTTTATCCATACAGACCTTTTCCTTCAAAGTCACCTCCTGTTCAGTCATCTTCCTATAGTTACTGCA
AACAGTGTCCTGGAGGGAGTGAATGTACTCCAAACGTTTCTGTAGGTCAGCAAACATTTTCATAGACTCCCTCACCTGAAG
AGAGAAGACAGATTTGGACTTTTTAAAATCAATTTTAGGTGTTTATCATGACTAATAACTTATGTTTAATTACTGATCTAA
AATGTATCTCCTGGCCCTAAGTTCATGGAGAGTCCAAATACTATGTACCTTCAATTGTTTAGAATGAAAACTTGAATGTGA
AGATGAAAAAGCTCTGTAATTACAGCACTTGGTGAGTTTCTTGTCCCTTAGACTCTTAAACAAAGTCAGACAACAGTAATA
TGTAACATGGTTTGTTTTCGGATCAGAAATCATTAAAATATCTGGTATCAGATGAATAAAATCTCATTATGCTACAGAGAA
TAAAAGATTCATTAAATGTTGGCATGTTGACAAATAATTGAGAAGACTGAGAGAACGACCTCATGCCTGTAGATTGAATAA
ACAAGCTGGGTGACGATTAGGTAAAATACAACCAACCATAAACTCTGTCTGACTTGAACCTGCACTTTGAACGTCTTTAAC
AACTCACCGTCAAAAGAGTTTACTTGAGTACTTAAGTGTACACTAGTGTTCTTCACTTATATGCCTTCAAATAACAGCACT
GAAAGCCTTAATGCACTTTGAATCACCTGAACATTCTTGGATAATGGAGTCTAAATTCAGAACACATTTGCTCTAACAAGA
CAAAGTTCTTAAGATGAATAATTAAAATGAATATGTGACCAAAAGAAGAATCTCAGAGGATCTGAATCGTCCATTTGCAAA
CCCTGCGAAGGTGAAACAGGTAAAACTAGGGTATTGGCTTCTTATGAATGTCTTTCTTATTGTTTGGGCTGATTTTTCTCT
TTGCGTGACCTGTAATCATACAAGTATGTAATGTGTGTGTTTCTGTACCATAACAGCATATTTTGAGAAGTCATGTAAGTC
TTGGGGTTCTGGCTTGAGTTCAGCTGCAGTCCTCTCTAAGTCTGATATGACACTTTCTGAGAGCAGCTTTATCTGCTCAAC
GAGCTGCTCCAGCACTTCCTGCTCAATCATCTTCAGCTGTTGCCCTTGAAAAAACAAACGCAAAGAGTAATAAAGCATTCA
GGATAAAAACAAAAAAGTAACCCATCAACTGGCCATCCAGTTTACTGTTTTACAAAGCCAGGGAACAATAAAGAGTAATTT
CTTTTAGGAAACATTGTTTCATCACTCCTCCCTGGTTTGGGGCAAAGTCAATTCAAAAAGTTTTTGAAAAGGATTTTGTAT
CTTAGAGTCGTCAAGTAAGACTGAAGCGAAGATTTAATTACTTTAGAGCCGACATACCTAGATTGTCCTTGATGCAGTTGC
ACTGGATGGTGAACAGCTGGTTGGAGGTGGAGATTGATGAGGGAACTGTGTGGATTGCTTCTGTCCAGTGGCGTACCTTCT
TTATATGTTCCTCATATACTAGTGCAGGCTGACTCATCATGGATTCCAGAGAGGCTCCACTCCATTGGCTAATGAACATGT
GGATTTCCATCAGCCATGAGTAGCTCTCGCACAGCTGCTGGATTTCTAGCTCAGCTTCCTGTACAGAGAACAGTGACAGTT
ACTAAAAGCACATGAGCAGGTGTGCAAATTAGTTAACATTTGATTGATGTGATAAAGTGACAGTCTTGCCTGCATAATCTT
GGCCTGCTCTTTCTCAATTTGTTTTCTAACATCGTTGATGCTGATCTGCCATTCCAGCTGCCTCTTGGGGAGGGGATAGTA
GCAGCCGTGTACCCTGCTGCCTTGCACCATCAGCGAGGTCTCTTGAGGCAGCACCAGCCAGCGAGATGACAGGTCTCTGAA
AAACTTCCAGCAGCAAAACTTTCTGCAACCAGCTGTTTCATCACTGTTCTTATCCTCCCCTGGGAATTAAAACACACGGAG
AATGAAAATAACCTGTCTTCCCACTGTTTACAAAATAAACGACATAAAATATAACTGCTCTGGCAAATTTTCATATGAATA
CTGCAGGTATGGATGAGCAGATGCTCAAATTTATTGAGATCAGAGCACTGAAAAGACCAGCAACTTTAATGCAAATGGCTG
TACCTGTAAAAGTCAAATGTTCAATACAACTGCACTCAGAGGTTAATTCCTGAGAGAAACCAGAGCTGGAGACACTGTTGC
TGTTCTCTAGGAAGAATCCACAAGTGTCACACATCTGATGGAAAAGAAAAATGTTAAGAGGCTGGTGATAAACTCATTATC
AAAAGGCTGATCAAAATGACAACAACATTGCCAGAATGAACAGATGCAATGAATGAAACTATTAAATATAACACAAGTATT
ACTATTGACGCTGTAGAGCAAATTAAATAGATCATAAGTGTATTAAAGGCTTTACATCCATGCAAATAAACCTTTTGGAGA
TATTGATTTATTCATTTATATTCATTCATTTAATTTTTGTCCTTCACATTTCAGTTTGTTTAAGTAATACAGTCACTCTGT
TAGTCAGCCACTTTTCTGTCGATGGTGTGATGAGATTATGAAATGAAACATTATGTTGTGAACAATACCTATTGTGACAAC
CATGAAGCACAGTTCCAGTAAGTGGCTGGCTTTGTGCAAGTGGAAATTTTTCTAAATTACCTGAATTACTGAATCACCAAC
AGTCAGGACGGCTTCACTCACTGCTTCTTGGAATAGATGCACTGGTGGGTCCAAGGTCAGCTGACTACTGGCACTGAAACA
AAGCTCAGTGTGGAAGAGACAGCACTGCTGAGATGTCTTCCTCTAACAAACAAAAAGACAGCATACATCATCAATAAAGCA
TTAAGATGATTGATGTTTGAATCCATTGTCAATAAGCTTTATCTGATTCTGTCTCCATTATTTTATGTAGTTGTGTTCCTT
CATTGCAATTCAAGTTTTGTATCCATAGCTCTCCTGTTATAACCTTGAGAGAAGTATGCTACAGTGCTCTATCTGAAAATC
ATGTGTTTTAAAGAAAGAGAGGACTATGCTCTCTCCTTACCTTCAAAACATGGCTGACAAAAGAAATGGCATCTTGTCGGA
TGATCGTGACGAGACTCTGCACAATCATTTGGTTGACGAGAGCAGCGAAGTTGCCCAATTTCTGCAAGACGCTTTCAGACC
GAGCCAACTCTTTATTCAACTCCCGCTGGTGAGCCAGATGGAGGTGAATGGGTTCGTAGCTTTTATGTGGCTTTTTAGCAT
ATTCCATATGCAGCTGCAGCTCCTGGTGTGCCTTGTAGCTGCCCTCTTTCACCTGGTGACAAAAACAGCACCGCAGTAAAA
ATAAAACAAGTTCTACAGTGATAAGATTTTTGCAACATATTTATTTTACTTCCTAGCAAACTGACCAGATGGCAGAAATGG
AAAGGCTTCTATCTCAAATAAAACTGACATTTTTGTCTATGCTCAGAAACTTTTCTCATGAACCAATTAAAAACTGTCGCT
CGATTGCTCTTTCATTAAAACTATTCATCACAGCTTAAAACAGGTTTGAGTTTGTTACAACAGAGAAAACTGTACCGCATT
CAAGATGACAGCGCGACACTGTGATAATTCCTCCAGTATCTTCAAACGCTCTTGATTCATGGTTACCAGCACATTTTTTAA
TTCCAGCAAAGTGTATGATCTTGACTCGTCCTGGGGCAGCCGGTGTGTCCCTTTCAGTTCTTCAATGACTCTGTAAAAGAT
ACAGAATCATTGCTAGGTAATCTGAACAACTGAAGCTTAACGTTGGTCTCGTCTTAAAAAATCAACAATTTAACTAATATT
GAATGATTTACATGTGAACGTGACGCACCTGGTGAACAGAAAAAGAGCATTTCTGAACTGAGGTACAGCTATAACCAGCAT
ATCCTGCAGATCCTCACTGCGGCGCTGAAAAATGTTTTTACGCACATTTCTATGCCACCTGGCATGAAAAAAACATAAAGC
AGACGAAGGATGAGGATAAAACACAAAACAAGAGCAGGGAAGAAATATGTACGCTGGAGCACATGTCCTGGAAGATTTCAA
TGTCCAGGACAAACAGAAGAATTTCCGTTCAAATATGGTCTGTGTTTATTGAGCTTAAAGTGGCAACTAACAACTTCCCCC

ACAGTGTTTCCAAGCAGTTTTTTTGTTGCTGCCACTGTTGCAAAGACAACTAGATCGCTTCTGTTTTCCTCAGAGTTACAA
ATAACAACAACATAGGACAAAACATGAGTTTATTCAATCATTTAGTCTATAGCAGAGATGGATCGCCAGTAAATCGCTTTC
TCTGGGGGGCCGCGTGCCTGGTGGCAGACAAAGTTGTACAGTGATAGAGAGGTTTATAGCGAACCAATGTAAGCTCTGCTG
ATTTCATTATTCTTTATCCATTACATTGCACAATCAACATTTACTTCAGAATGATACAGTGGAGCAGAAAACCTGTCTATT
GCCAGGTAAAAAAACAATTCAGAGACTGCTCATGCCGTGCATCACCTACTCAAAGCGTTAGGGTTTGATGACCTGGAAATG
AGACTCAACAATCAACTATCTTTTTTTTTAAATCACTTACTCCACCTTTAATACGATTCAAATTCTGCTTAGAGGGAATTT
GTTTAGACAGACATTATGCATGGAGGGATTCAGATATGTCAGACTTCAACAGGTTTTCCTGGTAAAATAAACAGTTATTAG
GGGAAAACAAAACCCTGAAGAGCAGAGAGAAAGCATAGTTTGTATGTAACTTACCAGGTGAAAGTTTTCCGCAGTCTAAAG
TCCCTGAAAAACTGGATCTCCTGCAAGGCTGTCCACAATGTAGACTCTCTGTGCCACTCTGCCAGACTCACATGTCCACCA
TAACCCCCCCCTGTCACATGCAGGACAGTAGTAGGGGAGAAGATGTAGTGGTCAGAACCAGCTTCACTGGAATCGACCACT
CGCAGGTCATATGGTCTGGAAATAAAGCAGACATATCACTGTGTATTCACTATTTAATTGTGTGCACCCCAGCTGGTACAT
AACCTTATTTTAGTGTACCTGTAAGAGTCTCCGGCCAATTCTTTCAGGTAATAAAGCTCCAATTCCCCCAGGTCTCTTTTC
TTGGCAAAGATATGAACTACCTCTGTGCCAGTGAGAGGAGCTTTATTTGGTTTGGTCACTTTTCCATTTATCACAGTCGTT
ATCTTTTTATCGTTACATGTTGCGATTTTTATGGCTTCATCTGTCCCTGTAAGACTGAAAAAATAAAAAATGAGTGTAAAC
AGAAGAGCAATTAATTCAGTCGTCAAATACTTTGAGGAGTAAAATGTTGATGGCTAAATTTGTCACCCGTTTATGGAAGGT
AATTGAAAAACTTACTGCTTGACAAGCTCCTATAATATACTGTATACACTATATCTGGAAAAATATGAATTACCCTACAAC
CAGACACTTTAACAAAGATCTATAAATACACATCCAGTCCACCTGCAAACCATCAACAACATCAGATTTCTCTCATTGTCC
TGATTTTATCTGTAACAGTGTGATAACAGTAGGAATTACACTCATAACCACAACTTTACAATTTGTATCTGAACAAAGTAC
TCCATGTACCTCTGTGCTGTTGACTCATCAGCAGTTCTGTTAGGGATGTGGGTCCCTAATGCAGAGGCCAGTAGTCGAGGT
CTTACTTGGGCTGTAAGACGAGGGAGTTCCACCACTGATGAAGGCCGAACAGATGGGAAATTTCTGAACCGAGGCCTGGCC
AAAAAAACTGCTGAGAGAGATGGCAGCTCCCGGCATAGTGGAGGTAAAGTCACTTCAGGAGCGTGGGCCTTTTTCTTAACC
TTGGATCCAGACCTAGTAATGTCTCCATCATGGGACCTTTTCTCTGAAGAGGCAGCGAACATGCTGCTGATCTTTCTCTGA
ATACCATCAGCAGGTTTAAGTAGCACAAACGTTGTCTTGCCTCTTTATTCTGGCAATTCTGGAAGAAAAGAACAAAAACTT
AATGTACATTTTTAGTGTGAATCACCAGTATATCATGCTAAACATATCCTGTATTGTGAAAGGGGCACTACTTAGTATTTG
GAGAAGAAATTCAGACTCAGAATTTTAATATTGAAATATTAATGAGGTAATAATACAAACTCAGTTTGTTTATTCATTTTA
TTCAGTCACGGAAACCAACAGAGTTTGTTTATTTAGTTTGTTTAGGGATTAAAAAAATGAGTCGCTGAGGAACTTTCTATT
TTTGCATTACTTTTTTTCTTTTTCTGTGATTTTCCCTGTTTTATCACTTTTAGTCCTTTACTTGTAATGACTCATACTGAC
TTTTTGTGTTCTTACATTTTCTGTTCTGCCTTCTCTTTTTAACTCGTGTTAGACGAGGCTTCCTTGTGTTTGGCTTTACTA
TTGAGTGTTTATTCTGTAATACCGTCTCTCTGAGTTTCCTGTTTTTGCTGGATTGTCAAAGCACTTTGTAAACTCTGTTAT
ATTAAGTTTATTATTTCTCTTCTGATTACAATTTCTTTTCCCAAAACTACATAGTGCTCCTTTGAGTGGCAACATACAACA
AAACAGATAAAGATATACATGATATCAGCAGTCTCAACAAACATAATTAGACAGGGATAAGCAGAAACTTTGAATACAGAA
TTATCAGTTTAATGGCATAAAACAGCCCATTGAAAAATATCTGTAACCAAGATAAGCATTTTAAGCTTTCATTCATTCATA
AAGCCTGACGCTATACGCACTTTTTGCTAGTCAACGTTAGCTAAAGTTAGTTACATTTACACCAGCTAACTAAAGTTTACT
GTGCACCGAGGACGTTTCACTCGTCGCAGTTTAACACCCCGTTCACATTACTGCTCCACGAGAGGATTAGAAAGCAGGTGG
TGATGTTTCTAAAGTTAAATAAGTGCTTTTAAAACAACGAGCTAGCTAACAACACGAGTAGACAAACAAACTTCTGTGACT
CTTACCTGTCTGTGAACGAGTTGTCAAGGCAACCACGGCGACGGCTCGTTGCCACGGTAACGCACTGTTAGTTGTGGAGCT
GGCCGGTGAAGGTCAAGTATGTCAATAACTTTTAGCCTTAAAAGTTTTTATTTAACCCGGTATGTCGTGTCAAGACTGCAC
GAAACAACAGAAAACACTGACAAGAAATCACAGCGAGTCAGTCAGGTGTTACACCGTATTTGGTGACCCAGCAGATCCGTG
TTGTGAAGAGGTGAGGCCTACTCTTATAGCTATAAACATCAACAACACAGTGTAAAAAGTACTACTGCGAGTACTGCTTTT
TAAAATCTTACATTAAAACTTTATTATAACCATTAACAATAGCAGTAACTTTATATTTAATGTGTTTTTATCTTTATTTGT
AGAGGGACCATGTACAATAATAAACATAAATGATGCCATTTGATGCATTGTACCAGAGTTAGCTTAGAGCTAATTTACATG
TGCAGTCATAATTAAAACTTCACATAAAAAAAATAAACTAAAGCAAACATAACAGAGTAACACAGATCAGTACATCACAAG
CACACAATACAAACACACACACACACACACACATAACATATGTTATTCAAGTTAAACACAGTGATATAAGACCAGGAGTGT
CTTTCAAACTTTAGTTAATAGTTATTTCACTGTTAACATACAATGAAATGCTTTATACAACATTTATTTAGCAGTTGTAAA
GTAAGTACACAGTATTGATTAACCCTTTTCAATGTGCTCTAAATAACAACAATTAAGCAATAAACAACTGCTTAATAAATG
TTTTTAAAGCAATTCATTGTTTGTTCACAGTAAACTAACTATTATTAACTTGGTTACCAACTAAGTTTTTTTTTAAGTAGG
CCTAAATAAGTAATAAATACGAGCTGTGGTTGATATTTTATAGCTGCTTATGCATTGACTTATCAATATAAATCATTTGCT
TTTGTAATTTACTTATATATATATCAGTTACAGACCAATTTTCTGCTATATTGGTACTTTCATTCTATTATTTTCAGTAAA
TTTGCTTGATTAAAGTTTTAAATGCAGGATTTTTACATGTAATTAAGTATTTTTATGACGTGGCATTAATACTTTTAAAGG
ATCTTCTTCCAAACACAAATTGCTGGGTCACCAGCTGCACTGTAACACCTGTAACTCTAATATTAAATTTCTCAAGCACAT
CTCCTCCGCTGCCCGTGTACAACCATGCAGAGGTGAAAGTGTAGCGTCAATATGTGAGCTGCCTCACCAACTGAAAGAGTT
CATGGCTTAGTGTTGATGATTTTTAGATGAACACATCATCCTCCGGCATGAAACTCAATGCAATAACATCTGAACATTTAT
ATTTTAGGCTGACTCTTCAGCCTAGTAGACCTTCATGTTGATCAACATCAAAGAGGGTTCAGTAAGAAGCCAAACTCGAGC
CATGACTCTCCCGCTGTGTGCCGAGCAGCAGGTAACAACTTCGTTAACTCTTCCCTAACCTGCATTATAAAGAGTATGACT
TCTGAGTCTGACCTCTTACAAAACACTGTACTCAGTATAAACCTACTCATCAGGAAGTCCAGTATGACCAGATCGATGAAG

TCCACCAGTCTTGTGCCTTTGTCATACGGCGGGGTCTTTTTCACTGTCGAGCAGTAGTCTGGTTCTGACTCCCACCTGAAA
CAGACAGACAGCATTCACTCTTTACATTACCACCTGTGGTCTATATCACAGGTAGACTCTTAGACCCGTTTTAGAGGATTG
TAATCCCTGGTAACACCACTGTAGCTGTTGTTCTGGTCTTGCGTAGAGACATTTGCTGAGCTGAATATGTTGAATCCCTTT
ATCTCATCCAGCATACTGATTCCTCTATAACTAAAGACCCACTAACTTTGCCAGTTTGCTGCGGCTGTAGGAGCGTCTCCA
GGGGGATCTCCAGGACCTGCGGGACGCCAGGGAGAGATTGGGCAGCATGACAGCCAGGGAGGCCTCCAGCTTCCTGGGGCG
ACCACAGACAGCGTGCTCTGTCGAACAGTAGTAGGAGCACTGGCCGTAGAAACAAACGTTACCCACTAGGAGGAGCCAGAG
GAGCACAGAGCAGGAGAGGGGGAAATGAAAATGACAAAAGAAAACATTTGGATGAAAGAGTTTGTGGGTGGTTGTGACCAG
AGAGAATTAGAAGAAAGGAAACACAAATCAAAGAGATTCAGCATCAGTTGACAAACAGGGGCATTTTGAGCAGGCTAAAAG
AATAATGGAGGCTTGTGTGAGCGGTACCAGGGGAAGTGAAAAAGGTTCGAGCCACCTTGCGGTCTATAGTGACATCTTTGA
TCTCCTCGACCACGTCCACCAGCCTCCCCACCGCCGGCGGGATTCTCCTGTAGCCCAAAATCCTGCAGGGAGAGTTGGGGG
GAAAAGGAGGAAATGAGGACAAGAAGACAAACACAAAAGAAAATGGGAAGGGAGTAGAGAAGAAAATACGATTCAGTGTTA
CGTAACGTGTTTTCATTTTGTGGCCATTTCATCCAGCATTACACTCACTGTGCAAACTCTAGCAGTGTTGTACTTGTAGAA
AAAAACTGGACTTGGTACCTGTCCAAGTGAAAAGCTGAGATTTCTGCATTGTGCCTCTCAAAGTCAGAGAAGTAGTAGAGG
TTGTAGTTGGTTTCCTCATCTCTCTCCTGCCTGTCAGAGAGAAAGAAAGTCAAACTATAGCACATAATGTGTTCATTAAAT
TAATATAAGCCCCTTTTATTTGCTCACTTGCTTTGAACAAGCACACACGGGCTGTGGACGTTTCTGTTCTCAATAAAATCC
AACACTAACTGGACATAAGGAAATATTTGACACAATCCTTTCTAATATTTACTCACTCACAACATTTCTCAGTTGTGTGAA
GGATTAAAAATGTCTTTCTATGCATCCACAGTTTCCCCTCGTGATTTACTGAATCGATTCACTCTTTCTTGGATTTTATAA
GCAGTGTGATGGATTTGCCACCTGCGACTCACTTCATGGGTTTGAACATGGCTTGTCCATAGTTGGGAAATGACATCACCA
GCTTCAGTTGCGTCCCCCCAGACTTCTGCACTGAAAAACAATGCAGACAATGTTTCCATAGCAACAAAGCACACAGTTTGC
ACACACACACATGCAACACACCATGCTGTGACCAAACACGCTGGCCTTTAATGGGGCACACATGCAGAACAAAATATAACA
ATAGACAATATGTGCGTCATCGCGATATAACACAGTTCACTCACACCACTGTGTCATCTCTCTCTCACACACACACACACA
CACACACACGGTTTTTGCTCCGTTTGATGGGACATTACTTGCATTCATTCACGTGTCACTCAGCCTTCACTCTGTAACATG
GAGTGAACAATGAGTCAGAAGGACTGATTATTATATTTTCTACCACATATATAGATAATTATTTTTTCAACTGGGTAAATG
TATCTCTATTTTTATTCCATATCTTCAGTTATTTAGACAAAGCACACGTTCTTGTGCTCATTCTCACCGTCCTTGATCTGT
GTTGATTACATTCACTGTCACGCTGGGCTGTAATAGTCACAGACATAGTCTTTCTGTAAGCGTGCTTTGAAGTTTTAATAT
CAAAAAGAATTAATCAAAGCGATGAACCAAATTTGCATAGGCACAAACACGGTGAAAAAATTAACTGTTTGTTCCGCTGTT
TTGTTACTGGAAGTGGAATTGGAAAAATTATTAGAACGTGAGGCCTTGTGCTTTCAAATTACGTGCAGTTTATCAAGGTTG
AGTGAAGATTTCGAGCACAAAAAGCTGCTTCCTGCCGATATTTATGCCACTGCTCGTCGGGGCAAGGCTGTGTCACCTTTG
GGAGATGAAATAACGCTTTTGCTTGGGAAATCAAAAGTTAAATTCAGAAACAATAAGACACATCCTTGCTCAATTTACGTC
AGCGTTACTAGTATGAAAAGAGGCTAATGACAGTACACTTATCTGGTCTGATAATGTGGTTTTCTTGTTGTTAGAAAAGAG
GCCAAGACTGCACCTTCAGCTGATGTCTTTGTTCGGTACTATTTTTTACTAGATGGGTGCGCTCAAACTGATGAGTACTGA
ATACTCGTGTAATAATGTAGTAACAACTACTGAGTAGTCGTGGGTCTGAACGTCAGCAGGTTGACAGGCGTCGTATGAGTG
CTGATATCATTTTTCTAACCCTGTCTCTATTTACACCCTTTTTGAGCCAAAACCTTTACAGTAGCTGCTCAGCTTAAAGTG
TCAGAAGTGGGTGCATAAGCTTGCCTGCACTGTTAGAGTGTAAATAAAAAAAAATCTTTTCAAATTGATTTAGGATCTCGG
GGCTTCCGGACACTTTAGTTCGAGCTGTGTGGAGTTTTAAAACAAGTTATATTTCAGTTGTTGCTGAATGCCATTCTGCTG
TGAAGCTCCAGAGATGTTTTGTGGACTTGGAAACTTCCCCCAAGTTTGAGGGTGAGTAGATGTTGACCAAACTTTCATTTT
TGGTTGAACTTATCCTTTGAAAATGAAGAATAGCATTAAAAATGCACAAGCTAGCCAGATCAAGAGCTTTTTATGACAGGA
GGCAGTCATGTGTTCATACCACCTTTGTCCACGAGGGCCGACAGGATCAACAGAAGAGGACATTTCCTTGTCACAGCTTTA
ATTAAACCAGACCAAATACACAAACACACTGACAGTGAAAAAAGTTAGTGTGGAGAACCGTGGCTGATTGACTGGCTGAGA
TGTTTGCTCCCTGGCCAGTGATTCATACAGTATATTCATATCCACCCTCTTGCTGAGCCCCCAAGGCCCTTCAAAGGTTCA
CAGTTGCTTTCATTTCAAGAGAAGTAAGAAGTAAATCTACATTTGAAAGTCTAACCCTAAACCTCAGGTGTCACGCAATCT
TAACAACCCTTCCCTTCATCTGCCCTCCCTCTCCCTTTTCTCACCTTTTCCACTACAGGTTTCGCCTTGGAGGCAAAATTA
GTCTTGGTTCTGATAGATCTACTGCCTTTCACGACATCTTGTTTTTGTGCCGACTGCAGTCCTCCAAAAGTCTCTCGCTTA
TTATATCAAGAAGTAAATTGAGTCACCCTCGAAGCTGACTCAGATCCTCCTGTCCACTACTTGAAAAAACATCTCTCTTTT
GATCGTCTGACACCTCCTCTGTCAAGCATGATCAGTGTGTTGTTGATTTCAGAGAGAAACTTTGCACAGGCACATATCACC
CCGGCTCAGAAATGTTGTGTCACACCTGCACTGACGATGCGTTGCGTGGCGAGCTGCTTGGTCAGCGGCTCCATGTTGGGG
TCATGACGGGGGTACAGCTGCCAGCGGGAGATGCCCAGGTGGAAGCGCAGCCATGGAGGATGACTGTTACTGCTGCTGTTC
CACTGGATGTCCTCGTAGCCGTCCCGGCTGGCACTCACCCTGCAGAGAGACGGAGGCGAGGGAGTGACCAGGTGGGCGAGT
GTGTCAGCGTTATGTTCTTCTCTCTGATTTTAATTGTGAGACAGACACTGGCACCAGACATGCCTTGGCTAAACAAAGAAT
TGATGATTTGTTCAGCAGCATCATTAATAAATTTACATAAAACACAGGGAAAAAAAGAATCAGAGACAATTTTGACAATGG
TTGAGTCATGTTTTAGGGAAAATTGCTTTCTAAATGTGAAACGTGCTGCTTCTCTTTGGGTTTTGGACTGCTGCTCATTTG
AAAATGCCATCTTAGGTTCTTGATCGCGATATTTTTACTGTGCTCAAAATTTTACAGATAAGACAATAATCAATAACTTAA
AACAAACAAATTAATCTGTTAAAAAGTTAGTTAGTTTCAGCTTTAAATGCACAGTATGTCATTTCTGCCCTGAGGAGTCTC
TCTGTAAAAACAAAAGATACAAGTAGCTTGGGATCATGGGATTTACTGTCTTCATTGTTAAACAACCACTATTGATGAAAT
TCTATCTTTGTGACTCAGACAGAAATGTAAAAACGGACGCGGTACTTTTTAAAGTATTTATTCACGTTATGTTTAGTCGCT

CACGAATCATCTGATGTTTTTCCAGAAGTTTTAGCGACAGGCGACCAAATGAAACACACCGTTACCTCGAGTAAACTCCCC
GATTTCTCTGGGTTTGAACATTGTTGGAAACATTCCAGACCATGTGAGTACAAAGATCAAGGCTAATGCAGAAATGGGTGG
GAAGGCCACCGGAAAGATGCCACAGAATATTACGAAGCATACCAAGGAGGCCGCTCATATTTCTAGTGTCTGCAAATGCTG
CGTGGAAATACATTAACCGACAACGTACAGAAGCTCCAAGCATACCTGCAGAGACCTATAGTAACAGCACTGTGACGAATA
TAAACACGACTTGACAACAGGGGATCATGTTTCCTCCCTGCAGTCTGACCCATCTGCTGAAAATCTTGCATGTACTGTACA
TTTCAGAGGAAAATGCTGAGAAACTGAGTGTAATATTAGCAGGGTTCTACATACAGTTGTCCATGAAGTGTTTTCGCTGTT
GGCTTTTGCTGCTTTAGATTCATATTGAGTGGATATGTTGTGCCTGGGTAGCGAGGTGTCGAATATAATAACCCTTGCAGG
CATTCTGTTTTATGTGACTTGACTACAGCAACTGAGAGAAACTGAAATGGTGAAAGACGTTAGATGAAGCAAATACAATTG
TGTTTGCTCAAGGTTAAGGGGTTATGGGAAGATTTTTTTTTTTTTTTAAAGGAGAAATCTCACACACACAAAGAGTCTATTG
CACATTAACTGTATTATACACTGACCACATCTGGGAGCTCCTCTCGCTCGCCTTGATCTTCGGTTTCACTCTCAATAGCCA
GTCTTCCTCAGGGATAGGAGGGCTGGGCATATTGTAAAGAGGATGGTCAAACAATGCCTCCAGCTTTGACAGCCCAGACTC
CACAGATTCTGCTCGGTCGGTGCCATAGCGACGCACATCACCCGGCATGTTCATTGAGGCATTTTCACCCTCTGTCGCTGA
TGAGAACCCATCAGAAAGTGCCCGCACGTGGCCTTTGGCATCCTTATTGGCATCCTTATTGGAAGAATCTCTATGTGTGGC
ATCAGTTGGACCACTCAGCCCCACAGAGGTGTAGTTGGCGCGTGCCAGGTCTCGCAGGATGTGTGTCCTCGGTGACAGCAG
GGGGTCACAGGGCTGGTGGTAAATGGGAAGAGAGACAAGCGCCAGCAGGAGGTAGAGGAAAACGGTGATGAAGGCCAAGGA
CAGACAGATTGTCCTGGAGCGACTGCGCATACGCCACATCATCCTACTGAGAGGAGCTGAGAGAGGGTTAGGAGAGGTGAA
CACATGGAGGGAAGAAGCGAGGAAGAAAATTCGGTGCAAAAGATGCTCGGAGAATATGAGGAAAAGCCATATGGTGTAGAG
CGTTAACAACTAATTAAAGTCAGAGCATCACACAGTCCGTATATGATAAAAGGCTTTTTAAAAAGTCCATATCAGCCTACT
TTTACTTTCTGACCTTCCTGAAGTCAGTTTACATAAAGAGAAAAACAAAACTCAACTTACACATAGTTTCCTTCCTTTAAA
AATCCCTCTTGTCAACTCACTGCTGTTGCTGGTCCTCAGTGGAATCAGACGGGACTCTATCCAGTATCAAGGCAGCTTTAC
TCGGGTTGTTTGCACTTTTCTGTGCTCCTGCTACCAGACTCAGAGGTTGGTGCCAGGTTAAAGTGATCTGGATGTGGATCA
ATGTGTTTACTCTCTCTCTCTCTCTTTTTTTTTTAGTCGGTCAGACTTGTTCTGCCAGTTTATTTGTCTTGTCTGTCCTTTT
GTCATTGCGTTCATCAGTTAGACCTTTGAAAAATTCTACTTCAGGGTGTCACTTCCACCTCTTCATTTCCACAGCACGGAT
TGATCTTATTATACAAAGTCTCGGAAAGTTACTTCACAAAAGAGTGTAACTTTTTTTTTTTTTTTTTAAACCACTATATGTG
AACGTGCCTTTAATGCTTAACAGGCAGTGTTGGAAGGGCAAACCTCCTGATCATTCATGACAGCCAGGACTGTCCAGTCAG
GAGCTACTTCAGTACACACACACACACACTTGAACATGCACTGCAGATGCAGGATGATCTGAATTAATACTGATAGGTCGA
CATTTTAAATATGATTTCAAAGGAACATGAAAAGAAAATCAAGATAAAAATGTACATATATTCATGCTATGAAAAAAGTAT
TACTCCTGAACTTAAATTCAATATTTTCTGAACTTAGTTCTGCATCTGCCTCTTTTGTGCAGGAAATCACTTTTACACAAC
TGACCTTTAGCCAAACCTTTACTTTTTGTTGCACTGCTGACGACAATGTAGAGATGTAGAATCTGTTACCAGAGACACATC
CTTACTCACTCACAGACTTAGTACAGAAATCCATCAGACAGACAGGTTTTACACCATTCTGTGACCTTTATTGACATCAAT
CTGCAGCTTGTATGAGGTACAGTACCTGTGGTTGTTGCTGAAAACACCCGTCCGCACACACCAAATGGCAGCTTCGTCTCT
TGTCACATTAAATATTAAAGTGGCCGATGTTAGCTGACATGCCAGTTTTGGGAGTTGCTGGCAAAAAAAAACCTAGCCAGC
CATTTTGATATGAGGTTGTGTTGTTCCTGAAATATGTTGTTTCAAAGGTAAATCCTTTACCACAGTGGCTTTAATAGTTGT
TCCTGTAGATCTAACACCATACTGCAAGAGAAATAAGAGCAGAGAGGAAGATGTATTCTGTGTGCTGCCAGAAAATGTATG
TGTAAATATGCTCTATGTACATAAACAGTGAATTATGTGCAGTCTGTTCAAGGCTTCAACAATGTGGACTTAATAACCAGG
CAGTATGAAGGCAGATATTTTATATTCTGTGAGTGTATTTGCTCTGAGACTTTGATTCAGGCACCTCAGACTTCATGGAGA
TTCCCACAGGATACACTGTTCCTGTGAAGTATGGTTCTTTAAAGTCTGGGTGTGTGTGCGTGTATCTACATGTTGTAAGCT
TTATGTGTGTCTCATACGGAGATCTGATAAAGTTTAACAAAATGCATGCCTCATACCCCTATGACTATCTAAATAGTGTAT
TGAATAGAGTATTGACAGTGCAGAGAAAGGGATCTCCTACCTTAAAAATGACTCATCTCCATCAGCTTGATAGAGTGCATT
GAACAGATTTCGTGCAAACCTCCTGTATTCATGCAGGTGGTCATATTCATTTATTATTCACCGGCGCTCATAAAACATTGA
TG
