# Supplementary material for: Exploring Fish Parvalbumins through Allergen Names and Gene Identities
Source: Genes (Basel). 2024 Oct 18;15(10):1337. doi: 10.3390/genes15101337 (PMC11507022; doi:10.3390/genes15101337)
Supplement: Supplementary file 1 [file genes-15-01337-s001.zip › Supplementary File S2.pdf]

Supplementary File S2

Teleost fish Parvalbumin Sequences

This file contains (A, page 1) the sequences from the fish parvalbumins listed in the ALLERGEN NOMENCLATURE database (A.N.d.) mentioned in main text Table 2, with an explanation for their new annotations (gene assignments); (B, page 9) the parvalbumins from Red seabream (*Pagrus major*) and chum salmon (*Oncorhynchus keta*) as determined/polished in the present study; and (C, page 13) other teleost fish parvalbumins, most of which also have been listed in the Dijkstra and Kondo 2022 study (Biology (Basel), 2022, 11(12):1713; https://www.mdpi.com/2079-7737/11/12/1713). The sequences, or in some cases a sequence variant as mentioned, are aligned in Supplementary file 3.

(A) The sequences from the fish parvalbumins listed in the ALLERGEN NOMENCLATURE database mentioned in main text Table 2, with explanations for their gene assignments

***Clupea_harengus* (Atlantic herring)**

All parvalbumin genes in the Atlantic herring genome were determined by Dijkstra and Kondo 2022. Genes for all three molecules listed in the Allergen Nomenclature database (A.N.d.), being pvalb3, pvalb1, and pvalb4, are at perfectly conserved locations (Dijkstra and Kondo 2022) and these molecules carry pvalb3, -1, and -4 characteristic motifs, respectively (Supplementary file 3).

A.N.d. name: Clu_h_1.0101 New name: pvalb3

GenBank accession mentioned in A.N.d.: CAQ72970

MALASLLKGADIDAALKACEAKDSFKHKDFFAKIGLATKSAADLKKAFEIIDQDKSGFIEEEELKLFLQNFKAGARAL
TDAETKAFLKAGDADGDGMIGVDEFAVMIKP

Note: The gray-shaded alanine is a serine in GenBank accession XP_012694365 (which is shown in Supplementary file 3); this may represent allelic variation or a sequencing error.

A.N.d. name: Clu_h_1.0101 New name: pvalb1

GenBank accession mentioned in A.N.d.: CAQ72971

MAFAGLLSDADIAAALGACTAADTFDHKSFFKKVGLSGKSADDVKKPFYIIDQDKSGFIEEEELKLFLQNFKAGARA
LSDKETKAFLAAGDADGDGMIGVDEFAVMVKAR

Note: The gray-shaded proline, which is quite unusual at this position, is an alanine in the sequence encoded by Ensembl dataset Ch_v2.0.2 transcript pvalb4-204 (which is shown in Supplementary file 3); this may represent allelic variation or a sequencing error.

A.N.d. name: Clu_h_1.0101 New name: pvalb4

GenBank accession mentioned in A.N.d.: CAQ72972

MAFAAFLKEADITAALGACKGADSFDHKAFFAKVGLKGKSGDELKKAFEIIDQDKSGFIEEEELKLFLQNFCKGARA
LTDGETKKFLKAGDSDNDGKIGIDEFAALINH

Note: The gray-shaded alanine and asparagine, the latter being quite unusual at this position, are a threonine and a lysine in XP_031435403 (which is shown in Supplementary file 3); this may represent allelic variation or a sequencing error.

***Ctenopharyngodon_idella* (Grass carp)**

The gene is located at the *PVALB4* location (see GenBank Gene ID: 127509828) and the molecule carries pvalb4 characteristic motifs (Supplementary file 3).

A.N.d. name: Cten_i_1.0101 New name: pvalb4

GenBank accession mentioned in A.N.d.: QCY53440 (shown in Supplementary file 3)

MAFAGILNDADIAAALEACKAADSFNHKAFFAKVGLSAKSGDDVKKAFAIIDQDKSGFIEEDELKLFLQNFKADAR
ALTDAETKIFLKAGDSDGDGKIGVDEFAALVKA

Note: The gray-shaded isoleucine is a valine in XP_051744793; this may represent allelic variation or a sequencing error.

***Cyprinus_carpio* (Common carp)**

The genes are located at the *PVALB3* location (see GenBank Gene ID: 109100278) and the *PVALB4* location (see GenBank Gene ID: 109096998). See also Mukerjee et al. 2021

(https://www.mdpi.com/2410-3888/6/4/70). Furthermore, the molecules carry pvalb3 and pvalb4 characteristic motifs, respectively (Supplementary file 3).

A.N.d. name: Cyp_c_1.0101 New name: pvalb3_(Chr.B12)

GenBank accession mentioned in A.N.d.: CAC83658 (shown in Supplementary file 3)

MAFAGILNDADITAALQGCQAADSFDYKSFFAKVGLSAKTPDDIKKAFAVIDQDKSGFIEEDELKLFLQNFSAGAR
ALTDAETKAFLKAGDSDGDGKIGVDEFAALVKA

A.N.d. name: Cyp_c_1.0201 New name: pvalb4_(Chr.B3)

GenBank accession mentioned in A.N.d.: CAC83659 (shown in Supplementary file 3)

MAFAGVLNDADITAALEACKAADSFNHKTFFAKVGLTSKSADDVKKAFAIIDQDKSGFIEEDELKLFLQNFKAGAR
ALTDGETKTFLKAGDSDGDGKIGVDEFTALVKA

***Gadus_morhua* (Atlantic cod)**

All parvalbumin genes in the Atlantic cod genome were determined by Dijkstra and Kondo 2022. The sequences pvalb2.01 (shown in Supplementary file 3) and pvalb2.02, reported by different research groups, are only one amino acid different (highlighted in gray shading) and this may represent allelic variation or sequencing errors. The same is true for the sequences pvalb3.01 (shown in Supplementary file 3) and pvalb3.02. The genes for pvalb2 and pvalb3 are at perfectly conserved locations (Dijkstra and Kondo 2022) and these molecules have respective characteristic motifs (Supplementary file 3).

A.N.d. name: Gad_m_1.0101 New name: pvalb2.01

GenBank accession mentioned in A.N.d.: AAK63086

MAFAGILADADCAAAVKACEAAESFSYKAFFAKCGLSGKSADDIKKAFFVIDQDKSGFIEEDELKLFLQVFKAGARA
LTDAETKAFLKAGDSDGDGAIGVDEWAVLVKA

A.N.d. name: Gad_m_1.0102 New name: pvalb2.02

GenBank accession mentioned in A.N.d.: CAM56785

MAFAGILADADCAAAVKACEAAESFSYKAFFAKCGLSGKSADDIKKAFFVIDQDKSGFIEEDELKLFLQVFKAGARA LTDAETKAFLKAGDSDGDGAIGVEEWAVLVKA

A.N.d. name: Gad_m_1.0201 New name: pvalb3.01

GenBank accession mentioned in A.N.d.: AAK63087

MAFAGILNDADITAALAACKAEGSFDHKAFFTKVGLAAKSPADIKKVFEIIDQDKSDFVEEDELKLFLQNFSAGARA LSDAETKVFLKAGDSDGDGKIGVDEFGAMIKA

A.N.d. name: Gad_m_1.0202 New name: pvalb3.02

GenBank accession mentioned in A.N.d.: CAM56786

MAFAGILNDADITAALAACKAEGSFDHKAFFTKVGLAAKSSADIKKVFEIIDQDKSDFVEEDELKLFLQNFSAGARA LSDAETKVFLKAGDSDGDGKIGVDEFGAMIKA

***Lates_calcarifer* (Barramundi)**

The genes for pvalb3 and pvalb4 are located at the expected genomic position (see GenBank Gene ID: 108880784 and 108880019, respectively) and the molecules have characteristic pvalb3 and pvalb4 motifs, respectively (shown in Supplementary file 3).

A.N.d. name: Lat_c_1.0101 New name: pvalb3

GenBank accession mentioned in A.N.d.: AAV97933

MAFAGILNEADITAALAACQAADSFKHKDFFVKVGLAGKSDDDVKKAFAVIDQDKSGFIEEDELKLFLQNFSASAR ALTDAETKEFLKAGDSDGDGKIGVDEFAALVKV

A.N.d. name: Lat_c_1.0101 New name: pvalb4

GenBank accession mentioned in A.N.d.: AAT45383

MAFSNVLSDSDVAAALDGCKDAGTFDHKKFFSACGLSNKTSDDVKKAFAIIDQDKSGFIEEEELKLFLQNFKADAR VLTDVETSTFLKAGDTDGDGKIGADEFTALVKP

***Lepidorhombus_whiffiagonis* (Whiff)**

The gene encodes a molecule that is very similar to pvalb1 in *Scophthalmus maximus* (turbot) (see their alignment in Supplementary file 3), the gene of which is at the expected *PVALB1* location (GenBank Gene ID: 118289318). The molecules have characteristic pvalb1 motifs (Supplementary file 3).

A.N.d. name: Lep_w_1.0101 New name: pvalb1

GenBank accession mentioned in A.N.d.: CAP17694

MTFAGLDAAEIKAALDGCAAADSFDYKKFFGACGLAKKSAEEVKAAFNKIDQDESGFIEEDELKLFLQNFSASARA
LTDKETANFLKAGDVDGDGKIGIEEFTDLVRSK

***Pangasianodon_hypophthalmus* (Striped catfish)**

The genes are located at the *PVALB4* location (see GenBank Gene ID: 113527990) and the *PVALB7* location (see GenBank Gene ID: 113547569), respectively. They are very similar to their pvalb4 and pvalb7 counterparts in channel catfish (see their alignment in Supplementary file 3), which were analyzed in detail by Dijkstra and Kondo 2022. Furthermore, the molecules carry pvalb4 and pvalb7 characteristic motifs, respectively (Supplementary file 3).

A.N.d. name: Pan_h_1.0101 New name: pvalb4

GenBank accession mentioned in A.N.d.: XP_026772003

MAFAGVLNDADIAAALDACKADGSFDHKAFFTKVGLTGKSADDVKKAFSIIDQDKSGFIEEDELKLFLQNFKSDAR
ALTDNETKIFLKAGDTDGDGKIGVDEFASLVKA

A.N.d. name: Pan_h_1.0101 New name: pvalb7

GenBank accession mentioned in A.N.d.: XP_026803769

MAMQDLLKADDIKKALDTFKVADTFDHKKFFELVGLKAMSAENVKKVFSVLDVDASGFIEEDELKFVLKGFSKDG
RDLTDKETKAFLTAADRDGDGKIGIDEFEAIVHQ

Rastrelliger_kanagurta **(Indian mackerel)**

The sequence retrieved as topmatch from GenBank is pvalb4 of *Scomber scombrus* (Atlantic mackerel) (see below), directly followed by pvalb4 of *Scomber japonicus* (Pacific mackerel), for which the *PVALB4*-typical gene position can be seen at GenBank Gene ID: 128378285. The three sequences have pvalb4 characteristic motifs (see Supplementary file 3).

A.N.d. name: Ras_k_1.0101 New name: pvalb4

GenBank accession mentioned in A.N.d.: ANW10058

MAFASVLKDAEITAALDGCKADGSFDHKKFFKACGLAGKSGDDVKKAFAIIDQDKSGFIEEEELKLFLQNFKAGAR
TLSDAETKAFLKAGDTDGDGKIGVDEFAAMIKG

Salmo_salar **(Atlantic)**

The gene is located at the *PVALB4* location (see GenBank Gene ID: 106601823). See also Mukerjee et al. 2021 (https://www.mdpi.com/2410-3888/6/4/70) and the analysis of pvalb4 of the related *Esox lucius* (Northern pike) in Dijkstra and Kondo 2022. Furthermore, the molecule carries pvalb4 characteristic motifs (Supplementary file 3).

A.N.d. name: Sal_s_1.0101 New name: pvalb4_(Chr.3)

GenBank accession mentioned in A.N.d.: CAA66403_[]

MACAHLCKEADIKTALEACKAADTFSFKTFFHTIGFASKSADDVKKAFKVIDQDASGFIEVEELKLFLQNFCPKARE
LTDAETKAFLKAGDADGDGMIGIDEFAVLVKQ

Note: The gray-shaded serine is an asparagine in XP_014049696; this may represent allelic variation or a sequencing error.

Sardinops_sagax **(Pacific pilchard)**

The gene encodes a molecule that is very similar to pvalb4 in *Sardinops melanostictus* (Japanese sardine), followed as GenBank topmacth by pvalb4 of *Alosa sapidissima* (American shad), the gene

of which is situated at a typical *PVALB4* location (GenBank Gene ID: 121705096). The molecules have motifs characteristic for pvalb4 (Supplementary file 3).

A.N.d. name: Sar_sa_1.0101 New name: pvalb4

GenBank accession mentioned in A.N.d.: CAQ68366

MALAGLVKEADITAALEACKAADSFDHKAFFHKVGMSGKSADELKKAFAIIDQDKSGFIEEEELKLFLQNFCKKAR ALTDGETKKFLKAGDNVGDGKIGIDEFNHLVKH

Note: The gray-shaded valine is an aspartic acid in other investigated parvalbumins (Supplementary file 3), and caution regarding the reliability of the sequence seems to be justified.

***Scomber_scombrus* (Atlantic mackerel)**

The sequence retrieved as topmatch from GenBank is *Scomber japonicus* pvalb4, for which the *PVALB4*-typical gene position can be seen at GenBank Gene ID: 128378285. The sequences have pvalb4 characteristic motifs (see Supplementary file 3).

A.N.d. name: Sar_sa_1.0101 New name: pvalb4

GenBank accession mentioned in A.N.d.: CAX32965

MAFASVLKDAEITAALDGCKAAGSFDHKKFFKACGLSGKSADEVKKAFAIIDQDKSGYIEEEELKLFLQNFKAGARA LSDAETKAFLKAGDSDGDGKIGVDEFAAMIKG

***Sebastes_marinus* (Ocean perch)**

The pvalb3 molecule is very similar to pvalb3 in *Sebastes umbrosus* (honeycomb rockfish) (see their alignment in Supplementary file 3), the gene of which is situated at a typical *PVALB3* location (GenBank Gene ID: 118289318). The pvalb4 molecule is very similar to pvalb4 in *Sebastes umbrosus* (honeycomb rockfish) (see their alignment in Supplementary file 3), the gene of which is situated at a typical *PVALB4* location (GenBank Gene ID: 119501528). The sequences also have motifs characteristic for pvalb3 and pvalb4, respectively (Supplementary file 4).

A.N.d. name: Seb_m_1.0101 New name: pvalb3

GenBank accession mentioned in A.N.d.: CAQ72968

MALAASLNAADITAALAACSGVDTFKHKDFFGKVGLSAKSADDIKNAFKVIDQDKSGFIEEEELKLFLQNFSATAR
ALTEAETTAFLKAGDSDGDGMIGMDEFAAMVKG

A.N.d. name: Seb_m_1.0201 New name: pvalb4

GenBank accession mentioned in A.N.d.: CAQ72969

MAFASVGLKDADIAAALDGCKDAGKFNHKTFFKTCGLSGKSSDEVKKAFAIIDQDISGFIEEEELKLFLQTFKAGAR
ALSDAETKEFLKAGDSDGDGKIGADEWAAMVKQ

_*Thunnus_albacares*_ (Yellowfin tuna)

The pvalb3 molecule is very similar to pvalb3 in *Thunnus maccoyii* (bluefin tuna) (see their alignment in Supplementary file 3), the gene of which is situated at a typical *PVALB3* location (GenBank Gene ID: 121886871). The sequences also have motifs characteristic for pvalb3 (Supplementary file 4).

A.N.d. name: Thu_a_1.0101 New name: pvalb3

GenBank accession mentioned in A.N.d.: CAQ72967

MAFAGILTEADITAALAACQAADSFKYKDFFTKVGLAAKTPEDIKKAFAVIDQDKSGFIEEDELKLFLQNFSAGARA
LTDAETKAFLMAGDSDGDGKIGIDEFAALVKA

_*Xiphias_gladius*_ (Swordfish)

The gene is located at the *PVALB4* location (GenBank Gene ID: 120788431), and the molecule has motifs characteristic for pvalb4 (Supplementary file 4).

A.N.d. name: Xip_g_1.0101 New name: pvalb4

GenBank accession mentioned in A.N.d.: CAR48256

MAFAGVLSDADVAAALEACKDAGTFDYKKFFKSCGLAAKSTDDVKKAFAIIDQDKSGFIEEDELKLFLQNFKAAAR
PLTDAETEAFLKAGDSDGDGKIGAEEFAALVTA

(B) The parvalbumins from Red seabream (*Pagrus major*) and chum salmon (*Oncorhynchus keta*) as determined/polished in the present study

Pagrus major **(Red seabream) parvalbumins**

The below sequences are predicted from the genomic scaffold sequences shown in Supplementary file 1.

Pagrus major **(Red seabream) pvalb1**

MAFSGVLSDADMKAALDGCSAADSFDYKKFFKACGLSGKSADEVKKAFAIIDQDNSGFIEEEELKLFLQNFGKSA
RALTDKETKAFLAAGDSDGDGKIGVDEFAALVKA

Pagrus major **(Red seabream) pvalb3**

MAFGGILKDADITAALAACQAADSFKHKDFFAKVGLAAKSADEIKKAFAVIDQDKSGFIEEDELKLFLQNFSASARA
LTDAETKTFLKAGDSDGDGKIGVDEFAAMMKA

Pagrus major **(Red seabream) pvalb4**

MPFKGLKDADVAKALEGCKDAGTFDHKKFFHACGLAGKSGADVKEAFFVIDQDKSGFIEEEELKLFLQNFKAGAR
ALTDDETKKFLKAGDSDGDGKIGADEFAEMVKV

Pagrus major **(Red seabream) pvalb5**

MAFAGMLSDEDIKAAVQACQTPGTFDFKSFFAQVGLTGSSEADGKKVFTILDQDRSGYIEEEELKLFLQNFSPGAR
ELTVAETKALMAAGDKDGDGKIGLEEFCDMLK

Pagrus major **(Red seabream) pvalb6**

MAMSSILNADNIKKALDAFAAADSFEYNKFFDMVGLKAKSSDDVKKVFKVLDADNSGFIEEEELKFVLKGFAKDG
RDLTDKETKAFLQAADKDGDGKIGVDEFVSLVKE

Pagrus major **(Red seabream) pvalb7**

MSMTDLLKAEEIKKALDAFAAETFDPKKFFEMVGMKAMTAENVKKVFKVLDVDGSGFIEEEELKYVLKGFSQEG
RDLTDDETRAFLKAADKDGDGKIGIDEFEVLVHE

***Pagrus major* (Red seabream) pvalb8**

MSLSSILSADAIDSAIKDCQAPDSFCPKKFFQACGLAQKSPQDVKKAFGILDNDGSGFIEEEELKFFLQRFSPGARV
LTDKETKGFLAAADDDSDGRIGAEEFQAMVLS

***Pagrus major* (Red seabream) pvalb9**

MSLTSILSAEAIENAVKDCQAPDTFCYKKFFELCGLSSKSPKEIKDVFQILDEDNSGFIEESELKYFLQRFIPGARTLTE
AETKRFISAADDDSDGRIGAEEFQTMVLS

***Pagrus major* (Red seabream) pvalb10**

MAITDFLAASDITSAINACKAKDSFCPKMFFKTVGLSKKTPTEIEGVFKILDQDKSGFIEQDELQLFLQNFSKGARTL
TAAETRAFLLEGDSDGDGKIGWEEFSALVKSS

***Oncorhynchus keta* (chum salmon) parvalbumins**

The below-listed chum salmon parvalbumin sequences are automatically predicted by GenBank from genomic DNA and agree with our own predictions. The absence of sequencing errors and the correct assembly of exons was confirmed by overlapping RNA-seq reads, encoding identical sequences, that were retrieved from the Single Reads Archive (SRA) of GenBank by tBlastn similarity searches.

***Oncorhynchus keta* (chum salmon) pvalb2A_(Chr.2)**

Sequence: GenBank XP_035614887; confirmed by SRAs of datasets SRX6595884 (SRR9841173.78012718.2) and SRX6595887 (SRR9841174.6150073.2 and SRR9841174.47569122.1)

MSFAGLNDADVAAALAACAAADSFNHKAFFAKVGLASKSNDDVKKAFYVIDQDKSGFIEEDELKLFLQNFSASAR
ALTDAETKAFLADGDKDGDGMIGVDEFAAMIKG

***Oncorhynchus keta* (chum salmon) pvalb2A_(Chr.24)**

Sequence: GenBank XP_035657206; confirmed by SRAs of datasets SRX6595884 (SRR9841173.43785054.1, SRR9841173.76718842.1, and SRR9841173.2553632.2)

MSFAGLNDADVAAALAACTAADSFNHKAFFAKVGLAGKSNDDVKKAFYVIDQDKSGFIEEDELKLFLQNFSASAR
ALTDAETKAFLADGDKDGDGMIGVDEFAAMIKG

Oncorhynchus keta **(chum salmon) pvalb2B_(Chr.2)**

Sequence: GenBank XP_035614863; confirmed by SRAs of datasets SRX6595895 (SRR9841163.7903812.2, SRR9841163.50212082.1) and SRX6595880 (SRR9841178.13723931.2)

MAFKGMLKDEDIAAALKHSAAAESFNHKEFFAKVGLTGKSAEDLKKAFYFVDQDKSGFIEEDELKLFLQTFSAGAR
ALTEKETKAFLAAGDVDGDGMIGVDEFVTLVNA

Oncorhynchus keta **(chum salmon) pvalb2B_(Chr.24)**

Sequence: GenBank XP_035657205; confirmed by SRAs of datasets SRX6595880 (SRR9841178.24742941.2, SRR9841178.64289939.2, and SRR9841178.59031046.2)

MAFKGMLKDEDIAAALQHCAAADSFNHKEFFAKVGLAGKSTEDLKKAFYFVDQDKSGFIEEDELKLFLQTFSAGA
RALTDKETKVFLAAGDADGDGMIGVDEFTTLVKV

Oncorhynchus keta **(chum salmon) pvalb4**

Sequence: GenBank XP_035614493; confirmed by SRAs of datasets SRX6595880 (SRR9841178.64473650.2, SRR9841178.64172284.2, and SRR9841178.62612532.2)

MACAHLCKEADIKAALEACKGADTFNFKTFFHTIGFASKSADDVKKAFKVIDQDASGFIEEEELKLFLQNFCPKARV
LTDAETKAFLKAGDADGDGMIGIDEFAVLVKQ

Oncorhynchus keta **(chum salmon) pvalb5**

Sequence: GenBank XP_035614494; confirmed by SRAs of dataset SRX6595880 (SRR9841178.11737964.2, SRR9841178.40292990.1, and SRR9841178.6240319.2)

MAFTGLLREEDIRAAVKACQAPGTFNFKLFFAQVGLTGKSEAEGQRVFRVLDQDQSGYIEEEELKLFLQNFSSGAR
ELTDAETRTLLAAGDRDGDGKIGMEEFCALLK

Oncorhynchus keta **(chum salmon) pvalb6_(Chr.5)**

Sequence: GenBank XP_052374525; confirmed by SRAs of dataset SRX6595880 (SRR9841178.60355684.1, SRR9841178.49983093.2, and SRR9841178.9397766.2)

MAMNSILNAADIKKALDAFAAADSFDHKKFFEMVGLKAKSAEDVKKAFLVLDADASGFIEEEELKFVLKGFASDG RDLTDKETKAFLNEADKDGDGMIGIDEFVALVQE

***Oncorhynchus keta* (chum salmon) pvalb6_(Chr.32)**

Sequence: GenBank XP_035603006; confirmed by SRAs of dataset SRX6595880 (SRR9841178.3326393.2, SRR9841178.31821333.2, and SRR9841178.61443176.2)

MAMNSILNAADIKKALEAFAAADSFDHKKFFEMVGLKAKSAEDVKKAFLVLDADASGFIEEEELKFVLKGFASDG RDLTDKETKAFLNEADKDGDGMIGIDEFVALVHE

***Oncorhynchus keta* (chum salmon) pvalb7**

Sequence: GenBank XP_052373800; confirmed by SRAs of dataset SRX6595880 (SRR9841178.20865393.2, SRR9841178.30241020.2, and SRR9841178.57901022.1)

MAALKDFLKADDIQKALDAVKAEGSFDHKKFFALVGLKAMTPDNVKKVFQAIDADQSGFIEEEELKFVLKSFAED GRDLTDAETRAFLNAADKDGDGKIGINEFEVLVHEV

***Oncorhynchus keta* (chum salmon) pvalb8**

Sequence: GenBank XP_052367866; confirmed by SRAs of dataset (SRR9841163.14154470.1 , SRR9841163.58337078.1, and SRR9841163.79600125.1)

MSLSSILSADAIDAAMKDCQAPDSFNCKKFFQQCGLTKKSPAEVKKVFGILDNDASGFIEEEELKFFLQRFNPGAR VLTEKETKAFMSAADDDSDGMIGADEFQAMVLS

***Oncorhynchus keta* (chum salmon) pvalb9_(Chr.2)**

Sequence: GenBank XP_052328035; confirmed by SRAs of dataset SRX6595895 (SRR9841163.79962096.2, SRR9841163.65649252.1, and SRR9841163.80379295.1)

MSLTSILSVEDIENAVKEFQAPDSFSFKKFFQLCGLTSKSPKEVKDVFQILDDDNSGYIEESELKFFLQRFVPGARTLT DAECKGFLSAADDDNDGKIGVEEFLIMVQS

***Oncorhynchus keta* (chum salmon) pvalb9_(Chr.24)**

Sequence: GenBank XP_035657203; confirmed by SRAs of dataset SRX6595895 (SRR9841163.53771327.2, SRR9841163.53195052.1, and SRR9841163.80379295.1)

MSLNSILSAEAIENAVKECQAPDSFSFKKFSQLCGLTSKSPKEVKDVFQILDEDNSGFIEESELKFFLQRFVPGARTLT
DAECKGFLSAADDDNDGKIGVEEFLIMVQS

Oncorhynchus keta **(chum salmon) pvalb10**

Sequence: GenBank XP_035657215; confirmed by SRAs of dataset SRX6595880 (SRR9841178.16574873.2, SRR9841178.30638325.1, and SRR9841178.44360359.2)

MALTDFLAASDITSAINACRANDSFSPKKFFAMVGLSKKSPPEIEKIFMILDQDKSGYIEQDELQLFLQNFSKGARPL
TAAETRAFLLAGDKDGDGKIGWDEFSNLVMSS

(C) Other teleost fish parvalbumins

Alosa sapidissima (American shad)

Alosa_sapidissima_**(American shad)_pvalb4**
Sequence: GenBank XP_048101815
MALAGMVKEADVTAALSACKAADSFDHKTFFGKVGLKAKSADELKKAFAIIDQDKSGFIEEEELKLFLQNFCK
GARALTDGETKTFLKAGDSDGDGKIGIDEFSALVKA

Anguilla anguilla (European eel)

Anguilla_anguilla_(**European_eel)_pvalb1**
Sequence: GenBank XP_035253170
MAFAGVLKDADITAALAACQGADSFDYKSFFAKVGLSGKTPDEIKKAFSIIDQDNSGFIEEDELKLFLQNFSA
GARALTDKETKTFLAAGDTDGDGKIGIDEFAALVKA

Anguilla_anguilla_(**European_eel)_pvalb3**
Sequence: GenBank XP_035258683
MAFAGVLNDADITAALQACQAADSFNYKSFFAKVGISGKSADIVKKVFAILDQDKSGFIEEDELKLFLQNFSG
SARALTDAETSAFLKAGDTDGDGKIGIDEFSALVKA

Anguilla_anguilla_(**European_eel)_pvalb4**
Sequence: GenBank XP_035253204
MAFAGVLKDADITAALEACKAADSFNYKAFFAKVGLATKSADDIKKAFNIIDQDKSGFIEEDELKLFLQNFSK
TARALTDKETKAFLHAGDTDGDGKIGIDEFAAVVKA

Anguilla_anguilla_(**European_eel)_pvalb5**
Sequence: GenBank XP_035253199
MSFSGILKDEDIKAAVLACEAPGTFDYKLFFTQVGLLSQQDVEGKTVFNVLDRDQSGFIEEAELKFFLQNFCL
GARELTEAETKAFISAVDRDGDGKIGVEEFCTLLK

Anguilla_anguilla_**(European_eel)_pvalb6**
Sequence: GenBank XP_035253026
MAMKDILKAEDIKKAMDACKVADTFDHKKFFEMVGLKTRSTDDLKKAFLALDVDNSGFIEEEELKFFLKGFAT
DGRDLTDKETKAFLRAADKDGDGKIGMEEFAAMVRE

Anguilla_anguilla_**(European_eel)_pvalb7**
Sequence: GenBank XP_035261400
MAMKDILNADEIKKALDAFKAAESFNHMKFFEMIGLKAKSAEDVKKVFKVLDVDNSGFIEEEELKFVLKGFAS
DGRDLTDKETKAFLEAADRDGDGKIGVDEFEALVHE

Anguilla_anguilla_**(European_eel)_pvalb8**
Sequence: GenBank XP_035253329
MSLTSILSAGAIDSAIKDCSAPDSFCSKKFFQICGLTKKSPQEVKEVFRVLDNDDSGYIEEEELKFFLQRFSS
GARVLTEKETKAFLVAADGDSDGKIGVQEFQKLVLS

Anguilla_anguilla_**(European_eel)_pvalb9**
Sequence: GenBank XP_035258685
MSLTSILAADAIENAIKDCQAPDSFNSKKFFQLSGLTKKSPQEVKDVFCILDNDGSGFIEEEELKFFLQRFSP
GARLLTDKETKSFLSDADDDSDGKIGAEEFQAMVLS

Anguilla_anguilla_**(European_eel)_pvalb10**
Sequence: GenBank XP_035259276
MAMTDLLTMSDITSAITACQDADSFNLKAFFRDVGLSKKSPLDIEKVFKILDQDKSGFIEHDELQLFLQNFSK
GARSLTAAETKAFLQAGDMDGDGKIGLEEFSALVKAS

Anguilla_anguilla_**(European_eel)_pvalb-like**
Sequence: GenBank XP_035261997
MADDFSLQVKKVTLAMGASLSDQDIDRIPREMRMQGNFNYSKFFEYMRQYKTSDQQEEVIKKAFQVLDKDGSG
YIEWNEIKYILSTVPSSVPIVPLSDEEAEAVIQAADADGDGRIDFREFSDLVKLEKKPRK

Danio rerio (Zebrafish)

Danio_rerio_**(zebrafish)_pvalb1**
Sequence: GenBank NP_991135
MAVGALLAAADVDAALAACQAADSFDYKSFFAKVGLSAKSADEVKKAFAIIDQDNSGFIEEEELKLFLQNFKA
NARVLTDKETKAFLSAGDSDGDGKIGAEEFAALVKA

Danio_rerio_**(zebrafish)-pvalb2**
Sequence: GenBank NP_571591
MAFAGILKDEDVAAALKDCAAADSFNYKNFFAKVGLSAKSPDDIKKAFFVIDQDKSGFIEEDELKLFLQNFSA
GARALTDAETKAFLSAGDSDGDGKIGVDEFALLVKA

***Danio_rerio*_(zebrafish)_pvalb3**
Sequence: GenBank NP_956506
MAFAGILNEADITAALQACQAADSFDYKSFFAKVGLSAKTPDDIKKAFAVIDQDKSGFIEEDELKLFLQNFSA
GARALTDAETKAFLKAGDSDGDGKIGVDEFASLVKA

***Danio_rerio*_(zebrafish)_pvalb4**
Sequence: GenBank NP_997948
MAFAGVLNDADISAALEACKAADSFNHKSFFAKVGLASKSADEVKKAFAIIDQDKSGFIEEEELKLFLQNFKA
DARALTDGETKTFLKAGDSDGDGKIGIDEFAALVKA

***Danio_rerio*_(zebrafish)_pvalb5**
Sequence: GenBank NP_997050
MALAGILKQDDIAAAMQACQAQGSFNHESFFEQVGLIGRASSDGEKVFKALDQDKSGYIEKEELKRFLQNFCS
KARELTEAETNTLLAAGDKDGDGKIGIEEFCALLK

***Danio_rerio*_(zebrafish)_pvalb6**
Sequence: GenBank NP_991136
MAMSSILNHDDIKKALDACKAPDSFNHKSFFEMVGLKAKASDDVKKAFHLLDADNSGFIEEEELKFVLKAFAT
DGRDLTDKETKAFLQAADKDGDGKIGAEEFAALVRE

***Danio_rerio*_(zebrafish)-pvalb7**
Sequence: GenBank NP_991137
MAMKNLLKDDDIKKALDQFKAADSFDHKKFFDVVGLKALSADNVKLVFKALDVDASGFIEEEELKFVLKGFSA
DGRDLTDKETKAFLAAADKDGDGKIGIDEFEALVHE

***Danio_rerio*_(zebrafish)_pvalb8**
Sequence: GenBank NP_891982
MSLTSILSADAIDCALKDCQAPDSFSPKKFFQLCGMTKKSPQDVKNIFNILDNDASGFIEEDELKFFLQRFSG
GARVLTDKETKAFLAAADDDSDGKIGAEEFQAMVLS

***Danio_rerio*_(zebrafish)-pvalb9**
Sequence: GenBank NP_891983
MSLTSILSAEAIENAVKDCQAPDSFCYKKFFQLCGLSQKTPQEVKDVFRIIDEDNSGFIEEAELKFFLQRFFP
GARTLTEKEIKSLLTAADDDSDGRIGVDEFQTMVSS

***Clupea harengus* (Atlantic herring)**

***Clupea_harengus*_(Atlantic_herring)_pvalb1**
Sequence: Encoded by Ensembl dataset Ch_v2.0.2 transcript pvalb4-204
MAFAGLLSDADIAAALGACTAADTFDHKSFFKKVGLSGKSADDVKKAFYIIDQDKSGFIEEEELKLFLQNFKA
GARALSDKETKAFLAAGDADGDGMIGVDEFAVMVKA

***Clupea_harengus*_(Atlantic_herring)_pvalb2A**
Sequence: GenBank XP_012694367
MAFAGILKDADIAAAITACAAADSFNYKTFFAKCGLSAKSADEVKKAFFVIDQDKSGFIEEDELKLFLQTFKA
GARALTDAETKAFLAAGDADGDGMIGVDEFAALVKA

***Clupea_harengus*_(Atlantic_herring)_pvalb2B**

Sequence: GenBank XP_012694369
MAFAGILKDADIAAAIKDCAAADSFNYKTFFVKCGLSAKSSDEVKKAFFVIDQDKSGFIEEDELKFFLQTFKA
GARALTDAETKAFLAAGDEDGDGMIGVDEFAVLVKA

***Clupea_harengus*_(Atlantic_herring)_pvalb3**
Sequence: GenBank XP_012694365
MALASLLKGADIDAALKACEAKDSFKHKDFFAKIGLATKSAADLKKAFEIIDQDKSGFIEEEELKLFLQNFKA
GARALTDAETKAFLKAGDADGDGMIGVDEFSVMIKP

***Clupea_harengus*_(Atlantic_herring)_pvalb4**
Sequence: GenBank XP_031435403
MAFAAFLKEADITAALGACKGADSFDHKAFFAKVGLKGKSGDELKKAFEIIDQDKSGFIEEEELKLFLQNFCK
GARALTDGETKKFLKAGDSDNDGKIGIDEFTALIKH

***Clupea_harengus*_(Atlantic_herring)_pvalb5**
Sequence: GenBank XP_031426141
MAITGMLKEDDIRNAIEACQGPDTFDFKSFFKLAGLCSRPQADREKVFGVVDQDQSGFIEEAELKFFLQNFLL
GARELTEAETKSFLLAADCDGDGKIGMEEFCSLMN

***Clupea_harengus*_(Atlantic_herring)_pvalb6**
Sequence: GenBank XP_031416779
MTMDSILSADDIKKALDSFKAAESFDHNKFFEMVGLKAKSDEEIKKAFLVLDADNSGFIEEEELKFVLKCFAP
DGRDLTDKETKAFLNAADKDGDGKIGVDEFTALVHE

***Clupea_harengus*_(Atlantic_herring)_pvalb7**
Sequence: GenBank XP_031419732
MAMKDLLTEENIKKAMDAFKAADSFDYKKFFDMVGLKAMSADSVKQVFKLLDVDESGYIEEEELKFVLKGFSS
EGRDLTDKETQTFLSAADKDGDGKIGIDEFEALVHE

***Clupea_harengus*_(Atlantic_herring)_pvalb8**
Sequence: GenBank XP_012681239
MSISSILSADAIDSAVKDCQAPDSFNCKKFFQLCGLTKKSAQDVKNVFSIIDNDGDGFVEEKELKCFLQKFSP
GARVLTEKEAKAFLSAADGDGDGKIGADEFQAMVMS

***Clupea_harengus*_(Atlantic_herring)_pvalb9**
Sequence: GenBank XP_012694346
MSLTSILSAEAIDNAIKDCQDPDTFCYKKFFQLCGLSQKTPQEVKDVFRILDDDDSGFIEESELKFFLQQFVP
TARVLTEKEAKSFMAAADKDSDGKIGVDEFQSMILS

***Clupea_harengus*_(Atlantic_herring)_pvalb10**
Sequence: Modification of GenBank XP_012694332
MAVKDLFAASDITSAISSCQANDSFSPKAFFATVGLSKKSPPEIEKVFRILDQDKSGFIEQEELQLFLQNFSK
GARTLTAAETRAFMLDGDMDGDGKIGWEEFSALVKSS

Coryphaenoides rupestris **(roundnose grenadier)**

***Coryphaenoides_rupestris*_(Roundnose_grenadier)_pvalb1_(partial,_maybe_pseudogene)**
Sequence: Encoded by GenBank genomic sequence PIJE02000705
MAFEGLAEADVKAALAGCTAADSFNYKTFFAACGLAKKSDADVKKAFFVIDQDQSGFIEEDEL

***Coryphaenoides_rupestris_*(Roundnose_grenadier)_pvalb4**
Sequence: Encoded by GenBank genomic sequence PIJE02000705
MALRGILKDADVTAALDACKVAGSFDHKKFFSSCGLSSKSSADVKKAFGIIDQDKSDFIEEDELKLFLQNFSA
GARALSDAETKVFLQAGDTDGDGKIGVDEFAAMVKA

***Coryphaenoides_rupestris_*(Roundnose_grenadier)_pvalb5**
Sequence: Encoded by GenBank genomic sequence PIJE02000705
MTFTEMLKDEDVKAAVQACQAPGTFDHKAFFEQVGLSGSAEDMAERVFTALDQDKSGYIEEEELKTSSPGARV
LTVAETKALLAAGDKDGDGKIGMAEFCALLK

C*oryphaenoides_rupestris_*(Roundnose_grenadier)_pvalb8_(maybe_pseudogene)
Sequence: Encoded by GenBank genomic sequence PIJE02000705
MSLSSILSADAIDNAIKDCQAPRSSLKLCGLSKKSAADVKKAFSIMDNDASGFIEEEELKFFLQRFSPGARVL
TASETKAFLNAADDDSDGKIGADEFQSLVLS

***Ctenopharyngodon Idella* (grass carp)**

Ctenopharyngodon_idella_(grass_carp)_pvalb4
Sequence: GenBank QCY53440
MAFAGILNDADIAAALEACKAADSFNHKAFFAKVGLSAKSGDDVKKAFAIIDQDKSGFIEEDELKLFLQNFKA
DARALTDAETKIFLKAGDSDGDGKIGVDEFAALVKA

***Cyprinus carpio* (common carp)**

***Cyprinus_carpio_*(common _carp)_cyp_c 1.01_pvalb3_(Chr.B12)**
Sequence: GenBank XP_018969314
MAFAGILNDADITAALQGCQAADSFDYKSFFAKVGLSAKTPDDIKKAFAVIDQDKSGFIEEDELKLFLQNFSA
GARALTDAETKAFLKAGDSDGDGKIGVDEFAALVKA

***Cyprinus_carpio_*(common carp)_pvalb4(Chr.A3)**
Sequence: GenBank XP_018966256
MAFAGVLNDADIAAALEACKAADSFNHKAFFAKVGLTSKSADDVKKAFAIIDQDKSGFIEEDELKLFLQNFKA
DARALTDGETKTFLKAGDSDGDGKIGVDEFTALVKA

***Cyprinus_carpio_*(common carp)_pvalb4(Chr.B3)**
Sequence: GenBank XP_018966236
MAFAGVLNDADITAALEACKAADSFNHKTFFAKVGLTSKSADDVKKAFAIIDQDKSGFIEEDELKLFLQNFKA
GARALTDGETKTFLKAGDSDGDGKIGVDEFTALVKA

***Esox lucius* (Northern pike)**

***Esox_lucius_*(Northern_pike)_pvalb2A**
Sequence: Encoded by Ensembl dataset Eluc_v4 transcript pvalb2-204
MSFAGLKDADVAAALAACSAADSFKHKEFFAKVGLASKSLDDVKKAFYVIDQDKSGFIEEDELKLFLQNFSPS
ARALTDAETKAFLADGDKDGDGMIGVDEFAAMIKA

***Esox_lucius_*(Northern_pike)_pvalb2B**
Sequence: Encoded by Ensembl dataset Eluc_v4 transcript pvalb2-203

MAFKGMLKDEDVAAALQAFAAADSFNHKQFFAKVGLAGKSDEDLKKAFYLVDQDKSGFIEEEELKLFLQTFSA
GARALTDTETKAFLAAGDVDGDGMIGVDEFVTLVKA

***Esox_lucius*_(Northern_pike)_pvalb4**
Sequence: Encoded by Ensembl dataset Eluc_v4 transcript pvalb4-201
MACAHLCKEADIKTALEACKAPESFNFTAFFKAVGFASKTADEVKKAFQVIDQDASGFIEEEELKLFLQNFCP
NARTLTDAETKAFLKAGDVDGDGMIGIDEFAELVKQ

***Esox_lucius*_(Northern_pike)_pvalb6**
Sequence: Encoded by Ensembl dataset Eluc_v4 transcript pvalb6-201
MAMSSILNAADIKKALDAFAAEGSFDHKKFFEMVGLKAKSAEDVKKAFLVLDADASGFIEEEELKFVLKGFAS
DGRDLTDKETKAFLNEADKDGDGMIGIDEFVALVHE

***Esox_lucius*_(Northern_pike)_pvalb7**
Sequence: Encoded by Ensembl dataset Eluc_v4 transcript pvalb7-202
MAAKDLLKADDIKKALDAVKAEGSFDHKKFFALVGLKAMSADNVKKVFKAIDADASGFIEEEELKFVLKSFAA
DGRDLTDAETKAFLKAADKDGDGKIGIDEFETLVHEA

***Esox_lucius*_(Northern_pike)_pvalb8**
Sequence: Encoded by Ensembl dataset Eluc_v4 transcript pvalb8-201
MSLSSILCTDAIETALKECLAPDSFNCRKFFQQCGLSKKSPAEVKTVFKILDNDNSGYIEEEELKYILQRFSL
GARVLTDKETKAFMSACDDDSDGMIGADEFQAMVLS

***Esox_lucius*_(Northern_pike)_pvalb9**
Sequence: Encoded by Ensembl dataset Eluc_v4 transcript pvalb9-201
MSLTSILSAEAIENAVKEFQAPESFSFKKFFQLCGLSSKSPKEVKDVFQILDSDNSGYIEEAELKFFLQRFVP
GARTLTDTECKGFLSAADDDNDGKIGVEEFLTMVQS

Gadus morhua (Atlantic cod)

***Gadus_morhua*_(Atlantic_cod)_pvalb1**
Sequence: GenBank XP_030204978
MSFAGVLADADVKAALAGCAAADSFSYKTFFKACGLAAKSHEEVKKAFFVIDQDQSGFIEEDELKLFLQTFGA
GARELTAAETKAFLAAGDVDGDGMIGVDEFVTLVKA

***Gadus_morhua*_(Atlantic_cod)_pvalb2**
Sequence: GenBank XP_030196388
MAFAGILADADCAAAVKACEAAESFSYKAFFAKCGLSGKSADDIKKAFFVIDQDKSGFIEEDELKLFLQVFKA
GARALTDAETKAFLKAGDSDGDGAIGVDEWAVLVKA

***Gadus_morhua*_(Atlantic_cod)_pvalb3**
Sequence: GenBank XP_030196389
MAFAGILNDADITAALAACKAEGSFDHKAFFTKVGLAAKSPADIKKVFEIIDQDKSDFVEEDELKLFLQNFSA
GARALSDAETKVFLKAGDSDGDGKIGVDEFGAMIKA

***Gadus_morhua*_(Atlantic_cod)_pvalb4**
Sequence: GenBank XP_030205636
MAFAGILKDAEVAAALEACKSAGSFDHTKFFKSCGLAGKSSDDVKKAFGIIDQDQSDFIEEEELKLFLQNFSA
SARALSDAETKAFLKAGDSDGDGKIGVDEFAAMVKA

_Gadus_morhua__(Atlantic_cod)_pvalb6
Sequence: GenBank XP_030198131
MAMTSILSADDIKKALNAFAAADSFDHKKFFEVVGLKALSAEEVKKAFLVLDADNSGFIEEEELKFMLKGFSS
SGRDLTDQETKAFLNAADKDGDGKIGVEEFTALVKE

_Gadus_morhua__(Atlantic_cod)_pvalb7
Sequence: GenBank XP_030208758
MAMTDLLKAEDIKKALDAFAVADSFDHRKFFELVGMRAMEADSVNKVFQALDVDASGYIEEEELKFVLKGFSE
EGRDLTDAETKKFLDAADKDGDGKIGMDEFQALVHE

_Gadus_morhua__(Atlantic_cod)_pvalb8
Sequence: GenBank XP_030203868
MSLSSILSADAIDNAIKDCQAPDSFCPKKFFQICGLSKKSPAEVKKAFAIMDNDASGFIEEEELKFFLQRFSP
GARVLTAAETKNFLSAADDDSDGKIGADEFQSLVLA

_Gadus_morhua__(Atlantic_cod)_pvalb9
Sequence: GenBank XP_030196391
MSLTSILSAEAIESAVKDCQEPNSFCYKKFFKLCGLTSKSPQEVKDVFSILDEDNSGYIEESELKFFLQRFVP
GARTLTEAEAKSFVSAADDDNDGKIGVEEFQTMVQS

_Gadus_morhua__(Atlantic_cod)_pvalb10
Sequence: GenBank XP_030196982
MAITDFLAASDITSAISACKAKDSFSAKMFFKTVGLSKKTAAEVEKVFKILDQDKSGFIEQDELQLFLQNFSK
GARNLTAAETRAFLLAGDSDGDGKIGWEEFSALVKSS

Ictalurus punctatus **(channel catfish)**

_Ictalurus_punctatus__(channel_catfish)_pvalb1
Sequence: GenBank XP_017348365
MAFAGVLADADVTAAIAACSAPDSFDYKAFFAKVGLSAKSADDVKKAFSIIDQDNSGFIEEDELKLFLQNFKS
GARALTDKETKAFLAAGDSDGDGKIGAEEFAALVKA

_Ictalurus_punctatus__(channel_catfish)_pvalb2
Sequence: GenBank NP_001187965
MAFAGILKDEDVAAAISACSAADSFNYKTFFAKVGLSAKSADEIKKAFFVIDQDKSGFIEEDELKLFLQNFSA
GARALTDAETKAFLAAGDSDGDGKIGVDEFAALVKA

_Ictalurus_punctatus__(channel_catfish)_pvalb3
Sequence: GenBank XP_017338080
MAFAGLLSDADITAALQACQAADSFNYKTFFSKVGLSGKSSDDVKKAFAVIDQDKSGFIEEDELKLFLQNFSA
GARALTDAETKAFLAAGDSDGDGKIGVDEFAALVKA

_Ictalurus_punctatus__(channel_catfish)_pvalb4
Sequence: GenBank AAO25757
MAFAGVLNDADITAALDACKADGSFNHKSFFTKVGLTGKSADDVKKAFAIIDQDKSGFIEEDELKLFLQNFKS
SARALTDAETKTFLKAGDTDGDGKIGVDEFASLVKA

_Ictalurus_punctatus__(channel_catfish)_pvalb5
Sequence: GenBank XP_017348340
MAFAGILKEEDIAAAVQACLDPGTFCHNSFFEQVGLFSNSNVDGEKVFKVLDQDKSGFIEEEELKLFLQNFVA
GARELTDAETKAFLAAGDSDGDGKIGMQEFCALLN

***Ictalurus_punctatus*_(channel_catfish)_pvalb6**
Sequence: GenBank XP_017312511
MAMSSILNADDIKKALDTFKAADSFDHKKFFEMVGLKAKSADDVKKAFHVLDADNSGFIEEEELKFVLKGFAT
NGRDLTDKETKAFLKAADKDGDGKIGVEEFAALVHE

***Ictalurus_punctatus*_(channel_catfish)_pvalb7**
Sequence: GenBank NP_001187453
MAMQDLLKADNIKKALDTFKAADSFDHKKFFELVGLTAMSAEDVKKVFRVLDVDASGFIEEDELKFVLKGFSK
DGRDLTDKETKAFLTAADKDGDGKIGIDEFQAIVHQ

***Ictalurus_punctatus*_(channel_catfish)_pvalb8**
Sequence: GenBank NP_001188142
MSLTSILSADAIDSAIKDCQAPESFNHKKFFQLCGLSKKTPQEIRTVFGILDNDGSGFIEEEELKFFLQRFSA
GARVLTERETKTLLSAADDDGDGMIGAEEFQSMVVS

***Ictalurus_punctatus*_(channel_catfish)_pvalb9**
Sequence: GenBank XP_017338324
MSLTDILSAEAIENAIKDCEAPDSFSYKKFFQVIGLTKKSPEEVREVFRLLDDNNSGFIEESELKFFLRRFVP
GARTLTENETKNFMSGSDGNSDGKIGVDEFQSMVLS

***Ictalurus_punctatus*_(Channel_catfish)_pvalb10**
Sequence: GenBank XP_017338318
MAFTGFLAASDISSAINACKAKDSFSPKTFFATLGLSKKTPSEIEKVFKMLDQDQSGFIEQDELQLFLQNFSK
GARTLTAAEVKAFLVAGDMDGDGKIGWDEFSALVNDS

***Lates calcarifer* (barramundi perch)**

***Lates_calcarifer*_(barramundi perch)_pvalb3**
Sequence: GenBank XP_018528002
MAFAGILNEADITAALAACQAADSFKHKDFFVKVGLAGKSDDDVKKAFAVIDQDKSGFIEEDELKLFLQNFSA
SARALTDAETKEFLKAGDSDGDGKIGVDEFAALVKV

***Lates_calcarifer*_(barramundi perch)_pvalb4**
Sequence: GenBank XP_018526999
MAFSNVLSDSDVAAALDGCKDAGTFDHKKFFSACGLSNKTSDDVKKAFAIIDQDKSGFIEEEELKLFLQNFKA
DARVLTDVETSTFLKAGDTDGDGKIGADEFTALVKP

***Onychostoma macrolepis* (a diploid cyprinid fish without an English name)**

***Onychostoma_macrolepis*_pvalb1**
Sequence: Modification of GenBank KAF4115537
MAVAAMLAAADVDAAIAACQAADSFNYKAFFAKVGLSAKSADDVKKAFAIIDQDNSGFIEEDELKLFLQNFKA
GARALTDKETKAFLTAGDSDGDGKIGAEEFAALVKA

***Onychostoma_macrolepis*_pvalb2**
Sequence: GenBank KAF4106695
MAFAGILKDEDVAAALKDCAAADSFNYKNFFAKVGLSAKTPDDIKKAFFVIDQDKSGFIEEDELKLFLQNFSA
GARALTDGETKAFLSAGDSDGDGKIGVDEFALLVKA

***Onychostoma_macrolepis*_pvalb3**
Sequence: GenBank KAF4106696
MAFAGILNDADITAALQACQAADSFNYKSFFAKVGLSAKTPDDIKKAFAVIDQDKSGFIEEDELKLFLQNFSA
GARALTDAETKAFLKAGDSDGDGKIGIDEFAVMVKA

***Onychostoma_macrolepis*_pvalb4**
Sequence: Modification of GenBank KAF4115536
MAFSGVLKDADITAALEACKAADSFNHKAFFAKVGLTSKSADDVKKAFAIIDQDKSGFIEEDELKLFLQNFKA
NARALTDAETKTFLKAGDSDGDGKIGVDEFAALVKS

***Onychostoma_macrolepis*_pvalb5**
Sequence: GenBank KAF4115538
MALAGHLKQDDIAAAIQACQAPGSFNYQTFFEHVGLAGKAASDGEKVFKALDQDKSGYIEKEELKRFLQNFCP
KARELTEAETNTLLAAGDSDGDGKIGMEEFCGLLK

***Onychostoma_macrolepis*_pvalb6**
Sequence: Modification of GenBank KAF4116378
MAMNSILNPDDIKKALDAFQAVDSFDPKRFFEMVGLKAKSAEDVKKAFHALDADNSGFIEEEELKFVFKHFAT
DGRDLSDKETKAFLQAADKDGDGKIGAEEFAALVRE

***Onychostoma_macrolepis*_pvalb7**
Sequence: GenBank KAF4097886
MAMKNLLKEDDIKKALDQFKAADSFDHKKFFDVVGLKALSAENVKLVFKALDVDASGFIEEEELKFVLKGFSA
DGRDLTDKETKAFLAAADKDGDGKIGIDEFEALVHE

***Onychostoma_macrolepis*_pvalb8**
Sequence: GenBank KAF4115535
MSLSSVLSADAIDSALKDCQAPDSFNPKKFFQLCGLTKKSPQEVKNVFNILDNDASGFIEEEELKFFLQRFSP
GARVLTDKETKGFLSAADDDSDGKIGADEFQAMVLS

***Onychostoma_macrolepis*_pvalb9**
Sequence: GenBank KAF4106697
MSLTSILSAEAIENAVKDCQAPDSFCYKKFFQLCGLSQKSPQEVKDVFRIIDEDNSGFIEEAELKFFLQRFFP
GARTLTEKEIKSLLTAADDDSDGRIGVDEFQTMVLS

***Onychostoma_macrolepis*_pvalb10**
Sequence: GenBank KAF4106694
MAVTDVLAASDISTAINACKAKDSFSPKTFFATVGLSKKSPPEIEKVFKMLDQDKSGFIEQDELQLFLQNFSK
GARALTAAETKAFLMAGDLDGDGKIGWEEFSALVNAS

***Oreochromis niloticus* (Nile tilapia)**

***Oreochromis_niloticus*_(Nile_tilapia)_pvalb1**
Sequence: GenBank XP_003452874
MAFAGVLSDADVKAALDGCAAADSFDYKKFFKASGLAAKTADDVKKAFKIIDQDNSGFIEEEELKLFLQNFSA
GARALTDKETKAFLSAGDSDGDGKIGIDEFAALVKA

***Oreochromis_niloticus*_(Nile_tilapia)_pvalb3**
Sequence: GenBank XP_013129480
MAFAGILTDADITAALAACQAADSFKHKDFFAKVGLAGKSADDIKKAFAVIDQDKSGFIEEDELKLFLQNFSA
SARALTDAETKTFLKAGDSDGDGKIGVDEFAALVKS

***Oreochromis_niloticus_*(Nile_tilapia)_pvalb4**
Sequence: GenBank XP_003452873
MAFAGVLTDADIAAALDASKDAGTFDHKKFFKSCGLHGKSADDIKKAFAIIDQDKSGFIEEDELKLFLQNFKS
GARVLNDAETKAFLGAGDTDGDGKIGADEFVVLVKAT

***Oreochromis_niloticus_*(Nile_tilapia)_pvalb5**
Sequence: GenBank XP_005457817
MAFAGMLSDEDIQAAVQACQAPGTFNFKSFFEQVGLTGLSEADRRKVFTVLDQDKSGYIEEEELKLFLQNFSP
GARELTVAETKALMAAGDKDSDGKIGIEEFCDLLK

***Oreochromis_niloticus_*(Nile_tilapia)_pvalb6**
Sequence: GenBank XP_003443184
MAMSSILNTDDIKKALDAFAVADSFDHKKFFEMVGLKSKSADDVKKVFTVLDADNSGFIEEEELKFVLKGFAK
DGRDLTDKETKAFLKAADKDGDGKIGVDEFTALVKE

***Oreochromis_niloticus_*(Nile_tilapia)_pvalb7**
Sequence: GenBank XP_003459401
MVMTDLLKPEEIKKALDAFAAETFDPKKFFEMVGMKAMSAENVKKVFQVLDVDGSGFIEEEELKFVLKGFSKD
GRDLTDAETKAFLTAADKDGDGKIGIDEFEALVHE

***Oreochromis_niloticus_*(Nile_tilapia)_pvalb8**
Sequence: GenBank XP_003452872
MSLSSILSADAIDSAIKDCQAPDSFCAKKFFQMCGLTKKSPQDIKKVFGILDNDASGFIEEEELKFFLQRFCP
GARVLTDKETKAFLSAADDDSDGMIGADEFQAMVLS

***Oreochromis_niloticus_*(Nile_tilapia)_pvalb9**
Sequence: GenBank XP_005448188
MSLTSILSADDIQNAIKDCEAPDTFCYKKFFKLCGLSSKTPKEVKDVFQILDEDNSGYIEHSELKFFLQRFIP
SARTLTDAETKSFISAADDDSDGKIGVEEFQSMVLS

Oryzias latipes (Japanese medaka)

***Oryzias_latipes_*(Japanese_medaka_drRD)_pvalb1**
Sequence: GenBank XP_004071520
MAFAGLNDADVKAALDGCAAADSFDYKKFFKACGLASKSADEVKKAFATIDQDNSGFIEEEELKLFLQNFSAG
ARALTDKETKTFLAAGDSDGDGKIGVDEFASLVKA

***Oryzias_latipes_*(Japanese_medaka_drRD)_pvalb3**
Sequence: GenBank XP_011486186
MAFAGVLNDADITAALAACKAADSFKHKEFFAKVGLAAKSADEIKKAFGIIDQDQSGFIEEEELKLFLQNFAA
GARVLTDAETKTFLKAGDSDGDGKIGVDEFAAMVKG

***Oryzias_latipes_*(Japanese_medaka_drRD)_pvalb4**
Sequence: GenBank XP_004071524
MAFAGVLNDADITKALDECKGADTFDYKKFFKTCGLAGKSADEVKKAFYIIDQDKSGFIEEDELKLFLQNFSA
GARVLSDGETKTFLKAGDVDGDGKIGADEFSALVKA

***Oryzias_latipes_*(Japanese_medaka_drRD)_pvalb5**
Sequence: GenBank XP_004071522
MAFAGMLSEENIKAAVQACQAPGTFDFRSFFAQVGLIDSSEADRRKVFTVLDRDESGYIEEEELKLFLQNFFP
GARELTGAETKALIAAGDKDCDGKIGMDEFCDLLE

***Oryzias_latipes_*(Japanese_medaka_drRD)_pvalb6**

Sequence: GenBank XP_023813489
MAMSSILNADDIKKALDAFAVADSFDHKKFFEILGLRAKSADDVKKVFTVLDADNSGYIEEEELKFVLKGFAK
DGRDLTDKETKAFLKAADKDGDGKIGVDEFTALVKE

***Oryzias_latipes_*(Japanese_medaka_drRD)_pvalb7**
Sequence: GenBank XP_023814662
MVMTDLLKPEEIKKALDAFAAETFDPKKFFEMIGMRAMTAENVKKVFQVLDVDASGYIEEDELKFVLKGFSKD
GRDLTDAETKAFLQAADKDGDGKIGIDEFEALVHE

***Oryzias_latipes_*(Japanese_medaka_drRD)_pvalb8**
Sequence: GenBank XP_020561005
MSLSSILSADAIDKAIKDCQAPDSFCPKKFFQICGLTQKSPQDIKKVFGILDNDASGYIEDEELKFFLQRFTP
SARVLTDKETKAFLVAADGDSDGRIGAAEFQTLVLS

***Oryzias_latipes_*(Japanese_medaka_drRD)_pvalb9**
Sequence: GenBank XP_004080202
MSLSSILSAEAIENAVKDCQAPDSFSFKKFSQLCGLSSKTPQEIRDVFQILDEDNSGYIEESELKFFLQRFLP
GARTLTEAETKSFISAADDNSDGRIGVDEFQAMVLS

Paramormyrops kingsleyae (Elephant fish)

***Paramormyrops_kingsleyae_*(elephant_fish)_pvalb1**
Sequence: GenBank XP_023691426
MALASVLKEADITAAMKDCQAADSFNYKSFFAKVGLSSKSADDVKKAFAIIDQDNSGFIEEDELKLFLQNFSA
GARVLTDGETKTFLAAGDSDGDGKIGVDEFAALVKA

***Paramormyrops_kingsleyae_*(elephant_fish)_pvalb2**
Sequence: GenBank XP_023664883
MAFAGILKEEDVAAALKDCAAPDSFNHKSFFAKVGLASKSADDVKKAFAVIDQDKSGFIEEDELKLFLQNFSA
SARALTDAETKTFLAAGDSDGDGKIGADEFAAIVKA

***Paramormyrops_kingsleyae_*(elephant_fish)_pvalb3**
Sequence: GenBank XP_023664886
MAFAGILNDADITAALQACQAADSFSHKAFFEKVGLSGKSADDVKKAFAVIDQDKSGFIEEDELKLFLQNFSA
GARVLTDTETKTFLKAGDTDGDGKIGIDEFTALVKS

***Paramormyrops_kingsleyae_*(elephant_fish)_pvalb4**
Sequence: GenBank XP_023691425
MAFAGILNDGDITAALDACKAADSFSYKAFFAKVGLSSKSADDVKKAFAIIDQDKSGFIEEDELKLFLQNFSA
SARALTDAETKTFLKAGDSDGDGKIGADEFAALVKA

***Paramormyrops_kingsleyae_*(elephant fish)_pvalb5(probably_pseudogene)**
Sequence: Ensembl dataset PKINGS_01 gene pvalb5 (only the last two exons look normal and we could not find proper sequences for the first two exons, despite that the available genomic sequence information seems to be of good quality)
LFLQSFSSGARKLTEADTKDLLRTGDQDGDSKIGMEEFCSLMKRSEEV

***Paramormyrops_kingsleyae_*(elephant fish)_pvalb6**
Sequence: GenBank XP_023672368
MAMNDILKAEDIKKALDAFKAADTFDHKKFFEMVGLKNKSTDDVKKAFLALDVDNSGFIEEEELKFVLKGFST
DGRDLTDKETKAFLQAADKDGDGKIGMEEFAALVLE

_Paramormyrops_kingsleyae__(elephant fish)_pvalb7
Sequence: GenBank XP_023670307
MAMKDLLKAEDIKKALDAFKAADTFDPLKFFDMVGLKAKSSEDVKKAFKALDVDNSGFIEEEELKFVLKGFAS
DGRDLTDKETKAFLALADKDGDGKIGAEEFEALVHQ

_Paramormyrops_kingsleyae__(elephant_fish)_pvalb8
Sequence: GenBank XP_023691172
MSLTSVLSADAIANALKECKAPDTFCHKKFFQTCGLTKKTSQEVKNVFRILDDDGSEFIEEDELQFFLQRFSP
SARVLTESETKKFMLALDEDNDGKIGIDEFEHAVLS

_Paramormyrops_kingsleyae__(elephant_fish)_pvalb9
Sequence: GenBank XP_023665037
MSLTSILSADAIENAIKDCQAPDSFCYKKFFQLCGLSSKTPQEVKDVFRCIDDDDSGFIEDEELKFFLQKFSP
SARLLTEKETQAILNACDDDGDGKIGVDEFQVLVLS

Plecoglossus altivelis (ayu)

_Plecoglossus_altivelis__(ayu)_pvalb2
Sequence: Encoded by GenBank genomic sequence BNHK01000005
MAFAGILKDDDVAAAIKACAAADSFKYKEFFAKVGLSAKSADDVKKAFYVIDQDKSGFIEEDELKLFLQNFAA
GARALTDAETKAFLKAGDADGDGMIGVDEFAALVKA

_Plecoglossus_altivelis__(ayu)_pvalb3
Sequence: Encoded by GenBank genomic sequence BNHK01000005
MAFAGVLKDDDITAALAACKAADSFKYKDFFAKVGLASKSADDVKKAFGIIDQDKSGFIEEEELKLFLQNFAA
GARALTDAETKAFLKAGDADGDGMIGMDEFAALVKS

Salmo salar (Atlantic salmon)

>_Salmo_salar__(Atlantic_salmon)_pvalb4_(Chr.3)
Sequence: GenBank NP_001117190
MACAHLCKEADIKTALEACKAADTFSFKTFFHTIGFASKSADDVKKAFKVIDQDASGFIEVEELKLFLQNFCP
KARELTDAETKAFLKAGDADGDGMIGIDEFAVLVKQ

>_Salmo_salar__(Atlantic_salmon)_pvalb5_(Chr.3)
Sequence: GenBank XP_014049628
MAFTGLLREEDIRAAVKACQAPSTFNFKLFFAQVGLTGKPEAEGQRVFRVLDQDQSGYIEEEELKLFLQNFSS
GARELTDAETRTLLAAGDRDGDGKIGMEEFCALLK

>_Salmo_salar__(Atlantic_salmon)_pvalb10_(Chr.28)
Sequence: GenBank NP_001134150
MALTDFLAASDITSAINACRANDSFSPKKFFAMVGLSKKSPPEIEKIFMILDQDKSGYIEQDELQLFLQNFSK
GARPLTAAETRAFLLAGDKDGDGKIGWDEFSNLVMSS

Sardinops melanostictus (crimson seabream)

>_Sardinops_melanostictus__(crimson_seabream)_pvalb4
Sequence: GenBank BAF98921
MALAGLVKEADITAALEACKAADSFDHKAFFHKVGMSGKSADELKKSFAIIDQDKSGFIEEEELKLFLQNFCK
KARALTDGETKNFLKAGDTDGDGKIGIDNFNHLVKH

***Scomber japonicus* (Pacific mackerel)**

>*Scomber_japonicus_*(Pacific_mackerel)_pvalb4
Sequence: GenBank ABJ98932
MAFASVLKDAEVTAALDGCKAAGSFDHKKFFKACGLSGKSTDEVKKAFAIIDQDKSGFIEEEELKLFLQNFKA
GARALSDAETKAFLKAGDSDGDGKIGIDEFAAMIKG

***Scophthalmus maximus* (turbot)**

***Scophthalmus_maximus_*(turbot)_pvalb1**
Sequence: GenBank
XP_035472137MSFAGLNAAEIKTALDGCAAADTFNYKKFFGACGLAKKSADDVKAAFQIIDQDNSGFIEEE
ELKLFLQNFSAGARALTDKETAEFLKAGDADGDGKIGIEEFANLVKE

***Scophthalmus_maximus_*(turbot)_ pvalb3**
Sequence: GenBank XP_035469716
MAFGKILSEAEITAALAACQAADSFKHKEFFAKVGLSAKSAEQIKEAFYVIDQDKSGFIEEEELKLFLQAFSA
SARALTDTETKEFLKAGDVDGDGKIGIDEFAEMVKQ

***Scophthalmus_maximus_*(turbot)_pvalb4**
Sequence: GenBank XP_035472139
MAFKGVLDDAKITAALEECKKPESFCHKKFFGTCGLSAKSPADVKAAFDIIDQDKSGFIEEDELKLFLQTFKA
GARSLTDTETKNLLKAGDTDNDGKIGADEFASMVKV

***Scophthalmus_maximus_*(turbot)_pvalb5**
Sequence: GenBank XP_035472138
MAFAGMLNEEDIKAAVQACQAPGTFNFKSFFAQVGLTGSTVADGEKVFTVLDQDRSGYIEEEELKLFLQNFSP
GARELTVAETKTLMASGDKDSDGKIGMEEFCDLLK

***Scophthalmus_maximus_*(turbot)_pvalb6**
Sequence: GenBank XP_035470782
MAMSSILNTDDIKKALHAFAAADSFDHKKFFEMVGLKAKPFDDVRKVFTVLDADNSGFIEEEELKYVLKGFAK
DGRDLTDKETQKFLKAADKDGDGKIGVDEFAALVKE

***Scophthalmus_maximus_*(turbot)_pvalb7**
Sequence: GenBank XP_035492367
MTMTDLLKAEEIKNALEAFAAETFDHRKFFEMVGMRAMSAENVKKVFQVLDVDGSGFIEEEELKFVLKGFSQE
GRDLTEAETKEFLKAADKDGDGKIGIDEFEAMVHE

***Scophthalmus_maximus_*(turbot)_pvalb8**
Sequence: GenBank XP_035472136
MSLSSILSADAIASAIKDCQAPDSFSPKKFFELSGLSKKSAQDVKKVFGILDNDASGFIEEDELKFFLQRFSP
GARVLTDKETKAFLSAADDDSDGRIGAEEFQAMILS

***Scophthalmus_maximus_*(turbot)_pvalb9**
Sequence: GenBank XP_035469767
MSLTSILSAEAIEKAVMDCQVPDSFCHKKFFSLCGLSSKTPKEVQDVFQILDDDKSGFIEESELKYFLQRFVP
GARTLTEAETKSFISAADGDSDGKVGVEEFRSLVQS

***Scophthalmus_maximus_*(turbot)_pvalb10**

Sequence: GenBank XP_035469765
MAVTDFLAASDVTLAINACKAKDSFSPKTFFKTVGLSKKSPKEIERVFKILDQDKSGFIEQDELQLFLQNFAK
GARPLTAAETRAFLMEGDSDGDGKIGWEEFSALVKST

***Sebastes umbrosus* (honeycomb rockfish)**

***Sebastes_umbrosus*_(honeycomb rockfish)_pvalb3**
Sequence: GenBank XP_037611559
MALAASLNAADITAALAACQGADTFKHKDFFGKVGLSAKSADDIKNAFKVIDQDKSGFIEEDELKLFLQNFSA
SARALTEAETKAFLKAGDSDGDGMIGMDEFAAMVKG

***Sebastes_umbrosus*_(honeycomb rockfish)_pvalb4**
Sequence: GenBank XP_037647877
MAFASVGLKDADIAAALDGCKDAGKFNHKTFFKTCGLSGKSADEVKKAFAIIDQDVSGFIEEEELKLFLQTFK
AGARALSDAETKEFLKAGDSDGDGKIGADEWAAMVKQ

***Seriola dumerili* (greater amberjack)**

***Seriola_dumerili*_(greater amberjack)_pvalb1**
Sequence: GenBank XP_022608146
MAFASVLKEAEVKAALDGCSAADSFDYKTFFKACGLAGKTAEDVKKAFAIIDQDNSGFIEEEELKLFLQNFSA
GARALTDKETKAFLAAGDSDGDGKIGVDEFAALVKA

***Seriola_dumerili*_(greater amberjack)_pvalb3**
Sequence: GenBank XP_022598222
MAFAGVLSDADITAALAACQAADSFKHKDFFAKVGLAAKSADDIKKAFAIIDQDKSGFIEEEELKLFLQNFSA
GARALTDAETKTFLKAGDSDGDGKIGVDEFAAMVKH

***Seriola_dumerili*_(greater amberjack)_pvalb4**
Sequence: GenBank XP_022608144
MAFKGVLNDAEITAALDACKNADSFDHKKFFKACGLAAKSADEVKKAFAIIDQDKSGFIEEEELKLFLQTFKA
GARALTDAETKTFLKAGDTDGDGKIGVDEFAAMVKH

***Seriola_dumerili*_(greater amberjack)_pvalb5**
Sequence: GenBank XP_022608145
MAFAGMLSDEDIKAAVKACQAPGTFNFKSFFTQVGLTGSSEADGKKVFTVLDQDKSGYIEEEELKLFLQNFSP
GARELTVAETKSLMAAGDRDGDGKIGMEEFCDLLK

***Seriola_dumerili*_(greater amberjack)_pvalb6**
Sequence: GenBank XP_022612726
MAMSSILNADDIKKALDAFAVADSFDHKKFFEMVGLKAKSCDDVKKVFTVLDADNSGFIEEEELKFVLKGFAK
DGRDLTDKETKAFLRAADKDGDGKIGVDEFAALVKE

***Seriola_dumerili*_(greater amberjack)_pvalb7**
Sequence: GenBank XP_022593842
MSMTDLLKAEEIKKALDAFAAETFDPKKFFEMVGMKAMTAENVKKVFQVLDVDGSGFIEEEELKFVLKGFSKE
GRDLTDAETKAFLQAADKDGDGKIGIDEFEAMVHE

***Seriola_dumerili*_(greater amberjack)_pvalb8**

Sequence: GenBank XP_022608123
MSLSSILSADAIDSAIKDCQAPDSFCPKKFFQLCGLTKKSPQDVKKVFAILDNDASGFVEEEELKFFLQRFSP
GARVLTDTETKAFLCAADDDSDGRIGADEFQAMVLS

***Seriola_dumerili_*(greater amberjack)_pvalb9**
Sequence: GenBank XP_022598299
MSLTSILSAEAIENAVKDCQAPDSFCYKKFFQLCGLSSKTPKEVKDVFQILDEDNSGFIEESELKYFLQRFIP
GARTLTEAETKSFISAADDDSDGRIGAEEFQTMVLS

***Seriola_dumerili_*(greater amberjack)_pvalb10**
Sequence: GenBank XP_022598278
MAITDFLAASDINSAINACKAKDSFSPKMFFKTVGLSKKSPTEIERVFKILDQDKSGYIEQDELQLFLQNFAK
GARPLTAAETRAFLLEGDSDGDGKIGWEEFSALVKSS

***Seriola lalandi* (Yellowtail amberjack)**

***Seriola_lalandi_*(Yellowtail_amberjack)_pvalb1**
Sequence: GenBank XP_023262140
MAFASVLKEAEVKAALDGCSAADSFNYKTFFKACGLSGKTAEDVKKAFAIIDQDNSGFIEEEELKLFLQNFSA
GARALTDKETKAFLAAGDSDGDGKIGVDEFAALVKA

***Seriola_lalandi_*(Yellowtail_amberjack)_pvalb3**
Sequence: GenBank XP_023285078
MAFAGVLSDADITAALAACQAADSFKHKDFFAKVGLAAKSADDIKKAFAIIDQDKSGFIEEEELKLFLQNFSA
GARALTDAETKAFLKAGDSDGDGKIGVDEFAAMVKH

***Seriola_lalandi_*(Yellowtail_amberjack)_pvalb4**
Sequence: GenBank XP_023250942
MAFKGVLNDADITAALDGCKNADSFDHKKFFKACGLAGKSADEVKKAFAIIDQDKSGFIEEEELKLFLQTFKA
GARALTDAETKTFLKAGDTDGDGKIGVDEFAAMVKH

***Seriola_lalandi_*(Yellowtail_amberjack)_ pvalb5**
Sequence: GenBank XP_023250944
MAFAGMLSDEDIKAAVKACQAPGTFNFKSFFTQVGLTGSSEADGKKVFTVLDQDKSGYIEEEELKLFLQNFSP
GARELTVDETKSLMAVGDRDGDGKIGMEEFCDLLK

***Seriola_lalandi_*(Yellowtail_amberjack)_ pvalb6**
Sequence: GenBank XP_023251550
MAMSSILNADDIKKALDAFAVADSFDHKKFFEMVGLKAKSCDDVKKVFTVLDADNSGFIEEEELKFVLKGFAK
DGRDLTDKETKAFLRAADKDGDGKIGVDEFAALVKE

***Seriola_lalandi_*(Yellowtail_amberjack)_pvalb7**
Sequence: GenBank XP_023286972
MSMTDLLKAEEIKKALDAFAAETFDPKKFFEMVGMKAMTAENVKKVFQVLDVDGSGFIEEEELKFVLKGFSKE
GRDLTDAETKAFLQAADKDGDGKIGIDEFETMVHE

***Seriola_lalandi_*(Yellowtail_amberjack)_pvalb8**

Sequence: GenBank XP_023250929
MSLSSILSADAIDSAIKDCQAPDSFCPKKFFQLCGLTKKSPQDVKKVFAILDNDASGFVEEEELKFFLQRFSP
GARVLTDTETKAFLCAADDDSDGRIGADEFQAMVLS

***Seriola_lalandi*_(Yellowtail_amberjack)_ pvalb9**
Sequence: GenBank XP_023285083
MSLTSILSAEAIENAVKDCQAPDSFCYKKFFQLCGLSSKTPKEVKDVFQILDEDNSGFIEESELKYFLQRFIP
GARTLTEAETKSFISAADDDSDGRIGAEEFQTMVLS

***Seriola_lalandi*_(Yellowtail_amberjack)_pvalb10**
Sequence: GenBank XP_023285072
MAITDFLAASDITSAINACKAKDSFSPKMFFKTVGLSKKSPTEIERVFKILDQDKSGYIEQDELQLFLQNFAK
GARPLTAAETRAFLLEGDSDGDGKIGWEEFSALVKSS

***Sparus aurata* (gilthead seabream)**

***Sparus_aurata*_(gilthead_seabream)_pvalb1**
Sequence: GenBank XP_030263619
MAFAGVLKDAEVKAALDGCSAADSFNYKSFFKACGLSGKSSDEVKKAFAIIDQDNSGFIEEDELKLFLQNFVA
SARALTDKETKAFLAAGDSDGDGKIGVDEFAALVKA

***Sparus_aurata*_(gilthead_seabream)_pvalb3**
Sequence: GenBank XP_030255186
MAFAGLLKEADITAALAACQAADSFKHKDFFAKVGLAGKSADDIKKAFAVIDQDKSGFIEEDELKLFLQNFSA
SARALTDGETKTFLKAGDSDGDGKIGVDEFAAMMKA

***Sparus_aurata*_(gilthead_seabream)_pvalb4**
Sequence: GenBank XP_030262240
MPFAGLTDADVAAALDGCKDAGSFDHKKFFAACGLSKKSGDDVKKAFAVIDQDKSGFIEEEELKLFLQNFKAG
ARALTDDETKKFLKAGDTDGDGKIGVDEFAAMVKV

***Sparus_aurata*_(gilthead_seabream)_pvalb5**
Sequence: GenBank XP_030263620
MAFAGMLSDEDVKAAVQACQTPGTFDFKTFFARVGLTGSSEADGKRVFTILDQDRSGYIEEEELKLFLQNFSP
EARELTVAETKALMAAGDKDGDGKIGLEEFCDILK

***Sparus_aurata*_(gilthead_seabream)_pvalb6**
Sequence: GenBank XP_030264822
MAMSSILNADDIKKALDAFAAADSFDYRKFFDMVGLKAKSSDDVKKVFKVLDADNSGFIEEEELKFVLKGFAK
DGRDLTDKETKAFLNAADKDGDGKIGVDEFVQLVTE

***Sparus_aurata*_(gilthead_seabream)_pvalb7**
Sequence: GenBank XP_030272489
MSMTDLLKAEEIKKALDAFAAETFDPKKFFEMVGLKAMTAENVKRVFKVLDVDGSGFIEEEELKYVLKGFSQE
GRDLTDDETRAFLKAADKDGDGKIGIDEFEVLVHE

***Sparus_aurata*_(gilthead_seabream)_pvalb8**
Sequence: GenBank XP_030262580
MSLSSILSADAIDSAIKDCQAPDTFCPKKFFQACGLTQKSPQDVKKAFAILDNDGSGFIEEEELKFFLQRFSP
GARVLTDKETKGFLAAADDDSDGRIGAEEFQAMVLS

***Sparus_aurata*_(gilthead_seabream)_pvalb9**

Sequence: GenBank XP_030255407
MSLTSILSAEAIEKAVKDCQAPDTFCYKKFFQLCGLSSKSPKEIKDVFQILDEDNSGFIEESELKYFLQRFVP
GARTLTEAETKRFISAADDDSDGRIGAEEFQTMVLS

***Sparus_aurata*_(gilthead_seabream)_pvalb10**
Sequence: GenBank XP_030255320
MAITDFLAASDITSAINACKGKDSFSPKMFFKTVGLSKKTPTEIEGVFKILDQDKSGFIEQDELQLFLQNFSK
GARTLTAAETRAFLLEGDSDGDGKIGWEEFSALVKSS

***Sparus_aurata*_(gilthead_seabream)_pvalb-like**
Sequence: GenBank XP_ XP_030256701
MEDDFRPQMKKMAVAMGASLTEQDIDHMPPGMTSQGIFHYSRFLEYMRQFKTSEQREEAIKKAFVMLDKDASG
YIEWNEIKYILSTVPTATPSAPLSDEEVEAMIQAVDTDGDGRISYREFSDMVKKEKELKK

***Takifugu rubripes* (Fugu)**

***Takifugu_rubripes*_(Fugu)_pvalb1**
Sequence: GenBank XP_011602188
MAFAGILNDADVKAALDSCSAADSFNYKSFFQKCGLAAKSPDDLKKAFAIIDQDNSGFIEEEELKLFLQNFSA
SARALSDKETKAFLAAGDSDGDGKIGVEEFAALMKA

***Takifugu_rubripes*_(Fugu)_pvalb3**
Sequence: GenBank XP_011602756
MAFSGILSDADISAALAACQAADSFNHKSFFAKVGLAGKSKEDIAKAFGIIDQDKSGFIEEDELKLFLQNFSA
SARALTDTETKAFLKAGDTDGDGKIGVDEFAALVK

***Takifugu_rubripes*_(Fugu)_pvalb4**
Sequence: Modification of GenBank XP_011602190
MAFSGTLKDADVTAALEACKADGTFEYKKFFATCGLASKSGEELKKAFEIIDQDKSGFIEEEELKLFLQNFKA
GARALTDAETSAFLKAGDTDGDGKIGAQEFSDMVKA

***Takifugu_rubripes*_(Fugu)_pvalb5**
Sequence: GenBank XP_003964605
MAFAEMLSDENINAAVQACHAPGTFDLDSFFTQVGLSGSSQDVGEKVFTVLDQDKSGYIEEEELKRFLQNFSP
GARELTVAETKAVMAVLDKDGDGKIGMEEFCDILK

***Takifugu_rubripes*_(Fugu)_pvalb6**
Sequence: GenBank XP_029692838
MAMSSILNNDDIKKALDAFAVADSFDHKKFFEMVGLKSKSPDEVKKVFTVLDADNSGFIEEEELKFVLKGFAK
EGRDLTDKETKAFLQAADKDGDGKIGVDEFTSLVKE

***Takifugu_rubripes*_(Fugu)_pvalb7**
Sequence: Encoded by Ensembl dataset fTakRub1.2 transcript
ENSTRUT00000061856
MLMTDLLKAEEIKTALEAFAGETFDPKKFFELVGLTAMSPESVKDVFRVLDVDGSGFIEEDELKYILKGFSKE
GRDLTDDETKAFLKAADKDGDGKIGIDEFEVMVHE

***Takifugu_rubripes*_(Fugu)_pvalb8**
Sequence: GenBank XP_003964606
MSLSSILPGDAIESAIKACQAPDSFCPKKFFQMCGLSKKSPQDIKKVFAILDNDGSGYIEEEELKFFLQRFCT
GARVLTDKETKGFMTLADDDSDGRIGADEFQAMVSS

Takifugu_rubripes_**(Fugu)_pvalb9**
Sequence: GenBank XP_003961387
MSLTSILSAEAIENAVKDCQAPESFCYKRFFQLCGLSSKTPKEIQDVFHILDDDDSGYFEESELKYFLQRFQP
GARTLTEAETKSFISAADDDCDGRIGVEEFQSMVLS

Takifugu_rubripes_**(Fugu)_pvalb10**
Sequence: GenBank XP_003961539
MAIANFLTASDITSAINACKAKDSFNPSVFFKTIGLSKKTPAEIETVFKILDQDKSGYIEQDELQLFLQNFSK
GARPLTAAETRAFLMEGDSDGDGKIGWDEFSALVKSS

Thunnus maccoyii (southern bluefin tuna)

***Thunnus maccoyii* (southern bluefin tuna) pvalb3**
Sequence: GenBank XP_042253153
MAFAGILTEADITAALAACQAADSFKYKDFFAKVGLAAKTPDDIKKAFAVIDQDKSGFIEEDELKLFLQNFSA
GARALTDAETKAFLMAGDSDGDGKIGIDEFAALVKA
